# Comparative Gene Expression Profiling in Human Cumulus Cells according to Ovarian Gonadotropin Treatments

**DOI:** 10.1155/2013/354582

**Published:** 2013-09-12

**Authors:** Said Assou, Delphine Haouzi, Hervé Dechaud, Anna Gala, Alice Ferrières, Samir Hamamah

**Affiliations:** ^1^Université Montpellier 1, UFR de Médecine, Montpellier, France; ^2^CHU Montpellier, Institute for Research in Biotherapy, Hôpital Saint-Eloi, INSERM U1040, 34295 Montpellier, France; ^3^ART-PGD Department, CHU Montpellier, Hôpital Arnaud de Villeneuve, 34295 Montpellier, France

## Abstract

In *in vitro* fertilization cycles, both HP-hMG and rFSH gonadotropin treatments are widely used to control human follicle development. The objectives of this study are (i) to characterize and compare gene expression profiles in cumulus cells (CCs) of periovulatory follicles obtained from patients stimulated with HP-hMG or rFSH in a GnRH antagonist cycle and (ii) to examine their relationship with *in vitro* embryo development, using Human Genome U133 Plus 2.0 microarrays. Genes that were upregulated in HP-hMG-treated CCs are involved in lipid metabolism (*GM2A*) and cell-to-cell interactions (*GJA5*). Conversely, genes upregulated in rFSH-treated CCs are implicated in cell assembly and organization (*COL1A1* and *COL3A1*). Interestingly, some genes specific to each gonadotropin treatment (*NPY1R* and *GM2A* for HP-hMG; *GREM1* and *OSBPL6* for rFSH) were associated with day 3 embryo quality and blastocyst grade at day 5, while others (*STC2* and *PTX3*) were related to *in vitro* embryo quality in both gonadotropin treatments. These genes may prove valuable as biomarkers of *in vitro* embryo quality.

## 1. Introduction

The gonadotropin-releasing hormone (GnRH) antagonist and agonist protocols with either highly purified human menopausal gonadotropin (HP-hMG) or recombinant FSH (rFSH) preparations are the most widely used protocols for controlled ovarian stimulation (COS) for both intracytoplasmic sperm injection (ICSI) and *in vitro* fertilization (IVF) [1–3]. At present, most of the mature oocytes retrieved after COS are capable of fertilization; however, only half of them develop into good embryos and only a few implants. There is increasing evidence that cumulus cells (CCs), which are somatic cells that surround the oocyte, play a crucial role in folliculogenesis and oocyte developmental competence acquisition [4, 5]. Several authors propose the use of CC gene expression as a noninvasive approach to predict oocyte aneuploidy, and oocyte competence, as well as embryo and pregnancy outcomes during assisted reproductive technology (ART) procedures [6–17]. Despite the recent molecular advances in the knowledge of human CCs, our understanding is far from complete. We believe that the characterization of the biology of these cells following COS might explain observed changes in *in vitro* embryo development. Several studies have compared the effects of HP-hMG and rFSH on oocyte and embryo quality, follicular fluid biochemical profile, and pregnancy rate [18–23]. However, their specific effects on the gene expression profile of individual CC samples have not been investigated. To date, only two such studies have been reported. They compared the gene expression profiles of pooled human granulosa cells (GCs) from periovulatory follicles of six patients in one study and eight patients in the other study. In both studies, the patients were treated with HP-hMG or rFSH in a GnRH agonist long protocol. Significant differences have been observed [24, 25]. The aims of the present study were (i) to compare the gene expression profiles of large cohorts of individual CCs isolated from periovulatory follicles of patients stimulated with HP-hMG or rFSH in a GnRH antagonist protocol and (ii) to determine the relationship between *in vitro* embryo development and expression profiles of CCs isolated from mature oocytes after COS.

## 2. Materials and Methods

### 2.1. Study Oversight

This research was approved by our Institutional Review Board. All patients provided their written informed consent for the use of CC samples for research.

### 2.2. Sample Collection and Treatment Cycle

**Figure 1:**
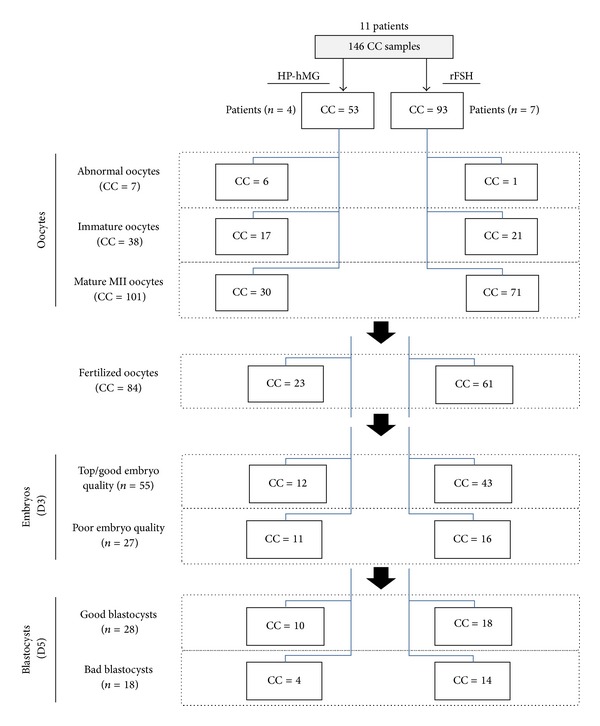
Distribution tree of cumulus cell (CC) samples and embryo outcome relative to the used COS protocol.

This study is a retrospective analysis of data from of a subgroup of eleven randomly selected patients, who participated in an open-label, assessor-blind, parallel groups, multicenter trial (ClinicalTrials.gov Identifier: NCT00884221) that was previously described [26]. CCs (*n* = 146) were collected from all oocytes retrieved from four patients treated with HP-hMG (Menopur, Ferring Pharmaceuticals) and seven patients treated with rFSH (Follitropin beta, Puregon; MSD) following a GnRH antagonist protocol (Ganirelix Acetate, Orgalutran; MSD), respectively. Stimulation with HP-hMG or rFSH was started at a dose of 150 IU/day (first 5 days of the COS protocol), and the patients' follicular response during stimulation was monitored by transvaginal ultrasound. The GnRH antagonist (daily dose of 0.25 mg) was initiated at day 6 and continued throughout the stimulation period. Transvaginal ultrasound echo guidance, FSH, LH, and estradiol levels were used to monitor the ovarian response. A single injection of 250 *μ*g human chorionic gonadotropin (hCG) (choriogonadotropin alfa, Ovitrelle; Merck Serono) was administered to induce the final follicular maturation when three or more follicles ≥17 mm in diameter were observed. Cumulus-oocyte-complexes were collected 36 h after hCG administration (day 0). Supplemental Table SI (see Supplementary Materials available online at http://dx.doi.org/10.1155/2013/354582) shows a summary of the patients' clinical features, end-of-stimulation data, and the number of retrieved oocytes/patients. All CCs were mechanically removed shortly after oocyte retrieval, washed in culture medium, and frozen immediately prior to total RNA extraction. MII oocytes were used for ICSI. All embryos and blastocysts were assessed daily by the embryologists until 5 days after oocyte retrieval. Embryo quality was assessed at 26 ± 2 and 92 ± 2 hours after insemination. On day 5, the quality evaluations of blastocysts consisted of expansion and hatching status, inner cell mass grading (grade A-C), and trophectoderm grading (grade A-C) [26–28]. Each CC sample included only CCs from a single oocyte. The number of CCs isolated from oocytes at GV, MI, and MII stages and the *in vitro* embryo outcome for the two patients' groups (HP-hMG or rFSH) are reported in (Figure 1). 

### 2.3. Cumulus Cells RNA Extraction

The RNeasy Micro kit (ref. 74004, Qiagen) was used to extract total RNA from each CCs sample (*n* = 146) according to the manufacturers' recommended protocols. The quantity and purity of the total RNAs were determined by using a NanoDrop ND-1000 spectrophotometer (NanoDrop ND-Thermo Fisher Scientific, Wilmington, DE, USA) and their integrity by using the Agilent 2100 Bioanalyzer (Agilent Technologies, Palo Alto, CA, http://www.agilent.com/). All RNA samples were stored at −80°C until the microarray experiments.

### 2.4. Preparation of cRNA and Microarray Hybridization

Total RNA (50 ng) was used to prepare cRNA (one cycle of amplification) using the Affymetrix 3′ IVT express protocol. An oligo-dT primer with a T7 promoter sequence was used to synthesize the first-strand cDNA. After generating the second strand, the complete cDNA was amplified by *in vitro* transcription (linear amplification) with a T7 RNA polymerase. The amplified RNA (aRNA) was generated and quantified by using a NanoDrop ND-1000 spectrophotometer (NanoDrop ND-Thermo Fisher Scientific, Wilmington, DE, USA), and biotinylated nucleotide analog was incorporated during *in vitro* transcription step. RNA from the GeneChip Eukaryotic Poly-A RNA Control Kit (Affymetrix, Santa Clara, CA), which contains mRNAs from *Bacillus subtilis* genes (*lys*, *phe*, *thr*, and *dap*), was amplified and labeled under the same conditions as positive controls. After fragmentation, the labeled antisense aRNA (15 *μ*g) was hybridized to HG-U133 Plus 2.0 GeneChip pan-genomic oligonucleotide arrays (Affymetrix) containing 54,675 sets of oligonucleotide probes (probeset) which correspond to *≈*25,000 unique human genes or predicted genes. Each cumulus cell sample was put individually on a microarray chip. Microarray experiments were performed in DNA microarray platform of our Institute of Research in Biotherapy at the Montpellier University Hospital.

### 2.5. Data Processing and Gene Expression Profile Analysis

After image processing with the Affymetrix GeneChip Operating 1.4 software (GCOS), the CEL files were analyzed using the Affymetrix Expression Console Software v1.3.1 and normalized with the MAS5.0 algorithm by scaling each array to a target value (TGT) of 100 using the global scaling method to obtain an intensity value signal for each probe set. This algorithm also determines whether a gene is expressed with a defined confidence level or not (“detection call”). This “call” can either be “present” (when the perfect match probes are significantly more hybridized than the mismatch probes, *P* < 0.04), “marginal” (for *P* values of >0.04 and <0.06) or “absent” (*P* > 0.06). Gene annotation was performed using NetAffx (http://www.affymetrix.com/, March 2009). A first selection of microarray data was based on the detection call (present in at least 50% of the CC samples of each group). Then, the Significant Analysis of Microarrays (SAM) (http://www-stat.stanford.edu/~tibs/SAM/) with the Wilcoxon test and sample label permutation (*n* = 300) was used to identify genes of which expression varied significantly between the HP-hMG and rFSH CC samples. The lists of significant genes (fold change, FC ≥1.5 and false discovery rate, FDR ≤5%) as well as common genes were analyzed using the Ingenuity Pathway Analysis (IPA) software (http://www.ingenuity.com/) to identify the biological functions that were specific of each CC group and in common between the two treatments, respectively. Only annotations with significant *P* value (*P* < 0.05) were considered. Then, the SAM analysis (FC ≥1.5, FDR ≤5%) was used to link gonadotropin-specific genes in CCs or those that are irrespective of gonadotropin treatment to subsequent embryo outcome at day 3 (top, good embryo versus poor) or day 5 (good blastocyst versus bad). Hierarchical clustering analyses based on the expression levels of the differentially expressed genes were performed by using the Cluster and Treeview software packages [29]. Box-and-whisker plots depicted the comparisons of the expression levels of candidate genes carried out using SPSS 12.0 (SPSS, Chicago, IL, USA) software.

### 2.6. Microarray Data Validation by Quantitative RT-PCR

Quantitative RT-PCR was performed to validate the expression of selected genes identified as differentially expressed between the two CC groups by using mRNAs from HP-hMG (*n* = 4) and rFSH (*n* = 4) CC samples as described in [30]. The primer sequences are shown in (Supplementary data, Table SII). Briefly, cDNA was reverse transcribed (RT) following the manufacturer's instructions using 500 ng of amplified RNA in a 20 *μ*L reaction volume that included Superscript II (ref. 18064-014, Invitrogen), oligo-dT primer, dNTP mixture, MgCl_2_, and RNase inhibitor. Quantitative PCR was performed using a LightCycler 480 apparatus with the LC480 SYBR Green I Master kit (Roche Diagnostics, Mannheim, Germany) and 2 *μ*L of diluted cDNA (1/25) and 0.6 mMol primers in a total volume of 10 *μ*L. After 10 min of activation at 95°C, cycling conditions were 10 s at 95°C, 30 s at 63°C, and 1 s at 72°C for 45 cycles. Gene expression levels were normalized to the housekeeping gene glyceraldehyde 3-phosphate dehydrogenase (*GAPDH*), because its expression was stable between all CC groups using the following formula 100/2^ΔΔCt^, where ΔΔCt = ΔCt unknown  −  ΔCt positive control. 

### 2.7. Statistical Analysis

Statistical analyses were performed with SPSS 12.0 software. A repartition difference between sample groups was considered significant when the Kruskal-Wallis nonparametric test and Wilcoxon test gave a *P* value ≤0.05. For q-RT-PCR, a statistical analysis was performed with the GraphPad InStat software (Mann-Whitney *U* test; GraphPad, San Diego, CA). A value of *P* ≤ 0.05 was considered to be statistically significant.

## 3. Results

### 3.1. Identification of Differentially Expressed Genes in Human CCs following Stimulation with HP-hMG or rFSH

**Figure 2:**
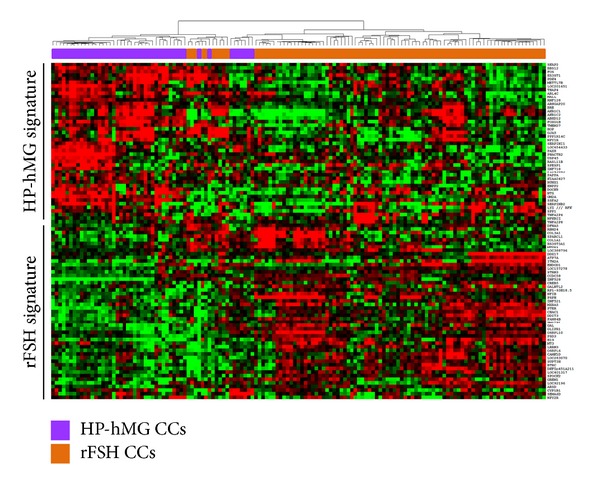
Gene expression patterns of the HP-hMG and rFSH CC samples. Supervised hierarchical clustering of CC samples based on the 94 genes that are differentially expressed between the two treatment groups (HP-hMG and rFSH). We can see a distinct signature in each CCs category. The color intensity indicates the level of gene expression (red for upregulated genes and green for downregulated genes).

A first selection is based on the detection call between all the CC samples from patients stimulated with HP-hMG or rFSH delineated 9,899 genes. Then, using SAM, 94 genes that significantly differentiated between HP-hMG and rFSH CCs were identified. Among them, 45 and 49 genes were upregulated in HP-hMG and rFSH CC samples, respectively (fold-change, FDR, and annotation are in Tables 1 and 2). The HP-hMG CC list included genes implicated in lipid metabolism such as *GM2A *(x2.3, FDR = 0), *AKR1C1* (x1.5, FDR = 0), *AKR1C2* (x1.6, FDR = 0.005), and in cell-to-cell interaction like *GJA5* (x1.9, FDR = 0), *NTS *(x1.8, FDR = 0.005), *FOS* (x1.6, FDR = 0), and* NPY1R *(x2.1, FDR = 0), *NPY2R *(x1.6, FDR = 0). Conversely, the rFSH CC list was significantly enriched in genes important for cellular assembly and organization such as *COL3A1* (x2, FDR = 0.015), *COL1A1* (x1.5; FDR = 0), *MT3* (x1.5; FDR = 0), and *CAMK1D* (x1.5; FDR = 0). Other genes of the rFSH list are members of the tumour necrosis factor (*TNF*) family such as *TNFAIP6* (x1.7; FDR = 0.01) and *TNFAIP8 *(x1.6, FDR = 0.005). The clustering based on these 94 genes segregates the majority of the HP-hMG (85%) from the rFSH CC samples (Figure 2). RT-qPCR validated the differential expression of some of these genes (Supplementary data, Figure SI). 

### 3.2. Common Transcriptional Gene Profile in HP-hMG/rFSH CCs

In view of few differences between the two gonadotropin treatments, we examined the list of genes in common to HP-hMG and rFSH groups (list of 9,805 genes; see Supplementary data, Table SIII). We used IPA software to explore the specific functional properties of this common molecular signature. Estrogen receptor signaling (83 genes) (*P* value = 8.17*E* − 08) was one of the top canonical pathways related to this molecular signature. On the other hand, the top network involving 35 genes was articulated around the “cell death and survival, DNA replication, recombination, and repair” functions. The detailed list of genes involved in this network can be found in (Supplementary data, Table SIV). Interestingly, the most common HP-hMG/rFSH genes were associated with multiple signaling pathways including *FGF* signaling (*FGFR and GRB2*), *IGF* signaling (*IGF1R* and *IGFBP3*), *EGF* signaling (*EGFR* and *MAPK1*), and *PDGF* signaling (*PDGFRA* and *PDGFD*). It is important to note that no difference was observed in the mRNA CC level between treatments for receptors (*LHCGR* and *BMPR2*), aromatase (*CYP19A1*), cytochrome *P450* (*CYP11A1*), or steroidogenic genes (*StAR*, *HSD3B2*, *ACVR1*, *ACVR1B*, *INHBC*, and *INHBB*).

### 3.3. Relationship between the HP-hMG or rFSH CC Expression Profiles and **In Vitro ** Embryo Development

**Figure 3:**
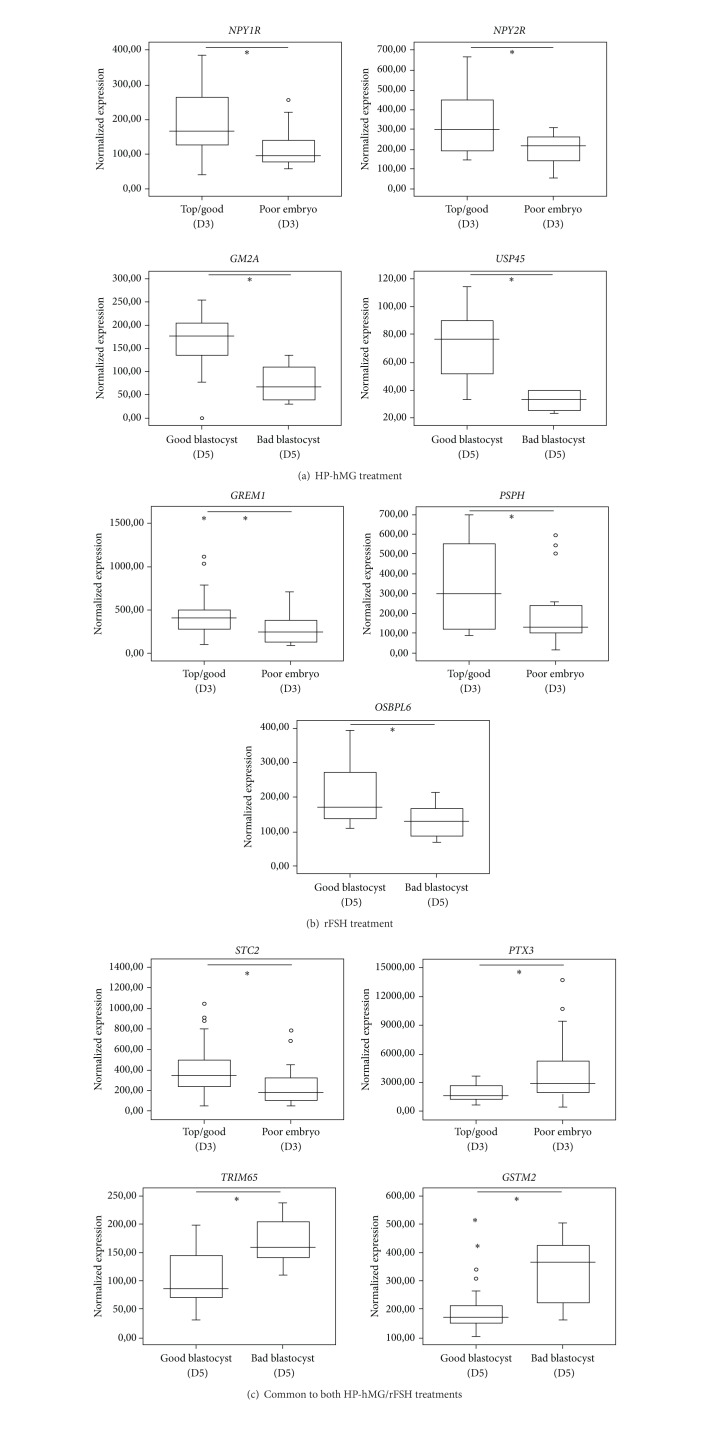
Gonadotropin gene expression associated with *in vitro* embryo development. (a) and (b) Box-and-whisker plots comparing the expression level of gonadotropin-specific gene in CCs from oocytes that developed into top/good quality embryos (*n* = 43 in the rFSH and *n* = 12 in the HP-hMG group) or poor quality embryos (*n* = 16 in the rFSH and *n* = 11 in the HP-hMG group) and into good blastocysts (*n* = 18 in the rFSH and *n* = 10 in the HP-hMG group) or bad blastocysts (*n* = 14 in the rFSH and *n* = 4 in the HP-hMG group). (c) Box-and-whisker plots comparing the expression level of gonadotropin common genes in CCs from oocytes that developed into top/good quality embryos (*n* = 55 CCs) or poor quality embryos (*n* = 27 CCs) and into good blastocysts (*n* = 28 CCs) or bad blastocysts (*n* = 18 CCs). The signal intensity for each gene is shown on the *y*-axis as arbitrary units determined by the Affymetrix GCOS software. *A significant difference with FDR ≤0.05.

Of the 146 CC samples, 101 were isolated from MII mature oocytes which underwent ICSI. In the HP-hMG group, 77% of injected oocytes were fertilized and 61% achieved blastocyst stage at day 5. In the rFSH group, these values were, respectively, 86% and 52%. Fertilized MII oocytes (*n* = 23 in the HP-hMG and *n* = 61 in the rFSH group) were divided into oocytes that developed into (i) top/good quality (52% in the HP-hMG and 70% in the rFSH group, no significant difference (*ε* = 1.65)) or poor quality embryos at day 3; and then into (ii) good (AA and AB) (43% for the HP-hMG and 29% for the rFSH group, no significant difference (*ε* = 1.28)) or bad grade (AC, BC, CC, and CB) blastocysts at day 5 (Figure 1). Then, the transcription profile of the cumulus cell samples isolated from these 101 MII oocytes was evaluated relative to day 3 embryo quality and blastocyst grading at day 5. In the HP-hMG group, *NPY1R *(x1.58, FDR = 0.0004) and *NPY2R* (x1.67, FDR = 0.0004) upregulation was observed in CCs isolated from MII oocytes that developed into top/good day 3 embryos, whereas *GM2A* (x2.10, FDR = 0.0005) and *USP45* (x2.32, FDR = 0.0005) were upregulated in cumulus cells from MII oocytes with good blastocyst grading (Figure 3(a)). After rFSH treatment, upregulation of *GREM1* (x1.59, FDR = 0) and *PSPH *(x1.6, FDR = 0) was significantly associated with top/good quality day 3 embryos; *OSBPL6 *(x1.59, FDR = 0) upregulation was found in CCs from oocytes that developed into good blastocyst at day 5 (Figure 3(b)). In the two gonadotropin groups, *PTX3 *(x-1.81, FDR = 0) downregulation and *STC2* (x1.76, FDR = 0) upregulation were observed in CCs isolated from MII oocytes that developed into top/good day 3 embryos, whereas *TRIM65* (x-1.62, FDR = 0) and *GSTM2* (x-1.67, FDR = 0) expressions were downregulated in CCs associated with good blastocyst grading (Figure 3(c)).

### 3.4. CC mRNA Content and **In Vitro ** Blastocyst Outcome at Day 5

**Figure 4:**
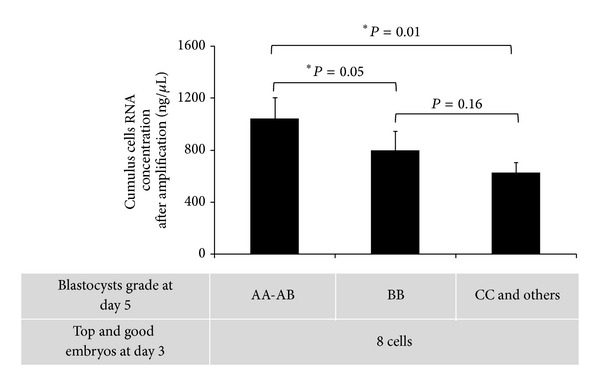
Relationship between amount of amplified CCs mRNA and blastocyst quality. Three groups of blastocysts (good, intermediary, or bad quality) were obtained from top and good 8-cell embryos at day 3. The Kruskal-Wallis test was used to indicate that at least one of the groups is different from the others (*P* = 0.011, Kruskal-Wallis test), and the Wilcoxon test was used to establish whether group AA-AB is significantly different from group BB and/or group CC. *A significant difference in the concentration of amplified CC mRNA between two groups of blastocysts. CC samples (*n* = 17) were from oocytes that developed in top and good 8-cell embryos at day 3. AA-AB: good blastocyst grades (*n* = 7); BB: intermediary blastocyst grades (*n* = 6); CC and others: bad blastocyst grades (*n* = 4). Bars represent the mean ± SEM.

Independently of the type of gonadotropin treatment used, the relation between amplified mRNA content of CC samples and *in vitro* blastocyst development at day 5 was also investigated. Seventeen CC samples, isolated from MII oocytes that developed into top quality 8-cell embryos at day 3, were selected and divided in three groups: (i) CCs from MII oocytes that developed into good quality (grade AA-AB, *n* = 7), (ii) intermediary (grade BB, *n* = 6), and (iii) bad (grade CC and others, *n* = 4) blastocysts. The amount (mean ± SEM) of amplified mRNA from CCs from MII oocytes leading to good quality blastocysts was 1044.28 ± 159.18 ng/*μ*L. This value decreased to 796.66 ± 150 ng/*μ*L in the intermediary group and to 627.50 ± 76.25 ng/*μ*L in the bad blastocyst grade group (Figure 4). 

## 4. Discussion

Following global genomic assessment of 146 human CCs transcriptome under HP-hMG and rFSH treatments, the present study revealed a small but significant distinct molecular signature of 94 genes between the two treatments, suggesting that these treatments impact differentially the CC gene expression profile. This may be accounted for by the differences in the origin of the two pharmaceutical preparations. More precisely, overexpression of genes involved in the metabolism of lipids such as *GM2A*, *AKR1C1* and *AKR1C2*, as well as genes related to the intercellular signaling (*GJA5* and *FOS*) was observed in the CCs treated with HP-hMG, while genes involved in “cellular assembly and organization” (*COL1A1*, *COL3A1*, *MT3*, *TNFAIP6*, and* TNFAIP8*) were overexpressed in the rFSH CCs. Each of these functions plays a central role in oocyte maturation and/or oocyte competence [31–33]. Indeed, the metabolism of lipids represents the main energy source for protein synthesis during oocyte nuclear maturation and early embryo development [34, 35]. Simultaneously, adequate communication between oocyte and CCs and appropriate assembly and organization of the CC matrix are required for both oocyte maturation and competence [36–38]. Most of the genes, identified in the present investigation as differentially expressed in CCs treated with HP-hMG and rFSH, were reported for the first time, except for *TNFAIP6 *and *GJA5* (connexin 40) which have been previously identified as potential markers of oocyte competence in CCs from bovine preovulatory follicles [39] and biomarker of oocyte maturation in canine cumulus-oocyte complexes matured *in vitro*, respectively [38].

Furthermore, the comparison of our data with the two other transcriptomic studies comparing the same gonadotropin treatment in granulosa cells (GCs) using the GnRH agonist long protocols [24, 25] indicates that *GM2* ganglioside activator is upregulated in HP-hMG CCs (this study) and rFSH GCs [24]. *GM2A* is known to play an important role in the hydrolysis of phospholipids or small glycolipids [40]. In addition, among the 9 common genes of our study and the one by Brannian et al. [25], six genes (*ATP7A*, *BTRC*, *LRRN3*, *STRN3*, *PTER*, and *SUPT3*) are upregulated in both CCs and GCs after rFSH treatment; one (*H19*) was upregulated in both rFSH CCs and HP-hMG GCs and the two others (*SERPINI1* and *SSFA2*) in HP-hMG CCs and rFSH. The use of different GnRH analogs might explain these discrepancies, but we cannot exclude the possibility that gonadotropin stimulation might have different effects on CCs and GCs. More investigations are required to address this issue.

On the other hand, we reported an important common CC molecular signature revealing the preservation of numerous growth factor signaling between the two types of treatments including the *IGF*, *PDGF*, *FGF*, and *EGF* pathways (See Figure SIII). These signaling pathways have been previously reported to play a central role in the control of the intrafollicular androgen/estrogen ratio for the *IGF* members [41], in angiogenesis and embryo development for the *FGF* and *PDGF* members [42] and in oocyte maturation for the members of the *EGF* family [43–45]. The interactions between these signaling pathways in CCs under COS will be a precious itinerary to explore in future works in order to complete the oocyte competence puzzle. 

Another important finding of this study is that the mRNA level for key genes involved in ovulation process including hormonal receptors (*LHCGR* and *BMPR2*) and regulators of steroidogenesis (*StAR*, *HSD3B2*, *Activins*, and *Inhibins*) was comparable in the HP-hMG and rFSH CC groups. This suggests a similar potency of the two protocols to induce hormonal receptors and similar estrogenic capacity of the CC samples stimulated by HP-hMG and rFSH. This is in line with several studies reporting that CCs *in vitro* were able to secrete estradiol during COCs culture from patients undergoing stimulated cycles, probably as a consequence of the action of gonadotropins [46].

We also identified a significant relationship between some CC genes that were specifically upregulated following stimulation with HP-hMG or rFSH and *in vitro* embryo development. In the HP-hMG group, upregulation of *NPY1R* and *NPY2R* in CCs was associated with top/good embryo quality at day 3. *NPY* modulates steroid production through *NPY* receptors [47] and plays a role in human ovarian steroidogenesis directly at the level of the granulosa cells of the follicles in the early stage of luteinization [48, 49]. Additionally, the association of ubiquitin specific protease 45 (*USP45*) with good blastocyst quality suggests the requirement of proteasomal activity in HP-hMG-treated CCs. Proteasomal activity has been reported to have multiple functions in CCs expansion, in oocyte meiosis, and in the modification of cumulus-oocyte communication [50]. 

In the rFSH group, upregulation of gremlin 1 (*GREM1*) in CCs was associated with top/good embryo quality at day 3 and *OSBPL6* upregulation with good blastocyst grading at day 5. Only CC expression of *GREM1*, a member of the bone morphogenic protein (*BMP*) antagonist family, has been reported as positively correlated with embryo quality [7, 12, 51]. The regulation of *BMP* through *GREM1* is thought to contribute to CCs expansion and therefore to the final maturation of oocytes [52]. The gene *OSBPL6* codes for the oxysterol binding protein-like-6 receptor. Oxysterols, which bind to this receptor, are potent modulators of expression of cholesterol synthesis in human granulosa cells [53]. Recently, Watanabe et al. [54] reported that variation in cholesterol contents in cumulus-oocyte complexes during *in vitro* maturation of porcine oocytes affected their ability to be fertilized, suggesting that, under rFSH regime, cholesterogenesis at a nearby site of oocyte growth and maturation might also be involved in *in vitro* blastocyst outcome.

On the other hand, we also identified CC genes associated with day 3 embryo quality and blastocyst grading at day 5, independently of the type of gonadotropins. Among these genes, we report for the first time the expression of *STC2*, *GSTM2*, and *TRIM65*, as well as *PTX3* which has been shown in previous studies to either be associated with fertilization rate [55] or to have no relationship with high-quality embryo on day 3 [51]. A possible reason for higher stanniocalcin 2 (*STC2*) expression in the CCs isolated from MII oocytes that developed into top/good day 3 is the modulation of the angiogenic [56] or steroidogentic pathways [57] or principal processes in ovarian function [58–60]. Conversely, we observed an increased expression of *GSTM2 *and *TRIM65* in CCs from oocytes that developed into bad blastocyst grading. *GSTM2* and *TRIM65* play a role in the protection against lipid peroxidation [61] and in DNA repair [62] respectively, suggesting an increase in cellular resistance against oxidative stress and damaged DNA. The implications of these genes, at the CC level, deserve to be addressed in future studies in order to understand their function in follicular growth. 

Furthermore, independently of the type of gonadotropin treatment, we found an association between blastocyst grading at day 5 and the amount of amplified mRNA in CC samples from MII mature oocytes with comparable top/good embryo quality at day 3. Lower mRNA values were detected in CCs from MII oocytes that developed into bad blastocysts as compared to CC samples from oocytes that developed into intermediary or good quality blastocysts at day 5. This suggests that CCs surrounding an incompetent oocyte are less transcriptionally active.

These results are in line with our previously published data showing a general reduction in transcriptomic activity of CCs associated with poor oocyte competence and negative clinical outcome [6]. 

## 5. Conclusion

Analysis of the microarray data of CCs from patients, who underwent GnRH-antagonist COS, highlights a significant difference in the gene expression profile of CCs following treatment with HP-hMG or rFSH. Components of signaling pathways (the *EGF*, *IGF*, *FGF*, and *PDGF* cascades) were conserved in CCs under the two gonadotropin stimulation regimens. Some genes specific to each gonadotropin treatment or commonly expressed in both groups were associated with *in vitro* embryo development. Moreover, independently of the gonadotropin preparation used, the amount of amplified mRNA in each CC was associated with blastocyst grading at day 5. These genes may prove valuable as biomarkers of *in vitro* embryo quality and can be useful for understanding the biology of stimulation.

## Supplementary Material

Supplementary Material available online includes: (i) Baseline clinical characteristics, end-of-stimulation data and number of oocytes retrieved from each patient following controlled ovarian stimulation with HP-hMG or rFSH. (ii) Primer pairs used for validation of the array data by qRT-PCR. (iii) List of the 9,805 common genes expressed in both HP-hMG and rFSH CC samples. (iv) Validation by qRT-PCR of some of the genes that were differentially expressed in HP-hMG and rFSH CC samples. (v) Top-ranked networks evidenced in common HP-hMG/rFSH signature by Ingenuity Pathway software. (vi) Detailed list of the genes presented in the network. (vii) The major signaling pathways that occur in CCs following the gonadotropin (HP-hMG and rFSH) stimulation.

## Figures and Tables

**Table 1:** List of genes that were significantly upregulated in HP-hMG CCs compared with rFSH CCs.

Gene name	Gene title	Probesets	Fold change	FDR (%)
PHACTR2	Phosphatase and actin regulator 2	244774_at	2.9	0
GM2A	GM2 ganglioside activator	235678_at	2.3	0
LOC654433	*Homo sapiens*, clone IMAGE:4826696, mRNA	228425_at	2.2	0
LOC201651	Similar to esterase/N-deacetylase (EC 3.5.1.-), 50 K hepatic-rabbit	1569582_at	2.1	0
PAX8	Transcribed locus, moderately similar to XP_375099.1 hypothetical protein LOC283585 (*Homo sapiens*)	227474_at	2.1	0
NPY1R	Neuropeptide Y receptor Y1	205440_s_at	2.1	0
GJA5	Gap junction protein, alpha 5, 40 kDa (connexin 40)	226701_at	1.9	0
FOXG1B	Forkhead box G1B	206018_at	1.9	0
SPP1	Secreted phosphoprotein 1	209875_s_at	1.9	0.58
NTS	Neurotensin	206291_at	1.8	0.58
THAP4	THAP domain containing 4	220417_s_at	1.8	0
SPESP1	Sperm equatorial segment protein 1	229352_at	1.8	0.58
SEMA6D	Sema domain, transmembrane domain (TM), and cytoplasmic domain, (semaphorin) 6D	233882_s_at	1.8	0.58
DOCK8	Dedicator of cytokinesis 8	225502_at	1.8	0.58
SERPINB2	Serine (or cysteine) proteinase inhibitor, clade B (ovalbumin), member 2	204614_at	1.7	0.58
PPP1R14C	Protein phosphatase 1, regulatory (inhibitor) subunit 14C	226907_at	1.7	0
CTIF	CBP80/20-dependent translation initiation factor	243090_at	1.7	0
SSFA2	Sperm-specific antigen 2	236207_at	1.7	0
HS3ST1	Heparan sulfate (glucosamine) 3-O-sulfotransferase 1	205466_s_at	1.7	0
CYP1B1	Cytochrome P450, family 1, subfamily B, polypeptide 1	202437_s_at	1.7	0
TMEM37	Transmembrane protein 37	1554485_s_at	1.6	0
BBS12	Hypothetical protein FLJ35630	229603_at	1.6	0
AKR1C2	Aldo-keto reductase family 1, member C2	211653_x_at	1.6	0.58
MALL	BENE protein	209373_at	1.6	0
NPY2R	Neuropeptide Y receptor Y2	210729_at	1.6	0
METTL7B	Hypothetical protein MGC17301	227055_at	1.6	0
RNF128	Ring finger protein 128	219263_at	1.6	0
ARL4C	ADP-ribosylation factor-like 7	202207_at	1.6	0
PAPPA	Pregnancy-associated plasma protein A, pappalysin 1	240450_at	1.6	0
USP45	Ubiquitin-specific protease 45	224441_s_at	1.6	0
FOS	v-fos FBJ murine osteosarcoma viral oncogene homolog	209189_at	1.6	0
PDK4	Pyruvate dehydrogenase kinase, isozyme 4	225207_at	1.6	0
ZNF718	Hypothetical protein FLJ90036	1553269_at	1.6	0
ARHGAP20	Rho GTPase activating protein 20	228368_at	1.5	0
FLJ43663	CDNA FLJ26188 fis, clone ADG04821	238619_at	1.5	0
HOP	Homeodomain-only protein	211597_s_at	1.5	0
ENPP2	Ectonucleotide pyrophosphatase/phosphodiesterase 2 (autotaxin)	209392_at	1.5	2.95
LYZ	Lysozyme (renal amyloidosis)	213975_s_at	1.5	1.05
SKAP2	src family associated phosphoprotein 2	204361_s_at	1.5	0
ABHD12	Chromosome 20 open reading frame 22	228124_at	1.5	0
RUNX1	Runt-related transcription factor 1	236114_at	1.5	0
AKR1C1	Aldo-keto reductase family 1, member C2	216594_x_at	1.5	0
BRE	Brain and reproductiveorgan-expressed(TNFRSF1A modulator)	211566_x_at	1.5	0
SERPINI1	Serine (or cysteine) proteinase inhibitor, clade I (neuroserpin), member 1	205352_at	1.5	0
RASL11B	RAS-like, family 11, member B	219142_at	1.5	0

**Table 2:** List of genes that were significantly upregulated in rFSH CCs compared with HP-hMG CCs.

Gene name	Gene title	Probesets	Fold change	FDR (%)
ITM2A	Integral membrane protein 2A	202746_at	4.2	0
H19	H19, imprinted maternally expressed transcript (nonprotein coding)	224646_x_at	3.8	0
PSPH	Phosphoserine phosphatase	205048_s_at	2.4	0
GAL	Galanin	214240_at	2.4	0
ZNF528	Zinc finger-like	232315_at	2.3	0
NFKBIZ	Nuclear factor of kappa light polypeptide gene enhancer in B-cell inhibitor, zeta	223217_s_at	2.2	4.73
FAM84B	Breast cancer membrane protein 101	225864_at	2	0
COL3A1	Collagen, type III, alpha 1 (Ehlers-Danlos syndrome type IV, autosomal dominant)	211161_s_at	2	1.53
DKFZp451A211	DKFZp451A211 protein	1556114_a_at	1.8	0
SPARCL1	SPARC-like 1 (mast9, hevin)	200795_at	1.8	0
PTER	Phosphotriesterase related	222798_at	1.8	0
NFIB	Nuclear factor I/B	213032_at	1.8	0
MXRA5	Adlican	209596_at	1.8	0
GALNTL2	UDP-N-acetyl-alpha-D-galactosamine:polypeptide N-acetylgalactosaminyltransferase-like 2	228501_at	1.8	0
SUPT3H	Suppressor of Ty 3 homolog (*S*. cerevisiae)	211106_at	1.7	0
DDX17	DEAD (Asp-Glu-Ala-Asp) box polypeptide 17 /// DEAD (Asp-Glu-Ala-Asp) box polypeptide 17	208151_x_at	1.7	4.15
TNFAIP6	Tumor necrosis factor, alpha-induced protein 6	206026_s_at	1.7	1.05
MTUS1	Mitochondrial tumor suppressor 1	212096_s_at	1.7	4.73
RP1-93H18.5	Similar to RIKEN cDNA A630077B13 gene, RIKEN cDNA 2810048G17	229390_at	1.7	0
LOC92196	Hypothetical LOC92196 (uncharacterized)	229290_at	1.6	0
LOC401317	Hypothetical LOC402472 (uncharacterized)	242329_at	1.6	0
CHAC1	Hypothetical protein MGC4504	219270_at	1.6	0
STRN3	Striatin, calmodulin binding protein 3	215505_s_at	1.6	0
OSBPL10	Oxysterol binding protein-like 10	219073_s_at	1.6	0
GLIPR1	HIV-1 rev binding protein 2	214085_x_at	1.6	0
BTRC	Beta-transducin repeat containing E3 ubiquitin protein ligase	237862_at	1.6	0
TNFAIP8	Tumor necrosis factor, alpha-induced protein 8	208296_x_at	1.6	0.54
PMAIP1	Phorbol-12-myristate-13-acetate-induced protein 1	204286_s_at	1.6	0
RBM24	RNA binding motif protein 24	235004_at	1.6	1.53
LOC388796	Hypothetical LOC388796 (uncharacterized)	65588_at	1.6	0
LOC157278	*Homo sapiens*, clone IMAGE:5285282, mRNA (uncharacterized)	238716_at	1.6	0
GREM1	Gremlin 1	218468_s_at	1.6	0
OSBPL6	Oxysterol binding protein-like 6	223805_at	1.6	0
CREB5	cAMP responsive element binding protein 5	205931_s_at	1.5	0
CAMK1D	Calcium/calmodulin-dependent protein kinase ID	235626_at	1.5	0
CCDC58	Hypothetical LOC131076	235244_at	1.5	0
LRRN3	Leucine-rich repeat neuronal 3	209840_s_at	1.5	0
HS3ST3A1	Heparan sulfate (glucosamine) 3-O-sulfotransferase 3A1	219985_at	1.5	0
ARSD	Arylsulfatase D	232423_at	1.5	0
ENDOD1	KIAA0830 protein	212570_at	1.5	0
ZNF521	Zinc finger protein 521	226676_at	1.5	0
DFNA5	Deafness, autosomal dominant 5	203695_s_at	1.5	0
PSD3	Pleckstrin and Sec7 domain containing 3	203354_s_at	1.5	0
LOC283070	Hypothetical protein LOC283070 (uncharacterized)	226382_at	1.5	0
COL1A1	Collagen, type I, alpha 1	1556499_s_at	1.5	0
SPOCK2	Sparc/osteonectin, cwcv and kazal-like domains proteoglycan (testican) 2	202523_s_at	1.5	0
ATP7A	ATPase, Cu++ transporting, alpha polypeptide (Menkes syndrome)	205197_s_at	1.5	0
MT3	Metallothionein 3 (growth inhibitory factor (neurotrophic))	205970_at	1.5	0
DDIT3	DNA-damage-inducible transcript 3	209383_at	1.5	0
