# Supplementary material for: Comparative Gene Expression Profiling in Human Cumulus Cells according to Ovarian Gonadotropin Treatments
Source: Biomed Res Int. 2013 Sep 12;2013:354582. doi: 10.1155/2013/354582 (PMC3786475; doi:10.1155/2013/354582)
Supplement: Supplementary file 1 — Supplementary Material available online includes: (i) Baseline clinical characteristics, end-of-stimulation data and number of oocytes retrieved from each patient following controlled ovarian stimulation with HP-hMG or rFSH. (ii) Primer pairs used for validation of the array data by qRT-PCR. (iii) List of the 9,805 common genes expressed in both HP-hMG and rFSH CC samples. (iv) Validation by qRT-PCR of some of the genes that were differentially expressed in HP-hMG and rFSH CC samples. (v) Top-ranked networks evidenced in common HP-hMG/rFSH signature by Ingenuity Pathway software. (vi) Detailed list of the genes presented in the network. (vii) The major signaling pathways that occur in CCs following the gonadotropin (HP-hMG and rFSH) stimulation. [file 354582.f1.doc]

Supplementary Figures

Supplementary Tables

Supplementary Table SIII: List of the 9,805 common genes expressed in both HP-hMG and rFSH CC samples

| **Probe Set ID** | **Gene Title** | **Gene Symbol** | **Representative Public ID** | **UniGene ID** | **Chromosomal Location** |
| --- | --- | --- | --- | --- | --- |
| 91952_at | hypothetical protein BC002926 | LOC90379 | AI363375 | Hs.443636 | chr19p13.12 |
| 91816_f_at | ring finger and KH domain containing 1 | RKHD1 | C18318 | Hs.436495 | chr19p13.3 |
| 90610_at | leucine-rich repeats and calponin homology (CH) domain containing 4 | LRCH4 | AI654857 | Hs.125742 | chr7q22 |
| 89948_at | Chromosome X open reading frame 45 | CXorf45 | AI743331 | Hs.272814 | chr20q13.12 |
| 81811_at | Programmed cell death 6 | PDCD6 | AI744451 | Hs.481452 | chr5p15.33 |
| 78495_at | hypothetical protein DKFZp762P2111 | DKFZp762P2111 | R61320 |  | chr7q36.1 |
| 78383_at | Doublesex and mab-3 related transcription factor 1 /// NADH dehydrogenase (ubiquinone) 1 beta subcomplex, 6, 17kDa | DMRT1 | AI150117 | Hs.533976 | chr9p21 |
| 74694_s_at | rabaptin, RAB GTPase binding effector protein 2 | RABEP2 | AA907940 | Hs.170253 | chr16p11.2 |
| 71933_at | wingless-type MMTV integration site family, member 6 | WNT6 | AI218134 | Hs.29764 | chr2q35 |
| 66053_at | heterogeneous nuclear ribonucleoprotein U-like 2 | HNRPUL2 | AI738452 | Hs.406377 | chr11q12.3 |
| 65635_at | endo-beta-N-acetylglucosaminidase | FLJ21865 | AL044097 | Hs.29288 | chr17q25.3 |
| 65585_at | family with sequence similarity 86, member B1 | FAM86B1 | AA527515 | Hs.458413 | chr8p23.1 |
| 65521_at | ubiquitin-conjugating enzyme E2D 4 (putative) | UBE2D4 | W74577 | Hs.415497 | chr7p13 |
| 65472_at | Ubiquitin specific peptidase 39 | USP39 | AI161338 |  |  |
| 65133_i_at | zinc finger, HIT type 4 | ZNHIT4 | AI862454 | Hs.410786 | chr2p13.1 |
| 64883_at | motile sperm domain containing 2 | MOSPD2 | AI744083 | Hs.190043 | chrxp22.2 |
| 64440_at | interleukin 17 receptor C | IL17RC | AI560217 | Hs.129959 | chr3p25.3 |
| 64438_at | hypothetical protein FLJ22222 | FLJ22222 | W19668 | Hs.436237 | chr17q25.3 |
| 64432_at | chromosome 12 open reading frame 47 | C12orf47 | W05463 | Hs.333120 | chr12q24.12 |
| 635_s_at | protein phosphatase 2, regulatory subunit B (B56), beta isoform | PPP2R5B | L42374 | Hs.75199 | chr11q12-q13 |
| 632_at | glycogen synthase kinase 3 alpha | GSK3A | L40027 | Hs.435970 | chr19q13.2 |
| 63009_at | SHQ1 homolog (S. cerevisiae) | SHQ1 | AI188402 | Hs.528650 | chr3p13 |
| 61732_r_at | intraflagellar transport 74 homolog (Chlamydomonas) | IFT74 | AI610355 | Hs.275906 | chr9p21.2 |
| 61297_at | CASK interacting protein 2 | CASKIN2 | AL037338 | Hs.274408 | chr17q25.1 |
| 60815_at | hypothetical protein MGC13098 | MGC13098 | AA601208 | Hs.488152 | chr7p13 |
| 60474_at | chromosome 20 open reading frame 42 | C20orf42 | AA469071 | Hs.180479 | chr20p12.3 |
| 60084_at | cylindromatosis (turban tumor syndrome) | CYLD | AI453099 | Hs.386952 | chr16q12.1 |
| 59999_at | hypoxia-inducible factor 1, alpha subunit inhibitor | HIF1AN | W37897 | Hs.14595 | chr10q24 |
| 59705_at | selenocysteine lyase | SCLY | AA911739 | Hs.512606 | chr2q37.3 |
| 59697_at | RAB15, member RAS onocogene family | RAB15 | AA582932 | Hs.21349 | chr14q23.3 |
| 59631_at | thioredoxin reductase 3 | TXNRD3 | AI247566 | Hs.20030 | chr3p13-q13.33 |
| 59433_at | hypothetical protein LOC729137 | LOC729137 | N32185 | Hs.535223 | chrxp22.33 |
| 58994_at | coiled-coil and C2 domain containing 1A | CC2D1A | AI689402 | Hs.269592 | chr19p13.12 |
| 58696_at | exosome component 4 | EXOSC4 | AL039469 | Hs.343589 | chr8q24.3 |
| 58367_s_at | zinc finger protein 419 | ZNF419 | AA429615 | Hs.98593 | chr19q13.43 |
| 57739_at | dead end homolog 1 (zebrafish) /// similar to dead end homolog 1 /// similar to dead end homolog 1 /// similar to dead end homolog 1 | DND1 | AI949010 | Hs.519625 | chr5q31.3 |
| 57703_at | SUMO1/sentrin specific peptidase 5 | SENP5 | N32782 | Hs.478826 | chr3q29 |
| 57540_at | ribokinase | RBKS | AI823980 | Hs.11916 | chr2p23.3 |
| 57539_at | zinc finger, CCCH-type with G patch domain | ZGPAT | AA535065 | Hs.11900 | chr20q13.3 |
| 57532_at | dishevelled, dsh homolog 2 (Drosophila) | DVL2 | AW016304 | Hs.118640 | chr17p13.2 |
| 57516_at | zinc finger protein 764 | ZNF764 | AA746290 | Hs.132227 | chr16p11.2 |
| 57082_at | low density lipoprotein receptor adaptor protein 1 | LDLRAP1 | AA169780 | Hs.184482 | chr1p36-p35 |
| 56829_at | NIK and IKK{beta} binding protein | NIBP | H61826 | Hs.416372 | chr8q24.3 |
| 56256_at | SID1 transmembrane family, member 2 | SIDT2 | AA150165 | Hs.410977 | chr11q23.3 |
| 56197_at | phospholipid scramblase 3 | PLSCR3 | AI783924 | Hs.512653 | chr17p13.1 |
| 55872_at | KIAA1196 protein | GM632 | AI493119 | Hs.158654 | chr20q13.33 |
| 55692_at | engulfment and cell motility 2 | ELMO2 | W22924 | Hs.199334 | chr20q13 |
| 55662_at | chromosome 10 open reading frame 76 | C10orf76 | H27225 | Hs.9444 | chr10q24.32 |
| 55616_at | per1-like domain containing 1 | PERLD1 | AI703342 | Hs.91668 | chr17q12 |
| 55583_at | dedicator of cytokinesis 6 | DOCK6 | AI198543 | Hs.131592 | chr19p13.2 |
| 55065_at | MAP/microtubule affinity-regulating kinase 4 | MARK4 | AL120554 | Hs.118843 | chr19q13.3 |
| 53968_at | integrator complex subunit 5 | INTS5 | AI869988 | Hs.458390 | chr11q12.3 |
| 53202_at | chromosome 7 open reading frame 25 | C7orf25 | AA402435 | Hs.214247 | chr7p14-p11.2 |
| 53076_at | xylosylprotein beta 1,4-galactosyltransferase, polypeptide 7 (galactosyltransferase I) | B4GALT7 | AI040029 | Hs.54702 | chr5q35.2-q35.3 |
| 52940_at | single immunoglobulin and toll-interleukin 1 receptor (TIR) domain | SIGIRR | AA085764 | Hs.433036 | chr11p15.5 |
| 52741_at | chromosome 14 open reading frame 172 | C14orf172 | AI962879 | Hs.339834 | chr14q32.32 |
| 52731_at | hypothetical protein FLJ20294 | FLJ20294 | AI359466 | Hs.278241 | chr11p11.2 |
| 52651_at | collagen, type VIII, alpha 2 | COL8A2 | AI806793 | Hs.353001 | chr1p34.2 |
| 52285_f_at | centrosomal protein 76kDa | CEP76 | AW002970 | Hs.236940 | chr18p11.21 |
| 52169_at | protein kinase LYK5 | LYK5 | AI302185 | Hs.534614 | chr17q23.3 |
| 52164_at | chromosome 11 open reading frame 24 | C11orf24 | AA065185 | Hs.303025 | chr11q13 |
| 52159_at | HemK methyltransferase family member 1 | HEMK1 | W93807 | Hs.345319 | chr3p21.3 |
| 52078_at | chromosome 1 open reading frame 160 | C1orf160 | AI828080 | Hs.321214 | chr1p36.11 |
| 52005_at | WIZ zinc finger | WIZ | AA422049 | Hs.442138 | chr19p13.1 |
| 51200_at | hypothetical protein FLJ20850 | FLJ20850 | AI744084 | Hs.30783 | chr19p13.11 |
| 51176_at | cofactor required for Sp1 transcriptional activation, subunit 8, 34kDa | CRSP8 | AA131335 | Hs.374262 | chr9q34.1-q34.3 |
| 51146_at | GDNF family receptor alpha 1 /// phosphatidylinositol glycan anchor biosynthesis, class V | GFRA1 | AA203365 | Hs.259605 | chr1p36.11 |
| 50376_at | zinc finger protein 444 | ZNF444 | AI278629 | Hs.24545 | chr19q13.43 |
| 50314_i_at | chromosome 20 open reading frame 27 | C20orf27 | AI761506 | Hs.274422 | chr20p13 |
| 50277_at | golgi associated, gamma adaptin ear containing, ARF binding protein 1 | GGA1 | AW001443 | Hs.405689 | chr22q13.31 |
| 49878_at | peroxisomal biogenesis factor 16 | PEX16 | AA523441 | Hs.100915 | chr11p11.2 |
| 49679_s_at | Matrix metallopeptidase 24 (membrane-inserted) | MMP24 | AA243774 | Hs.212581 | chr20q11.22 |
| 49329_at | kelch-like 22 (Drosophila) | KLHL22 | N38751 | Hs.351563 | chr22q11.21 |
| 49327_at | sirtuin (silent mating type information regulation 2 homolog) 3 (S. cerevisiae) | SIRT3 | AI492888 |  |  |
| 48825_at | inhibitor of growth family, member 4 | ING4 | AA887083 | Hs.108183 | chr12p13.31 |
| 48659_at | invasion inhibitory protein 45 /// zinc finger protein 557 | RP5-1077B9.4 | W60802 | Hs.8595 | chr1p36.22 |
| 48580_at | CXXC finger 1 (PHD domain) | CXXC1 | U55777 | Hs.180933 | chr18q12 |
| 48117_at | coiled-coil domain containing 101 | CCDC101 | AA160496 | Hs.110407 | chr16p11.2 |
| 48031_r_at | chromosome 5 open reading frame 4 | C5orf4 | H93077 | Hs.10235 | chr5q31-q32 |
| 47608_at | tight junction associated protein 1 (peripheral) | TJAP1 | AI697401 | Hs.9741 | chr6p21.1 |
| 47571_at | zinc finger protein 236 | ZNF236 | AA004757 | Hs.369037 | chr18q22-q23 |
| 47560_at | latrophilin 1 | LPHN1 | AI525402 | Hs.94229 | chr19p13.2 |
| 47530_at | chromosome 9 open reading frame 156 | C9orf156 | AA748492 | Hs.9196 | chr9q22.33 |
| 47105_at | dihydrouridine synthase 2-like, SMM1 homolog (S. cerevisiae) | DUS2L | AA886893 | Hs.155049 | chr16q22.1 |
| 47083_at | chromosome 7 open reading frame 26 | C7orf26 | AI280108 | Hs.81057 | chr7p22.1 |
| 46665_at | sema domain, immunoglobulin domain (Ig), transmembrane domain (TM) and short cytoplasmic domain, (semaphorin) 4C | SEMA4C | AI949392 | Hs.7188 | chr2q11.2 |
| 46256_at | splA/ryanodine receptor domain and SOCS box containing 3 | SPSB3 | AA522670 | Hs.7247 | chr16p13.3 |
| 46167_at | tetratricopeptide repeat domain 4 /// chromosome 1 open reading frame 175 | TTC4 /// C1orf175 | W22690 | Hs.412482 | chr1p31.3 |
| 46142_at | transmembrane protein 112 | TMEM112 | AI003763 | Hs.6382 | chr16p13.3 |
| 45828_at | hypothetical protein FLJ10241 | FLJ10241 | AI768100 | Hs.351099 | chr19q13.2 |
| 45749_at | family with sequence similarity 65, member A | FAM65A | AA400206 | Hs.152717 | chr16q22.1 |
| 45714_at | host cell factor C1 regulator 1 (XPO1 dependent) | HCFC1R1 | AA436930 | Hs.423103 | chr16p13.3 |
| 45653_at | potassium channel tetramerisation domain containing 13 | KCTD13 | AW026481 | Hs.106817 | chr16p11.2 |
| 44822_s_at | mesoderm induction early response 1, family member 2 | MIER2 | AW003889 | Hs.101891 | chr19p13.3 |
| 44702_at | synapse defective 1, Rho GTPase, homolog 1 (C. elegans) | SYDE1 | R77097 | Hs.528701 | chr19p13.12 |
| 44696_at | TBC1 domain family, member 13 | TBC1D13 | AA915989 | Hs.3376 | chr9q34.11 |
| 44669_at | hypothetical protein LOC644096 | LOC644096 | N31716 | Hs.356460 |  |
| 44654_at | glucose 6 phosphatase, catalytic, 3 | G6PC3 | AI669655 | Hs.294005 | chr17q21.31 |
| 44617_at | 2-oxoglutarate and iron-dependent oxygenase domain containing 2 | OGFOD2 | AI431902 | Hs.425608 | chr12q24.31 |
| 44563_at | WD repeat domain 79 | WDR79 | AI858000 | Hs.437460 | chr17p13.1 |
| 44111_at | vacuolar protein sorting 33 homolog B (yeast) | VPS33B | AI672363 | Hs.327378 | chr15q26.1 |
| 43977_at | transmembrane protein 161A | TMEM161A | AI660497 | Hs.26570 | chr19p13.11 |
| 43934_at | G protein-coupled receptor 137 | GPR137 | AA479495 | Hs.25274 | chr11cen-q22.3 |
| 41329_at | SCY1-like 3 (S. cerevisiae) | SCYL3 | AI458463 | Hs.435560 | chr1q24.2 |
| 41220_at | septin 9 | septin 9 | AB023208 | Hs.288094 | chr17q25 |
| 41160_at | methyl-CpG binding domain protein 3 | MBD3 | AC005943 | Hs.178728 | chr19p13.3 |
| 41037_at | TEA domain family member 4 | TEAD4 | U63824 | Hs.94865 | chr12p13.2-p13.3 |
| 40612_at | dopey family member 1 | DOPEY1 | AB029040 | Hs.348982 | chr6q15 |
| 40524_at | protein tyrosine phosphatase, non-receptor type 21 | PTPN21 | X79510 | Hs.437040 | chr14q31.3 |
| 40465_at | DEAD (Asp-Glu-Ala-Asp) box polypeptide 23 | DDX23 | AF026402 | Hs.130098 | chr12q13.12 |
| 40446_at | PHD finger protein 1 /// deleted in lung and esophageal cancer 1 | PHF1 | AL021366 | Hs.166204 | chr6p21.3 |
| 40420_at | serine/threonine kinase 10 | STK10 | AB015718 | Hs.16134 | chr5q35.1 |
| 40149_at | SH2B adaptor protein 1 | SH2B1 | AL049924 | Hs.15744 | chr16p11.2 |
| 40020_at | cadherin, EGF LAG seven-pass G-type receptor 3 (flamingo homolog, Drosophila) | CELSR3 | AB011536 | Hs.55173 | chr3p24.1-p21.2 |
| 39966_at | chondroitin sulfate proteoglycan 5 (neuroglycan C) | CSPG5 | AF059274 | Hs.45127 | chr3p21.3 |
| 39891_at | zinc finger protein 710 | ZNF710 | AI246730 | Hs.323053 | chr15q26.1 |
| 39835_at | SET binding factor 1 | SBF1 | U93181 | Hs.112049 | chr22q13.33 |
| 39817_s_at | chromosome 6 open reading frame 108 | C6orf108 | AF040105 | Hs.109752 | chr6p21.1 |
| 39650_s_at | pecanex-like 2 (Drosophila) | PCNXL2 | AB007895 | Hs.520606 | chr1q42.2 |
| 39548_at | neuronal PAS domain protein 2 | NPAS2 | U77970 | Hs.321164 | chr2q11.2 |
| 39402_at | interleukin 1, beta | IL1B | M15330 | Hs.126256 | chr2q14 |
| 39248_at | aquaporin 3 (Gill blood group) | AQP3 | N74607 | Hs.234642 | chr9p13 |
| 38710_at | OTU domain, ubiquitin aldehyde binding 1 | OTUB1 | AL096714 | Hs.473788 | chr11q13.1 |
| 38703_at | aspartyl aminopeptidase | DNPEP | AF005050 | Hs.258551 | chr2q35 |
| 38671_at | plexin D1 | PLXND1 | AB014520 | Hs.301685 | chr3q21.3 |
| 38269_at | protein kinase D2 | PRKD2 | AL050147 | Hs.205431 | chr19q13.3 |
| 38241_at | butyrophilin, subfamily 3, member A3 | BTN3A3 | U90548 | Hs.167741 | chr6p21.3 |
| 38157_at | dom-3 homolog Z (C. elegans) | DOM3Z | AF059252 | Hs.153299 | chr6p21.3 |
| 38069_at | chloride channel 7 | CLCN7 | Z67743 | Hs.80768 | chr16p13 |
| 38043_at | family with sequence similarity 3, member A | FAM3A | X55448 | Hs.289108 | chrxq28 |
| 37996_s_at | dystrophia myotonica-protein kinase | DMPK | L08835 | Hs.898 | chr19q13.3 |
| 37860_at | zinc finger protein 337 | ZNF337 | AL049942 | Hs.213735 | chr20p11.21 |
| 37802_r_at | family with sequence similarity 63, member B | FAM63B | AL049226 |  |  |
| 37577_at | Rho GTPase activating protein 19 | ARHGAP19 | U79256 |  |  |
| 37549_g_at | parathyroid hormone-responsive B1 | PTHB1 | U87408 | Hs.79340 | chr7p14 |
| 37425_g_at | coiled-coil alpha-helical rod protein 1 | CCHCR1 | AB029343 | Hs.110746 | chr6p21.3 |
| 37408_at | mannose receptor, C type 2 | MRC2 | AB014609 | Hs.7835 | chr17q23.3 |
| 37152_at | peroxisome proliferator-activated receptor delta | PPARD | L07592 | Hs.519689 | chr6p21.2-p21.1 |
| 37079_at | YDD19 protein | YDD19 | U82319 | Hs.289008 | chr6q22.1 |
| 37005_at | neuroblastoma, suppression of tumorigenicity 1 | NBL1 | D28124 | Hs.439671 | chr1p36.13-p36.11 |
| 36994_at | ATPase, H+ transporting, lysosomal 16kDa, V0 subunit c | ATP6V0C | M62762 | Hs.389107 | chr16p13.3 |
| 36936_at | tissue specific transplantation antigen P35B | TSTA3 | U58766 | Hs.404119 | chr8q24.3 |
| 36920_at | myotubularin 1 | MTM1 | U46024 | Hs.386205 | chrxq28 |
| 36865_at | angel homolog 1 (Drosophila) | ANGEL1 | AB018302 | Hs.497448 | chr14q24.3 |
| 36830_at | mitochondrial intermediate peptidase | MIPEP | U80034 | Hs.68583 | chr13q12 |
| 36552_at | hypothetical protein | DKFZP586P0123 | AL080220 | Hs.376803 | chr11q13.4 |
| 36499_at | cadherin, EGF LAG seven-pass G-type receptor 2 (flamingo homolog, Drosophila) | CELSR2 | D87469 | Hs.57652 | chr1p21 |
| 36475_at | glycine C-acetyltransferase (2-amino-3-ketobutyrate coenzyme A ligase) | GCAT | Z97630 | Hs.54609 | chr22q13.1 |
| 36129_at | RUN and TBC1 domain containing 1 | RUTBC1 | AB007857 | Hs.273138 | chr17p13.3 |
| 36084_at | cullin 7 | CUL7 | D38548 | Hs.420639 | chr6p21.1 |
| 36019_at | serine/threonine kinase 19 | STK19 | L26260 | Hs.444 | chr6p21.3 |
| 35626_at | N-sulfoglucosamine sulfohydrolase (sulfamidase) | SGSH | U30894 | Hs.31074 | chr17q25.3 |
| 35617_at | mitogen-activated protein kinase 7 | MAPK7 | U29725 | Hs.150136 | chr17p11.2 |
| 35179_at | beta-1,3-glucuronyltransferase 3 (glucuronosyltransferase I) | B3GAT3 | AB009598 | Hs.201362 | chr11q12.3 |
| 35150_at | CD40 molecule, TNF receptor superfamily member 5 | CD40 | X60592 | Hs.504816 | chr20q12-q13.2 |
| 34868_at | Smg-5 homolog, nonsense mediated mRNA decay factor (C. elegans) | SMG5 | AB029012 | Hs.434979 | chr1q21.2 |
| 34858_at | potassium channel tetramerisation domain containing 2 | KCTD2 | D79998 | Hs.4935 | chr17q25.1 |
| 34764_at | leucyl-tRNA synthetase 2, mitochondrial | LARS2 | D21851 | Hs.438303 | chr3p21.3 |
| 34697_at | low density lipoprotein receptor-related protein 6 | LRP6 | AF074264 | Hs.210343 | chr12p11-p13 |
| 34478_at | RAB11B, member RAS oncogene family | RAB11B | X79780 | Hs.433888 | chr19p13.2 |
| 34408_at | reticulon 2 | RTN2 | AF004222 | Hs.47517 | chr19q13.32 |
| 34406_at | phosphofurin acidic cluster sorting protein 2 | PACS2 | AB011174 | Hs.436281 | chr14q32.33 |
| 34260_at | TEL2, telomere maintenance 2, homolog (S. cerevisiae) | TELO2 | AB014583 | Hs.352687 | chr16p13.3 |
| 34221_at | KIAA0194 protein | KIAA0194 | D83778 | Hs.535954 | chr5q33.1 |
| 34206_at | centaurin, delta 2 | CENTD2 | AB018325 | Hs.192190 | chr11q13.4 |
| 34187_at | RNA binding motif, single stranded interacting protein 2 | RBMS2 | D28483 | Hs.438778 | chr12q13.3 |
| 33760_at | peroxisomal biogenesis factor 14 | PEX14 | AB017546 | Hs.19851 | chr1p36.22 |
| 33736_at | stomatin (EPB72)-like 1 | STOML1 | Y16522 | Hs.194816 | chr15q24-q25 |
| 33322_i_at | stratifin | SFN | X57348 | Hs.184510 | chr1p36.11 |
| 33307_at | CGI-96 protein | CTA-126B4.3 | AL022316 | Hs.534041 | chr22q13.2-q13.31 |
| 33197_at | myosin VIIA | MYO7A | U39226 | Hs.370421 | chr11q13.5 |
| 32837_at | 1-acylglycerol-3-phosphate O-acyltransferase 2 (lysophosphatidic acid acyltransferase, beta) | AGPAT2 | U56418 | Hs.209119 | chr9q34.3 |
| 32836_at | 1-acylglycerol-3-phosphate O-acyltransferase 1 (lysophosphatidic acid acyltransferase, alpha) | AGPAT1 | U56417 | Hs.301920 | chr6p21.3 |
| 32723_at | cleavage stimulation factor, 3' pre-RNA, subunit 1, 50kDa | CSTF1 | L02547 | Hs.172865 | chr20q13.31 |
| 32541_at | protein phosphatase 3 (formerly 2B), catalytic subunit, gamma isoform (calcineurin A gamma) | PPP3CC | S46622 | Hs.75206 | chr8p21.3 |
| 32259_at | enhancer of zeste homolog 1 (Drosophila) | EZH1 | AB002386 | Hs.194669 | chr17q21.1-q21.3 |
| 32209_at | family with sequence similarity 89, member B | FAM89B | AF052151 | Hs.25723 | chr11q23 |
| 32099_at | scaffold attachment factor B2 | SAFB2 | D50928 | Hs.159384 | chr19p13.3 |
| 32091_at | solute carrier family 25, member 44 | SLC25A44 | AB007915 | Hs.512854 | chr1q22 |
| 32088_at | basic leucine zipper nuclear factor 1 (JEM-1) | BLZF1 | U79751 | Hs.444697 | chr1q24 |
| 31874_at | growth arrest-specific 2 like 1 | GAS2L1 | Y07846 | Hs.322852 | chr22q12.2 |
| 31861_at | immunoglobulin mu binding protein 2 | IGHMBP2 | L14754 | Hs.33286 | chr11q13.2-q13.4 |
| 31837_at | transmembrane protein 153 | TMEM153 | U62317 | Hs.150540 | chr22q13.33 |
| 31826_at | FK506 binding protein 15, 133kDa | FKBP15 | AB014574 | Hs.14799 | chr9q32 |
| 31637_s_at | thyroid hormone receptor, alpha (erythroblastic leukemia viral (v-erb-a) oncogene homolog, avian) /// nuclear receptor subfamily 1, group D, member 1 | THRA | X72631 | Hs.724 | chr17q11.2 |
| 244881_at | leishmanolysin-like (metallopeptidase M8 family) | LMLN | AA629059 |  |  |
| 244871_s_at | ubiquitin specific peptidase 32 | USP32 | AW268357 | Hs.436133 | chr17q23.2 |
| 244842_x_at | Tubulin, gamma complex associated protein 2 | TUBGCP2 | AA235663 |  |  |
| 244841_at | SEC24 related gene family, member A (S. cerevisiae) /// family with sequence similarity 39, member D pseudogene | SEC24A | AI080364 | Hs.211612 | chr5q31.1 |
| 244832_at | Endothelin receptor type A | EDNRA | AI088707 | Hs.211202 | chr4q31.22-q31.23 |
| 244804_at | Sequestosome 1 | SQSTM1 | AW293441 |  |  |
| 244803_at | YY1 associated protein 1 | YY1AP1 | AI335191 |  |  |
| 244743_x_at | zinc finger protein 138 /// similar to Zinc finger protein 431 /// similar to Zinc finger protein 431 | ZNF138 | AA114243 | Hs.173174 | chr7q11.21-q11.23 |
| 244740_at | Hypothetical protein MGC9913 | MGC9913 | BE855713 |  |  |
| 244705_at | hypothetical protein LOC401398 | LOC401398 | AW470690 | Hs.444667 | chr7 |
| 244704_at | nuclear transcription factor Y, beta | NFYB | AW083948 | Hs.84928 | chr12q22-q23 |
| 244687_at | dihydrolipoamide branched chain transacylase E2 | DBT | AI632010 |  |  |
| 244646_at | Musashi homolog 2 (Drosophila) | MSI2 | AW972881 |  |  |
| 244631_at | hypothetical gene supported by AK123403 /// hypothetical LOC642398 /// hypothetical protein LOC727834 | LOC389834 | AI765607 | Hs.481436 |  |
| 244597_at | DNA polymerase-transactivated protein 6 | DNAPTP6 | AA701247 |  |  |
| 244569_at | chromosome 8 open reading frame 37 | C8orf37 | N92755 | Hs.128189 | chr8q22.1 |
| 244563_at | glutamine and serine rich 1 | QSER1 | BG026723 | Hs.143509 | chr11p13 |
| 244546_at | cytochrome c, somatic | CYCS | AI760495 | Hs.437060 | chr7p15.3 |
| 244523_at | Villin 2 (ezrin) | VIL2 | AW104453 | Hs.79889 | chr17q |
| 244498_x_at | similar to ubiquitin-conjugating enzyme E2Q 2 /// hypothetical LOC388160 /// hypothetical LOC388163 /// hypothetical LOC440296 /// similar to ubiquitin-conjugating enzyme E2Q 2 /// hypothetical protein LOC730145 | LOC388153 | AI921868 | Hs.513097 | chr15q25.2 |
| 244476_at | Tight junction protein 1 (zona occludens 1) | TJP1 | R39769 |  |  |
| 244462_at | Zinc finger protein 224 | ZNF224 | AA811983 |  |  |
| 244406_at | zinc finger protein 20 | ZNF20 | AI352507 | Hs.512823 /// Hs.528709 | chr19p13.2 |
| 244401_at | chromosome 6 open reading frame 152 | C6orf152 | AI922939 |  |  |
| 244396_at | GTPase activating protein (SH3 domain) binding protein 1 | G3BP1 | BE673925 | Hs.3353 | chr5q33.1 |
| 244360_at | F-box and leucine-rich repeat protein 17 | FBXL17 | AW002273 |  |  |
| 244334_at | translocation associated membrane protein 1-like 1 | TRAM1L1 | AA169554 | Hs.154218 | chr4q26 |
| 244324_at | chromosome 18 open reading frame 54 | C18orf54 | BG283921 | Hs.208701 | chr18q21.2 |
| 244287_at | splicing factor, arginine/serine-rich 12 | SFRS12 | R94399 | Hs.371166 | chr5q12.3 |
| 244268_x_at | similar to p21-activated kinase 2 | LOC646214 | BF435769 | Hs.284275 | chr3q29 |
| 244242_at | Mitochondrial ribosomal protein S28 | MRPS28 | AI652192 |  |  |
| 244197_x_at | CCR4-NOT transcription complex, subunit 2 | CNOT2 | AI859990 |  |  |
| 244189_at | KIAA1648 protein | KIAA1648 | AI888657 |  |  |
| 244165_at | chromosome 10 open reading frame 18 | C10orf18 | AI809511 |  |  |
| 244163_at | sema domain, immunoglobulin domain (Ig), short basic domain, secreted, (semaphorin) 3A | SEMA3A | BF215018 |  |  |
| 244149_at | Myosin, light chain 9, regulatory | MYL9 | AA864758 | Hs.134192 |  |
| 244103_at | chromosome 1 open reading frame 55 | C1orf55 | AW963092 | Hs.443157 | chr1q42.12 |
| 244075_at | Hydroxysteroid (17-beta) dehydrogenase 7 pseudogene 2 | HSD17B7P2 | BF224218 |  |  |
| 244050_at | protein tyrosine phosphatase-like A domain containing 2 | PTPLAD2 | AI804932 | Hs.136247 | chr9p21.3 |
| 244038_at | WD repeat domain 89 | WDR89 | AW069315 | Hs.497301 | chr14q23.2 |
| 244024_at | zinc finger protein 182 | ZNF182 | T67481 | Hs.189690 | chrxp22.11-p11.23 |
| 243982_at | BTB (POZ) domain containing 5 | BTBD5 | AA455180 |  |  |
| 243973_at | Zinc finger protein 320 | ZNF320 | R67076 |  |  |
| 243931_at | CD58 molecule | CD58 | R64696 |  |  |
| 243927_x_at | KIAA1429 | KIAA1429 | AI636247 | Hs.50273 | chr8q22.1 |
| 243899_at | ADP-ribosylation factor-like 17 pseudogene 1 | ARL17P1 | BE674920 | Hs.434982 |  |
| 243895_x_at | SMAD family member 2 | SMAD2 | AW452435 |  |  |
| 243846_x_at | Hypothetical protein FLJ32810 | FLJ32810 | H58000 |  |  |
| 243816_at | Zinc finger protein 70 | ZNF70 | N62996 | Hs.382874 | chr22q11.2 |
| 243805_at | collagen and calcium binding EGF domains 1 | CCBE1 | BG035826 | Hs.443699 | chr18q21.32 |
| 243790_at | zinc finger protein 585A | ZNF585A | AA203136 | Hs.390568 | chr19q13.12 |
| 243786_at | zinc finger, DHHC-type containing 20 | ZDHHC20 | AA454190 | Hs.24283 | chr13q12.11 |
| 243751_at | Chromodomain helicase DNA binding protein 2 | CHD2 | AA709148 |  |  |
| 243750_x_at | chromosome 21 open reading frame 70 | C21orf70 | BF109193 | Hs.410830 | chr21q22.3 |
| 243747_at | zinc finger protein 599 | ZNF599 | AI222019 |  |  |
| 243683_at | Mortality factor 4 like 2 | MORF4L2 | H43976 | Hs.411358 | chrXq22 |
| 243643_x_at | solute carrier family 30 (zinc transporter), member 6 | SLC30A6 | AI623693 | Hs.352702 | chr2p22.3 |
| 243584_at | Protease, serine, 23 | PRSS23 | AW976035 |  |  |
| 243561_at | YY1 associated factor 2 | YAF2 | AA651631 |  |  |
| 243553_x_at | Hypothetical LOC643749 | LOC643749 | R59353 |  |  |
| 243539_at | KIAA1841 | KIAA1841 | AI560205 | Hs.223206 | chr2q14 |
| 243529_at | methionine-tRNA synthetase 2 (mitochondrial) | MARS2 | BE542381 | Hs.116602 | chr2q33.1 |
| 243515_at | Chromosome 22 open reading frame 23 | C22orf23 | AI740589 |  |  |
| 243496_at | candidate tumor suppressor in ovarian cancer 2 | OVCA2 | AW367507 |  |  |
| 243462_s_at | BTB (POZ) domain containing 3 | BTBD3 | BF590303 |  |  |
| 243442_x_at | Phosphatidic acid phosphatase type 2A | PPAP2A | AI732221 |  |  |
| 243405_at | Thioredoxin domain containing 5 | TXNDC5 | AI097337 |  |  |
| 243370_at | GPI-anchored membrane protein 1 | GPIAP1 | AA642143 | Hs.278672 | chr11p13 |
| 243329_at | Polycomb group ring finger 3 | PCGF3 | AI074450 | Hs.435065 | chr4p16.3 |
| 243302_at | Branched chain keto acid dehydrogenase E1, beta polypeptide (maple syrup urine disease) | BCKDHB | AI452738 |  |  |
| 243213_at | signal transducer and activator of transcription 3 (acute-phase response factor) | STAT3 | BF508977 | Hs.421342 | chr17q21.31 |
| 243166_at | solute carrier family 30 (zinc transporter), member 5 | SLC30A5 | AA836340 |  |  |
| 243141_at | hypothetical protein MGC26963 | MGC26963 | D31421 | Hs.353519 | chr4q25 |
| 243118_at | chromosome 6 open reading frame 182 | C6orf182 | AW592456 | Hs.375746 | chr6q21 |
| 243042_at | family with sequence similarity 73, member A | FAM73A | BE645144 | Hs.107637 | chr1p31.1 |
| 243031_at | Reticulon 4 | RTN4 | N90377 |  |  |
| 243016_at | Thymidylate synthetase | TYMS | AW271958 |  |  |
| 242961_x_at | DEAD (Asp-Glu-Ala-Asp) box polypeptide 58 | DDX58 | AI304317 | Hs.438386 | chr9p12 |
| 242920_at | KIAA0999 protein | KIAA0999 | AW590838 |  |  |
| 242915_at | zinc finger protein 682 | ZNF682 | AA603590 | Hs.306298 | chr19p12 |
| 242907_at | guanylate binding protein 2, interferon-inducible | GBP2 | BF509371 | Hs.386567 | chr1p22.2 |
| 242905_at | partner of NOB1 homolog (S. cerevisiae) | PNO1 | AV733347 | Hs.262858 | chr2p14 |
| 242895_x_at | Ring finger protein 39 | RNF39 | R63837 |  |  |
| 242889_x_at | hypothetical protein LOC645431 | LOC645431 | AI820076 | Hs.357318 | chr14q24.3 |
| 242879_x_at | V-akt murine thymoma viral oncogene homolog 3 (protein kinase B, gamma) | AKT3 | AI939442 |  |  |
| 242873_at | Killer cell lectin-like receptor subfamily C, member 4 | KLRC4 | BE567130 |  |  |
| 242865_at | Neuroplastin | NPTN | AI332638 |  |  |
| 242857_at | FERM, RhoGEF and pleckstrin domain protein 2 | FARP2 | AA748613 | Hs.155206 | chr2q37.3 |
| 242851_at | KIAA1919 | KIAA1919 | BE964989 | Hs.400572 | chr6q22 |
| 242837_at | Splicing factor, arginine/serine-rich 4 | SFRS4 | AI435248 |  |  |
| 242828_at | Fidgetin | FIGN | BG109695 |  |  |
| 242794_at | mastermind-like 3 (Drosophila) | MAML3 | AI569476 |  |  |
| 242790_at | SNF8, ESCRT-II complex subunit, homolog (S. cerevisiae) | SNF8 | AA935515 |  |  |
| 242787_at | Inner centromere protein antigens 135/155kDa | INCENP | AI924134 |  |  |
| 242775_at | Leukotriene B4 12-hydroxydehydrogenase | LTB4DH | BF477980 |  |  |
| 242761_s_at | zinc finger protein 420 | ZNF420 | AI061288 | Hs.133437 | chr19q13.12 |
| 242760_x_at | phosphatidylinositol glycan anchor biosynthesis, class B | PIGB | AA808203 | Hs.126115 | chr15q21-q22 |
| 242750_at | Methylmalonic aciduria (cobalamin deficiency) cblA type | MMAA | AA678047 | Hs.117747 | chr4q31.21 |
| 242727_at | ADP-ribosylation factor-like 5B | ARL5B | BG032269 | Hs.25362 | chr10p12.31 |
| 242725_at | KDEL (Lys-Asp-Glu-Leu) endoplasmic reticulum protein retention receptor 2 | KDELR2 | BG026159 | Hs.446645 | chr7p22.1 |
| 242696_at | NudC domain containing 3 | NUDCD3 | AI252004 |  |  |
| 242688_at | Thyroid hormone receptor interactor 12 | TRIP12 | AI149880 |  |  |
| 242669_at | Ubiquitin-fold modifier 1 | UFM1 | BF514975 |  |  |
| 242665_at | formin-like 2 | FMNL2 | AL042120 | Hs.503863 | chr2q23.3 |
| 242664_at | Protein tyrosine phosphatase, receptor type, M | PTPRM | AI040744 |  |  |
| 242663_at | Hypothetical protein LOC148189 | LOC148189 | AA115127 | Hs.190090 | chr19q12 |
| 242662_at | Proprotein convertase subtilisin/kexin type 6 | PCSK6 | AI056815 |  |  |
| 242652_at | Erythrocyte membrane protein band 4.1-like 2 | EPB41L2 | AI760942 |  |  |
| 242648_at | kelch-like 8 (Drosophila) | KLHL8 | BE858995 | Hs.8346 | chr4q22.1 |
| 242639_at | NMDA receptor regulated 2 | NARG2 | AW340004 |  |  |
| 242633_x_at | Zinc finger protein 75a | ZNF75A | AA829635 | Hs.50456 | chr16p13.11 |
| 242618_at | HLA complex group 18 | HCG18 | AA430072 | Hs.301985 |  |
| 242602_x_at | zinc finger protein 254 | ZNF254 | AW051899 |  |  |
| 242592_at | G protein-coupled receptor 137C | GPR137C | AV727446 | Hs.416214 | chr14q22.1 |
| 242590_at | RNA (guanine-9-) methyltransferase domain containing 3 | RG9MTD3 | BE464643 | Hs.31293 | chr9p11 |
| 242586_at | Fibronectin type III and SPRY domain containing 1-like | FSD1L | H07986 |  |  |
| 242578_x_at | Solute carrier family 22 (extraneuronal monoamine transporter), member 3 | SLC22A3 | AV699746 |  |  |
| 242576_x_at | Phosphonoformate immuno-associated protein 5 | PFAAP5 | AW503542 |  |  |
| 242555_at | similar to RIKEN cDNA 4921524J17 | LOC388272 | AW578854 | Hs.406551 | chr16q11.2 |
| 242539_at | similar to nuclear receptor binding factor 2 /// similar to nuclear receptor binding factor 2 | LOC730069 | AW665509 |  |  |
| 242477_at | chromosome 9 open reading frame 52 | C9orf52 | BF056282 | Hs.130086 | chr9p22.3 |
| 242471_at | Hypothetical protein LOC730266 | LOC730266 | AI916641 |  |  |
| 242470_at | EP300 interacting inhibitor of differentiation 2B | EID2B | AI093963 | Hs.135181 | chr19q13.2 |
| 242463_x_at | zinc finger protein 600 | ZNF600 | AI620827 | Hs.166312 | chr19q13.41 |
| 242459_at | Chromosome 1 open reading frame 141 | C1orf141 | AI025795 |  |  |
| 242457_at | Peptidylglycine alpha-amidating monooxygenase | PAM | AW451107 |  |  |
| 242447_at | hypothetical gene supported by AK091454 | LOC285382 | AI656180 |  |  |
| 242442_x_at | RNA (guanine-9-) methyltransferase domain containing 2 | RG9MTD2 | AL515269 | Hs.380412 | chr4q23 |
| 242431_at | Ariadne homolog, ubiquitin-conjugating enzyme E2 binding protein, 1 (Drosophila) | ARIH1 | AI656728 |  |  |
| 242429_at | zinc finger protein 567 | ZNF567 | BF435183 | Hs.412517 | chr19q13.12 |
| 242427_at | WW domain containing adaptor with coiled-coil | WAC | AI982694 |  |  |
| 242425_at | V-raf-1 murine leukemia viral oncogene homolog 1 | RAF1 | BF823525 |  |  |
| 242403_at | Ubiquitin-conjugating enzyme E2D 3 (UBC4/5 homolog, yeast) | UBE2D3 | AI459177 |  |  |
| 242385_at | RAR-related orphan receptor B | RORB | R18374 | Hs.117956 | chr9q22 |
| 242384_at | Cytoplasmic polyadenylation element binding protein 4 | CPEB4 | AI452469 |  |  |
| 242377_x_at | THUMP domain containing 3 | THUMPD3 | AI051976 |  |  |
| 242371_x_at | Nuclear factor of activated T-cells 5, tonicity-responsive | NFAT5 | AW974747 |  |  |
| 242369_x_at | Nuclear receptor coactivator 2 | NCOA2 | AI561070 |  |  |
| 242365_at | Coiled-coil domain containing 32 | CCDC32 | AA921687 |  |  |
| 242362_at | Cullin 3 | CUL3 | AI797788 |  |  |
| 242349_at | HECT domain containing 1 | HECTD1 | AW275658 | Hs.283622 | chr14q12 |
| 242343_x_at | Zinc finger protein 518 | ZNF518 | H57111 |  |  |
| 242320_at | Forkhead box O3A | FOXO3A | AI435586 | Hs.423523 | chr6q21 |
| 242310_at | Formin binding protein 1-like | FNBP1L | AA665058 |  |  |
| 242293_at | inhibitor of growth family, member 3 | ING3 | AI286142 | Hs.143198 | chr7q31 |
| 242283_at | chromosome 1 open reading frame 67 | C1orf67 | AI076810 | Hs.133977 | chr1q42.12 |
| 242272_at | zinc finger protein 785 | ZNF785 | AI375066 | Hs.352367 | chr16p11.2 |
| 242255_at | WD repeat domain 37 | WDR37 | R49102 | Hs.76038 | chr10p15.3 |
| 242247_at | methyltransferase 5 domain containing 1 | METT5D1 | AL580319 | Hs.366407 | chr11p14.1 |
| 242245_at | Synapse defective 1, Rho GTPase, homolog 2 (C. elegans) | SYDE2 | N90719 | Hs.533853 | chr1p22.3 |
| 242229_at | N-acyl-phosphatidylethanolamine-hydrolyzing phospholipase D | NAPE-PLD | W01715 | Hs.431871 | chr7q22.1 |
| 242224_at | G patch domain containing 2 | GPATCH2 | R40111 | Hs.53913 | chr1q41 |
| 242214_at | ribosomal protein S27a /// similar to ubiquitin and ribosomal protein S27a precursor | RPS27A | AU152194 | Hs.311640 | chr2p16 |
| 242207_at | Inositol 1,3,4,5,6-pentakisphosphate 2-kinase | IPPK | AW292595 | Hs.494449 | chr9q21.33-q22.31 |
| 242195_x_at | numb homolog (Drosophila)-like | NUMBL | BE350811 |  |  |
| 242191_at | neuroblastoma breakpoint family, member 11 /// neuroblastoma breakpoint family, member 10 | NBPF11 | AI701905 | Hs.445556 | chr1q21.1 /// chr1 |
| 242188_at | Protein tyrosine phosphatase, receptor type, G | PTPRG | AI743332 |  |  |
| 242146_at | Small nuclear ribonucleoprotein polypeptide A' | SNRPA1 | AA872471 |  |  |
| 242139_s_at | similar to envelope protein | LOC113386 | AA635618 | Hs.435404 | chr19q13.43 |
| 242136_x_at | Hypothetical LOC403340 | MGC70870 | T66145 | Hs.459574 |  |
| 242131_at | OK/SW-cl.16 | LOC440552 | AI535835 |  | chrmitochondrion |
| 242127_at | Interleukin 28 receptor, alpha (interferon, lambda receptor) | IL28RA | W73921 |  |  |
| 242126_at | Exostoses (multiple) 1 | EXT1 | T53962 |  |  |
| 242123_at | progestin and adipoQ receptor family member VII | PAQR7 | BF764937 | Hs.356075 | chr1p36.11 |
| 242100_at | chondroitin sulfate synthase 3 | CSS3 | AI076484 | Hs.213137 | chr5q23.3 |
| 242088_at | kelch-like 24 (Drosophila) | KLHL24 | AI961401 |  |  |
| 242074_at | Phosphatidylinositol transfer protein, beta | PITPNB | AA833902 |  |  |
| 242068_at | Baculoviral IAP repeat-containing 6 (apollon) | BIRC6 | AA608834 |  |  |
| 242064_at | sidekick homolog 2 (chicken) | SDK2 | N23651 |  |  |
| 242062_at | sterile alpha motif domain containing 8 | SAMD8 | BE890410 | Hs.282105 | chr10q22.2 |
| 242060_x_at | PHD finger protein 11 | PHF11 | R08619 |  |  |
| 242048_at | prefoldin subunit 6 | PFDN6 | BE905316 | Hs.446374 | chr6p21.3 |
| 242008_at | ATP/GTP binding protein 1 | AGTPBP1 | BE350312 |  |  |
| 241992_at | Damage-regulated autophagy modulator | DRAM | AA724995 |  |  |
| 241985_at | junction-mediating and regulatory protein | JMY | AI814405 | Hs.396853 | chr5q14.1 |
| 241961_at | steroid 5 alpha-reductase 2-like 2 | SRD5A2L2 | AI822082 | Hs.227752 | chr4q13.1 |
| 241959_at | anaphase promoting complex subunit 10 /// similar to Anaphase-promoting complex subunit 10 (APC10) (Cyclosome subunit 10) /// similar to Anaphase-promoting complex subunit 10 (APC10) (Cyclosome subunit 10) | ANAPC10 | AW025455 | Hs.410760 | chr4q31 |
| 241956_at | Polycomb group ring finger 5 | PCGF5 | AI521883 |  |  |
| 241937_s_at | WD repeat domain 4 | WDR4 | AA577678 | Hs.143638 | chr21q22.3 |
| 241917_at | FCH and double SH3 domains 2 | FCHSD2 | AA875908 |  |  |
| 241887_at | Ubiquitin-conjugating enzyme E2W (putative) | UBE2W | AI370381 | Hs.128841 |  |
| 241876_at | Mdm4, transformed 3T3 cell double minute 4, p53 binding protein (mouse) | MDM4 | AW663060 |  |  |
| 241865_at | Guanine nucleotide binding protein (G protein), beta polypeptide 1 | GNB1 | AI056689 |  |  |
| 241827_at | zinc finger protein 615 | ZNF615 | AL577866 | Hs.368355 | chr19q13.41 |
| 241824_at | FOS-like antigen 2 | FOSL2 | AA019641 |  |  |
| 241808_at | Chromosome 8 open reading frame 70 | C8orf70 | AW190593 | Hs.72927 |  |
| 241786_at | Protein phosphatase 3 (formerly 2B), regulatory subunit B, 19kDa, alpha isoform (calcineurin B, type I) | PPP3R1 | AI380514 |  |  |
| 241780_at | Cadherin 11, type 2, OB-cadherin (osteoblast) | CDH11 | AI040305 |  |  |
| 241779_at | metaxin 3 | MTX3 | BE898126 | Hs.128173 | chr5q14.1 |
| 241762_at | F-box protein 32 | FBXO32 | BF244402 |  |  |
| 241757_x_at | dynein, cytoplasmic 2, light intermediate chain 1 | DYNC2LI1 | AA947051 | Hs.309257 | chr2p25.1-p24.1 |
| 241738_at | Zinc finger protein 250 | ZNF250 | AA478429 |  |  |
| 241734_at | serum response factor binding protein 1 | SRFBP1 | AI391443 | Hs.107622 | chr5q23.1 |
| 241731_x_at | zinc finger protein 440 | ZNF440 | AA883653 | Hs.44158 | chr19p13.2 |
| 241727_x_at | dihydrofolate reductase-like 1 | DHFRL1 | AA578546 | Hs.448003 | chr3q11.2 |
| 241718_x_at | KIAA0947 protein | KIAA0947 | AI148165 |  |  |
| 241710_at | similar to C1GALT1-specific chaperone 1 | LOC728819 | AI436290 |  |  |
| 241709_s_at | dedicator of cytokinesis 1 | DOCK1 | AA599017 | Hs.437620 | chr10q26.13-q26.3 |
| 241701_at | Rho GTPase activating protein 21 | ARHGAP21 | BF369489 | Hs.414164 | chr10p12.1 |
| 241699_at | Putative nucleic acid binding protein RY-1 | RY1 | R71414 |  |  |
| 241672_at | hypothetical LOC400120 | LOC400120 | BG413606 | Hs.422375 | chr13q13.3 |
| 241644_at | bolA homolog 2 (E. coli) | BOLA2 | AW770588 | Hs.449815 | chr16p12 |
| 241632_x_at | Staufen, RNA binding protein, homolog 2 (Drosophila) | STAU2 | AI335223 |  |  |
| 241627_x_at | hypothetical protein FLJ10357 | FLJ10357 | AI640434 | Hs.22451 | chr14q11.2 |
| 241613_at | Syntrophin, beta 2 (dystrophin-associated protein A1, 59kDa, basic component 2) | SNTB2 | AW296081 |  |  |
| 241611_s_at | fibronectin type III domain containing 3A | FNDC3A | BE675600 | Hs.103329 | chr13q14.2 |
| 241599_at | LSM11, U7 small nuclear RNA associated | LSM11 | AW014922 | Hs.23648 | chr5q33.3 |
| 241596_at | nudix (nucleoside diphosphate linked moiety X)-type motif 10 | NUDT10 | AL045306 | Hs.375178 | chrxp11.23 |
| 241484_x_at | Similar to FRG1 protein (FSHD region gene 1 protein) | MGC72104 | AI668696 |  |  |
| 241453_at | PTK2 protein tyrosine kinase 2 | PTK2 | AA912743 | Hs.345444 | chr8q24-qter |
| 241425_at | Nucleoporin like 1 | NUPL1 | AA769986 |  |  |
| 241392_at | Transmembrane protein 39A | TMEM39A | BE551352 |  |  |
| 241391_at | Jumonji domain containing 1C | JMJD1C | AA654772 |  |  |
| 241385_at | La ribonucleoprotein domain family, member 7 | LARP7 | AW082329 | Hs.278635 | chr4q25 |
| 241370_at | hypothetical protein LOC286052 | LOC286052 | AA278233 |  | chr8q24.13 |
| 241353_s_at | KIAA0773 gene product | KIAA0773 | AW471181 | Hs.131743 | chr7 |
| 241344_at | Galactosidase, beta 1 | GLB1 | AI821780 |  |  |
| 241303_x_at | Inositol polyphosphate-4-phosphatase, type II, 105kDa | INPP4B | AA436194 |  |  |
| 241268_x_at | SAM domain and HD domain 1 | SAMHD1 | AI939447 |  |  |
| 241218_at | Sec1 family domain containing 2 | SCFD2 | AI927309 | Hs.201679 |  |
| 241198_s_at | chromosome 11 open reading frame 70 | C11orf70 | BE645435 | Hs.98328 | chr11q22.2 |
| 241065_x_at | Cytidine monophosphate N-acetylneuraminic acid synthetase | CMAS | AI076273 | Hs.311346 | chr12p12.1 |
| 240971_x_at | Cullin 4A | CUL4A | AI950451 |  |  |
| 240721_at | KIAA1967 | KIAA1967 | BE672858 |  |  |
| 240557_at | TSC22 domain family, member 2 | TSC22D2 | N34514 | Hs.445692 |  |
| 240542_at | Microtubule-associated protein 4 | MAP4 | AA039509 |  |  |
| 240539_at | Autism susceptibility candidate 2 | AUTS2 | AI684551 |  |  |
| 240486_at | Hypothetical protein LOC284019 | LOC284019 | AA761673 |  |  |
| 240456_at | Ankyrin repeat domain 55 | ANKRD55 | AA459699 |  |  |
| 240436_at | Similar to FRAS1-related extracellular matrix protein 2 precursor (ECM3 homolog) | LOC650794 | AA688101 | Hs.534793 | chr13q12.11 |
| 240429_at | zinc finger protein 546 | ZNF546 | AA130174 | Hs.103388 | chr19q13.2 |
| 240421_x_at | similar to WW45 protein | LOC646561 | AI693524 |  |  |
| 240385_at | GATA binding protein 6 | GATA6 | BF002339 |  |  |
| 240382_at | Desmoplakin | DSP | AW444944 | Hs.15550 |  |
| 240363_at | ankyrin 1, erythrocytic | ANK1 | AW044553 | Hs.443711 | chr8p11.1 |
| 240312_at | similar to CG4768-PA | LOC389895 | AA608813 | Hs.127679 | chrXq27.1 |
| 240261_at | target of myb1-like 1 (chicken) | TOM1L1 | AA913079 | Hs.436988 | chr17q22 |
| 240239_at | zinc finger protein 566 | ZNF566 | N63953 | Hs.131582 | chr19q13.12 |
| 240237_at | Chloride channel 3 | CLCN3 | H23230 |  |  |
| 240231_at | Antizyme inhibitor 1 | AZIN1 | AI742383 |  |  |
| 240205_x_at | KIAA0528 | KIAA0528 | AW188170 |  |  |
| 240172_at | ERGIC and golgi 2 | ERGIC2 | AA102332 |  |  |
| 240139_at | Ubiquitin-conjugating enzyme E2D 2 (UBC4/5 homolog, yeast) | UBE2D2 | AI949690 |  |  |
| 240125_at | Dystrobrevin, alpha | DTNA | AI697494 |  |  |
| 240121_x_at | Similar to Tricarboxylate transport protein, mitochondrial precursor (Citrate transport protein) (CTP) (Tricarboxylate carrier protein) (Solute carrier family 25 member 1) | LOC642290 | AI042344 |  |  |
| 240108_at | Zinc finger, UBR1 type 1 | ZUBR1 | BE349858 |  |  |
| 240106_at | N-acetylglucosamine-1-phosphate transferase, alpha and beta subunits | GNPTAB | AI633523 | Hs.412128 | chr12q23.3 |
| 240038_at | Elongation factor, RNA polymerase II, 2 | ELL2 | AW057518 |  |  |
| 240024_at | SEC14-like 2 (S. cerevisiae) | SEC14L2 | AI571796 |  |  |
| 239999_at | Chromosome 21 open reading frame 34 | C21orf34 | N66393 |  |  |
| 239960_x_at | Lyrm7 homolog (mouse) | LYRM7 | AW303539 |  |  |
| 239930_at | UDP-N-acetyl-alpha-D-galactosamine:polypeptide N-acetylgalactosaminyltransferase 2 (GalNAc-T2) | GALNT2 | AA604126 | Hs.443307 | chr1q41-q42 |
| 239896_at | RAB guanine nucleotide exchange factor (GEF) 1 pseudogene | LOC493754 | AW190479 |  |  |
| 239872_at | Oxysterol binding protein 2 | OSBP2 | AW302848 |  |  |
| 239834_at | Potassium channel tetramerisation domain containing 1 | KCTD1 | AW874669 |  |  |
| 239833_at | Copper metabolism (Murr1) domain containing 1 | COMMD1 | H15900 |  |  |
| 239824_s_at | transmembrane protein 107 | TMEM107 | BF971873 | Hs.413219 | chr17p13.1 |
| 239809_at | Kruppel-like factor 7 (ubiquitous) | KLF7 | BF197708 |  |  |
| 239798_at | Pyruvate dehydrogenase kinase, isozyme 1 | PDK1 | AI825068 |  |  |
| 239796_x_at | toll-interleukin 1 receptor (TIR) domain containing adaptor protein | TIRAP | AA284071 |  |  |
| 239794_at | Isoleucine-tRNA synthetase | IARS | AI356405 |  |  |
| 239785_at | DAZ interacting protein 1-like | DZIP1L | BF340245 | Hs.351403 | chr3q22.3 |
| 239784_at | SET and MYND domain containing 3 | SMYD3 | AA398740 |  |  |
| 239780_at | Kinesin family member 1B | KIF1B | AA468422 |  |  |
| 239777_at | hypothetical protein LOC283551 | LOC283551 | BF691523 | Hs.292580 | chr14q21.3 |
| 239757_at | Zinc finger, AN1-type domain 6 | ZFAND6 | AA741493 |  |  |
| 239752_at | Cat eye syndrome chromosome region, candidate 2 | CECR2 | BE551781 | Hs.231895 |  |
| 239748_x_at | OCIA domain containing 1 | OCIAD1 | H09533 |  |  |
| 239735_at | Region containing SMA4; hypothetical protein LOC153561 | LOC643367 | N67106 |  |  |
| 239697_x_at | FLJ42117 protein | FLJ42117 | AA521262 | Hs.368434 | chr3p14.2 |
| 239675_at | hypothetical protein LOC283143 | LOC283143 | BE348537 | Hs.130499 | chr11q23.3 |
| 239648_at | DCN1, defective in cullin neddylation 1, domain containing 3 (S. cerevisiae) | DCUN1D3 | AI765327 | Hs.101007 | chr16p12.3 |
| 239603_x_at | F-box protein 11 | FBXO11 | AI082479 |  |  |
| 239596_at | solute carrier family 30 (zinc transporter), member 7 | SLC30A7 | AA521381 | Hs.38856 | chr1p21.2 |
| 239538_at | zinc finger, RAN-binding domain containing 3 | ZRANB3 | BG548811 | Hs.307082 | chr2q21.3 |
| 239536_at | Chromosome 1 open reading frame 56 | C1orf56 | AW969265 |  |  |
| 239516_at | Lysophospholipase-like 1 | LYPLAL1 | AI806583 |  |  |
| 239482_x_at | Zinc finger protein 708 | ZNF708 | BF223715 | Hs.105151 | chr19p12 |
| 239481_at | hypothetical protein FLJ37659 | RP1-32F7.2 | AI864183 | Hs.110069 | chrxq21.33 |
| 239468_at | mohawk homeobox | MKX | AW023227 | Hs.128193 | chr10p12.1 |
| 239466_at | hypothetical LOC344595 | LOC344595 | AA463827 | Hs.450057 | chr3q13.12 |
| 239462_at | zinc finger protein 284 | ZNF284 | AI559740 | Hs.445395 | chr19q13.31 |
| 239450_at | NADH dehydrogenase (ubiquinone) flavoprotein 2, 24kDa | NDUFV2 | AA846867 |  |  |
| 239448_at | SMAD family member 3 | SMAD3 | AI475033 |  |  |
| 239435_x_at | Shroom family member 1 | SHROOM1 | AA588854 | Hs.352292 | chr5q23.3 |
| 239432_at | hypothetical protein FLJ31306 | FLJ31306 | AV729086 |  |  |
| 239415_at | microtubule-associated protein 9 | MAP9 | AW117322 |  |  |
| 239409_at | RAP1A, member of RAS oncogene family | RAP1A | AA828280 |  |  |
| 239391_at | Family with sequence similarity 120A opposite strand | FAM120AOS | AA195528 | Hs.350364 | chr9q22.31 |
| 239390_at | glycosyltransferase-like domain containing 1 | GTDC1 | BE327650 | Hs.159460 | chr2q22.3 |
| 239376_at | DNA cross-link repair 1C (PSO2 homolog, S. cerevisiae) | DCLRE1C | AA489041 | Hs.536300 |  |
| 239367_at | brain-derived neurotrophic factor | BDNF | AI127530 | Hs.439027 | chr11p13 |
| 239346_at | Chromosome 12 open reading frame 38 | C12orf38 | AI097560 |  |  |
| 239343_at | hypothetical protein LOC728705 | LOC728705 | AW451176 | Hs.129828 |  |
| 239331_at | Hypothetical protein FLJ43663 | FLJ43663 | AW954199 |  |  |
| 239300_at | Phosphoinositide-3-kinase, class 3 | PIK3C3 | AI632214 | Hs.239340 |  |
| 239296_at | Zinc finger homeobox 1b | ZFHX1B | AI623184 |  |  |
| 239288_at | TRAF2 and NCK interacting kinase | TNIK | BF431017 | Hs.20755 | chr3q26.2 |
| 239283_at | transmembrane emp24 protein transport domain containing 5 | TMED5 | AA259174 | Hs.348996 | chr1pter-q31.3 |
| 239243_at | Zinc finger protein 638 | ZNF638 | AA279654 |  |  |
| 239233_at | KIAA1212 | KIAA1212 | AA744613 | Hs.292925 | chr2p16.3 |
| 239228_at | Casein kinase 2, alpha 1 polypeptide | CSNK2A1 | AI298887 |  |  |
| 239223_s_at | F-box and leucine-rich repeat protein 20 | FBXL20 | AA649201 | Hs.194498 | chr17q12 |
| 239207_at | jumonji, AT rich interactive domain 1C | JARID1C | BE503653 | Hs.103381 | chrxp11.22-p11.21 |
| 239196_at | ankyrin repeat domain 22 | ANKRD22 | AI097229 | Hs.217484 | chr10q23.31 |
| 239171_at | Adducin 3 (gamma) | ADD3 | AA766886 |  |  |
| 239161_at | ferredoxin 1 | FDX1 | BE302085 | Hs.744 | chr11q22 |
| 239143_x_at | ring finger protein 138 | RNF138 | AW291944 | Hs.180403 | chr18q12.1 |
| 239142_at | Rieske (Fe-S) domain containing | RFESD | AI082852 | Hs.399758 | chr5q15 |
| 239133_at | CTD (carboxy-terminal domain, RNA polymerase II, polypeptide A) small phosphatase like 2 | CTDSPL2 | AW169159 |  |  |
| 239106_at | Hypothetical protein LOC728999 | LOC728999 | AI801563 | Hs.31535 | chrXp22.2 |
| 239094_at | hypothetical protein LOC730961 | LOC730961 | AA479155 | Hs.91773 | chr5q23-q31 |
| 239090_at | Nuclear receptor subfamily 1, group D, member 2 | NR1D2 | BF110321 | Hs.440466 | chr2p15 |
| 239083_at | zinc finger protein 786 | ZNF786 | AI560246 | Hs.201648 | chr7q36.1 |
| 239054_at | Scm-like with four mbt domains 1 | SFMBT1 | BE348277 |  |  |
| 239046_at | TATA box binding protein (TBP)-associated factor, RNA polymerase I, B, 63kDa | TAF1B | AA322245 |  |  |
| 239043_at | zinc finger protein 404 | ZNF404 | AA084273 | Hs.76561 | chr19q13.31 |
| 239042_at | TSR1, 20S rRNA accumulation, homolog (S. cerevisiae) | TSR1 | AI282511 | Hs.388170 | chr17p13.3 |
| 239041_at | Histone cluster 1, H2ak | HIST1H2AK | AV747226 |  |  |
| 239035_at | 5,10-methylenetetrahydrofolate reductase (NADPH) | MTHFR | AL138431 | Hs.152547 | chr1p36.3 |
| 239034_at | chromosome X open reading frame 24 | CXorf24 | BE548277 |  | chrxp11.3 |
| 239010_at | Hypothetical gene supported by AK096951; BC066547 | LOC440157 | AI744280 | Hs.517325 | chr14q11.1 |
| 239007_at | zinc finger protein 616 | ZNF616 | BG170845 | Hs.253272 | chr19q13.41 |
| 238992_at | Polymerase (DNA directed) iota | POLI | AW129145 |  |  |
| 238990_x_at | similar to ring finger protein 129 /// similar to ring finger protein 129 | LOC653794 | AI763262 | Hs.130059 | chr4q32.3 |
| 238965_at | Chromosome 21 open reading frame 2 | C21orf2 | AW300217 | Hs.155361 | chr21q22.3 |
| 238953_at | Sp2 transcription factor | SP2 | AA993833 | Hs.318639 |  |
| 238952_x_at | hypothetical protein DKFZp779O175 | DKFZp779O175 | BF439163 | Hs.124047 | chr19q13.12 |
| 238949_at | hypothetical gene supported by NM_144726 | LOC401805 | BF681162 |  |  |
| 238948_at | Transmembrane 9 superfamily member 1 | TM9SF1 | AA448858 |  |  |
| 238946_at | Chromosome 11 open reading frame 54 | C11orf54 | AW469495 |  |  |
| 238935_at | ribosomal protein S27-like | RPS27L | AW958475 |  |  |
| 238931_at | Methyltransferase 10 domain containing | METT10D | AL045793 | Hs.224187 | chr17p13.3 |
| 238929_at | splicing factor, arginine/serine-rich 2B | SFRS2B | N30132 |  |  |
| 238923_at | speckle-type POZ protein | SPOP | AW449169 |  |  |
| 238917_s_at | hypothetical protein MGC24039 | MGC24039 | AI732405 | Hs.436082 | chr12p11.21 |
| 238913_at | Cleavage and polyadenylation specific factor 6, 68kDa | CPSF6 | AW235215 |  |  |
| 238912_x_at | chromosome 9 open reading frame 85 | C9orf85 | AA824363 | Hs.494155 | chr9q21.13 |
| 238909_at | S100 calcium binding protein A10 | S100A10 | BF126155 |  |  |
| 238893_at | hypothetical protein LOC338758 | LOC338758 | AI377324 | Hs.136888 | chr12q21.33 |
| 238883_at | Thyroid hormone receptor associated protein 2 | THRAP2 | AW975051 |  |  |
| 238869_at | Hypoxia-inducible factor 1, alpha subunit (basic helix-loop-helix transcription factor) | HIF1A | AA913703 |  |  |
| 238863_x_at | Component of oligomeric golgi complex 8 | COG8 | AI524240 |  |  |
| 238856_s_at | Pantothenate kinase 2 (Hallervorden-Spatz syndrome) | PANK2 | BG108346 | Hs.203589 | chr20p13 |
| 238848_at | OTU domain containing 4 | OTUD4 | BF750565 | Hs.252722 | chr4q31.21 |
| 238844_s_at | nephronophthisis 1 (juvenile) | NPHP1 | BF216535 | Hs.280388 | chr2q13 |
| 238833_at | hypothetical protein LOC729088 | LOC729088 | AW139053 |  |  |
| 238829_at | KIAA1840 | KIAA1840 | AI540253 |  |  |
| 238825_at | acidic repeat containing | ACRC | AI091533 | Hs.135167 | chrxq13.1 |
| 238823_at | formin-like 3 | FMNL3 | AA481044 | Hs.179838 | chr12q13.12 |
| 238821_at | Cleavage stimulation factor, 3' pre-RNA, subunit 2, 64kDa | CSTF2 | AI826277 |  |  |
| 238800_s_at | hypothetical protein MGC13114 | MGC13114 | AA776496 | Hs.12742 | chr9q21 |
| 238794_at | chromosome 10 open reading frame 78 | C10orf78 | N23586 | Hs.93667 | chr10q25.1 |
| 238793_at | tigger transposable element derived 7 | TIGD7 | AI925903 | Hs.458452 | chr16p13.3 |
| 238785_at | Chromosome 3 open reading frame 63 | C3orf63 | AI632091 |  |  |
| 238775_at | Integrin alpha FG-GAP repeat containing 1 | ITFG1 | AA088543 |  |  |
| 238774_at | KIAA1267 | KIAA1267 | AW960454 |  |  |
| 238765_at | ATPase, H+ transporting, lysosomal 13kDa, V1 subunit G1 | ATP6V1G1 | AA732007 | Hs.90336 | chr9q32 |
| 238756_at | Growth arrest-specific 2 like 3 /// Myotrophin | GAS2L3 | AI860012 | Hs.20575 | chr12q23.1 |
| 238727_at | Hypothetical gene supported by BC008048 | LOC440934 | AA031832 | Hs.238964 | chr2q36.1 |
| 238726_at | Ubiquitin specific peptidase 46 | USP46 | AW576443 |  |  |
| 238724_at | 2,3-bisphosphoglycerate mutase | BPGM | R63824 |  |  |
| 238711_s_at | Solute carrier family 12 (potassium/chloride transporters), member 8 | SLC12A8 | AI669304 |  |  |
| 238706_at | PAP associated domain containing 4 | PAPD4 | BG168850 | Hs.220277 | chr5q14.1 |
| 238701_x_at | FLJ45803 protein | FLJ45803 | BE176566 | Hs.125166 | chr11q23.1 |
| 238700_at | Protein inhibitor of activated STAT, 2 | PIAS2 | AI825302 |  |  |
| 238698_at | calcium/calmodulin-dependent serine protein kinase (MAGUK family) | CASK | AI659225 | Hs.460584 | chrxp11.4 |
| 238695_s_at | RAB39B, member RAS oncogene family | RAB39B | AW960289 | Hs.24970 | chrxq28 |
| 238686_at | F-box protein 3 | FBXO3 | AA130258 | Hs.406787 | chr11p13 |
| 238672_at | Peroxisomal biogenesis factor 6 | PEX6 | AW953952 |  |  |
| 238649_at | phosphatidylinositol transfer protein, cytoplasmic 1 | PITPNC1 | AA815089 | Hs.112703 | chr17q24.2 |
| 238647_at | chromosome 14 open reading frame 28 /// synaptophysin-like 2 | C14orf28 | AA496213 | Hs.82098 | chr14q21.2 |
| 238633_at | Enhancer of polycomb homolog 1 (Drosophila) | EPC1 | W93523 |  |  |
| 238615_at | SPFH domain family, member 2 | SPFH2 | AI817403 | Hs.125849 | chr8p11.2 |
| 238614_x_at | zinc finger protein 430 | ZNF430 | AW954842 |  |  |
| 238609_at | chromosome 7 open reading frame 38 | C7orf38 | T53175 | Hs.213586 | chr7q22.1 |
| 238606_at | zinc finger protein 747 | ZNF747 | AV698217 | Hs.460604 | chr16p11.2 |
| 238602_at | hypothetical protein MGC42174 | MGC42174 | AL036541 | Hs.471637 | chr2q37.1 |
| 238598_s_at | Ring finger protein 32 | RNF32 | BE962709 | Hs.146037 |  |
| 238597_at | Ankyrin repeat domain 13C | ANKRD13C | BG028770 |  |  |
| 238593_at | hypothetical protein FLJ22531 | FLJ22531 | AW962511 | Hs.292088 | chr11q13.2 |
| 238587_at | Cbl-interacting protein Sts-1 | STS-1 | AI927919 | Hs.55067 | chr11q24.1 |
| 238568_s_at | Chromosome 18 open reading frame 8 | C18orf8 | BE742377 |  |  |
| 238562_at | Chromosome 8 open reading frame 53 | C8orf53 | BE542779 | Hs.86970 | chr8q24.11 |
| 238551_at | fucosyltransferase 11 (alpha (1,3) fucosyltransferase) | FUT11 | BF541967 | Hs.500082 | chr10q22.2 |
| 238542_at | UL16 binding protein 2 | ULBP2 | AA831769 | Hs.250618 | chr6q25 |
| 238539_at | Hermansky-Pudlak syndrome 3 | HPS3 | BG163294 | Hs.282804 | chr3q24 |
| 238534_at | Leucine rich repeat (in FLII) interacting protein 1 | LRRFIP1 | AA262583 |  |  |
| 238516_at | bone morphogenetic protein receptor, type II (serine/threonine kinase) | BMPR2 | BF247383 | Hs.53250 | chr2q33-q34 |
| 238513_at | Proline rich Gla (G-carboxyglutamic acid) 4 (transmembrane) | PRRG4 | BF905445 | Hs.146937 | chr11p13 |
| 238510_at | zinc finger protein 720 | ZNF720 | AA744964 | Hs.412961 | chr16p11.2 |
| 238505_at | ADP-ribosylarginine hydrolase | ADPRH | BF510789 | Hs.99884 | chr3q13.31-q13.33 |
| 238504_at | chromosome 6 open reading frame 57 | C6orf57 | AA521023 |  |  |
| 238497_at | transmembrane protein 136 | TMEM136 | N51836 | Hs.380228 | chr11q23.3 |
| 238496_at | Wolf-Hirschhorn syndrome candidate 1-like 1 | WHSC1L1 | AA741074 |  |  |
| 238495_at | Synaptosomal-associated protein, 23kDa | SNAP23 | N22360 | Hs.43153 |  |
| 238494_at | TNF receptor-associated factor 3 interacting protein 1 | TRAF3IP1 | AI623155 | Hs.43086 | chr2q37.3 |
| 238490_at | KIAA2026 | KIAA2026 | BG109896 | Hs.446387 | chr9p24.1 |
| 238472_at | F-box protein 9 | FBXO9 | R73399 |  |  |
| 238465_at | chromosome 5 open reading frame 35 | C5orf35 | AI265933 | Hs.85950 | chr5q11.2 |
| 238458_at | EF-hand domain family, member A2 | EFHA2 | AI868167 | Hs.368054 | chr8p22 |
| 238449_at | PI-3-kinase-related kinase SMG-1 pseudogene | LOC595101 | BG534511 | Hs.460278 | chr16p12.1 |
| 238444_at | zinc finger protein 618 | ZNF618 | AV704303 | Hs.142372 | chr9q32 |
| 238430_x_at | schlafen family member 5 | SLFN5 | AI923675 | Hs.14691 | chr17q12 |
| 238417_at | phosphoglucomutase 2-like 1 | PGM2L1 | BE138757 | Hs.26612 | chr11q13.4 |
| 238402_s_at | hypothetical protein FLJ35220 | FLJ35220 | AI311040 | Hs.389678 | chr17q25.3 |
| 238332_at | ankyrin repeat domain 29 | ANKRD29 | AI307802 | Hs.374774 | chr18q11.2 |
| 238299_at | LAG1 homolog, ceramide synthase 5 (S. cerevisiae) | LASS5 | AW005866 |  |  |
| 238295_at | Chromosome 17 open reading frame 42 | C17orf42 | N22706 |  |  |
| 238205_at | WD repeat domain 40B | WDR40B | AW572906 | Hs.120403 | chrxq25 |
| 238156_at | Ribosomal protein S6 | RPS6 | AW205632 |  |  |
| 238151_at | Tubulin, beta 6 | TUBB6 | BF511636 |  |  |
| 238148_s_at | FLJ46385 protein | FLJ46385 | AI651641 | Hs.512510 | chr19q13.42 |
| 238124_at | Myomesin family, member 3 | MYOM3 | AI124053 | Hs.174497 | chr1p36.11 |
| 238122_at | RNA binding motif protein 12B | RBM12B | AI807494 | Hs.33540 | chr8q22.1 |
| 238121_at | glycerol kinase 5 (putative) | GK5 | AI473796 | Hs.135904 |  |
| 238114_at | Protein-L-isoaspartate (D-aspartate) O-methyltransferase domain containing 1 | PCMTD1 | AI962276 |  |  |
| 238078_at | SEC22 vesicle trafficking protein homolog A (S. cerevisiae) | SEC22A | N71074 |  |  |
| 238071_at | lipocalin 10 | LCN10 | AI823802 | Hs.98132 | chr9q34.3 |
| 238067_at | TBC1 domain family, member 8B (with GRAM domain) | TBC1D8B | AW172431 | Hs.351798 | chrxq22.3 |
| 238057_at | ubiquitin specific peptidase 45 | USP45 | AW195800 | Hs.213248 | chr6q16.3 |
| 238056_at | succinate dehydrogenase complex, subunit C, integral membrane protein, 15kDa | SDHC | AW183074 |  |  |
| 238045_at | transmembrane protein 65 | TMEM65 | BG167675 | Hs.187646 | chr8q24.13 |
| 238043_at | similar to AT rich interactive domain 1B (SWI1-like) isoform 1 | LOC729446 | AI913123 |  |  |
| 238041_at | Transcription factor 12 (HTF4, helix-loop-helix transcription factors 4) | TCF12 | AA151712 |  |  |
| 238035_at | Sp3 transcription factor | SP3 | N66313 | Hs.154295 | chr2q31 |
| 238034_at | calnexin | CANX | AI890021 |  |  |
| 238030_at | Zinc finger protein 268 | ZNF268 | AI277336 |  |  |
| 238026_at | Ribosomal protein L35a | RPL35A | AI458020 |  |  |
| 238021_s_at | hypothetical LOC643911 /// hypothetical protein LOC650242 | LOC643911 | AA954994 | Hs.237396 | chr16q12.2 |
| 238020_at | Proteasome (prosome, macropain) 26S subunit, ATPase, 2 | PSMC2 | BG166796 |  |  |
| 238018_at | hypothetical protein LOC285016 | LOC285016 | BF449053 |  |  |
| 238012_at | Dipeptidyl-peptidase 7 | DPP7 | AI620209 | Hs.530313 | chr9q34.3 |
| 238011_at | Chromosome 16 open reading frame 72 | C16orf72 | BF668314 | Hs.406602 | chr16p13.2 |
| 238010_at | chromosome 1 open reading frame 174 | C1orf174 | BF970340 | Hs.103939 | chr1p36.32 |
| 238007_at | Zinc finger protein 271 | ZNF271 | AA464946 |  |  |
| 238001_at | potassium channel tetramerisation domain containing 6 | KCTD6 | BF693998 | Hs.13982 | chr3p14.3 |
| 237939_at | EPH receptor A5 | EPHA5 | BE218107 |  |  |
| 237817_at | Signal sequence receptor, gamma (translocon-associated protein gamma) | SSR3 | AI939474 |  |  |
| 237702_at | Developmentally regulated RNA-binding protein 1 | DRB1 | AW451271 | Hs.377257 | chr2q31.2 |
| 237690_at | G protein-coupled receptor 115 | GPR115 | W67511 | Hs.150131 | chr6p12.3 |
| 237623_at | Cystatin C (amyloid angiopathy and cerebral hemorrhage) | CST3 | AI829605 | Hs.304682 | chr20p11.21 |
| 237515_at | Transmembrane protein 56 | TMEM56 | AA054642 | Hs.84522 | chr1p21.3 |
| 237465_at | Ubiquitin specific peptidase 53 | USP53 | BF196943 | Hs.534941 | chr4q26 |
| 237464_at | SLC7A5 pseudogene | IMAA | AI241501 |  |  |
| 237426_at | SP100 nuclear antigen | SP100 | AA286940 | Hs.371696 | chr2q37.1 |
| 237411_at | ADAM metallopeptidase with thrombospondin type 1 motif, 6 | ADAMTS6 | N71063 | Hs.382857 | chr5q12.3 |
| 237400_at | ATP synthase, H+ transporting, mitochondrial F0 complex, subunit s (factor B) | ATP5S | AI308101 |  |  |
| 237333_at | Syncoilin, intermediate filament 1 | SYNC1 | T90771 |  |  |
| 237305_at | Cadherin 2, type 1, N-cadherin (neuronal) | CDH2 | AW450381 |  |  |
| 237116_at | hypothetical LOC646903 | LOC646903 | AI262277 | Hs.91747 | chr3q25.1-q25.2 |
| 237040_at | CWF19-like 2, cell cycle control (S. pombe) | CWF19L2 | BF185264 | Hs.24380 | chr11q22.3 |
| 237030_at | acid phosphatase, prostate | ACPP | AI659898 | Hs.388677 | chr3q21-q23 |
| 236978_at | Thyroid hormone receptor associated protein 1 | THRAP1 | N51961 |  |  |
| 236961_at | Atrophin 1 | ATN1 | AI539426 |  |  |
| 236946_at | G protein-coupled receptor 75 | GPR75 | AI220134 |  |  |
| 236908_at | Acid phosphatase-like 2 | ACPL2 | BE550429 |  |  |
| 236851_x_at | Golgi phosphoprotein 3 (coat-protein) | GOLPH3 | AI693378 |  |  |
| 236841_at | Family with sequence similarity 39, member D pseudogene | FAM39DP | BE464132 |  |  |
| 236836_at | Tubulin folding cofactor A | TBCA | BE503070 | Hs.433254 | chr5q14.1 |
| 236816_at | chromosome 12 open reading frame 30 | C12orf30 | BF110370 | Hs.314063 | chr12q24.13 |
| 236808_at | FGFR1 oncogene partner 2 | FGFR1OP2 | AA947475 | Hs.406184 | chr12p11.23 |
| 236796_at | BTB and CNC homology 1, basic leucine zipper transcription factor 2 | BACH2 | AI052447 |  |  |
| 236782_at | sterile alpha motif domain containing 3 | SAMD3 | AI129628 | Hs.440508 | chr6q23.1 |
| 236769_at | hypothetical protein LOC158402 | LOC158402 | BE504242 | Hs.153799 | chr9q32 |
| 236671_at | Catechol-O-methyltransferase | COMT | AI829569 |  |  |
| 236649_at | DTW domain containing 1 | DTWD1 | AA907927 | Hs.127432 | chr15q21.2 |
| 236622_at | phosphatidylinositol glycan anchor biosynthesis, class M | PIGM | AI761712 |  |  |
| 236621_at | ribosomal protein S27 (metallopanstimulin 1) | RPS27 | AV683684 | Hs.337307 | chr1q21 |
| 236606_at | Salvador homolog 1 (Drosophila) | SAV1 | N50912 |  |  |
| 236600_at | spastic paraplegia 20, spartin (Troyer syndrome) | SPG20 | AI651603 | Hs.205088 | chr13q13.3 |
| 236594_at | lethal giant larvae homolog 1 (Drosophila) | LLGL1 | AI570526 | Hs.513983 | chr17p11.2 |
| 236586_at | Chloride channel, nucleotide-sensitive, 1A | CLNS1A | R37396 |  |  |
| 236574_at | Hypothetical protein LOC284373 | LOC284373 | AI304870 |  |  |
| 236557_at | zinc finger and BTB domain containing 38 | ZBTB38 | AW085625 | Hs.23853 | chr3q23 |
| 236551_at | zinc finger protein 311 | ZNF311 | BF508689 | Hs.148218 | chr6p22.1 |
| 236545_at | Protein phosphatase 3 (formerly 2B), catalytic subunit, alpha isoform (calcineurin A alpha) | PPP3CA | AA532718 |  |  |
| 236535_at | structural maintenance of chromosomes 6 | SMC6 | AW069285 | Hs.424559 | chr2p24.2 |
| 236526_x_at | hypothetical protein LOC643079 | LOC643079 | BE222610 | Hs.184664 | chr5q12.3 |
| 236520_at | FK506 binding protein 1A, 12kDa | FKBP1A | AW972380 |  |  |
| 236515_at | SAPS domain family, member 3 | SAPS3 | AI055865 |  |  |
| 236514_at | Acyl-CoA thioesterase 8 | ACOT8 | AI885067 |  |  |
| 236513_at | Hypothetical protein MGC21644 | MGC21644 | AW770245 |  |  |
| 236487_at | Sodium channel and clathrin linker 1 | SCLT1 | AW513286 | Hs.404000 | chr4q28.2 |
| 236477_at | PRP40 pre-mRNA processing factor 40 homolog A (yeast) | PRPF40A | AA176247 |  |  |
| 236476_at | Mature T-cell proliferation 1 | MTCP1 | AW779983 | Hs.3548 | chrXq28 |
| 236472_at | Oxysterol binding protein-like 9 | OSBPL9 | AI806586 |  |  |
| 236471_at | nuclear factor (erythroid-derived 2)-like 3 | NFE2L3 | AI949827 | Hs.404741 | chr7p15-p14 |
| 236462_at | YEATS domain containing 2 | YEATS2 | AA742310 |  |  |
| 236453_at | hypothetical LOC441440 | LOC441440 | AW243154 | Hs.99081 | chr9p11.2 |
| 236449_at | Cystatin B (stefin B) | CSTB | AI885390 |  |  |
| 236429_at | Zinc finger protein 83 | ZNF83 | AI831874 |  |  |
| 236384_at | Integrin, alpha E (antigen CD103, human mucosal lymphocyte antigen 1; alpha polypeptide) | ITGAE | BE675101 | Hs.41877 |  |
| 236378_at | calcium and integrin binding family member 4 | CIB4 | BF681360 | Hs.269961 | chr2p23.3 |
| 236356_at | NADH dehydrogenase (ubiquinone) Fe-S protein 1, 75kDa (NADH-coenzyme Q reductase) | NDUFS1 | AI808395 | Hs.420563 | chr2q33-q34 |
| 236338_at | Insulin receptor substrate 2 | IRS2 | AI766299 |  |  |
| 236328_at | zinc finger protein 285 | ZNF285 | AW513227 | Hs.466896 | chr19q13.32 |
| 236313_at | cyclin-dependent kinase inhibitor 2B (p15, inhibits CDK4) | CDKN2B | AW444761 | Hs.72901 | chr9p21 |
| 236302_at | protein phosphatase 1E (PP2C domain containing) | PPM1E | R40892 | Hs.166351 | chr17q23.2 |
| 236281_x_at | 5-hydroxytryptamine (serotonin) receptor 7 (adenylate cyclase-coupled) | HTR7 | R44298 | Hs.73739 | chr10q21-q24 |
| 236278_at | Protein kinase, cAMP-dependent, catalytic, beta | PRKACB | AV705309 |  |  |
| 236277_at | P21 (CDKN1A)-activated kinase 3 | PAK3 | H23551 | Hs.134802 | chrXq22.3-q23 |
| 236268_at | SEC22 vesicle trafficking protein homolog C (S. cerevisiae) | SEC22C | BF732413 |  |  |
| 236265_at | Sp4 transcription factor | SP4 | BF438799 | Hs.88013 |  |
| 236254_at | vacuolar protein sorting 13 homolog B (yeast) | VPS13B | BE048857 | Hs.367994 | chr8q22.2 |
| 236247_at | NOL1/NOP2/Sun domain family, member 4 | NSUN4 | AI536692 | Hs.439794 | chr1p34 |
| 236243_at | Zinc finger, CCHC domain containing 6 | ZCCHC6 | AW070776 | Hs.12742 | chr9q21 |
| 236241_at | Mediator of RNA polymerase II transcription, subunit 31 homolog (S. cerevisiae) | MED31 | BF593977 | Hs.43566 | chr17p13.1 |
| 236240_at | chromosome 4 open reading frame 29 | C4orf29 | BE788256 | Hs.407155 | chr4q28.2 |
| 236229_at | Similar to Zinc finger protein 418 | LOC730051 | AW014345 |  |  |
| 236223_s_at | Ras-like without CAAX 1 | RIT1 | AL037450 |  |  |
| 236204_at | COP9 constitutive photomorphogenic homolog subunit 8 (Arabidopsis) | COPS8 | AI193769 |  |  |
| 236193_at | histone cluster 1, H2bc | HIST1H2BC | AA037483 |  |  |
| 236192_at | Farnesyltransferase, CAAX box, alpha | FNTA | BF447112 |  |  |
| 236180_at | Solute carrier family 2 (facilitated glucose transporter), member 3 | SLC2A3 | W57613 |  |  |
| 236167_at | Tensin 3 | TNS3 | BE348318 |  |  |
| 236146_at | synaptotagmin binding, cytoplasmic RNA interacting protein | SYNCRIP | BF593158 | Hs.436376 | chr6q14-q15 |
| 236140_at | glutamate-cysteine ligase, modifier subunit | GCLM | AI753488 | Hs.315562 | chr1p22.1 |
| 236128_at | zinc finger protein 91 | ZNF91 | AI672373 |  |  |
| 236123_at | Suppression of tumorigenicity 7 like | ST7L | BE220209 | Hs.146513 | chr1p13.2 |
| 236118_at | hypothetical protein LOC728473 | LOC728473 | N63706 | Hs.104573 | chr18q11.2 |
| 236075_s_at | zinc finger protein 169 | ZNF169 | AI693543 | Hs.279914 | chr17p13-p12 |
| 236047_at | chromosome 8 open reading frame 5 | C8orf5 | AA742584 | Hs.197096 | chr8p23.1 |
| 236007_at | A kinase (PRKA) anchor protein 10 | AKAP10 | AU147278 | Hs.372446 | chr17p11.1 |
| 235996_at | Ras association (RalGDS/AF-6) domain family 8 | RASSF8 | BF979984 | Hs.140821 | chr12p12.3 |
| 235979_at | complement component 7 | C7 | AI697588 | Hs.78065 | chr5p13 |
| 235956_at | KIAA1377 | KIAA1377 | AI797063 |  |  |
| 235955_at | MARVEL domain containing 2 | MARVELD2 | N57927 | Hs.444195 | chr5q13.2 |
| 235953_at | zinc finger protein 610 | ZNF610 | AA776810 | Hs.357663 | chr19q13.41 |
| 235949_at | Tetratricopeptide repeat domain 26 | TTC26 | BF664545 |  |  |
| 235944_at | hemicentin 1 | HMCN1 | BF446673 | Hs.334485 | chr1q25.3-q31.1 |
| 235931_at | Family with sequence similarity 119, member A | FAM119A | AI332764 |  |  |
| 235923_at | vesicle transport through interaction with t-SNAREs homolog 1A (yeast) | VTI1A | AA333161 | Hs.368025 | chr10q25.2 |
| 235917_at | Chromosome 9 open reading frame 80 | C9orf80 | AI950319 |  |  |
| 235911_at | Antigen p97 (melanoma associated) identified by monoclonal antibodies 133.2 and 96.5 | MFI2 | AI885815 | Hs.184727 | chr3q29 |
| 235902_at | Similar to M-phase phosphoprotein 10 | LOC729908 | AI090764 | Hs.404449 | chr15p13 |
| 235880_at | hypothetical protein FLJ37078 | FLJ37078 | BF593867 | Hs.26309 | chr7q11.23 |
| 235874_at | protease, serine, 35 | PRSS35 | AL574912 | Hs.98381 | chr6q14.2 |
| 235867_at | glutathione S-transferase M3 (brain) | GSTM3 | AI459140 | Hs.2006 | chr1p13.3 |
| 235857_at | potassium channel tetramerisation domain containing 11 | KCTD11 | AI859242 | Hs.440328 | chr17p13.1 |
| 235850_at | WD repeat domain 5B | WDR5B | BF434228 |  |  |
| 235847_at | Zinc finger, AN1-type domain 3 | ZFAND3 | BF111312 |  |  |
| 235846_at | RAD54 homolog B (S. cerevisiae) | RAD54B | AI208616 | Hs.128501 | chr8q21.3-q22 |
| 235833_at | Phosphoribosylaminoimidazole carboxylase, phosphoribosylaminoimidazole succinocarboxamide synthetase | PAICS | BF111128 | Hs.444439 | chr4pter-q21 |
| 235812_at | chromosome 16 open reading frame 69 | C16orf69 | AI935115 | Hs.138563 | chr16q12.1 |
| 235803_at | Cytokine receptor-like factor 3 | CRLF3 | AA843122 |  |  |
| 235799_at | NSL1, MIND kinetochore complex component, homolog (S. cerevisiae) | NSL1 | AW168886 | Hs.25027 |  |
| 235791_x_at | chromodomain helicase DNA binding protein 1 | CHD1 | AU155298 | Hs.311553 | chr5q15-q21 |
| 235758_at | paraneoplastic antigen like 6A | PNMA6A | BF508272 | Hs.11849 | chrxq28 |
| 235745_at | endoplasmic reticulum to nucleus signalling 1 | ERN1 | AV704183 | Hs.129166 | chr17q24.2 |
| 235744_at | PTC7 protein phosphatase homolog (S. cerevisiae) | PPTC7 | BG252924 | Hs.13854 | chr12q24.11 |
| 235729_at | zinc finger protein 514 | ZNF514 | T93113 | Hs.274414 | chr2q11.1 |
| 235728_at | zinc finger protein 3 homolog (mouse) | ZFP3 | AA845646 | Hs.48832 | chr17p13.2 |
| 235722_at | Synaptojanin 2 binding protein | SYNJ2BP | AI671431 | Hs.443661 | chr14q24.2 |
| 235721_at | deltex 3 homolog (Drosophila) | DTX3 | N62126 | Hs.32374 | chr12q13.3 |
| 235711_at | purine-rich element binding protein B | PURB | AA495775 | Hs.349150 | chr7p13 |
| 235707_at | hypothetical protein LOC221710 | LOC221710 | AW994270 |  |  |
| 235704_at | DAZ associated protein 2 | DAZAP2 | AI307251 |  |  |
| 235702_at | Zinc finger protein 225 | ZNF225 | AA507442 |  |  |
| 235691_at | Hypothetical protein LOC729970 | LOC729970 | AW269338 | Hs.297988 | chr1p21.3 |
| 235690_at | zinc finger protein 594 | ZNF594 | AA872562 | Hs.458408 | chr17p13 |
| 235689_at | mitochondrial methionyl-tRNA formyltransferase | MTFMT | AL563572 | Hs.54627 | chr15q22.31 |
| 235687_at | zinc finger protein 626 | ZNF626 | AA908777 | Hs.50158 | chr19p12 |
| 235677_at | Serine racemase | SRR | BE857570 |  |  |
| 235653_s_at | THAP domain containing 6 | THAP6 | BF685315 | Hs.352601 | chr4q21.1 |
| 235647_at | Adaptor-related protein complex 4, sigma 1 subunit | AP4S1 | AI298279 |  |  |
| 235645_at | establishment of cohesion 1 homolog 1 (S. cerevisiae) | ESCO1 | AW501507 | Hs.290931 | chr18q11.2 |
| 235635_at | Rho GTPase activating protein 5 | ARHGAP5 | N50119 |  |  |
| 235610_at | AlkB, alkylation repair homolog 8 (E. coli) | ALKBH8 | AI590659 | Hs.91559 | chr11q22.3 |
| 235604_x_at | zinc finger protein 493 | ZNF493 | AI758697 | Hs.256801 | chr19p12 |
| 235603_at | heterogeneous nuclear ribonucleoprotein U (scaffold attachment factor A) | HNRPU | N95466 |  |  |
| 235591_at | somatostatin receptor 1 | SSTR1 | R62424 | Hs.248160 | chr14q13 |
| 235587_at | hypothetical protein LOC202781 | LOC202781 | BG400596 | Hs.193172 | chr7q36.3 |
| 235579_at | Splicing factor, arginine/serine-rich 2, interacting protein | SFRS2IP | AA679858 |  |  |
| 235567_at | Hypothetical protein LOC283666 | LOC283666 | AA034012 | Hs.37648 |  |
| 235564_at | Zinc finger protein 117 | ZNF117 | BF107006 | Hs.250693 | chr7q11.2 |
| 235552_at | KIAA1627 protein | KIAA1627 | AA354181 | Hs.158958 | chr4q26 |
| 235548_at | adenomatosis polyposis coli down-regulated 1-like | APCDD1L | BG326592 | Hs.119286 | chr20q13.32 |
| 235536_at | ring finger protein 149 /// similar to ring finger protein 149 | RNF149 | AI640483 | Hs.171802 | chr2q11.2 |
| 235535_x_at | FSHD region gene 1 /// similar to FRG1 protein (FSHD region gene 1 protein) /// similar to FRG1 protein (FSHD region gene 1 protein) /// similar to FRG1 protein (FSHD region gene 1 protein) | FRG1 | AI369073 | Hs.203772 | chr4q35 /// chr20q11.1 |
| 235527_at | hypothetical protein LOC284214 | LOC284214 | U55983 |  | chr18p11.31 |
| 235526_at | SRY (sex determining region Y)-box 6 | SOX6 | BE748802 | Hs.78050 | chr11p15.1 |
| 235520_at | suppressor of hairy wing homolog 3 (Drosophila) | SUHW3 | AA805654 | Hs.308418 | chrxq25 |
| 235509_at | chromosome 8 open reading frame 38 | C8orf38 | AV662196 | Hs.435500 | chr8q22.1 |
| 235497_at | hypothetical protein LOC643837 | LOC643837 | AL079648 | Hs.129660 | chr1p36.33 |
| 235484_at | protein prenyltransferase alpha subunit repeat containing 1 | PTAR1 | BE892889 | Hs.220677 | chr9q21.11 |
| 235482_at | hypothetical gene supported by BC040598 | LOC400960 | BE886868 |  | chr2p13.3 |
| 235479_at | cytoplasmic polyadenylation element binding protein 2 | CPEB2 | AI948598 | Hs.44833 | chr4p15.33 |
| 235476_at | tripartite motif-containing 59 | TRIM59 | AW182459 | Hs.534739 | chr3q25.33 |
| 235473_at | Mediator of RNA polymerase II transcription, subunit 6 homolog (S. cerevisiae) | MED6 | BF434383 |  |  |
| 235471_at | Chromosome 10 open reading frame 72 | C10orf72 | BE858464 | Hs.279639 | chr10q11.23 |
| 235466_s_at | Dispatched homolog 1 (Drosophila) | DISP1 | AI677948 |  |  |
| 235463_s_at | LAG1 homolog, ceramide synthase 6 (S. cerevisiae) | LASS6 | AI081356 | Hs.387400 | chr2q24.3 |
| 235450_at | F-box and leucine-rich repeat protein 4 /// CDNA FLJ30658 fis, clone DFNES2000432 | FBXL4 | BF571480 | Hs.132809 |  |
| 235443_at | Creatine kinase, mitochondrial 2 (sarcomeric) | CKMT2 | BG284827 | Hs.193632 |  |
| 235440_at | SPT2, Suppressor of Ty, domain containing 1 (S. cerevisiae) | SPTY2D1 | BE780878 | Hs.356030 | chr11p15.1 |
| 235433_at | family with sequence similarity 121A | FAM121A | BG290639 | Hs.356269 | chrXq21.1 |
| 235432_at | nephronophthisis 3 (adolescent) | NPHP3 | BE865779 | Hs.511991 | chr3q22.1 |
| 235429_at | Eukaryotic translation initiation factor 3, subunit 6 48kDa | EIF3S6 | AW965494 |  |  |
| 235419_at | ERBB receptor feedback inhibitor 1 | ERRFI1 | AW612461 |  |  |
| 235411_at | piggyBac transposable element derived 1 | PGBD1 | BE883300 | Hs.144527 | chr6p22.1 |
| 235405_at | glutathione S-transferase A4 | GSTA4 | N79662 | Hs.169907 | chr6p12.1 |
| 235399_at | Kinesin light chain 3 | KLC3 | AA682499 |  |  |
| 235398_at | zinc finger protein 805 | ZNF805 | BF109670 |  |  |
| 235392_at | Insulin receptor substrate 1 | IRS1 | BG403162 |  |  |
| 235388_at | chromodomain helicase DNA binding protein 9 | CHD9 | BG538482 | Hs.119053 | chr16q12.2 |
| 235378_at | Chromosome 14 open reading frame 44 | C14orf44 | AI082216 |  |  |
| 235374_at | Malate dehydrogenase 1, NAD (soluble) | MDH1 | AW952547 |  |  |
| 235371_at | similar to CG9996-PA | LOC727936 | AI452595 | Hs.97600 | chr3p13 |
| 235356_at | NHL repeat containing 2 | NHLRC2 | AW297204 | Hs.435722 | chr10q25.3 |
| 235354_s_at | Arginine/serine-rich coiled-coil 1 | RSRC1 | BG398744 |  |  |
| 235349_at | family with sequence similarity 82, member A | FAM82A | AI261321 | Hs.9905 | chr2p22.2 |
| 235348_at | abhydrolase domain containing 13 | ABHD13 | AA404347 | Hs.183528 | chr13q33.3 |
| 235346_at | FUN14 domain containing 1 | FUNDC1 | BF033615 | Hs.7549 | chrxp11.3 |
| 235338_s_at | SET domain, bifurcated 2 | SETDB2 | W65369 | Hs.446217 | chr13q14 |
| 235334_at | ST6 (alpha-N-acetyl-neuraminyl-2,3-beta-galactosyl-1,3)-N-acetylgalactosaminide alpha-2,6-sialyltransferase 3 | ST6GALNAC3 | AW963951 | Hs.85618 | chr1p31.1 |
| 235333_at | UDP-Gal:betaGlcNAc beta 1,4- galactosyltransferase, polypeptide 6 | B4GALT6 | BG503479 |  |  |
| 235327_x_at | UBX domain containing 4 | UBXD4 | BG111015 | Hs.528329 | chr2p23.3 |
| 235324_at | Splicing factor, arginine/serine-rich 3 | SFRS3 | AI445255 |  |  |
| 235320_at | ADP-ribosylation factor-like 6 | ARL6 | AL138043 | Hs.196918 | chr3q11.2 |
| 235315_at | TSC22 domain family, member 1 | TSC22D1 | AI809519 |  |  |
| 235304_at | Zinc finger protein 573 | ZNF573 | AI400209 |  |  |
| 235301_at | KIAA1324-like | KIAA1324L | AI797353 | Hs.224507 | chr7q21.12 |
| 235300_x_at | ring finger and CHY zinc finger domain containing 1 | RCHY1 | AW236209 |  |  |
| 235289_at | eukaryotic translation initiation factor 5A2 | EIF5A2 | AV747725 | Hs.164144 | chr3q26.2 |
| 235275_at | 3-oxoacid CoA transferase 2 | OXCT2 | AA610122 | Hs.534309 | chr1p35-p32 |
| 235274_at | GA binding protein transcription factor, beta subunit 2 | GABPB2 | AA740632 |  |  |
| 235266_at | ATPase family, AAA domain containing 2 | ATAD2 | AI139629 | Hs.298646 | chr8q24.13 |
| 235260_s_at | chromosome 4 open reading frame 28 | C4orf28 | AA398590 | Hs.388749 | chr4p15.31 |
| 235258_at | DCP2 decapping enzyme homolog (S. cerevisiae) | DCP2 | AI873425 | Hs.442039 | chr5q22.2 |
| 235256_s_at | galactose mutarotase (aldose 1-epimerase) | GALM | BE788984 | Hs.264610 | chr2p22.1 |
| 235253_at | RAD1 homolog (S. pombe) | RAD1 | AI742925 | Hs.7179 | chr5p13.2 |
| 235252_at | Kinase suppressor of ras 1 | KSR1 | AI090141 | Hs.276238 | chr17q11.2 |
| 235241_at | hypothetical protein FLJ90709 | FLJ90709 | BF696757 | Hs.128696 | chr5q11.2 |
| 235230_at | Phosphatidylinositol-specific phospholipase C, X domain containing 2 | PLCXD2 | AW170015 | Hs.7378 | chr3q13.2 |
| 235223_at | General transcription factor IIIC, polypeptide 2, beta 110kDa | GTF3C2 | AW515799 |  |  |
| 235219_at | hypothetical protein BC014011 | LOC116349 | BE897119 | Hs.446702 | chr5p15.33 |
| 235208_at | coiled-coil domain containing 112 | CCDC112 | AW157712 | Hs.47534 | chr5q22.3 |
| 235202_x_at | IKK interacting protein | IKIP | BG498328 | Hs.406199 | chr12q23.1 |
| 235201_at | Forkhead box P2 | FOXP2 | AW167727 |  |  |
| 235200_at | zinc finger protein 561 | ZNF561 | AL135342 |  |  |
| 235197_s_at | osteopetrosis associated transmembrane protein 1 | OSTM1 | AV713913 | Hs.163724 | chr6q21 |
| 235195_at | F-box and WD-40 domain protein 2 | FBXW2 | BG109988 | Hs.444354 | chr9q34 |
| 235182_at | chromosome 20 open reading frame 82 | C20orf82 | AI816793 | Hs.348297 | chr20p12.1 |
| 235179_at | zinc finger protein 641 | ZNF641 | BF541598 | Hs.23492 | chr12q13.11 |
| 235174_s_at | Hypothetical protein LOC728923 | LOC728923 | AW952781 | Hs.302631 |  |
| 235173_at | hypothetical LOC401093 | LOC401093 | AA093668 | Hs.534938 | chr3q25.1 |
| 235170_at | zinc finger protein 92 | ZNF92 | T52999 | Hs.9521 | chr7q11.21 |
| 235167_at | hypothetical gene LOC283846 | DKFZp547E087 | BE972419 | Hs.375005 /// Hs.460278 /// Hs.513480 | chr16p12.2 /// chr16p12.1 /// chr16p11.2 |
| 235164_at | zinc finger protein 25 | ZNF25 | BG433539 | Hs.5856 | chr10p11.21 |
| 235159_at | Transmembrane protein 140 | TMEM140 | AW296028 |  |  |
| 235158_at | hypothetical protein FLJ14803 | FLJ14803 | AI807036 | Hs.267245 | chr7q32.2 |
| 235154_at | TAF3 RNA polymerase II, TATA box binding protein (TBP)-associated factor, 140kDa | TAF3 | BG250498 | Hs.130285 | chr10p15.1 |
| 235151_at | hypothetical protein LOC283357 | LOC283357 | AA807060 | Hs.126558 | chr12p13.33 |
| 235143_at | Chromosome 4 open reading frame 13 | C4orf13 | AI963571 | Hs.146162 |  |
| 235142_at | zinc finger and BTB domain containing 8 /// similar to zinc finger and BTB domain containing 8 | ZBTB8 | AW006067 | Hs.129837 | chr1p35.1 |
| 235138_at | Vacuolar protein sorting 35 (yeast) | VPS35 | AA565051 |  |  |
| 235132_at | hypothetical protein LOC254128 | LOC254128 | BF939830 | Hs.296995 | chr2q14.2 |
| 235124_at | Hypothetical LOC645212 | LOC645212 | BE502930 | Hs.153400 |  |
| 235117_at | ChaC, cation transport regulator homolog 2 (E. coli) | CHAC2 | AI191897 | Hs.333184 |  |
| 235114_x_at | hook homolog 3 (Drosophila) | HOOK3 | N67300 | Hs.162852 | chr8p11.21 |
| 235113_at | peptidylprolyl isomerase (cyclophilin)-like 5 | PPIL5 | AA742244 | Hs.374811 | chr14q21.3 |
| 235099_at | CKLF-like MARVEL transmembrane domain containing 8 | CMTM8 | AW080832 | Hs.154986 | chr3p23 |
| 235096_at | Leo1, Paf1/RNA polymerase II complex component, homolog (S. cerevisiae) | LEO1 | AA074729 | Hs.511399 | chr15q21.2 |
| 235094_at | Tropomyosin 4 | TPM4 | AI972661 | Hs.419445 | chr19p13.1 |
| 235088_at | hypothetical protein LOC201725 | LOC201725 | AI276663 | Hs.380920 | chr4q32.1 |
| 235086_at | Thrombospondin 1 | THBS1 | AW956580 | Hs.164226 | chr15q15 |
| 235081_x_at | tripartite motif-containing 65 | TRIM65 | AI739057 | Hs.189823 | chr17q25.1 |
| 235078_at | Neurobeachin-like 1 | NBEAL1 | AI393725 |  |  |
| 235074_at | Sprouty-related, EVH1 domain containing 1 | SPRED1 | BF342524 |  |  |
| 235071_at | monad | LOC116143 | AI653809 | Hs.493450 | chr2p14 |
| 235061_at | protein phosphatase 1K (PP2C domain containing) | PPM1K | AV706522 | Hs.291000 | chr4q22.1 |
| 235059_at | RAB12, member RAS oncogene family | RAB12 | BF574430 | Hs.187585 | chr18p11.22 |
| 235056_at | ets variant gene 6 (TEL oncogene) | ETV6 | AV722693 | Hs.504765 | chr12p13 |
| 235054_at | nudix (nucleoside diphosphate linked moiety X)-type motif 16 | NUDT16 | BF941983 | Hs.282050 | chr3q21.3 |
| 235045_at | RNA binding motif protein 7 | RBM7 | AI493853 |  |  |
| 235044_at | cysteine/tyrosine-rich 1 | CYYR1 | H06649 | Hs.132275 | chr21q21.2 |
| 235040_at | RUN domain containing 1 | RUNDC1 | BG168618 | Hs.218182 | chr17q21.31 |
| 235033_at | Aminopeptidase-like 1 | NPEPL1 | AL577823 | Hs.378135 | chr20q13.32 |
| 235024_at | PHD finger protein 17 | PHF17 | AI868315 | Hs.12420 | chr4q26-q27 |
| 235022_at | chromosome 18 open reading frame 19 | C18orf19 | BE326738 | Hs.13034 | chr18p11.21 |
| 235020_at | TAF4b RNA polymerase II, TATA box binding protein (TBP)-associated factor, 105kDa | TAF4B | AI366784 | Hs.192130 | chr18q11.2 |
| 235016_at | receptor accessory protein 3 | REEP3 | AL118571 | Hs.352398 | chr10q21.3 |
| 235014_at | Hypothetical protein LOC147727 | LOC147727 | BF345728 |  |  |
| 235013_at | solute carrier family 31 (copper transporters), member 1 | SLC31A1 | AI754064 |  |  |
| 235009_at | family with sequence similarity 44, member A | FAM44A | AI049791 | Hs.444517 | chr4p16.1 |
| 235006_at | similar to RIKEN cDNA A430101B06 gene | MGC13017 | AL571598 | Hs.156506 | chr5q31.1 |
| 235005_at | hypothetical protein MGC4562 | MGC4562 | AA192361 | Hs.269496 | chr15q22.31 |
| 234995_at | coiled-coil domain containing 52 | CCDC52 | AA668779 | Hs.72402 | chr3q13.2 |
| 234994_at | KIAA1913 | KIAA1913 | AA088177 | Hs.172870 | chr6q23.1 |
| 234991_at | ZXD family zinc finger C | ZXDC | AA702187 |  |  |
| 234988_at | valosin containing protein (p97)/p47 complex interacting protein 1 | VCPIP1 | AW629515 | Hs.287727 | chr8q13 |
| 234987_at | Chromosome 20 open reading frame 118 | C20orf118 | AV715309 | Hs.371264 | chr20pter-q12 |
| 234979_at | hypothetical protein LOC144233 | LOC144233 | AI803078 | Hs.142736 | chr12q13.12 |
| 234978_at | solute carrier family 36 (proton/amino acid symporter), member 4 | SLC36A4 | AL542358 | Hs.292653 | chr11q21 |
| 234972_at | ADP-ribosylation factor-like 16 | ARL16 | BE746724 | Hs.306806 | chr17q25.3 |
| 234954_at | similar to Glyceraldehyde-3-phosphate dehydrogenase (GAPDH) /// similar to Glyceraldehyde-3-phosphate dehydrogenase (GAPDH) | LOC442262 | AL035604 |  |  |
| 234947_s_at | chromosome 10 open reading frame 84 | C10orf84 | AK026630 | Hs.11859 | chr10q26.11 |
| 234937_x_at | zinc finger protein 28 homolog (mouse) | ZFP28 | AC007228 | Hs.14794 | chr19q13.43 |
| 234936_s_at | KIAA1345 protein | KIAA1345 | AK023876 | Hs.303820 | chr4p15.33 |
| 234929_s_at | spermatogenesis associated 7 | SPATA7 | AF144488 | Hs.414464 | chr14q31.3 |
| 234923_at | GTPase activating Rap/RanGAP domain-like 1 | GARNL1 | AK022988 | Hs.167031 | chr14q13.2 |
| 234915_s_at | density-regulated protein | DENR | BC000925 | Hs.22393 | chr12q24.31 |
| 234902_s_at | zinc finger protein 416 | ZNF416 | AC003682 | Hs.247711 | chr19q13.4 |
| 234875_at | similar to 60S ribosomal protein L7a /// similar to 60S ribosomal protein L7a | LOC729926 | AJ224082 |  |  |
| 234788_x_at | Hypothetical protein FLJ13611 | FLJ13611 | AK024819 | Hs.184664 | chr5q12.3 |
| 234723_x_at | Hypothetical LOC641808 | LOC641808 | AK024881 | Hs.306716 |  |
| 234710_s_at | poly (ADP-ribose) polymerase family, member 6 | PARP6 | AK021790 | Hs.8694 | chr15q23 |
| 234697_x_at | chromosome 3 open reading frame 31 | C3orf31 | AL136788 | Hs.370293 | chr3p25.2 |
| 234687_x_at | LIM domains containing 1 | LIMD1 | M80651 | Hs.193370 | chr3p21.3 |
| 234583_at | Cell adhesion molecule with homology to L1CAM (close homolog of L1) | CHL1 | AL359583 | Hs.388344 | chr3p26.1 |
| 234562_x_at | hypothetical protein LOC728678 /// hypothetical protein LOC731914 | LOC728678 | AK000115 | Hs.154986 | chr3p23 |
| 234512_x_at | similar to 60S ribosomal protein L7a (Surfeit locus protein 3) | LOC728179 | AL136226 | Hs.535303 | chr6p24.3 |
| 234488_s_at | germ cell-less homolog 1 (Drosophila) /// germ cell-less homolog 1 (Drosophila)-like | GMCL1 | AF198534 | Hs.484313 | chr5q35.3 /// chr2p13.3 |
| 234485_at | similar to eukaryotic translation initiation factor 3, subunit 6 interacting protein /// hypothetical protein LOC732107 | LOC340947 | AL117339 | Hs.164084 | chr10p11.21 |
| 234464_s_at | essential meiotic endonuclease 1 homolog 1 (S. pombe) | EME1 | AK021607 | Hs.370927 | chr17q21.33 |
| 234405_s_at | RNA U, small nuclear RNA export adaptor (phosphorylation regulated) | RNUXA | AK024065 | Hs.339612 | chr5q23.2 |
| 234344_at | RAP2C, member of RAS oncogene family | RAP2C | AF093744 | Hs.119889 | chrXq25 |
| 234339_s_at | glioma tumor suppressor candidate region gene 2 | GLTSCR2 | AF296124 | Hs.421907 | chr19q13.3 |
| 234312_s_at | acyl-CoA synthetase short-chain family member 2 | ACSS2 | AK000162 | Hs.189998 | chr20q11.22 |
| 234311_s_at | GTP-binding protein 10 (putative) | GTPBP10 | AC006153 | Hs.131729 | chr7q21.13 |
| 234192_s_at | G kinase anchoring protein 1 | GKAP1 | AK026487 | Hs.147568 | chr9q21.32 |
| 234118_at | Phosphodiesterase 7B | PDE7B | AK000141 | Hs.368024 | chr6q23-q24 |
| 234107_s_at | histidyl-tRNA synthetase 2 | HARS2 | AL121780 | Hs.444706 | chr20p11.23 |
| 234103_at | Potassium channel, subfamily T, member 2 | KCNT2 | AU145191 | Hs.444376 | chr1q31.3 |
| 234032_at | Zinc finger, CCHC domain containing 7 | ZCCHC7 | AF119847 |  |  |
| 233977_at | KIAA1772 | KIAA1772 | AB051559 | Hs.54838 | chr18q11.1-q11.2 |
| 233974_s_at | family with sequence similarity 129, member B | FAM129B | AK023580 | Hs.109857 | chr9q34.11 |
| 233946_at | Smu-1 suppressor of mec-8 and unc-52 homolog (C. elegans) | SMU1 | AL512690 | Hs.327749 | chr9p21.1 |
| 233943_x_at | pleckstrin homology domain containing, family A (phosphoinositide binding specific) member 8 | PLEKHA8 | AF308300 | Hs.358898 | chr7p21-p11.2 |
| 233924_s_at | exocyst complex component 6 | EXOC6 | AK002113 | Hs.292097 | chr10q23.33 |
| 233914_s_at | SET binding factor 2 | SBF2 | AK022478 | Hs.438794 | chr11p15.4 |
| 233893_s_at | KIAA1530 protein | KIAA1530 | AB040963 | Hs.380475 | chr4p16.3 |
| 233888_s_at | SLIT-ROBO Rho GTPase activating protein 1 | SRGAP1 | AK023899 | Hs.450763 | chr12q14.2 |
| 233878_s_at | 5'-3' exoribonuclease 2 | XRN2 | BE536170 | Hs.268555 | chr20p11.2-p11.1 |
| 233852_at | polymerase (DNA directed), eta | POLH | AK025631 | Hs.439153 | chr6p21.1 |
| 233841_s_at | suppressor of defective silencing 3 homolog (S. cerevisiae) | SUDS3 | AK026749 | Hs.416630 | chr12q24.23 |
| 233824_at | LIM domain containing preferred translocation partner in lipoma | LPP | AK025081 | Hs.287663 |  |
| 233819_s_at | zinc finger protein 294 | ZNF294 | AK023499 | Hs.288773 | chr21q22.11 |
| 233810_x_at | PRO1268 protein | PRO1268 | AF113017 |  |  |
| 233750_s_at | chromosome 1 open reading frame 25 | C1orf25 | AL109865 | Hs.107149 | chr1q25.2 |
| 233746_x_at | Huntingtin interacting protein K | HYPK | AW953521 | Hs.511978 | chr15q15.3 |
| 233734_s_at | oxysterol binding protein-like 5 | OSBPL5 | AW271225 | Hs.309576 | chr11p15.4 |
| 233720_at | Sorbin and SH3 domain containing 2 | SORBS2 | AK000782 | Hs.156880 | chr4q35.1 |
| 233657_at | opsin 5 | OPN5 | AI810121 | Hs.213717 | chr6p12.3 |
| 233655_s_at | family with sequence similarity 29, member A | FAM29A | AK022964 | Hs.54617 | chr9p22.1 |
| 233642_s_at | KIAA1414 protein | KIAA1414 | AK001513 | Hs.92991 | chr2p22.2 |
| 233638_s_at | protein O-linked mannose beta1,2-N-acetylglucosaminyltransferase | POMGNT1 | AK026430 | Hs.435108 | chr1p34.1 |
| 233595_at | ubiquitin specific peptidase 34 | USP34 | AK024341 | Hs.507665 | chr2p15 |
| 233563_s_at | cleavage and polyadenylation specific factor 3-like | CPSF3L | AK023356 | Hs.6449 | chr1p36.33 |
| 233559_s_at | WD repeat and FYVE domain containing 1 | WDFY1 | AK023415 | Hs.44743 | chr2q36.1 |
| 233557_s_at | MON1 homolog B (yeast) | MON1B | AK023374 | Hs.513742 | chr16q23.1 |
| 233555_s_at | sulfatase 2 | SULF2 | AL034418 | Hs.43857 | chr20q12-q13.2 |
| 233543_s_at | coiled-coil domain containing 98 | CCDC98 | AK021582 | Hs.334772 | chr4q21.21-q21.23 |
| 233532_x_at | intraflagellar transport 52 homolog (Chlamydomonas) | IFT52 | N47376 | Hs.24994 | chr20q12-q13.1 |
| 233528_s_at | hypothetical protein LOC652968 | LOC652968 | AC004997 | Hs.505579 | chr22q12.1-qter |
| 233518_at | Similar to lymphocyte-specific protein 1 | LOC654342 | AU144449 | Hs.469287 | chr2p11.1 |
| 233490_at | dynactin 4 (p62) | DCTN4 | AI985890 |  |  |
| 233411_at | Protein kinase C, eta | PRKCH | AU147253 | Hs.315366 | chr14q22-q23 |
| 233399_x_at | Transmembrane emp24-like trafficking protein 10 (yeast) | TMED10 | AU145662 | Hs.367856 | chr8q24.3 |
| 233375_at | EF-hand calcium binding domain 2 | EFCAB2 | AK001393 | Hs.438469 | chr1q44 |
| 233321_x_at | hypothetical protein BC001742 | LOC90834 | BC001742 |  | chr22q13.33 |
| 233319_x_at | Phosphatase and actin regulator 4 | PHACTR4 | AK023907 | Hs.225641 | chr1p35.3 |
| 233307_x_at | Microsomal glutathione S-transferase 3 | MGST3 | AK000073 | Hs.212507 | chr1q23 |
| 233296_x_at | Zinc finger and BTB domain containing 20 | ZBTB20 | AU147291 | Hs.270591 |  |
| 233276_at | F-box and leucine-rich repeat protein 7 | FBXL7 | AU146390 | Hs.76798 | chr5p15.1 |
| 233270_x_at | Pericentriolar material 1 | PCM1 | AU146939 | Hs.348501 | chr8p22-p21.3 |
| 233255_s_at | basic, immunoglobulin-like variable motif containing | BIVM | AF339793 | Hs.288809 | chr13q32-q33.1 |
| 233222_at | Glypican 6 | GPC6 | AF339789 | Hs.508411 | chr13q32 |
| 233219_at | Muskelin 1, intracellular mediator containing kelch motifs | MKLN1 | AK023427 | Hs.145599 | chr7q32 |
| 233208_x_at | cleavage and polyadenylation specific factor 2, 100kDa | CPSF2 | AA583986 | Hs.435675 | chr14q31.1 |
| 233204_at | similar to hypothetical protein MGC40405 | FLJ11903 | AA115105 |  |  |
| 233191_at | RUN and FYVE domain containing 2 | RUFY2 | AB040970 | Hs.534534 | chr10q21.3 |
| 233182_x_at | Ataxin 3 | ATXN3 | AU146105 | Hs.419756 |  |
| 233179_x_at | sirtuin (silent mating type information regulation 2 homolog) 6 (S. cerevisiae) | SIRT6 | AL365375 | Hs.423756 | chr19p13.3 |
| 233177_s_at | paroxysmal nonkinesiogenic dyskinesia | PNKD | AB033010 | Hs.516697 | chr2q35 |
| 233173_x_at | general transcription factor IIIC, polypeptide 5, 63kDa | GTF3C5 | AK000194 | Hs.286088 | chr9q34 |
| 233168_s_at | selenoprotein O | RP3-402G11.5 | BC001099 | Hs.365405 | chr22q13.33 |
| 233164_x_at | rhomboid domain containing 1 | RHBDD1 | AK026955 | Hs.233419 | chr2q36.3 |
| 233152_x_at | Tankyrase, TRF1-interacting ankyrin-related ADP-ribose polymerase | TNKS | AL049979 | Hs.409194 | chr8p23.1 |
| 233124_s_at | enoyl Coenzyme A hydratase domain containing 1 | ECHDC1 | AK021464 | Hs.437091 | chr6q22.33 |
| 233112_at | Chromosome 9 open reading frame 150 | C9orf150 | AW301393 | Hs.445356 | chr9p23 |
| 233110_s_at | BCL2-like 12 (proline rich) | BCL2L12 | AF289220 | Hs.289052 | chr19q13.3 |
| 233106_at | chromosome 14 open reading frame 82 | C14orf82 | AU148054 | Hs.367825 | chr14q22.1 |
| 233085_s_at | oligonucleotide/oligosaccharide-binding fold containing 2A | OBFC2A | AV734843 | Hs.118183 | chr2q32.3 |
| 233082_at | zinc finger protein 630 | ZNF630 | AK000580 | Hs.130534 | chrXp11.3-p11.1 |
| 233070_at | zinc finger protein 197 | ZNF197 | AL117657 | Hs.157035 | chr3p21 |
| 233056_x_at | discs, large (Drosophila) homolog-associated protein 4 | DLGAP4 | AK024674 | Hs.249600 | chr20q11.23 |
| 233041_x_at | BTB (POZ) domain containing 9 | BTBD9 | AK025009 | Hs.127729 | chr6p21 |
| 233017_x_at | Mitogen activated protein kinase binding protein 1 | MAPKBP1 | AK022388 | Hs.513661 |  |
| 233003_at | Development and differentiation enhancing factor 1 | DDEF1 | AK022004 |  |  |
| 232964_at | Williams Beuren syndrome chromosome region 19 | WBSCR19 | AL137266 | Hs.512067 | chr7p13 |
| 232946_s_at | NAD synthetase 1 | NADSYN1 | AL512694 | Hs.436071 | chr11q13.4 |
| 232935_at | Lipoma HMGIC fusion partner | LHFP | AA569225 | Hs.93765 | chr13q12 |
| 232902_s_at | arginyl-tRNA synthetase-like | RARSL | BG165118 | Hs.432918 | chr6q16.1 |
| 232899_at | ribosomal protein L23a pseudogene 7 /// family with sequence similarity 41, member C /// similar to RPL23AP7 protein | RPL23AP7 | R17536 | Hs.449006 | chr1p36.33 /// chr22q13.33 |
| 232864_s_at | AF4/FMR2 family, member 4 | AFF4 | N59653 | Hs.446658 | chr5q31 |
| 232811_x_at | Prickle homolog 1 (Drosophila) | PRICKLE1 | AU145382 | Hs.6786 | chr12q12 |
| 232800_at | Similar to sorting nexin associated golgi protein 1 | LOC730943 | AW086077 | Hs.153272 |  |
| 232785_at | retrotransposon gag domain containing 1 | RGAG1 | AI807872 | Hs.201071 | chrxq23 |
| 232773_at | 3-hydroxyisobutyryl-Coenzyme A hydrolase | HIBCH | AI932618 | Hs.389311 | chr2q32.2 |
| 232744_x_at | Ataxin 1 | ATXN1 | BG485129 | Hs.484773 |  |
| 232720_at | Leucine rich repeat neuronal 6C | LRRN6C | AL353746 | Hs.145642 | chr9p21.2-p21.1 |
| 232710_at | POM121 membrane glycoprotein (rat) | POM121 | AK021477 | Hs.520965 | chr7q11.23 |
| 232693_s_at | zinc finger protein 395 /// F-box protein 16 | ZNF395 | AK021850 | Hs.32352 | chr8p21.1 |
| 232668_at | Chromosome 8 open reading frame 72 | C8orf72 | AW903934 | Hs.34054 | chr8q12.1 |
| 232661_s_at | hypothetical protein DKFZp564O0523 | DKFZP564O0523 | AF161422 | Hs.21590 | chr7q21.2 |
| 232652_x_at | SCAN domain containing 1 | SCAND1 | AF207829 | Hs.274411 | chr20q11.1-q11.23 |
| 232649_at | gliomedin | GLDN | AU157510 | Hs.268764 | chr15q21.2 |
| 232641_at | zinc finger protein 596 | ZNF596 | AC004908 | Hs.249181 | chr8p23.3 |
| 232636_at | SLIT and NTRK-like family, member 4 | SLITRK4 | AL080239 | Hs.272284 | chrxq27.3 |
| 232617_at | cathepsin S | CTSS | AK024855 | Hs.181301 | chr1q21 |
| 232600_at | Ankyrin repeat domain 42 | ANKRD42 | AL137429 | Hs.170234 | chr11q14.1 |
| 232594_at | Hypothetical gene supported by AK001829 | LOC440498 | AK001829 | Hs.210305 | chr18q23 |
| 232591_s_at | transmembrane protein 30A | TMEM30A | AK022883 | Hs.108530 | chr6q14.1 |
| 232584_at | Teashirt family zinc finger 2 | TSHZ2 | AU147926 | Hs.150825 | chr20q13.2 |
| 232569_at | Chromosome 7 open reading frame 44 | C7orf44 | AK024255 | Hs.162990 |  |
| 232563_at | zinc finger protein 684 | ZNF684 | AW275016 | Hs.292343 | chr1p34.2 |
| 232553_at | phosphate cytidylyltransferase 1, choline, beta | PCYT1B | AI857508 |  |  |
| 232543_x_at | Rho GTPase activating protein 9 | ARHGAP9 | BE675685 | Hs.437126 | chr12q14 |
| 232541_at | Epidermal growth factor receptor (erythroblastic leukemia viral (v-erb-b) oncogene homolog, avian) | EGFR | AK000106 | Hs.133100 |  |
| 232531_at | empty spiracles homolog 2 (Drosophila) opposite strand | EMX2OS | AL137578 | Hs.312592 | chr10q26.12 |
| 232524_x_at | anaphase promoting complex subunit 4 | ANAPC4 | AK001881 | Hs.436579 | chr4p15.2 |
| 232511_at | RANBP2-like and GRIP domain containing 5 | RGPD5 | AK022838 | Hs.434959 | chr2q13 |
| 232488_at | alanine-glyoxylate aminotransferase 2-like 2 | AGXT2L2 | AK023470 | Hs.534604 | chr5q35.3 |
| 232469_x_at | Chromosome 1 open reading frame 191 | C1orf191 | AK023883 | Hs.288801 | chr1p32.3 |
| 232463_at | chromosome X and Y open reading frame 10 | CXYorf10 | T77995 | Hs.287570 | chrxp22.33; yp11.3 |
| 232455_x_at | hypothetical protein LOC340085 | LOC340085 | AU145408 |  | chr5q11.2 |
| 232436_at | zinc finger protein 274 | ZNF274 | AI057616 | Hs.83761 | chr19qter |
| 232421_at | scavenger receptor class B, member 1 | SCARB1 | AV703311 | Hs.130981 | chr12q24.31 |
| 232420_x_at | hypothetical protein LOC286260 | LOC286260 | AA706480 | Hs.158966 | chr9q34.3 |
| 232389_at | WAS/WASL interacting protein family, member 3 | WIPF3 | AW444467 | Hs.143503 |  |
| 232387_at | AP1 gamma subunit binding protein 1 | AP1GBP1 | AK025700 | Hs.292087 | chr17q12 |
| 232371_at | Membrane-associated ring finger (C3HC4) 7 | 07-mars | AU155401 | Hs.2441 | chr2q24.2 |
| 232366_at | KIAA0232 gene product | KIAA0232 | AF143884 | Hs.79276 | chr4p16.1 |
| 232344_at | RAS p21 protein activator (GTPase activating protein) 1 | RASA1 | AK021812 | Hs.758 | chr5q13.3 |
| 232341_x_at | hyaluronan binding protein 4 | HABP4 | AK025144 | Hs.301839 | chr9q22.3-q31 |
| 232338_at | Zinc finger protein 714 | ZNF714 | AU144815 | Hs.285657 | chr19p12 |
| 232336_at | zinc finger, SWIM-type containing 5 | ZSWIM5 | AI479419 | Hs.135673 | chr1p34.1 |
| 232331_at | Peroxisome proliferator-activated receptor gamma | PPARG | AK027107 | Hs.387667 | chr3p25 |
| 232313_at | transmembrane protein 132C | TMEM132C | AL122107 | Hs.49599 | chr12q24.32 |
| 232293_at | ligand dependent nuclear receptor corepressor-like | LCORL | AL133031 | Hs.443468 | chr4p15.32 |
| 232283_at | LysM, putative peptidoglycan-binding, domain containing 1 | LYSMD1 | AL122088 | Hs.528504 | chr1q21.2 |
| 232272_at | zinc finger protein 624 | ZNF624 | BF435259 | Hs.128078 | chr17p11.2 |
| 232270_at | Chromosome 9 open reading frame 3 | C9orf3 | AL137535 | Hs.494517 |  |
| 232267_at | G protein-coupled receptor 133 | GPR133 | AL162032 | Hs.130920 | chr12q24.33 |
| 232264_at | E3 ubiquitin protein ligase, HECT domain containing, 1 | EDD1 | AK022204 | Hs.94262 | chr8q22 |
| 232254_at | F-box protein 25 | FBXO25 | AI953354 | Hs.7063 |  |
| 232239_at | Hypothetical LOC643529 | LOC643529 | AL137595 | Hs.142517 | chr10q23.31 |
| 232235_at | chromosome 18 open reading frame 4 | C18orf4 | AK021539 | Hs.124673 | chr18q22.1 |
| 232229_at | senataxin | SETX | AK024331 | Hs.460317 | chr9q34.13 |
| 232221_x_at | round spermatid basic protein 1-like | RSBN1L | AC004955 | Hs.444823 | chr7q11.23 |
| 232219_x_at | ubiquitin specific peptidase 21 | USP21 | AL157417 | Hs.8015 | chr1q22 |
| 232218_at | Chromosome 9 open reading frame 119 | C9orf119 | AI937687 |  |  |
| 232216_at | YME1-like 1 (S. cerevisiae) | YME1L1 | AA828049 | Hs.439510 | chr10p14 |
| 232206_at | unc-51-like kinase 4 (C. elegans) | ULK4 | AL133104 | Hs.255960 | chr3p21.33 |
| 232197_x_at | arylsulfatase B | ARSB | AW168942 | Hs.149103 | chr5p11-q13 |
| 232195_at | G protein-coupled receptor 158 | GPR158 | R41459 | Hs.21896 | chr10p12.1 |
| 232194_at | methyltransferase like 4 | METTL4 | AA764787 | Hs.122546 | chr18p11.32 |
| 232183_at | serine active site containing 1 | SERAC1 | AA128978 | Hs.154706 | chr6q25.3 |
| 232182_at | hypothetical protein LOC286272 | LOC286272 | AI142853 | Hs.494047 | chr9q12 |
| 232171_x_at | kelch domain containing 4 | KLHDC4 | AK001742 | Hs.67991 | chr16q24.3 |
| 232160_s_at | TNFAIP3 interacting protein 2 | TNIP2 | AL137262 | Hs.325630 | chr4p16.3 |
| 232155_at | KIAA1618 | KIAA1618 | AK023113 | Hs.437033 | chr17q25.3 |
| 232149_s_at | neutral sphingomyelinase (N-SMase) activation associated factor | NSMAF | BF056507 | Hs.372000 | chr8q12-q13 |
| 232145_at | hypothetical LOC388969 | LOC388969 | AU147777 | Hs.370214 | chr2p11.2 |
| 232103_at | 3'(2'), 5'-bisphosphate nucleotidase 1 | BPNT1 | AI439695 | Hs.271752 | chr1q41 |
| 232098_at | dystonin | DST | AK025142 | Hs.443518 | chr6p12-p11 |
| 232096_x_at | Forkhead box P1 | FOXP1 | BF841212 | Hs.518065 |  |
| 232087_at | chromosome X open reading frame 23 | CXorf23 | AW628045 | Hs.28896 | chrxp22.12 |
| 232080_at | HECT, C2 and WW domain containing E3 ubiquitin protein ligase 2 | HECW2 | AL390186 | Hs.210381 | chr2q32.3 |
| 232075_at | WD repeat domain 61 | WDR61 | BF791874 | Hs.125457 | chr15q25.1 |
| 232071_at | Mitochondrial ribosomal protein L19 | MRPL19 | BF670827 | Hs.158969 | chr2p11.2-p11.1 |
| 232066_x_at | hypothetical protein MGC21675 | MGC21675 | AL390154 | Hs.26954 | chr4p16.3 |
| 232063_x_at | phenylalanine-tRNA synthetase-like, beta subunit | FARSLB | AW024617 | Hs.9081 | chr2q36.1 |
| 232057_at | solute carrier family 7, member 6 opposite strand | SLC7A6OS | AK023353 | Hs.334848 | chr16q22.1 |
| 232053_x_at | rhomboid domain containing 2 | RHBDD2 | AL533352 | Hs.7788 | chr7q11.23 |
| 232038_at | chromosome 6 open reading frame 170 | C6orf170 | AI052103 | Hs.121396 | chr6q22.31 |
| 232035_at | histone cluster 1, H4h | HIST1H4H | BE740761 |  |  |
| 232034_at | hypothetical protein LOC203274 | LOC203274 | AL117607 | Hs.175563 | chr9q21.11 |
| 232028_at | Zinc finger protein 678 | ZNF678 | AK026475 | Hs.18978 |  |
| 232023_at | transmembrane protein 67 | TMEM67 | AL575584 | Hs.116240 | chr8q22.1 |
| 232021_at | glycosyltransferase 8 domain containing 3 | GLT8D3 | AI864273 | Hs.259347 | chr12q12 |
| 232020_at | SMAD specific E3 ubiquitin protein ligase 2 | SMURF2 | AU157259 | Hs.387385 | chr17q22-q23 |
| 232018_at | leukocyte receptor cluster (LRC) member 1 | LENG1 | AI701895 | Hs.467277 | chr19q13.4 |
| 232011_s_at | microtubule-associated protein 1 light chain 3 alpha | MAP1LC3A | AL118520 | Hs.408932 | chr20cen-q13 |
| 232007_at | 1-acylglycerol-3-phosphate O-acyltransferase 5 (lysophosphatidic acid acyltransferase, epsilon) | AGPAT5 | AU145356 | Hs.281895 | chr8p23.1 |
| 232004_at | heterogeneous nuclear ribonucleoprotein R | HNRPR | AK001846 |  |  |
| 232001_at | hypothetical gene supported by AY007155 | LOC439949 | AW193600 | Hs.46919 | chr10p15.1 |
| 231997_at | tubulin folding cofactor E-like | TBCEL | R69910 | Hs.414251 | chr11q23.3 |
| 231995_at | chromosome 9 open reading frame 82 | C9orf82 | AF131768 | Hs.178357 | chr9p21.2 |
| 231990_at | ubiquitin specific peptidase 15 | USP15 | AK023703 | Hs.339425 | chr12q14 |
| 231988_x_at | zinc finger protein 490 | ZNF490 | AB033024 | Hs.175475 | chr19p13.2 |
| 231975_s_at | mesoderm induction early response 1, family member 3 | MIER3 | AK002183 | Hs.419195 | chr5q11.2 |
| 231974_at | myeloid/lymphoid or mixed-lineage leukemia 2 | MLL2 | AI742164 | Hs.534396 | chr12q12-q14 |
| 231973_s_at | anaphase promoting complex subunit 1 | ANAPC1 | AK001223 | Hs.436527 | chr2q12.1 |
| 231970_at | Chromosome 14 open reading frame 118 | C14orf118 | AK025117 |  |  |
| 231959_at | chromosome 14 open reading frame 46 | C14orf46 | AK023979 | Hs.351381 | chr14q24.3 |
| 231955_s_at | 3-hydroxyisobutyrate dehydrogenase | HIBADH | AC007130 | Hs.284170 | chr7p15.2 |
| 231954_at | hypothetical protein DKFZP434I0714 | DKFZP434I0714 | AL137273 | Hs.142307 | chr4q31.23 |
| 231952_at | hypothetical protein LOC731450 | LOC731450 | AU157271 | Hs.7111 | chr18q23 |
| 231950_at | zinc finger protein 658 | ZNF658 | AW874634 | Hs.150751 | chr9p13.1 |
| 231948_s_at | ubiquitin-conjugating enzyme E2F (putative) | UBE2F | BG171548 | Hs.157804 | chr2q37.3 |
| 231944_at | ERO1-like beta (S. cerevisiae) | ERO1LB | AL045717 | Hs.424926 | chr1q42.2-q43 |
| 231936_at | homeobox C9 | HOXC9 | AK000445 | Hs.40408 | chr12q13.3 |
| 231918_s_at | G elongation factor, mitochondrial 2 | GFM2 | AK025314 | Hs.171055 | chr5q13 |
| 231902_at | Hypothetical protein LOC152485 | LOC152485 | R34396 | Hs.133916 | chr4q31.21-q31.22 |
| 231899_at | zinc finger CCCH-type containing 12C | ZC3H12C | AB051513 | Hs.376289 | chr11q22.3 |
| 231878_at | Major vault protein | MVP | AK026112 | Hs.80680 | chr16p13.1-p11.2 |
| 231876_at | tripartite motif-containing 56 | TRIM56 | AL512757 | Hs.415240 | chr7q22.1 |
| 231875_at | kinesin family member 21A | KIF21A | AF155117 | Hs.374201 | chr12q12 |
| 231874_at | family with sequence similarity 126, member B | FAM126B | AU155930 |  |  |
| 231871_at | G protein-coupled receptor 180 | GPR180 | AU160685 | Hs.159422 | chr13q32.1 |
| 231869_at | KIAA1586 | KIAA1586 | BF339845 | Hs.180663 | chr6p12.1 |
| 231864_at | zinc finger protein 33A | ZNF33A | D31763 |  |  |
| 231861_at | low density lipoprotein receptor-related protein 10 | LRP10 | AK025898 | Hs.28368 | chr14q11.2 |
| 231855_at | KIAA1524 | KIAA1524 | AB040957 |  |  |
| 231854_at | Phosphoinositide-3-kinase, catalytic, alpha polypeptide | PIK3CA | W63579 | Hs.120905 | chr3q26.3-q27 |
| 231853_at | tubulin, delta 1 | TUBD1 | AK022771 | Hs.270847 | chr17q23.2 |
| 231850_x_at | KIAA1712 | KIAA1712 | AB051499 | Hs.29798 | chr4q34 |
| 231847_at | ankyrin repeat domain 54 | ANKRD54 | Z97630 | Hs.135259 | chr22q13.1 |
| 231844_at | hypothetical protein MGC27345 | MGC27345 | AW072547 | Hs.511404 | chr7q32.2 |
| 231843_at | DEAD (Asp-Glu-Ala-Asp) box polypeptide 55 | DDX55 | AB046815 | Hs.286173 | chr12q24.31 |
| 231842_at | KIAA1462 | KIAA1462 | AL553774 | Hs.534551 | chr10p11.23 |
| 231837_at | ubiquitin specific peptidase 28 | USP28 | AB040948 | Hs.142856 | chr11q23 |
| 231836_at | GLI-Kruppel family member HKR1 | HKR1 | BC004513 | Hs.422323 | chr19q13.12 |
| 231835_at | chromosome 1 open reading frame 93 | C1orf93 | AL359575 | Hs.380483 | chr1p36.32 |
| 231832_at | UDP-N-acetyl-alpha-D-galactosamine:polypeptide N-acetylgalactosaminyltransferase 4 (GalNAc-T4) | GALNT4 | AI890347 | Hs.25130 | chr12q21.3-q22 |
| 231830_x_at | RAB11 family interacting protein 1 (class I) | RAB11FIP1 | BE544375 | Hs.96125 | chr8p11.22 |
| 231829_at | virus-induced signaling adapter | VISA | AB033097 | Hs.471993 | chr20p13 |
| 231824_at | La ribonucleoprotein domain family, member 2 | LARP2 | AK027164 | Hs.444068 | chr4q28.2 |
| 231823_s_at | SH3 and PX domains 2B | SH3PXD2B | BG054798 | Hs.26204 | chr5q35.2 |
| 231822_at | CTTNBP2 N-terminal like | CTTNBP2NL | AI921883 | Hs.374649 | chr1p13.2 |
| 231815_at | PHD finger protein 12 | PHF12 | AB040956 | Hs.135890 | chr17q11.2 |
| 231810_at | BRI3 binding protein | BRI3BP | BG106919 | Hs.131886 | chr12q24.31 |
| 231809_x_at | programmed cell death 7 | PDCD7 | AW953770 | Hs.143253 | chr15q22.31 |
| 231808_at | keratin associated protein 4-7 | KRTAP4-7 | AY007106 | Hs.380164 | chr17q12-q21 |
| 231806_s_at | serine/threonine kinase 36, fused homolog (Drosophila) | STK36 | AL133630 | Hs.26996 | chr2q35 |
| 231779_at | interleukin-1 receptor-associated kinase 2 | IRAK2 | AI246590 | Hs.424542 | chr3p25.3 |
| 231775_at | tumor necrosis factor receptor superfamily, member 10a | TNFRSF10A | W65310 | Hs.401745 | chr8p21 |
| 231773_at | angiopoietin-like 1 | ANGPTL1 | BF002046 | Hs.220745 | chr1q25.2 |
| 231769_at | F-box protein 6 | FBXO6 | AF129536 | Hs.284226 | chr1p36.23-p36.11 |
| 231764_at | chromatin accessibility complex 1 | CHRAC1 | AK023537 | Hs.279704 | chr8q24.3 |
| 231730_at | soluble liver antigen/liver pancreas antigen | SLA/LP | NM_016955 | Hs.253305 | chr4p15.2 |
| 231727_s_at | MIF4G domain containing | MIF4GD | NM_020679 | Hs.534550 | chr17q25.1 |
| 231726_at | protocadherin beta 14 | PCDHB14 | NM_018934 | Hs.283678 | chr5q31 |
| 231723_at | sorting nexin 12 | SNX12 | NM_013346 | Hs.260750 | chrxq13.1 |
| 231714_s_at | adaptor-related protein complex 4, beta 1 subunit | AP4B1 | NM_006594 | Hs.130858 | chr1p13.2 |
| 231698_at | hypothetical LOC647115 | FLJ36848 | AV661152 | Hs.417361 | chr2p14-p13 |
| 231697_s_at | Transmembrane protein 49 | TMEM49 | AV660825 |  |  |
| 231577_s_at | guanylate binding protein 1, interferon-inducible, 67kDa | GBP1 | AW014593 | Hs.62661 | chr1p22.2 |
| 231530_s_at | chromosome 11 open reading frame 1 | C11orf1 | BG150085 | Hs.17546 | chr11q13-q22 |
| 231437_at | Solute carrier family 35, member D2 | SLC35D2 | AA693722 |  |  |
| 231332_at | Antagonist of mitotic exit network 1 homolog (S. cerevisiae) | AMN1 | AW295037 |  |  |
| 231329_at | Inositol 1,4,5-triphosphate receptor, type 1 | ITPR1 | N21631 |  |  |
| 231325_at | Unc-5 homolog D (C. elegans) | UNC5D | AI802048 | Hs.238889 | chr8p12 |
| 231297_at | DOT1-like, histone H3 methyltransferase (S. cerevisiae) | DOT1L | AI479899 | Hs.289848 | chr19p13.3 |
| 231270_at | carbonic anhydrase XIII | CA13 | BF111998 | Hs.127189 | chr8q21.2 |
| 231269_at | activating signal cointegrator 1 complex subunit 3 | ASCC3 | AU153330 |  | chr6q16.1-q16.3 |
| 231252_at | hypothetical protein FLJ23861 | FLJ23861 | AI680874 | Hs.122934 | chr2q34 |
| 231238_at | Solute carrier family 35, member E4 | SLC35E4 | AW294793 |  |  |
| 231227_at | Wingless-type MMTV integration site family, member 5A | WNT5A | AI703321 |  |  |
| 231212_x_at | Tyrosylprotein sulfotransferase 1 | TPST1 | AA161476 |  |  |
| 231202_at | aldehyde dehydrogenase 1 family, member L2 | ALDH1L2 | AI654224 | Hs.321988 | chr12q23.3 |
| 231199_at | N-acetyltransferase 13 | NAT13 | AA701676 |  |  |
| 231192_at | Endothelial differentiation, lysophosphatidic acid G-protein-coupled receptor, 7 | EDG7 | AW274018 | Hs.99083 | chr1p22.3-p31.1 |
| 231188_at | zinc finger and SCAN domain containing 2 | ZSCAN2 | AW206602 | Hs.444502 | chr15q25.2 |
| 231175_at | chromosome 6 open reading frame 65 | C6orf65 | N48613 | Hs.47403 | chr6p12.1 |
| 231164_at | hypothetical gene supported by AK095200; BC042853 | LOC440331 | AI570450 | Hs.376281 | chr16p13.3 |
| 231111_at | Chromosome 1 open reading frame 112 | C1orf112 | AI122787 |  |  |
| 231108_at | Fusion (involved in t(12;16) in malignant liposarcoma) | FUS | H43040 |  |  |
| 231106_at | hypothetical protein LOC399761 /// centaurin, gamma-like family, member 6 /// centaurin, gamma-like family, member 7 | LOC399761 | AI684591 | Hs.314437 | chr10q11.22 |
| 231102_at | carnitine O-octanoyltransferase | CROT | BE674103 | Hs.125039 | chr7q21.1 |
| 231101_at | Protein phosphatase 2, regulatory subunit B (B56), epsilon isoform | PPP2R5E | AI689803 |  |  |
| 231094_s_at | methylenetetrahydrofolate dehydrogenase (NADP+ dependent) 1-like | MTHFD1L | AL035086 | Hs.44155 | chr6q25.1 |
| 231057_at | Myotubularin related protein 2 | MTMR2 | AU144266 | Hs.181326 | chr11q22 |
| 231050_at | HRAS-like suppressor family, member 5 | HRASLS5 | AW771586 |  |  |
| 231029_at | Coagulation factor V (proaccelerin, labile factor) | F5 | AI740541 |  |  |
| 231024_at | hypothetical locus LOC572558 | LOC572558 | H12280 |  |  |
| 231022_at | similar to Occludin | NAIP | AI139990 |  |  |
| 231015_at | Kruppel-like factor 15 | KLF15 | AW014734 | Hs.272215 | chr3q13-q21 |
| 231002_s_at | Rabaptin, RAB GTPase binding effector protein 1 | RABEP1 | AA778438 |  |  |
| 230999_at | Hypothetical gene supported by AK096370 | FLJ39051 | AI743903 |  |  |
| 230918_at | Galactokinase 2 | GALK2 | BE856598 |  |  |
| 230893_at | DnaJ homology subfamily A member 5 | DNAJA5 | AI223870 | Hs.131887 | chr5p13.2 |
| 230887_at | CDC14 cell division cycle 14 homolog B (S. cerevisiae) /// CDC14 cell division cycle 14 homolog C (S. cerevisiae) | CDC14B | AI921238 | Hs.22116 | chr9q22.33 |
| 230840_at | hypothetical gene supported by BC035379; BC042129 | LOC388588 | BE504634 | Hs.22047 | chr1p36.32 |
| 230837_at | similar to phosphodiesterase 4D interacting protein isoform 1 | LOC647500 | N67108 |  |  |
| 230799_at | hypothetical protein LOC150837 | LOC150837 | W72564 |  | chr2p21 |
| 230782_at | similar to Sorbitol dehydrogenase (L-iditol 2-dehydrogenase) | LOC653381 | AV699883 | Hs.293672 |  |
| 230779_at | trinucleotide repeat containing 6B | TNRC6B | BF594371 | Hs.442983 |  |
| 230777_s_at | PR domain containing 15 /// similar to PR domain containing 15 | PRDM15 | AL109788 | Hs.314683 | chr21q22.3 |
| 230769_at | DENN/MADD domain containing 2C | DENND2C | AI916261 | Hs.127350 | chr1p13.2-p13.1 |
| 230764_at | Chromosome 9 open reading frame 5 | C9orf5 | BF109998 | Hs.418097 | chr9q31 |
| 230747_s_at | Chromosome 18 open reading frame 17 | C18orf17 | AA406435 | Hs.441254 | chr18q11.2 |
| 230737_s_at | Hypothetical gene supported by BC014163 | LOC387647 | AW118878 |  |  |
| 230733_at | Myosin regulatory light chain MRCL3 | MRCL3 | H98113 |  |  |
| 230722_at | basonuclin 2 | BNC2 | AI377043 |  |  |
| 230721_at | chromosome 16 open reading frame 52 | C16orf52 | BF436957 | Hs.513424 | chr16p12.1 |
| 230720_at | ring finger protein 182 | RNF182 | AI884906 | Hs.111164 | chr6p23 |
| 230713_at | Zinc finger, CCHC domain containing 11 | ZCCHC11 | BF115786 |  |  |
| 230703_at | Chromosome 14 open reading frame 32 | C14orf32 | AA001543 |  |  |
| 230685_at | hypothetical protein LOC644873 | FLJ33630 | AI799695 | Hs.536891 |  |
| 230679_at | WD repeat domain 32 | WDR32 | AA976778 | Hs.86043 |  |
| 230669_at | Polymerase (DNA directed), lambda | POLL | W38444 | Hs.241548 | chr3q22-q23 |
| 230659_at | ER degradation enhancer, mannosidase alpha-like 1 | EDEM1 | AW139300 |  |  |
| 230657_at | Clock homolog (mouse) | CLOCK | AI423466 |  |  |
| 230653_at | hypothetical protein LOC728555 /// hypothetical protein LOC730391 | LOC728555 | AI469960 |  |  |
| 230651_at | THO complex 2 | THOC2 | AI018256 |  |  |
| 230634_x_at | hypothetical protein BC011824 | LOC113179 | AI820875 |  | chr19p13.3 |
| 230626_at | tetraspanin 12 | TSPAN12 | AI056699 | Hs.16529 | chr7q31.31 |
| 230620_at | Ubiquitin specific peptidase 27, X-linked | USP27X | BE550967 |  |  |
| 230619_at | aryl hydrocarbon receptor nuclear translocator | ARNT | AI768497 | Hs.131494 | chr1q21 |
| 230599_at | Ring finger protein 19 | RNF19 | AI681558 |  |  |
| 230563_at | RasGEF domain family, member 1A | RASGEF1A | BF446578 | Hs.125293 | chr10q11.21 |
| 230559_x_at | FYVE, RhoGEF and PH domain containing 4 | FGD4 | AI277617 | Hs.409311 | chr12p11.21 |
| 230556_at | IMP1 inner mitochondrial membrane peptidase-like (S. cerevisiae) | IMMP1L | AA977197 | Hs.355515 | chr11p13 |
| 230546_at | vasohibin 1 | VASH1 | AA142893 | Hs.155182 | chr14q24.3 |
| 230538_at | SHC (Src homology 2 domain containing) family, member 4 | SHC4 | AI027957 | Hs.207589 | chr15q21.1-q21.2 |
| 230533_at | protein kinase C binding protein 1 | PRKCBP1 | AF144233 | Hs.191990 | chr20q13.12 |
| 230532_at | chromosome X open reading frame 38 | CXorf38 | BF001685 | Hs.175652 | chrxp11.4 |
| 230508_at | Dickkopf homolog 3 (Xenopus laevis) | DKK3 | AL569601 |  |  |
| 230503_at | Sterile alpha motif domain containing 4A | SAMD4A | AA151917 |  |  |
| 230502_s_at | Hypothetical protein LOC149832 | LOC149832 | BE501966 |  |  |
| 230480_at | piwi-like 4 (Drosophila) | PIWIL4 | AI808955 | Hs.58248 | chr11q21 |
| 230465_at | heparan sulfate 2-O-sulfotransferase 1 | HS2ST1 | AI806674 | Hs.169939 | chr1p31.1-p22.1 |
| 230462_at | numb homolog (Drosophila) | NUMB | AU158026 | Hs.445301 | chr14q24.3 |
| 230458_at | solute carrier family 45, member 1 | SLC45A1 | AF118274 | Hs.128740 | chr1p36.1-p36.2 |
| 230440_at | zinc finger protein 469 | ZNF469 | AW015537 | Hs.54925 | chr16q24 |
| 230435_at | hypothetical LOC375190 | LOC375190 | BF108666 | Hs.368780 | chr2p23.3 |
| 230434_at | phosphatase, orphan 2 | PHOSPHO2 | AA769615 | Hs.125465 | chr2q31.1 |
| 230426_at | dihydrolipoamide dehydrogenase | DLD | BF212846 |  |  |
| 230424_at | chromosome 5 open reading frame 13 | C5orf13 | AU144860 | Hs.508741 | chr5q22.1 |
| 230421_at | similar to hypothetical protein 9630041N07 | DKFZp686E2433 | AI340241 | Hs.445740 | chr5q35.3 |
| 230388_s_at | hypothetical protein LOC644246 | LOC644246 | AI797017 | Hs.58104 | chr17q21.31 |
| 230387_at | ATPase, Ca++ transporting, type 2C, member 1 | ATP2C1 | AL038450 |  |  |
| 230376_at | Glucosaminyl (N-acetyl) transferase 3, mucin type | GCNT3 | AI339915 |  |  |
| 230370_x_at | serine/threonine/tyrosine interacting-like 1 | STYXL1 | AI244335 | Hs.11615 | chr7q11.23 |
| 230369_at | G protein-coupled receptor 161 | GPR161 | AI743151 | Hs.271809 | chr1q24.2 |
| 230364_at | choline phosphotransferase 1 | CHPT1 | BF940025 | Hs.225567 | chr12q |
| 230352_at | Phosphoribosyl pyrophosphate synthetase 2 | PRPS2 | AI392908 |  |  |
| 230351_at | hypothetical protein LOC283481 | LOC283481 | AW070248 |  | chr13q33.1 |
| 230336_at | Chromosome 2 open reading frame 27 | C2orf27 | AW665278 |  |  |
| 230333_at | Spermidine/spermine N1-acetyltransferase 1 | SAT1 | BE326919 |  |  |
| 230326_s_at | chromosome 11 open reading frame 73 | C11orf73 | AA782955 | Hs.283322 | chr11q14.2 |
| 230298_at | similar to metallo-beta-lactamase superfamily protein | LOC153364 | AI692906 | Hs.64004 | chr5q14.3 |
| 230285_at | hypothetical protein DKFZp313A2432 | DKFZp313A2432 | BF447829 | Hs.349096 | chr11p14.2 |
| 230270_at | PRP38 pre-mRNA processing factor 38 (yeast) domain containing B | PRPF38B | N32872 | Hs.342307 | chr1p13.3 |
| 230259_at | chromosome 10 open reading frame 125 | C10orf125 | AI471699 | Hs.155823 | chr10q26.3 |
| 230256_at | Farnesyl diphosphate synthase (farnesyl pyrophosphate synthetase, dimethylallyltranstransferase, geranyltranstransferase) | FDPS | AW009436 |  |  |
| 230241_at | Interferon responsive gene 15 | IFRG15 | BF196874 |  |  |
| 230226_s_at | jumonji, AT rich interactive domain 1A | JARID1A | AW612682 | Hs.76272 | chr12p11 |
| 230192_at | tripartite motif-containing 13 | TRIM13 | AI472310 | Hs.436922 | chr13q14 |
| 230185_at | THAP domain containing 9 | THAP9 | AW301218 | Hs.409270 | chr4q21.3 |
| 230179_at | hypothetical protein LOC285812 | LOC285812 | N52572 | Hs.435694 | chr6p23 |
| 230172_at | Family with sequence similarity 14, member B | FAM14B | AL039706 | Hs.19414 | chr14q32.13 |
| 230166_at | KIAA1958 | KIAA1958 | BG026236 | Hs.276754 | chr9q32 |
| 230165_at | shugoshin-like 2 (S. pombe) | SGOL2 | N31731 |  |  |
| 230164_at | zinc finger protein 621 | ZNF621 | AA417970 | Hs.19977 | chr3p22.1 |
| 230161_at | CD99 molecule | CD99 | BF513346 |  |  |
| 230151_at | chromosome 13 open reading frame 1 | C13orf1 | AW009330 | Hs.44235 | chr13q14 |
| 230146_s_at | frequenin homolog (Drosophila) | FREQ | BF111850 | Hs.301760 | chr9q34 |
| 230141_at | AT rich interactive domain 4A (RBP1-like) | ARID4A | AI640594 | Hs.19110 | chr14q23.1 |
| 230136_at | hypothetical gene supported by BC024195 | LOC400099 | AI573252 | Hs.400432 | chr13q12.11 |
| 230133_at | Membrane associated DNA binding protein | MNAB | BF972355 | Hs.497095 | chr9q34 |
| 230129_at | chromosome 10 open reading frame 89 | C10orf89 | BF589448 | Hs.281004 | chr10q26.13 |
| 230097_at | Phosphoribosylglycinamide formyltransferase, phosphoribosylglycinamide synthetase, phosphoribosylaminoimidazole synthetase | GART | AI207338 |  |  |
| 230081_at | phosphatidylinositol-specific phospholipase C, X domain containing 3 | PLCXD3 | AI694325 | Hs.145404 | chr5p13.1 |
| 230077_at | succinate dehydrogenase complex, subunit A, flavoprotein (Fp) /// succinate dehydrogenase complex, subunit A, flavoprotein pseudogene /// succinate dehydrogenase complex, subunit A, flavoprotein pseudogene 1 /// succinate dehydrogenase complex, subunit A, | SDHA | W90764 | Hs.440475 | chr5p15 |
| 230069_at | sideroflexin 1 | SFXN1 | BF593817 | Hs.135742 | chr5q35.3 |
| 230068_s_at | Paternally expressed 3 | PEG3 | W93046 |  |  |
| 230056_at | bromodomain PHD finger transcription factor | BPTF | AI735696 | Hs.440947 | chr17q24.3 |
| 230040_at | ADAM metallopeptidase with thrombospondin type 1 motif, 18 | ADAMTS18 | AI733120 | Hs.188746 | chr16q23 |
| 230036_at | sterile alpha motif domain containing 9-like | SAMD9L | BE669858 | Hs.319825 | chr7q21.3 |
| 230032_at | O-sialoglycoprotein endopeptidase-like 1 | OSGEPL1 | AW130077 | Hs.60772 | chr2q32.2 |
| 230031_at | heat shock 70kDa protein 5 (glucose-regulated protein, 78kDa) | HSPA5 | AW052044 | Hs.310769 | chr9q33-q34.1 |
| 230029_x_at | zinc finger protein 650 | ZNF650 | AI708334 | Hs.379548 | chr2q31.1 |
| 230027_s_at | mitochondrial ribosomal protein L43 | MRPL43 | N74662 | Hs.421848 | chr10q24.31 |
| 230009_at | family with sequence similarity 118, member B | FAM118B | AI936072 | Hs.276315 | chr11q24.2 |
| 229991_s_at | Synaptotagmin-like 4 (granuphilin-a) | SYTL4 | AI167292 | Hs.376981 |  |
| 229986_at | zinc finger protein 717 | ZNF717 | AW205616 | Hs.512536 | chr3p12.3 |
| 229983_at | tigger transposable element derived 2 | TIGD2 | AI610112 | Hs.58924 | chr4q22.1 |
| 229963_at | NGFRAP1-like 1 | NGFRAP1L1 | AV726956 | Hs.47209 | chrXq22.1 |
| 229958_at | chromosome 8 open reading frame 61 | C8orf61 | W93695 | Hs.127675 | chr8p23 |
| 229957_at | transmembrane protein 91 | TMEM91 | BF446281 | Hs.433307 | chr19q13.1-q13.2 |
| 229951_x_at | Hypothetical protein LOC728855 | LOC728855 | H24473 |  |  |
| 229949_at | DKFZp434A0131 protein | DKFZP434A0131 | AA554827 |  |  |
| 229946_at | hypothetical protein LOC130074 | LOC130074 | BF056651 | Hs.296356 | chr2q21.1 |
| 229933_at | chromosome 1 open reading frame 74 | C1orf74 | AW295407 | Hs.128893 | chr1q32.2 |
| 229910_at | Src homology 2 domain containing E | SHE | AI797276 | Hs.199749 | chr1q21.3 |
| 229893_at | FERM domain containing 3 | FRMD3 | BF589413 | Hs.55501 | chr9q21.32 |
| 229886_at | chromosome 5 open reading frame 34 | C5orf34 | AA502768 | Hs.448041 | chr5p12 |
| 229884_s_at | mitochondrial ribosomal protein L2 | MRPL2 | AA187325 | Hs.55041 | chr6p21.3 |
| 229881_at | Kruppel-like factor 12 | KLF12 | R41200 | Hs.164467 | chr13q22 |
| 229874_x_at | hypothetical protein LOC729604 /// hypothetical protein LOC731302 | LOC729604 | BE865517 | Hs.465405 | chr1p36.13 |
| 229873_at | potassium channel tetramerisation domain containing 21 | KCTD21 | AI391633 | Hs.440537 | chr11q14.1 |
| 229865_at | fibronectin type III domain containing 3B | FNDC3B | AW058617 | Hs.526785 |  |
| 229863_s_at | transmembrane protein 103 | TMEM103 | BE503382 | Hs.311100 | chr3p21.31 |
| 229862_x_at | zinc finger and BTB domain containing 45 | ZBTB45 | W63776 | Hs.445346 | chr19q13.43 |
| 229860_x_at | hypothetical gene supported by BC038466; BC062790 | LOC401115 | AI341602 | Hs.446474 | chr4p16.3 |
| 229852_at | nicotinamide nucleotide adenylyltransferase 1 | NMNAT1 | AW779092 | Hs.58251 | chr1p32-p35 |
| 229845_at | mitogen-activated protein kinase associated protein 1 | MAPKAP1 | BF939919 | Hs.324178 | chr9q33.3 |
| 229835_s_at | ATP synthase, H+ transporting, mitochondrial F1 complex, epsilon subunit | ATP5E | AA004210 |  |  |
| 229831_at | contactin 3 (plasmacytoma associated) | CNTN3 | BE221817 | Hs.529543 | chr3p26 |
| 229819_at | alpha-1-B glycoprotein | A1BG | AI022193 | Hs.390608 | chr19q13.4 |
| 229817_at | zinc finger protein 608 | ZNF608 | AI452715 | Hs.93738 | chr5q23.2 |
| 229797_at | mucolipin 3 | MCOLN3 | AI636080 | Hs.534528 | chr1p22.3 |
| 229793_at | N-acylsphingosine amidohydrolase (non-lysosomal ceramidase) 2B like | RP11-564C4.1 | AI656964 |  |  |
| 229785_at | KRIT1, ankyrin repeat containing | KRIT1 | BF939071 | Hs.438833 | chr7q21-q22 |
| 229784_at | Hypothetical protein MGC16121 | MGC16121 | AA234096 |  |  |
| 229781_at | Calmodulin binding transcription activator 1 /// Full-length cDNA clone CS0DI036YE11 of Placenta Cot 25-normalized of Homo sapiens (human) | CAMTA1 | AW005640 |  |  |
| 229779_at | collagen, type IV, alpha 4 | COL4A4 | BF476080 | Hs.418040 |  |
| 229771_at | Transmembrane protein 41A | TMEM41A | AI290653 | Hs.377067 |  |
| 229766_at | zinc finger protein 445 | ZNF445 | AW264273 | Hs.534732 | chr3p21.32 |
| 229765_at | Ras homolog gene family, member T1 | RHOT1 | AW511239 |  |  |
| 229758_at | tigger transposable element derived 5 | TIGD5 | AW168771 | Hs.71574 | chr8q24.3 |
| 229757_at | Hypothetical protein BC008207 | LOC92345 | AI741365 |  |  |
| 229744_at | Sperm specific antigen 2 | SSFA2 | AL556611 | Hs.438599 | chr2q31.3 |
| 229742_at | hypothetical LOC145853 | LOC145853 | AA420989 | Hs.97896 | chr15q23 |
| 229732_at | ZFP-36 for a zinc finger protein | HSZFP36 | AI417785 | Hs.142167 | chr19p13.2 |
| 229700_at | zinc finger protein 738 | ZNF738 | BE966267 | Hs.444206 | chr19p13.11 |
| 229697_at | HIRA interacting protein 3 | HIRIP3 | AI392830 | Hs.446619 | chr16p11.2 |
| 229695_at | Family with sequence similarity 107, member B | FAM107B | AU156189 | Hs.103378 | chr10p13 |
| 229693_at | similar to RIKEN cDNA A730055C05 gene | LOC388335 | AI952836 | Hs.332174 | chr17p13.1 |
| 229665_at | cleavage stimulation factor, 3' pre-RNA, subunit 3, 77kDa | CSTF3 | AI653037 | Hs.180034 | chr11p13 |
| 229663_at | lon peptidase 2, peroxisomal | LONP2 | AW574915 | Hs.118173 | chr16q12.1 |
| 229644_at | Prolyl endopeptidase | PREP | AA534420 |  |  |
| 229640_x_at | Hypothetical LOC646208 | LOC646208 | AW439242 | Hs.380803 | chr13q12.12 |
| 229638_at | iroquois homeobox protein 3 | IRX3 | AI681917 | Hs.3321 | chr16q12.2 |
| 229632_s_at | integrator complex subunit 10 | INTS10 | AA115512 | Hs.227074 | chr8p21.3 |
| 229626_at | similar to expressed sequence AI836003 | LOC387856 | AI971535 | Hs.251699 | chr12q13.11 |
| 229624_at | Optic atrophy 3 (autosomal recessive, with chorea and spastic paraplegia) | OPA3 | AI970518 | Hs.123473 | chr19q13.32 |
| 229623_at | Hypothetical LOC441027 | FLJ12993 | BF508344 |  |  |
| 229595_at | coiled-coil-helix-coiled-coil-helix domain containing 4 | CHCHD4 | AI493303 | Hs.349494 | chr3p25.1 |
| 229590_at | Ribosomal protein L13 | RPL13 | AI369389 | Hs.410817 | chr16q24.3 |
| 229588_at | DnaJ (Hsp40) homolog, subfamily C, member 10 | DNAJC10 | AA651899 | Hs.1098 | chr2q32.1 |
| 229587_at | SUMO1 activating enzyme subunit 2 | SAE2 | AA974493 | Hs.440661 | chr19q12 |
| 229573_at | ubiquitin specific peptidase 9, X-linked | USP9X | AI659456 |  |  |
| 229571_at | Calmodulin 2 (phosphorylase kinase, delta) | CALM2 | AV763524 |  |  |
| 229568_at | MOB1, Mps One Binder kinase activator-like 2B (yeast) | MOBKL2B | AI692878 | Hs.128905 | chr9p21.2 |
| 229558_at | Esophageal cancer associated protein | MGC16824 | AI927643 |  |  |
| 229549_at | Opsin 1 (cone pigments), short-wave-sensitive (color blindness, tritan) | OPN1SW | AA868461 |  |  |
| 229535_at | protein tyrosine phosphatase, mitochondrial 1 | PTPMT1 | BE856827 | Hs.440695 | chr11p11.2 |
| 229534_at | acyl-CoA thioesterase 4 | ACOT4 | AA046424 | Hs.49433 | chr14q24.3 |
| 229533_x_at | zinc finger protein 680 | ZNF680 | AI963028 | Hs.180546 | chr7q11.21 |
| 229532_at | zinc finger protein 502 | ZNF502 | AI056364 | Hs.224843 | chr3p21.31 |
| 229531_at | Mitochondrial carrier triple repeat 6 | MCART6 | AW182938 |  |  |
| 229528_at | hypothetical protein LOC283378 | LOC283378 | AI670935 |  | chr12q24.31 |
| 229526_at | aquaporin 11 | AQP11 | AI886656 | Hs.312529 | chr11q13.5 |
| 229523_at | two transmembrane domain family member A | TTMA | N66694 |  |  |
| 229521_at | hypothetical protein FLJ36031 | FLJ36031 | BE466274 | Hs.29692 | chr7q22.3 |
| 229517_at | protein tyrosine phosphatase domain containing 1 | PTPDC1 | BE046919 | Hs.437943 | chr9q22.32 |
| 229515_at | PRKC, apoptosis, WT1, regulator | PAWR | AA632147 | Hs.406074 | chr12q21 |
| 229513_at | Spermatid perinuclear RNA binding protein | STRBP | AK025613 | Hs.287659 | chr9q33.3 |
| 229511_at | SWI/SNF related, matrix associated, actin dependent regulator of chromatin, subfamily e, member 1 | SMARCE1 | AA195255 | Hs.437546 | chr17q21.2 |
| 229501_s_at | Ubiquitin specific peptidase 8 | USP8 | AI393759 |  |  |
| 229491_at | hypothetical protein BC009732 | LOC133308 | BF433180 | Hs.123183 | chr4q24 |
| 229483_at | Ubiquitin-conjugating enzyme E2H (UBC8 homolog, yeast) | UBE2H | AA760738 | Hs.372758 | chr7q32 |
| 229466_at | hypothetical protein LOC256273 | LOC256273 | AU144187 | Hs.99626 | chr11p15.3 |
| 229465_s_at | Protein tyrosine phosphatase, receptor type, S | PTPRS | BF433071 |  |  |
| 229459_at | family with sequence similarity 19 (chemokine (C-C motif)-like), member A5 | FAM19A5 | AV723914 | Hs.436854 | chr22q13.32 |
| 229434_at | Heterogeneous nuclear ribonucleoprotein D (AU-rich element RNA binding protein 1, 37kDa) | HNRPD | AA865357 |  |  |
| 229431_at | regulatory factor X-associated protein | RFXAP | AI742868 | Hs.24422 | chr13q14 |
| 229422_at | Nardilysin (N-arginine dibasic convertase) | NRD1 | AA448346 | Hs.136336 | chr1p32.2-p32.1 |
| 229418_at | chromosome 17 open reading frame 63 | C17orf63 | AV709958 |  |  |
| 229411_at | pregnancy upregulated non-ubiquitously expressed CaM kinase | PNCK | AI986390 | Hs.436667 | chrxq28 |
| 229410_at | Progestagen-associated endometrial protein (placental protein 14, pregnancy-associated endometrial alpha-2-globulin, alpha uterine protein) | PAEP | AI659219 | Hs.134074 | chr19p13.11 |
| 229407_at | sidekick homolog 1 (chicken) | SDK1 | AF131799 | Hs.128059 | chr7p22.2 |
| 229399_at | chromosome 10 open reading frame 118 | C10orf118 | BF438440 | Hs.159066 | chr10q25.3 |
| 229394_s_at | glucocorticoid receptor DNA binding factor 1 | GRLF1 | AI275597 | Hs.102548 | chr19q13.3 |
| 229393_at | l(3)mbt-like 3 (Drosophila) | L3MBTL3 | AA088346 | Hs.445813 | chr6q23 |
| 229377_at | growth hormone regulated TBC protein 1 | GRTP1 | BF001483 | Hs.108118 | chr13q34 |
| 229374_at | EPH receptor A4 | EPHA4 | AI758962 | Hs.244624 | chr2q36.1 |
| 229371_at | Solute carrier family 20 (phosphate transporter), member 2 | SLC20A2 | BF940010 | Hs.347527 | chr8p12-q21 |
| 229366_at | Cereblon | CRBN | BG149765 |  |  |
| 229362_at | Hypothetical protein FLJ32312 | FLJ32312 | AA878516 |  |  |
| 229360_at | suppressor of hairy wing homolog 2 (Drosophila) | SUHW2 | N22886 | Hs.43834 | chr22q11.2 |
| 229356_x_at | INO80 complex homolog 1 (S. cerevisiae) | INOC1 | AK002176 | Hs.409362 | chr15q15.1 |
| 229354_at | aryl-hydrocarbon receptor repressor | AHRR | AB033060 | Hs.24087 | chr5p15.3 /// chr5pter-p15.2 |
| 229344_x_at | family with sequence similarity 80, member B | FAM80B | AW135012 | Hs.372288 | chr12p13.31 |
| 229336_at | ST3 beta-galactoside alpha-2,3-sialyltransferase 2 /// hypothetical protein LOC729518 | ST3GAL2 | BE300666 |  |  |
| 229331_at | spermatogenesis associated 18 homolog (rat) | SPATA18 | AI559300 | Hs.335499 | chr4q11 |
| 229325_at | zinc finger, ZZ-type containing 3 | ZZZ3 | AW515772 | Hs.5364 | chr1p31.1 |
| 229285_at | ribonuclease L (2',5'-oligoisoadenylate synthetase-dependent) | RNASEL | AI669749 | Hs.404277 | chr1q25 |
| 229269_x_at | single stranded DNA binding protein 4 /// similar to single stranded DNA binding protein 4 isoform a /// similar to single stranded DNA binding protein 4 isoform a | SSBP4 | AA847828 | Hs.324618 | chr19p13.1 |
| 229264_at | hypothetical LOC642441 /// hypothetical protein LOC730256 /// hypothetical protein LOC730257 | LOC642441 | AI675152 | Hs.456577 | chr1q21.1 |
| 229257_at | KIAA1856 protein | KIAA1856 | AI625045 | Hs.383245 | chr7p22.1 |
| 229253_at | thioesterase superfamily member 4 | THEM4 | AI184512 | Hs.293691 | chr1q21 |
| 229240_at | Zinc finger, DHHC-type containing 21 | ZDHHC21 | AI658548 | Hs.21388 | chr9p22.3 |
| 229236_s_at | sideroflexin 4 | SFXN4 | AI346445 | Hs.534635 | chr10q26.11 |
| 229232_at | leucine rich repeat containing 57 | LRRC57 | AW135740 | Hs.437483 | chr15q15.1 |
| 229231_at | leucine rich repeat containing 37B | LRRC37B | H04996 | Hs.367593 | chr17q11.2 |
| 229224_x_at | hypothetical LOC643085 | LOC643085 | AW303605 |  |  |
| 229211_at | similar to RIKEN cDNA 0710001B24 | LOC285193 | AI672432 | Hs.369297 | chr2q37.3 |
| 229210_at | ribonuclease H2, subunit B | RNASEH2B | AI025023 | Hs.306291 | chr13q14.3 |
| 229208_at | centrosomal protein 27kDa | CEP27 | AK022939 |  |  |
| 229204_at | Heterochromatin protein 1, binding protein 3 | HP1BP3 | BE218428 |  |  |
| 229200_at | hypothetical protein LOC729810 | LOC729810 | N40199 |  |  |
| 229198_at | ubiquitin specific peptidase 35 | USP35 | AB037793 | Hs.128867 | chr11q14.1 |
| 229193_at | Cisplatin resistance-associated overexpressed protein | CROP | AA005430 | Hs.534420 |  |
| 229187_at | Zinc finger protein 542 /// Similar to FRG1 protein (FSHD region gene 1 protein) | ZNF542 | AI026708 | Hs.406330 /// Hs.450763 | chr12q14.2 /// chr19q13.43 |
| 229160_at | melanoma associated antigen (mutated) 1-like 1 | MUM1L1 | AI967987 | Hs.10653 | chrxq22.3 |
| 229159_at | Thrombospondin, type I, domain containing 7A | THSD7A | AW612111 |  |  |
| 229131_at | Exonuclease 3'-5' domain-like 2 | EXDL2 | AI702450 |  |  |
| 229130_at | hypothetical protein LOC285535 | LOC285535 | AU145323 | Hs.400256 | chr4p16.1 |
| 229126_at | transmembrane protein 19 | TMEM19 | AI597651 |  |  |
| 229119_s_at | actin, beta /// SWIM-domain containing Srs2 interacting protein 1 | ACTB | BG108034 | Hs.171044 | chr17p12 |
| 229113_s_at | chromosome 1 open reading frame 86 | C1orf86 | W16779 | Hs.525241 | chr1p36.33 |
| 229106_at | dynein, light chain, LC8-type 2 | DYNLL2 | AA401429 | Hs.356832 | chr17q23.2 |
| 229103_at | wingless-type MMTV integration site family, member 3 | WNT3 | AA463626 |  |  |
| 229101_at | Hypothetical protein LOC150166 | LOC150166 | AI963142 |  |  |
| 229100_s_at | receptor (G protein-coupled) activity modifying protein 2 /// translocase of inner mitochondrial membrane 22 homolog (yeast) | RAMP2 | H87708 | Hs.87595 | chr17p13 |
| 229099_at | hypothetical protein LOC790955 | LOC790955 | AW051379 | Hs.473437 |  |
| 229097_at | diaphanous homolog 3 (Drosophila) | DIAPH3 | AI813331 |  |  |
| 229092_at | Nuclear receptor subfamily 2, group F, member 2 | NR2F2 | AI420144 | Hs.91954 |  |
| 229083_at | Heterogeneous nuclear ribonucleoprotein A0 | HNRPA0 | AI672356 |  |  |
| 229076_s_at | Translocase of outer mitochondrial membrane 22 homolog (yeast) | TOMM22 | AI808192 |  |  |
| 229071_at | hypothetical gene supported by BC052606 | LOC388327 | AI681419 | Hs.262480 | chr17p13.1 |
| 229069_at | cytokine induced protein 29 kDa | CIP29 | BF477573 | Hs.410597 | chr12q13.2 |
| 229068_at | chaperonin containing TCP1, subunit 5 (epsilon) | CCT5 | BF197357 |  |  |
| 229063_s_at | coiled-coil domain containing 107 | CCDC107 | AI912238 | Hs.245342 | chr9p13.3 |
| 229061_s_at | solute carrier family 25, member 13 (citrin) | SLC25A13 | AW268880 | Hs.9599 | chr7q21.3 |
| 229050_s_at | small nucleolar RNA host gene (non-protein coding) 7 | SNHG7 | AL533103 | Hs.536090 | chr9q34.3 |
| 229027_at | Protein phosphatase 1A (formerly 2C), magnesium-dependent, alpha isoform | PPM1A | AI241945 |  |  |
| 229010_at | Cas-Br-M (murine) ecotropic retroviral transforming sequence | CBL | AI807026 | Hs.41324 | chr11q23.3 |
| 229007_at | hypothetical protein LOC283788 | LOC283788 | N50864 |  | chr15 |
| 228996_at | ring finger and CCCH-type zinc finger domains 1 | RC3H1 | AW205418 | Hs.154133 | chr1q25.1 |
| 228988_at | zinc finger protein 711 | ZNF711 | AU157017 |  |  |
| 228986_at | oxysterol binding protein-like 8 | OSBPL8 | AW978375 | Hs.109694 | chr12q14 |
| 228980_at | ring finger and FYVE-like domain containing 1 | RFFL | AI760772 |  |  |
| 228972_at | Neural precursor cell expressed, developmentally down-regulated 1 | NEDD1 | AI028602 |  |  |
| 228967_at | Eukaryotic translation initiation factor 1 | EIF1 | BE964053 |  |  |
| 228964_at | PR domain containing 1, with ZNF domain | PRDM1 | AI692659 | Hs.381140 | chr6q21-q22.1 |
| 228954_at | LysM, putative peptidoglycan-binding, domain containing 4 | LYSMD4 | AW452620 | Hs.283107 | chr15q26.3 |
| 228950_s_at | G protein-coupled receptor 177 | GPR177 | AL534095 | Hs.297792 | chr1p31.2 |
| 228944_at | Aristaless-like homeobox 3 | ALX3 | AI076018 |  |  |
| 228933_at | Nance-Horan syndrome (congenital cataracts and dental anomalies) | NHS | BF111819 | Hs.21470 | chrxp22.13 |
| 228931_at | coenzyme Q4 homolog (S. cerevisiae) | COQ4 | AW628685 | Hs.534509 | chr9q34.11 |
| 228930_at | Small Cajal body-specific RNA 15 | SCARNA15 | AI679611 | Hs.374400 | chr15q25.2 |
| 228928_x_at | BTG3 associated nuclear protein | BANP | AL157429 | Hs.448828 | chr16q24 |
| 228927_at | zinc finger protein 397 | ZNF397 | AW291411 | Hs.125353 | chr18q12.2 |
| 228925_at | Transmembrane protein 116 | TMEM116 | AW195586 |  |  |
| 228922_at | Src homology 2 domain containing F | SHF | AI760446 | Hs.310399 | chr15q21.1 |
| 228920_at | zinc finger protein 260 | ZNF260 | BE328273 | Hs.18103 | chr19q13.12 |
| 228909_at | Chromosome 21 open reading frame 86 | C21orf86 | AW131553 | Hs.11637 | chr21q22.3 |
| 228899_at | Cullin 1 | CUL1 | AI870903 | Hs.535181 | chr7q36.1 |
| 228885_at | MAM domain containing 2 | MAMDC2 | AI862120 | Hs.130341 | chr9q21.11 |
| 228882_at | tubby homolog (mouse) | TUB | AL042088 | Hs.54468 | chr11p15.5 |
| 228879_at | small nucleolar RNA, H/ACA box 76 | SNORA76 | BG502554 |  |  |
| 228867_at | TatD DNase domain containing 3 | TATDN3 | BE541548 | Hs.374419 | chr1q32.3 |
| 228863_at | protocadherin 17 | PCDH17 | N69091 |  |  |
| 228855_at | similar to Peroxisomal coenzyme A diphosphatase NUDT7 (Nucleoside diphosphate-linked moiety X motif 7) (Nudix motif 7) /// similar to Peroxisomal coenzyme A diphosphatase NUDT7 (Nucleoside diphosphate-linked moiety X motif 7) (Nudix motif 7) | LOC729777 | AI927964 | Hs.115920 | chr16q23.1 |
| 228853_at | similar to serine/threonine/tyrosine interacting protein | LOC730432 | AI652546 |  |  |
| 228842_at | Hypothetical LOC646509 | LOC646509 | AK025716 | Hs.444182 | chr2q21 |
| 228840_at | angiomotin like 1 | AMOTL1 | AW451115 | Hs.292781 | chr11q14.3 |
| 228839_s_at | hypothetical gene supported by AF064843; AK025716 /// hypothetical LOC642361 | LOC439994 | AA810864 | Hs.434988 /// Hs.444182 | chr10q23.2 /// chr2q21 |
| 228834_at | transducer of ERBB2, 1 | TOB1 | BF240286 | Hs.178137 | chr17q21 |
| 228832_at | hypothetical LOC90024 | FLJ20021 | AI884670 | Hs.356100 | chr4q24 |
| 228831_s_at | guanine nucleotide binding protein (G protein), gamma 7 | GNG7 | AL039870 | Hs.6750 | chr19p13.3 |
| 228830_s_at | activating transcription factor 7 | ATF7 | AI279868 | Hs.405395 | chr12q13 |
| 228810_at | hypothetical protein FLJ40432 | FLJ40432 | AW135279 | Hs.4190 | chr2q33.3 |
| 228809_at | chromosome X open reading frame 40A | CXorf40A | AW006998 | Hs.534613 | chrxq28 |
| 228805_at | chromosome 5 open reading frame 25 | C5orf25 | BF116060 | Hs.348516 | chr5q35.2 |
| 228802_at | RNA binding protein with multiple splicing 2 | RBPMS2 | BE348466 | Hs.436518 | chr15q22.31 |
| 228801_at | ORM1-like 1 (S. cerevisiae) | ORMDL1 | AI809749 | Hs.512600 | chr2q32.3 |
| 228791_at | Chromosome 15 open reading frame 38 | C15orf38 | BF434655 | Hs.6734 | chr15q26.1 |
| 228783_at | blood vessel epicardial substance | BVES | AA993518 |  |  |
| 228778_at | Microcephaly, primary autosomal recessive 1 | MCPH1 | BE673677 |  |  |
| 228777_at | kelch repeat and BTB (POZ) domain containing 3 | KBTBD3 | AI436268 | Hs.101949 | chr11q22.3 |
| 228776_at | gap junction protein, alpha 7, 45kDa (connexin 45) | GJA7 | AA430014 | Hs.438749 | chr17q21.31 |
| 228774_at | centrosomal protein 78kDa | CEP78 | AW268594 | Hs.374421 | chr9q21.2 |
| 228772_at | histamine N-methyltransferase | HNMT | AU157303 |  |  |
| 228769_at | zinc finger and SCAN domain containing 22 | ZSCAN22 | BE794668 | Hs.388162 | chr19q13.43 |
| 228765_at | GTF2I repeat domain containing 2 | GTF2IRD2 | BG032651 | Hs.352142 | chr7q11.23 |
| 228764_s_at | chromatin modifying protein 4A /// magnesium-dependent phosphatase 1 | CHMP4A | AI813313 | Hs.220963 | chr14q11.2 |
| 228758_at | B-cell CLL/lymphoma 6 (zinc finger protein 51) | BCL6 | AW264036 | Hs.188032 | chr3q27.3 |
| 228754_at | solute carrier family 6 (neurotransmitter transporter, taurine), member 6 | SLC6A6 | BG150485 |  |  |
| 228752_at | chromosome X open reading frame 10 | CXorf10 | N57931 | Hs.288512 | chrxq26 |
| 228751_at | CDC-like kinase 4 | CLK4 | AW975057 | Hs.406557 | chr5q35 |
| 228749_at | KIAA1571 protein | KIAA1571 | AV734793 | Hs.28838 | chr2q33.3 |
| 228748_at | CD59 molecule, complement regulatory protein | CD59 | AI653117 | Hs.278573 | chr11p13 |
| 228747_at | Sec61 alpha 2 subunit (S. cerevisiae) | SEC61A2 | AI694646 | Hs.534527 | chr10p14 |
| 228745_at | small glutamine-rich tetratricopeptide repeat (TPR)-containing, beta | SGTB | AI376997 | Hs.184664 | chr5q12.3 |
| 228744_at | Hypothetical protein LOC146481 | LOC146481 | AW611729 |  |  |
| 228737_at | chromosome 20 open reading frame 100 | C20orf100 | AA211909 | Hs.26608 | chr20q13.12 |
| 228736_at | DNA helicase HEL308 | HEL308 | AW084661 | Hs.194109 | chr4q21.23 |
| 228730_s_at | secernin 2 | SCRN2 | AW006952 | Hs.239718 | chr17q21.32 |
| 228722_at | protein arginine methyltransferase 2 | PRMT2 | AI928367 | Hs.154163 | chr21q22.3 |
| 228721_at | chromosome 3 open reading frame 41 | C3orf41 | AA225165 | Hs.146346 | chr3p22.1 |
| 228718_at | zinc finger protein 44 | ZNF44 | AI379070 |  | chr19p13.2 |
| 228711_at | zinc finger protein 37A | ZNF37A | BF059259 | Hs.446382 | chr10p11.2 |
| 228680_at | kinesin family member 3A | KIF3A | AW340096 |  |  |
| 228670_at | telomerase-associated protein 1 | TEP1 | BF197089 | Hs.132743 | chr14q11.2 |
| 228660_x_at | sema domain, immunoglobulin domain (Ig), transmembrane domain (TM) and short cytoplasmic domain, (semaphorin) 4F | SEMA4F | AA523537 | Hs.25887 | chr2p13.1 |
| 228656_at | Prospero-related homeobox 1 | PROX1 | AK025453 | Hs.159437 | chr1q32.2-q32.3 |
| 228654_at | hypothetical protein LOC139886 | RP11-93B10.1 | AU145277 | Hs.111496 | chrxq11.2 |
| 228652_at | zinc finger protein 776 | ZNF776 | AI279532 | Hs.109540 | chr19q13.43 |
| 228647_at | Ninjurin 2 | NINJ2 | AI675844 | Hs.535512 |  |
| 228646_at | protein phosphatase 1, regulatory (inhibitor) subunit 1C | PPP1R1C | AI806944 | Hs.10941 | chr2q31.3-q32.1 |
| 228641_at | caspase recruitment domain family, member 8 | CARD8 | BF338389 | Hs.446146 | chr19q13.32 |
| 228638_at | Family with sequence similarity 76, member A | FAM76A | AI631072 |  |  |
| 228635_at | protocadherin 10 | PCDH10 | AI640307 | Hs.146858 | chr4q28.3 |
| 228633_s_at | centrobin, centrosomal BRCA2 interacting protein | CNTROB | AI668799 | Hs.348012 | chr17p13.1 |
| 228630_at | Zinc finger protein 84 | ZNF84 | W92744 | Hs.22664 | chr12q24.33 |
| 228625_at | Cbp/p300-interacting transactivator, with Glu/Asp-rich carboxy-terminal domain, 4 | CITED4 | AI858001 | Hs.355820 | chr1p34.2 |
| 228622_s_at | DnaJ (Hsp40) homolog, subfamily C, member 4 | DNAJC4 | AW071239 | Hs.172847 | chr11q13 |
| 228619_x_at | TIP41, TOR signalling pathway regulator-like (S. cerevisiae) | TIPRL | AI694231 | Hs.209431 | chr1q23.2 |
| 228615_at | hypothetical protein LOC286161 | LOC286161 | AW291761 |  | chr8p23.3 |
| 228614_at | LOC205251 | LOC205251 | AW182614 | Hs.128499 | chr2q13 |
| 228613_at | RAB11 family interacting protein 3 (class II) | RAB11FIP3 | BF183535 | Hs.119004 | chr16p13.3 |
| 228606_at | hypothetical protein MGC33212 | MGC33212 | AW016718 |  | chr3q29 |
| 228603_at | Hypothetical LOC440900 | LOC440900 | AA977481 |  |  |
| 228602_at | sarcoglycan, delta (35kDa dystrophin-associated glycoprotein) | SGCD | AA479286 | Hs.131380 | chr5q33-q34 |
| 228600_x_at | chromosome 7 open reading frame 46 | C7orf46 | BE220330 | Hs.128757 | chr7p15.3 |
| 228598_at | dipeptidyl-peptidase 10 | DPP10 | AL538781 | Hs.176247 | chr2q14.1 |
| 228597_at | chromosome 21 open reading frame 45 | C21orf45 | AW151538 | Hs.49932 | chr21q22.11 |
| 228596_at | similar to rho guanine nucleotide exchange factor 5 | LOC728377 | AW009638 | Hs.334 | chr7q33-q35 |
| 228595_at | hydroxysteroid (17-beta) dehydrogenase 1 | HSD17B1 | AA894611 | Hs.448861 | chr17q11-q21 |
| 228587_at | Family with sequence similarity 83, member G | FAM83G | BE504892 |  |  |
| 228584_at | sarcoglycan, beta (43kDa dystrophin-associated glycoprotein) | SGCB | AI089025 | Hs.438953 | chr4q12 |
| 228577_x_at | outer dense fiber of sperm tails 2-like | ODF2L | AI338465 | Hs.435537 | chr1p22.3 |
| 228571_at | RB-associated KRAB zinc finger | RBAK | BE963438 |  |  |
| 228568_at | GRINL1A combined protein | Gcom1 | AI926697 | Hs.50841 | chr15q21.3 |
| 228565_at | mixed lineage kinase 4 | KIAA1804 | AI809005 | Hs.50883 | chr1q42 |
| 228561_at | cell division cycle 37 homolog (S. cerevisiae)-like 1 | CDC37L1 | BF510563 |  |  |
| 228557_at | L(3)mbt-like 4 (Drosophila) | L3MBTL4 | AK026733 | Hs.95111 |  |
| 228555_at | Calcium/calmodulin-dependent protein kinase (CaM kinase) II delta | CAMK2D | AA029441 | Hs.111460 | chr4q26 |
| 228549_at | Pleckstrin homology domain containing, family G (with RhoGef domain) member 4 /// Leucine rich repeat containing 36 /// Hydroxysteroid (11-beta) dehydrogenase 2 | PLEKHG4 | AI491983 |  |  |
| 228544_s_at | CSRP2 binding protein | CSRP2BP | AA780252 | Hs.210584 | chr20p11.23 |
| 228542_at | MRS2-like, magnesium homeostasis factor (S. cerevisiae) | MRS2L | AI631209 | Hs.146030 | chr6p22.3-p22.1 |
| 228541_x_at | hypothetical gene supported by AK098314 | LOC400446 | AI049608 | Hs.534262 | chr15q26.1 |
| 228538_at | zinc finger protein 662 | ZNF662 | BE671164 | Hs.293388 | chr3p22.1 |
| 228531_at | sterile alpha motif domain containing 9 | SAMD9 | AA741307 | Hs.65641 | chr7q21.2 |
| 228523_at | nanos homolog 1 (Drosophila) | NANOS1 | AW970089 | Hs.351851 | chr10q26.11 |
| 228521_s_at | RAB4B, member RAS oncogene family | RAB4B | AW242973 | Hs.25367 | chr19q13.2 |
| 228517_at | chromosome 1 open reading frame 149 | C1orf149 | AW466905 | Hs.17118 | chr1p35.3-p33 |
| 228516_at | congenital dyserythropoietic anemia, type I | CDAN1 | AI122852 | Hs.437819 | chr15q15.2 |
| 228515_at | hypothetical protein LOC90784 | LOC90784 | AU149280 |  | chr2p11.2 |
| 228505_s_at | transmembrane protein 170 | TMEM170 | N49836 | Hs.487510 | chr16q23.1 |
| 228500_at | THAP domain containing 8 | THAP8 | AW411259 | Hs.350209 | chr19q13.12 |
| 228495_at | Coiled-coil domain containing 75 | CCDC75 | AI880633 | Hs.335599 | chr2p22.2 |
| 228485_s_at | solute carrier family 44, member 1 | SLC44A1 | AW165999 | Hs.414728 | chr9q31.2 |
| 228482_at | CMT1A duplicated region transcript 4 | CDRT4 | AV702789 | Hs.164595 | chr17p12 |
| 228480_at | VAMP (vesicle-associated membrane protein)-associated protein A, 33kDa | VAPA | AW296039 | Hs.165195 | chr18p11.22 |
| 228477_at | Hypothetical protein FLJ10154 | FLJ10154 | R53084 |  |  |
| 228471_at | Nei endonuclease VIII-like 1 (E. coli) /// Ankyrin repeat domain 44 | NEIL1 | AA744636 | Hs.11571 | chr2q33.1 |
| 228469_at | Peptidylprolyl isomerase D (cyclophilin D) | PPID | BF431902 |  |  |
| 228468_at | microtubule associated serine/threonine kinase-like | MASTL | BF108964 | Hs.276905 | chr10p12.1 |
| 228458_at | similar to RIKEN cDNA 2310039H08 | LOC441150 | AI636501 | Hs.133864 | chr6p21.1 |
| 228452_at | chromosome 17 open reading frame 39 | C17orf39 | AA827865 | Hs.128339 | chr17p11.2 |
| 228445_at | apoptosis-inducing factor, mitochondrion-associated, 2 | AIFM2 | AI922797 | Hs.117062 | chr10q22.1 |
| 228436_at | potassium voltage-gated channel, Shaw-related subfamily, member 4 | KCNC4 | BF061003 | Hs.124212 | chr1p21 |
| 228420_at | programmed cell death 2 | PDCD2 | AW590850 | Hs.367900 | chr6q27 |
| 228412_at | hypothetical LOC643072 | LOC643072 | AI991451 |  |  |
| 228411_at | par-3 partitioning defective 3 homolog B (C. elegans) | PARD3B | BF109252 | Hs.271903 | chr2q33.3 |
| 228402_at | zinc finger, BED-type containing 3 | ZBED3 | AI679968 | Hs.355933 | chr5q13.3 |
| 228400_at | shroom family member 3 | SHROOM3 | AW025141 |  |  |
| 228398_at | SUMO/sentrin specific peptidase family member 8 | SENP8 | AW173305 | Hs.111991 | chr15q23 |
| 228392_at | zinc finger protein 302 | ZNF302 | BF508739 | Hs.436350 | chr19q13.11 |
| 228389_at | SRB7 suppressor of RNA polymerase B homolog (yeast) | SURB7 | BF197118 |  |  |
| 228385_at | DEAD (Asp-Glu-Ala-Asp) box polypeptide 59 | DDX59 | AI831738 | Hs.95321 | chr1q32.1 |
| 228379_at | Nuclear transport factor 2 | NUTF2 | AW071847 | Hs.356630 | chr16q22.1 |
| 228378_at | chromosome 12 open reading frame 29 | C12orf29 | BF439204 | Hs.535026 | chr12q21.33 |
| 228375_at | immunoglobulin superfamily, member 11 | IGSF11 | BE221674 | Hs.6658 | chr3q13.32 |
| 228367_at | alpha-kinase 2 | ALPK2 | BE551416 | Hs.388674 | chr18q21.31 |
| 228365_at | copine VIII | CPNE8 | AI765180 | Hs.71818 | chr12q12 |
| 228357_at | zinc finger CCCH-type containing 5 | ZC3H5 | BE966979 | Hs.133872 | chr17q25.1 |
| 228355_s_at | NDUFA12-like | NDUFA12L | AI110585 | Hs.180848 | chr5q12.1 |
| 228351_at | HEAT repeat containing 1 | HEATR1 | AA149823 | Hs.257300 | chr1q43 |
| 228346_at | Hypothetical protein FLJ14959 | FLJ14959 | H47630 |  |  |
| 228340_at | transducin-like enhancer of split 3 (E(sp1) homolog, Drosophila) | TLE3 | BE967118 |  |  |
| 228338_at | hypothetical protein LOC120376 | LOC120376 | AA812993 | Hs.31409 | chr11q23.1 |
| 228337_at | MSTP101 | MST101 | BE552097 | Hs.438789 | chr5q33.3 |
| 228335_at | claudin 11 (oligodendrocyte transmembrane protein) | CLDN11 | AW264204 | Hs.31595 | chr3q26.2-q26.3 |
| 228330_at | chromosome 6 open reading frame 113 | C6orf113 | AI927382 | Hs.29857 | chr6q22.1 |
| 228325_at | KIAA0146 | KIAA0146 | AI363213 | Hs.381058 | chr8q11.21 |
| 228324_at | chromosome 9 open reading frame 41 | C9orf41 | BF031819 | Hs.257556 | chr9q21.13 |
| 228306_at | Cornichon homolog 4 (Drosophila) | CNIH4 | T87074 | Hs.445890 | chr1q42.12 |
| 228305_at | zinc finger protein 565 | ZNF565 | BE466825 | Hs.20369 | chr19q13.12 |
| 228301_x_at | NADH dehydrogenase (ubiquinone) 1 beta subcomplex, 10, 22kDa | NDUFB10 | AA854479 | Hs.198274 | chr16p13.3 |
| 228298_at | family with sequence similarity 113, member B | FAM113B | BF056901 | Hs.258494 | chr12q13.11 |
| 228291_s_at | chromosome 20 open reading frame 19 | C20orf19 | AI806322 | Hs.187635 | chr20pter-q11.23 |
| 228289_at | Bromodomain containing 7 | BRD7 | AI131537 |  |  |
| 228287_at | inhibitor of growth family, member 5 /// similar to inhibitor of growth family, member 5 | ING5 | BG054893 | Hs.390538 | chr2q37.3 |
| 228286_at | hypothetical protein FLJ40869 | FLJ40869 | AK025489 | Hs.502848 | chr2p24.2 |
| 228285_at | tudor domain containing 9 | TDRD9 | AI989706 | Hs.21454 | chr14q32.33 |
| 228283_at | hypothetical protein MGC61571 | MGC61571 | BE963026 | Hs.534675 | chr3p24.1 |
| 228282_at | Hypothetical protein MGC33302 | MGC33302 | AW611550 | Hs.406037 | chr4q28.1-q28.2 |
| 228280_at | zinc finger CCCH-type, antiviral 1-like | ZC3HAV1L | AI188445 | Hs.152618 | chr7q34 |
| 228266_s_at | hepatoma-derived growth factor, related protein 3 | HDGFRP3 | BE703418 | Hs.127842 | chr15q11.2 |
| 228256_s_at | erythrocyte membrane protein band 4.1 like 4A | EPB41L4A | AU144565 | Hs.12082 | chr5q21-q22 |
| 228249_at | chromosome 11 open reading frame 74 | C11orf74 | AA535128 | Hs.406726 | chr11p12 |
| 228245_s_at | helicase with zinc finger /// ovostatin 2 /// similar to cDNA sequence BC048546 | HELZ | AW594320 | Hs.512260 | chr12p13 |
| 228238_at | growth arrest-specific 5 | GAS5 | AW105301 | Hs.19340 | chr1q23.3 |
| 228234_at | toll-like receptor adaptor molecule 2 | TICAM2 | AI423165 | Hs.534007 | chr5q23.1 |
| 228228_at | dapper, antagonist of beta-catenin, homolog 3 (Xenopus laevis) | DACT3 | AI809234 | Hs.134185 | chr19q13.32 |
| 228221_at | solute carrier family 44, member 3 | SLC44A3 | AA809640 | Hs.414416 | chr1p21.3 |
| 228220_at | FCH domain only 2 | FCHO2 | AI627666 | Hs.356358 | chr5q13.2 |
| 228217_s_at | hypothetical LOC389362 | RP11-506K6.3 | BF973374 | Hs.431456 | chr6p25.2 |
| 228211_at | chromosome 9 open reading frame 102 | C9orf102 | AI693726 | Hs.432364 | chr9q22.32 |
| 228208_x_at | Hypothetical LOC645944 | LOC645944 | AL134573 |  |  |
| 228204_at | Proteasome (prosome, macropain) subunit, beta type, 4 | PSMB4 | AA630330 |  |  |
| 228201_at | ADP-ribosylation factor-like 13B | ARL13B | BE858032 | Hs.369885 | chr3q11.2 |
| 228196_s_at | La ribonucleoprotein domain family, member 5 | LARP5 | BE974210 |  |  |
| 228195_at | hypothetical protein MGC13057 | MGC13057 | BE645119 | Hs.389311 | chr2q32.2 |
| 228190_at | Ctr9, Paf1/RNA polymerase II complex component, homolog (S. cerevisiae) | CTR9 | BF197009 |  |  |
| 228189_at | BCL2-associated athanogene 4 | BAG4 | T32429 |  |  |
| 228183_s_at | RPA interacting protein | RPAIN | BF342428 | Hs.355584 | chr17p13.2 |
| 228181_at | solute carrier family 30 (zinc transporter), member 1 | SLC30A1 | AI553933 | Hs.55610 | chr1q32-q41 |
| 228176_at | endothelial differentiation, sphingolipid G-protein-coupled receptor, 3 | EDG3 | AA534817 |  | chr9q22.1-q22.2 |
| 228174_at | Golgi autoantigen, golgin subfamily a, 1 | GOLGA1 | AI832363 |  |  |
| 228168_at | ATP synthase, H+ transporting, mitochondrial F0 complex, subunit C3 (subunit 9) | ATP5G3 | AU153583 |  |  |
| 228167_at | kelch-like 6 (Drosophila) | KLHL6 | AW574798 | Hs.128637 | chr3q27.3 |
| 228164_at | adaptor-related protein complex 4, epsilon 1 subunit | AP4E1 | BE964704 | Hs.413366 | chr15q21.2 |
| 228158_at | similar to lymphocyte-specific protein 1 isoform 1 | LOC645166 | AI623211 | Hs.488939 | chr1q21.1 |
| 228152_s_at | hypothetical protein FLJ31033 | FLJ31033 | AK023743 | Hs.164299 | chr4q32.3 |
| 228149_at | hypothetical protein FLJ31818 | FLJ31818 | BF432926 |  |  |
| 228145_s_at | zinc finger protein 398 | ZNF398 | AI950078 | Hs.169452 | chr7q36.1 |
| 228144_at | zinc finger protein 300 | ZNF300 | N49841 | Hs.134885 | chr5q33.1 |
| 228142_at | ubiquinol-cytochrome c reductase complex (7.2 kD) | UCRC | BE208777 | Hs.284292 | chr22cen-q12.3 |
| 228141_at | Similar to RIKEN cDNA 2310016C16 | LOC493869 | AA173223 | Hs.289044 |  |
| 228135_at | chromosome 1 open reading frame 52 | C1orf52 | AA738437 | Hs.26226 | chr1p22.3 |
| 228123_s_at | abhydrolase domain containing 12 | ABHD12 | AW303865 | Hs.441550 | chr20p11.21 |
| 228122_at | coiled-coil domain containing 66 | CCDC66 | AW572928 | Hs.534731 | chr3p14.3 |
| 228121_at | Transforming growth factor, beta 2 | TGFB2 | AU145950 | Hs.169300 | chr1q41 |
| 228099_at | zinc finger protein 550 | ZNF550 | AI805301 | Hs.180257 | chr19q13.43 |
| 228089_x_at | similar to RIKEN cDNA 1810059G22 | LOC374395 | H72927 | Hs.381134 | chr11q12.3 |
| 228087_at | coiled-coil domain containing 126 | CCDC126 | AK026684 | Hs.510548 | chr7p15.3 |
| 228082_at | adipocyte-specific adhesion molecule | ASAM | BF056275 | Hs.135121 | chr11q24.1 |
| 228080_at | layilin | LAYN | BE856341 | Hs.317614 | chr11q23.1 |
| 228077_at | hypothetical protein MGC3207 | MGC3207 | AK026666 | Hs.439370 | chr19p13.13 |
| 228073_at | N-acetylneuraminic acid phosphatase | NANP | AI146450 |  |  |
| 228072_at | synaptotagmin XII | SYT12 | AK024280 | Hs.287636 | chr11q13.2 |
| 228062_at | nucleosome assembly protein 1-like 5 | NAP1L5 | AW025330 | Hs.12554 | chr4q22.1 |
| 228053_s_at | chromosome 9 open reading frame 105 | C9orf105 | BF965064 |  | chr9p13.1 |
| 228050_at | UTP15, U3 small nucleolar ribonucleoprotein, homolog (S. cerevisiae) | UTP15 | AA046406 | Hs.406703 | chr5q13.2 |
| 228044_at | chromosome 13 open reading frame 21 | C13orf21 | AI420319 | Hs.377972 | chr13q14.11 |
| 228041_at | 2-aminoadipic 6-semialdehyde dehydrogenase | AASDH | AW772229 | Hs.104347 | chr4q12 |
| 228039_at | DEAD (Asp-Glu-Ala-Asp) box polypeptide 46 | DDX46 | AI765169 | Hs.486709 | chr5q31.1 |
| 228033_at | E2F transcription factor 7 | E2F7 | AI341146 | Hs.416375 | chr12q21.2 |
| 228031_at | chromosome 20 open reading frame 121 | C20orf121 | AW444778 |  | chr20q13.12 |
| 228029_at | zinc finger protein 721 | ZNF721 | AW513477 | Hs.435791 | chr4p16.3 |
| 228027_at | G protein-coupled receptor associated sorting protein 2 | GPRASP2 | BF732712 | Hs.348493 | chrxq22.1 |
| 228024_at | Vacuolar protein sorting 37 homolog A (S. cerevisiae) | VPS37A | AW028100 | Hs.309697 | chr8p22 |
| 228023_x_at | amylase, alpha 2B (pancreatic) | AMY2B | AV699389 | Hs.288166 | chr1p21 |
| 228020_at | Pentatricopeptide repeat domain 3 | PTCD3 | BG055431 | Hs.323489 | chr2p11.2 |
| 228019_s_at | mitochondrial ribosomal protein S18C | MRPS18C | AV758614 | Hs.436161 | chr4q21.23 |
| 228014_at | peptidyl-tRNA hydrolase 1 homolog (S. cerevisiae) | PTRH1 | BG030151 | Hs.197141 | chr9q34.11 |
| 228013_at | Protein phosphatase 2 (formerly 2A), regulatory subunit B (PR 52), alpha isoform | PPP2R2A | AV702575 | Hs.179574 | chr8p21.2 |
| 228012_at | Matrin 3 | MATR3 | N53862 |  |  |
| 228011_at | family with sequence similarity 92, member A1 | FAM92A1 | BF338870 | Hs.445714 | chr8q22.1 |
| 228007_at | Chromosome 6 open reading frame 204 | C6orf204 | AL133101 | Hs.536932 |  |
| 228005_at | zinc finger, X-linked, duplicated B | ZXDB | BE677308 | Hs.156257 | chrxp11.1 |
| 228003_at | RAB30, member RAS oncogene family | RAB30 | AW294640 |  |  |
| 228002_at | isopentenyl-diphosphate delta isomerase 2 | IDI2 | AI814569 | Hs.9270 | chr10p15.3 |
| 227999_at | PWWP domain containing 2 | PWWP2 | AI290476 | Hs.157728 | chr10q26.3 |
| 227998_at | S100 calcium binding protein A16 | S100A16 | AA045184 | Hs.8182 | chr1q21 |
| 227997_at | Interleukin 17 receptor D | IL17RD | AW007080 | Hs.284192 | chr3p14.3 |
| 227996_at | FERM, RhoGEF (ARHGEF) and pleckstrin domain protein 1 (chondrocyte-derived) | FARP1 | BF725250 | Hs.403917 | chr13q32.2 |
| 227993_at | methionyl aminopeptidase 2 | METAP2 | AW003997 | Hs.144906 | chr12q22 |
| 227990_at | SLU7 splicing factor homolog (S. cerevisiae) | SLU7 | AA843238 |  |  |
| 227986_at | zinc finger protein 343 | ZNF343 | AW014719 | Hs.317169 | chr20p13 |
| 227983_at | hypothetical protein MGC7036 | MGC7036 | AI810244 | Hs.488173 | chr12q24.31 |
| 227980_at | HLA complex group 12 | HCG12 | AW511258 | Hs.530271 | chr9q22.33 |
| 227979_at | RNA binding motif protein 4B | RBM4B | AU152162 | Hs.49994 | chr11q13 |
| 227977_at | zinc binding alcohol dehydrogenase, domain containing 2 | ZADH2 | BF591611 | Hs.408128 | chr18q22.3 |
| 227973_at | hypothetical protein FLJ38973 | FLJ38973 | BF130937 | Hs.40782 | chr2q33.1 |
| 227972_at | torsin family 2, member A | TOR2A | AA873275 | Hs.444106 | chr9q34.11 |
| 227968_at | Parkinson disease 7 domain containing 1 | PDDC1 | AI139988 | Hs.378971 | chr11p15.5 |
| 227966_s_at | coiled-coil domain containing 74A /// coiled-coil domain containing 74B | CCDC74A | AA524895 | Hs.351461 | chr2q21.1 |
| 227964_at | FKSG44 gene | FKSG44 | BF435621 | Hs.362974 | chr11q13 |
| 227962_at | acyl-Coenzyme A oxidase 1, palmitoyl | ACOX1 | BF435852 | Hs.379991 | chr17q24-q25 |
| 227940_at | Hypothetical protein LOC339803 | LOC339803 | N90774 | Hs.252433 | chr2p16.1 |
| 227936_at | transmembrane protein 68 | TMEM68 | AI671172 | Hs.420076 | chr8q12.1 |
| 227930_at | Eukaryotic translation initiation factor 2C, 4 | EIF2C4 | AI669957 |  |  |
| 227925_at | Hypothetical LOC644563 /// Chromosome 6 open reading frame 51 /// Hypothetical gene supported by AK096370 | LOC644563 | BF343672 | Hs.445673 | chr11q24.2 |
| 227920_at | KIAA1553 | KIAA1553 | AV700885 | Hs.418045 | chr6q21 |
| 227918_s_at | zyg-11 homolog B (C. elegans) | ZYG11B | N21008 | Hs.370232 | chr1p32.3 |
| 227917_at | Similar to CG7889-PA /// CDNA FLJ37098 fis, clone BRACE2019004 /// Hypothetical protein LOC157278 | FLJ10661 | AW192692 | Hs.511708 |  |
| 227916_x_at | exosome component 3 | EXOSC3 | AA747303 | Hs.31293 | chr9p11 |
| 227904_at | 5-azacytidine induced 2 | AZI2 | BF000175 | Hs.437336 | chr3p24.1 |
| 227897_at | RAP2B, member of RAS oncogene family | RAP2B | N20927 |  |  |
| 227896_at | BRCA2 and CDKN1A interacting protein | BCCIP | AI373643 | Hs.337008 | chr10q26.1 |
| 227895_at | family with sequence similarity 120B | FAM120B | BE966082 | Hs.351307 | chr6q27 |
| 227890_at | similar to RIKEN cDNA A230078I05 gene | MGC99813 | AL524643 | Hs.446664 | chr2q35 |
| 227887_at | hypothetical LOC400236 | LOC400236 | AW138940 | Hs.281207 | chr14q32.11 |
| 227884_at | TAF15 RNA polymerase II, TATA box binding protein (TBP)-associated factor, 68kDa | TAF15 | AW296067 | Hs.402752 | chr17q11.1-q11.2 |
| 227882_at | fukutin related protein | FKRP | AW291054 | Hs.193261 | chr19q13.32 |
| 227880_s_at | family with sequence similarity 11, member A | FAM11A | AW300965 | Hs.37106 | chrxq28 |
| 227875_at | kelch-like 13 (Drosophila) | KLHL13 | AB037730 | Hs.348262 | chrxq23-q24 |
| 227870_at | likely ortholog of mouse neighbor of Punc E11 | NOPE | AB046848 | Hs.20924 | chr15q22.31 |
| 227869_at | Family with sequence similarity 104, member B | FAM104B | BG500573 | Hs.415414 | chr17p13.3 |
| 227868_at | hypothetical protein LOC154761 | LOC154761 | AI928764 | Hs.296695 | chr7q34 |
| 227867_at | hypothetical protein LOC129293 | LOC129293 | AA005361 | Hs.36723 | chr2p11.2 |
| 227866_at | Hypothetical protein LOC729436 | LOC729436 | AI743557 | Hs.3737 |  |
| 227864_s_at | family with sequence similarity 125, member A | FAM125A | AI091713 | Hs.408789 | chr19p13.11 |
| 227861_at | transmembrane protein 161B | TMEM161B | AI650251 | Hs.379972 | chr5q14.3 |
| 227859_at | Ras-associated protein Rap1 | RBJ | AV706343 | Hs.434993 | chr2p23.3 |
| 227856_at | chromosome 4 open reading frame 32 | C4orf32 | AI110850 | Hs.23439 | chr4q25 |
| 227846_at | G protein-coupled receptor 176 | GPR176 | AA526584 | Hs.37196 | chr15q14-q15.1 |
| 227840_at | hypothetical protein LOC130355 | LOC130355 | AA738440 | Hs.99488 | chr2q14.2 |
| 227837_at | Hypothetical protein LOC729570 | LOC729570 | BF057711 | Hs.512622 | chr2q33.3 |
| 227822_at | zinc finger protein 605 | ZNF605 | AI341321 | Hs.29698 | chr12q24.33 |
| 227820_at | Emopamil binding protein (sterol isomerase) | EBP | BG171352 | Hs.30619 | chrXp11.3-p11.23 |
| 227812_at | tumor necrosis factor receptor superfamily, member 19 | TNFRSF19 | BF432648 | Hs.334174 | chr13q12.11-q12.3 |
| 227810_at | zinc finger protein 558 | ZNF558 | AW119060 | Hs.349444 | chr19p13.2 |
| 227809_at | Zinc finger CCCH-type containing 6 | ZC3H6 | AI703114 |  |  |
| 227803_at | ectonucleotide pyrophosphatase/phosphodiesterase 5 (putative function) | ENPP5 | AA609053 | Hs.35198 | chr6p21.1-p11.2 |
| 227797_x_at | LYR motif containing 2 | LYRM2 | AI652464 | Hs.483841 | chr6q14.2-q16.1 |
| 227796_at | zinc finger protein 62 homolog (mouse) /// similar to Zinc finger protein 62 homolog (Zfp-62) (ZT3) | ZFP62 | AW157773 | Hs.30503 | chr5p15.33 |
| 227792_at | Hypothetical protein LOC162073 | LOC162073 | AA490685 |  |  |
| 227786_at | thyroid hormone receptor associated protein 6 | THRAP6 | AI026938 | Hs.336898 | chr8q24.11 |
| 227784_s_at | component of oligomeric golgi complex 1 | COG1 | AK025633 | Hs.283109 | chr17q25.1 |
| 227782_at | zinc finger and BTB domain containing 7C | ZBTB7C | AI278995 | Hs.374579 | chr18q21.1 |
| 227768_at | zinc finger protein 407 | ZNF407 | AB051490 | Hs.211805 | chr18q23 |
| 227754_at | ubiquitin specific peptidase 54 | USP54 | AV700815 | Hs.434502 | chr10q22.2 |
| 227748_at | RNA binding motif protein, X-linked-like 1 | RBMXL1 | AI971694 | Hs.134460 | chr1p22.2 |
| 227746_at | ELAV (embryonic lethal, abnormal vision, Drosophila)-like 1 (Hu antigen R) | ELAVL1 | BE047452 | Hs.184492 | chr19p13.2 |
| 227741_at | protein tyrosine phosphatase-like (proline instead of catalytic arginine), member b | PTPLB | AI813654 | Hs.5957 | chr3q21.1 |
| 227740_at | U2AF homology motif (UHM) kinase 1 | UHMK1 | AW173222 | Hs.127310 | chr1q23.3 |
| 227738_s_at | armadillo repeat containing 5 | ARMC5 | BG151528 | Hs.121915 | chr16p11.2 |
| 227737_at | Signal recognition particle receptor, B subunit | SRPRB | AI160339 | Hs.432799 | chr3q22.1 |
| 227732_at | ataxin 7-like 1 | ATXN7L1 | AB033044 | Hs.437205 | chr7q22.1 |
| 227729_at | Zinc finger protein 211 | ZNF211 | AL038092 |  |  |
| 227722_at | ribosomal protein S23 | RPS23 | AW043594 | Hs.534369 | chr5q14.2 |
| 227721_at | C3 and PZP-like, alpha-2-macroglobulin domain containing 8 | CPAMD8 | AB033109 | Hs.375610 | chr19p13.11 |
| 227715_at | similar to hypothetical protein MGC27019 /// similar to Tektin-3 | LOC389833 | AL562298 | Hs.381187 | chr4 |
| 227713_at | katanin p60 subunit A-like 1 | KATNAL1 | AI807482 | Hs.243596 |  |
| 227709_at | Similar to Reticulocalbin-1 precursor | LOC728913 | BE670492 | Hs.381026 | chr13q14.13 |
| 227708_at | eukaryotic translation elongation factor 1 alpha 1 | EEF1A1 | AW469790 |  |  |
| 227705_at | transcription elongation factor A (SII)-like 7 | TCEAL7 | BF591534 | Hs.21861 | chrxq22.1 |
| 227699_at | chromosome 14 open reading frame 149 | C14orf149 | BF511003 | Hs.29706 | chr14q23.1 |
| 227698_s_at | RAB40C, member RAS oncogene family | RAB40C | AW007215 |  |  |
| 227697_at | suppressor of cytokine signaling 3 | SOCS3 | AI244908 | Hs.436943 | chr17q25.3 |
| 227693_at | WD repeat domain 20 | WDR20 | AI092930 | Hs.55209 | chr14q32.32 |
| 227688_at | leucine-rich repeats and calponin homology (CH) domain containing 2 | LRCH2 | AK022128 | Hs.65366 | chrxq23 |
| 227684_at | DNA (cytosine-5-)-methyltransferase 1 | DNMT1 | AI032786 |  |  |
| 227680_at | zinc finger protein 326 | ZNF326 | AI057121 | Hs.306221 | chr1p22.2 |
| 227678_at | XRCC6 binding protein 1 | XRCC6BP1 | AI628122 | Hs.61188 | chr12q14.1 |
| 227677_at | Janus kinase 3 (a protein tyrosine kinase, leukocyte) | JAK3 | BF512748 | Hs.210387 | chr19p13.1 |
| 227668_at | chromosome 17 open reading frame 56 | C17orf56 | AA552953 | Hs.66219 | chr17q25.3 |
| 227658_s_at | pleckstrin homology domain containing, family A (phosphoinositide binding specific) member 3 | PLEKHA3 | BE550332 | Hs.41086 | chr2q31.2 |
| 227656_at | chromosome 6 open reading frame 70 | C6orf70 | AW968493 | Hs.47546 | chr6q27 |
| 227653_at | TRM5 tRNA methyltransferase 5 homolog (S. cerevisiae) | TRMT5 | AI168767 | Hs.380159 | chr14q23.1 |
| 227650_at | heat shock 70kDa protein 14 | HSPA14 | AV757332 | Hs.430666 | chr10p13 |
| 227644_at | regulating synaptic membrane exocytosis 4 | RIMS4 | AL049000 | Hs.349189 | chr20q13.12 |
| 227640_s_at | retinitis pigmentosa 9 (autosomal dominant) /// PNAS-13 | RP9 | AI492167 | Hs.326805 | chr7p14.3 |
| 227639_at | phosphatidylinositol glycan anchor biosynthesis, class K | PIGK | AI275605 | Hs.293653 | chr1p31.1 |
| 227638_at | KIAA1632 | KIAA1632 | AI393091 | Hs.433076 | chr18q12.3 |
| 227636_at | THAP domain containing 5 | THAP5 | BG500677 | Hs.290259 | chr7q31.1 |
| 227632_at | TBC1 domain family, member 24 | TBC1D24 | N74056 | Hs.353087 | chr16p13.3 |
| 227624_at | KIAA1546 | KIAA1546 | AB046766 | Hs.531250 | chr4q24 |
| 227622_at | PCF11, cleavage and polyadenylation factor subunit, homolog (S. cerevisiae) | PCF11 | AW118175 | Hs.123654 | chr11q13 |
| 227616_at | B-cell CLL/lymphoma 9-like | BCL9L | BG481877 | Hs.414740 | chr11q23.3 |
| 227615_at | Tuftelin interacting protein 11 | TFIP11 | BG231758 |  |  |
| 227613_at | zinc finger protein 331 | ZNF331 | AW450874 | Hs.147644 | chr19q13.3-q13.4 |
| 227611_at | threonyl-tRNA synthetase-like 2 | TARSL2 | AA442856 | Hs.498500 | chr15q26.3 |
| 227609_at | epithelial stromal interaction 1 (breast) | EPSTI1 | AA633203 | Hs.343800 | chr13q13.3 |
| 227606_s_at | STAM binding protein-like 1 | STAMBPL1 | AI638611 | Hs.16229 | chr10q23.31 |
| 227598_at | chromosome 7 open reading frame 29 | C7orf29 | AI762857 | Hs.534622 | chr7q36.1 |
| 227593_at | hypothetical protein LOC645580 | FLJ37453 | AW964477 | Hs.119298 |  |
| 227590_at | similar to RIKEN cDNA 2210021J22 | LOC150383 | BE501980 | Hs.275711 | chr22q13.31 |
| 227587_at | hypothetical protein FLJ12949 | FLJ12949 | N46431 | Hs.534560 | chr19p13.2 |
| 227581_at | DKFZP434B0335 protein | DKFZP434B0335 | BE616972 | Hs.22627 | chr7q21.3 |
| 227577_at | exocyst complex component 8 | EXOC8 | AI168350 | Hs.356198 | chr1q42.2 |
| 227575_s_at | chromosome 14 open reading frame 102 | C14orf102 | AI653729 | Hs.210265 | chr14q32.12 |
| 227572_at | Ubiquitin specific peptidase 30 | USP30 | AA528138 | Hs.446158 | chr12q24.11 |
| 227569_at | ligand of numb-protein X 2 | LNX2 | AU151331 | Hs.27179 | chr13q12.2 |
| 227568_at | HECT domain containing 2 | HECTD2 | AF131793 | Hs.437398 | chr10q23.32 |
| 227558_at | chromobox homolog 4 (Pc class homolog, Drosophila) | CBX4 | AI570531 | Hs.405046 | chr17q25.3 |
| 227556_at | non-metastatic cells 7, protein expressed in (nucleoside-diphosphate kinase) | NME7 | AI094580 | Hs.534506 | chr1q24 |
| 227551_at | chromosome 9 open reading frame 77 | C9orf77 | BE856596 | Hs.380389 | chr9q21.13 |
| 227546_x_at | aurora kinase A interacting protein 1 /// similar to Cyclin-L2 (Paneth cell-enhanced expression protein) | AURKAIP1 | AI738987 | Hs.76239 | chr1p36.33 |
| 227544_at | chromosome 14 open reading frame 83 | C14orf83 | H07095 |  | chr14q23.3 |
| 227540_at | eukaryotic elongation factor, selenocysteine-tRNA-specific | EEFSEC | AW614362 | Hs.6522 | chr3q21.3 |
| 227536_at | Zinc finger CCCH-type containing 13 | ZC3H13 | AI472196 | Hs.136102 | chr13q14.13 |
| 227534_at | chromosome 9 open reading frame 21 | C9orf21 | AI655189 | Hs.44640 | chr9q22.32 |
| 227530_at | A kinase (PRKA) anchor protein (gravin) 12 | AKAP12 | BF511276 | Hs.197081 | chr6q24-q25 |
| 227526_at | Cdon homolog (mouse) | CDON | AU151222 | Hs.38034 |  |
| 227525_at | glucocorticoid induced transcript 1 | GLCCI1 | AA058770 | Hs.435806 | chr7p21.3 |
| 227522_at | carboxymethylenebutenolidase homolog (Pseudomonas) | CMBL | AA209487 | Hs.192586 | chr5p15.2 |
| 227521_at | F-box protein 33 | FBXO33 | N22902 | Hs.324342 | chr14q21.1 |
| 227516_at | splicing factor 3a, subunit 1, 120kDa | SF3A1 | AI655996 | Hs.505587 | chr22q12.2 |
| 227515_at | STAM binding protein | STAMBP | AU158421 | Hs.407994 | chr2p13.1 |
| 227510_x_at | PRO1073 protein | PRO1073 | AL037917 |  |  |
| 227507_at | zinc finger protein 592 | ZNF592 | BF593899 | Hs.459179 | chr15q25.3 |
| 227506_at | solute carrier family 16, member 9 (monocarboxylic acid transporter 9) | SLC16A9 | BG401568 | Hs.444696 | chr10q21.2 |
| 227493_s_at | KIAA1143 | KIAA1143 | AI863484 | Hs.173042 | chr3p21.31 |
| 227490_at | WD repeat and FYVE domain containing 2 | WDFY2 | T87542 | Hs.282230 | chr13q14.3 |
| 227481_at | CNKSR family member 3 | CNKSR3 | BG165333 | Hs.16064 | chr6q25.2 |
| 227479_at | KIAA1244 | KIAA1244 | AV712413 | Hs.194408 | chr6q23.3 |
| 227471_at | HECT domain and ankyrin repeat containing, E3 ubiquitin protein ligase 1 | HACE1 | AB037741 | Hs.276471 | chr6q21 |
| 227467_at | retinol dehydrogenase 10 (all-trans) | RDH10 | AV697515 | Hs.356086 | chr8q21.11 |
| 227461_at | stonin 2 | STON2 | AA632295 | Hs.14248 | chr14q31.1 |
| 227454_at | TAO kinase 1 | TAOK1 | AB037782 | Hs.15119 | chr17q11.2 |
| 227451_s_at | Coiled-coil domain containing 90A | CCDC90A | BF507383 | Hs.474401 | chr6p23 |
| 227450_at | chromosome 12 open reading frame 46 | C12orf46 | AI051248 | Hs.162143 | chr12p12.3 |
| 227446_s_at | chromosome 14 open reading frame 167 | C14orf167 | BF445127 | Hs.439201 | chr14q11.2-14q21.3 |
| 227444_at | Armadillo repeat containing, X-linked 4 | ARMCX4 | AW519141 |  |  |
| 227442_at | COX18 cytochrome c oxidase assembly homolog (S. cerevisiae) | COX18 | BG283902 | Hs.356697 | chr4q13.3 |
| 227436_at | OTU domain containing 7B | OTUD7B | BG291552 | Hs.8852 | chr1q21.2 |
| 227435_at | KIAA2018 | KIAA2018 | AI962192 | Hs.439631 | chr3q13.2 |
| 227432_s_at | Insulin receptor | INSR | AI215106 |  |  |
| 227430_at | zinc finger CCCH-type containing 10 | ZC3H10 | AI969773 | Hs.528696 | chr12q13.2 |
| 227428_at | GA binding protein transcription factor, alpha subunit 60kDa | GABPA | BE876628 | Hs.78 | chr21q21.3 |
| 227425_at | RALBP1 associated Eps domain containing 2 | REPS2 | AI984607 | Hs.5740 |  |
| 227424_x_at | chromosome 21 open reading frame 119 | C21orf119 | AI800837 | Hs.58149 | chr21q22.11 |
| 227421_at | chromosome 21 open reading frame 57 | C21orf57 | BF026326 | Hs.58668 | chr21q22.3 |
| 227420_at | tumor necrosis factor, alpha-induced protein 8-like 1 | TNFAIP8L1 | BF338045 | Hs.356359 | chr19p13.3 |
| 227418_at | KIAA1826 | KIAA1826 | AI808746 | Hs.266782 | chr11q22 |
| 227415_at | hypothetical protein LOC283508 | LOC283508 | BF109303 |  | chr13q13.3 |
| 227413_at | ubiquitin-like domain containing CTD phosphatase 1 | UBLCP1 | BF965546 | Hs.42251 | chr5q33.3 |
| 227408_s_at | sorting nexin 25 | SNX25 | AW008976 | Hs.380726 | chr4q35.1 |
| 227407_at | hypothetical protein FLJ90013 | FLJ90013 | AI341541 | Hs.434489 | chr4p15.32 |
| 227399_at | vestigial like 3 (Drosophila) | VGLL3 | AI754423 |  |  |
| 227388_at | tumor suppressor candidate 1 | TUSC1 | AA479016 | Hs.26268 | chr9p21.1 |
| 227385_at | phosphatidic acid phosphatase type 2 domain containing 2 | PPAPDC2 | BF507862 | Hs.29669 |  |
| 227383_at | hypothetical protein LOC727820 | LOC727820 | AW340595 | Hs.105433 | chr1q21.1 |
| 227382_at | cytochrome b5 type B (outer mitochondrial membrane) | CYB5B | AA649048 |  | chr16q22.1 |
| 227374_at | glutamyl-tRNA synthetase 2 (mitochondrial)(putative) | EARS2 | AA833716 | Hs.444353 | chr16p12.3 |
| 227372_s_at | BAI1-associated protein 2-like 1 | BAIAP2L1 | AA496034 | Hs.489237 | chr7q21.3-q22.1 |
| 227370_at | KIAA1946 | KIAA1946 | AW043602 | Hs.172792 | chr2q32.1 |
| 227368_at | Chromosome 6 open reading frame 166 | C6orf166 | AW009630 |  |  |
| 227363_s_at | COX4 neighbor | COX4NB | AI831653 | Hs.173162 | chr16q24 |
| 227354_at | phosphoprotein associated with glycosphingolipid microdomains 1 | PAG1 | BF589359 | Hs.492106 | chr8q21.13 |
| 227345_at | tumor necrosis factor receptor superfamily, member 10d, decoy with truncated death domain | TNFRSF10D | AI738556 | Hs.129844 | chr8p21 |
| 227341_at | Chromosome 10 open reading frame 30 | C10orf30 | AW195407 | Hs.436344 | chr10p13 |
| 227338_at | hypothetical gene supported by BC066916 | LOC440983 | H99038 | Hs.535148 | chr3q25.1 |
| 227327_at | chromosome 19 open reading frame 49 | C19orf49 | BF062538 | Hs.110783 | chr19q13.2 |
| 227325_at | hypothetical protein LOC255783 | LOC255783 | AW172584 | Hs.128690 | chr19q13.33 |
| 227317_at | LIM and cysteine-rich domains 1 | LMCD1 | AA045042 |  |  |
| 227313_at | PRotein Associated with Tlr4 | MGC40499 | AI870866 | Hs.369867 | chr7q22.1 |
| 227309_at | YOD1 OTU deubiquinating enzyme 1 homolog (S. cerevisiae) | YOD1 | AI982535 | Hs.391944 | chr1q32.2 |
| 227301_at | chaperonin containing TCP1, subunit 6A (zeta 1) pseudogene 1 | CCT6AP1 | BE644816 | Hs.378753 /// Hs.510847 | chr7 |
| 227299_at | Cyclin I | CCNI | AA020986 |  |  |
| 227298_at | hypothetical gene supported by AK095117 | FLJ37798 | AI806330 | Hs.79191 | chr6p12.3 |
| 227294_at | zinc finger protein 689 | ZNF689 | AI474448 | Hs.454685 | chr16p11.2 |
| 227292_at | hypothetical protein BC007540 | LOC144097 | AI866590 | Hs.63405 | chr11q13.1 |
| 227291_s_at | bolA homolog 3 (E. coli) | BOLA3 | AI380704 | Hs.61472 | chr2p13.1 |
| 227286_at | coiled-coil domain containing 95 | CCDC95 | AA743390 | Hs.434864 | chr16p11.2 |
| 227284_at | zinc finger protein 766 | ZNF766 | AA081007 | Hs.439662 | chr19q13.41 |
| 227282_at | protocadherin 19 | PCDH19 | AB037734 | Hs.4993 | chrXq13.3 |
| 227279_at | transcription elongation factor A (SII)-like 3 | TCEAL3 | AA847654 | Hs.311776 | chrxq22.2 |
| 227276_at | plexin domain containing 2 | PLXDC2 | AI264121 | Hs.498939 | chr10p12.31 |
| 227271_at | fibroblast growth factor 11 | FGF11 | AU151265 | Hs.31339 | chr17p13.1 |
| 227270_at | hypothetical protein LOC285550 | LOC285550 | BF593037 | Hs.399980 | chr4p15.33 |
| 227267_at | hypothetical protein FLJ35779 | FLJ35779 | AI953478 | Hs.432726 | chr5q13.3 |
| 227263_at | chromosome 8 open reading frame 58 | C8orf58 | AL519427 | Hs.521443 | chr8p21.2 |
| 227259_at | CD47 molecule | CD47 | BF439618 | Hs.446414 | chr3q13.1-q13.2 |
| 227255_at | PDLIM1 interacting kinase 1 like | PDIK1L | AI806633 | Hs.29911 | chr1p36.11 |
| 227251_at | WD repeat domain 22 | WDR22 | AI431597 | Hs.509783 | chr14q23-q24.1 |
| 227250_at | kringle containing transmembrane protein 1 | KREMEN1 | BF221745 | Hs.474502 | chr22q12.1 |
| 227239_at | family with sequence similarity 126, member A | FAM126A | AV734839 | Hs.85603 | chr7p15.3 |
| 227234_at | Zinc finger protein 724 pseudogene | ZNF724P | AW274874 | Hs.369773 |  |
| 227224_at | Ral GEF with PH domain and SH3 binding motif 2 | RALGPS2 | AW003297 | Hs.209609 | chr1q25.2 |
| 227220_at | nuclear transcription factor, X-box binding-like 1 | NFXL1 | AI743731 | Hs.351839 | chr4p12 |
| 227215_at | golgi associated PDZ and coiled-coil motif containing | GOPC | AI337304 | Hs.191539 | chr6q21 |
| 227208_at | coiled-coil domain containing 84 | CCDC84 | BF446390 | Hs.333550 | chr11q23.3 |
| 227207_x_at | zinc finger protein 213 | ZNF213 | BF510572 | Hs.115284 | chr16p13.3 |
| 227205_at | TAF1 RNA polymerase II, TATA box binding protein (TBP)-associated factor, 250kDa | TAF1 | AW575233 |  |  |
| 227198_at | AF4/FMR2 family, member 3 | AFF3 | AW085505 | Hs.38070 | chr2q11.2-q12 |
| 227197_at | Src homology 3 domain-containing guanine nucleotide exchange factor | SGEF | AI989530 | Hs.240845 | chr3q25.2 |
| 227196_at | rhophilin, Rho GTPase binding protein 2 | RHPN2 | BG054987 | Hs.335798 | chr19q13.11 |
| 227195_at | zinc finger protein 503 | ZNF503 | AA603467 | Hs.158210 | chr10q22.2 |
| 227188_at | chromosome 21 open reading frame 63 | C21orf63 | AI744591 | Hs.368175 | chr21q22.11 |
| 227186_s_at | mitochondrial ribosomal protein L41 | MRPL41 | AI090841 | Hs.44017 | chr9q34.3 |
| 227185_at | hypothetical LOC643988 | LOC643988 | BE378494 | Hs.6872 | chr1p36.32 |
| 227177_at | Coronin, actin binding protein, 2A | CORO2A | AL515381 | Hs.439725 | chr9q22.3 |
| 227176_at | solute carrier family 2 (facilitated glucose transporter), member 13 | SLC2A13 | AL565362 | Hs.117979 | chr12q12 |
| 227166_at | DnaJ (Hsp40) homolog, subfamily C, member 18 | DNAJC18 | AI017750 | Hs.4845 | chr5q31.2 |
| 227164_at | splicing factor, arginine/serine-rich 1 (splicing factor 2, alternate splicing factor) | SFRS1 | AL521786 |  |  |
| 227162_at | zinc finger and BTB domain containing 26 | ZBTB26 | BF214688 | Hs.5638 | chr9q33.2 |
| 227161_at | nucleolar protein with MIF4G domain 1 | NOM1 | AI419857 | Hs.436872 | chr7q36.3 |
| 227160_s_at | chromosome 20 open reading frame 7 | C20orf7 | AW102828 | Hs.420282 | chr20p12.1 |
| 227159_at | GH3 domain containing | GHDC | AI827015 | Hs.38039 | chr17q21.2 |
| 227157_at | coiled-coil domain containing 111 | CCDC111 | AV758242 | Hs.534705 | chr4q35.1 |
| 227156_at | trinucleotide repeat containing 8 | TNRC8 | AK025872 | Hs.3327 | chrxp11.4 |
| 227152_at | chromosome 12 open reading frame 35 | C12orf35 | AI979334 | Hs.236844 |  |
| 227148_at | pleckstrin homology domain containing, family H (with MyTH4 domain) member 2 | PLEKHH2 | AI913749 | Hs.255938 | chr2p21 |
| 227146_at | quiescin Q6-like 1 | QSCN6L1 | AW873348 | Hs.144073 | chr9q34.3 |
| 227145_at | lysyl oxidase-like 4 | LOXL4 | AW190565 | Hs.306814 | chr10q24 |
| 227141_at | tRNA-yW synthesizing protein 3 homolog (S. cerevisiae) | TYW3 | AW205739 | Hs.348411 | chr1p31.1 |
| 227132_at | zinc finger protein 706 | ZNF706 | N38730 | Hs.374485 | chr8q22.3 |
| 227129_x_at | hypothetical gene supported by AK127273 | FLJ45340 | AW006934 | Hs.356758 | chr19p13.3 |
| 227125_at | Interleukin 10 receptor, beta | IL10RB | BF526978 |  |  |
| 227123_at | RAB3B, member RAS oncogene family | RAB3B | AU156710 | Hs.123072 | chr1p32-p31 |
| 227119_at | CCR4-NOT transcription complex, subunit 6-like | CNOT6L | BF103856 | Hs.183085 | chr4q13.3 |
| 227114_at | hypothetical protein DKFZp547C195 | DKFZp547C195 | BG435876 |  |  |
| 227113_at | alcohol dehydrogenase, iron containing, 1 | ADHFE1 | BE048349 | Hs.347107 | chr8q13.1 |
| 227111_at | zinc finger and BTB domain containing 34 | ZBTB34 | BG179317 | Hs.177633 | chr9 |
| 227110_at | heterogeneous nuclear ribonucleoprotein C (C1/C2) | HNRPC | AA126793 | Hs.476302 | chr14q11.2 |
| 227109_at | cytochrome P450, family 2, subfamily R, polypeptide 1 | CYP2R1 | AV700558 | Hs.371427 | chr11p15.2 |
| 227106_at | similar to RIKEN cDNA 1110012D08 | LOC440104 | AW139191 | Hs.445532 | chr12q13.2 |
| 227102_at | tripartite motif-containing 35 | TRIM35 | AA115933 | Hs.137732 | chr8p21.2 |
| 227101_at | zinc finger protein 800 | ZNF800 | AF218032 | Hs.159006 | chr7q32.2 |
| 227099_s_at | hypothetical LOC387763 | LOC387763 | AW276078 | Hs.448680 | chr11p11.2 |
| 227098_at | dual specificity phosphatase 18 | DUSP18 | BC004110 | Hs.128782 | chr22q12.2 |
| 227096_at | Josephin domain containing 2 | JOSD2 | AI218850 | Hs.116708 | chr19q13.33 |
| 227086_at | HIR histone cell cycle regulation defective homolog A (S. cerevisiae) | HIRA | AA194312 | Hs.433195 | chr22q11.21 |
| 227081_at | dynein, axonemal, light intermediate chain 1 | DNALI1 | AW299538 | Hs.406050 | chr1p35.1 |
| 227080_at | zinc finger protein 697 | ZNF697 | AW003092 | Hs.381105 | chr1p12 |
| 227077_at | zinc finger protein 286 | ZNF286 | BF432238 | Hs.22216 | chr17p11.2 |
| 227066_at | MOB1, Mps One Binder kinase activator-like 2C (yeast) | MOBKL2C | BG167841 | Hs.97927 | chr1p33 |
| 227063_at | chromosome 17 open reading frame 61 | C17orf61 | BF975929 | Hs.433154 | chr17p13.1 |
| 227062_at | trophoblast-derived noncoding RNA | TncRNA | AU155361 | Hs.240443 | chr11q13.1 |
| 227056_at | KIAA0141 | KIAA0141 | AA181172 | Hs.210532 | chr5q31.3 |
| 227048_at | laminin, alpha 1 | LAMA1 | AI990816 | Hs.270364 | chr18p11.31 |
| 227047_x_at | zinc finger and BTB domain containing 4 | ZBTB4 | N63748 | Hs.35096 | chr17p13.1 |
| 227046_at | solute carrier family 39 (metal ion transporter), member 11 | SLC39A11 | BF062384 | Hs.3402 | chr17q24.3-q25.1 |
| 227045_at | zinc finger protein 614 | ZNF614 | AI087872 | Hs.426228 | chr19q13.41 |
| 227040_at | similar to RIKEN cDNA 8030451K01 | RP11-50D16.3 | AI655763 | Hs.356618 | chr13q13.3 |
| 227039_at | A kinase (PRKA) anchor protein 13 | AKAP13 | AI674926 | Hs.350631 | chr15q24-q25 |
| 227037_at | similar to CG12314 gene product | LOC201164 | AA029500 | Hs.31652 | chr17p11.2 |
| 227035_x_at | PNAS-13 | LOC441212 | BE670798 | Hs.292057 | chr7 |
| 227033_at | protein disulfide isomerase family A, member 3 | PDIA3 | AI825800 | Hs.308709 | chr15q15 |
| 227029_at | chromosome 14 open reading frame 24 | C14orf24 | AI949662 | Hs.446357 | chr14q13.2 |
| 227025_at | periphilin 1 | PPHLN1 | BG284497 | Hs.371140 | chr12q12 |
| 227022_at | glucosamine-6-phosphate deaminase 2 | GNPDA2 | AI817388 | Hs.511814 | chr4p13 |
| 227021_at | amine oxidase (flavin containing) domain 1 | AOF1 | BE348688 | Hs.237691 | chr6p22.3 |
| 227020_at | yippee-like 2 (Drosophila) | YPEL2 | BE502982 | Hs.463613 | chr17q23.2 |
| 227018_at | dipeptidyl-peptidase 8 | DPP8 | AV734194 | Hs.319095 | chr15q22 |
| 227016_at | glutamate-rich 1 | ERICH1 | AA767385 | Hs.169802 | chr8p23.3 |
| 227014_at | aspartate beta-hydroxylase domain containing 2 | ASPHD2 | BE550881 | Hs.534545 | chr22q12.1 |
| 227012_at | solute carrier family 25, member 40 | SLC25A40 | AI823986 | Hs.208414 | chr7q21.12 |
| 227007_at | transmembrane and coiled-coil domains 4 | TMCO4 | AI972419 | Hs.43018 | chr1p36.13 |
| 227006_at | protein phosphatase 1, regulatory (inhibitor) subunit 14A | PPP1R14A | AA156998 | Hs.348037 | chr19q13.1 |
| 227005_at | Ribonuclease P 14kDa subunit | RPP14 | BF439230 |  |  |
| 227004_at | Cyclin-dependent kinase-like 5 | CDKL5 | AI611074 |  |  |
| 227002_at | family with sequence similarity 78, member A | FAM78A | BF515132 | Hs.143878 | chr9q34 |
| 226996_at | lysocardiolipin acyltransferase | LYCAT | AV717041 | Hs.14355 | chr2p23.1 |
| 226992_at | nitric oxide synthase trafficker | NOSTRIN | AK002203 | Hs.416324 | chr2q24.3 |
| 226989_at | RGM domain family, member B | RGMB | BE855765 | Hs.161394 | chr5q21.1 |
| 226987_at | RNA binding motif protein 15B | RBM15B | W68720 | Hs.84162 | chr3p21.2 |
| 226977_at | similar to bovine IgA regulatory protein | LOC492311 | N26029 | Hs.293782 |  |
| 226975_at | RNA-binding region (RNP1, RRM) containing 3 | RNPC3 | BF116157 | Hs.534533 | chr1p21 |
| 226973_at | chromosome 20 open reading frame 102 | C20orf102 | AA206763 | Hs.7991 | chr20q11.23 |
| 226969_at | hypothetical protein LOC149448 | LOC149448 | AV706396 |  | chr1q42.3 |
| 226965_at | family with sequence similarity 116, member A | FAM116A | BF438017 | Hs.91085 | chr3p14.3 |
| 226962_at | zinc finger and BTB domain containing 41 | ZBTB41 | AW299558 | Hs.509424 | chr1q31.3 |
| 226958_s_at | mediator of RNA polymerase II transcription, subunit 11 homolog (S. cerevisiae) | MED11 | AL531790 | Hs.82407 | chr17p13.2 |
| 226957_x_at | ralA binding protein 1 | RALBP1 | BF433757 | Hs.75447 | chr18p11.3 |
| 226952_at | ELL associated factor 1 | EAF1 | AU149385 | Hs.474479 | chr3p25.1 |
| 226949_at | golgi autoantigen, golgin subfamily a, 3 | GOLGA3 | N25563 | Hs.4953 | chr12q24.33 |
| 226947_at | glucuronidase, beta-like 2 | GUSBL2 | AW001628 | Hs.186180 | chr6 |
| 226946_at | chromosome 5 open reading frame 33 | C5orf33 | AI453203 | Hs.81907 | chr5p13.2 |
| 226944_at | HtrA serine peptidase 3 | HTRA3 | AW518728 | Hs.390421 | chr4p16.1 |
| 226938_at | WD repeat domain 21A | WDR21A | AA160604 | Hs.331491 | chr14q24.3 |
| 226936_at | chromosome 6 open reading frame 173 | C6orf173 | BG492359 | Hs.35962 |  |
| 226933_s_at | Inhibitor of DNA binding 4, dominant negative helix-loop-helix protein | ID4 | AV646610 |  |  |
| 226932_at | Sarcospan (Kras oncogene-associated gene) | SSPN | AW467136 |  |  |
| 226927_at | Hypothetical protein LOC728568 | LOC728568 | AU144442 | Hs.42547 |  |
| 226926_at | dermokine | DMKN | AA706316 | Hs.417795 | chr19q13.12 |
| 226924_at | hypothetical gene supported by BC036588 | LOC400657 | AI016355 | Hs.61508 | chr18q22.3 |
| 226921_at | ubiquitin protein ligase E3 component n-recognin 1 | UBR1 | AV715153 | Hs.145209 | chr15q13 |
| 226909_at | KIAA1729 protein | KIAA1729 | AW270138 | Hs.99073 | chr4p16.1 |
| 226908_at | leucine-rich repeats and immunoglobulin-like domains 3 | LRIG3 | AI627704 | Hs.19669 | chr12q14.1 |
| 226901_at | chromosome 17 open reading frame 58 | C17orf58 | AI214996 | Hs.90790 | chr17q24.2 |
| 226899_at | unc-5 homolog B (C. elegans) | UNC5B | AK022859 | Hs.13350 | chr10q22.1 |
| 226897_s_at | zinc finger CCCH-type containing 7A | ZC3H7A | AW169959 | Hs.371856 | chr16p13-p12 |
| 226896_at | coiled-coil-helix-coiled-coil-helix domain containing 1 | CHCHD1 | AI992290 | Hs.181174 | chr10q22.2 |
| 226892_at | chromosome 10 open reading frame 12 | C10orf12 | AK025166 | Hs.14555 | chr10q24.1 |
| 226881_at | GrpE-like 2, mitochondrial (E. coli) | GRPEL2 | BF185904 | Hs.17121 | chr5q32 |
| 226876_at | family with sequence similarity 101, member B | FAM101B | AI961778 | Hs.345588 | chr17p13 |
| 226871_s_at | ATG4 autophagy related 4 homolog D (S. cerevisiae) | ATG4D | BF791801 | Hs.104938 | chr19p13.2 |
| 226870_at | catechol-O-methyltransferase domain containing 1 | COMTD1 | AI924025 | Hs.355333 | chr10q22.2 |
| 226864_at | Protein kinase (cAMP-dependent, catalytic) inhibitor alpha | PKIA | BF245954 | Hs.534360 | chr8q21.11 |
| 226863_at | family with sequence similarity 110 member C | FAM110C | AI674565 | Hs.8379 |  |
| 226859_at | DnaJ-like protein | bA16L21.2.1 | AI765321 | Hs.433898 | chr9q31.3 |
| 226850_at | sulfatase modifying factor 1 | SUMF1 | AA683501 | Hs.350475 | chr3p26.1 |
| 226849_at | DENN/MADD domain containing 1A | DENND1A | AB046828 | Hs.300842 | chr9q33.3 |
| 226848_at | Nuclear receptor subfamily 2, group C, member 2 | NR2C2 | AI571166 | Hs.378877 | chr3p25 |
| 226845_s_at | myeloma overexpressed 2 | MYEOV2 | AL036350 | Hs.293884 | chr2q37.3 |
| 226843_s_at | PAP associated domain containing 5 | PAPD5 | AU146704 | Hs.294030 | chr16q12.1 |
| 226842_at | hypothetical protein LOC90110 | LOC90110 | AV694039 |  | chr17q21.2 |
| 226838_at | tetratricopeptide repeat domain 32 | TTC32 | BE465877 | Hs.127767 | chr2p24.1 |
| 226832_at | Ring finger protein 168 /// Hypothetical protein LOC727900 | RNF168 | BF978778 | Hs.250648 | chr3q29 |
| 226828_s_at | hairy/enhancer-of-split related with YRPW motif-like | HEYL | AL040198 | Hs.23823 | chr1p34.3 |
| 226821_at | RAP1 interacting factor homolog (yeast) | RIF1 | R41296 | Hs.19150 |  |
| 226820_at | FLJ25476 protein | FLJ25476 | W72455 | Hs.396807 | chr1p35.1 |
| 226807_at | zinc finger protein 1 homolog (mouse) | ZFP1 | AL038511 | Hs.388813 | chr16q22.3 |
| 226805_at | chromosome 20 open reading frame 142 | C20orf142 | AW006621 |  |  |
| 226803_at | chromatin modifying protein 4C | CHMP4C | AK000049 |  |  |
| 226801_s_at | hypothetical LOC646890 /// hypothetical protein LOC731076 | LOC646890 | W72220 | Hs.428779 | chr1q41 |
| 226800_at | KIAA1799 protein | KIAA1799 | AL109925 | Hs.269768 | chr1p31.3 |
| 226794_at | syntaxin binding protein 5 (tomosyn) | STXBP5 | AI825998 | Hs.93534 | chr6q24.3 |
| 226790_at | MORN repeat containing 2 | MORN2 | AW015683 | Hs.99841 | chr2p22.1 |
| 226786_at | regulatory factor X, 1 (influences HLA class II expression) | RFX1 | BF507952 | Hs.73677 | chr19p13.1 |
| 226785_at | ATPase, Class VI, type 11C | ATP11C | BF475862 | Hs.88252 | chrxq27.1 |
| 226784_at | TWIST neighbor | TWISTNB | AA121481 | Hs.353035 | chr7p21.1 |
| 226782_at | solute carrier family 25, member 30 | SLC25A30 | BF001919 | Hs.132543 | chr13q14.13 |
| 226781_at | hypothetical protein HSPC268 | HSPC268 | BF540829 | Hs.370475 | chr7q34 |
| 226778_at | chromosome 8 open reading frame 42 | C8orf42 | AI632224 | Hs.289293 | chr8p23.3 |
| 226776_at | Enhancer of yellow 2 homolog (Drosophila) | ENY2 | BF433516 |  |  |
| 226770_at | membrane associated guanylate kinase, WW and PDZ domain containing 3 | MAGI3 | AI692181 | Hs.356314 | chr1p12-p11.2 |
| 226767_s_at | fumarylacetoacetate hydrolase domain containing 1 | FAHD1 | AI341467 | Hs.322482 | chr16p13.3 |
| 226763_at | SEC14 and spectrin domains 1 | SESTD1 | AW409611 | Hs.444228 | chr2q31.2 |
| 226760_at | hypothetical protein LOC203411 | LOC203411 | BF666325 |  | chrxp22.13 |
| 226759_at | IKAROS family zinc finger 4 (Eos) | IKZF4 | AI261467 | Hs.388622 | chr12q13 |
| 226752_at | transmembrane protein 157 | TMEM157 | AI816071 | Hs.356108 | chr5q21.1 |
| 226751_at | chromosome 2 open reading frame 32 | C2orf32 | AW193693 | Hs.371496 | chr2p14 |
| 226749_at | mitochondrial ribosomal protein S9 | MRPS9 | AL582429 | Hs.385862 | chr2q12.1 |
| 226748_at | LysM, putative peptidoglycan-binding, domain containing 2 | LYSMD2 | AI674731 | Hs.293658 | chr15q21.2 |
| 226747_at | KIAA1344 | KIAA1344 | AB037765 | Hs.292164 | chr14q22.1 |
| 226745_at | cytochrome P450, family 4, subfamily V, polypeptide 2 | CYP4V2 | AU146978 | Hs.237642 | chr4q35.1-q35.2 |
| 226741_at | solute carrier family 12 (potassium/chloride transporters), member 6 | SLC12A6 | N21320 | Hs.250905 | chr15q14 |
| 226740_x_at | neuroblastoma breakpoint family, member 14 /// neuroblastoma breakpoint family, member 1 /// neuroblastoma breakpoint family, member 11 /// neuroblastoma breakpoint family, member 15 /// neuroblastoma breakpoint family, member 10 /// neuroblastoma breakpo | NBPF14 | BF740216 | Hs.488862 | chr1q21.1 /// chr1q21.2 /// chr1q12-1q21.2 /// chr1 |
| 226739_at | ring finger protein 169 | RNF169 | AA536004 | Hs.203755 | chr11q13.4 |
| 226738_at | WD repeat domain 81 | WDR81 | BE675297 | Hs.234572 | chr17p13.3 |
| 226734_at | Eukaryotic translation initiation factor 4E family member 2 | EIF4E2 | AW242220 | Hs.211823 | chr2q37.1 |
| 226732_at | RNA binding motif protein 33 | RBM33 | AI823400 | Hs.17240 | chr7q36.3 |
| 226730_s_at | ubiquitin specific peptidase 37 | USP37 | BF789968 | Hs.166068 | chr2q35 |
| 226728_at | solute carrier family 27 (fatty acid transporter), member 1 | SLC27A1 | BF056007 | Hs.363138 | chr19p13.11 |
| 226727_at | hypothetical protein LOC284106 | LOC284106 | BG171264 | Hs.435390 | chr17q12 |
| 226726_at | membrane bound O-acyltransferase domain containing 2 | MBOAT2 | W63676 | Hs.90797 | chr2p25.1 |
| 226723_at | coiled-coil domain containing 23 | CCDC23 | AI581511 | Hs.113919 | chr1p34.2 |
| 226715_at | forkhead box K1 | FOXK1 | AW007319 | Hs.536022 |  |
| 226714_at | sterile alpha motif domain containing 4B | SAMD4B | AA149818 | Hs.534523 | chr19q13.2 |
| 226713_at | coiled-coil domain containing 50 | CCDC50 | AI247881 | Hs.478682 | chr3q28 |
| 226712_at | Signal sequence receptor, alpha (translocon-associated protein alpha) | SSR1 | BF206389 | Hs.101810 |  |
| 226711_at | forkhead box N2 | FOXN2 | BF590117 | Hs.103126 | chr2p22-p16 |
| 226710_at | similar to RIKEN cDNA C030006K11 gene | MGC70857 | AI199072 | Hs.534997 | chr8q24 |
| 226707_at | nicotinate phosphoribosyltransferase domain containing 1 | NAPRT1 | AI925574 | Hs.333388 | chr8q24.3 |
| 226706_at | hypothetical protein FLJ23867 | FLJ23867 | BE301252 |  | chr1q25.1 |
| 226704_at | ubiquitin-conjugating enzyme E2, J2 (UBC6 homolog, yeast) | UBE2J2 | R76659 | Hs.441183 | chr1p36.33 |
| 226696_at | retinoblastoma binding protein 9 | RBBP9 | AI761595 | Hs.69330 | chr20p11.2 |
| 226692_at | Small EDRK-rich factor 2 | SERF2 | AI092931 | Hs.424126 | chr15q15.3 |
| 226689_at | zinc finger, CDGSH-type domain 2 | ZCD2 | AI749451 | Hs.295953 |  |
| 226683_at | Sorting nexin associated golgi protein 1 | SNAG1 | AU146771 | Hs.432755 | chr5q11.2 |
| 226680_at | IKAROS family zinc finger 5 (Pegasus) | IKZF5 | BF056303 | Hs.29397 | chr10q26 |
| 226679_at | solute carrier family 26, member 11 | SLC26A11 | BE964598 | Hs.4866 | chr17q25.3 |
| 226674_at | transmembrane protein 58 | TMEM58 | AL522395 | Hs.423618 | chr1q32.1 |
| 226671_at | Lysosomal-associated membrane protein 2 | LAMP2 | AI150000 | Hs.537071 |  |
| 226668_at | WD repeat, sterile alpha motif and U-box domain containing 1 | WDSUB1 | W80623 | Hs.41840 | chr2q24.2 |
| 226664_at | TBC1 domain family, member 20 | TBC1D20 | AL121747 | Hs.203514 | chr20p13 |
| 226657_at | transcript expressed during hematopoiesis 2 | MGC33894 | H27948 | Hs.14478 | chr17p11.1 |
| 226653_at | MAP/microtubule affinity-regulating kinase 1 | MARK1 | AB040910 | Hs.12808 | chr1q41 |
| 226652_at | ubiquitin specific peptidase 3 | USP3 | AA523542 |  |  |
| 226651_at | homer homolog 1 (Drosophila) | HOMER1 | AW052119 | Hs.129051 | chr5q14.2 |
| 226650_at | zinc finger, AN1-type domain 2A | ZFAND2A | AI984061 | Hs.116567 | chr7p22.3 |
| 226649_at | pantothenate kinase 1 | PANK1 | AI373299 | Hs.116122 | chr10q23.31 |
| 226644_at | mindbomb homolog 2 (Drosophila) | MIB2 | BE222279 |  |  |
| 226642_s_at | NudC domain containing 2 | NUDCD2 | BF735901 | Hs.140443 | chr5q34 |
| 226641_at | Ankyrin repeat domain 44 | ANKRD44 | AU157224 | Hs.11571 | chr2q33.1 |
| 226639_at | SFT2 domain containing 3 | SFT2D3 | AI304320 | Hs.101660 | chr2q14.3 |
| 226631_at | similar to CG9643-PA | LOC399818 | C06331 | Hs.468488 | chr10q26.13 |
| 226619_at | SUMO1/sentrin specific peptidase 1 | SENP1 | AW665440 | Hs.389263 | chr12q13.1 |
| 226612_at | similar to CG4502-PA | FLJ25076 | H17038 | Hs.351357 | chr5p15.31 |
| 226611_s_at | proline rich 6 | PRR6 | AA722878 | Hs.433422 | chr17p11.2 |
| 226609_at | discoidin, CUB and LCCL domain containing 1 | DCBLD1 | N22751 | Hs.449321 | chr6q22.1 |
| 226604_at | transmembrane and tetratricopeptide repeat containing 3 | TMTC3 | AA418403 | Hs.197687 | chr12q21.33 |
| 226599_at | KIAA1727 protein | KIAA1727 | AA527080 | Hs.319946 | chr4q31.3 |
| 226597_at | receptor accessory protein 6 | REEP6 | AI348159 | Hs.76277 | chr19p13.3 |
| 226596_x_at | hypothetical protein LOC729852 /// hypothetical protein LOC730358 /// hypothetical protein LOC730538 | LOC729852 | AK027125 | Hs.112716 | chr7p22.1 |
| 226594_at | Ectonucleoside triphosphate diphosphohydrolase 5 | ENTPD5 | AA528157 | Hs.285290 | chr14q24.3 |
| 226592_at | hypothetical protein LOC286334 | LOC286334 | AA031404 | Hs.349208 | chr9q33.1 |
| 226589_at | hypothetical protein FLJ38482 | FLJ38482 | BE964222 |  |  |
| 226588_at | KIAA1604 protein | KIAA1604 | AB046824 | Hs.311363 | chr2q31.3 |
| 226583_at | FLJ40142 protein | FLJ40142 | AL560962 | Hs.44817 | chr12q24.11 |
| 226582_at | hypothetical gene supported by BC009385 | LOC400043 | AL520272 | Hs.19193 | chr12q13.13 |
| 226581_at | zinc finger, FYVE domain containing 20 | ZFYVE20 | AA524034 |  |  |
| 226576_at | Rho GTPase activating protein 26 | ARHGAP26 | AI768563 | Hs.132942 | chr5q31 |
| 226575_at | zinc finger protein 462 | ZNF462 | T89120 | Hs.16493 | chr9q31.2 |
| 226572_at | Suppressor of cytokine signaling 7 | SOCS7 | AL045666 | Hs.270564 | chr17q12 |
| 226568_at | family with sequence similarity 102, member B | FAM102B | AI478747 | Hs.141269 | chr1p13.3 |
| 226566_at | tripartite motif-containing 11 | TRIM11 | AI885021 | Hs.13543 | chr1q42.13 |
| 226565_at | transmembrane protein 99 | TMEM99 | AW054855 | Hs.353163 | chr17q21.2 |
| 226562_at | zinc finger protein 690 | ZNF690 | BE622486 | Hs.418287 | chr15q15.3 |
| 226561_at | hypothetical protein LOC285086 | LOC285086 | BE551849 | Hs.352962 | chr2q36.3 |
| 226558_at | similar to CG32820-PA, isoform A | LOC653071 | BE856637 | Hs.381187 /// Hs.481434 /// Hs.543822 | chr4p16.3 /// chr4 |
| 226557_at | tetratricopeptide repeat domain 5 | TTC5 | AW044606 | Hs.102480 | chr14q11.2 |
| 226551_at | receptor (TNFRSF)-interacting serine-threonine kinase 1 | RIPK1 | AI743085 | Hs.390758 | chr6p25.2 |
| 226543_at | muted homolog (mouse) | MUTED | AI768114 |  | chr6p25.1-p24.3 |
| 226541_at | F-box protein 30 | FBXO30 | AI808182 | Hs.421095 | chr6q24 |
| 226537_at | histidine triad nucleotide binding protein 3 | HINT3 | AW418666 | Hs.72325 | chr6q22.32 |
| 226536_at | non-SMC element 2, MMS21 homolog (S. cerevisiae) | NSMCE2 | AL562908 | Hs.368147 | chr8q24.13 |
| 226534_at | KIT ligand | KITLG | AI446414 | Hs.5921 | chr12q22 |
| 226524_at | chromosome 3 open reading frame 38 | C3orf38 | AI458735 | Hs.406060 | chr3p11.1 |
| 226520_at | Ligand dependent nuclear receptor corepressor | LCOR | AI831506 |  |  |
| 226513_at | hypothetical protein LOC145758 | LOC145758 | AW003222 |  | chr15q26.3 |
| 226510_at | HEAT repeat containing 5A | HEATR5A | BF435286 | Hs.509053 | chr14q12 |
| 226504_at | family with sequence similarity 109, member B | FAM109B | AA522720 | Hs.25614 | chr22q13.2 |
| 226501_at | X-prolyl aminopeptidase (aminopeptidase P) 3, putative | XPNPEP3 | BE966628 | Hs.20797 | chr22q13.31-q13.33 |
| 226493_at | potassium channel tetramerisation domain containing 18 | KCTD18 | AI627249 | Hs.379185 | chr2q33.1 |
| 226492_at | sema domain, transmembrane domain (TM), and cytoplasmic domain, (semaphorin) 6D | SEMA6D | AL036088 | Hs.191098 | chr15q21.1 |
| 226489_at | transmembrane and coiled-coil domain family 3 | TMCC3 | BG177562 | Hs.173392 | chr12q22 |
| 226478_at | Transmembrane 7 superfamily member 3 | TM7SF3 | BF540749 | Hs.10071 | chr12q11-q12 |
| 226477_at | Vpr (HIV-1) binding protein | VPRBP | AK023239 | Hs.118738 | chr3p21.2 |
| 226474_at | NLR family, CARD domain containing 5 | NLRC5 | AA005023 | Hs.377097 | chr16q13 |
| 226472_at | peptidylprolyl isomerase (cyclophilin)-like 4 | PPIL4 | BF515889 | Hs.11065 | chr6q24-q25 |
| 226466_s_at | family with sequence similarity 58, member A | FAM58A | AL544688 | Hs.13580 | chrxq28 |
| 226464_at | chromosome 3 open reading frame 58 | C3orf58 | BE348597 | Hs.288954 | chr3q24 |
| 226463_at | ATPase, H+ transporting, lysosomal 42kDa, V1 subunit C1 | ATP6V1C1 | AW241758 |  |  |
| 226454_at | membrane-associated ring finger (C3HC4) 9 | 09-mars | BF508604 | Hs.65377 | chr12q14.1 |
| 226453_at | ribonuclease H2, subunit C | RNASEH2C | BF982002 | Hs.534587 | chr11q13.1 |
| 226451_at | similar to RIKEN cDNA B230118G17 gene | MGC19604 | AI453531 | Hs.159366 | chr19p13.2 |
| 226445_s_at | tripartite motif-containing 41 | TRIM41 | AI743109 | Hs.346945 | chr5q35.3 |
| 226444_at | Solute carrier family 39 (zinc transporter), member 10 | SLC39A10 | AI700476 | Hs.32793 | chr2q32.3 |
| 226443_at | family with sequence similarity 122A | FAM122A | BG397561 | Hs.217748 | chr9q21.11 |
| 226439_s_at | neurobeachin | NBEA | AI246710 | Hs.3821 | chr13q13 |
| 226438_at | Mdm2, transformed 3T3 cell double minute 2, p53 binding protein (mouse) binding protein, 104kDa | MTBP | AK025100 | Hs.432544 | chr8q23-q24 |
| 226437_at | Yip1 interacting factor homolog B (S. cerevisiae) | YIF1B | AI079540 | Hs.280741 | chr19q13.2 |
| 226434_at | chromosome 7 open reading frame 47 | C7orf47 | BF000655 | Hs.413359 | chr7q22.1 |
| 226430_at | hypothetical protein LOC253981 | LOC253981 | AI394438 | Hs.283378 | chr4p14 |
| 226429_at | KIAA1704 | KIAA1704 | BE218238 | Hs.283709 | chr13q13-q14 |
| 226428_at | transportin 2 (importin 3, karyopherin beta 2b) | TNPO2 | AI885873 | Hs.278378 | chr19p13.13 |
| 226425_at | restin-like 2 | RSNL2 | AU144247 | Hs.341806 | chr2p23.2 |
| 226423_at | progestin and adipoQ receptor family member VIII | PAQR8 | AW006774 | Hs.239388 | chr6p12.1 |
| 226416_at | three prime histone mRNA exonuclease 1 | THEX1 | AL137679 | Hs.20000 | chr8p23.1 |
| 226414_s_at | APC11 anaphase promoting complex subunit 11 homolog (yeast) /// similar to APC11 anaphase promoting complex subunit 11 isoform 2 | ANAPC11 | BG180679 | Hs.500798 | chr17q25.3 |
| 226413_at | hypothetical gene supported by BC047417 | LOC400027 | AA044705 | Hs.17872 | chr12q12 |
| 226406_at | chromosome 18 open reading frame 25 | C18orf25 | AI823360 | Hs.33862 | chr18q21.1 |
| 226405_s_at | arrestin domain containing 1 | ARRDC1 | AK001822 | Hs.12999 | chr9q34.3 |
| 226404_at | RNA binding motif protein 39 /// similar to RNA-binding region-containing protein 2 (Hepatocellular carcinoma protein 1) (Splicing factor HCC1) | RBM39 | AA768866 | Hs.282901 | chr20q11.22 |
| 226398_s_at | chromosome 10 open reading frame 4 | C10orf4 | AI804930 | Hs.303727 | chr10q23.33 |
| 226395_at | hypothetical protein LOC286170 | LOC286170 | AI912618 | Hs.102495 | chr8p11.21 |
| 226394_at | membrane-associated ring finger (C3HC4) 5 | 05-mars | BE966193 | Hs.343748 | chr10q23.32-q23.33 |
| 226393_at | cytochrome P450, family 2, subfamily U, polypeptide 1 | CYP2U1 | AW055161 | Hs.109087 | chr4q25 |
| 226392_at | RAS p21 protein activator 2 | RASA2 | AI888503 | Hs.98445 |  |
| 226391_at | NADH dehydrogenase (ubiquinone) 1 beta subcomplex, 2, 8kDa | NDUFB2 | AW271788 | Hs.27262 | chr7q34 |
| 226390_at | START domain containing 4, sterol regulated | STARD4 | AA628398 | Hs.93842 | chr5q22.1 |
| 226388_at | transcription elongation factor A (SII), 3 | TCEA3 | AI675780 | Hs.446354 | chr1p36.12 |
| 226385_s_at | chromosome 7 open reading frame 30 | C7orf30 | BG397444 | Hs.87385 | chr7p15.3 |
| 226383_at | Chromosome 11 open reading frame 46 | C11orf46 | AI652452 |  |  |
| 226381_at | HBV preS1-transactivated protein 4 | PS1TP4 | AW450329 | Hs.355655 | chr2 |
| 226378_s_at | chromosome 19 open reading frame 25 | C19orf25 | AI554705 | Hs.532840 | chr19p13.3 |
| 226374_at | Coxsackie virus and adenovirus receptor | CXADR | BG260087 |  |  |
| 226370_at | kelch-like 15 (Drosophila) | KLHL15 | BG149487 | Hs.61603 | chrxp22.1-p21 |
| 226366_at | SNF2 histone linker PHD RING helicase | SHPRH | AI828221 | Hs.196953 | chr6q24.3 |
| 226363_at | ATP-binding cassette, sub-family C (CFTR/MRP), member 5 | ABCC5 | BE550362 | Hs.22010 | chr3q27 |
| 226361_at | transmembrane protein 42 | TMEM42 | BF056095 | Hs.432780 | chr3p21.31 |
| 226360_at | zinc and ring finger 3 | ZNRF3 | AK022809 | Hs.18919 | chr22q12.1 |
| 226358_at | hypothetical protein LOC145842 | LOC145842 | AW237258 | Hs.528457 | chr15q22.2 |
| 226357_at | ubiquitin specific peptidase 19 | USP19 | AW473649 | Hs.255596 | chr3p21.31 |
| 226356_at | family with sequence similarity 73, member B | FAM73B | AA535864 | Hs.325309 | chr9q34.11 |
| 226353_at | signal peptide peptidase-like 2A | SPPL2A | AI674647 | Hs.28980 | chr15q21.2 |
| 226350_at | choroideremia-like (Rab escort protein 2) | CHML | AU155565 | Hs.534313 | chr1q42-qter |
| 226349_at | chromosome 12 open reading frame 45 | C12orf45 | BE264828 |  |  |
| 226346_at | hypothetical protein LOC92312 | LOC92312 | AA527151 |  |  |
| 226344_at | zinc finger, matrin type 1 | ZMAT1 | AI741051 | Hs.296317 | chrXq21 |
| 226342_at | spectrin, beta, non-erythrocytic 1 | SPTBN1 | AW593244 | Hs.205401 |  |
| 226339_at | TruB pseudouridine (psi) synthase homolog 1 (E. coli) | TRUB1 | AW500239 | Hs.21187 | chr10q25.3 |
| 226337_at | SCY1-like 1 binding protein 1 | SCYL1BP1 | AK021814 | Hs.183702 | chr1q24.2 |
| 226334_s_at | AHA1, activator of heat shock 90kDa protein ATPase homolog 2 (yeast) | AHSA2 | AW117717 | Hs.122440 | chr2p15 |
| 226332_at | hypothetical protein MGC40405 /// hypothetical protein LOC728066 /// hypothetical protein LOC728153 | MGC40405 | AI569932 | Hs.119172 | chr7q21.2 |
| 226329_s_at | MIT, microtubule interacting and transport, domain containing 1 | MITD1 | BF055289 | Hs.14222 | chr2q11.2 |
| 226327_at | zinc finger protein 507 | ZNF507 | N64593 | Hs.144028 | chr19q13.11 |
| 226323_at | coiled-coil domain containing 16 | CCDC16 | AA398517 | Hs.413678 | chr17q12 |
| 226321_at | LysM, putative peptidoglycan-binding, domain containing 3 | LYSMD3 | AW080618 | Hs.136235 | chr5q14.3 |
| 226320_at | THO complex 4 | THOC4 | AF047002 | Hs.446673 | chr17q25.3 |
| 226318_at | transforming growth factor beta regulator 1 | TBRG1 | AI701055 | Hs.443668 | chr11q24.2 |
| 226314_at | dermatan 4 sulfotransferase 1 | D4ST1 | AA039350 | Hs.442449 | chr15q15.1 |
| 226310_at | rapamycin-insensitive companion of mTOR | RICTOR | AI743511 | Hs.9343 | chr5p13.1 |
| 226306_at | chromosome 6 open reading frame 1 | C6orf1 | BF984592 | Hs.381300 | chr6p21.31 |
| 226303_at | phosphoglucomutase 5 | PGM5 | AA706788 | Hs.46531 | chr9p12-q12 |
| 226302_at | ATPase, Class I, type 8B, member 1 | ATP8B1 | BG290908 |  |  |
| 226301_at | chromosome 6 open reading frame 192 | C6orf192 | AV729072 | Hs.347144 | chr6q22.3-q23.3 |
| 226297_at | Homeodomain interacting protein kinase 3 | HIPK3 | AV693403 | Hs.30148 | chr11p13 |
| 226294_x_at | family with sequence similarity 91, member A1 | FAM91A1 | AI245517 | Hs.412846 | chr8q24.13 |
| 226293_at | mediator of RNA polymerase II transcription, subunit 19 homolog (S. cerevisiae) | MED19 | BE781103 | Hs.43619 | chr11q12.1 |
| 226291_at | amyotrophic lateral sclerosis 2 (juvenile) | ALS2 | AB046783 | Hs.27669 | chr2q33.1 |
| 226290_at | B double prime 1, subunit of RNA polymerase III transcription initiation factor IIIB | BDP1 | AW051603 |  |  |
| 226288_s_at | neuroligin 2 | NLGN2 | AI951319 | Hs.26229 | chr17p13.1 |
| 226287_at | coiled-coil domain containing 34 | CCDC34 | AI458313 | Hs.335951 | chr11p14.1 |
| 226284_at | zinc finger and BTB domain containing 2 | ZBTB2 | BF111616 | Hs.24106 | chr6q25.1 |
| 226283_at | WD repeat domain 51B | WDR51B | BF131947 | Hs.27804 | chr12q21.33 |
| 226281_at | delta/notch-like EGF repeat containing | DNER | BF059512 | Hs.234074 | chr2q36.3 |
| 226275_at | MAX dimerization protein 1 | MXD1 | AI188653 | Hs.379930 | chr2p13-p12 |
| 226274_at | chloride channel 5 (nephrolithiasis 2, X-linked, Dent disease) | CLCN5 | AK025562 |  | chrxp11.23 |
| 226269_at | ganglioside-induced differentiation-associated protein 1 | GDAP1 | BF002104 | Hs.168950 | chr8q21.11 |
| 226268_at | RAB21, member RAS oncogene family | RAB21 | AI309554 | Hs.202225 | chr12q21.1 |
| 226267_at | jun dimerization protein 2 | JDP2 | AA716425 | Hs.154095 | chr14q24.3 |
| 226264_at | sushi domain containing 1 | SUSD1 | AL137432 | Hs.28564 | chr9q31.3-q33.1 |
| 226263_at | chromosome 6 open reading frame 151 | C6orf151 | AA630674 | Hs.13366 | chr6p24.3 |
| 226261_at | zinc and ring finger 2 | ZNRF2 | AI831561 | Hs.127294 | chr7p15.1 |
| 226255_at | zinc finger and BTB domain containing 33 | ZBTB33 | BE302089 | Hs.143604 | chrxq23 |
| 226254_s_at | KIAA1430 | KIAA1430 | AI912523 | Hs.126914 | chr4q35.1 |
| 226249_at | sorting nexin family member 30 | SNX30 | AI674625 | Hs.356340 | chr9q32 |
| 226247_at | pleckstrin homology domain containing, family A (phosphoinositide binding specific) member 1 | PLEKHA1 | AI346026 | Hs.287830 | chr10q26.13 |
| 226243_at | similar to CG14903-PA | LOC391356 | BF590958 | Hs.406607 | chr2p23.3 |
| 226242_at | chromosome 1 open reading frame 131 | C1orf131 | BF515750 | Hs.434945 | chr1q42.2 |
| 226241_s_at | mitochondrial ribosomal protein L52 | MRPL52 | BG497211 | Hs.355935 | chr14q11.2 |
| 226240_at | transcriptional adaptor 2 (ADA2 homolog, yeast)-beta | MGC21874 | AI862537 | Hs.467614 | chr4p16.1 |
| 226239_at | transmembrane protein 150 | TMEM150 | AL573722 | Hs.356187 | chr2p11.2 |
| 226238_at | methylmalonyl CoA epimerase | MCEE | AI934339 | Hs.94949 | chr2p13.3 |
| 226236_at | hypothetical gene supported by AF147354 | LOC388789 | BF675218 | Hs.349092 | chr20p11.23 |
| 226233_at | beta-1,3-N-acetylgalactosaminyltransferase 2 | B3GALNT2 | BG112197 |  |  |
| 226225_at | mutated in colorectal cancers | MCC | BE967311 |  |  |
| 226224_at | forkhead box K2 | FOXK2 | AI798846 | Hs.458404 | chr17q25 |
| 226221_at | KIAA1432 | KIAA1432 | AL138104 | Hs.279556 | chr9p24.1 |
| 226215_s_at | F-box and leucine-rich repeat protein 10 | FBXL10 | AI989996 | Hs.405932 | chr12q24.31 |
| 226211_at | maternally expressed 3 | MEG3 | AI291123 | Hs.418271 | chr14q32 |
| 226209_at | NADH dehydrogenase (ubiquinone) flavoprotein 3, 10kDa | NDUFV3 | AI129346 | Hs.199471 | chr21q22.3 |
| 226208_at | zinc finger, SWIM-type containing 6 | ZSWIM6 | AI692595 | Hs.449290 | chr5q12.1 |
| 226207_at | hypothetical protein FLJ39378 | FLJ39378 | AI358954 | Hs.427552 | chr12q24.31 |
| 226206_at | v-maf musculoaponeurotic fibrosarcoma oncogene homolog K (avian) | MAFK | BG231691 | Hs.11607 | chr7p22.3 |
| 226201_at | Splicing factor 3a, subunit 2, 66kDa | SF3A2 | AI224128 | Hs.512626 | chr19p13.3 |
| 226200_at | valyl-tRNA synthetase like | VARSL | BE222664 | Hs.534546 | chr6p21 |
| 226199_at | uracil phosphoribosyltransferase (FUR1) homolog (S. cerevisiae) | UPRT | AL563795 | Hs.91612 | chrxq13.3 |
| 226198_at | target of myb1-like 2 (chicken) | TOM1L2 | AV751731 | Hs.375628 | chr17p11.2 |
| 226195_at | chromosome 14 open reading frame 179 | C14orf179 | AW827281 | Hs.446344 | chr14q24.3 |
| 226194_at | chromosome 13 open reading frame 8 | C13orf8 | BC004820 | Hs.7542 | chr13q34 |
| 226189_at | integrin, beta 8 | ITGB8 | BF513121 | Hs.367688 |  |
| 226181_at | tubulin, epsilon 1 | TUBE1 | AI613127 | Hs.34851 | chr6q21 |
| 226180_at | WD repeat domain 36 | WDR36 | BF439595 | Hs.175596 | chr5q22.1 |
| 226178_at | suppressor of cytokine signaling 4 | SOCS4 | BF446961 | Hs.349397 | chr14q22.2 |
| 226177_at | glycolipid transfer protein | GLTP | AI052020 | Hs.381256 | chr12q24.11 |
| 226176_s_at | ubiquitin specific peptidase 42 | USP42 | BE048999 | Hs.31856 | chr7p22.1 |
| 226175_at | tetratricopeptide repeat domain 9C | TTC9C | AI890604 | Hs.31704 | chr11q12.3 |
| 226171_at | hypothetical protein FLJ20209 | FLJ20209 | BF111925 | Hs.528655 | chr3p21.31 |
| 226168_at | zinc finger, AN1-type domain 2B | ZFAND2B | AA524690 | Hs.534644 | chr2q35 |
| 226163_at | zinc finger and BTB domain containing 9 | ZBTB9 | AW291499 | Hs.380417 | chr6p21.32 |
| 226160_at | hexose-6-phosphate dehydrogenase (glucose 1-dehydrogenase) | H6PD | AW138757 | Hs.194728 | chr1p36 |
| 226159_at | hypothetical protein LOC285636 | LOC285636 | N31982 | Hs.29899 | chr5p13.1 |
| 226155_at | KIAA1600 | KIAA1600 | AB046820 | Hs.192619 | chr10q25.3 |
| 226152_at | tetratricopeptide repeat domain 7B | TTC7B | BE963437 | Hs.400383 | chr14q32.12 |
| 226146_at | Hypothetical protein LOC255458 | LOC255458 | BE503186 | Hs.348292 | chr5q35.3 |
| 226143_at | retinoic acid induced 1 | RAI1 | BF984830 | Hs.438904 | chr17p11.2 |
| 226140_s_at | OTU domain containing 1 | OTUD1 | AI934347 | Hs.102647 | chr10p12.31 |
| 226136_at | GLI pathogenesis-related 1 (glioma) | GLIPR1 | N32834 | Hs.511765 | chr12q21.1 |
| 226135_at | chromosome 6 open reading frame 107 | C6orf107 | BE856657 | Hs.534519 | chr6p21 |
| 226133_s_at | TBC1 domain family, member 10A | TBC1D10A | AW628835 | Hs.505579 | chr22q12.1-qter |
| 226129_at | family with sequence similarity 83, member H | FAM83H | AI949095 | Hs.67776 | chr8q24.3 |
| 226128_at | Chromosome 1 open reading frame 58 | C1orf58 | N73682 | Hs.6236 | chr1q41 |
| 226126_at | hypothetical protein MGC16169 | MGC16169 | AA702160 | Hs.433215 | chr4q24 |
| 226124_at | zinc finger protein 90 homolog (mouse) | ZFP90 | AA747309 | Hs.434928 | chr16q22.1 |
| 226122_at | pleckstrin homology domain containing, family G (with RhoGef domain) member 1 | PLEKHG1 | AL035086 | Hs.51965 | chr6q25.1 |
| 226120_at | tetratricopeptide repeat domain 8 | TTC8 | AW293939 | Hs.55158 | chr14q31.3 |
| 226117_at | TRAF-interacting protein with a forkhead-associated domain | TIFA | AA195074 | Hs.310640 | chr4q25 |
| 226113_at | zinc finger protein 436 | ZNF436 | AB051497 | Hs.293798 | chr1p36 |
| 226108_at | conserved nuclear protein NHN1 | NHN1 | AK026317 | Hs.93670 | chr16q24.2-q24.3 |
| 226103_at | nexilin (F actin binding protein) | NEXN | AF114264 | Hs.22370 | chr1p31.1 |
| 226095_s_at | hypothetical protein LOC146517 | LOC146517 | AW138861 | Hs.451351 | chr16q22.2 |
| 226093_at | DCP1 decapping enzyme homolog B (S. cerevisiae) | DCP1B | AW204088 | Hs.255996 | chr12p13.33 |
| 226091_s_at | Mof4 family associated protein 1 | MRFAP1 | BG435643 | Hs.406590 | chr4p16.1 |
| 226083_at | transmembrane protein 70 | TMEM70 | AA886902 |  |  |
| 226081_at | Leucine zipper and CTNNBIP1 domain containing | LZIC | BE219717 | Hs.327252 | chr1p36.22 |
| 226080_at | slingshot homolog 2 (Drosophila) | SSH2 | BE676214 | Hs.126798 | chr17q11.2 |
| 226077_at | hypothetical protein FLJ31951 | FLJ31951 | AL553942 | Hs.349306 | chr5q33.3 |
| 226076_s_at | methyl-CpG binding domain protein 6 | MBD6 | AI174548 | Hs.348428 | chr12q13.2 |
| 226074_at | protein phosphatase 1M (PP2C domain containing) | PPM1M | N22918 | Hs.373560 | chr3p21.2 |
| 226073_at | hypothetical protein LOC219854 | LOC219854 | BE857362 | Hs.7626 | chr11q24.2 |
| 226063_at | vav 2 oncogene | VAV2 | AA481141 | Hs.4248 | chr9q34.1 |
| 226062_x_at | family with sequence similarity 63, member A | FAM63A | AB037811 | Hs.3346 | chr1q21.2 |
| 226060_at | RFT1 homolog (S. cerevisiae) | RFT1 | BF475369 | Hs.156512 | chr3p21.1 |
| 226059_at | translocase of outer mitochondrial membrane 40 homolog-like (yeast) | TOMM40L | AK022832 | Hs.321653 | chr1q23.3 |
| 226058_at | hypothetical protein MGC4655 | MGC4655 | AI651445 | Hs.15403 | chr16q22.1 |
| 226056_at | Cdc42 GTPase-activating protein | CDGAP | AB033030 | Hs.212169 | chr3q13.32-q13.33 |
| 226055_at | arrestin domain containing 2 | ARRDC2 | AK000689 | Hs.30882 | chr19p13.11 |
| 226051_at | selenoprotein M | SELM | BF973568 | Hs.55940 | chr22q12.2 |
| 226048_at | mitogen-activated protein kinase 8 | MAPK8 | N92719 | Hs.445864 | chr10q11.22 |
| 226045_at | fibroblast growth factor receptor substrate 2 | FRS2 | AW264515 | Hs.202404 | chr12q15 |
| 226043_at | G-protein signalling modulator 1 (AGS3-like, C. elegans) | GPSM1 | AI242661 | Hs.239370 | chr9q34.3 |
| 226038_at | LON peptidase N-terminal domain and ring finger 1 | LONRF1 | BF680438 | Hs.180178 | chr8p23.1 |
| 226037_s_at | TAF9B RNA polymerase II, TATA box binding protein (TBP)-associated factor, 31kDa /// similar to transcription associated factor 9B | TAF9B | AL049589 | Hs.534514 | chrxq13.1-q21.1 |
| 226031_at | coiled-coil domain containing 132 | CCDC132 | AA523733 | Hs.222282 | chr7q21.3 |
| 226029_at | vang-like 2 (van gogh, Drosophila) | VANGL2 | AB033041 | Hs.137507 | chr1q22-q23 |
| 226026_at | disrupted in renal carcinoma 2 | DIRC2 | AI147467 | Hs.11360 | chr3q21.1 |
| 226020_s_at | disabled homolog 1 (Drosophila) /// OMA1 homolog, zinc metallopeptidase (S. cerevisiae) | DAB1 | AI927931 | Hs.477370 | chr1p32-p31 /// chr1p32.2-p32.1 |
| 226019_at | OMA1 homolog, zinc metallopeptidase (S. cerevisiae) | OMA1 | AI927931 | Hs.8108 | chr1p32.2-p32.1 |
| 226018_at | chromosome 7 open reading frame 41 | C7orf41 | W73230 | Hs.7913 | chr7p15.1 |
| 226017_at | CKLF-like MARVEL transmembrane domain containing 7 | CMTM7 | AI708432 | Hs.440494 | chr3p23 |
| 226012_at | ankyrin repeat domain 11 | ANKRD11 | AI686514 | Hs.402727 | chr16q24.3 |
| 226011_at | coiled-coil domain containing 12 | CCDC12 | BG107767 | Hs.425365 | chr3p21.31 |
| 226010_at | solute carrier family 25 (mitochondrial carrier; phosphate carrier), member 23 | SLC25A23 | BG290819 | Hs.356231 | chr19p13.3 |
| 226008_at | necdin-like 2 | NDNL2 | AA627644 | Hs.94011 | chr15q13.1 |
| 226007_at | HesB like domain containing 1 | HBLD1 | AV715993 | Hs.291079 | chr14q24.3 |
| 226006_at | hypothetical gene LOC554363 | LOC554363 | AV758342 |  |  |
| 226005_at | ubiquitin-conjugating enzyme E2G 1 (UBC7 homolog, yeast) | UBE2G1 | BG170762 | Hs.78563 | chr1q42 |
| 226001_at | kelch-like 5 (Drosophila) | KLHL5 | AK002174 | Hs.272251 | chr4p14 |
| 225998_at | GRB2-associated binding protein 1 | GAB1 | AK022142 | Hs.80720 | chr4q31.21 |
| 225997_at | MOB1, Mps One Binder kinase activator-like 1A (yeast) | MOBKL1A | AL162039 | Hs.31422 | chr4q13.3 |
| 225996_at | LON peptidase N-terminal domain and ring finger 2 | LONRF2 | AV709727 | Hs.21380 |  |
| 225995_x_at | family with sequence similarity 39, member B | FAM39B | BG178695 | Hs.363274 | chr2q14.1 |
| 225985_at | protein kinase, AMP-activated, alpha 1 catalytic subunit | PRKAA1 | AI935917 | Hs.43322 | chr5p12 |
| 225980_at | chromosome 14 open reading frame 43 | C14orf43 | AV740879 | Hs.130323 | chr14q24.3 |
| 225976_at | basic transcription factor 3-like 4 | BTF3L4 | AL568049 | Hs.429839 | chr1p32.3 |
| 225973_at | transporter 2, ATP-binding cassette, sub-family B (MDR/TAP) | TAP2 | AA573502 | Hs.502 | chr6p21.3 |
| 225972_at | transmembrane protein 64 | TMEM64 | AA524005 | Hs.129276 | chr8q21.3 |
| 225969_at | alkB, alkylation repair homolog 6 (E. coli) | ALKBH6 | AW105337 | Hs.71941 | chr19q13.12 |
| 225968_at | prickle homolog 2 (Drosophila) | PRICKLE2 | BG285881 | Hs.367669 | chr3p14.1 |
| 225967_s_at | hypothetical LOC284184 | LOC284184 | BF683512 | Hs.356545 | chr17q25.3 |
| 225965_at | DDHD domain containing 1 | DDHD1 | H16810 | Hs.88558 | chr14q21 |
| 225963_at | kelch domain containing 5 | KLHDC5 | AW293538 | Hs.284270 | chr12p11.22 |
| 225962_at | zinc and ring finger 1 | ZNRF1 | AI199541 | Hs.320834 | chr16q22.3 |
| 225958_at | polyhomeotic homolog 1 (Drosophila) | PHC1 | AI554106 | Hs.305985 | chr12p13 |
| 225955_at | mediator of RNA polymerase II transcription, subunit 25 homolog (S. cerevisiae) /// meteorin, glial cell differentiation regulator-like /// similar to meteorin, glial cell differentiation regulator-like | MED25 | BG231494 | Hs.514615 | chr17q25.3 |
| 225954_s_at | midnolin | MIDN | AL512725 | Hs.302969 | chr19p13.3 |
| 225949_at | nuclear receptor binding protein 2 | NRBP2 | N21030 | Hs.274401 | chr8q24.3 |
| 225948_at | chromosome 14 open reading frame 153 | C14orf153 | BF197057 | Hs.116029 | chr14q32.32-q32.33 |
| 225947_at | myosin head domain containing 1 | MYOHD1 | AA994178 | Hs.302051 | chr17q12 |
| 225943_at | neurolysin (metallopeptidase M3 family) | NLN | BF222737 | Hs.22151 | chr5q12.3 |
| 225939_at | eukaryotic translation initiation factor 4E family member 3 | EIF4E3 | AL161983 | Hs.476782 | chr3p14 |
| 225936_at | EP300 interacting inhibitor of differentiation 2 | EID2 | BE747815 | Hs.18949 | chr19q13.2 |
| 225933_at | similar to RIKEN cDNA 3110023B02 | MGC16597 | BF115231 | Hs.449864 | chr17q25.3 |
| 225930_at | NFKB inhibitor interacting Ras-like 1 | NKIRAS1 | AI970120 | Hs.173202 | chr3p24.2 |
| 225929_s_at | chromosome 17 open reading frame 27 | C17orf27 | AA233374 | Hs.195642 | chr17q25.3 |
| 225927_at | mitogen-activated protein kinase kinase kinase 1 | MAP3K1 | AA541479 | Hs.170610 | chr5q11.2 |
| 225926_at | vesicle transport through interaction with t-SNAREs homolog 1B (yeast) | VTI1B | AI307763 |  |  |
| 225922_at | KIAA1450 protein | KIAA1450 | BE501838 | Hs.377588 | chr4q32.1 |
| 225921_at | ninein (GSK3B interacting protein) | NIN | AL359571 | Hs.385985 | chr14q22.1 |
| 225920_at | hypothetical protein LOC148413 | LOC148413 | AW452640 | Hs.432100 | chr1p36.33 |
| 225919_s_at | chromosome 9 open reading frame 72 | C9orf72 | AI832598 | Hs.23607 | chr9p21.2 |
| 225918_at | hypothetical protein LOC146346 | LOC146346 | AI742940 | Hs.109731 | chr16q22.3 |
| 225916_at | zinc finger protein 131 | ZNF131 | AA789302 | Hs.97845 | chr5p12-p11 |
| 225914_s_at | calcium binding protein 39-like | CAB39L | AW242839 | Hs.87159 | chr13q14.2 |
| 225913_at | KIAA2002 protein | KIAA2002 | AK025943 | Hs.9587 | chr15q24.3 |
| 225912_at | tumor protein p53 inducible nuclear protein 1 | TP53INP1 | AW341649 | Hs.75497 | chr8q22 |
| 225908_at | isoamyl acetate-hydrolyzing esterase 1 homolog (S. cerevisiae) | IAH1 | AI829927 | Hs.535771 | chr2p25.2 |
| 225904_at | chromosome 1 open reading frame 96 | C1orf96 | N64686 | Hs.497984 | chr1q42.13 |
| 225903_at | phosphatidylinositol glycan anchor biosynthesis, class U | PIGU | AL118520 | Hs.62918 | chr20q11.22 |
| 225899_x_at | FLJ45445 protein /// hypothetical gene supported by AK127273 /// hypothetical LOC653340 /// similar to FLJ45445 protein /// similar to FLJ45445 protein /// hypothetical protein LOC728797 /// hypothetical protein LOC729660 | FLJ45445 | AL040396 | Hs.356758 | chr19p13.3 |
| 225898_at | WD repeat domain 54 | WDR54 | AK023015 | Hs.323537 | chr2p13.1 |
| 225896_at | Myosin phosphatase-Rho interacting protein | M-RIP | AI656836 | Hs.270299 | chr17p11.2 |
| 225892_at | Iron-responsive element binding protein 2 | IREB2 | BF438417 |  |  |
| 225890_at | chromosome 20 open reading frame 72 | C20orf72 | AI678096 | Hs.320823 | chr20p11.23 |
| 225889_at | AE binding protein 2 | AEBP2 | BF475280 | Hs.528315 | chr12p12.3 |
| 225884_s_at | GDNF-inducible zinc finger protein 1 | GZF1 | AL046381 | Hs.28921 | chr20p12.3-p11.21 |
| 225882_at | solute carrier family 35, member B4 | SLC35B4 | AI948585 | Hs.292566 | chr7q33 |
| 225876_at | NIPA-like domain containing 3 | NPAL3 | T84558 | Hs.389516 | chr1p36.12-p35.1 |
| 225872_at | solute carrier family 35, member F5 | SLC35F5 | AA044835 | Hs.437608 | chr2q14.1 |
| 225871_at | six transmembrane epithelial antigen of the prostate 2 | STEAP2 | BF680588 | Hs.408200 | chr7q21 |
| 225870_s_at | trafficking protein particle complex 5 | TRAPPC5 | BF569208 | Hs.432413 | chr19p13.2 |
| 225867_at | vasorin | VASN | BE741869 | Hs.436584 | chr16p13.3 |
| 225866_at | brix domain containing 1 | BXDC1 | AA976536 | Hs.372265 | chr6q21 |
| 225862_at | solute carrier family 25, member 26 | SLC25A26 | AA928255 | Hs.379386 | chr3p14.1 |
| 225860_at | hypothetical protein LOC729580 /// hypothetical protein LOC730672 | LOC729580 | BG469257 | Hs.212581 | chr20q11.22 |
| 225855_at | erythrocyte membrane protein band 4.1 like 5 | EPB41L5 | AB046768 | Hs.129368 | chr2q14.2 |
| 225853_at | glucosamine-phosphate N-acetyltransferase 1 | GNPNAT1 | BE789346 | Hs.478025 | chr14q22.1 |
| 225852_at | ankyrin repeat domain 17 | ANKRD17 | BE463523 | Hs.131059 | chr4q13.3 |
| 225851_at | farnesyltransferase, CAAX box, beta | FNTB | BF131248 | Hs.276 | chr14q23-q24 |
| 225849_s_at | SFT2 domain containing 1 | SFT2D1 | BF975327 | Hs.284265 | chr6q27 |
| 225848_at | zinc finger protein 746 | ZNF746 | AI300168 | Hs.24643 | chr7q36.1 |
| 225847_at | arylacetamide deacetylase-like 1 | AADACL1 | AB037784 | Hs.22941 | chr3q26.31 |
| 225844_at | polymerase (DNA-directed), epsilon 4 (p12 subunit) | POLE4 | NM_019896 | Hs.19980 | chr2p12 |
| 225841_at | chromosome 1 open reading frame 59 | C1orf59 | BE502436 | Hs.7962 | chr1p13.3 |
| 225840_at | thyrotrophic embryonic factor | TEF | AA779795 | Hs.181159 | chr22q13.2 |
| 225838_at | enhancer of polycomb homolog 2 (Drosophila) | EPC2 | AK002010 | Hs.23270 | chr2q23.1 |
| 225836_s_at | chromosome 12 open reading frame 32 | C12orf32 | AL528840 | Hs.157148 | chr12p13.33 |
| 225834_at | family with sequence similarity 72, member A /// similar to family with sequence similarity 72, member A /// similar to family with sequence similarity 72, member A | FAM72A | AL135396 | Hs.534827 |  |
| 225833_at | diacylglycerol lipase beta | DAGLBETA | BE795104 | Hs.131899 | chr7p22.1 |
| 225831_at | leucine zipper protein 1 | LUZP1 | AW016830 | Hs.396497 | chr1p36 |
| 225830_at | PDZ domain containing 8 | PDZD8 | AI052247 | Hs.501149 | chr10q26.11 |
| 225826_at | methylmalonic aciduria (cobalamin deficiency) cblB type | MMAB | AV702487 | Hs.12106 | chr12q24 |
| 225825_at | chromosome 20 open reading frame 194 | C20orf194 | AL110249 | Hs.119021 | chr20p13 |
| 225823_at | hypothetical protein P117 | P117 | AA699669 | Hs.356626 | chr19p13.3 |
| 225812_at | hypothetical protein LOC619208 | LOC619208 | N36759 | Hs.328097 |  |
| 225810_at | myotubularin related protein 10 | MTMR10 | AL572015 | Hs.126721 | chr15q13.3 |
| 225808_at | hypothetical protein LOC124512 | LOC124512 | AA883486 | Hs.74655 | chr17q25.1 |
| 225807_at | jub, ajuba homolog (Xenopus laevis) | JUB | AK025567 | Hs.434997 | chr14q11.2 |
| 225804_at | cytochrome b5 domain containing 2 | CYB5D2 | BE044480 | Hs.284163 | chr17p13.2 |
| 225802_at | topoisomerase (DNA) I, mitochondrial | TOP1MT | AW592604 | Hs.348605 | chr8q24.3 |
| 225800_at | JAZF zinc finger 1 | JAZF1 | AI990891 | Hs.368944 | chr7p15.2-p15.1 |
| 225799_at | hypothetical protein MGC4677 | MGC4677 | BF209337 | Hs.432419 | chr2p11.2 |
| 225797_at | mitochondrial ribosomal protein L54 | MRPL54 | AV707568 | Hs.356578 | chr19p13.3 |
| 225794_s_at | chromosome 22 open reading frame 32 | C22orf32 | AV751709 | Hs.306083 | chr22q13.2 |
| 225793_at | Lix1 homolog (mouse)-like | LIX1L | AW500180 | Hs.356109 | chr1q21.1 |
| 225790_at | methionine sulfoxide reductase B3 | MSRB3 | AL048386 | Hs.339024 | chr12q14.3 |
| 225789_at | centaurin, gamma 3 | CENTG3 | BE876194 | Hs.249728 | chr7q36.1 |
| 225788_at | chromosome 6 open reading frame 153 | C6orf153 | AF161371 | Hs.309231 | chr6p21.1 |
| 225779_at | solute carrier family 27 (fatty acid transporter), member 4 | SLC27A4 | AK000722 | Hs.415702 | chr9q34.11 |
| 225772_s_at | chromosome 12 open reading frame 62 | C12orf62 | BF203664 | Hs.388645 | chr12q13.12 |
| 225770_at | ring finger and SPRY domain containing 1 | RSPRY1 | AI206557 | Hs.181161 | chr16q13 |
| 225769_at | component of oligomeric golgi complex 6 | COG6 | AF116827 | Hs.435994 | chr13q14.11 |
| 225762_x_at | hypothetical protein LOC284801 | LOC284801 | AL531683 | Hs.370699 | chr20p11.1 |
| 225760_at | myb-like, SWIRM and MPN domains 1 | MYSM1 | AI302244 | Hs.437866 | chr1p32.1 |
| 225755_at | kelch domain containing 8B | KLHDC8B | AI160540 | Hs.13781 | chr3p21.31 |
| 225754_at | adaptor-related protein complex 1, gamma 1 subunit | AP1G1 | AA142984 | Hs.5344 | chr16q23 |
| 225753_at | zinc finger protein 513 | ZNF513 | AW003280 | Hs.440595 | chr2p23.3 |
| 225751_at | RNA binding motif protein 17 | RBM17 | BF063156 | Hs.107001 | chr10p15.1 |
| 225749_at | Hypothetical protein LOC283951 | LOC283951 | BE889319 | Hs.58373 | chr16p13.3 |
| 225748_at | LTV1 homolog (S. cerevisiae) | LTV1 | AI458051 | Hs.33944 | chr6q24.2 |
| 225744_at | zinc finger, DHHC-type containing 8 | ZDHHC8 | AB033118 | Hs.63128 | chr22q11.21 |
| 225738_at | Rap guanine nucleotide exchange factor (GEF) 1 | RAPGEF1 | AA761259 | Hs.130969 | chr9q34.3 |
| 225737_s_at | F-box protein 22 | FBXO22 | BE966247 | Hs.438891 | chr15q23 |
| 225735_at | ankyrin repeat domain 50 | ANKRD50 | AL110131 | Hs.28783 | chr4q28.1 |
| 225733_at | UDP-Gal:betaGal beta 1,3-galactosyltransferase polypeptide 6 | B3GALT6 | AI890557 | Hs.284284 | chr1p36.33 |
| 225727_at | pleckstrin homology domain containing, family H (with MyTH4 domain) member 1 | PLEKHH1 | AB033026 | Hs.509715 | chr14q24.1 |
| 225723_at | chromosome 6 open reading frame 129 | C6orf129 | BE794699 | Hs.284207 | chr6p21.2 |
| 225722_at | Family with sequence similarity 78, member B | FAM78B | BF246937 | Hs.205616 /// Hs.493518 | chr1q24.1 |
| 225721_at | synaptopodin 2 | SYNPO2 | AI658662 | Hs.24192 | chr4q26 |
| 225719_s_at | mitochondrial ribosomal protein L55 | MRPL55 | BG497783 | Hs.63236 | chr1q42.13 |
| 225717_at | KIAA1715 | KIAA1715 | AI814587 | Hs.209561 | chr2q31 |
| 225713_at | serine/threonine kinase 11 interacting protein | STK11IP | AV727346 | Hs.22410 | chr2q35 |
| 225712_at | gem (nuclear organelle) associated protein 5 | GEMIN5 | AW024563 |  |  |
| 225711_at | ADP-ribosylation-like factor 6 interacting protein 6 | ARL6IP6 | AA654338 | Hs.141347 | chr2q23.3 |
| 225708_at | intersex-like (Drosophila) | IXL | AI561202 | Hs.164649 | chr19q13.2 |
| 225705_at | coiled-coil domain containing 45 | CCDC45 | AI697340 | Hs.528712 | chr17q24.2 |
| 225702_at | chromosome 8 open reading frame 76 | C8orf76 | AA973041 | Hs.334824 | chr8q24.13 |
| 225701_at | AT-hook transcription factor | AKNA | AK024431 | Hs.159578 | chr9q32 |
| 225699_at | chromosome 7 open reading frame 40 | C7orf40 | AI937446 | Hs.25892 | chr7p13 |
| 225698_at | chromosome 5 open reading frame 26 | C5orf26 | BF314746 | Hs.12082 | chr5q21-q22 |
| 225696_at | COP9 constitutive photomorphogenic homolog subunit 7B (Arabidopsis) | COPS7B | AK024273 | Hs.335061 | chr2q37.1 |
| 225695_at | chromosome 2 open reading frame 18 | C2orf18 | BG497776 | Hs.11759 | chr2p23.3 |
| 225690_at | Cdc2-related kinase, arginine/serine-rich | CRKRS | AW051345 | Hs.416108 | chr17q12 |
| 225689_at | chromosome 3 open reading frame 39 | C3orf39 | BE856822 | Hs.12313 | chr3p22.1 |
| 225688_s_at | pleckstrin homology-like domain, family B, member 2 | PHLDB2 | AK025444 | Hs.7378 | chr3q13.2 |
| 225686_at | family with sequence similarity 33, member A | FAM33A | BE048371 | Hs.69476 | chr17q23.2 |
| 225683_x_at | phosphohistidine phosphatase 1 | PHPT1 | AA514384 | Hs.409834 | chr9q34.3 |
| 225681_at | collagen triple helix repeat containing 1 | CTHRC1 | AA584310 | Hs.283713 | chr8q22.3 |
| 225680_at | hypothetical protein DKFZp434K1815 | DKFZp434K1815 | BE896303 | Hs.274135 | chr7q22.1 |
| 225679_at | N-acetyltransferase 12 | NAT12 | AA534210 | Hs.285280 | chr14q22.3 |
| 225678_at | polymerase (RNA) III (DNA directed) polypeptide H (22.9kD) | POLR3H | AB051452 |  |  |
| 225676_s_at | WD repeats and SOF1 domain containing | WDSOF1 | BE409290 | Hs.273344 | chr8q22.3 |
| 225675_at | chromosome 14 open reading frame 101 | C14orf101 | AW976269 | Hs.509426 | chr14q22.3 |
| 225672_at | golgi autoantigen, golgin subfamily a, 2 | GOLGA2 | AL514295 | Hs.24049 | chr9q34.11 |
| 225670_at | hypothetical protein LOC134145 | LOC134145 | AI384017 | Hs.409027 | chr5p15.2 |
| 225669_at | interferon (alpha, beta and omega) receptor 1 | IFNAR1 | AA133989 | Hs.181315 | chr21q22.11 |
| 225666_at | transmembrane and tetratricopeptide repeat containing 4 | TMTC4 | D80480 | Hs.172963 | chr13q32.3 |
| 225659_at | hypothetical protein LOC339745 | LOC339745 | BF516590 | Hs.185018 | chr2q22.1 |
| 225657_at | hypothetical protein BC007882 | LOC152217 | BE563042 | Hs.118820 | chr3q29 |
| 225656_at | EF-hand domain (C-terminal) containing 1 | EFHC1 | AI564473 | Hs.446047 | chr6p12.3 |
| 225654_at | hypothetical protein LOC202347 | LOC202347 | AI768720 | Hs.106861 | chr5q35.3 |
| 225651_at | ubiquitin-conjugating enzyme E2E 2 (UBC4/5 homolog, yeast) | UBE2E2 | BF431962 | Hs.444289 | chr3p24.2 |
| 225648_at | serine/threonine kinase 35 | STK35 | AA001414 | Hs.100057 | chr20p13 |
| 225646_at | cathepsin C | CTSC | AI246687 | Hs.128065 | chr11q14.1-q14.3 |
| 225644_at | coiled-coil domain containing 117 | CCDC117 | BF060776 | Hs.406460 | chr22q12.1 |
| 225642_at | KTI12 homolog, chromatin associated (S. cerevisiae) | KTI12 | AL581536 | Hs.534620 | chr1p32.3 |
| 225638_at | chromosome 1 open reading frame 31 | C1orf31 | AI564054 | Hs.5250 | chr1q42.2 |
| 225636_at | signal transducer and activator of transcription 2, 113kDa | STAT2 | H98105 | Hs.72988 | chr12q13.3 |
| 225635_s_at | Hypothetical gene supported by AK091718 | LOC401504 | BG535378 | Hs.446271 | chr9p13.2 |
| 225633_at | dpy-19-like 3 (C. elegans) | DPY19L3 | BF057717 | Hs.194392 | chr19q13.11 |
| 225627_s_at | cache domain containing 1 | CACHD1 | AK024256 | Hs.24790 | chr1p31.3 |
| 225624_at | similar to RIKEN cDNA 4933437K13 | LOC92017 | AL044019 | Hs.145047 | chr16p13.13-p13.12 |
| 225623_at | KIAA1737 | KIAA1737 | AI621225 | Hs.22452 | chr14q24.3 |
| 225621_at | asparagine-linked glycosylation 2 homolog (S. cerevisiae, alpha-1,3-mannosyltransferase) | ALG2 | BE967331 | Hs.40919 | chr9q22.33 |
| 225620_at | RAB35, member RAS oncogene family | RAB35 | AL534848 | Hs.94308 | chr12q24.31 |
| 225619_at | SLAIN motif family, member 1 | SLAIN1 | AV730849 | Hs.349955 | chr13q22.3 |
| 225617_at | outer dense fiber of sperm tails 2 | ODF2 | AL138382 | Hs.129055 | chr9q34.11 |
| 225616_at | SPRY domain containing 4 | SPRYD4 | AI570493 | Hs.128676 | chr12q13.3 |
| 225614_at | serum amyloid A-like 1 | SAAL1 | AI815207 | Hs.183733 | chr11p15.1 |
| 225613_at | microtubule associated serine/threonine kinase family member 4 | MAST4 | AI096389 | Hs.212787 | chr5q12.3 |
| 225612_s_at | UDP-GlcNAc:betaGal beta-1,3-N-acetylglucosaminyltransferase 5 | B3GNT5 | BE672260 | Hs.257222 | chr3q28 |
| 225610_at | ubiquitin-like, containing PHD and RING finger domains, 2 | UHRF2 | BF511410 | Hs.394916 | chr9p24.1 |
| 225607_at | coiled-coil domain containing 43 | CCDC43 | AI948491 | Hs.378913 | chr17q21.31 |
| 225606_at | BCL2-like 11 (apoptosis facilitator) | BCL2L11 | AI949179 | Hs.84063 | chr2q13 |
| 225605_at | tumor protein p53 inducible protein 13 | TP53I13 | AL540867 | Hs.445264 | chr17q11.2 |
| 225604_s_at | chromosome 9 open reading frame 19 | C9orf19 | AA284532 | Hs.302766 | chr9p13-p12 |
| 225600_at | PRO0845 | DKFZP779L1068 | AW303300 | Hs.440643 | chr8q22.1 |
| 225593_at | LSM10, U7 small nuclear RNA associated | LSM10 | AL542359 | Hs.3496 | chr1p34.3 |
| 225592_at | nurim (nuclear envelope membrane protein) | NRM | D81048 | Hs.57222 | chr6p21.33 |
| 225589_at | SH3 domain containing ring finger 1 | SH3RF1 | AB040927 | Hs.301804 | chr4q32.3-q33 |
| 225587_at | transmembrane protein 129 | TMEM129 | BE646231 | Hs.436886 | chr4p16.3 |
| 225582_at | KIAA1754 | KIAA1754 | AA425726 | Hs.28501 | chr10q25.1 |
| 225581_s_at | mitochondrial ribosomal protein L50 | MRPL50 | BG028213 | Hs.288224 | chr9q31.1 |
| 225579_at | PQ loop repeat containing 3 | PQLC3 | AL516202 | Hs.274415 | chr2p25.1 |
| 225578_at | similar to RIKEN cDNA 2410129H14 | RP11-11C5.2 | AI885466 | Hs.28465 | chr13q22.1 |
| 225576_at | chromosome 6 open reading frame 72 | C6orf72 | AI948460 | Hs.438872 | chr6q25.1 |
| 225574_at | RWD domain containing 4A | RWDD4A | BE613081 | Hs.133337 | chr4q35.1 |
| 225573_at | acyl-Coenzyme A dehydrogenase family, member 11 | ACAD11 | AL518293 | Hs.23100 | chr3q22.1 |
| 225570_at | solute carrier family 41, member 1 | SLC41A1 | AW439816 | Hs.20274 | chr1q32.1 |
| 225569_at | Eukaryotic translation initiation factor 2C, 2 | EIF2C2 | AI813489 | Hs.449415 | chr8q24 |
| 225568_at | transmembrane protein 141 | TMEM141 | BE728983 | Hs.356744 | chr9q34.3 |
| 225564_at | spermatogenesis associated 13 | SPATA13 | AW269397 |  |  |
| 225563_at | PAN3 polyA specific ribonuclease subunit homolog (S. cerevisiae) | PAN3 | AI970788 | Hs.369984 | chr13q12.2 |
| 225560_at | protein-O-mannosyltransferase 2 | POMT2 | AK023804 | Hs.132989 | chr14q24 |
| 225559_at | chromosome 3 open reading frame 19 | C3orf19 | AK023163 | Hs.236257 | chr3p25.1 |
| 225557_at | AXIN1 up-regulated 1 | AXUD1 | AI091372 | Hs.6607 | chr3p22 |
| 225556_at | hypothetical protein LOC203547 | LOC203547 | BG290058 | Hs.58633 | chrxq28 |
| 225547_at | small nucleolar RNA host gene (non-protein coding) 6 | SNHG6 | BG169443 | Hs.372680 |  |
| 225546_at | eukaryotic elongation factor-2 kinase | EEF2K | W68180 | Hs.259855 | chr16p12.1 |
| 225544_at | T-box 3 (ulnar mammary syndrome) | TBX3 | AI806338 | Hs.129895 | chr12q24.1 |
| 225541_at | ribosomal protein L22-like 1 | RPL22L1 | BE274422 | Hs.380933 | chr3q26.2 |
| 225539_at | zinc finger protein 295 | ZNF295 | AP001745 | Hs.441363 | chr21q22.3 |
| 225538_at | zinc finger, CCHC domain containing 9 | ZCCHC9 | BE539792 | Hs.15536 | chr5q14.1 |
| 225537_at | trafficking protein particle complex 6B | TRAPPC6B | AA936745 | Hs.13303 | chr14q21.1 |
| 225530_at | MOB1, Mps One Binder kinase activator-like 2A (yeast) | MOBKL2A | BE396735 | Hs.511804 | chr19p13.3 |
| 225528_at | importin 8 | IPO8 | AU149465 | Hs.316977 | chr12p11.21 |
| 225527_at | CCAAT/enhancer binding protein (C/EBP), gamma | CEBPG | BE622659 | Hs.2227 | chr19q13.11 |
| 225524_at | anthrax toxin receptor 2 | ANTXR2 | AU152178 | Hs.5897 | chr4q21.21 |
| 225523_at | mitochondrial ribosomal protein L53 | MRPL53 | AV726817 | Hs.430199 | chr2p13.1 |
| 225522_at | AP2 associated kinase 1 | AAK1 | AW628987 | Hs.135941 | chr2p13.3 |
| 225521_at | anaphase promoting complex subunit 7 | ANAPC7 | AL137586 | Hs.530379 | chr12q24.11 |
| 225517_at | zinc finger protein 770 | ZNF770 | AW236976 | Hs.5327 | chr15q14 |
| 225515_s_at | hypothetical protein LOC220906 | LOC220906 | BG402817 | Hs.372013 /// Hs.446506 | chr10p12.1 /// chr6p21.1 |
| 225509_at | hypothetical protein LOC56757 | LOC56757 | AI862477 |  | chr5q31-q32 |
| 225508_at | KIAA1468 | KIAA1468 | BF055274 | Hs.406541 | chr18q21.33 |
| 225505_s_at | family with sequence similarity 113, member A | FAM113A | NM_022760 | Hs.29341 | chr20p13 |
| 225497_at | arginyltransferase 1 | ATE1 | AL589591 | Hs.355589 | chr10q26.13 |
| 225496_s_at | synaptotagmin-like 2 | SYTL2 | N21426 | Hs.390463 | chr11q14 |
| 225493_at | hypothetical protein LOC144438 | LOC144438 | AI052701 | Hs.92308 | chr12q13.12 |
| 225489_at | transmembrane protein 18 | TMEM18 | AI720705 | Hs.43899 | chr2p25.3 |
| 225486_at | AT rich interactive domain 2 (ARID, RFX-like) | ARID2 | AB046777 | Hs.317304 | chr12q12 |
| 225484_at | testis specific, 14 | TSGA14 | AW157525 | Hs.282386 | chr7q32 |
| 225483_at | vacuolar protein sorting 26 homolog B (S. cerevisiae) | VPS26B | AI971602 | Hs.334684 | chr11q25 |
| 225480_at | chromosome 1 open reading frame 122 | C1orf122 | BE408081 | Hs.46736 | chr1p34.3 |
| 225479_at | leucine rich repeat containing 58 | LRRC58 | AL524175 |  |  |
| 225475_at | mesoderm induction early response 1 homolog (Xenopus laevis) | MIER1 | AI668786 | Hs.21757 | chr1p31.2 |
| 225473_at | chromosome 20 open reading frame 117 | C20orf117 | BE044516 | Hs.23213 | chr20q11.23 |
| 225472_at | HLA-B associated transcript 4 | BAT4 | AF129756 | Hs.247478 | chr6p21.3 |
| 225471_s_at | v-akt murine thymoma viral oncogene homolog 2 | AKT2 | BE734905 | Hs.326445 | chr19q13.1-q13.2 |
| 225470_at | nucleoporin 35kDa | NUP35 | AL529634 | Hs.180591 | chr2q32.1 |
| 225469_at | LYR motif containing 5 | LYRM5 | AA015609 | Hs.447488 | chr12p12.1 |
| 225468_at | FLJ36874 protein | FLJ36874 | AI761804 | Hs.374897 | chr11q12.1 |
| 225464_at | FERM domain containing 6 | FRMD6 | N30138 | Hs.439190 | chr14q22.1 |
| 225462_at | transmembrane protein 128 | TMEM128 | AV705805 | Hs.12845 | chr4p16.3 |
| 225461_at | euchromatic histone-lysine N-methyltransferase 1 | EHMT1 | BE504570 | Hs.416692 | chr9q34.3 |
| 225457_s_at | hypothetical LOC25845 | LOC25845 | BF528646 | Hs.529371 | chr5p15.33 |
| 225455_at | transcriptional adaptor 1 (HFI1 homolog, yeast)-like | TADA1L | AI760812 | Hs.435967 | chr1q24.1 |
| 225454_at | coiled-coil domain containing 124 | CCDC124 | AW248770 | Hs.100043 | chr19p13.11 |
| 225451_at | GRIP1 associated protein 1 | GRIPAP1 | AL136847 | Hs.109929 | chrxp11 |
| 225445_at | Hypothetical protein FLJ25778 | FLJ25778 | AI332346 |  |  |
| 225441_x_at | LSM domain containing 1 | LSMD1 | AA828224 | Hs.333414 | chr17p13.1 |
| 225440_at | 1-acylglycerol-3-phosphate O-acyltransferase 3 | AGPAT3 | BE737251 | Hs.443657 | chr21q22.3 |
| 225438_at | NudC domain containing 1 | NUDCD1 | BF445343 | Hs.195870 | chr8q23 |
| 225437_s_at | chromosome 7 open reading frame 27 | C7orf27 | AK024482 | Hs.236547 | chr7p22.3 |
| 225434_at | death effector domain containing 2 | DEDD2 | AW245401 | Hs.293225 | chr19q13.2 |
| 225433_at | General transcription factor IIA, 1, 19/37kDa | GTF2A1 | AU144104 | Hs.192232 | chr14q31.1 |
| 225431_x_at | aminoacylase 1-like 2 | ACY1L2 | BE779764 | Hs.356247 | chr6q15 |
| 225427_s_at | apolipoprotein A-I binding protein | APOA1BP | BE871169 | Hs.528320 | chr1q22 |
| 225422_at | cell division cycle 26 homolog (S. cerevisiae) | CDC26 | AI363359 | Hs.404060 | chr9q32 |
| 225420_at | glycerol-3-phosphate acyltransferase, mitochondrial | GPAM | AV699379 | Hs.42586 | chr10q25.2 |
| 225419_at | chromosome 7 open reading frame 11 | C7orf11 | AI869704 |  |  |
| 225415_at | deltex 3-like (Drosophila) | DTX3L | AA577672 | Hs.334875 | chr3q21.1 |
| 225413_at | upregulated during skeletal muscle growth 5 homolog (mouse) | USMG5 | BG291685 | Hs.500921 | chr10q24.33 |
| 225412_at | Transmembrane protein 87B | TMEM87B | AA761169 | Hs.266134 | chr2q13 |
| 225409_at | hypothetical protein MGC52110 | MGC52110 | AA115117 | Hs.177576 | chr2q12 |
| 225404_at | chromosome 1 open reading frame 212 | C1orf212 | R75637 | Hs.27160 | chr1p34.3 |
| 225403_at | chromosome 9 open reading frame 23 | C9orf23 | AL528391 | Hs.15961 | chr9p13.3 |
| 225402_at | TP53 regulating kinase | TP53RK | BG339450 | Hs.440263 | chr20q13.2 |
| 225399_at | chromosome 1 open reading frame 19 | C1orf19 | AF288394 | Hs.440663 | chr1q25 |
| 225398_at | RNA pseudouridylate synthase domain containing 4 | RPUSD4 | AL580514 | Hs.150458 | chr11q24.2 |
| 225396_at | Zinc finger and BTB domain containing 8 opposite strand | ZBTB8OS | AI928212 | Hs.534580 | chr1p34.3-p33 |
| 225394_s_at | zinc finger CCHC-type and RNA binding motif 1 | ZCRB1 | AI146850 | Hs.43847 | chr12q12 |
| 225389_at | BTB (POZ) domain containing 6 | BTBD6 | AW149498 | Hs.7367 | chr14q32 |
| 225388_at | tetraspanin 5 | TSPAN5 | AI928507 | Hs.8037 | chr4q23 |
| 225385_s_at | heterogeneous nuclear ribonucleoprotein L-like | HNRPLL | AW273811 | Hs.404336 | chr2p22.1 |
| 225384_at | dedicator of cytokinesis 7 | DOCK7 | BF001267 | Hs.406156 | chr1p31.3 |
| 225382_at | zinc finger protein 275 | ZNF275 | U82670 | Hs.348963 | chrxq28 |
| 225376_at | chromosome 20 open reading frame 11 | C20orf11 | BG480592 | Hs.103808 | chr20q13.33 |
| 225374_at | chromosome 17 open reading frame 32 | C17orf32 | BG110004 | Hs.368878 | chr17q11.2 |
| 225373_at | chromosome 10 open reading frame 54 | C10orf54 | BE271644 | Hs.132569 | chr10q22.3 |
| 225371_at | GLE1 RNA export mediator-like (yeast) | GLE1L | AI638714 | Hs.201157 | chr9q34.11 |
| 225362_at | family with sequence similarity 122B | FAM122B | AI341165 | Hs.404706 | chrxq26.3 |
| 225358_at | DnaJ (Hsp40) homolog, subfamily C, member 19 | DNAJC19 | AI003794 | Hs.389826 | chr3q26.33 |
| 225355_at | hypothetical protein DKFZP761M1511 | DKFZP761M1511 | AK026748 | Hs.91521 | chr5q35.2 |
| 225351_at | family with sequence similarity 45, member A | FAM45A | AI697488 | Hs.434241 | chr10q25 |
| 225348_at | similar to FUS-interacting serine-arginine-rich protein 1 (TLS-associated protein with Ser-Arg repeats) (TLS-associated protein with SR repeats) (TASR) (TLS-associated serine-arginine protein) (TLS-associated SR protein) (Neural-specific SR protein... | LOC727922 | AI954700 |  |  |
| 225347_at | ADP-ribosylation factor-like 8A | ARL8A | AL520984 | Hs.348709 | chr1q32.1 |
| 225344_at | nuclear receptor coactivator 7 | NCOA7 | AL035689 | Hs.292676 | chr6q22.32 |
| 225343_at | transmembrane emp24 protein transport domain containing 8 | TMED8 | AK025695 | Hs.212992 | chr14q24.3 |
| 225341_at | MTERF domain containing 3 | MTERFD3 | BF697312 | Hs.5009 | chr12q24.1 |
| 225335_at | zinc finger protein 496 | ZNF496 | AA191336 | Hs.22051 | chr1q44 |
| 225334_at | chromosome 10 open reading frame 32 | C10orf32 | AI147621 | Hs.34492 | chr10q24.32 |
| 225329_at | hypothetical protein LOC348262 | LOC348262 | BF338291 | Hs.285165 | chr17p13.3 |
| 225327_at | KIAA1370 | KIAA1370 | AB037791 | Hs.308019 | chr15q21.2-q21.3 |
| 225326_at | RNA binding motif protein 27 | RBM27 | AB037732 | Hs.61441 | chr5q32 |
| 225323_at | coiled-coil and C2 domain containing 1B | CC2D1B | AW514783 | Hs.318773 | chr1p32.3 |
| 225321_s_at | paired immunoglobin-like type 2 receptor beta | PILRB | AV762653 | Hs.349256 | chr7q22.1 |
| 225320_at | coiled-coil domain containing 109A | CCDC109A | AA579630 | Hs.128021 | chr10q22.1 |
| 225319_s_at | family with sequence similarity 104, member A | FAM104A | AA127686 | Hs.334878 | chr17q25.1 |
| 225318_at | DDHD domain containing 2 | DDHD2 | AW292751 | Hs.434966 | chr8p12 |
| 225317_at | acyl-Coenzyme A binding domain containing 6 | ACBD6 | AL574669 | Hs.63220 | chr1q25.2-q25.3 |
| 225315_at | mitochondrial ribosomal protein L21 | MRPL21 | BF344406 | Hs.380253 | chr11q13.3 |
| 225314_at | OCIA domain containing 2 | OCIAD2 | BG291649 | Hs.95835 | chr4p12 |
| 225312_at | COMM domain containing 6 | COMMD6 | AV704551 | Hs.356605 | chr13q22 |
| 225309_at | PHD finger protein 5A | PHF5A | AL008582 | Hs.273234 | chr22q13.2 |
| 225308_s_at | tetratricopeptide repeat, ankyrin repeat and coiled-coil containing 1 | TANC1 | AB051515 | Hs.437362 | chr2q24.2 |
| 225307_at | zinc finger protein 511 | ZNF511 | AL583632 | Hs.422113 | chr10q26.3 |
| 225304_s_at | NADH dehydrogenase (ubiquinone) 1 alpha subcomplex, 11, 14.7kDa | NDUFA11 | BE741920 | Hs.406062 | chr19p13.3 |
| 225303_at | kin of IRRE like (Drosophila) | KIRREL | AI049973 | Hs.375003 | chr1q21-q25 |
| 225302_at | thioredoxin domain containing 10 | TXNDC10 | BE503286 | Hs.440534 | chr18q22 |
| 225300_at | chromosome 15 open reading frame 23 | C15orf23 | BF792864 | Hs.181466 | chr15q15.1 |
| 225299_at | myosin VB | MYO5B | AB032945 | Hs.512590 | chr18q21 |
| 225297_at | coiled-coil domain containing 5 (spindle associated) | CCDC5 | AV715391 | Hs.436617 | chr18q21.1 |
| 225296_at | zinc finger protein 317 | ZNF317 | AB046808 |  |  |
| 225294_s_at | trafficking protein particle complex 1 | TRAPPC1 | BG340967 | Hs.24379 | chr17p13.1 |
| 225293_at | collagen, type XXVII, alpha 1 | COL27A1 | AK021957 | Hs.59892 | chr9q32 |
| 225291_at | polyribonucleotide nucleotidyltransferase 1 | PNPT1 | AI967971 | Hs.392004 | chr2p15 |
| 225287_s_at | transmembrane protein 55B | TMEM55B | AI992151 | Hs.7001 | chr14q11.2 |
| 225283_at | arrestin domain containing 4 | ARRDC4 | AV701177 | Hs.6093 | chr15q26.3 |
| 225282_at | stromal membrane-associated protein 1-like | SMAP1L | AL137764 | Hs.7750 | chr1p35.3-p34.1 |
| 225281_at | chromosome 3 open reading frame 17 | C3orf17 | AK024325 | Hs.377974 | chr3q13.2 |
| 225277_at | solute carrier family 39 (zinc transporter), member 13 | SLC39A13 | AL525780 | Hs.9070 | chr11p11.2 |
| 225272_at | spermidine/spermine N1-acetyltransferase 2 | SAT2 | AA128261 | Hs.10846 | chr17p13.1 |
| 225271_at | transmembrane protein 63B | TMEM63B | AI732727 | Hs.414473 | chr6p21.1 |
| 225263_at | similar to Heparan-sulfate 6-O-sulfotransferase 1 (HS6ST-1) | LOC728969 | BC001196 | Hs.380792 | chr2q21 |
| 225260_s_at | mitochondrial ribosomal protein L32 | MRPL32 | AL551823 | Hs.50252 | chr7p14 |
| 225258_at | filamin binding LIM protein 1 | FBLIM1 | AL133035 | Hs.8728 | chr1p36.13 |
| 225257_at | coiled-coil domain containing 97 | CCDC97 | AA534536 | Hs.437497 | chr19q13.2 |
| 225252_at | sulfiredoxin 1 homolog (S. cerevisiae) | SRXN1 | AL121758 | Hs.135056 | chr20p13 |
| 225251_at | RAB24, member RAS oncogene family | RAB24 | AK021761 | Hs.16258 | chr5q35.3 |
| 225250_at | stromal interaction molecule 2 | STIM2 | N48266 | Hs.446203 | chr4p15.2 |
| 225247_at | chromosome 19 open reading frame 6 | C19orf6 | BF568836 | Hs.380962 | chr19p13.3 |
| 225244_at | chromosome 1 open reading frame 142 | C1orf142 | AA019893 | Hs.325081 | chr1q42.13 |
| 225242_s_at | coiled-coil domain containing 80 | CCDC80 | AW303375 | Hs.356289 | chr3q13.2 |
| 225236_at | RNA binding motif protein 18 | RBM18 | AA167623 |  |  |
| 225235_at | tetraspanin 17 | TSPAN17 | AW007710 | Hs.207085 | chr5q35.3 |
| 225232_at | myotubularin related protein 12 | MTMR12 | AA524700 | Hs.93872 | chr5p13.3 |
| 225228_at | transmembrane protein 77 | TMEM77 | AI474054 | Hs.245537 | chr1p13.3 |
| 225226_at | family with sequence similarity 40, member A | FAM40A | AB051548 | Hs.169577 | chr1p13.3 |
| 225224_at | chromosome 20 open reading frame 112 | C20orf112 | AL034550 |  |  |
| 225222_at | hippocampus abundant transcript 1 | HIAT1 | AI243268 | Hs.131799 | chr1p21.3 |
| 225220_at | Small nucleolar RNA, H/ACA box 24 | SNORA24 | BF340290 | Hs.296141 |  |
| 225218_at | zinc finger, FYVE domain containing 27 | ZFYVE27 | AA205754 | Hs.293224 | chr10q24.2 |
| 225217_s_at | bromodomain and PHD finger containing, 3 | BRPF3 | AB033112 | Hs.246795 | chr6p21 |
| 225216_at | Chromosome X open reading frame 39 | CXorf39 | AI590719 | Hs.54347 | chrxq22.2 |
| 225208_s_at | family with sequence similarity 103, member A1 | FAM103A1 | AW575350 | Hs.80624 | chr15q25.2 |
| 225206_s_at | Mitochondrial translational release factor 1-like | MTRF1L | AI479199 | Hs.225836 | chr6q25-q26 |
| 225203_at | protein phosphatase 1, regulatory (inhibitor) subunit 16A | PPP1R16A | AI742931 | Hs.437099 | chr8q24.3 |
| 225201_s_at | mitochondrial ribosomal protein L14 | MRPL14 | BF569593 | Hs.343579 | chr6p21.3 |
| 225196_s_at | mitochondrial ribosomal protein S26 | MRPS26 | AF308301 | Hs.18946 | chr20p13 |
| 225195_at | DPH3, KTI11 homolog (S. cerevisiae) | DPH3 | AI825858 | Hs.388087 | chr3p25.1 |
| 225194_at | pleiotropic regulator 1 (PRL1 homolog, Arabidopsis) | PLRG1 | NM_002669 | Hs.249996 | chr4q31.2-q32.1 |
| 225192_at | chromosome 10 open reading frame 46 | C10orf46 | AA044726 | Hs.408319 | chr10q26.11 |
| 225186_at | Ras association (RalGDS/AF-6) and pleckstrin homology domains 1 | RAPH1 | BF114947 | Hs.170399 | chr2q33 |
| 225184_at | AT rich interactive domain 1B (SWI1-like) /// similar to AT rich interactive domain 1B (SWI1-like) isoform 1 | ARID1B | AK000921 | Hs.436008 | chr6q25.1 |
| 225178_at | tetratricopeptide repeat domain 14 | TTC14 | W73788 | Hs.43213 | chr3q26.33 |
| 225176_at | Leucyl/cystinyl aminopeptidase | LNPEP | AA156754 | Hs.356079 |  |
| 225175_s_at | solute carrier family 44, member 2 | SLC44A2 | AI569503 | Hs.105509 | chr19p13.1 |
| 225172_at | Crm, cramped-like (Drosophila) | CRAMP1L | AB037847 |  | chr16p13.3 |
| 225166_at | Rho GTPase activating protein 18 | ARHGAP18 | AU158022 | Hs.413282 | chr6q22.33 |
| 225164_s_at | eukaryotic translation initiation factor 2 alpha kinase 4 | EIF2AK4 | AB037759 | Hs.412102 | chr15q14 |
| 225162_at | SH3 domain protein D19 | SH3D19 | BG285417 | Hs.24715 | chr4q31.3 |
| 225160_x_at | hypothetical protein MGC5370 | MGC5370 | AI952357 | Hs.334873 | chr12q14.3 |
| 225158_at | G elongation factor, mitochondrial 1 | GFM1 | BF978647 | Hs.274543 | chr3q25.1-q26.2 |
| 225156_at | elongation factor 1 homolog (S. cerevisiae) | ELOF1 | AK001171 | Hs.321391 | chr19p13.2 |
| 225155_at | small nucleolar RNA host gene (non-protein coding) 5 | SNHG5 | BG339050 | Hs.292457 | chr6q14.3 |
| 225154_at | synapse associated protein 1, SAP47 homolog (Drosophila) | SYAP1 | BG029566 | Hs.489336 | chrxp22.2 |
| 225152_at | zinc finger protein 622 | ZNF622 | BF940944 | Hs.60300 | chr5p15.1 |
| 225148_at | ribosomal protein S19 binding protein 1 | RPS19BP1 | BE907429 | Hs.350200 | chr22q13.1 |
| 225147_at | pleckstrin homology, Sec7 and coiled-coil domains 3 | PSCD3 | AL521959 | Hs.7984 | chr7p22.1 |
| 225146_at | chromosome 9 open reading frame 25 | C9orf25 | AW005237 | Hs.7961 | chr9p13.3 |
| 225145_at | nuclear receptor coactivator 5 | NCOA5 | AB046857 | Hs.445597 | chr20q12-q13.12 |
| 225142_at | KIAA1718 protein | KIAA1718 | AW294022 | Hs.222707 | chr7q34 |
| 225141_at | RNA binding motif protein 35B | RBM35B | AW298438 | Hs.172674 | chr16q22.2 |
| 225140_at | Kruppel-like factor 3 (basic) | KLF3 | BF438116 | Hs.145754 | chr4p14 |
| 225136_at | Pleckstrin homology domain containing, family A (phosphoinositide binding specific) member 2 | PLEKHA2 | BF968578 | Hs.470847 | chr8p11.23 |
| 225135_at | SIN3 homolog A, transcription regulator (yeast) | SIN3A | AI433017 | Hs.22583 | chr15q23 |
| 225132_at | F-box and leucine-rich repeat protein 3 | FBXL3 | AI983021 | Hs.314326 | chr13q22 |
| 225131_at | zinc finger, RAN-binding domain containing 1 | ZRANB1 | H60265 | Hs.187711 | chr10q26.13 |
| 225129_at | copine II | CPNE2 | AW170571 | Hs.339809 | chr16q13 |
| 225128_at | KDEL (Lys-Asp-Glu-Leu) containing 2 | KDELC2 | AL548941 | Hs.83286 | chr11q22.3 |
| 225127_at | transmembrane protein 181 | TMEM181 | BF217531 | Hs.99145 | chr6q25.3 |
| 225126_at | mitochondrial ribosome recycling factor | MRRF | BF438352 | Hs.368011 | chr9q33.2 |
| 225125_at | transmembrane protein 32 | TMEM32 | BF978280 | Hs.110702 | chrxq26.3 |
| 225121_at | TBC1 domain family, member 23 | TBC1D23 | AK001750 | Hs.16986 | chr3q12.2 |
| 225119_at | chromatin modifying protein 4B | CHMP4B | AW299290 | Hs.399981 | chr20q11.22 |
| 225108_at | HEAT repeat containing 2 | HEATR2 | BF111719 | Hs.407933 | chr2q31 |
| 225107_at | Heterogeneous nuclear ribonucleoprotein A2/B1 | HNRPA2B1 | AI963008 | Hs.232400 | chr7p15 |
| 225105_at | hypothetical protein | LOC387882 | BF969397 | Hs.301711 | chr12q23.3 |
| 225104_at | zinc finger protein 598 | ZNF598 | AI143307 | Hs.343828 | chr16p13.3 |
| 225103_at | mitochondrial ribosomal protein L38 | MRPL38 | BE855557 | Hs.442609 | chr17q25.3 |
| 225101_s_at | sorting nexin 14 | SNX14 | AL578668 | Hs.512657 | chr6q14.3 |
| 225100_at | F-box protein 45 | FBXO45 | BF590021 | Hs.6994 | chr3q29 |
| 225096_at | chromosome 17 open reading frame 79 | C17orf79 | AJ272196 | Hs.8179 | chr17q11.2 |
| 225094_at | SET domain containing (lysine methyltransferase) 8 | SETD8 | AL578116 | Hs.287493 | chr12q24.31 |
| 225093_at | utrophin | UTRN | N66570 | Hs.250607 | chr6q24 |
| 225091_at | zinc finger, CCHC domain containing 3 | ZCCHC3 | AL034548 | Hs.28608 | chr20p13-p12.2 |
| 225089_at | ubiquitin specific peptidase 40 | USP40 | AA127740 | Hs.96513 | chr2q37.1 |
| 225088_at | chromosome 16 open reading frame 63 | C16orf63 | BG546917 | Hs.350424 | chr16p13.11 |
| 225083_at | chromosome 6 open reading frame 51 | C6orf51 | AL536986 | Hs.418520 | chr6q21 |
| 225082_at | cleavage and polyadenylation specific factor 3, 73kDa | CPSF3 | NM_016207 | Hs.16251 | chr2p25.1 |
| 225081_s_at | cell division cycle associated 7-like | CDCA7L | AK022955 | Hs.351623 | chr7p15.3 |
| 225077_at | hypothetical protein LOC283680 | LOC283680 | AA890703 |  | chr15q26.1 |
| 225076_s_at | zinc finger, NFX1-type containing 1 | ZNFX1 | AA150460 | Hs.371794 | chr20q13.13 |
| 225075_at | p53 and DNA damage regulated 1 | PDRG1 | AL031658 | Hs.22181 | chr20q11.21 |
| 225074_at | RAB2B, member RAS oncogene family | RAB2B | AA531016 | Hs.22399 | chr14q11.2 |
| 225070_at | nuclear undecaprenyl pyrophosphate synthase 1 homolog (S. cerevisiae) | NUS1 | BF112132 | Hs.289008 | chr6q22.1 |
| 225067_at | unc-51-like kinase 3 (C. elegans) | ULK3 | AL117482 | Hs.7978 | chr15q24.1 |
| 225065_x_at | chromosome 17 open reading frame 45 | C17orf45 | AI826279 | Hs.270232 | chr17p11.2 |
| 225063_at | ubiquitin-like 7 (bone marrow stromal cell-derived) | UBL7 | BF568780 | Hs.334713 | chr15q24.1 |
| 225062_at | hypothetical gene supported by AL713796 | LOC389831 | BF667120 | Hs.389638 |  |
| 225061_at | DnaJ (Hsp40) homolog, subfamily A, member 4 | DNAJA4 | N45231 | Hs.76853 | chr15q25.1 |
| 225060_at | low density lipoprotein receptor-related protein 11 | LRP11 | BF696304 | Hs.408355 | chr6q25.1 |
| 225059_at | angiotensin II receptor-associated protein | AGTRAP | BE875567 | Hs.12854 | chr1p36.22 |
| 225058_at | G protein-coupled receptor 108 | GPR108 | AL365404 | Hs.167641 | chr19p13.3 |
| 225056_at | signal-induced proliferation-associated 1 like 2 | SIPA1L2 | AB037810 | Hs.498063 | chr1q42.2 |
| 225055_at | Archaemetzincins-2 | AMZ2 | AV735241 | Hs.443457 | chr17q24.2 |
| 225052_at | hypothetical protein MGC14327 | MGC14327 | AL520657 | Hs.231029 | chr9q34.3 |
| 225049_at | biogenesis of lysosome-related organelles complex-1, subunit 2 | BLOC1S2 | BF382281 | Hs.34906 | chr10q24.31 |
| 225046_at | Hypothetical protein LOC645524 | FLJ36840 | AL521247 | Hs.389638 |  |
| 225044_at | 5'-nucleotidase, cytosolic III-like | NT5C3L | AL526783 | Hs.237536 | chr17q21.2 |
| 225043_at | solute carrier family 15, member 4 | SLC15A4 | AW304786 | Hs.274268 | chr12q24.32 |
| 225042_s_at | chromosome 12 open reading frame 22 | C12orf22 | AW449343 | Hs.18879 | chr12q13.11-q13.12 |
| 225038_s_at | surfeit 6 | SURF6 | AI745183 | Hs.460333 | chr9q34.2 |
| 225036_at | Src homology 2 domain containing adaptor protein B /// chromosome 9 open reading frame 105 | SHB | BF969806 |  | chr9p13.1 |
| 225034_at | hypothetical protein LOC286167 | LOC286167 | AI885015 |  | chr8q24.22 |
| 225030_at | family with sequence similarity 44, member B | FAM44B | AA824341 | Hs.425091 | chr5q35.2 |
| 225028_at | hypothetical protein LOC550643 | LOC550643 | AI417268 | Hs.355559 |  |
| 225026_at | chromodomain helicase DNA binding protein 6 | CHD6 | BF572029 | Hs.45207 | chr20q12 |
| 225024_at | chromosome 20 open reading frame 77 | C20orf77 | AL117521 | Hs.278839 | chr20q11.21-q12 |
| 225021_at | zinc finger protein 532 | ZNF532 | AA861416 | Hs.368756 | chr18q21.32 |
| 225020_at | DAB2 interacting protein | DAB2IP | AB051530 | Hs.238465 | chr9q33.1-q33.3 |
| 225017_at | coiled-coil domain containing 14 | CCDC14 | AK022954 | Hs.17731 | chr3q21.1 |
| 225014_at | hypothetical gene supported by BC032431 | LOC389203 | BF508958 | Hs.235026 | chr4p15.2 |
| 225008_at | Hypothetical protein MGC34646 | MGC34646 | AW469351 | Hs.55258 | chr8q12.2-q12.3 |
| 225005_at | PHD finger protein 13 | PHF13 | AL039384 | Hs.7299 | chr1p36.31 |
| 225004_at | transmembrane protein 101 | TMEM101 | AL525613 | Hs.282883 | chr17q21.31 |
| 225003_at | hypothetical protein MGC3205 /// MBC3205 | MGC3205 | BF343862 | Hs.8036 | chr19p13.2 |
| 225002_s_at | sulfatase modifying factor 2 | SUMF2 | BE349022 | Hs.279696 | chr7q11.1 |
| 224998_at | CKLF-like MARVEL transmembrane domain containing 4 | CMTM4 | AK000855 | Hs.325825 | chr16q22.1 |
| 224997_x_at | H19, imprinted maternally expressed untranslated mRNA | H19 | AL575306 | Hs.415722 | chr11p15.5 |
| 224993_at | myeloid/lymphoid or mixed-lineage leukemia (trithorax homolog, Drosophila); translocated to, 1 /// similar to myeloid/lymphoid or mixed-lineage leukemia (trithorax homolog, Drosophila); translocated to, 1 | MLLT1 | AW962605 | Hs.10095 | chr19p13.3 |
| 224991_at | c-Maf-inducing protein | CMIP | AI819630 | Hs.461510 | chr16q23 |
| 224990_at | chromosome 4 open reading frame 34 | C4orf34 | BE972723 | Hs.500350 | chr4p14 |
| 224987_at | chromosome 6 open reading frame 89 | C6orf89 | BG170130 | Hs.433381 | chr6p21.2 |
| 224981_at | hypothetical protein BC017488 | LOC124446 | AL520900 | Hs.291623 | chr16p11.2 |
| 224975_at | nuclear factor I/A | NFIA | AB037860 | Hs.173933 | chr1p31.3-p31.2 |
| 224972_at | chromosome 20 open reading frame 52 | C20orf52 | BF381837 | Hs.401703 | chr20q11.22 |
| 224969_at | ataxin 7-like 3 | ATXN7L3 | AL390158 | Hs.181391 | chr17q21.31 |
| 224968_at | coiled-coil domain containing 104 | CCDC104 | AL518311 | Hs.23128 | chr2p16.1 |
| 224964_s_at | guanine nucleotide binding protein (G protein), gamma 2 | GNG2 | AK026424 | Hs.112928 | chr14q21 |
| 224963_at | solute carrier family 26 (sulfate transporter), member 2 | SLC26A2 | AK025078 | Hs.302738 | chr5q31-q34 |
| 224962_at | hypothetical gene supported by AK023162 | LOC90120 | AU153030 | Hs.9100 | chr9q34.3 |
| 224960_at | SCY1-like 2 (S. cerevisiae) | SCYL2 | AK024274 | Hs.71573 | chr12q23.1 |
| 224958_at | nuclear fragile X mental retardation protein interacting protein 2 | NUFIP2 | AV728521 | Hs.355608 | chr17q11.2 |
| 224957_at | putative NFkB activating protein | LOC497661 | AL572206 | Hs.374588 | chr18q21 |
| 224950_at | prostaglandin F2 receptor negative regulator | PTGFRN | BF476250 | Hs.418093 | chr1p13.1 |
| 224948_at | mitochondrial ribosomal protein S24 | MRPS24 | BF970023 | Hs.284286 | chr7p14 |
| 224947_at | ring finger protein 26 | RNF26 | AL559247 | Hs.26412 | chr11q23 |
| 224946_s_at | coiled-coil domain containing 115 | CCDC115 | AL571677 | Hs.104203 | chr2q21.1 |
| 224943_at | BTB (POZ) domain containing 7 | BTBD7 | AI580162 | Hs.379758 | chr14q32.13 |
| 224932_at | chromosome 22 open reading frame 16 | C22orf16 | AI814909 | Hs.66915 | chr22q11.23 |
| 224931_at | solute carrier family 41, member 3 | SLC41A3 | AK021925 | Hs.247547 | chr3q21.2 |
| 224928_at | SET domain containing (lysine methyltransferase) 7 | SETD7 | AK024846 |  |  |
| 224927_at | KIAA1949 | KIAA1949 | BG251556 | Hs.101150 | chr6p21.3 |
| 224926_at | exocyst complex component 4 | EXOC4 | AB051486 | Hs.446140 | chr7q31 |
| 224923_at | tetratricopeptide repeat domain 7A | TTC7A | AL117512 | Hs.438277 | chr2p21 |
| 224921_at | secretory carrier membrane protein 2 | SCAMP2 | AW409599 | Hs.238030 | chr15q23-q25 |
| 224920_x_at | myeloid-associated differentiation marker | MYADM | AA909044 | Hs.380906 | chr19q13.42 |
| 224919_at | mitochondrial ribosomal protein S6 | MRPS6 | AL555227 | Hs.268016 | chr21q21.3-q22.1 |
| 224917_at | microRNA 21 | MIRN21 | BF674052 | Hs.166254 | chr17q23.2 |
| 224913_s_at | translocase of inner mitochondrial membrane 50 homolog (S. cerevisiae) | TIMM50 | AA877820 | Hs.355819 | chr19q13.2 |
| 224910_at | calcium regulated heat stable protein 1, 24kDa | CARHSP1 | AL575747 | Hs.355723 | chr16p13.2 |
| 224908_s_at | tubulin tyrosine ligase | TTL | AI888594 | Hs.358997 | chr2q13 |
| 224906_at | transmembrane protein 16F | TMEM16F | AL041280 | Hs.410941 | chr12q12 |
| 224904_at | pyruvate dehydrogenase phosphatase regulatory subunit | PDPR | AV724415 | Hs.356439 | chr16q22.1 |
| 224903_at | cirrhosis, autosomal recessive 1A (cirhin) /// family with sequence similarity 83, member G | CIRH1A | AL519818 | Hs.151001 | chr16q22.1 |
| 224901_at | stearoyl-CoA desaturase 5 | SCD5 | AL571375 | Hs.379191 | chr4q21.3 |
| 224900_at | ankyrin repeat and FYVE domain containing 1 | ANKFY1 | AK025960 | Hs.128020 | chr17p13.3 |
| 224898_at | WD repeat domain 26 | WDR26 | BG177759 | Hs.406339 | chr1q42.12 |
| 224890_s_at | similar to CG14977-PA | LOC389541 | BE727643 | Hs.406520 | chr7 |
| 224887_at | N-acetylglucosamine-1-phosphate transferase, gamma subunit | GNPTG | AF302786 | Hs.241575 | chr16p13.3 |
| 224886_at | hypothetical LOC339123 | LOC339123 | AL577395 | Hs.534746 | chr16p13.3 |
| 224885_s_at | keratinocyte associated protein 2 | KRTCAP2 | BE260771 | Hs.374854 | chr1q22 |
| 224883_at | pallidin homolog (mouse) | PLDN | AI344311 | Hs.7037 | chr15q21.1 |
| 224882_at | acyl-CoA synthetase short-chain family member 1 | ACSS1 | AL035661 | Hs.7218 | chr20p11.23-p11.21 |
| 224879_at | chromosome 9 open reading frame 123 | C9orf123 | BF315994 | Hs.7517 | chr9p24.1 |
| 224877_s_at | mitochondrial ribosomal protein S5 | MRPS5 | BE970056 | Hs.387988 | chr2p11.2-q11.2 |
| 224873_s_at | mitochondrial ribosomal protein S25 | MRPS25 | AK024433 | Hs.128201 | chr3p25 |
| 224872_at | DIP2 disco-interacting protein 2 homolog B (Drosophila) | DIP2B | AB040896 | Hs.21104 | chr12q13.12 |
| 224871_at | family with sequence similarity 79, member A | FAM79A | AK025464 | Hs.20529 | chr1p36.32 |
| 224870_at | KIAA0114 | KIAA0114 | BG255416 | Hs.535909 | chr4q12 |
| 224868_at | zinc finger, DHHC-type containing 5 | ZDHHC5 | BE961925 | Hs.27239 | chr11q12.1 |
| 224867_at | chromosome 1 open reading frame 151 | C1orf151 | BF244614 | Hs.343588 | chr1p36.13 |
| 224859_at | CD276 molecule | CD276 | AL360136 | Hs.77873 | chr15q23-q24 |
| 224855_at | pyrroline-5-carboxylate reductase family, member 2 | PYCR2 | AL561868 | Hs.274287 | chr1q42.12 |
| 224851_at | cyclin-dependent kinase 6 | CDK6 | AW274756 | Hs.38481 | chr7q21-q22 |
| 224850_at | ATPase family, AAA domain containing 1 | ATAD1 | BE645232 | Hs.435724 | chr10q23.31 |
| 224845_s_at | SLAIN motif family, member 2 | SLAIN2 | AU159395 | Hs.27263 | chr4p12 |
| 224836_at | tumor protein p53 inducible nuclear protein 2 | TP53INP2 | AL109824 | Hs.11114 | chr20q11.22 |
| 224835_at | hypothetical protein KIAA1434 | RP5-1022P6.2 | AL109935 | Hs.472040 | chr20p12.3 |
| 224834_at | ubiquitin domain containing 2 | UBTD2 | BF243404 | Hs.179852 | chr5q35.1 |
| 224825_at | deoxynucleotidyltransferase, terminal, interacting protein 1 | DNTTIP1 | AL050348 | Hs.26213 | chr20q13.12 |
| 224823_at | myosin, light chain kinase | MYLK | AA526844 | Hs.386078 | chr3q21 |
| 224821_at | abhydrolase domain containing 14B | ABHD14B | AL520200 | Hs.236025 | chr3p21.2 |
| 224820_at | family with sequence similarity 36, member A | FAM36A | AA831192 | Hs.411490 | chr1q44 |
| 224819_at | transcription elongation factor A (SII)-like 8 | TCEAL8 | AI743979 | Hs.389734 | chrxq22.1 |
| 224815_at | COMM domain containing 7 | COMMD7 | AA148301 | Hs.408427 | chr20q11.21 |
| 224810_s_at | ankyrin repeat domain 13A | ANKRD13A | AL569476 | Hs.528703 | chr12q24.11 |
| 224807_at | GRAM domain containing 1A | GRAMD1A | AB040966 | Hs.365476 | chr19q13.11 |
| 224806_at | tripartite motif-containing 25 | TRIM25 | BE563152 | Hs.88914 | chr17q23.2 |
| 224804_s_at | chromosome 15 open reading frame 17 | C15orf17 | AU152410 | Hs.367690 | chr15q24.1 |
| 224799_at | Nedd4 family interacting protein 2 | NDFIP2 | AW290956 | Hs.30340 | chr13q31.1 |
| 224797_at | arrestin domain containing 3 | ARRDC3 | AB037797 | Hs.24684 | chr5q14.3 |
| 224794_s_at | cerebral endothelial cell adhesion molecule 1 | CEECAM1 | AA654142 | Hs.23954 | chr9q34.11 |
| 224793_s_at | transforming growth factor, beta receptor I (activin A receptor type II-like kinase, 53kDa) | TGFBR1 | AA604375 | Hs.28005 | chr9q22 |
| 224792_at | tankyrase 1 binding protein 1, 182kDa | TNKS1BP1 | AL566438 | Hs.9645 | chr11q12.1 |
| 224789_at | WD repeat domain 40A | WDR40A | AL555107 | Hs.102669 | chr9p13.3 |
| 224784_at | myeloid/lymphoid or mixed-lineage leukemia (trithorax homolog, Drosophila); translocated to, 6 | MLLT6 | BG024886 | Hs.282233 | chr17q21 |
| 224783_at | family with sequence similarity 100, member B | FAM100B | AA831661 | Hs.149931 | chr17q25.1 |
| 224782_at | zinc finger, matrin type 2 | ZMAT2 | AA205643 | Hs.350194 | chr5q31.3 |
| 224779_s_at | family with sequence similarity 96, member A | FAM96A | AI193090 | Hs.439548 | chr15q22.31 |
| 224776_at | 1-acylglycerol-3-phosphate O-acyltransferase 6 (lysophosphatidic acid acyltransferase, zeta) | AGPAT6 | BF513102 | Hs.355753 | chr8p11.21 |
| 224774_s_at | neuron navigator 1 | NAV1 | AK022622 | Hs.6298 | chr1q32.3 |
| 224768_at | IWS1 homolog (S. cerevisiae) | IWS1 | AK001717 | Hs.130259 | chr2q14.3 |
| 224763_at | Ribosomal protein L37 | RPL37 | BF724210 | Hs.80545 | chr5p13 |
| 224761_at | guanine nucleotide binding protein (G protein), alpha 13 | GNA13 | AI928136 | Hs.9691 | chr17q24.3 |
| 224759_s_at | chromosome 12 open reading frame 23 | C12orf23 | AK001731 | Hs.191981 | chr12q23.3 |
| 224758_at | solute carrier family 13 (sodium/sulfate symporters), member 4 /// hypothetical protein LOC641845 /// hypothetical LOC647087 | SLC13A4 | BG393032 | Hs.9098 | chr7q33 |
| 224756_s_at | HLA-B associated transcript 5 | BAT5 | AL578487 | Hs.388188 | chr6p21.3 |
| 224754_at | Sp1 transcription factor | SP1 | BG431266 | Hs.9460 | chr12q13.1 |
| 224751_at | hypothetical protein LOC641845 /// hypothetical LOC647087 | LOC641845 | BE738276 | Hs.200022 |  |
| 224749_at | integrin alpha FG-GAP repeat containing 3 | ITFG3 | AI688331 | Hs.322456 | chr16p13.3 |
| 224748_at | WD repeat domain 68 | WDR68 | AK025925 | Hs.410596 | chr17q23.3 |
| 224747_at | ubiquitin-conjugating enzyme E2Q (putative) 2 | UBE2Q2 | AK000617 | Hs.95262 | chr15q23 |
| 224746_at | KIAA1522 | KIAA1522 | AB040955 | Hs.528659 | chr1p35.1 |
| 224745_x_at | OTU domain containing 5 | OTUD5 | AK026260 | Hs.444460 | chrxp11.23 |
| 224744_at | inositol monophosphatase domain containing 1 | IMPAD1 | AW628838 | Hs.378857 | chr8q12.1 |
| 224740_at | hypothetical protein LOC643155 | DKFZP686E2158 | BE613001 | Hs.5064 | chr5q12.1 |
| 224739_at | pim-3 oncogene | PIM3 | BE778706 | Hs.530381 |  |
| 224738_x_at | Ribosomal protein L7-like 1 | RPL7L1 | BE858199 | Hs.446506 | chr6p21.1 |
| 224736_at | cell division cycle and apoptosis regulator 1 | CCAR1 | BE617899 | Hs.444635 | chr10q21.3 |
| 224735_at | cytochrome b, ascorbate dependent 3 | CYBASC3 | AA683481 | Hs.22546 | chr11q12.2 |
| 224734_at | high-mobility group box 1 /// similar to High mobility group protein B1 (High mobility group protein 1) (HMG-1) (Amphoterin) (Heparin-binding protein p30) /// similar to High mobility group protein B1 (High mobility group protein 1) (HMG-1) (Amphoterin) ( | HMGB1 | N92507 | Hs.434102 | chr13q12 |
| 224732_at | chromosome transmission fidelity factor 8 homolog (S. cerevisiae) | CTF8 | AI309784 | Hs.535601 | chr16q22.1 |
| 224729_s_at | ATP synthase mitochondrial F1 complex assembly factor 1 | ATPAF1 | AI653592 | Hs.10964 | chr1p33-p32.3 |
| 224727_at | hematopoietic signal peptide-containing | LOC284361 | AL045545 | Hs.250465 | chr19q13.33 |
| 224723_x_at | hypothetical LOC401397 | LOC401397 | AV700969 | Hs.117929 | chr7 |
| 224722_at | mindbomb homolog 1 (Drosophila) | MIB1 | BE048628 | Hs.34892 | chr18q11.2 |
| 224721_at | WD repeat domain 75 | WDR75 | AI917328 | Hs.399984 | chr2q32.2 |
| 224719_s_at | chromosome 12 open reading frame 57 | C12orf57 | BG339653 | Hs.405913 | chr12p13.31 |
| 224716_at | solute carrier family 35, member B2 | SLC35B2 | BG163267 | Hs.182885 | chr6p12.1-p11.2 |
| 224715_at | WD repeat domain 34 | WDR34 | BG338983 | Hs.163502 | chr9q34.11 |
| 224713_at | MKI67 (FHA domain) interacting nucleolar phosphoprotein | MKI67IP | AL577809 | Hs.367842 | chr2q14.3 |
| 224712_x_at | chromosome 19 open reading frame 42 | C19orf42 | AI656658 | Hs.356467 | chr19p13.11 |
| 224707_at | chromosome 5 open reading frame 32 | C5orf32 | AL522667 | Hs.323512 | chr5q31.2 |
| 224706_at | KIAA2013 | KIAA2013 | BE879846 | Hs.438889 | chr1p36.22 |
| 224705_s_at | trinucleotide repeat containing 6A | TNRC6A | AI684626 | Hs.407740 | chr16p11.2 |
| 224702_at | transmembrane protein 167 | TMEM167 | BG501219 |  |  |
| 224700_at | STT3, subunit of the oligosaccharyltransferase complex, homolog B (S. cerevisiae) | STT3B | BF221532 | Hs.160918 | chr3p24.1 |
| 224695_at | chromosome 2 open reading frame 29 | C2orf29 | AK024221 | Hs.30819 | chr2q11.2 |
| 224694_at | anthrax toxin receptor 1 | ANTXR1 | AF279145 | Hs.274520 | chr2p13.1 |
| 224693_at | chromosome 20 open reading frame 108 | C20orf108 | AI133137 | Hs.143736 | chr20q13.31 |
| 224692_at | protein phosphatase 1, regulatory (inhibitor) subunit 15B | PPP1R15B | BF796046 | Hs.355489 | chr1q32.1 |
| 224689_at | mannosidase, beta A, lysosomal-like | MANBAL | AL034422 | Hs.6126 | chr20q11.23-q12 |
| 224688_at | chromosome 7 open reading frame 42 | C7orf42 | BE962299 | Hs.287955 | chr7q11.21 |
| 224686_x_at | Hypothetical protein LOC728871 /// Leucine rich repeat containing 37, member A2 | LOC728871 | AA045233 | Hs.500300 |  |
| 224685_at | myeloid/lymphoid or mixed-lineage leukemia (trithorax homolog, Drosophila); translocated to, 4 | MLLT4 | AI675354 | Hs.100469 | chr6q27 |
| 224683_at | F-box protein, helicase, 18 | FBXO18 | BE961916 | Hs.387099 | chr10p15.1 |
| 224682_at | ankyrin repeat and IBR domain containing 1 | ANKIB1 | AA253488 | Hs.83293 | chr7q21.2 |
| 224679_at | mesoderm development candidate 2 | MESDC2 | BE963495 | Hs.78871 | chr15q13 |
| 224674_at | tweety homolog 3 (Drosophila) | TTYH3 | AI934753 | Hs.440899 | chr7p22 |
| 224673_at | leukocyte receptor cluster (LRC) member 8 | LENG8 | AI613244 | Hs.502378 | chr19q13.42 |
| 224671_at | mitochondrial ribosomal protein L10 | MRPL10 | AL571373 | Hs.347535 | chr17q21.32 |
| 224668_at | SYS1 Golgi-localized integral membrane protein homolog (S. cerevisiae) | SYS1 | N30607 | Hs.130463 | chr20q13.12 |
| 224666_at | non-SMC element 1 homolog (S. cerevisiae) | NSMCE1 | AF161451 | Hs.284295 | chr16p12.1 |
| 224664_at | chromosome 10 open reading frame 104 | C10orf104 | BE962336 | Hs.426296 | chr10q22.1 |
| 224661_at | phosphatidylinositol glycan anchor biosynthesis, class Y | PIGY | AW028485 | Hs.26136 | chr4q22.1 |
| 224659_at | selenoprotein N, 1 | SEPN1 | AL020996 | Hs.8518 | chr1p36.13 |
| 224658_x_at | phosphofurin acidic cluster sorting protein 1 | PACS1 | AL530612 | Hs.328142 | chr11q13.1-q13.2 |
| 224656_s_at | myotrophin | MTPN | AL533334 | Hs.333178 | chr7q33 |
| 224652_at | chromosome 10 open reading frame 9 | C10orf9 | AV701987 | Hs.6820 | chr10p11.21 |
| 224645_at | Eukaryotic translation initiation factor 4E binding protein 2 | EIF4EBP2 | BG106477 | Hs.278712 | chr10q21-q22 |
| 224643_at | proline-rich coiled-coil 1 | PRRC1 | AL524045 | Hs.6614 | chr5q23.2 |
| 224642_at | forty-two-three domain containing 1 | FYTTD1 | BG291550 | Hs.277533 | chr3q29 |
| 224638_at | signal peptide peptidase 3 | UNQ1887 | AI338356 | Hs.21143 | chr12q24.31 |
| 224632_at | G patch domain containing 4 | GPATCH4 | BE794289 | Hs.446535 | chr1q22 |
| 224631_at | zinc finger protein 91 homolog (mouse) | ZFP91 | AA758013 | Hs.46366 | chr11q12 |
| 224629_at | Lectin, mannose-binding, 1 | LMAN1 | BF217539 | Hs.436593 | chr18q21.3-q22 |
| 224628_at | chromosome 2 open reading frame 30 | C2orf30 | AF131743 | Hs.438336 | chr2p16.3 |
| 224627_at | glucosidase, beta (bile acid) 2 | GBA2 | AB046825 | Hs.443134 | chr9p13.3 |
| 224626_at | solute carrier family 35, member A4 | SLC35A4 | BE618656 | Hs.519623 | chr5q31.3 |
| 224624_at | leucine rich repeat containing 8 family, member A | LRRC8A | BF030331 | Hs.173484 | chr9q34.11 |
| 224623_at | THO complex 3 /// similar to THO complex 3 | THOC3 | BE614567 | Hs.19574 | chr5q35.2 |
| 224622_at | TBC1 domain family, member 14 | TBC1D14 | AB037743 | Hs.72242 | chr4p16.1 |
| 224619_at | cancer susceptibility candidate 4 | CASC4 | BE904551 | Hs.323966 | chr15q15.3 |
| 224615_x_at | histocompatibility (minor) 13 | HM13 | AL110115 | Hs.444601 | chr20q11.21 |
| 224612_s_at | DnaJ (Hsp40) homolog, subfamily C, member 5 | DNAJC5 | AI935180 | Hs.165563 | chr20q13.33 |
| 224610_at | small nucleolar RNA host gene (non-protein coding) 1 | SNHG1 | AL530869 | Hs.433345 | chr11q13 |
| 224608_s_at | vacuolar protein sorting 25 homolog (S. cerevisiae) | VPS25 | AL528911 | Hs.500165 | chr17q21.2 |
| 224607_s_at | signal recognition particle 68kDa | SRP68 | BG398597 | Hs.273307 | chr17q25.1 |
| 224602_at | HCV F-transactivated protein 1 | LOC401152 | BF244081 | Hs.173705 | chr4q26 |
| 224589_at | X (inactive)-specific transcript | XIST | BF223193 | Hs.83623 | chrxq13.2 |
| 224580_at | solute carrier family 38, member 1 | SLC38A1 | BF515894 | Hs.132246 | chr12q13.11 |
| 224578_at | regulator of chromosome condensation 2 | RCC2 | AB040903 | Hs.380857 | chr1p36.13 |
| 224575_at | chromosome 3 open reading frame 10 | C3orf10 | BE868361 | Hs.421654 | chr3p25.3 |
| 224574_at | chromosome 17 open reading frame 49 /// similar to DNA segment, Chr 11, Brigham & Womens Genetics 0434 expressed | C17orf49 | AV759602 | Hs.206824 | chr17p13.1 |
| 224573_at | similar to DNA segment, Chr 11, Brigham & Womens Genetics 0434 expressed | MGC71993 | BE744389 |  | chr17p13.1 |
| 224572_s_at | interferon regulatory factor 2 binding protein 2 | IRF2BP2 | BG485163 | Hs.350268 | chr1q42.3 |
| 224562_at | WAS protein family, member 2 | WASF2 | AK025566 | Hs.446403 | chr1p36.11-p34.3 |
| 224559_at | metastasis associated lung adenocarcinoma transcript 1 (non-coding RNA) | MALAT1 | AF001540 | Hs.187199 | chr11q13.1 |
| 224535_s_at | mitochondrial ribosomal protein 63 /// mitochondrial ribosomal protein 63 | MRP63 | AB049957 | Hs.458367 | chr13p11.1-q11 |
| 224524_s_at | ankyrin repeat and SOCS box-containing 3 /// ankyrin repeat and SOCS box-containing 3 | ASB3 | BC006488 | Hs.40763 | chr2p16-p14 |
| 224523_s_at | chromosome 3 open reading frame 26 /// chromosome 3 open reading frame 26 | C3orf26 | BC006475 | Hs.128861 | chr3q12.1 |
| 224521_s_at | coiled-coil domain containing 77 /// coiled-coil domain containing 77 | CCDC77 | BC006444 | Hs.512781 | chr12p13.33 |
| 224518_s_at | zinc finger protein 559 /// zinc finger protein 559 | ZNF559 | BC006436 | Hs.512786 | chr19p13.2 |
| 224511_s_at | thioredoxin-like 5 /// thioredoxin-like 5 | TXNL5 | BC006405 | Hs.408236 | chr17p13.1 |
| 224509_s_at | reticulon 4 interacting protein 1 /// reticulon 4 interacting protein 1 | RTN4IP1 | BC006399 | Hs.155839 | chr6q21 |
| 224504_s_at | BUD13 homolog (yeast) /// BUD13 homolog (yeast) | BUD13 | BC006350 | Hs.39143 | chr11q23.3 |
| 224502_s_at | KIAA1191 /// KIAA1191 | KIAA1191 | BC006316 | Hs.8594 | chr5q35.2 |
| 224493_x_at | chromosome 18 open reading frame 45 /// chromosome 18 open reading frame 45 | C18orf45 | BC006280 |  |  |
| 224492_s_at | zinc finger protein 627 /// zinc finger protein 627 | ZNF627 | BC006279 | Hs.14831 | chr19p13.2 |
| 224486_s_at | chromosome 15 open reading frame 41 /// chromosome 15 open reading frame 41 | C15orf41 | BC006254 | Hs.48348 | chr15q14 |
| 224484_s_at | breast cancer metastasis-suppressor 1-like /// breast cancer metastasis-suppressor 1-like | BRMS1L | BC006250 | Hs.181035 | chr14q13.2 |
| 224480_s_at | lysophosphatidic acid acyltransferase theta /// lysophosphatidic acid acyltransferase theta | LPAAT-THETA | BC006236 | Hs.99196 | chr4q21.23 |
| 224479_s_at | mitochondrial ribosomal protein L45 /// mitochondrial ribosomal protein L45 | MRPL45 | BC006235 | Hs.19347 | chr17q21.2 |
| 224478_s_at | hypothetical protein MGC11257 /// hypothetical protein MGC11257 | MGC11257 | BC006224 | Hs.433208 | chr7p22.3 |
| 224474_x_at | SMEK homolog 2, suppressor of mek1 (Dictyostelium) /// SMEK homolog 2, suppressor of mek1 (Dictyostelium) | SMEK2 | BC006215 | Hs.130900 | chr2p16.1 |
| 224468_s_at | chromosome 19 open reading frame 48 /// chromosome 19 open reading frame 48 | C19orf48 | BC006151 | Hs.256301 | chr19q13.33 |
| 224467_s_at | programmed cell death 2-like /// programmed cell death 2-like | PDCD2L | BC006146 | Hs.363006 | chr19q13.11 |
| 224465_s_at | within bgcn homolog (Drosophila) /// within bgcn homolog (Drosophila) | WIBG | BC006135 | Hs.333524 | chr12q13.2 |
| 224453_s_at | ethanolamine kinase 1 /// ethanolamine kinase 1 | ETNK1 | BC006111 | Hs.120439 | chr12p12.1 |
| 224452_s_at | hypothetical protein LOC84792 /// hypothetical protein LOC84792 | MGC12966 | BC006110 | Hs.7980 | chr7p22.1 |
| 224450_s_at | RIO kinase 1 (yeast) /// RIO kinase 1 (yeast) | RIOK1 | BC006104 | Hs.437474 | chr6p24.3 |
| 224448_s_at | chromosome 6 open reading frame 125 /// chromosome 6 open reading frame 125 | C6orf125 | BC006007 | Hs.93235 | chr6p21.31 |
| 224447_s_at | chromosome 17 open reading frame 37 /// chromosome 17 open reading frame 37 | C17orf37 | BC006006 | Hs.333526 | chr17q12 |
| 224446_at | chromosome 12 open reading frame 31 /// chromosome 12 open reading frame 31 | C12orf31 | BC006002 | Hs.504820 | chr12q14.3 |
| 224444_s_at | chromosome 1 open reading frame 97 /// chromosome 1 open reading frame 97 | C1orf97 | BC005997 | Hs.132816 | chr1q32.3 |
| 224442_at | PHD finger protein 6 /// PHD finger protein 6 | PHF6 | BC005994 | Hs.356501 | chrxq26 |
| 224436_s_at | nipsnap homolog 3A (C. elegans) /// nipsnap homolog 3A (C. elegans) | NIPSNAP3A | BC005935 | Hs.24608 | chr9q31.1 |
| 224435_at | chromosome 10 open reading frame 58 /// chromosome 10 open reading frame 58 | C10orf58 | BC005871 | Hs.495628 | chr10q22.3 |
| 224434_s_at | mitogen-activated protein kinase organizer 1 /// mitogen-activated protein kinase organizer 1 | MORG1 | BC005870 | Hs.534592 | chr19p13.13 |
| 224430_s_at | mitochondrial translation optimization 1 homolog (S. cerevisiae) /// mitochondrial translation optimization 1 homolog (S. cerevisiae) | MTO1 | BC005808 | Hs.33979 | chr6q13 |
| 224428_s_at | cell division cycle associated 7 /// cell division cycle associated 7 | CDCA7 | AY029179 | Hs.435733 | chr2q31 |
| 224427_s_at | poly(A) polymerase gamma /// poly(A) polymerase gamma | PAPOLG | AY029162 | Hs.146123 | chr2p16.1 |
| 224415_s_at | histidine triad nucleotide binding protein 2 /// histidine triad nucleotide binding protein 2 | HINT2 | AF356515 | Hs.70573 | chr9p13.3 |
| 224413_s_at | TM2 domain containing 2 /// TM2 domain containing 2 | TM2D2 | AF353991 | Hs.7471 | chr8p11.23 |
| 224397_s_at | transmembrane and tetratricopeptide repeat containing 1 /// transmembrane and tetratricopeptide repeat containing 1 | TMTC1 | AF319520 | Hs.401954 | chr12p11.22 |
| 224392_s_at | opsin 3 (encephalopsin, panopsin) /// opsin 3 (encephalopsin, panopsin) | OPN3 | AF303588 | Hs.534448 | chr1q43 |
| 224391_s_at | sialic acid acetylesterase /// sialic acid acetylesterase | SIAE | AF303378 | Hs.10056 | chr11q24 |
| 224374_s_at | elastin microfibril interfacer 2 /// elastin microfibril interfacer 2 | EMILIN2 | AF270513 | Hs.270143 | chr18p11.3 |
| 224369_s_at | F-box protein 38 /// F-box protein 38 | FBXO38 | AF251055 | Hs.163825 | chr5q32 |
| 224367_at | brain expressed X-linked 2 /// brain expressed X-linked 2 | BEX2 | AF251053 | Hs.398989 | chrxq22 |
| 224366_s_at | RALBP1 associated Eps domain containing 1 /// RALBP1 associated Eps domain containing 1 | REPS1 | AF251052 | Hs.333141 | chr6q23.1-q24.1 |
| 224364_s_at | peptidylprolyl isomerase (cyclophilin)-like 3 /// peptidylprolyl isomerase (cyclophilin)-like 3 | PPIL3 | AF251049 | Hs.121076 | chr2q33.1 |
| 224352_s_at | cofilin 2 (muscle) /// cofilin 2 (muscle) | CFL2 | AF134802 | Hs.180141 | chr14q12 |
| 224336_s_at | dual specificity phosphatase 16 /// dual specificity phosphatase 16 | DUSP16 | AB052156 | Hs.20281 | chr12p13 |
| 224334_s_at | mitochondrial ribosomal protein L51 /// mitochondrial ribosomal protein L51 | MRPL51 | AB049959 | Hs.55847 | chr12p13.3-p13.1 |
| 224331_s_at | mitochondrial ribosomal protein L36 /// mitochondrial ribosomal protein L36 | MRPL36 | AB049654 | Hs.32196 | chr5p15.3 |
| 224330_s_at | mitochondrial ribosomal protein L27 /// mitochondrial ribosomal protein L27 | MRPL27 | AB049647 | Hs.7736 | chr17q21.3-q22 |
| 224326_s_at | polycomb group ring finger 6 /// polycomb group ring finger 6 | PCGF6 | AB047006 | Hs.335808 | chr10q24.33 |
| 224325_at | frizzled homolog 8 (Drosophila) /// frizzled homolog 8 (Drosophila) | FZD8 | AB043703 | Hs.302634 | chr10p11.21 |
| 224323_s_at | maestro /// maestro | MRO | AB042647 | Hs.131072 | chr18q21 |
| 224321_at | transmembrane protein with EGF-like and two follistatin-like domains 2 /// transmembrane protein with EGF-like and two follistatin-like domains 2 | TMEFF2 | AB004064 | Hs.22791 | chr2q32.3 |
| 224320_s_at | MCM8 minichromosome maintenance deficient 8 (S. cerevisiae) | MCM8 | BC005170 | Hs.528998 | chr20p12.3 |
| 224318_s_at | hypothetical protein FLJ10081 | FLJ10081 | AF311326 | Hs.337625 | chr2p12-p11.2 |
| 224316_at | potassium channel tetramerisation domain containing 9 | KCTD9 | AF130091 |  |  |
| 224315_at | DEAD (Asp-Glu-Ala-Asp) box polypeptide 20 | DDX20 | AL133598 | Hs.65412 | chr1p21.1-p13.2 |
| 224314_s_at | egl nine homolog 1 (C. elegans) | EGLN1 | AF277174 | Hs.130946 | chr1q42.1 |
| 224311_s_at | calcium binding protein 39 | CAB39 | AF151824 | Hs.433760 | chr2q37.1 |
| 224308_s_at | integrator complex subunit 2 | INTS2 | AL136800 | Hs.279646 | chr17q23.2 |
| 224302_s_at | mitochondrial ribosomal protein S36 | MRPS36 | AF271777 | Hs.408914 | chr5q13.2 |
| 224298_s_at | phosphoglycerate dehydrogenase like 1 | PHGDHL1 | BC004528 | Hs.355814 | chr13q32.3 |
| 224288_x_at | FKSG49 | FKSG49 | AF336882 |  |  |
| 224286_at | membrane frizzled-related protein | MFRP | AB055505 | Hs.512769 | chr11q23 |
| 224280_s_at | family with sequence similarity 54, member B | FAM54B | AL512766 | Hs.323396 | chr1p36.11 |
| 224232_s_at | px19-like protein | PX19 | AF112203 | Hs.442993 | chr5q35.3 |
| 224197_s_at | C1q and tumor necrosis factor related protein 1 | C1QTNF1 | AF232905 | Hs.201398 | chr17q25.3 |
| 224180_x_at | hypothetical protein LOC51057 | LOC51057 | AF131737 | Hs.414952 | chr2p15 |
| 224173_s_at | mitochondrial ribosomal protein L30 | MRPL30 | BC000217 | Hs.346736 | chr2q11.2 |
| 224170_s_at | tubby like protein 4 | TULP4 | AF288480 | Hs.102237 | chr6q25-q26 |
| 224164_at | tropomyosin 3 | TPM3 | AY004867 | Hs.178468 | chr1q21.2 |
| 224163_s_at | DNA methyltransferase 1 associated protein 1 | DMAP1 | AL136657 | Hs.8008 | chr1p34 |
| 224160_s_at | acyl-Coenzyme A dehydrogenase family, member 9 | ACAD9 | BC001817 | Hs.409209 | chr3q21.3 |
| 224151_s_at | adenylate kinase 3 | AK3 | AF183419 | Hs.43436 | chr9p24.1-p24.3 |
| 224150_s_at | centrosomal protein 70kDa | CEP70 | AF289495 | Hs.127217 | chr3q22-q23 |
| 224149_x_at | sarcolemma associated protein | SLMAP | AF304450 |  |  |
| 224130_s_at | steroid receptor RNA activator 1 | SRA1 | AF293026 | Hs.534416 | chr5q31.3 |
| 224129_s_at | dpy-30-like protein | LOC84661 | AF226998 | Hs.402525 | chr2p22.3 |
| 224097_s_at | F11 receptor | F11R | AF191495 | Hs.414880 | chr1q21.2-q21.3 |
| 224060_s_at | DPH5 homolog (S. cerevisiae) | DPH5 | AF157319 | Hs.484404 | chr1p21.2 |
| 224002_s_at | FK506 binding protein 7 | FKBP7 | AF100751 | Hs.410378 | chr2q31.2 |
| 223989_s_at | REX2, RNA exonuclease 2 homolog (S. cerevisiae) | REXO2 | BC003502 | Hs.7527 | chr11q23.1-q23.2 |
| 223983_s_at | chromosome 19 open reading frame 12 | C19orf12 | BC004957 | Hs.216485 | chr19q12 |
| 223980_s_at | SP110 nuclear body protein | SP110 | AF280095 | Hs.38125 | chr2q37.1 |
| 223978_s_at | cardiolipin synthase 1 | CRLS1 | AF241784 | Hs.170918 | chr20p13-p12.3 |
| 223959_at | dynein, axonemal, light chain 1 | DNAL1 | BC005343 | Hs.7086 | chr14q24.3 |
| 223931_s_at | checkpoint with forkhead and ring finger domains | CHFR | AF170724 | Hs.23794 | chr12q24.33 |
| 223917_s_at | solute carrier family 39 (zinc transporter), member 3 | SLC39A3 | BC000085 | Hs.30901 | chr19p13.3 |
| 223912_s_at | ceroid-lipofuscinosis, neuronal 8 (epilepsy, progressive with mental retardation) | CLN8 | AF123758 | Hs.127675 | chr8p23 |
| 223909_s_at | histone deacetylase 8 | HDAC8 | AF212246 | Hs.112272 | chrxq13 |
| 223907_s_at | PIN2-interacting protein 1 | PINX1 | AF205718 | Hs.400259 | chr8p23 |
| 223894_s_at | fused toes homolog (mouse) | FTS | BC001134 | Hs.380897 | chr16q12.2 |
| 223888_s_at | leucyl-tRNA synthetase | LARS | AF151026 | Hs.115568 | chr5q32 |
| 223882_at | chromosome 5 open reading frame 21 | C5orf21 | AL136630 | Hs.127788 | chr5q15 |
| 223879_s_at | oxidation resistance 1 | OXR1 | AF309387 | Hs.432398 | chr8q23 |
| 223871_x_at | similar to inhibitor of growth family, member 5 | LOC727773 | BC005370 | Hs.390538 | chr2q37.3 |
| 223854_at | protocadherin beta 10 | PCDHB10 | AF131761 | Hs.145256 | chr5q31 |
| 223849_s_at | Mov10, Moloney leukemia virus 10, homolog (mouse) | MOV10 | BC002548 | Hs.512586 | chr1p13.2 |
| 223847_s_at | endoplasmic reticulum-golgi intermediate compartment (ERGIC) 1 | ERGIC1 | AF267855 | Hs.519765 | chr5q35.1 |
| 223824_at | chromosome 10 open reading frame 59 | C10orf59 | BC005364 | Hs.274413 | chr10q23.31 |
| 223821_s_at | sushi domain containing 4 | SUSD4 | BC004888 | Hs.128038 | chr1q41-q42.11 |
| 223819_x_at | COMM domain containing 5 | COMMD5 | BC003055 | Hs.378713 | chr8q24-qter |
| 223814_at | tRNA nucleotidyl transferase, CCA-adding, 1 | TRNT1 | BC005184 | Hs.506382 |  |
| 223803_s_at | zinc finger, CCHC domain containing 10 | ZCCHC10 | BC005211 | Hs.29700 | chr5q31.1 |
| 223798_at | solute carrier family 41, member 2 | SLC41A2 | AL136828 | Hs.528692 | chr12q23.3 |
| 223797_at | hypothetical protein PRO2852 | PRO2852 | AF130079 |  |  |
| 223792_at | zinc finger protein 2 | ZNF2 | BC005068 | Hs.192285 | chr2q11.2 |
| 223784_at | transmembrane protein 27 | TMEM27 | AF229179 | Hs.129614 | chrxp22 |
| 223774_at | chromosome 1 open reading frame 79 | C1orf79 | AF277181 | Hs.528699 | chr1p35.3 |
| 223772_s_at | transmembrane protein 87A | TMEM87A | BC005335 | Hs.200692 | chr15q15.1 |
| 223758_s_at | general transcription factor IIH, polypeptide 2, 44kDa | GTF2H2 | BC005345 | Hs.191356 | chr5q12.2-q13.3 |
| 223746_at | serine/threonine kinase 4 | STK4 | BC005231 | Hs.35140 | chr20q11.2-q13.2 |
| 223742_at | mitochondrial ribosomal protein L4 | MRPL4 | BC000756 | Hs.279652 | chr19p13.2 |
| 223738_s_at | phosphoglucomutase 2 | PGM2 | AL136705 | Hs.23363 | chr4p14 |
| 223734_at | ovary-specific acidic protein | OSAP | AF329088 | Hs.154140 | chr4q31.1 |
| 223728_at | hypothetical protein MGC16385 /// hypothetical protein LOC728006 | MGC16385 | BC005105 | Hs.534616 | chr16q24.3 |
| 223714_at | zinc finger protein 256 | ZNF256 | BC001438 | Hs.288736 | chr19q13.43 |
| 223711_s_at | thymocyte nuclear protein 1 | THYN1 | AF182413 | Hs.13645 | chr11q25 |
| 223705_s_at | GC-rich promoter binding protein 1 | GPBP1 | BC000267 | Hs.71252 | chr5q11.2 |
| 223703_at | chromosome 10 open reading frame 11 | C10orf11 | AF267860 | Hs.160275 | chr10q22.2-q22.3 |
| 223700_at | meiotic nuclear divisions 1 homolog (S. cerevisiae) | MND1 | AY028916 | Hs.294088 | chr4q31.3 |
| 223697_x_at | chromosome 9 open reading frame 64 | C9orf64 | BC004407 | Hs.208914 | chr9q21.32 |
| 223686_at | thiamin pyrophosphokinase 1 | TPK1 | AB028138 | Hs.490454 | chr7q34-q35 |
| 223682_s_at | hypothetical protein MGC11102 | MGC11102 | BC005131 | Hs.425178 | chr11q13.1 |
| 223679_at | catenin (cadherin-associated protein), beta 1, 88kDa | CTNNB1 | AF130085 | Hs.410086 | chr3p21 |
| 223674_s_at | CDC42 small effector 1 | CDC42SE1 | AF286592 | Hs.22065 | chr1q21.2 |
| 223665_at | actin related protein M1 | ARPM1 | AB049117 | Hs.135411 | chr3q26.2 |
| 223664_x_at | BCL2-like 13 (apoptosis facilitator) | BCL2L13 | AF246665 | Hs.310922 | chr22q11 |
| 223658_at | potassium channel, subfamily K, member 6 | KCNK6 | AF134149 | Hs.240395 | chr19q13.1 |
| 223656_s_at | chromosome 1 open reading frame 91 | C1orf91 | BC004885 | Hs.272299 | chr1p36.11-p34.2 |
| 223652_at | arsenic (+3 oxidation state) methyltransferase | AS3MT | AF226730 | Hs.349396 | chr10q24.32 |
| 223651_x_at | cell division cycle 23 homolog (S. cerevisiae) | CDC23 | BC005258 | Hs.534403 | chr5q31 |
| 223649_s_at | solute carrier family 25, member 39 | SLC25A39 | AF317711 | Hs.237924 | chr17q12 |
| 223647_x_at | HscB iron-sulfur cluster co-chaperone homolog (E. coli) | HSCB | BC000004 | Hs.182898 | chr22q12.1 |
| 223639_s_at | zinc ribbon domain containing 1 | ZNRD1 | AF230337 | Hs.57813 | chr6p21.3 |
| 223638_at | neuroblastoma breakpoint family, member 3 | NBPF3 | AL136890 | Hs.325422 | chr1p36.12 |
| 223637_s_at | chromosome 11 open reading frame 56 | C11orf56 | AL136723 | Hs.8039 | chr11p15.4 |
| 223635_s_at | single stranded DNA binding protein 3 /// interleukin 17 receptor B | SSBP3 | BC003605 | Hs.288801 | chr1p32.3 |
| 223630_at | chromosome 7 open reading frame 13 | C7orf13 | AF063598 | Hs.124854 | chr7q36 |
| 223626_x_at | family with sequence similarity 14, member A | FAM14A | AF208232 | Hs.94695 | chr14q32.13 |
| 223624_at | AN1, ubiquitin-like, homolog (Xenopus laevis) | ANUBL1 | AF311324 | Hs.409813 | chr10q11.21 |
| 223622_s_at | hydroxypyruvate isomerase homolog (E. coli) | HYI | AF284751 | Hs.473855 | chr1p34.2 |
| 223620_at | G protein-coupled receptor 34 | GPR34 | AF039686 | Hs.29202 | chrxp11.4-p11.3 |
| 223619_x_at | peroxisomal trans-2-enoyl-CoA reductase | PECR | AF119841 |  |  |
| 223614_at | chromosome 8 open reading frame 57 | C8orf57 | AL136588 |  |  |
| 223611_s_at | ligand of numb-protein X 1 | LNX1 | AI738919 | Hs.407755 | chr4q12 |
| 223605_at | solute carrier family 25 (mitochondrial carrier), member 18 | SLC25A18 | AY008285 | Hs.282982 | chr22q11.2 |
| 223599_at | tripartite motif-containing 6 | TRIM6 | AF220030 | Hs.350518 | chr11p15.4 |
| 223595_at | transmembrane protein 133 | TMEM133 | AF247167 | Hs.44004 | chr11q22.1 |
| 223591_at | ring finger protein 135 | RNF135 | BC005084 | Hs.29874 | chr17q11.2 |
| 223590_at | zinc finger protein 700 | ZNF700 | AL136732 | Hs.398022 | chr19p13.2 |
| 223588_at | THAP domain containing, apoptosis associated protein 2 | THAP2 | AL136607 | Hs.245798 | chr12q21.1 |
| 223584_s_at | kelch repeat and BTB (POZ) domain containing 2 | KBTBD2 | BF000166 | Hs.20237 | chr7p14.3 |
| 223582_at | G protein-coupled receptor 98 | GPR98 | AF055084 | Hs.153692 | chr5q13 |
| 223576_at | chromosome 6 open reading frame 203 | C6orf203 | AF151064 | Hs.36069 | chr6q21 |
| 223569_at | phosphatidic acid phosphatase type 2 domain containing 1B | PPAPDC1B | AF212238 | Hs.437179 | chr8p12 |
| 223566_s_at | BCL6 co-repressor | BCOR | AF317391 | Hs.186424 | chrxp11.4 |
| 223555_at | gon-4-like (C. elegans) | GON4L | AL136565 | Hs.399846 | chr1q22 |
| 223548_at | chromosome 1 open reading frame 26 | C1orf26 | AF288392 | Hs.22133 | chr1q25 |
| 223542_at | ankyrin repeat domain 32 | ANKRD32 | AL136560 | Hs.91846 | chr5q15 |
| 223535_at | nudix (nucleoside diphosphate linked moiety X)-type motif 12 | NUDT12 | AL136592 | Hs.434289 | chr5q21.2 |
| 223533_at | leucine rich repeat containing 8 family, member C | LRRC8C | AL136919 | Hs.412836 | chr1p22.2 |
| 223531_x_at | G protein-coupled receptor 89A /// similar to G protein-coupled receptor 89 /// similar to G protein-coupled receptor 89 | GPR89A | AF151035 | Hs.504478 | chr1p36.13-q31.3 |
| 223528_s_at | methyltransferase 11 domain containing 1 /// similar to methyltransferase 11 domain containing 1 isoform 2 | METT11D1 | AF321002 | Hs.425446 | chr14q11.2 |
| 223527_s_at | cytidine and dCMP deaminase domain containing 1 | CDADC1 | AL138875 | Hs.388220 | chr13q14.2 |
| 223526_at | chromosome 18 open reading frame 21 | C18orf21 | AF277189 |  |  |
| 223524_s_at | transmembrane protein 108 | TMEM108 | BC000568 | Hs.280740 | chr3q21 |
| 223522_at | chromosome 9 open reading frame 45 | C9orf45 | AF251293 | Hs.21379 | chr9q33.3 |
| 223518_at | DNA fragmentation factor, 45kDa, alpha polypeptide | DFFA | AF087573 | Hs.484782 | chr1p36.3-p36.2 |
| 223515_s_at | coenzyme Q3 homolog, methyltransferase (S. cerevisiae) | COQ3 | AL136726 | Hs.299208 | chr6q16.3 |
| 223513_at | centromere protein J | CENPJ | AF139625 | Hs.434229 | chr13q12.12 |
| 223512_at | SAR1 gene homolog B (S. cerevisiae) | SAR1B | BE856242 | Hs.279582 | chr5q31.1 |
| 223511_at | chromosome 1 open reading frame 124 | C1orf124 | AL512744 | Hs.23971 | chr1q42.12-q43 |
| 223506_at | zinc finger CCCH-type containing 8 | ZC3H8 | AF334161 | Hs.418416 | chr2q13 |
| 223497_at | KIAA1411 | KIAA1411 | AL136820 | Hs.485713 | chr6q12-q13 |
| 223496_s_at | coiled-coil domain containing 8 | CCDC8 | AL136609 | Hs.97876 | chr19q13.32 |
| 223494_at | meningioma expressed antigen 5 (hyaluronidase) | MGEA5 | AF307332 | Hs.5734 | chr10q24.1-q24.3 |
| 223493_at | F-box protein 4 | FBXO4 | AF129534 | Hs.437345 | chr5p12 |
| 223491_at | COMM domain containing 2 | COMMD2 | BC001228 | Hs.432729 | chr3q25.1 |
| 223487_x_at | guanine nucleotide binding protein (G protein), beta polypeptide 4 | GNB4 | AW504458 | Hs.48695 | chr3q26.32 |
| 223482_at | transmembrane protein induced by tumor necrosis factor alpha | TMPIT | AF327923 | Hs.416916 | chr7q11.23 |
| 223480_s_at | mitochondrial ribosomal protein L47 | MRPL47 | AI189156 | Hs.283734 | chr3q26.33 |
| 223479_s_at | coiled-coil-helix-coiled-coil-helix domain containing 5 | CHCHD5 | BC004498 | Hs.375707 | chr2q13 |
| 223476_s_at | hypothetical protein FLJ38663 | FLJ38663 | AW007238 | Hs.130200 | chr12q24.31 |
| 223474_at | chromosome 14 open reading frame 4 | C14orf4 | AI932310 | Hs.179260 | chr14q24.3 |
| 223473_at | hypothetical protein MGC12972 | FKSG24 | BC005064 | Hs.13972 | chr19p13.11 |
| 223471_at | RAB3A interacting protein (rabin3) | RAB3IP | BC002556 | Hs.103267 | chr12q14.3 |
| 223467_at | RAS, dexamethasone-induced 1 | RASD1 | AF069506 | Hs.25829 | chr17p11.2 |
| 223466_x_at | collagen, type IV, alpha 3 (Goodpasture antigen) binding protein | COL4A3BP | BC000102 | Hs.21276 | chr5q13.3 |
| 223461_at | TBC1 domain family, member 7 | TBC1D7 | AF151073 | Hs.8645 | chr6p24.1 |
| 223457_at | coatomer protein complex, subunit gamma 2 | COPG2 | AB047847 | Hs.258811 | chr7q32 |
| 223452_s_at | DKFZP564J0863 protein | DKFZP564J0863 | AL117600 | Hs.356719 | chr11q13.1 |
| 223450_s_at | component of oligomeric golgi complex 3 | COG3 | AF332595 | Hs.105414 | chr13q14.13 |
| 223446_s_at | dystrobrevin binding protein 1 | DTNBP1 | AF061734 | Hs.43481 | chr6p22.3 |
| 223444_at | SUMO1/sentrin specific peptidase 7 | SENP7 | AL136599 | Hs.30443 | chr3q12 |
| 223443_s_at | hypothetical protein FLJ32065 | FLJ32065 | BC003669 | Hs.396447 | chr17q24.2 |
| 223442_at | nicolin 1 | NICN1 | BC005050 | Hs.191460 | chr3p21.31 |
| 223441_at | solute carrier family 17 (anion/sugar transporter), member 5 | SLC17A5 | AK026921 | Hs.117865 | chr6q14-q15 |
| 223440_at | chromosome 16 open reading frame 70 | C16orf70 | BC004556 | Hs.375206 | chr16q22.1 |
| 223439_at | NF-kappaB activating protein | NKAP | BC000940 | Hs.434870 | chrxq24 |
| 223438_s_at | peroxisome proliferator-activated receptor alpha | PPARA | BC004162 | Hs.534037 | chr22q13.31 |
| 223436_s_at | tRNA phosphotransferase 1 | TRPT1 | BC005133 | Hs.534584 | chr11q13.1 |
| 223435_s_at | protocadherin alpha 9 /// protocadherin alpha subfamily C, 2 /// protocadherin alpha subfamily C, 1 /// protocadherin alpha 13 /// protocadherin alpha 12 /// protocadherin alpha 11 /// protocadherin alpha 10 /// protocadherin alpha 8 /// protocadherin alp | PCDHA9 | AI268404 | Hs.247734 | chr5q31 |
| 223434_at | guanylate binding protein 3 | GBP3 | AL136680 | Hs.92287 | chr1p22.2 |
| 223433_at | chromosome 7 open reading frame 36 | C7orf36 | AF226046 | Hs.83313 | chr7p14.1 |
| 223431_at | cappuccino homolog (mouse) | CNO | BC001818 | Hs.7570 | chr4p16.1 |
| 223428_s_at | KIAA1160 protein | KIAA1160 | AI761808 | Hs.512661 | chr3q21.3 |
| 223427_s_at | erythrocyte membrane protein band 4.1 like 4B | EPB41L4B | AB032179 | Hs.207134 | chr9q31-q32 |
| 223424_s_at | zinc finger and SCAN domain containing 21 | ZSCAN21 | AL136865 | Hs.20082 | chr7q22.1 |
| 223422_s_at | Rho GTPase activating protein 24 | ARHGAP24 | AI743534 | Hs.444229 | chr4q21.23 |
| 223420_at | DnaJ (Hsp40) homolog, subfamily C, member 14 | DNAJC14 | AA156470 | Hs.375025 | chr12q13.2 |
| 223419_at | F-box and WD-40 domain protein 9 | FBXW9 | BC004290 | Hs.447945 | chr19p13.13 |
| 223417_at | RAD18 homolog (S. cerevisiae) | RAD18 | AF169796 | Hs.375684 | chr3p25-p24 |
| 223416_at | splicing factor 3B, 14 kDa subunit | SF3B14 | AF184213 | Hs.177861 | chr2pter-p25.1 |
| 223413_s_at | hypothetical protein FLJ20425 | LYAR | AW958593 | Hs.425427 | chr4p16.3 |
| 223412_at | kelch repeat and BTB (POZ) domain containing 7 | KBTBD7 | AL136782 | Hs.63841 | chr13q14.11 |
| 223402_at | dual specificity phosphatase 23 | DUSP23 | BC001140 | Hs.425801 | chr1q23.2 |
| 223401_at | chromosome 17 open reading frame 48 | C17orf48 | BC001294 | Hs.47668 | chr17p13.1 |
| 223400_s_at | polybromo 1 | PB1 | AF197569 | Hs.173220 | chr3p21 |
| 223398_at | chromosome 9 open reading frame 89 | C9orf89 | BC004500 | Hs.434213 | chr9q22.31 |
| 223397_s_at | nuclear import 7 homolog (S. cerevisiae) | NIP7 | AF161528 | Hs.501513 | chr16q22.1 |
| 223396_at | transmembrane protein 60 | TMEM60 | AF260336 | Hs.19025 | chr7q11.23 |
| 223394_at | SERTA domain containing 1 | SERTAD1 | BC002670 | Hs.269898 | chr19q13.1-q13.2 |
| 223392_s_at | teashirt family zinc finger 3 | TSHZ3 | BF510588 | Hs.278436 | chr19q12 |
| 223389_s_at | zinc finger protein 581 | ZNF581 | AF151023 | Hs.20768 | chr19q13.42 |
| 223387_at | zinc finger, FYVE domain containing 1 | ZFYVE1 | AB046809 | Hs.368384 | chr14q22-q24 |
| 223380_s_at | LATS, large tumor suppressor, homolog 2 (Drosophila) | LATS2 | AF207547 | Hs.78960 | chr13q11-q12 |
| 223376_s_at | brain protein I3 | BRI3 | AB055977 | Hs.410497 | chr7q21.3 |
| 223373_s_at | phospholipase A2, group XIIA | PLA2G12A | AF306567 | Hs.526406 | chr4q25 |
| 223369_at | chromosome 9 open reading frame 32 | C9orf32 | BC001396 | Hs.284250 | chr9q34.11 |
| 223367_at | Williams Beuren syndrome chromosome region 18 | WBSCR18 | BC005056 | Hs.534589 | chr7q11.23 |
| 223363_at | chromosome 7 open reading frame 48 | C7orf48 | BC004308 | Hs.446311 | chr7p22.3 |
| 223361_at | chromosome 6 open reading frame 115 | C6orf115 | AF116682 | Hs.238205 | chr6q24.1 |
| 223360_at | chromosome 21 open reading frame 56 | C21orf56 | AL136871 | Hs.381214 | chr21q22.3 |
| 223356_s_at | mitochondrial translational initiation factor 3 | MTIF3 | BG529919 | Hs.437583 | chr13q12.2 |
| 223353_at | HCCA2 protein | HCCA2 | AB028173 | Hs.19223 | chr11p15.5 |
| 223351_at | chromosome 17 open reading frame 80 | C17orf80 | N21028 |  |  |
| 223349_s_at | BCL2-related ovarian killer | BOK | BE614255 | Hs.293753 | chr2q37.3 |
| 223346_at | vacuolar protein sorting 18 homolog (S. cerevisiae) | VPS18 | AF308802 | Hs.23876 | chr15q14-q15 |
| 223342_at | ribonucleotide reductase M2 B (TP53 inducible) | RRM2B | AB036063 | Hs.512592 | chr8q23.1 |
| 223341_s_at | short coiled-coil protein | SCOC | AF330205 | Hs.286013 | chr4q31.1 |
| 223340_at | spastic paraplegia 3A (autosomal dominant) | SPG3A | AF131801 | Hs.241503 | chr14q21.3-q22.1 |
| 223339_at | ATPase inhibitory factor 1 | ATPIF1 | BC001867 | Hs.241336 | chr1p35.3 |
| 223336_s_at | RAB18, member RAS oncogene family | RAB18 | AL136734 | Hs.414357 | chr10p12.1 |
| 223335_at | transmembrane protein 69 | TMEM69 | AF151063 | Hs.184456 | chr1p34.1 |
| 223334_at | transmembrane protein 126A | TMEM126A | AL136941 | Hs.108338 | chr11q14.1 |
| 223332_x_at | ring finger protein 126 | RNF126 | BC001442 | Hs.69554 | chr19p13.3 |
| 223330_s_at | SGT1, suppressor of G2 allele of SKP1 (S. cerevisiae) | SUGT1 | BC000911 | Hs.107776 | chr13q14.3 |
| 223325_at | thioredoxin domain containing 11 | TXNDC11 | AF131780 | Hs.306811 | chr16p13.13 |
| 223324_s_at | transient receptor potential cation channel, subfamily M, member 7 | TRPM7 | AF346629 | Hs.143619 | chr15q21 |
| 223321_s_at | fibroblast growth factor receptor-like 1 | FGFRL1 | AF312678 | Hs.193326 | chr4p16 |
| 223320_s_at | ATP-binding cassette, sub-family B (MDR/TAP), member 10 | ABCB10 | AF277184 | Hs.1710 | chr1q42 |
| 223319_at | gephyrin | GPHN | AF272663 | Hs.13405 | chr14q23.3 |
| 223318_s_at | alkB, alkylation repair homolog 7 (E. coli) | ALKBH7 | BC004393 | Hs.111099 | chr19p13.3 |
| 223315_at | netrin 4 | NTN4 | AF278532 | Hs.102541 | chr12q22-q23 |
| 223313_s_at | melanoma antigen family D, 4 /// similar to melanoma antigen family D, 4 isoform 1 | MAGED4 | BC001207 | Hs.376347 | chrxp11 |
| 223312_at | chromosome 2 open reading frame 7 | C2orf7 | BC005069 | Hs.61268 | chr2p13.2 |
| 223311_s_at | metastasis associated 1 family, member 3 | MTA3 | BC004227 | Hs.435413 | chr2p21 |
| 223309_x_at | patatin-like phospholipase domain containing 8 | PNPLA8 | BG025248 | Hs.439046 | chr7q31 |
| 223308_s_at | WD repeat domain 5 | WDR5 | AL521101 | Hs.397638 | chr9q34 |
| 223306_at | emopamil binding protein-like | EBPL | AF243433 | Hs.433278 | chr13q12-q13 |
| 223305_at | HSPC244 | MGC13379 | AF151078 | Hs.26745 | chr11q13.1 |
| 223304_at | solute carrier family 37 (glycerol-3-phosphate transporter), member 3 | SLC37A3 | AL136583 | Hs.439590 | chr7q34 |
| 223300_s_at | coiled-coil domain containing 82 | CCDC82 | AI286012 | Hs.278944 | chr11q21 |
| 223299_at | SEC11 homolog C (S. cerevisiae) | SEC11C | AF212233 | Hs.45107 | chr18q21.32 |
| 223298_s_at | 5'-nucleotidase, cytosolic III | NT5C3 | AF312735 | Hs.415641 | chr7p14.3 |
| 223297_at | hypothetical protein MGC4268 | MGC4268 | BC004208 | Hs.444182 | chr2q21 |
| 223296_at | solute carrier family 25, member 33 | SLC25A33 | BC004991 | Hs.443826 | chr1p36.22 |
| 223294_at | chromosome X open reading frame 26 | CXorf26 | BC001220 | Hs.370100 | chrxq13.3 |
| 223289_s_at | ubiquitin specific peptidase 38 | USP38 | AF211481 | Hs.332841 | chr4q31.1 |
| 223284_at | N-acetyltransferase 14 | NAT14 | AB038651 | Hs.31854 | chr19q13.42 |
| 223282_at | teashirt family zinc finger 1 | TSHZ1 | W60810 | Hs.284217 | chr18q22.3 |
| 223279_s_at | uveal autoantigen with coiled-coil domains and ankyrin repeats | UACA | AF322916 | Hs.49753 | chr15q22-q24 |
| 223276_at | MSTP150 | MST150 | AF313413 | Hs.29444 | chr5q33.1 |
| 223275_at | protein arginine methyltransferase 6 | PRMT6 | BC002729 | Hs.26006 | chr1p13.3 |
| 223273_at | chromosome 14 open reading frame 142 | C14orf142 | AF277185 | Hs.20142 | chr14q32.13 |
| 223272_s_at | chromosome 1 open reading frame 57 | C1orf57 | BC005102 | Hs.16034 | chr1q42.2 |
| 223269_at | polymerase (RNA) III (DNA directed) polypeptide G (32kD)-like | POLR3GL | BC004355 | Hs.331667 | chr1q21.1 |
| 223267_at | RNA (guanine-9-) methyltransferase domain containing 1 | RG9MTD1 | AF226052 | Hs.57898 | chr3q12.3 |
| 223266_at | amyotrophic lateral sclerosis 2 (juvenile) chromosome region, candidate 2 | ALS2CR2 | AB038950 | Hs.259230 | chr2q33-q34 |
| 223265_at | SH3-binding domain protein 5-like | SH3BP5L | AL136569 | Hs.298573 | chr1q44 |
| 223264_at | mesoderm development candidate 1 | MESDC1 | BC001373 | Hs.290356 | chr15q13 |
| 223260_s_at | polymerase (DNA directed) kappa | POLK | AB027564 | Hs.135756 | chr5q13 |
| 223259_at | ORM1-like 3 (S. cerevisiae) | ORMDL3 | BC000638 | Hs.133167 | chr17q12-q21.1 |
| 223254_s_at | KIAA1333 | KIAA1333 | AA887053 | Hs.445887 | chr14q12 |
| 223253_at | ependymin related protein 1 (zebrafish) | EPDR1 | BC000686 | Hs.137635 | chr7p14.1 |
| 223251_s_at | ankyrin repeat domain 10 | ANKRD10 | BC001727 | Hs.164969 | chr13q34 |
| 223249_at | claudin 12 | CLDN12 | AL136770 | Hs.258576 | chr7q21 |
| 223248_at | hydroxysteroid dehydrogenase like 1 | HSDL1 | AK025626 | Hs.171937 | chr16q24.1 |
| 223247_at | mediator of RNA polymerase II transcription, subunit 10 homolog (NUT2, S. cerevisiae) | MED10 | BC003353 | Hs.220535 | chr5p15.31 |
| 223244_s_at | NADH dehydrogenase (ubiquinone) 1 alpha subcomplex, 12 | NDUFA12 | AF217092 | Hs.44163 | chr12q22 |
| 223241_at | sorting nexin 8 | SNX8 | AF121858 | Hs.12169 | chr7p22.3 |
| 223240_at | F-box protein 8 | FBXO8 | AF201932 | Hs.76917 | chr4q34.1 |
| 223239_at | chromosome 14 open reading frame 129 | C14orf129 | BC004818 |  |  |
| 223236_at | coiled-coil domain containing 55 | CCDC55 | AL136806 | Hs.374609 | chr17q11.2 |
| 223235_s_at | SPARC related modular calcium binding 2 | SMOC2 | AB014737 | Hs.22209 | chr6q27 |
| 223234_at | MAD2 mitotic arrest deficient-like 2 (yeast) | MAD2L2 | AF080398 | Hs.19400 | chr1p36 |
| 223231_at | TatD DNase domain containing 1 | TATDN1 | AF212250 | Hs.429857 | chr8q24.13 |
| 223230_at | PRP38 pre-mRNA processing factor 38 (yeast) domain containing A | PRPF38A | AL521713 | Hs.5301 | chr1p33-p32.1 |
| 223228_at | leucine zipper, down-regulated in cancer 1-like | LDOC1L | AL136553 | Hs.332795 | chr22q13.31 |
| 223227_at | Bardet-Biedl syndrome 2 | BBS2 | AF342736 | Hs.333738 | chr16q21 |
| 223225_s_at | SEH1-like (S. cerevisiae) | SEH1L | AI201534 | Hs.301048 | chr18p11.21 |
| 223223_at | ARV1 homolog (S. cerevisiae) | ARV1 | AF321442 | Hs.438645 | chr1q42.2 |
| 223222_at | solute carrier family 25 (mitochondrial deoxynucleotide carrier), member 19 | SLC25A19 | BC001075 | Hs.396222 | chr17q25.3 |
| 223221_at | SCO cytochrome oxidase deficient homolog 1 (yeast) | SCO1 | AF183424 | Hs.14511 | chr17p12-p13 |
| 223220_s_at | poly (ADP-ribose) polymerase family, member 9 | PARP9 | AF307338 | Hs.131315 | chr3q13-q21 |
| 223218_s_at | nuclear factor of kappa light polypeptide gene enhancer in B-cells inhibitor, zeta | NFKBIZ | AB037925 | Hs.390476 | chr3p12-q12 |
| 223215_s_at | chromosome 14 open reading frame 100 | C14orf100 | AF151047 | Hs.446850 | chr14q23.1 |
| 223213_s_at | zinc fingers and homeoboxes 1 | ZHX1 | AI123518 | Hs.12940 | chr8q24.13 |
| 223212_at | zinc finger, DHHC-type containing 16 | ZDHHC16 | BC004535 | Hs.76662 | chr10q24.1 |
| 223211_at | 2-hydroxyacyl-CoA lyase 1 | HACL1 | BC001627 |  |  |
| 223210_at | churchill domain containing 1 | CHURC1 | AF060510 | Hs.325531 | chr14q23.3 |
| 223209_s_at | selenoprotein S | SELS | AF328864 | Hs.32148 | chr15q26.3 |
| 223208_at | potassium channel tetramerisation domain containing 10 | KCTD10 | AF113208 | Hs.302746 | chr12q24.11 |
| 223206_s_at | NmrA-like family domain containing 1 | NMRAL1 | BC002927 | Hs.288969 | chr16p13.3 |
| 223205_s_at | l(3)mbt-like 2 (Drosophila) | L3MBTL2 | AI985709 | Hs.351141 | chr22q13.31-q13.33 |
| 223203_at | transmembrane protein 29 /// similar to transmembrane protein 29 | TMEM29 | BC000867 | Hs.6451 | chrxp11.22 |
| 223202_s_at | transmembrane protein 164 | TMEM164 | BC002716 | Hs.425072 | chrxq22.3 |
| 223199_at | MAP kinase interacting serine/threonine kinase 2 | MKNK2 | AA404592 | Hs.512094 | chr19p13.3 |
| 223197_s_at | SWI/SNF-related, matrix-associated actin-dependent regulator of chromatin, subfamily a, containing DEAD/H box 1 | SMARCAD1 | AI807204 | Hs.21356 | chr4q22-q23 |
| 223196_s_at | sestrin 2 | SESN2 | AL136551 | Hs.8026 | chr1p35.3 |
| 223194_s_at | chromosome 6 open reading frame 85 | C6orf85 | AL512737 | Hs.7734 | chr6p25.2 |
| 223192_at | solute carrier family 25, member 28 | SLC25A28 | AF267854 | Hs.326104 | chr10q23-q24 |
| 223189_x_at | myeloid/lymphoid or mixed-lineage leukemia 5 (trithorax homolog, Drosophila) | MLL5 | AW082219 | Hs.380021 | chr7q22.1 |
| 223186_at | ubiquitin-conjugating enzyme variant Kua | Kua | AB044550 |  | chr20q13.2 |
| 223181_at | chromosome 18 open reading frame 55 | C18orf55 | BC000892 | Hs.7922 | chr18q22.3 |
| 223179_at | yippee-like 3 (Drosophila) | YPEL3 | BC005009 | Hs.271599 | chr16p11.2 |
| 223178_s_at | 5'-nucleotidase domain containing 1 | NT5DC1 | AF245044 | Hs.348720 | chr6q22.1 |
| 223176_at | potassium channel tetramerisation domain containing 20 | KCTD20 | BC003697 | Hs.188757 | chr6p21.31 |
| 223175_s_at | fem-1 homolog a (C. elegans) | FEM1A | BC004988 | Hs.351747 | chr19p13.3 |
| 223174_at | BTB (POZ) domain containing 10 | BTBD10 | BC005071 |  |  |
| 223173_at | spinster | SPIN1 | AF212371 | Hs.379091 | chr16p11.2 |
| 223171_at | dymeclin | DYM | BC001252 | Hs.501125 | chr18q12-q21.1 |
| 223170_at | transmembrane protein 98 | TMEM98 | AF132000 | Hs.3447 | chr17q11.2 |
| 223168_at | ras homolog gene family, member U | RHOU | AL096776 | Hs.20252 | chr1q42.11-q42.3 |
| 223167_s_at | ubiquitin specific peptidase 25 | USP25 | AF170562 | Hs.186961 | chr21q11.2 |
| 223166_x_at | chromosome 9 open reading frame 86 | C9orf86 | BC002945 | Hs.528652 | chr9q34.3 |
| 223164_at | cerebral cavernous malformation 2 | CCM2 | BC004903 | Hs.9960 | chr7p13 |
| 223163_s_at | zinc finger, C3HC-type containing 1 | ZC3HC1 | BC000190 | Hs.13525 | chr7q32.2 |
| 223162_s_at | KIAA1147 | KIAA1147 | AF116707 | Hs.233044 | chr7q34 |
| 223158_s_at | NIMA (never in mitosis gene a)-related kinase 6 | NEK6 | BE616825 | Hs.387222 | chr9q33.3-q34.11 |
| 223157_at | chromosome 4 open reading frame 14 | C4orf14 | BC004894 | Hs.8715 | chr4q12 |
| 223156_at | mitochondrial ribosomal protein S23 | MRPS23 | BC000242 | Hs.5836 | chr17q22-q23 |
| 223155_at | haloacid dehalogenase-like hydrolase domain containing 2 | HDHD2 | AL136681 | Hs.380822 | chr18q21.1 |
| 223154_at | mitochondrial ribosomal protein L1 | MRPL1 | AF212225 | Hs.283693 | chr4q21.1 |
| 223152_at | protein phosphatase 1, regulatory (inhibitor) subunit 12C | PPP1R12C | AF312028 | Hs.437055 | chr19q13.42 |
| 223151_at | DCN1, defective in cullin neddylation 1, domain containing 5 (S. cerevisiae) | DCUN1D5 | BC004169 | Hs.74284 | chr11q22.3 |
| 223150_s_at | protein tyrosine phosphatase, non-receptor type 23 | PTPN23 | AF290614 | Hs.25524 | chr3p21.3 |
| 223148_at | phosphatidylinositol glycan anchor biosynthesis, class S | PIGS | BC001319 | Hs.414241 | chr17p13.2 |
| 223147_s_at | WD repeat domain 33 | WDR33 | AB044749 | Hs.516445 | chr2q14.3 |
| 223141_at | uridine-cytidine kinase 1 | UCK1 | AK022317 | Hs.9597 | chr9q34.13 |
| 223138_s_at | DEAH (Asp-Glu-Ala-His) box polypeptide 36 | DHX36 | AI937206 | Hs.9414 | chr3p13-q23 |
| 223136_at | androgen-induced 1 | AIG1 | AF151861 | Hs.390662 | chr6q24.2 |
| 223134_at | bobby sox homolog (Drosophila) | BBX | AI795970 | Hs.35380 | chr3q13.1 |
| 223124_s_at | chromosome 1 open reading frame 128 | C1orf128 | AF271784 | Hs.31819 | chr1p36.11 |
| 223120_at | fucosidase, alpha-L- 2, plasma | FUCA2 | BC003060 | Hs.17680 | chr6q24 |
| 223118_s_at | ubiquitin specific peptidase 47 | USP47 | AI123715 | Hs.441028 | chr11p15.3 |
| 223116_at | breast cancer anti-estrogen resistance 1 | BCAR1 | AF218451 | Hs.273219 | chr16q22-q23 |
| 223115_at | cofactor required for Sp1 transcriptional activation, subunit 6, 77kDa | CRSP6 | AK001674 | Hs.22630 | chr11q14 |
| 223114_at | coenzyme Q5 homolog, methyltransferase (S. cerevisiae) | COQ5 | BC004916 | Hs.17250 | chr12q24.31 |
| 223113_at | transmembrane protein 138 | TMEM138 | AF151030 | Hs.406530 | chr11q12.2 |
| 223111_x_at | AT rich interactive domain 4B (RBP1-like) | ARID4B | BF058966 | Hs.17428 | chr1q42.1-q43 |
| 223109_at | TruB pseudouridine (psi) synthase homolog 2 (E. coli) | TRUB2 | BC001457 | Hs.20159 | chr9q34.11 |
| 223107_s_at | zinc finger, CCHC domain containing 17 | ZCCHC17 | AF151077 | Hs.54971 | chr1p35.2 |
| 223106_at | transmembrane protein 14C | TMEM14C | BC002496 | Hs.30376 | chr6p24.1 |
| 223104_at | jagunal homolog 1 (Drosophila) | JAGN1 | AF212230 | Hs.24054 | chr3p25.2 |
| 223100_s_at | nudix (nucleoside diphosphate linked moiety X)-type motif 5 | NUDT5 | BC000025 | Hs.368481 | chr10p14-p13 |
| 223097_at | ADP-ribosylhydrolase like 2 | ADPRHL2 | AF212236 | Hs.18021 | chr1p34.3 |
| 223096_at | nucleolar protein NOP5/NOP58 | NOP5/NOP58 | AF161469 | Hs.119908 | chr2q33.1 |
| 223095_at | MARVEL domain containing 1 | MARVELD1 | BC004995 | Hs.209614 | chr10q24.2 |
| 223092_at | ankylosis, progressive homolog (mouse) | ANKH | AA854943 | Hs.156727 | chr5p15.1 |
| 223091_x_at | chromosome 2 open reading frame 33 | C2orf33 | AF258660 | Hs.105166 | chr2q36.3 |
| 223089_at | vezatin, adherens junctions transmembrane protein | VEZT | AI805297 | Hs.24135 | chr12q22 |
| 223084_s_at | cyclin D-type binding-protein 1 | CCNDBP1 | AF246144 | Hs.36794 | chr15q14-q15 |
| 223078_s_at | tropomodulin 3 (ubiquitous) | TMOD3 | AF177171 | Hs.22826 | chr15q21.1-q21.2 |
| 223076_s_at | NOL1/NOP2/Sun domain family, member 2 | NSUN2 | BC001041 | Hs.368537 | chr5p15.31 |
| 223073_at | hippocampus abundant transcript-like 1 | HIATL1 | AF255650 | Hs.494486 | chr9q22.32 |
| 223070_at | selenoprotein K | SELK | AF085359 | Hs.58471 | chr3p21.31 |
| 223067_at | hypothetical protein HSPC148 | HSPC148 | AF110775 | Hs.42743 | chr11q21 |
| 223066_at | SNAP-associated protein | SNAPAP | BC000761 | Hs.32018 | chr1q21.3 |
| 223065_s_at | STARD3 N-terminal like | STARD3NL | BC003074 | Hs.13467 | chr7p14-p13 |
| 223064_at | hypothetical protein LOC51255 | LOC51255 | AF151072 | Hs.11156 | chr2p11.2 |
| 223063_at | chromosome 1 open reading frame 198 | C1orf198 | BC004870 | Hs.26812 | chr1q42.13-q43 |
| 223061_at | chitinase domain containing 1 | CHID1 | AF212229 | Hs.193264 | chr11p15.5 |
| 223060_at | chromosome 14 open reading frame 119 | C14orf119 | AF061731 | Hs.303901 | chr14q11.2 |
| 223056_s_at | exportin 5 | XPO5 | AF298880 | Hs.203206 | chr6p21.1 |
| 223054_at | DnaJ (Hsp40) homolog, subfamily B, member 11 | DNAJB11 | BC001144 | Hs.317192 | chr3q27.3 |
| 223053_x_at | SSU72 RNA polymerase II CTD phosphatase homolog (S. cerevisiae) | SSU72 | AF277178 | Hs.30026 | chr1p36.33 |
| 223050_s_at | F-box and WD-40 domain protein 5 | FBXW5 | BC000850 | Hs.82023 | chr9q34.3 |
| 223048_at | hypothetical protein FLJ20487 | FLJ20487 | BC002331 | Hs.313247 | chr11q12.2 |
| 223044_at | solute carrier family 40 (iron-regulated transporter), member 1 | SLC40A1 | AL136944 | Hs.409875 | chr2q32 |
| 223043_at | transmembrane protein 85 | TMEM85 | AF151018 | Hs.250905 | chr15q14 |
| 223042_s_at | FUN14 domain containing 2 | FUNDC2 | BC000255 | Hs.356050 | chrxq28 |
| 223041_at | CD99 molecule-like 2 | CD99L2 | AL136580 | Hs.169388 | chrxq28 |
| 223040_at | N-acetyltransferase 5 | NAT5 | BC005181 | Hs.109253 | chr20p11.23 |
| 223039_at | chromosome 22 open reading frame 13 | C22orf13 | BC004144 | Hs.9850 | chr22q11.2 |
| 223037_at | PDZ domain containing 11 | PDZD11 | AF151061 | Hs.11042 | chrxq13.1 |
| 223033_s_at | SCY1-like 1 (S. cerevisiae) | SCYL1 | AF297709 | Hs.238839 | chr11q13 |
| 223031_s_at | TNF receptor-associated factor 7 | TRAF7 | AL136921 | Hs.534588 | chr16p13.3 |
| 223027_at | sorting nexin 9 | SNX9 | BF972871 | Hs.7905 | chr6q25.1-q26 |
| 223026_s_at | vacuolar protein sorting 29 homolog (S. cerevisiae) | VPS29 | AF201946 | Hs.445394 | chr12q24 |
| 223024_at | adaptor-related protein complex 1, mu 1 subunit | AP1M1 | AL562950 | Hs.71040 | chr19p13.12 |
| 223023_at | blocked early in transport 1 homolog (S. cerevisiae)-like | BET1L | BC000688 | Hs.414418 | chr11p15.5 |
| 223021_x_at | chromosome 6 open reading frame 55 | C6orf55 | BF241590 | Hs.267288 | chr6q24.1 |
| 223020_at | CLPTM1-like | CLPTM1L | AB045223 | Hs.444673 | chr5pter-p15.3 |
| 223018_at | NIN1/RPN12 binding protein 1 homolog (S. cerevisiae) | NOB1 | BC000050 | Hs.3566 | chr16q22.3 |
| 223017_at | thioredoxin domain containing 12 (endoplasmic reticulum) | TXNDC12 | AF131758 | Hs.241489 | chr1p32.3 |
| 223016_x_at | zinc finger, RAN-binding domain containing 2 | ZRANB2 | AF065392 | Hs.194718 | chr1p31 |
| 223015_at | eukaryotic translation initiation factor 2A, 65kDa | EIF2A | AF212241 | Hs.378808 | chr3q25.1 |
| 223014_at | ubiquitin-conjugating enzyme E2R 2 | UBE2R2 | BC004862 | Hs.11184 | chr9p13.3 |
| 223009_at | chromosome 11 open reading frame 59 | C11orf59 | BC001706 | Hs.421219 | chr11q13.4 |
| 223004_s_at | chromosome 3 open reading frame 1 | C3orf1 | AF210057 | Hs.210036 | chr3q13.33 |
| 223003_at | chromosome 19 open reading frame 43 | C19orf43 | AF061732 | Hs.239894 | chr19p13.13 |
| 223001_at | DC2 protein | DC2 | AF201937 | Hs.103180 | chr4q25 |
| 222999_s_at | cyclin L2 /// similar to Cyclin-L2 (Paneth cell-enhanced expression protein) | CCNL2 | AF251294 | Hs.143601 | chr1p36.33 |
| 222998_at | MAF1 homolog (S. cerevisiae) | MAF1 | AL136937 | Hs.19673 | chr8q24.3 |
| 222997_s_at | mitochondrial ribosomal protein S21 | MRPS21 | BC004566 | Hs.405880 | chr1q21 |
| 222996_s_at | CXXC finger 5 | CXXC5 | BC002490 | Hs.356509 | chr5q31.2 |
| 222993_at | mitochondrial ribosomal protein L37 | MRPL37 | AF325707 | Hs.4209 | chr1p32.1 |
| 222992_s_at | NADH dehydrogenase (ubiquinone) 1 beta subcomplex, 9, 22kDa | NDUFB9 | AF261090 | Hs.15977 | chr8q13.3 |
| 222989_s_at | ubiquilin 1 | UBQLN1 | NM_013438 | Hs.9589 | chr9q22 |
| 222987_s_at | transmembrane protein 9 | TMEM9 | NM_016456 | Hs.181444 | chr1q41 |
| 222986_s_at | scotin | SCOTIN | BC001463 | Hs.414579 | chr3p21.31 |
| 222985_at | tyrosine 3-monooxygenase/tryptophan 5-monooxygenase activation protein, gamma polypeptide | YWHAG | AB024334 | Hs.25001 | chr7q11.23 |
| 222983_s_at | poly(A) binding protein interacting protein 2 | PAIP2 | BC001716 | Hs.396644 | chr5q31.2 |
| 222980_at | RAB10, member RAS oncogene family | RAB10 | AL136650 | Hs.236494 | chr2p23.3 |
| 222979_s_at | surfeit 4 | SURF4 | AF078866 | Hs.284296 | chr9q34.2 |
| 222955_s_at | family with sequence similarity 45, member B /// family with sequence similarity 45, member A /// similar to family with sequence similarity 45, member A | FAM45B | AF168713 | Hs.434241 | chr10q25 /// chrxq25 |
| 222931_s_at | threonine synthase-like 1 (bacterial) | THNSL1 | AI809864 | Hs.183171 | chr10p12.1 |
| 222918_at | RAB9B, member RAS oncogene family | RAB9B | AL139228 |  |  |
| 222916_s_at | high density lipoprotein binding protein (vigilin) | HDLBP | AF116718 | Hs.427152 | chr2q37 |
| 222907_x_at | transmembrane protein 50B | TMEM50B | BC000569 | Hs.433668 | chr21q22.11 |
| 222906_at | feline leukemia virus subgroup C cellular receptor | FLVCR | AK001419 | Hs.497698 | chr1q32.3 |
| 222903_s_at | cytoplasmic polyadenylation element binding protein 1 | CPEB1 | AF329403 | Hs.137064 | chr15q25.2 |
| 222893_s_at | chromosome 1 open reading frame 82 | C1orf82 | AI609064 | Hs.444421 | chr1p22.1 |
| 222890_at | coiled-coil domain containing 113 | CCDC113 | BG054922 | Hs.11614 | chr16q21 |
| 222889_at | DNA cross-link repair 1B (PSO2 homolog, S. cerevisiae) | DCLRE1B | AI703304 | Hs.115660 | chr1p13.2 |
| 222887_s_at | transmembrane protein 127 | TMEM127 | AA034018 | Hs.355708 | chr2q11.2 |
| 222877_at | Neuropilin 2 | NRP2 | AK024680 | Hs.368746 | chr2q33.3 |
| 222875_at | DEAH (Asp-Glu-Ala-His) box polypeptide 33 | DHX33 | AI720923 | Hs.250456 | chr17p13.2 |
| 222866_s_at | chromosome 14 open reading frame 58 | C14orf58 | AW001026 | Hs.267566 | chr14q24.3 |
| 222863_at | zinc finger and BTB domain containing 10 | ZBTB10 | BG483802 | Hs.436991 | chr8q13-q21.1 |
| 222854_s_at | gem (nuclear organelle) associated protein 8 | GEMIN8 | AA044921 | Hs.528647 | chrxp22.2 |
| 222852_at | chromosome 10 open reading frame 88 | C10orf88 | AI339606 |  |  |
| 222851_at | zinc finger protein 654 | ZNF654 | AL120354 | Hs.27595 | chr3p11.1 |
| 222849_s_at | secernin 3 | SCRN3 | AI306487 | Hs.20999 | chr2q31.1 |
| 222848_at | centromere protein K | CENPK | BC005400 | Hs.164018 | chr5p15.2-q12.3 |
| 222843_at | fidgetin-like 1 | FIGNL1 | AK023411 | Hs.137516 | chr7p12.2 |
| 222837_s_at | NMDA receptor regulated 1 | NARG1 | AF327722 | Hs.518994 | chr4q31.1 |
| 222825_at | OTU domain containing 6B | OTUD6B | BF435752 | Hs.30532 | chr8q21.3 |
| 222820_at | trinucleotide repeat containing 6C | TNRC6C | AW005818 | Hs.80618 | chr17q25.3 |
| 222819_at | CTP synthase II | CTPS2 | AK025654 | Hs.414156 | chrxp22 |
| 222817_at | hydroxy-delta-5-steroid dehydrogenase, 3 beta- and steroid delta-isomerase 7 | HSD3B7 | BC004929 | Hs.23628 | chr16p12-p11.2 |
| 222815_at | ring finger protein 12 | RNF12 | BE966018 | Hs.163432 | chrxq13-q21 |
| 222811_at | hypothetical protein FLJ11171 | FLJ11171 | AK023183 | Hs.72782 | chr16q22.2 |
| 222803_at | phosphoribosyl transferase domain containing 1 | PRTFDC1 | AI871620 | Hs.22835 | chr10p12.1 |
| 222788_s_at | round spermatid basic protein 1 | RSBN1 | BE888593 | Hs.425559 | chr1p13.2 |
| 222787_s_at | transmembrane protein 106B | TMEM106B | AV705186 | Hs.396358 | chr7p21.3 |
| 222781_s_at | chromosome 9 open reading frame 40 | C9orf40 | BF590861 | Hs.264363 | chr9q21.13 |
| 222779_s_at | chromosome 17 open reading frame 85 | C17orf85 | AA706815 | Hs.120963 | chr17p13.2 |
| 222778_s_at | Wolf-Hirschhorn syndrome candidate 1 | WHSC1 | AW024870 | Hs.110457 | chr4p16.3 |
| 222775_s_at | mitochondrial ribosomal protein L35 | MRPL35 | AK026220 | Hs.433439 | chr2p11.2 |
| 222774_s_at | neuropilin (NRP) and tolloid (TLL)-like 2 | NETO2 | AI335263 | Hs.6823 | chr16q11 |
| 222772_at | myelin expression factor 2 | MYEF2 | BG179854 | Hs.533916 | chr15q21.1 |
| 222768_s_at | CGI-09 protein | CGI-09 | BE897074 | Hs.128791 | chr20p12.3 |
| 222765_x_at | ESF1, nucleolar pre-rRNA processing protein, homolog (S. cerevisiae) | ESF1 | AL161659 | Hs.369284 | chr20p12.1 |
| 222752_s_at | chromosome 1 open reading frame 75 | C1orf75 | AK024066 | Hs.30318 | chr1q32.3 |
| 222750_s_at | steroid 5 alpha-reductase 2-like | SRD5A2L | BC002480 | Hs.39311 | chr4q12 |
| 222749_at | suppressor of fused homolog (Drosophila) | SUFU | AF159447 | Hs.21431 | chr10q24.32 |
| 222747_s_at | sex comb on midleg-like 1 (Drosophila) | SCML1 | BF001786 | Hs.109655 | chrxp22.2-p22.1 |
| 222741_s_at | chromosome 6 open reading frame 64 | C6orf64 | AI761426 | Hs.58382 | chr6p21.2 |
| 222736_s_at | transmembrane protein 38B | TMEM38B | BC000049 | Hs.411925 | chr9q31.2 |
| 222732_at | tripartite motif-containing 39 | TRIM39 | BF514859 | Hs.413493 | chr6p21.3 |
| 222730_s_at | zinc finger, DHHC-type containing 2 | ZDHHC2 | AI814257 | Hs.292871 | chr8p21.3-p22 |
| 222726_s_at | exocyst complex component 5 | EXOC5 | BF696912 | Hs.365863 | chr14q22.3 |
| 222718_at | transmembrane protein 8 (five membrane-spanning domains) | TMEM8 | BC004276 | Hs.288940 | chr16p13.3 |
| 222713_s_at | Fanconi anemia, complementation group F | FANCF | AF181995 | Hs.65328 | chr11p15 |
| 222699_s_at | pleckstrin homology domain containing, family F (with FYVE domain) member 2 | PLEKHF2 | BF439250 | Hs.29724 | chr8q22.1 |
| 222697_s_at | abhydrolase domain containing 10 | ABHD10 | AI521709 | Hs.266514 | chr3q13.2 |
| 222694_at | hypothetical protein MGC2752 | MGC2752 | AI015612 | Hs.7845 | chr19q13.43 |
| 222691_at | solute carrier family 35, member B3 | SLC35B3 | AL355815 | Hs.285847 | chr6p24.3 |
| 222689_at | phytoceramidase, alkaline | PHCA | N51263 | Hs.23862 | chr11q13.5 |
| 222684_s_at | nucleolar protein 10 | NOL10 | BG031619 | Hs.235498 | chr2p25.1 |
| 222683_at | ring finger protein 20 | RNF20 | AK022532 | Hs.168095 | chr9q22 |
| 222679_s_at | DCN1, defective in cullin neddylation 1, domain containing 1 (S. cerevisiae) | DCUN1D1 | AW468880 | Hs.104613 | chr3q26.3 |
| 222673_x_at | transmembrane protein 57 /// family with sequence similarity 122B | TMEM57 | AI582192 | Hs.404706 | chrxq26.3 |
| 222667_s_at | ash1 (absent, small, or homeotic)-like (Drosophila) | ASH1L | AI806500 | Hs.445445 | chr1q22 |
| 222666_s_at | RNA terminal phosphate cyclase-like 1 | RCL1 | BC001025 | Hs.113052 | chr9p24.1-p23 |
| 222665_at | family with sequence similarity 82, member B /// similar to Protein FAM82B | FAM82B | AK000672 | Hs.349119 | chr8q21.3 |
| 222659_at | importin 11 | IPO11 | AK001696 | Hs.441043 | chr5q12.1 |
| 222658_s_at | aprataxin | APTX | BC001628 | Hs.444529 | chr9p13.3 |
| 222653_at | pyridoxamine 5'-phosphate oxidase | PNPO | AA005137 | Hs.327335 | chr17q21.32 |
| 222651_s_at | trichorhinophalangeal syndrome I | TRPS1 | BF701166 | Hs.130469 | chr8q24.12 |
| 222650_s_at | SLC2A4 regulator | SLC2A4RG | BE898559 | Hs.254837 | chr20q13.33 |
| 222649_at | exportin 4 | XPO4 | BF968638 | Hs.311097 | chr13q11 |
| 222645_s_at | potassium channel tetramerisation domain containing 5 | KCTD5 | AA872593 | Hs.61960 | chr16p13.3 |
| 222642_s_at | transmembrane protein 33 | TMEM33 | BC000948 | Hs.31082 | chr4p13 |
| 222640_at | DNA (cytosine-5-)-methyltransferase 3 alpha | DNMT3A | N26002 | Hs.241565 | chr2p23 |
| 222638_s_at | similar to CG11699-PA | LOC729515 | BF968686 | Hs.157212 | chr6q25.3 |
| 222636_at | mediator of RNA polymerase II transcription, subunit 28 homolog (S. cerevisiae) | MED28 | AF317680 | Hs.33032 | chr4p16 |
| 222632_s_at | leucine zipper transcription factor-like 1 | LZTFL1 | AA843132 | Hs.30824 | chr3p21.3 |
| 222631_at | phosphatidylinositol 4-kinase type 2 beta | PI4K2B | AI862887 | Hs.443733 | chr4p15.2 |
| 222630_at | regulatory factor X domain containing 2 | RFXDC2 | BF740077 | Hs.126908 | chr15q21.3 |
| 222628_s_at | REV1 homolog (S. cerevisiae) | REV1 | AA195405 | Hs.443077 | chr2q11.1-q11.2 |
| 222626_at | RNA binding motif protein 26 | RBM26 | T79937 | Hs.408487 | chr13q31.1 |
| 222624_s_at | zinc finger protein 639 | ZNF639 | AA224199 | Hs.22879 | chr3q26.32 |
| 222622_at | hypothetical protein LOC283871 | LOC283871 | BG284709 |  | chr16p13.3 |
| 222612_at | Paraspeckle component 1 | PSPC1 | N32583 | Hs.16364 | chr13q12.11 |
| 222610_s_at | S100P binding protein | S100PBP | T81452 | Hs.369253 | chr1p35.1 |
| 222609_s_at | exosome component 1 | EXOSC1 | AL582889 | Hs.14415 | chr10q24 |
| 222608_s_at | anillin, actin binding protein | ANLN | AK023208 | Hs.62180 | chr7p15-p14 |
| 222603_at | KIAA1815 | KIAA1815 | AL136980 | Hs.87128 | chr9p24 |
| 222598_s_at | neuron navigator 2 /// similar to neuron navigator 2 isoform 2 | NAV2 | AK001892 | Hs.407934 | chr11p15.1 |
| 222594_s_at | spermatogenesis associated, serine-rich 2 | SPATS2 | AL583538 | Hs.146679 | chr12q13.12 |
| 222586_s_at | oxysterol binding protein-like 11 | OSBPL11 | AI884890 | Hs.61260 | chr3q21 |
| 222584_at | misato homolog 1 (Drosophila) /// similar to misato | MSTO1 | AL573591 | Hs.279763 | chr1q22 |
| 222582_at | protein kinase, AMP-activated, gamma 2 non-catalytic subunit | PRKAG2 | AF087875 | Hs.511620 | chr7q35-q36 |
| 222581_at | xenotropic and polytropic retrovirus receptor | XPR1 | AF089744 | Hs.227656 | chr1q25.1 |
| 222578_s_at | ubiquitin-activating enzyme E1-domain containing 1 | UBE1DC1 | AW516242 | Hs.170737 | chr3q22.1 |
| 222576_s_at | eukaryotic translation initiation factor 2C, 1 | EIF2C1 | AW071829 | Hs.309452 | chr1p35-p34 |
| 222574_s_at | DEAH (Asp-Glu-Ala-His) box polypeptide 40 | DHX40 | BF431360 | Hs.29403 | chr17q23.2 |
| 222569_at | UDP-glucose ceramide glucosyltransferase-like 1 | UGCGL1 | AU153746 | Hs.105794 | chr2q14.3 |
| 222566_at | suppressor of variegation 4-20 homolog 1 (Drosophila) | SUV420H1 | AA056099 | Hs.442630 | chr11q13.2 |
| 222565_s_at | protein kinase D3 | PRKD3 | BF978541 | Hs.434387 | chr2p21 |
| 222562_s_at | tankyrase, TRF1-interacting ankyrin-related ADP-ribose polymerase 2 | TNKS2 | BF060683 | Hs.280776 | chr10q23.3 |
| 222558_at | hypothetical protein FLJ10656 | P15RS | T79568 | Hs.202217 | chr18q12.2 |
| 222557_at | stathmin-like 3 | STMN3 | AL353715 | Hs.285753 | chr20q13.3 |
| 222551_s_at | chromosome 8 open reading frame 33 | C8orf33 | AI197841 | Hs.169615 | chr8q24.3 |
| 222549_at | Claudin 1 | CLDN1 | AF101051 | Hs.7327 | chr3q28-q29 |
| 222541_at | remodeling and spacing factor 1 | RSF1 | AA173588 | Hs.20509 | chr11q13.5 |
| 222538_s_at | adaptor protein containing pH domain, PTB domain and leucine zipper motif 1 | APPL | AW467472 | Hs.27413 | chr3p21.1-p14.3 |
| 222527_s_at | RNA binding motif protein 22 | RBM22 | AL538762 | Hs.202023 | chr5q33.1 |
| 222526_at | GATA zinc finger domain containing 2A | GATAD2A | AK024670 | Hs.118964 | chr19p13.11 |
| 222525_s_at | coiled-coil domain containing 25 | CCDC25 | AU160632 | Hs.445512 | chr8p21.1 |
| 222521_x_at | NADH dehydrogenase (ubiquinone) 1, subcomplex unknown, 2, 14.5kDa | NDUFC2 | AL040789 | Hs.407860 | chr11q14.1 |
| 222518_at | ADP-ribosylation factor guanine nucleotide-exchange factor 2 (brefeldin A-inhibited) | ARFGEF2 | BF525399 | Hs.310323 | chr20q13.13 |
| 222516_at | adaptor-related protein complex 3, mu 1 subunit | AP3M1 | AA700485 | Hs.298442 | chr10q22.2 |
| 222510_s_at | makorin, ring finger protein, 2 | MKRN2 | AI809203 | Hs.279474 | chr3p25 |
| 222507_s_at | TMEM9 domain family, member B | TMEM9B | AI188389 | Hs.389439 | chr11p15.3 |
| 222505_at | limb region 1 homolog (mouse) | LMBR1 | BF510801 | Hs.534558 | chr7q36 |
| 222500_at | peptidylprolyl isomerase (cyclophilin)-like 1 | PPIL1 | BC003048 | Hs.27693 | chr6p21.1 |
| 222499_at | mitochondrial ribosomal protein S16 | MRPS16 | BE964789 | Hs.180312 | chr10q22.1 |
| 222495_at | chromosome 1 open reading frame 119 /// similar to K07F5.15 | C1orf119 | AV712912 | Hs.82933 | chr1p13.3 |
| 222492_at | pyridoxal (pyridoxine, vitamin B6) kinase | PDXK | AW262867 | Hs.458335 | chr21q22.3 |
| 222490_at | polymerase (RNA) III (DNA directed) polypeptide E (80kD) | POLR3E | AK023160 | Hs.355714 | chr16p12.1 |
| 222489_s_at | Werner helicase interacting protein 1 | WRNIP1 | AA621580 | Hs.236828 | chr6p25.2 |
| 222486_s_at | ADAM metallopeptidase with thrombospondin type 1 motif, 1 | ADAMTS1 | AF060152 | Hs.8230 | chr21q21.2 |
| 222483_at | EF-hand domain family, member D2 | EFHD2 | AW664179 | Hs.301342 | chr1p36.21 |
| 222479_s_at | dynein, cytoplasmic 1, light intermediate chain 1 | DYNC1LI1 | AK001081 | Hs.351916 | chr3p23 |
| 222478_at | vacuolar protein sorting 36 homolog (yeast) | VPS36 | AL576924 | Hs.109520 | chr13q14.3 |
| 222475_at | SAP30 binding protein | SAP30BP | AK023157 | Hs.27299 | chr17q25.1 |
| 222471_s_at | potassium channel modulatory factor 1 | KCMF1 | AI743396 | Hs.190595 | chr2p11.2 |
| 222469_s_at | toll interacting protein | TOLLIP | AL136835 | Hs.25413 | chr11p |
| 222466_s_at | mitochondrial ribosomal protein L42 | MRPL42 | AL136659 | Hs.331202 | chr12q22 |
| 222458_s_at | chromosome 1 open reading frame 108 | C1orf108 | AI205764 | Hs.23767 | chr1p34.3 |
| 222456_s_at | LIM domain and actin binding 1 | LIMA1 | BF197289 | Hs.10706 | chr12q13 |
| 222453_at | cytochrome b reductase 1 | CYBRD1 | AL136693 | Hs.31297 | chr2q31.1 |
| 222452_s_at | GC-rich promoter binding protein 1-like 1 | GPBP1L1 | AA741071 | Hs.169854 | chr1p34.1 |
| 222451_s_at | zinc finger, DHHC-type containing 9 | ZDHHC9 | BC003128 | Hs.274351 | chr9 |
| 222450_at | transmembrane, prostate androgen induced RNA | TMEPAI | AL035541 | Hs.83883 | chr20q13.31-q13.33 |
| 222447_at | methyltransferase like 9 | METTL9 | BC000195 | Hs.279583 | chr16p13-p12 |
| 222439_s_at | thyroid hormone receptor associated protein 3 | THRAP3 | BE967048 | Hs.250113 | chr1p34.3 |
| 222438_at | mediator of RNA polymerase II transcription, subunit 4 homolog (S. cerevisiae) | MED4 | AK001934 | Hs.181112 | chr13q14.2 |
| 222429_at | drebrin-like | DBNL | AF077353 | Hs.183373 | chr7p13 |
| 222425_s_at | polymerase (DNA-directed), delta interacting protein 2 | POLDIP2 | AW151250 | Hs.241543 | chr17q11.2 |
| 222422_s_at | Nedd4 family interacting protein 1 | NDFIP1 | AW167859 | Hs.9788 | chr5q31.3 |
| 222418_s_at | transmembrane protein 43 | TMEM43 | AA115485 | Hs.130330 | chr3p25.1 |
| 222407_s_at | zinc finger protein 106 homolog (mouse) | ZFP106 | AI493587 | Hs.188199 | chr15q15.1 |
| 222403_at | mitochondrial carrier homolog 2 (C. elegans) | MTCH2 | AV756532 | Hs.279609 | chr11p11.2 |
| 222402_at | proteasome maturation protein | POMP | BC003390 | Hs.268742 | chr13q12.3 |
| 222398_s_at | elongation factor Tu GTP binding domain containing 2 | EFTUD2 | BC002360 | Hs.151787 | chr17q21.31 |
| 222394_at | programmed cell death 6 interacting protein | PDCD6IP | BG484789 | Hs.9663 | chr3p23 |
| 222385_x_at | Sec61 alpha 1 subunit (S. cerevisiae) | SEC61A1 | AF346602 | Hs.306079 | chr3q21.3 |
| 222382_x_at | nucleoporin 205kDa | NUP205 | AW206115 | Hs.413636 | chr7q33 |
| 222366_at | Activity-dependent neuroprotector | ADNP | W86781 |  |  |
| 222316_at | Vesicle docking protein p115 | VDP | AW973253 |  |  |
| 222311_s_at | splicing factor, arginine/serine-rich 15 | SFRS15 | AA648521 | Hs.17255 | chr21q22.1 |
| 222309_at | Chromosome 6 open reading frame 62 | C6orf62 | AW972292 |  |  |
| 222301_at | Chromosome 1 open reading frame 61 | C1orf61 | BF530257 | Hs.380027 | chr1q22 |
| 222297_x_at | similar to ribosomal protein L18 | LOC390612 | AV738806 |  |  |
| 222293_at | immunoglobulin superfamily, member 4C | IGSF4C | AW204383 | Hs.370984 | chr19q13.31 |
| 222286_at | small nuclear RNA activating complex, polypeptide 3, 50kDa | SNAPC3 | R43279 | Hs.380092 | chr9p22.3 |
| 222279_at | hypothetical protein FLJ35429 | RP3-377H14.5 | AI669379 |  |  |
| 222266_at | Chromosome 19 open reading frame 2 | C19orf2 | BF796940 |  |  |
| 222263_at | solute carrier family 35, member E1 | SLC35E1 | BE904333 | Hs.134074 | chr19p13.11 |
| 222258_s_at | SH3-domain binding protein 4 | SH3BP4 | AF015043 | Hs.17667 | chr2q37.1-q37.2 |
| 222252_x_at | ubiquilin 4 | UBQLN4 | AK023354 |  |  |
| 222251_s_at | glucocorticoid modulatory element binding protein 2 | GMEB2 | AL133646 | Hs.28906 | chr20q13.33 |
| 222237_s_at | zinc finger protein 228 | ZNF228 | AC084239 | Hs.48589 | chr19q13.2 |
| 222235_s_at | chondroitin sulfate GalNAcT-2 | GALNACT-2 | AL139812 | Hs.189590 | chr10q11.21 |
| 222231_s_at | leucine rich repeat containing 59 | LRRC59 | AK025328 | Hs.370927 | chr17q21.33 |
| 222230_s_at | actin-related protein 10 homolog (S. cerevisiae) | ACTR10 | AK022248 | Hs.248569 | chr14q23.1 |
| 222229_x_at | similar to 60S ribosomal protein L26 | LOC392501 | AL121871 | Hs.406682 | chr17p13 |
| 222228_s_at | alkB, alkylation repair homolog 4 (E. coli) | ALKBH4 | AK026097 | Hs.531248 | chr7q22.1 |
| 222221_x_at | EH-domain containing 1 | EHD1 | AY007161 | Hs.155119 | chr11q13 |
| 222212_s_at | LAG1 homolog, ceramide synthase 2 (S. cerevisiae) | LASS2 | AK001105 | Hs.285976 | chr1q21.2 |
| 222209_s_at | transmembrane protein 135 | TMEM135 | AK000684 | Hs.183887 | chr11q14.2 |
| 222207_x_at | similar to Williams Beuren syndrome chromosome region 19 | LOC441258 | AK024602 | Hs.380443 |  |
| 222206_s_at | nicalin homolog (zebrafish) | NCLN | AA781143 | Hs.24983 | chr19p13.3 |
| 222203_s_at | retinol dehydrogenase 14 (all-trans/9-cis/11-cis) | RDH14 | AK023625 | Hs.288880 | chr2p24.2 |
| 222201_s_at | CASP8 associated protein 2 | CASP8AP2 | AB037736 | Hs.122843 | chr6q15 |
| 222200_s_at | BSD domain containing 1 | BSDC1 | AK021440 | Hs.353454 | chr1p35.1 |
| 222199_s_at | bridging integrator 3 | BIN3 | AK001289 | Hs.375560 | chr8p21.3 |
| 222196_at | hypothetical protein LOC286434 /// hypothetical protein LOC728146 /// hypothetical protein LOC729137 | LOC286434 | AK000470 | Hs.535223 | chrxp22.33 |
| 222193_at | chromosome 2 open reading frame 43 | C2orf43 | AK021672 | Hs.63300 | chr2p24.1 |
| 222190_s_at | chromosome 16 open reading frame 58 | C16orf58 | BC000822 | Hs.9003 | chr16p11.2 |
| 222183_x_at | CDNA FLJ35189 fis, clone PLACE6016210 /// SNW domain containing 1 | SNW1 | AL390153 | Hs.178728 /// Hs.375834 | chr19p13.3 /// chr14q24.3 |
| 222175_s_at | PC2 (positive cofactor 2, multiprotein complex) glutamine/Q-rich-associated protein | PCQAP | AK000003 | Hs.410347 | chr22q11.2 |
| 222169_x_at | SH2 domain containing 3A | SH2D3A | N71739 | Hs.439645 | chr19p13.3 |
| 222165_x_at | chromosome 9 open reading frame 16 | C9orf16 | AK022885 | Hs.409585 | chr9q34.1 |
| 222158_s_at | chromosome 1 open reading frame 121 | C1orf121 | AF229834 | Hs.211473 | chr1q44 |
| 222157_s_at | WD repeat domain 48 | WDR48 | AB040882 | Hs.109778 | chr3p21.33 |
| 222155_s_at | G protein-coupled receptor 172A | GPR172A | AK021918 | Hs.6459 | chr8q24.3 |
| 222150_s_at | hypothetical protein LOC54103 | LOC54103 | AK026747 | Hs.186649 | chr7q11.23 |
| 222143_s_at | myotubularin related protein 14 | MTMR14 | AY007098 | Hs.271931 | chr3p26 |
| 222138_s_at | WD repeat domain 13 | WDR13 | AF158978 | Hs.12142 | chrxp11.23 |
| 222132_s_at | multiple substrate lipid kinase | MULK | AJ278150 | Hs.260238 | chr7q34 |
| 222131_x_at | ras homolog gene family, member T2 | RHOT2 | BC004327 | Hs.444178 | chr16p13.3 |
| 222130_s_at | FtsJ homolog 2 (E. coli) | FTSJ2 | AK024635 | Hs.279877 | chr7p22 |
| 222129_at | Chromosome 2 open reading frame 17 | C2orf17 | AK026155 | Hs.22412 | chr2q35 |
| 222127_s_at | exocyst complex component 1 | EXOC1 | AK023461 | Hs.221377 | chr4q12 |
| 222126_at | HIV-1 Rev binding protein-like | HRBL | AI247494 | Hs.278502 | chr7q22.1 /// chr7q22 |
| 222125_s_at | hypoxia-inducible factor prolyl 4-hydroxylase | PH-4 | BC000580 | Hs.271224 | chr3p21.31 |
| 222118_at | centromere protein N | CENPN | AK023669 | Hs.283532 | chr16q23.2 |
| 222115_x_at | cytokine-like nuclear factor n-pac | N-PAC | BC003693 | Hs.8750 | chr16p13.3 |
| 222113_s_at | epidermal growth factor receptor pathway substrate 15-like 1 | EPS15L1 | AV710549 | Hs.292072 | chr19p13.11 |
| 222109_at | guanine nucleotide binding protein-like 3 (nucleolar)-like | GNL3L | AA558583 | Hs.438583 | chrxp11.22 |
| 222108_at | adhesion molecule with Ig-like domain 2 | AMIGO2 | AC004010 | Hs.121520 | chr12q13.11 |
| 222106_at | prion protein 2 (dublet) | PRND | AL133396 | Hs.406696 | chr20pter-p12 |
| 222105_s_at | NFKB inhibitor interacting Ras-like 2 | NKIRAS2 | AA452565 | Hs.532590 | chr17q21.2 |
| 222104_x_at | general transcription factor IIH, polypeptide 3, 34kDa | GTF2H3 | AI569458 | Hs.30724 | chr12q24.31 |
| 222102_at | glutathione S-transferase A3 | GSTA3 | NM_000847 | Hs.102484 | chr6p12.1 |
| 222101_s_at | dachsous 1 (Drosophila) | DCHS1 | BF222893 | Hs.410314 | chr11p15.4 |
| 222077_s_at | Rac GTPase activating protein 1 | RACGAP1 | AU153848 | Hs.23900 | chr12q13.12 |
| 222071_s_at | solute carrier organic anion transporter family, member 4C1 | SLCO4C1 | BE552428 | Hs.127648 | chr5q21.2 |
| 222067_x_at | histone cluster 1, H2bd | HIST1H2BD | AL353759 | Hs.130853 | chr6p21.3 |
| 222064_s_at | alanyl-tRNA synthetase domain containing 1 | AARSD1 | AI093187 | Hs.317403 | chr17q21.31 |
| 222052_at | chromosome 19 open reading frame 54 | C19orf54 | AA001552 | Hs.59839 | chr19q13.2 |
| 222043_at | clusterin | CLU | AI982754 | Hs.436657 | chr8p21-p12 |
| 222041_at | DPH1 homolog (S. cerevisiae) /// candidate tumor suppressor in ovarian cancer 2 | DPH1 | BG235929 | Hs.298399 | chr17p13.3 |
| 222036_s_at | Solute carrier family 12 (potassium/chloride transporters), member 4 | SLC12A4 | AI859865 | Hs.460184 | chr8q11.2 |
| 222034_at | Guanine nucleotide binding protein (G protein), beta polypeptide 2-like 1 | GNB2L1 | AA443762 | Hs.5662 | chr5q35.3 |
| 222028_at | zinc finger protein 45 | ZNF45 | AI967981 | Hs.381285 | chr19q13.2 |
| 222027_at | Nuclear casein kinase and cyclin-dependent kinase substrate 1 | NUCKS1 | AW515443 |  |  |
| 222026_at | RNA binding motif (RNP1, RRM) protein 3 | RBM3 | BF437591 |  |  |
| 222025_s_at | 5-oxoprolinase (ATP-hydrolysing) | OPLAH | AI991887 | Hs.305882 | chr8q24.3 |
| 222021_x_at | succinate dehydrogenase complex, subunit A, flavoprotein pseudogene 1 | SDHALP1 | AI348006 | Hs.440475 | chr5p15 |
| 222010_at | t-complex 1 | TCP1 | BF224073 | Hs.487054 | chr6q25-q27 |
| 222006_at | Leucine zipper-EF-hand containing transmembrane protein 1 | LETM1 | AI359368 | Hs.1420 | chr4p16.3 |
| 222001_x_at | family with sequence similarity 91, member A2 | FAM91A2 | AI160126 | Hs.456577 | chr1q21.1 |
| 221994_at | PDZ and LIM domain 5 | PDLIM5 | AA196325 | Hs.154103 | chr4q22 |
| 221989_at | ribosomal protein L10 | RPL10 | AW057781 | Hs.77091 | chrxq28 |
| 221988_at | Cofactor required for Sp1 transcriptional activation, subunit 7, 70kDa | CRSP7 | AA463853 | Hs.356467 | chr19p13.11 |
| 221974_at | Prader-Willi syndrome chromosome region 1 | PWCR1 | AW770748 | Hs.48375 | chr15q11.2 |
| 221971_x_at | centaurin, gamma-like family, member 1 /// centaurin, gamma-like family, member 6 /// centaurin, gamma-like family, member 9 pseudogene /// centaurin, gamma-like family, member 7 /// centaurin, gamma-like family, member 2 | CTGLF1 | BE672818 | Hs.446716 | chr10q11.21 |
| 221970_s_at | nucleolar protein 11 | NOL11 | AU158148 | Hs.26761 | chr17q24.2 |
| 221964_at | tubby like protein 3 | TULP3 | AI591305 | Hs.437046 | chr12p13.3 |
| 221957_at | pyruvate dehydrogenase kinase, isozyme 3 | PDK3 | BF939522 | Hs.193124 | chrxp22.11 |
| 221954_at | Chromosome 20 open reading frame 111 | C20orf111 | AA160474 |  |  |
| 221951_at | transmembrane protein 80 | TMEM80 | AI739035 | Hs.448664 | chr11p15.5 |
| 221950_at | empty spiracles homolog 2 (Drosophila) | EMX2 | AI478455 | Hs.202095 | chr10q26.1 |
| 221949_at | hypothetical protein LOC222070 | LOC222070 | AW006953 |  | chr7p13 |
| 221939_at | Yip1 domain family, member 2 | YIPF2 | AL529396 | Hs.323213 | chr19p13.2 |
| 221935_s_at | chromosome 3 open reading frame 64 | C3orf64 | AK023140 | Hs.5997 | chr3p14.1 |
| 221934_s_at | DALR anticodon binding domain containing 3 | DALRD3 | BF941492 | Hs.506231 | chr3p21.31 |
| 221933_at | neuroligin 4, X-linked | NLGN4X | AI338338 | Hs.21107 | chrxp22.32-p22.31 |
| 221932_s_at | glutaredoxin 5 homolog (S. cerevisiae) | GLRX5 | AA133341 | Hs.294083 | chr14q32.13 |
| 221927_s_at | abhydrolase domain containing 11 | ABHD11 | AI923458 | Hs.182476 | chr7q11.23 |
| 221924_at | zinc finger, MIZ-type containing 2 | ZMIZ2 | AW444969 | Hs.77978 | chr7p13 |
| 221920_s_at | solute carrier family 25, member 37 | SLC25A37 | BE677761 | Hs.283716 | chr8p21.2 |
| 221918_at | PCTAIRE protein kinase 2 | PCTK2 | AI742210 | Hs.258536 | chr12q23.1 |
| 221914_at | synapsin I | SYN1 | H19843 | Hs.225936 | chrxp11.23 |
| 221904_at | chromosome 3 open reading frame 40 | C3orf40 | AI141670 | Hs.436847 | chr3q27.1 |
| 221898_at | podoplanin | PDPN | AU154455 | Hs.468675 | chr1p36.21 |
| 221893_s_at | aarF domain containing kinase 2 | ADCK2 | N32831 | Hs.210397 | chr7q32-q34 |
| 221887_s_at | deafness, autosomal recessive 31 | DFNB31 | BE045998 | Hs.93836 | chr9q32-q34 |
| 221873_at | zinc finger protein 143 | ZNF143 | AW162015 | Hs.374355 | chr11p15.4 |
| 221870_at | EH-domain containing 2 | EHD2 | AI417917 | Hs.325650 | chr19q13.3 |
| 221865_at | chromosome 9 open reading frame 91 | C9orf91 | BF969986 | Hs.170226 | chr9q32 |
| 221864_at | transmembrane protein 142C | TMEM142C | AW517464 | Hs.333488 | chr16p11.2 |
| 221858_at | TBC1 domain family, member 12 | TBC1D12 | N34407 | Hs.100960 | chr10q23.33 |
| 221853_s_at | NODAL modulator 1 /// NODAL modulator 2 /// NODAL modulator 3 | NOMO1 | N39536 | Hs.374735 | chr16p12.3 /// chr16p13.11 /// chr16p13 |
| 221845_s_at | ClpB caseinolytic peptidase B homolog (E. coli) | CLPB | AI655698 | Hs.334045 | chr11q13.4 |
| 221843_s_at | KIAA1609 | KIAA1609 | AA195017 | Hs.14449 | chr16q24.1 |
| 221841_s_at | Kruppel-like factor 4 (gut) | KLF4 | BF514079 | Hs.376206 | chr9q31 |
| 221840_at | protein tyrosine phosphatase, receptor type, E | PTPRE | AA775177 | Hs.437980 | chr10q26 |
| 221830_at | RAP2A, member of RAS oncogene family | RAP2A | AI302106 | Hs.48554 | chr13q34 |
| 221826_at | angel homolog 2 (Drosophila) | ANGEL2 | BE671941 | Hs.157078 | chr1q32.3 |
| 221824_s_at | membrane-associated ring finger (C3HC4) 8 | 08-mars | AA770170 | Hs.288156 | chr10q11.21 |
| 221823_at | chromosome 5 open reading frame 30 | C5orf30 | AL565741 | Hs.25925 | chr5q21.1 |
| 221821_s_at | chromosome 12 open reading frame 41 | C12orf41 | AK022732 | Hs.268189 | chr12q13.11 |
| 221820_s_at | MYST histone acetyltransferase 1 | MYST1 | AK024102 | Hs.42343 | chr16p11.2 |
| 221817_at | dolichyl pyrophosphate phosphatase 1 | DOLPP1 | AI684664 | Hs.21701 | chr9q34.1 |
| 221813_at | F-box protein 42 | FBXO42 | AI129395 | Hs.62767 | chr1p36.23-p36.11 |
| 221809_at | RAN binding protein 10 | RANBP10 | AB040897 | Hs.6343 | chr16q22.1 |
| 221808_at | RAB9, member RAS oncogene family | RAB9 | NM_004251 | Hs.444327 | chrxp22.2 |
| 221803_s_at | nuclear receptor binding factor 2 | NRBF2 | AA883074 | Hs.449628 | chr10q21.3 |
| 221802_s_at | KIAA1598 | KIAA1598 | AU157109 | Hs.98002 | chr10q25.3 |
| 221800_s_at | chromosome 17 open reading frame 70 | C17orf70 | AA551370 | Hs.313905 | chr17q25.3 |
| 221799_at | chondroitin sulfate glucuronyltransferase | CSGlcA-T | AB037823 | Hs.86392 | chr7q36.1 |
| 221797_at | hypothetical protein LOC339229 | LOC339229 | AY007126 | Hs.449864 | chr17q25.3 |
| 221791_s_at | coiled-coil domain containing 72 | CCDC72 | BG167522 | Hs.356440 | chr3p21.31 |
| 221786_at | chromosome 6 open reading frame 120 | C6orf120 | BF197222 | Hs.435933 | chr6q27 |
| 221780_s_at | DEAD (Asp-Glu-Ala-Asp) box polypeptide 27 | DDX27 | AF336851 | Hs.65234 | chr20q13.13 |
| 221779_at | MICAL-like 1 | MICALL1 | BC001090 | Hs.8535 | chr22q13.1-q13.2 |
| 221773_at | ELK3, ETS-domain protein (SRF accessory protein 2) | ELK3 | AW575374 | Hs.288555 | chr12q23 |
| 221771_s_at | M-phase phosphoprotein, mpp8 | HSMPP8 | BC003542 | Hs.411347 | chr13q12.11 |
| 221770_at | ribulose-5-phosphate-3-epimerase | RPE | BE964473 | Hs.282260 | chr2q32-q33.3 |
| 221768_at | Splicing factor proline/glutamine-rich (polypyrimidine tract binding protein associated) | SFPQ | AV705803 | Hs.180610 | chr1p34.3 |
| 221766_s_at | family with sequence similarity 46, member A | FAM46A | AW246673 | Hs.10784 | chr6q14 |
| 221764_at | chromosome 19 open reading frame 22 | C19orf22 | AL574186 | Hs.388956 | chr19p13.3 |
| 221762_s_at | chromosome 20 open reading frame 67 | C20orf67 | AL162458 | Hs.272814 | chr20q13.12 |
| 221761_at | adenylosuccinate synthase | ADSS | AA628948 | Hs.90011 | chr1cen-q12 |
| 221758_at | armadillo repeat containing 6 | ARMC6 | BC003700 | Hs.77876 | chr19p13.11 |
| 221754_s_at | coronin, actin binding protein, 1B | CORO1B | AI341234 | Hs.6191 | chr11q13.2 |
| 221753_at | slingshot homolog 1 (Drosophila) | SSH1 | AI651213 | Hs.60377 | chr12q24.11 |
| 221751_at | Solute carrier family 2 (facilitated glucose transporter), member 3 pseudogene 1 | SLC2A3P1 | AL565516 | Hs.388400 | chr5q34 |
| 221747_at | Tensin 1 /// Tensin 1 | TNS1 | AL046979 | Hs.439442 | chr2q35-q36 |
| 221746_at | ubiquitin-like 4A | UBL4A | BE543027 | Hs.76480 | chrxq28 |
| 221741_s_at | YTH domain family, member 1 | YTHDF1 | AL096828 | Hs.11747 | chr20q13.33 |
| 221738_at | KIAA1219 /// KIAA1219 | KIAA1219 | BG236163 | Hs.348929 | chr20q11.23 |
| 221737_at | guanine nucleotide binding protein (G protein) alpha 12 | GNA12 | AK024696 | Hs.182874 | chr7p22-p21 |
| 221732_at | calcium activated nucleotidase 1 | CANT1 | AK026161 | Hs.8859 | chr17q25.3 |
| 221729_at | collagen, type V, alpha 2 | COL5A2 | AL575735 | Hs.283393 | chr2q14-q32 |
| 221712_s_at | WD repeat domain 74 /// WD repeat domain 74 | WDR74 | BC006351 | Hs.415220 | chr11q12.3 |
| 221711_s_at | HSPC142 protein /// HSPC142 protein | HSPC142 | BC006244 | Hs.190722 | chr19p13.11 |
| 221708_s_at | unc-45 homolog A (C. elegans) /// unc-45 homolog A (C. elegans) | UNC45A | BC006214 | Hs.389461 | chr15q26.1 |
| 221706_s_at | uncharacterized hematopoietic stem/progenitor cells protein MDS032 /// uncharacterized hematopoietic stem/progenitor cells protein MDS032 | MDS032 | BC006005 | Hs.16187 | chr19p13.11 |
| 221705_s_at | suppressor of IKK epsilon /// suppressor of IKK epsilon | SIKE | BC005934 | Hs.24808 | chr1p13.1 |
| 221704_s_at | vacuolar protein sorting 37 homolog B (S. cerevisiae) /// vacuolar protein sorting 37 homolog B (S. cerevisiae) | VPS37B | BC005882 | Hs.77870 | chr12q24.31 |
| 221700_s_at | ubiquitin A-52 residue ribosomal protein fusion product 1 /// ubiquitin A-52 residue ribosomal protein fusion product 1 | UBA52 | AF348700 | Hs.5308 | chr19p13.1-p12 |
| 221699_s_at | DEAD (Asp-Glu-Ala-Asp) box polypeptide 50 /// DEAD (Asp-Glu-Ala-Asp) box polypeptide 50 | DDX50 | AF334103 | Hs.396766 | chr10q22.1 |
| 221695_s_at | mitogen-activated protein kinase kinase kinase 2 /// mitogen-activated protein kinase kinase kinase 2 | MAP3K2 | AF239798 | Hs.28827 | chr2q14.3 |
| 221692_s_at | mitochondrial ribosomal protein L34 /// mitochondrial ribosomal protein L34 | MRPL34 | AB049652 | Hs.238808 | chr19p13.1 |
| 221689_s_at | phosphatidylinositol glycan anchor biosynthesis, class P | PIGP | AB035745 | Hs.127721 | chr21q22.2 |
| 221688_s_at | IMP3, U3 small nucleolar ribonucleoprotein, homolog (yeast) | IMP3 | AL136913 | Hs.6118 | chr15q24 |
| 221685_s_at | coiled-coil domain containing 99 | CCDC99 | AF269167 | Hs.32471 | chr5q35.1 |
| 221683_s_at | centrosomal protein 290kDa | CEP290 | AF317887 | Hs.150444 | chr12q21.33 |
| 221679_s_at | abhydrolase domain containing 6 | ABHD6 | AF225418 | Hs.39056 | chr3p14.3 |
| 221677_s_at | downstream neighbor of SON | DONSON | AF232674 | Hs.17834 | chr21q22.1 |
| 221676_s_at | coronin, actin binding protein, 1C | CORO1C | BC002342 | Hs.17377 | chr12q24.1 |
| 221673_s_at | casein kinase 1, gamma 1 | CSNK1G1 | AB042563 | Hs.405789 | chr15q22.1-q22.31 |
| 221671_x_at | immunoglobulin kappa constant /// immunoglobulin kappa variable 1-5 /// immunoglobulin kappa variable 2-24 | IGKC | M63438 | Hs.377975 | chr2p12 |
| 221669_s_at | acyl-Coenzyme A dehydrogenase family, member 8 | ACAD8 | BC001964 | Hs.14791 | chr11q25 |
| 221652_s_at | chromosome 12 open reading frame 11 | C12orf11 | AF274950 | Hs.22595 | chr12p11.23 |
| 221649_s_at | peter pan homolog (Drosophila) | PPAN | BC000535 | Hs.14468 | chr19p13 |
| 221647_s_at | resistance to inhibitors of cholinesterase 8 homolog A (C. elegans) | RIC8A | AL136935 | Hs.19306 | chr11p15.5 |
| 221643_s_at | arginine-glutamic acid dipeptide (RE) repeats | RERE | AF016005 | Hs.194369 | chr1p36.1-p36.2 |
| 221641_s_at | acyl-CoA thioesterase 9 | ACOT9 | AF241787 | Hs.298885 | chrxp22.11 |
| 221637_s_at | chromosome 11 open reading frame 48 | C11orf48 | BC001434 | Hs.9061 | chr11q12.3 |
| 221636_s_at | MOCO sulphurase C-terminal domain containing 2 | MOSC2 | AL136931 | Hs.4932 | chr1q41 |
| 221629_x_at | chromosome 8 open reading frame 30A | C8orf30A | AF151022 | Hs.300224 | chr8q24.3 |
| 221626_at | zinc finger protein 506 | ZNF506 | AL136548 | Hs.515282 | chr19p13.11 |
| 221622_s_at | transmembrane protein 126B | TMEM126B | AF246240 | Hs.476335 | chr11q14.1 |
| 221620_s_at | family with sequence similarity 121B /// NODAL modulator 3 | FAM121B | AF061264 | Hs.40065 | chrxp22.11 |
| 221619_s_at | mitochondrial carrier homolog 1 (C. elegans) | MTCH1 | AF189289 | Hs.279939 | chr6pter-p24.1 |
| 221614_s_at | rabphilin 3A-like (without C2 domains) | RPH3AL | BC005153 | Hs.437436 | chr17p13.3 |
| 221606_s_at | nucleosomal binding protein 1 | NSBP1 | BC005342 | Hs.282204 | chrxq13.3 |
| 221600_s_at | chromosome 11 open reading frame 67 | C11orf67 | BC002752 | Hs.503358 | chr11q13.5 |
| 221597_s_at | HSPC171 protein | HSPC171 | BC003080 | Hs.433203 | chr16q22.1 |
| 221590_s_at | Aldehyde dehydrogenase 6 family, member A1 | ALDH6A1 | AF130089 |  |  |
| 221587_s_at | chromosome 19 open reading frame 24 | C19orf24 | BC000890 | Hs.25489 | chr19p13.3 |
| 221586_s_at | E2F transcription factor 5, p130-binding | E2F5 | U15642 | Hs.447905 | chr8q21.2 |
| 221579_s_at | nudix (nucleoside diphosphate linked moiety X)-type motif 3 | NUDT3 | AF062530 | Hs.4815 | chr6p21.2 |
| 221577_x_at | growth differentiation factor 15 | GDF15 | AF003934 | Hs.296638 | chr19p13.1-13.2 |
| 221571_at | TNF receptor-associated factor 3 | TRAF3 | AI721219 | Hs.297660 | chr14q32.32 |
| 221570_s_at | methyltransferase like 5 | METTL5 | AF201938 | Hs.273063 | chr2q31.1 |
| 221569_at | Abelson helper integration site 1 | AHI1 | AL136797 | Hs.273294 | chr6q23.3 |
| 221566_s_at | nucleolar protein 3 (apoptosis repressor with CARD domain) | NOL3 | AF043244 | Hs.444594 | chr16q21-q23 |
| 221565_s_at | family with sequence similarity 26, member B | FAM26B | BC000039 | Hs.241545 | chr10pter-q26.12 |
| 221561_at | sterol O-acyltransferase (acyl-Coenzyme A: cholesterol acyltransferase) 1 | SOAT1 | L21934 | Hs.446331 | chr1q25 |
| 221559_s_at | MIS12, MIND kinetochore complex component, homolog (yeast) | MIS12 | BC000229 | Hs.267194 | chr17p13.2 |
| 221553_at | implantation-associated protein /// similar to implantation-associated protein | RP11-217H1.1 /// LOC728866 | AL136636 | Hs.323562 | chrxq21.1 |
| 221550_at | COX15 homolog, cytochrome c oxidase assembly protein (yeast) | COX15 | BC002382 | Hs.226581 | chr10q24 |
| 221549_at | glutamate-rich WD repeat containing 1 | GRWD1 | AF337808 | Hs.400625 | chr19q13.32 |
| 221548_s_at | integrin-linked kinase-associated serine/threonine phosphatase 2C | ILKAP | AY024365 | Hs.92033 | chr2q37.3 |
| 221547_at | PRP18 pre-mRNA processing factor 18 homolog (S. cerevisiae) | PRPF18 | BC000794 | Hs.306456 | chr10p13 |
| 221545_x_at | thyroid hormone receptor associated protein 5 | THRAP5 | AF106934 | Hs.365207 | chr19p13.3 |
| 221541_at | cysteine-rich secretory protein LCCL domain containing 2 | CRISPLD2 | AL136861 | Hs.262958 | chr16q24.1 |
| 221539_at | eukaryotic translation initiation factor 4E binding protein 1 | EIF4EBP1 | AB044548 | Hs.406408 | chr8p12 |
| 221537_at | plexin A1 | PLXNA1 | T16388 | Hs.432329 | chr3q21.3 |
| 221535_at | large subunit GTPase 1 homolog (S. cerevisiae) | LSG1 | AL136897 | Hs.436471 | chr3q29 |
| 221534_at | chromosome 11 open reading frame 68 | C11orf68 | AF073483 | Hs.433573 | chr11q13.1 |
| 221530_s_at | basic helix-loop-helix domain containing, class B, 3 | BHLHB3 | BE857425 | Hs.437282 | chr12p11.23-p12.1 |
| 221527_s_at | par-3 partitioning defective 3 homolog (C. elegans) | PARD3 | AF196185 | Hs.72249 | chr10p11.22-p11.21 |
| 221524_s_at | Ras-related GTP binding D | RRAGD | AF272036 | Hs.238679 | chr6q15-q16 |
| 221522_at | ankyrin repeat domain 27 (VPS9 domain) | ANKRD27 | AL136784 | Hs.59236 | chr19q13.11 |
| 221519_at | F-box and WD-40 domain protein 4 | FBXW4 | AF281859 | Hs.301075 | chr10q24 |
| 221515_s_at | leucine carboxyl methyltransferase 1 | LCMT1 | BC001214 | Hs.411135 | chr16p12.3-16p12.1 |
| 221514_at | UTP14, U3 small nucleolar ribonucleoprotein, homolog A (yeast) | UTP14A | BC001149 | Hs.271926 | chrxq25 |
| 221513_s_at | UTP14, U3 small nucleolar ribonucleoprotein, homolog C (yeast) /// UTP14, U3 small nucleolar ribonucleoprotein, homolog A (yeast) | UTP14C | BC001149 | Hs.271926 | chrxq25 /// chr13q14.2 |
| 221508_at | TAO kinase 3 | TAOK3 | AF181985 | Hs.12040 | chr12q |
| 221502_at | karyopherin alpha 3 (importin alpha 4) | KPNA3 | AL120704 | Hs.407765 | chr13q14.3 |
| 221501_x_at | hypothetical protein LOC339047 | LOC339047 | AF229069 | Hs.449809 | chr16p12.3 |
| 221499_s_at | syntaxin 16 | STX16 | AK026970 | Hs.349074 | chr20q13.32 |
| 221496_s_at | transducer of ERBB2, 2 | TOB2 | D64109 | Hs.4994 | chr22q13.2-q13.31 |
| 221493_at | TSPY-like 1 | TSPYL1 | AL136629 | Hs.458358 | chr6q22-q23 |
| 221490_at | ubiquitin associated protein 1 | UBAP1 | AL136733 | Hs.436169 | chr9p22-p21 |
| 221489_s_at | sprouty homolog 4 (Drosophila) | SPRY4 | W48843 | Hs.406507 | chr5q31.3 |
| 221488_s_at | cutA divalent cation tolerance homolog (E. coli) | CUTA | AF230924 | Hs.107187 | chr6pter-p21.31 |
| 221487_s_at | endosulfine alpha | ENSA | AF157510 | Hs.511916 | chr1q21.2 |
| 221478_at | BCL2/adenovirus E1B 19kDa interacting protein 3-like /// BCL2/adenovirus E1B 19kDa interacting protein 3-like | BNIP3L | AL132665 | Hs.132955 | chr8p21 |
| 221477_s_at | hypothetical protein MGC5618 | MGC5618 | BF575213 | Hs.535958 |  |
| 221475_s_at | ribosomal protein L15 | RPL15 | NM_002948 | Hs.381219 | chr3p24.2 |
| 221474_at | myosin regulatory light chain MRLC2 | MRLC2 | U26162 | Hs.511744 | chr18p11.31 |
| 221437_s_at | erythrocyte membrane protein band 4.1 (elliptocytosis 1, RH-linked) /// erythrocyte membrane protein band 4.1 (elliptocytosis 1, RH-linked) /// mitochondrial ribosomal protein S15 /// mitochondrial ribosomal protein S15 | EPB41 | NM_031280 | Hs.352839 | chr1p35-p34.1 |
| 221434_s_at | chromosome 14 open reading frame 156 /// chromosome 14 open reading frame 156 | C14orf156 | NM_031210 | Hs.324521 | chr14q24.3 |
| 221430_s_at | ring finger protein 146 /// ring finger protein 146 | RNF146 | NM_030963 | Hs.267120 | chr6q22.1-q22.33 |
| 221428_s_at | transducin (beta)-like 1X-linked receptor 1 /// transducin (beta)-like 1X-linked receptor 1 | TBL1XR1 | NM_030921 | Hs.438970 | chr3q26.32 |
| 221425_s_at | HESB like domain containing 2 /// HESB like domain containing 2 | HBLD2 | NM_030940 | Hs.449291 | chr9q21.33 |
| 221423_s_at | Yip1 domain family, member 5 /// Yip1 domain family, member 5 | YIPF5 | NM_030799 | Hs.5672 | chr5q31.3 |
| 221335_x_at | hypothetical protein FLJ12886 | FLJ12886 | NM_019108 | Hs.10116 | chr19q13.31 |
| 221323_at | UL16 binding protein 1 | ULBP1 | NM_025218 | Hs.326559 | chr6q25 |
| 221306_at | G protein-coupled receptor 27 | GPR27 | NM_018971 | Hs.356084 | chr3p21-p14 |
| 221290_s_at | melanoma associated antigen (mutated) 1 | MUM1 | NM_016473 | Hs.454758 | chr19p13.3 |
| 221277_s_at | pseudouridylate synthase 3 /// pseudouridylate synthase 3 | PUS3 | NM_031307 | Hs.98682 | chr11q24.2 |
| 221269_s_at | SH3 domain binding glutamic acid-rich protein like 3 /// SH3 domain binding glutamic acid-rich protein like 3 | SH3BGRL3 | NM_031286 | Hs.109051 | chr1p35-p34.3 |
| 221268_s_at | sphingosine-1-phosphate phosphatase 1 /// sphingosine-1-phosphate phosphatase 1 | SGPP1 | NM_030791 | Hs.24678 | chr14q23.2 |
| 221267_s_at | family with sequence similarity 108, member A1 /// family with sequence similarity 108, member A1 | FAM108A1 | NM_031213 | Hs.374437 | chr19p13.3 |
| 221265_s_at | chromosome 15 open reading frame 44 /// chromosome 15 open reading frame 44 | C15orf44 | NM_030800 | Hs.6686 | chr15q22.31 |
| 221264_s_at | TAR DNA binding protein /// TAR DNA binding protein | TARDBP | NM_031214 | Hs.300624 | chr1p36.22 |
| 221263_s_at | splicing factor 3b, subunit 5, 10kDa /// splicing factor 3b, subunit 5, 10kDa | SF3B5 | NM_031287 | Hs.110695 | chr6q24.2 |
| 221258_s_at | kinesin family member 18A /// kinesin family member 18A | KIF18A | NM_031217 | Hs.301052 | chr11p14.1 |
| 221256_s_at | haloacid dehalogenase-like hydrolase domain containing 3 /// haloacid dehalogenase-like hydrolase domain containing 3 | HDHD3 | NM_031219 | Hs.7739 | chr9q32 |
| 221255_s_at | transmembrane protein 93 /// transmembrane protein 93 | TMEM93 | NM_031298 | Hs.30011 | chr17p13.2 |
| 221249_s_at | family with sequence similarity 117, member A /// family with sequence similarity 117, member A | FAM117A | NM_030802 | Hs.9851 | chr17q21.33 |
| 221247_s_at | Williams-Beuren syndrome chromosome region 16 /// Williams-Beuren syndrome chromosome region 16 | WBSCR16 | NM_030798 | Hs.190466 | chr7q11.23 |
| 221245_s_at | frizzled homolog 5 (Drosophila) /// frizzled homolog 5 (Drosophila) | FZD5 | NM_030804 | Hs.17631 | chr2q34 |
| 221235_s_at | Hypothetical LOC644617 /// Hypothetical LOC644617 | LOC644617 | NM_030825 |  |  |
| 221229_s_at | hypothetical protein FLJ20628 /// hypothetical protein FLJ20628 | FLJ20628 | NM_017910 | Hs.32356 | chr2p23.2 |
| 221216_s_at | sex comb on midleg homolog 1 (Drosophila) | SCMH1 | NM_012236 | Hs.523167 | chr1p34 |
| 221214_s_at | nasal embryonic LHRH factor | NELF | NM_015537 | Hs.455285 | chr9q34.3 |
| 221213_s_at | suppressor of hairy wing homolog 4 (Drosophila) | SUHW4 | NM_017661 | Hs.46821 | chr15q21.3 |
| 221208_s_at | chromosome 11 open reading frame 61 | C11orf61 | NM_024631 | Hs.38592 | chr11q24.2 |
| 221206_at | PMS2 postmeiotic segregation increased 2 (S. cerevisiae) | PMS2 | NM_024521 | Hs.177548 | chr7p22.2 |
| 221194_s_at | PTD016 protein | LOC51136 | NM_016125 | Hs.30154 | chr17q23.2 |
| 221192_x_at | hypothetical protein ET | ET | NM_024311 | Hs.296924 | chr17q25 |
| 221185_s_at | IQ motif containing G | IQCG | NM_025111 | Hs.512778 | chr3q29 |
| 221175_at | chromosome 3 open reading frame 36 | C3orf36 | NM_025041 | Hs.287691 | chr3q22.1 |
| 221158_at | chromosome 21 open reading frame 66 | C21orf66 | NM_013329 | Hs.297559 | chr21q21.3 |
| 221139_s_at | cysteine sulfinic acid decarboxylase | CSAD | NM_015989 | Hs.279815 | chr12q13.11-q14.3 |
| 221135_s_at | asteroid homolog 1 (Drosophila) | ASTE1 | NM_014065 | Hs.254124 | chr3q21.3 |
| 221127_s_at | regulated in glioma | RIG | NM_006394 |  | chr11p15.1 |
| 221107_at | cholinergic receptor, nicotinic, alpha 9 | CHRNA9 | NM_017581 | Hs.272278 | chr4p14 |
| 221094_s_at | elongation protein 3 homolog (S. cerevisiae) | ELP3 | NM_018091 | Hs.267905 | chr8p21.1 |
| 221090_s_at | 2-oxoglutarate and iron-dependent oxygenase domain containing 1 | OGFOD1 | NM_018233 | Hs.231883 | chr16q12.2 |
| 221088_s_at | protein phosphatase 1, regulatory (inhibitor) subunit 9A | PPP1R9A | NM_017650 | Hs.489160 | chr7q21.3 |
| 221079_s_at | methyltransferase like 2B /// methyltransferase like 2A | METTL2B | NM_018396 | Hs.433213 | chr7q32.2 /// chr17q23.3 |
| 221069_s_at | coiled-coil domain containing 44 | CCDC44 | NM_016360 | Hs.174134 | chr17q23.3 |
| 221063_x_at | ring finger protein 123 | RNF123 | NM_022064 | Hs.512684 | chr3p24.3 |
| 221059_s_at | coactosin-like 1 (Dictyostelium) | COTL1 | NM_021615 | Hs.289092 | chr16q24.1 |
| 221046_s_at | GTP-binding protein 8 (putative) | GTPBP8 | NM_014170 | Hs.127496 | chr3q13.2 |
| 221044_s_at | tripartite motif-containing 34 /// tripartite motif-containing 6 and tripartite motif-containing 34 | TRIM34 | NM_021616 | Hs.125300 | chr11p15 |
| 221036_s_at | anterior pharynx defective 1 homolog B (C. elegans) /// anterior pharynx defective 1 homolog B (C. elegans) | APH1B | NM_031301 | Hs.42954 | chr15q22.2 |
| 221025_x_at | pseudouridylate synthase 7 homolog (S. cerevisiae)-like /// pseudouridylate synthase 7 homolog (S. cerevisiae)-like | PUS7L | NM_031292 | Hs.151093 | chr12q12 |
| 221024_s_at | solute carrier family 2 (facilitated glucose transporter), member 10 /// solute carrier family 2 (facilitated glucose transporter), member 10 | SLC2A10 | NM_030777 | Hs.305971 | chr20q13.1 |
| 221021_s_at | catenin, beta like 1 /// catenin, beta like 1 | CTNNBL1 | NM_030877 | Hs.178576 | chr20q11.23-q12 |
| 221020_s_at | solute carrier family 25, member 32 /// solute carrier family 25, member 32 | SLC25A32 | NM_030780 | Hs.196270 | chr8q22.3 |
| 221016_s_at | transcription factor 7-like 1 (T-cell specific, HMG-box) /// transcription factor 7-like 1 (T-cell specific, HMG-box) | TCF7L1 | NM_031283 | Hs.318517 | chr2p11.2 |
| 221014_s_at | RAB33B, member RAS oncogene family /// RAB33B, member RAS oncogene family | RAB33B | NM_031296 | Hs.534582 | chr4q28 |
| 221012_s_at | tripartite motif-containing 8 /// tripartite motif-containing 8 | TRIM8 | NM_030912 | Hs.54580 | chr10q24.3 |
| 221011_s_at | limb bud and heart development homolog (mouse) /// limb bud and heart development homolog (mouse) | LBH | NM_030915 | Hs.57209 | chr2p23.1 |
| 221007_s_at | FIP1 like 1 (S. cerevisiae) /// FIP1 like 1 (S. cerevisiae) | FIP1L1 | NM_030917 | Hs.66295 | chr4q12 |
| 221006_s_at | sorting nexin family member 27 /// sorting nexin family member 27 | SNX27 | NM_030918 | Hs.67619 | chr1q21.3 |
| 221004_s_at | integral membrane protein 2C /// integral membrane protein 2C | ITM2C | NM_030926 | Hs.111577 | chr2q37 |
| 221002_s_at | tetraspanin 14 /// tetraspanin 14 | TSPAN14 | NM_030927 | Hs.509050 | chr10q22.3 |
| 220987_s_at | chromosome 11 open reading frame 17 /// chromosome 11 open reading frame 17 /// NUAK family, SNF1-like kinase, 2 /// NUAK family, SNF1-like kinase, 2 | C11orf17 | NM_030952 | Hs.131180 | chr11p15.3 /// chr1q32.1 |
| 220985_s_at | ring finger protein 170 /// ring finger protein 170 | RNF170 | NM_030954 | Hs.170822 | chr8p11.21 |
| 220980_s_at | ADP-dependent glucokinase /// ADP-dependent glucokinase | ADPGK | NM_031284 | Hs.10748 | chr15q24.1 |
| 220966_x_at | actin related protein 2/3 complex, subunit 5-like /// actin related protein 2/3 complex, subunit 5-like | ARPC5L | NM_030978 | Hs.132499 | chr9q33.3 |
| 220964_s_at | RAB1B, member RAS oncogene family /// RAB1B, member RAS oncogene family | RAB1B | NM_030981 | Hs.300816 | chr11q12 |
| 220956_s_at | egl nine homolog 2 (C. elegans) | EGLN2 | NM_017555 | Hs.324277 | chr19q13.2 |
| 220955_x_at | RAB23, member RAS oncogene family | RAB23 | NM_016277 | Hs.94769 | chr6p11 |
| 220949_s_at | chromosome 7 open reading frame 49 | C7orf49 | NM_024033 | Hs.443969 | chr7q33 |
| 220948_s_at | ATPase, Na+/K+ transporting, alpha 1 polypeptide | ATP1A1 | NM_000701 | Hs.371889 | chr1p21 |
| 220947_s_at | TBC1 domain family, member 10B | TBC1D10B | NM_015527 | Hs.136581 | chr16p11.2 |
| 220946_s_at | SET domain containing 2 | SETD2 | NM_014159 | Hs.6947 | chr3p21.31 |
| 220945_x_at | MANSC domain containing 1 | MANSC1 | NM_018050 | Hs.5999 | chr12p13.2 |
| 220943_s_at | hypothetical protein PRO1853 | PRO1853 | NM_018607 | Hs.433466 | chr2p22.2 |
| 220942_x_at | chromosome 3 open reading frame 28 | C3orf28 | NM_014367 | Hs.5243 | chr3q21.1 |
| 220941_s_at | chromosome 21 open reading frame 91 | C21orf91 | NM_017447 | Hs.293811 | chr21q21.1 |
| 220937_s_at | ST6 (alpha-N-acetyl-neuraminyl-2,3-beta-galactosyl-1,3)-N-acetylgalactosaminide alpha-2,6-sialyltransferase 4 | ST6GALNAC4 | NM_014403 | Hs.3972 | chr9q34 |
| 220936_s_at | H2A histone family, member J | H2AFJ | NM_018267 | Hs.36727 | chr12p12 |
| 220935_s_at | CDK5 regulatory subunit associated protein 2 | CDK5RAP2 | NM_018249 | Hs.437967 | chr9q33.2 |
| 220934_s_at | hypothetical protein MGC3196 | MGC3196 | NM_024084 | Hs.530463 | chr11q12.3 |
| 220917_s_at | WD repeat domain 19 | WDR19 | NM_025132 | Hs.438482 | chr4p14 |
| 220892_s_at | phosphoserine aminotransferase 1 | PSAT1 | NM_021154 | Hs.286049 | chr9q21.2 |
| 220890_s_at | DEAD (Asp-Glu-Ala-Asp) box polypeptide 47 | DDX47 | NM_016355 | Hs.284288 | chr12p13.2 |
| 220865_s_at | prenyl (decaprenyl) diphosphate synthase, subunit 1 | PDSS1 | NM_014317 | Hs.534447 | chr10p12.1 |
| 220864_s_at | NADH dehydrogenase (ubiquinone) 1 alpha subcomplex, 13 | NDUFA13 | NM_015965 | Hs.279574 | chr19p13.2 |
| 220788_s_at | ring finger protein 31 | RNF31 | NM_017999 | Hs.375217 | chr14q11.2 |
| 220770_s_at | transposon-derived Buster3 transposase-like | LOC63920 | NM_022090 |  | chr5q33.3 |
| 220768_s_at | casein kinase 1, gamma 3 | CSNK1G3 | NM_004384 | Hs.129206 | chr5q23 |
| 220765_s_at | LIM and senescent cell antigen-like domains 2 | LIMS2 | NM_017980 | Hs.127273 | chr2q14.3 |
| 220764_at | protein phosphatase 4, regulatory subunit 2 | PPP4R2 | NM_019853 | Hs.446590 | chr3p13 |
| 220760_x_at | zinc finger protein 665 | ZNF665 | NM_024733 | Hs.117270 | chr19q13.42 |
| 220757_s_at | UBX domain containing 1 | UBXD1 | NM_025241 | Hs.435255 | chr19p13 |
| 220755_s_at | chromosome 6 open reading frame 48 | C6orf48 | NM_016947 | Hs.109798 | chr6p21.3 |
| 220750_s_at | leucine proline-enriched proteoglycan (leprecan) 1 | LEPRE1 | NM_022356 | Hs.437656 | chr1p34.1 |
| 220748_s_at | zinc finger protein 580 | ZNF580 | NM_016202 | Hs.94392 | chr19q13.42 |
| 220746_s_at | ubiquitin interaction motif containing 1 | UIMC1 | NM_016290 | Hs.300263 | chr5q35.2 |
| 220742_s_at | N-glycanase 1 | NGLY1 | NM_018297 | Hs.63657 | chr3p24.2 |
| 220739_s_at | cyclin M3 | CNNM3 | NM_017623 | Hs.414042 | chr2p12-p11.2 |
| 220731_s_at | NECAP endocytosis associated 2 | NECAP2 | NM_018090 | Hs.437385 | chr1p36.13 |
| 220725_x_at | Dynein, axonemal, heavy chain 3 | DNAH3 | NM_025095 | Hs.375739 | chr16p12.3 |
| 220720_x_at | family with sequence similarity 128, member B | FAM128B | NM_025029 | Hs.355815 | chr2q21.1 |
| 220688_s_at | mRNA turnover 4 homolog (S. cerevisiae) | MRT4 | NM_016183 | Hs.463797 | chr1p36.13 |
| 220672_at | KIAA1622 | KIAA1622 | NM_020958 | Hs.259599 | chr14q32.13 |
| 220671_at | CCR4 carbon catabolite repression 4-like (S. cerevisiae) | CCRN4L | NM_012118 | Hs.480763 | chr4q31.1 |
| 220661_s_at | zinc finger protein 692 | ZNF692 | NM_017865 | Hs.377705 | chr1q44 |
| 220647_s_at | coiled-coil-helix-coiled-coil-helix domain containing 8 | CHCHD8 | NM_016565 | Hs.31500 | chr11q13.4 |
| 220643_s_at | Fas apoptotic inhibitory molecule | FAIM | NM_018147 | Hs.173438 | chr3q22.3 |
| 220609_at | hypothetical protein LOC202181 | LOC202181 | NM_024651 |  |  |
| 220607_x_at | TH1-like (Drosophila) | TH1L | NM_016397 | Hs.5184 | chr20q13 |
| 220605_s_at | sirtuin (silent mating type information regulation 2 homolog) 2 (S. cerevisiae) | SIRT2 | NM_012237 | Hs.375214 | chr19q13 |
| 220595_at | PDZ domain containing RING finger 4 | PDZRN4 | NM_013377 | Hs.380044 | chr12q12 |
| 220587_s_at | G protein beta subunit-like | GBL | NM_022372 | Hs.29203 | chr16p13.3 |
| 220580_at | bicaudal C homolog 1 (Drosophila) | BICC1 | NM_025044 | Hs.287696 | chr10q21.1 |
| 220547_s_at | family with sequence similarity 35, member A | FAM35A | NM_019054 | Hs.458301 | chr10q23.2 |
| 220532_s_at | transmembrane protein 176B | TMEM176B | NM_014020 | Hs.190161 | chr7q36.1 |
| 220526_s_at | mitochondrial ribosomal protein L20 /// similar to mitochondrial ribosomal protein L20 | MRPL20 | NM_017971 | Hs.182698 | chr1p36.3-p36.2 |
| 220525_s_at | ancient ubiquitous protein 1 | AUP1 | NM_012103 | Hs.411480 | chr2p13 |
| 220500_s_at | RAB, member of RAS oncogene family-like 2B /// RAB, member of RAS oncogene family-like 2A | RABL2B | NM_007082 | Hs.446425 | chr2q13 /// chr22q13.33 |
| 220495_s_at | chromosome 5 open reading frame 14 | C5orf14 | NM_024715 | Hs.106534 | chr5q31.1 |
| 220477_s_at | chromosome 20 open reading frame 30 | C20orf30 | NM_014145 | Hs.380990 | chr20p12.3 |
| 220475_at | solute carrier family 28 (sodium-coupled nucleoside transporter), member 3 | SLC28A3 | NM_022127 | Hs.534556 | chr9q22.2 |
| 220462_at | TGF-beta induced apoptosis protein 2 | TAIP-2 | NM_024969 | Hs.287442 | chr2q24.3 |
| 220459_at | MCM3 minichromosome maintenance deficient 3 (S. cerevisiae) associated protein antisense | MCM3APAS | NM_018118 | Hs.517368 | chr21q22.3 |
| 220452_x_at | Cat eye syndrome chromosome region, candidate 7 | CECR7 | NM_021031 |  |  |
| 220408_x_at | family with sequence similarity 48, member A | FAM48A | NM_017569 | Hs.435815 | chr13q13.3 |
| 220391_at | zinc finger and BTB domain containing 3 | ZBTB3 | NM_024784 | Hs.147554 | chr11q12.3 |
| 220387_s_at | HERV-H LTR-associating 3 | HHLA3 | NM_007071 | Hs.142245 | chr1p31.1 |
| 220386_s_at | echinoderm microtubule associated protein like 4 | EML4 | NM_019063 | Hs.386198 | chr2p22-p21 |
| 220371_s_at | solute carrier family 12 (potassium/chloride transporters), member 9 | SLC12A9 | NM_020246 | Hs.521087 | chr7q22 |
| 220370_s_at | ubiquitin specific peptidase 36 | USP36 | NM_025090 | Hs.11387 | chr17q25.3 |
| 220368_s_at | SMEK homolog 1, suppressor of mek1 (Dictyostelium) | SMEK1 | NM_017936 | Hs.334657 | chr14q32.12 |
| 220367_s_at | Sin3A-associated protein, 130kDa | SAP130 | NM_024545 | Hs.133523 | chr2q14.3 |
| 220346_at | methylenetetrahydrofolate dehydrogenase (NADP+ dependent) 2-like | MTHFD2L | NM_025001 | Hs.535153 | chr4q13.3 |
| 220342_x_at | ER degradation enhancer, mannosidase alpha-like 3 | EDEM3 | NM_017992 | Hs.308809 | chr1q24-q25 |
| 220330_s_at | SAM domain, SH3 domain and nuclear localization signals 1 | SAMSN1 | NM_022136 | Hs.221851 | chr21q11 |
| 220317_at | lecithin retinol acyltransferase (phosphatidylcholine--retinol O-acyltransferase) | LRAT | NM_004744 | Hs.481028 | chr4q32.1 |
| 220311_at | N-6 adenine-specific DNA methyltransferase 1 (putative) | N6AMT1 | NM_013240 | Hs.163846 | chr21q21.3 |
| 220305_at | hypothetical protein MGC3260 | MGC3260 | NM_024030 | Hs.134883 | chr20p13 |
| 220295_x_at | DEP domain containing 1 /// similar to DEP domain containing 1 | DEPDC1 | NM_017779 | Hs.445098 | chr1p31.2 |
| 220261_s_at | zinc finger, DHHC-type containing 4 | ZDHHC4 | NM_018106 | Hs.5268 | chr7p22.1 |
| 220254_at | low density lipoprotein-related protein 12 | LRP12 | NM_013437 | Hs.301974 | chr8q22.2-q23.1 |
| 220251_at | chromosome 1 open reading frame 107 | C1orf107 | NM_024998 | Hs.194754 | chr1q32.2 |
| 220244_at | loss of heterozygosity, 3, chromosomal region 2, gene A | LOH3CR2A | NM_013343 | Hs.433151 | chr3p24-26 |
| 220242_x_at | zinc finger protein 701 | ZNF701 | NM_018260 | Hs.412951 | chr19q13.41 |
| 220241_at | transmembrane and coiled-coil domains 3 | TMCO3 | NM_017905 | Hs.27337 | chr13q34 |
| 220238_s_at | kelch-like 7 (Drosophila) | KLHL7 | NM_018846 | Hs.385861 | chr7p15.3 |
| 220237_at | ATG3 autophagy related 3 homolog (S. cerevisiae) | ATG3 | NM_022488 | Hs.26367 | chr3q13.2 |
| 220235_s_at | chromosome 1 open reading frame 103 | C1orf103 | NM_018372 | Hs.25245 | chr1p13.3 |
| 220234_at | carbonic anhydrase VIII | CA8 | NM_004056 | Hs.131583 | chr8q11-q12 |
| 220219_s_at | leucine rich repeat containing 37A /// hypothetical protein FLJ10120 /// leucine rich repeat containing 37, member A3 /// leucine rich repeat containing 37, member A2 /// hypothetical protein LOC728863 | LRRC37A | NM_018001 | Hs.500495 | chr17q24.2 /// chr17 /// chr17q21.31 |
| 220215_at | zinc finger protein 669 | ZNF669 | NM_024804 | Hs.163754 | chr1q44 |
| 220212_s_at | thyroid adenoma associated | THADA | NM_022065 | Hs.16063 | chr2p21 |
| 220206_at | zinc finger, MYM-type 1 | ZMYM1 | NM_024772 | Hs.348006 | chr1p34.3 |
| 220199_s_at | chromosome 1 open reading frame 80 | C1orf80 | NM_022831 | Hs.428779 | chr1q41 |
| 220195_at | methyl-CpG binding domain protein 5 | MBD5 | NM_018328 | Hs.458312 | chr2q23.1 |
| 220189_s_at | mannosyl (alpha-1,3-)-glycoprotein beta-1,4-N-acetylglucosaminyltransferase, isozyme B | MGAT4B | NM_014275 | Hs.437277 | chr5q35 |
| 220178_at | chromosome 19 open reading frame 28 | C19orf28 | NM_021731 | Hs.245372 | chr19p13.3 |
| 220176_at | nucleotide binding protein-like | NUBPL | NM_025152 | Hs.288981 | chr14q12 |
| 220175_s_at | COBW domain containing 1 /// COBW domain containing 2 /// COBW domain containing 5 /// COBW-like placental protein /// COBW domain containing 3 /// similar to COBW domain containing 3 /// similar to COBW domain containing 3 | CBWD1 | NM_020667 | Hs.355950 | chr9q21.13 /// chr9p24.3 /// chr2q14.1 /// chr9q13 |
| 220174_at | leucine rich repeat containing 8 family, member E | LRRC8E | NM_025061 | Hs.501511 | chr19p13.2 |
| 220173_at | chromosome 14 open reading frame 45 | C14orf45 | NM_025057 | Hs.260555 | chr14q24.3 |
| 220167_s_at | TP53TG3 protein /// similar to TP53TG3 protein /// similar to TP53TG3 protein | TP53TG3 | NM_015369 | Hs.499111 | chr16p13 |
| 220159_at | ATP-binding cassette, sub-family A (ABC1), member 11 (pseudogene) | ABCA11 | NM_024903 | Hs.478916 | chr4p16.3 |
| 220157_x_at | pleckstrin homology domain containing, family A (phosphoinositide binding specific) member 9 /// pleckstrin homology domain containing, family A (phosphoinositide binding specific) member 8 | PLEKHA9 | NM_015899 | Hs.334649 | chr12q |
| 220155_s_at | bromodomain containing 9 | BRD9 | NM_023924 | Hs.449278 | chr5p15.33 |
| 220150_s_at | chromosome 6 open reading frame 60 | C6orf60 | NM_024581 | Hs.20537 | chr6q22.31 |
| 220148_at | aldehyde dehydrogenase 8 family, member A1 | ALDH8A1 | NM_022568 | Hs.18443 | chr6q23.2 |
| 220147_s_at | family with sequence similarity 60, member A | FAM60A | NM_021238 | Hs.356223 | chr12p11 |
| 220143_x_at | LUC7-like (S. cerevisiae) | LUC7L | NM_018032 | Hs.16803 | chr16p13.3 |
| 220140_s_at | sorting nexin 11 | SNX11 | NM_013323 | Hs.15827 | chr17q21.32 |
| 220127_s_at | F-box and leucine-rich repeat protein 12 | FBXL12 | NM_017703 | Hs.12439 | chr19p13.2 |
| 220104_at | zinc finger CCCH-type, antiviral 1 | ZC3HAV1 | NM_020119 | Hs.35254 | chr7q34 |
| 220102_at | forkhead box L2 | FOXL2 | NM_023067 | Hs.289292 | chr3q23 |
| 220091_at | solute carrier family 2 (facilitated glucose transporter), member 6 | SLC2A6 | NM_017585 | Hs.244378 | chr9q34 |
| 220083_x_at | ubiquitin carboxyl-terminal hydrolase L5 | UCHL5 | NM_016017 | Hs.25223 | chr1q32 |
| 220081_x_at | hydroxysteroid (17-beta) dehydrogenase 7 /// hydroxysteroid (17-beta) dehydrogenase 7 pseudogene 2 /// similar to hydroxysteroid (17-beta) dehydrogenase 7 | HSD17B7 | NM_016371 | Hs.187579 | chr1q23 |
| 220078_at | ubiquitin specific peptidase 48 | USP48 | NM_018391 | Hs.334477 | chr1p36.12 |
| 220072_at | centrosome and spindle pole associated protein 1 | CSPP1 | NM_024790 | Hs.370147 | chr8q13.2 |
| 220052_s_at | TERF1 (TRF1)-interacting nuclear factor 2 | TINF2 | NM_012461 | Hs.7797 | chr14q11.2 |
| 220050_at | chromosome 9 open reading frame 9 | C9orf9 | NM_018956 | Hs.62595 | chr9q34 |
| 220046_s_at | cyclin L1 | CCNL1 | NM_020307 | Hs.4859 | chr3q25.31 |
| 220038_at | serum/glucocorticoid regulated kinase family, member 3 | SGK3 | NM_013257 | Hs.380877 | chr8q12.3-8q13.1 |
| 220036_s_at | syntaxin 6 /// limb region 1 homolog (mouse)-like | STX6 | NM_018113 | Hs.272838 | chr12q13.12 |
| 220034_at | interleukin-1 receptor-associated kinase 3 | IRAK3 | NM_007199 | Hs.268552 | chr12q14.3 |
| 220030_at | serine/threonine/tyrosine kinase 1 | STYK1 | NM_018423 | Hs.24979 | chr12p13.2 |
| 220027_s_at | Ras interacting protein 1 | RASIP1 | NM_017805 | Hs.233955 | chr19q13.33 |
| 220018_at | Cas-Br-M (murine) ecotropic retroviral transforming sequence-like 1 | CBLL1 | NM_024814 | Hs.458382 | chr7q31.1 |
| 220015_at | castor homolog 1, zinc finger (Drosophila) | CASZ1 | NM_017766 | Hs.534520 | chr1p36.22 |
| 219998_at | galectin-related protein | HSPC159 | NM_014181 | Hs.372208 | chr2p14 |
| 219996_at | ankyrin repeat and SOCS box-containing 7 | ASB7 | NM_024708 | Hs.357077 | chr15q26.3 |
| 219988_s_at | chromosome 1 open reading frame 164 | C1orf164 | NM_018150 | Hs.90375 | chr1p34.1 |
| 219983_at | HRAS-like suppressor | HRASLS | NM_020386 | Hs.36761 | chr3q29 |
| 219982_s_at | small EDRK-rich factor 1A (telomeric) /// small EDRK-rich factor 1B (centromeric) /// similar to small EDRK-rich factor 1A, telomeric | SERF1A | NM_022978 | Hs.482391 | chr5q12.2-q13.3 |
| 219972_s_at | chromosome 14 open reading frame 135 | C14orf135 | NM_022495 | Hs.413671 | chr14q23.1 |
| 219962_at | angiotensin I converting enzyme (peptidyl-dipeptidase A) 2 | ACE2 | NM_021804 | Hs.178098 | chrxp22 |
| 219940_s_at | PCI domain containing 2 | PCID2 | NM_018386 | Hs.7049 | chr13q34 |
| 219938_s_at | proline-serine-threonine phosphatase interacting protein 2 | PSTPIP2 | NM_024430 | Hs.413501 | chr18q12 |
| 219933_at | glutaredoxin 2 | GLRX2 | NM_016066 | Hs.458283 | chr1q31.2-q31.3 |
| 219932_at | solute carrier family 27 (fatty acid transporter), member 6 | SLC27A6 | NM_014031 | Hs.49765 | chr5q23.3 |
| 219931_s_at | kelch-like 12 (Drosophila) | KLHL12 | NM_021633 | Hs.3826 | chr1q32.1 |
| 219929_s_at | zinc finger, FYVE domain containing 21 | ZFYVE21 | NM_024071 | Hs.169575 | chr14q32.33 |
| 219927_at | chromosome 14 open reading frame 111 | C14orf111 | NM_015962 | Hs.343173 | chr14q24.3 |
| 219926_at | popeye domain containing 3 | POPDC3 | NM_022361 | Hs.458336 | chr6q21 |
| 219924_s_at | zinc finger, MYM-type 6 | ZMYM6 | NM_007167 | Hs.524353 | chr1p34.2 |
| 219922_s_at | latent transforming growth factor beta binding protein 3 | LTBP3 | NM_021070 | Hs.289019 | chr11q12 |
| 219921_s_at | dedicator of cytokinesis 5 | DOCK5 | NM_024940 | Hs.293748 | chr8p21.2 |
| 219920_s_at | GDP-mannose pyrophosphorylase B | GMPPB | NM_021971 | Hs.438691 | chr3p21.31 |
| 219913_s_at | Crn, crooked neck-like 1 (Drosophila) | CRNKL1 | NM_016652 | Hs.171342 | chr20p11.2 |
| 219910_at | Huntingtin interacting protein E | HYPE | NM_007076 | Hs.486371 | chr12q24.1 |
| 219905_at | erythroblast membrane-associated protein (Scianna blood group) | ERMAP | NM_018538 | Hs.427672 | chr1p34.2 |
| 219894_at | MAGE-like 2 | MAGEL2 | NM_019066 | Hs.141496 | chr15q11-q12 |
| 219889_at | frequently rearranged in advanced T-cell lymphomas | FRAT1 | NM_005479 | Hs.126057 | chr10q24.1 |
| 219888_at | sperm associated antigen 4 | SPAG4 | NM_003116 | Hs.123159 | chr20q11.21 |
| 219878_s_at | Kruppel-like factor 13 | KLF13 | NM_015995 | Hs.7104 | chr15q12 |
| 219872_at | chromosome 4 open reading frame 18 | C4orf18 | NM_016613 | Hs.323583 | chr4q32.1 |
| 219870_at | activating transcription factor 7 interacting protein 2 | ATF7IP2 | NM_024997 | Hs.145431 | chr16p13.13 |
| 219863_at | hect domain and RLD 5 | HERC5 | NM_016323 | Hs.26663 | chr4q22.1-q23 |
| 219862_s_at | nuclear prelamin A recognition factor | NARF | NM_012336 | Hs.256526 | chr17q25.3 |
| 219861_at | DnaJ (Hsp40) homolog, subfamily C, member 17 | DNAJC17 | NM_018163 | Hs.334475 | chr15q15.1 |
| 219858_s_at | FLJ20160 protein | FLJ20160 | NM_017694 | Hs.418581 | chr2q32.2 |
| 219855_at | nudix (nucleoside diphosphate linked moiety X)-type motif 11 | NUDT11 | NM_018159 | Hs.200016 | chrxp11.22 |
| 219854_at | zinc finger protein 14 | ZNF14 | NM_021030 | Hs.466261 | chr19p13.3-p13.2 |
| 219849_at | zinc finger protein 671 | ZNF671 | NM_024833 | Hs.180402 | chr19q13.43 |
| 219848_s_at | zinc finger protein 432 | ZNF432 | NM_014650 |  |  |
| 219843_at | intracisternal A particle-promoted polypeptide | IPP | NM_005897 | Hs.157180 | chr1p34-p32 |
| 219831_at | cyclin-dependent kinase-like 3 | CDKL3 | NM_016508 | Hs.105818 | chr5q31 |
| 219818_s_at | G patch domain containing 1 | GPATCH1 | NM_018025 | Hs.55014 | chr19q13.11 |
| 219816_s_at | RNA binding motif protein 23 | RBM23 | NM_018107 | Hs.4997 | chr14q11.2 |
| 219814_at | muscleblind-like 3 (Drosophila) | MBNL3 | NM_018388 | Hs.105134 | chrxq26.2 |
| 219809_at | WD repeat domain 55 | WDR55 | NM_017706 | Hs.286261 | chr5q31.3 |
| 219806_s_at | chromosome 11 open reading frame 75 | C11orf75 | NM_020179 | Hs.416456 | chr11q13.3-q23.3 |
| 219805_at | chromosome X open reading frame 56 /// similar to CG16865-PA /// similar to CG16865-PA | CXorf56 | NM_022101 | Hs.248572 | chrxq23 |
| 219802_at | hypothetical protein FLJ22028 | FLJ22028 | NM_024854 | Hs.533761 | chr12p12.1 |
| 219801_at | zinc finger protein 34 | ZNF34 | NM_030580 | Hs.17364 | chr8q24.3 |
| 219799_s_at | dehydrogenase/reductase (SDR family) member 9 | DHRS9 | NM_005771 | Hs.179608 | chr2q31.1 |
| 219798_s_at | bin3, bicoid-interacting 3, homolog (Drosophila) | BCDIN3 | NM_019606 | Hs.178011 | chr7q22.1 |
| 219797_at | mannosyl (alpha-1,3-)-glycoprotein beta-1,4-N-acetylglucosaminyltransferase, isozyme A | MGAT4A | NM_012214 | Hs.177576 | chr2q12 |
| 219793_at | sorting nexin 16 | SNX16 | NM_022133 | Hs.310194 | chr8q21.13 |
| 219790_s_at | natriuretic peptide receptor C/guanylate cyclase C (atrionatriuretic peptide receptor C) | NPR3 | NM_000908 | Hs.237028 | chr5p14-p13 |
| 219787_s_at | epithelial cell transforming sequence 2 oncogene | ECT2 | NM_018098 | Hs.293257 | chr3q26.1-q26.2 |
| 219785_s_at | F-box protein 31 | FBXO31 | NM_024735 | Hs.126145 | chr16q24.2 |
| 219778_at | zinc finger protein, multitype 2 | ZFPM2 | NM_012082 | Hs.106309 | chr8q23 |
| 219765_at | zinc finger protein 329 | ZNF329 | NM_024620 | Hs.458377 | chr19q13.43 |
| 219762_s_at | ribosomal protein L36 | RPL36 | NM_015414 | Hs.408018 | chr19p13.3 |
| 219754_at | RNA binding motif protein 41 | RBM41 | NM_018301 | Hs.139053 | chrxq22.1-24 |
| 219751_at | SET domain containing 6 | SETD6 | NM_024860 | Hs.534570 | chr16q21 |
| 219749_at | SH2 domain containing 4A | SH2D4A | NM_022071 | Hs.17433 | chr8p21.2 |
| 219743_at | hairy/enhancer-of-split related with YRPW motif 2 | HEY2 | NM_012259 | Hs.144287 | chr6q22.2-q22.33 |
| 219741_x_at | zinc finger protein 552 | ZNF552 | NM_024762 | Hs.129691 | chr19q13.43 |
| 219731_at | weakly similar to zinc finger protein 195 | FLJ34077 | NM_024343 | Hs.534994 | chr10q23-q24 |
| 219727_at | dual oxidase 2 | DUOX2 | NM_014080 | Hs.71377 | chr15q15.3 |
| 219717_at | chromosome 4 open reading frame 30 | C4orf30 | NM_017741 | Hs.163839 | chr4p15.32 |
| 219709_x_at | chromosome 16 open reading frame 24 | C16orf24 | NM_023933 | Hs.381943 | chr16p13.3 |
| 219706_at | chromosome 20 open reading frame 29 | C20orf29 | NM_018347 | Hs.146023 | chr20p13 |
| 219703_at | meiosis-specific nuclear structural 1 | MNS1 | NM_018365 | Hs.303573 | chr15q21.3 |
| 219696_at | hypothetical protein FLJ20054 | FLJ20054 | NM_019049 | Hs.369838 | chr1q31.3 |
| 219690_at | transmembrane protein 149 | TMEM149 | NM_024660 | Hs.352548 | chr19q13.12 |
| 219688_at | Bardet-Biedl syndrome 7 | BBS7 | NM_018190 | Hs.58974 | chr4q27 |
| 219683_at | frizzled homolog 3 (Drosophila) | FZD3 | NM_017412 | Hs.40735 | chr8p21 |
| 219677_at | splA/ryanodine receptor domain and SOCS box containing 1 | SPSB1 | NM_025106 | Hs.458389 | chr1p36.22 |
| 219676_at | zinc finger protein 435 | ZNF435 | NM_025231 | Hs.288539 | chr6p22.1 |
| 219675_s_at | UDP-glucuronate decarboxylase 1 | UXS1 | NM_025076 | Hs.288158 | chr2q12.2 |
| 219673_at | chromosome 6 open reading frame 61 | C6orf61 | NM_017696 | Hs.279008 | chr6q22.31 |
| 219671_at | hippocalcin like 4 | HPCAL4 | AL136591 | Hs.272891 | chr1p34.2 |
| 219665_at | nudix (nucleoside diphosphate linked moiety X)-type motif 18 | NUDT18 | NM_024815 | Hs.441321 | chr8p21.3 |
| 219662_at | chromosome 2 open reading frame 49 | C2orf49 | NM_024093 | Hs.303899 | chr2q12.1 |
| 219649_at | asparagine-linked glycosylation 6 homolog (S. cerevisiae, alpha-1,3-glucosyltransferase) | ALG6 | NM_013339 | Hs.80042 | chr1p31.3 |
| 219648_at | melanoregulin | MREG | NM_018000 | Hs.79741 | chr2q35 |
| 219647_at | popeye domain containing 2 | POPDC2 | NM_022135 | Hs.512688 | chr3q13.33 |
| 219646_at | hypothetical protein FLJ20186 | FLJ20186 | NM_017702 | Hs.62771 | chr16q24.3 |
| 219644_at | coiled-coil domain containing 41 | CCDC41 | NM_016122 | Hs.279209 | chr12q22 |
| 219641_at | de-etiolated homolog 1 (Arabidopsis) | DET1 | NM_017996 | Hs.42140 | chr15q25.3 |
| 219635_at | zinc finger protein 606 | ZNF606 | NM_025027 | Hs.287629 | chr19q13.4 |
| 219633_at | ADP-ribosyltransferase 4 (Dombrock blood group) /// chromosome 20 open reading frame 121 | ART4 | NM_024331 | Hs.283869 | chr20q13.12 |
| 219628_at | zinc finger, matrin type 3 | ZMAT3 | NM_022470 | Hs.386299 | chr3q26.3-q27 |
| 219626_at | hypothetical protein FLJ12649 | RP11-535K18.3 | NM_024597 | Hs.446275 | chrxq26.3 |
| 219623_at | ARP5 actin-related protein 5 homolog (yeast) | ACTR5 | NM_024855 | Hs.371585 | chr20q11.23 |
| 219622_at | RAB20, member RAS oncogene family | RAB20 | NM_017817 | Hs.179791 | chr13q34 |
| 219618_at | interleukin-1 receptor-associated kinase 4 | IRAK4 | NM_016123 | Hs.138499 | chr12q12 |
| 219616_at | FLJ21963 protein | FLJ21963 | NM_024560 | Hs.13222 | chr12q21.31 |
| 219603_s_at | zinc finger protein 226 | ZNF226 | NM_015919 | Hs.145956 | chr19q13.2 |
| 219599_at | hypothetical protein PRO1843 | PRO1843 | NM_018507 |  |  |
| 219598_s_at | RWD domain containing 1 | RWDD1 | NM_016104 | Hs.22679 | chr6q13-q22.33 |
| 219596_at | THAP domain containing 10 | THAP10 | NM_020147 | Hs.26815 | chr15q23 |
| 219588_s_at | non-SMC condensin II complex, subunit G2 | NCAPG2 | NM_017760 | Hs.18616 | chr7q36.3 |
| 219582_at | opioid growth factor receptor-like 1 | OGFRL1 | NM_024576 | Hs.16512 | chr6q13 |
| 219575_s_at | peptide deformylase (mitochondrial) /// component of oligomeric golgi complex 8 | PDF | NM_022341 | Hs.130849 | chr16q22.1 |
| 219573_at | leucine rich repeat containing 16 | LRRC16 | NM_017640 | Hs.116470 | chr6p22.2 |
| 219572_at | Ca2+-dependent activator protein for secretion 2 | CADPS2 | NM_017954 | Hs.270432 | chr7q31.32 |
| 219570_at | chromosome 20 open reading frame 23 | C20orf23 | NM_024704 | Hs.101774 | chr20p11.23 |
| 219569_s_at | transmembrane protein 22 | TMEM22 | NM_025246 | Hs.101257 | chr3q22.3 |
| 219565_at | cytochrome P450, family 20, subfamily A, polypeptide 1 | CYP20A1 | NM_020674 | Hs.446065 | chr2q33.2 |
| 219559_at | chromosome 20 open reading frame 59 | C20orf59 | NM_022082 | Hs.512686 | chr20q13.33 |
| 219557_s_at | nuclear receptor interacting protein 3 | NRIP3 | NM_020645 | Hs.32017 | chr11p15.3 |
| 219549_s_at | reticulon 3 | RTN3 | NM_006054 | Hs.404434 | chr11q13 |
| 219548_at | zinc finger protein 16 | ZNF16 | NM_006958 | Hs.23019 | chr8q24 |
| 219543_at | phenazine biosynthesis-like protein domain containing | PBLD | NM_022129 | Hs.16341 | chr10pter-q25.3 |
| 219542_at | NIMA (never in mitosis gene a)- related kinase 11 | NEK11 | NM_024800 | Hs.200813 | chr3q21.3 |
| 219540_at | zinc finger protein 267 | ZNF267 | AU150728 | Hs.145498 | chr16p11.2 |
| 219539_at | gem (nuclear organelle) associated protein 6 | GEMIN6 | NM_024775 | Hs.143818 | chr2p22.1 |
| 219532_at | elongation of very long chain fatty acids (FEN1/Elo2, SUR4/Elo3, yeast)-like 4 | ELOVL4 | NM_022726 | Hs.101915 | chr6q14 |
| 219530_at | partner and localizer of BRCA2 | PALB2 | NM_024675 | Hs.444664 | chr16p12.1 |
| 219526_at | chromosome 14 open reading frame 169 | C14orf169 | NM_024644 | Hs.48938 | chr14q24.3 |
| 219525_at | hypothetical protein FLJ10847 | FLJ10847 | NM_018242 | Hs.48403 | chr17p11.2 |
| 219523_s_at | odz, odd Oz/ten-m homolog 3 (Drosophila) | ODZ3 | NM_018104 | Hs.41793 | chr4q35.1 |
| 219522_at | four jointed box 1 (Drosophila) | FJX1 | NM_014344 | Hs.39384 | chr11p13 |
| 219520_s_at | WWC family member 3 | WWC3 | NM_018458 | Hs.12913 | chrxp22.32 |
| 219515_at | PR domain containing 10 | PRDM10 | NM_020228 | Hs.275086 | chr11q25 |
| 219512_at | DSN1, MIND kinetochore complex component, homolog (S. cerevisiae) | DSN1 | NM_024918 | Hs.266273 | chr20q11.23 |
| 219505_at | cat eye syndrome chromosome region, candidate 1 | CECR1 | NM_017424 | Hs.170310 | chr22q11.2 |
| 219501_at | proliferation-inducing protein 38 | RP11-301I17.1 | NM_017993 | Hs.13209 | chr13q14.11 |
| 219496_at | ankyrin repeat domain 57 | ANKRD57 | NM_023016 | Hs.534561 | chr2q13 |
| 219495_s_at | zinc finger protein 180 | ZNF180 | NM_013256 | Hs.130683 | chr19q13.2 |
| 219493_at | SHC SH2-domain binding protein 1 | SHCBP1 | NM_024745 | Hs.123253 | chr16q11.2 |
| 219492_at | cysteine-rich hydrophobic domain 2 | CHIC2 | NM_012110 | Hs.259209 | chr4q11 |
| 219491_at | leucine rich repeat and fibronectin type III domain containing 4 | LRFN4 | NM_024036 | Hs.148438 | chr11q13.2 |
| 219489_s_at | nucleoredoxin | NXN | NM_017821 | Hs.437447 | chr17p13.3 |
| 219487_at | Bardet-Biedl syndrome 10 | BBS10 | NM_024685 | Hs.96322 | chr12q21.2 |
| 219484_at | host cell factor C2 | HCFC2 | NM_013320 | Hs.55601 | chr12q23.3 |
| 219483_s_at | porcupine homolog (Drosophila) | PORCN | NM_022825 | Hs.386453 | chrxp11.23 |
| 219479_at | KDEL (Lys-Asp-Glu-Leu) containing 1 | KDELC1 | NM_024089 | Hs.408629 | chr13q33 |
| 219475_at | oxidative stress induced growth inhibitor 1 | OSGIN1 | NM_013370 | Hs.528383 | chr16q23.3 |
| 219474_at | chromosome 3 open reading frame 52 | C3orf52 | NM_024616 | Hs.434247 | chr3q13.2 |
| 219470_x_at | cyclin J | CCNJ | NM_019084 | Hs.281348 | chr10pter-q26.12 |
| 219469_at | dynein, cytoplasmic 2, heavy chain 1 | DYNC2H1 | NM_024606 | Hs.533731 | chr11q21-q22.1 |
| 219462_at | transmembrane protein 53 | TMEM53 | NM_024587 | Hs.22157 | chr1p34.1 |
| 219459_at | polymerase (RNA) III (DNA directed) polypeptide B | POLR3B | NM_018082 | Hs.102842 | chr12q23.3 |
| 219458_s_at | NOL1/NOP2/Sun domain family, member 3 | NSUN3 | NM_022072 | Hs.401062 | chr3q11.2 |
| 219453_at | chromosome 16 open reading frame 44 | C16orf44 | NM_024731 | Hs.222731 | chr16q24.1 |
| 219451_at | methionine sulfoxide reductase B2 | MSRB2 | NM_016064 | Hs.461420 | chr10p12 |
| 219447_s_at | solute carrier family 35, member C2 | SLC35C2 | NM_015945 | Hs.348381 | chr20q13.12 |
| 219446_at | resistance to inhibitors of cholinesterase 8 homolog B (C. elegans) | RIC8B | NM_018157 | Hs.370478 | chr12q23.3 |
| 219444_at | BCL6 co-repressor-like 1 | BCORL1 | NM_021946 | Hs.8929 | chrxq25-q26.1 |
| 219443_at | taspase, threonine aspartase, 1 | TASP1 | NM_017714 | Hs.88367 | chr20p12.1 |
| 219440_at | retinoic acid induced 2 | RAI2 | NM_021785 | Hs.49597 | chrxp22 |
| 219439_at | core 1 synthase, glycoprotein-N-acetylgalactosamine 3-beta-galactosyltransferase, 1 | C1GALT1 | NM_020156 | Hs.239666 | chr7p14-p13 |
| 219431_at | Rho GTPase activating protein 10 | ARHGAP10 | NM_024605 | Hs.127602 | chr4q31.23 |
| 219428_s_at | peroxisomal membrane protein 4, 24kDa | PXMP4 | BF057649 | Hs.368717 | chr20q11.22 |
| 219427_at | FAT tumor suppressor homolog 4 (Drosophila) | FAT4 | NM_024582 | Hs.25201 | chr4q28.1 |
| 219426_at | eukaryotic translation initiation factor 2C, 3 | EIF2C3 | NM_024852 | Hs.189829 | chr1p34.3 |
| 219421_at | tetratricopeptide repeat domain 33 | TTC33 | NM_012382 | Hs.130904 | chr5p15.2-p12 |
| 219420_s_at | chromosome 1 open reading frame 163 | C1orf163 | NM_023077 | Hs.349905 | chr1p32.3 |
| 219414_at | calsyntenin 2 | CLSTN2 | NM_022131 | Hs.12079 | chr3q23-q24 |
| 219410_at | transmembrane protein 45A | TMEM45A | NM_018004 | Hs.126598 | chr3q12.2 |
| 219409_at | Smad nuclear interacting protein 1 | SNIP1 | NM_024700 | Hs.99601 | chr1p34.3 |
| 219406_at | chromosome 1 open reading frame 50 | C1orf50 | NM_024097 | Hs.148845 | chr1p34.2 |
| 219403_s_at | heparanase | HPSE | NM_006665 | Hs.44227 | chr4q21.3 |
| 219399_at | lin-7 homolog C (C. elegans) | LIN7C | NM_018362 | Hs.91393 | chr11p14 |
| 219397_at | coenzyme Q10 homolog B (S. cerevisiae) | COQ10B | NM_025147 | Hs.288945 | chr2q33.1 |
| 219392_x_at | proline rich 11 | PRR11 | NM_018304 | Hs.528654 | chr17q23.2 |
| 219390_at | FK506 binding protein 14, 22 kDa | FKBP14 | NM_017946 | Hs.390838 | chr7p15.1 |
| 219384_s_at | adenosine deaminase, tRNA-specific 1 | ADAT1 | NM_012091 | Hs.188661 | chr16q23.1 |
| 219382_at | SERTA domain containing 3 | SERTAD3 | NM_013368 | Hs.169138 | chr19q13.2 |
| 219378_at | NMDA receptor regulated 1-like | NARG1L | NM_024561 | Hs.13277 | chr13q14.11 |
| 219377_at | family with sequence similarity 59, member A | FAM59A | NM_022751 | Hs.12727 | chr18q12.1 |
| 219376_at | zinc finger protein 322B | ZNF322B | NM_024639 | Hs.530271 | chr9q22.33 |
| 219375_at | choline/ethanolamine phosphotransferase 1 | CEPT1 | NM_006090 | Hs.363572 | chr1p13.3 |
| 219374_s_at | asparagine-linked glycosylation 9 homolog (S. cerevisiae, alpha- 1,2-mannosyltransferase) | ALG9 | NM_024740 | Hs.120824 | chr11q23 |
| 219373_at | dolichyl-phosphate mannosyltransferase polypeptide 3 | DPM3 | NM_018973 | Hs.110477 | chr1q22 |
| 219372_at | intraflagellar transport 81 homolog (Chlamydomonas) | IFT81 | NM_014055 | Hs.528382 | chr12q24.13 |
| 219371_s_at | Kruppel-like factor 2 (lung) | KLF2 | NM_016270 | Hs.107740 | chr19p13.13-p13.11 |
| 219368_at | nucleosome assembly protein 1-like 2 | NAP1L2 | NM_021963 | Hs.66180 | chrxq13 |
| 219366_at | apoptosis, caspase activation inhibitor | AVEN | NM_020371 | Hs.63168 | chr15q13.1 |
| 219363_s_at | MTERF domain containing 1 | MTERFD1 | NM_015942 | Hs.46680 | chr8q22.1 |
| 219362_at | MAK10 homolog, amino-acid N-acetyltransferase subunit, (S. cerevisiae) | MAK10 | NM_024635 | Hs.325923 | chr9q21.33 |
| 219357_at | GTP binding protein 1 | GTPBP1 | NM_014027 | Hs.283677 | chr22q13.1 |
| 219352_at | hect domain and RLD 6 | HERC6 | NM_017912 | Hs.435365 | chr4q22.1 |
| 219351_at | trafficking protein particle complex 2 | TRAPPC2 | NM_014563 | Hs.472258 | chrxp22 |
| 219350_s_at | diablo homolog (Drosophila) | DIABLO | NM_019887 | Hs.169611 | chr12q24.31 |
| 219349_s_at | exocyst complex component 2 | EXOC2 | NM_018303 | Hs.436664 | chr6p25.3 |
| 219347_at | nudix (nucleoside diphosphate linked moiety X)-type motif 15 | NUDT15 | NM_018283 | Hs.144407 | chr13q14.2 |
| 219345_at | bolA homolog 1 (E. coli) | BOLA1 | NM_016074 | Hs.13880 | chr1p36.13-q31.3 |
| 219342_at | CAS1 domain containing 1 | CASD1 | NM_022900 | Hs.324725 | chr7q21.3 |
| 219338_s_at | leucine rich repeat containing 49 | LRRC49 | NM_017691 | Hs.12692 | chr15q23 |
| 219335_at | armadillo repeat containing, X-linked 5 | ARMCX5 | NM_022838 | Hs.119699 | chrxq22.1-q22.3 |
| 219331_s_at | kelch domain containing 8A | KLHDC8A | NM_018203 | Hs.10414 | chr1q32.1 |
| 219330_at | vang-like 1 (van gogh, Drosophila) | VANGL1 | NM_024062 | Hs.99598 | chr1p11-p13.1 |
| 219329_s_at | chromosome 2 open reading frame 28 | C2orf28 | NM_016085 | Hs.9527 | chr2p23.3 |
| 219326_s_at | UDP-GlcNAc:betaGal beta-1,3-N-acetylglucosaminyltransferase 2 | B3GNT2 | NM_006577 | Hs.173203 | chr2p15 |
| 219325_s_at | elaC homolog 1 (E. coli) | ELAC1 | NM_018696 | Hs.47572 | chr18q21 |
| 219321_at | membrane protein, palmitoylated 5 (MAGUK p55 subfamily member 5) | MPP5 | NM_022474 | Hs.109122 | chr14q23.3 |
| 219310_at | chromosome 20 open reading frame 39 | C20orf39 | NM_024893 | Hs.233634 | chr20p11.21 |
| 219307_at | prenyl (decaprenyl) diphosphate synthase, subunit 2 | PDSS2 | NM_020381 | Hs.268733 | chr6q21 |
| 219304_s_at | platelet derived growth factor D | PDGFD | NM_025208 | Hs.112885 | chr11q22.3 |
| 219303_at | chromosome 13 open reading frame 7 | C13orf7 | NM_024546 | Hs.439014 | chr13q31.1 |
| 219299_at | tRNA methyltransferase 12 homolog (S. cerevisiae) | TRMT12 | NM_017956 | Hs.9925 | chr8q24.13 |
| 219298_at | enoyl Coenzyme A hydratase domain containing 3 | ECHDC3 | NM_024693 | Hs.98855 | chr10p14 |
| 219297_at | WD repeat domain 44 | WDR44 | NM_019045 | Hs.269636 | chrxq24 |
| 219296_at | zinc finger, DHHC-type containing 13 | ZDHHC13 | NM_019028 | Hs.370978 | chr11p15.1 |
| 219295_s_at | procollagen C-endopeptidase enhancer 2 | PCOLCE2 | NM_013363 | Hs.8944 | chr3q21-q24 |
| 219293_s_at | GTP-binding protein 9 (putative) | GTPBP9 | NM_013341 | Hs.348282 | chr2q31.1 |
| 219292_at | THAP domain containing, apoptosis associated protein 1 | THAP1 | NM_018105 | Hs.7432 | chr8p11.21 |
| 219290_x_at | dual adaptor of phosphotyrosine and 3-phosphoinositides | DAPP1 | NM_014395 | Hs.62643 | chr4q25-q27 |
| 219289_at | HEAT repeat containing 3 | HEATR3 | NM_017939 | Hs.313917 | chr16q12.1 |
| 219288_at | chromosome 3 open reading frame 14 | C3orf14 | NM_020685 | Hs.47166 | chr3p14.2 |
| 219287_at | potassium large conductance calcium-activated channel, subfamily M, beta member 4 | KCNMB4 | NM_014505 | Hs.348361 | chr12q |
| 219284_at | HSPB (heat shock 27kDa) associated protein 1 | HSPBAP1 | NM_024610 | Hs.29169 | chr3q21.1 |
| 219283_at | C1GALT1-specific chaperone 1 | C1GALT1C1 | NM_014158 | Hs.134856 | chrxq24 |
| 219282_s_at | transient receptor potential cation channel, subfamily V, member 2 | TRPV2 | NM_015930 | Hs.279746 | chr17p11.2 |
| 219281_at | methionine sulfoxide reductase A | MSRA | NM_012331 | Hs.26458 | chr8p23.1 |
| 219279_at | dedicator of cytokinesis 10 | DOCK10 | NM_017718 | Hs.21126 | chr2q36.3 |
| 219275_at | programmed cell death 5 | PDCD5 | NM_004708 | Hs.443831 | chr19q12-q13.1 |
| 219269_at | homeobox containing 1 | HMBOX1 | NM_024567 | Hs.446386 | chr8p21.1-p12 |
| 219266_at | zinc finger protein 350 | ZNF350 | NM_021632 | Hs.407694 | chr19q13.41 |
| 219260_s_at | chromosome 17 open reading frame 81 | C17orf81 | NM_015362 | Hs.417029 | chr17p13.1 |
| 219258_at | TIMELESS interacting protein | TIPIN | NM_017858 | Hs.426696 | chr15q22.31 |
| 219257_s_at | sphingosine kinase 1 | SPHK1 | NM_021972 | Hs.68061 | chr17q25.2 |
| 219253_at | family with sequence similarity 11, member B | FAM11B | NM_024121 | Hs.44680 | chr2q14.2 |
| 219250_s_at | fibronectin leucine rich transmembrane protein 3 | FLRT3 | NM_013281 | Hs.41296 | chr20p11 |
| 219249_s_at | FK506 binding protein 10, 65 kDa | FKBP10 | NM_021939 | Hs.3849 | chr17q21.2 |
| 219248_at | THUMP domain containing 2 | THUMPD2 | NM_025264 | Hs.368545 | chr2p22.1 |
| 219244_s_at | mitochondrial ribosomal protein L46 | MRPL46 | NM_022163 | Hs.424264 | chr15q24-q25 |
| 219242_at | centrosomal protein 63kDa | CEP63 | NM_025180 | Hs.443301 | chr3q22.1 |
| 219237_s_at | DnaJ (Hsp40) homolog, subfamily B, member 14 | DNAJB14 | NM_024920 | Hs.266957 | chr4q23 |
| 219231_at | trimethylguanosine synthase homolog (S. cerevisiae) | TGS1 | NM_024831 | Hs.179909 | chr8q11 |
| 219230_at | transmembrane protein 100 | TMEM100 | NM_018286 | Hs.173233 | chr17q23.1 |
| 219220_x_at | mitochondrial ribosomal protein S22 | MRPS22 | NM_020191 | Hs.14074 | chr3q23 |
| 219219_at | transmembrane protein 160 | TMEM160 | NM_017854 | Hs.105606 | chr19q13.32 |
| 219217_at | asparaginyl-tRNA synthetase 2 (mitochondrial)(putative) | NARS2 | NM_024678 | Hs.90012 | chr11q14.1 |
| 219216_at | Similar to Heterogeneous nuclear ribonucleoprotein A1 (Helix-destabilizing protein) (Single-strand binding protein) (hnRNP core protein A1) (HDP-1) (Topoisomerase-inhibitor suppressed) /// Annexin A8 | LOC728643 | NM_019002 | Hs.82664 | chr2p13-p15 |
| 219215_s_at | solute carrier family 39 (zinc transporter), member 4 | SLC39A4 | NM_017767 | Hs.411274 | chr8q24.3 |
| 219214_s_at | 5', 3'-nucleotidase, cytosolic | NT5C | NM_021163 | Hs.67201 | chr17q25.1 |
| 219210_s_at | RAB8B, member RAS oncogene family | RAB8B | NM_016530 | Hs.365655 | chr15q22.2 |
| 219209_at | interferon induced with helicase C domain 1 | IFIH1 | NM_022168 | Hs.389539 | chr2p24.3-q24.3 |
| 219206_x_at | transmembrane BAX inhibitor motif containing 4 | TMBIM4 | NM_016056 | Hs.126372 | chr12q14.1-q15 |
| 219203_at | chromosome 14 open reading frame 122 | C14orf122 | NM_016049 | Hs.271614 | chr14q11.2 |
| 219202_at | rhomboid 5 homolog 2 (Drosophila) | RHBDF2 | NM_024599 |  |  |
| 219201_s_at | twisted gastrulation homolog 1 (Drosophila) | TWSG1 | NM_020648 | Hs.247302 | chr18p11.3 |
| 219200_at | FAST kinase domains 3 | FASTKD3 | NM_024091 | Hs.253699 | chr5p15.3-p15.2 |
| 219198_at | general transcription factor IIIC, polypeptide 4, 90kDa | GTF3C4 | NM_012204 | Hs.22302 | chr9q34.13 |
| 219193_at | WD repeat domain 70 | WDR70 | NM_018034 | Hs.16079 | chr5p13.2 |
| 219192_at | ubiquitin associated protein 2 | UBAP2 | NM_018449 | Hs.14953 | chr9p13.3 |
| 219189_at | F-box and leucine-rich repeat protein 6 | FBXL6 | NM_024555 | Hs.12271 | chr8q24.3 |
| 219188_s_at | LRP16 protein | LRP16 | NM_014067 | Hs.10824 | chr11q11 |
| 219187_at | FK506 binding protein like | FKBPL | NM_022110 | Hs.99134 | chr6p21.3 |
| 219186_at | zinc finger and BTB domain containing 7A | ZBTB7A | NM_020224 | Hs.446424 | chr19p13.3 |
| 219185_at | sirtuin (silent mating type information regulation 2 homolog) 5 (S. cerevisiae) | SIRT5 | NM_012241 | Hs.282331 | chr6p23 |
| 219184_x_at | translocase of inner mitochondrial membrane 22 homolog (yeast) | TIMM22 | NM_013337 | Hs.87595 | chr17p13 |
| 219182_at | hypothetical protein FLJ22167 | FLJ22167 | NM_024533 | Hs.156784 | chr16q22.3 |
| 219181_at | lipase, endothelial | LIPG | NM_006033 | Hs.65370 | chr18q21.1 |
| 219180_s_at | peroxisome biogenesis factor 26 | PEX26 | AI817074 | Hs.444960 | chr22q11.21 |
| 219179_at | dapper, antagonist of beta-catenin, homolog 1 (Xenopus laevis) | DACT1 | NM_016651 | Hs.48950 | chr14q23.1 |
| 219178_at | queuine tRNA-ribosyltransferase domain containing 1 | QTRTD1 | NM_024638 | Hs.407138 | chr3q13.31 |
| 219177_at | brix domain containing 2 | BXDC2 | NM_018321 | Hs.38114 | chr5p13.2 |
| 219176_at | chromosome 2 open reading frame 47 | C2orf47 | NM_024520 | Hs.3592 | chr2q33.1 |
| 219169_s_at | transcription factor B1, mitochondrial | TFB1M | NM_016020 | Hs.279908 | chr6q25.1-q25.3 |
| 219166_at | chromosome 14 open reading frame 104 | C14orf104 | NM_018139 | Hs.265960 | chr14q21.3 |
| 219165_at | PDZ and LIM domain 2 (mystique) | PDLIM2 | NM_021630 | Hs.521443 | chr8p21.2 |
| 219164_s_at | chromosome 14 open reading frame 103 | C14orf103 | NM_018036 | Hs.168241 | chr14q32.2 |
| 219163_at | zinc finger protein 562 | ZNF562 | NM_017656 | Hs.165948 | chr19p13.2 |
| 219161_s_at | chemokine-like factor | CKLF | NM_016951 | Hs.15159 | chr16q22.1 |
| 219157_at | kelch-like 2, Mayven (Drosophila) | KLHL2 | NM_007246 | Hs.122967 | chr4q21.2 |
| 219149_x_at | debranching enzyme homolog 1 (S. cerevisiae) | DBR1 | NM_016216 | Hs.118194 | chr3q22.3 |
| 219147_s_at | chromosome 9 open reading frame 95 | C9orf95 | NM_017881 | Hs.232459 | chr9q21.13 |
| 219133_at | 3-oxoacyl-ACP synthase, mitochondrial | OXSM | NM_017897 | Hs.55781 | chr3p24.2 |
| 219132_at | pellino homolog 2 (Drosophila) | PELI2 | NM_021255 | Hs.105103 | chr14q21 |
| 219131_at | UbiA prenyltransferase domain containing 1 | UBIAD1 | NM_013319 | Hs.443567 | chr1pter |
| 219130_at | coiled-coil domain containing 76 | CCDC76 | NM_019083 | Hs.40337 | chr1pter-q31.3 |
| 219129_s_at | SAP30-like | SAP30L | NM_024632 | Hs.120425 | chr5q33.2 |
| 219128_at | chromosome 2 open reading frame 42 | C2orf42 | NM_017880 | Hs.413123 | chr2p13.3 |
| 219126_at | PHD finger protein 10 | PHF10 | NM_018288 | Hs.435933 | chr6q27 |
| 219125_s_at | recombination activating gene 1 activating protein 1 | RAG1AP1 | NM_018845 | Hs.292154 | chr1q22 |
| 219124_at | chromosome 8 open reading frame 41 | C8orf41 | NM_025115 | Hs.534577 | chr8p12 |
| 219123_at | zinc finger protein 232 | ZNF232 | NM_014519 | Hs.279914 | chr17p13-p12 |
| 219122_s_at | tRNA-histidine guanylyltransferase 1-like (S. cerevisiae) | THG1L | NM_017872 | Hs.353090 | chr5q33.3 |
| 219119_at | LSM8 homolog, U6 small nuclear RNA associated (S. cerevisiae) | LSM8 | NM_016200 | Hs.446179 | chr7q31.1-q31.3 |
| 219117_s_at | FK506 binding protein 11, 19 kDa | FKBP11 | NM_016594 | Hs.438695 | chr12q13.12 |
| 219111_s_at | DEAD (Asp-Glu-Ala-Asp) box polypeptide 54 | DDX54 | NM_024072 | Hs.203581 | chr12q24.13 |
| 219110_at | nucleolar protein family A, member 1 (H/ACA small nucleolar RNPs) | NOLA1 | NM_018983 | Hs.69851 | chr4q25 |
| 219109_at | sperm associated antigen 16 | SPAG16 | NM_024532 | Hs.6783 | chr2q34 |
| 219105_x_at | origin recognition complex, subunit 6 like (yeast) | ORC6L | NM_014321 | Hs.49760 | chr16q12 |
| 219104_at | ring finger protein 141 | RNF141 | NM_016422 | Hs.44685 | chr11p15.4 |
| 219102_at | reticulocalbin 3, EF-hand calcium binding domain | RCN3 | NM_020650 | Hs.439184 | chr19q13.33 |
| 219100_at | oligonucleotide/oligosaccharide-binding fold containing 1 | OBFC1 | NM_024928 | Hs.134491 | chr10q25.1 |
| 219099_at | chromosome 12 open reading frame 5 | C12orf5 | NM_020375 | Hs.24792 | chr12p13.3 |
| 219098_at | MYB binding protein (P160) 1a | MYBBP1A | NM_014520 | Hs.22824 | chr17p13.3 |
| 219093_at | hypothetical protein FLJ20701 /// hypothetical protein FLJ20701 | FLJ20701 | NM_017933 | Hs.424598 | chr2q36.3 |
| 219089_s_at | zinc finger protein 576 | ZNF576 | NM_024327 | Hs.11110 | chr19q13.31 |
| 219086_at | chromosome 14 open reading frame 131 | C14orf131 | NM_018335 | Hs.106005 | chr14q32.32 |
| 219081_at | ankyrin repeat and KH domain containing 1 | ANKHD1 | NM_024668 | Hs.406840 | chr5q31.2 |
| 219079_at | cytochrome b5 reductase 4 | CYB5R4 | NM_016230 | Hs.5741 | chr6pter-q22.33 |
| 219077_s_at | WW domain containing oxidoreductase | WWOX | NM_016373 | Hs.461453 | chr16q23.3-q24.1 |
| 219074_at | transmembrane protein 34 | TMEM34 | NM_018241 | Hs.203896 | chr4q31.23 |
| 219070_s_at | motile sperm domain containing 3 | MOSPD3 | NM_023948 | Hs.296200 | chr7q22 |
| 219069_at | ankyrin repeat domain 49 | ANKRD49 | NM_017704 | Hs.29052 | chr11q21 |
| 219065_s_at | chromosome 2 open reading frame 4 | C2orf4 | NM_015955 | Hs.355622 | chr2p22-p21 |
| 219064_at | inter-alpha (globulin) inhibitor H5 | ITIH5 | NM_030569 | Hs.245326 | chr10p14 |
| 219063_at | chromosome 1 open reading frame 35 | C1orf35 | NM_024319 | Hs.445952 | chr1q42.13 |
| 219062_s_at | zinc finger, CCHC domain containing 2 | ZCCHC2 | NM_017742 | Hs.254672 | chr18q21.33 |
| 219061_s_at | L antigen family, member 3 | LAGE3 | NM_006014 | Hs.444619 | chrxq28 |
| 219060_at | chromosome 8 open reading frame 32 | C8orf32 | NM_018024 | Hs.18029 | chr8q24.13 |
| 219055_at | S1 RNA binding domain 1 | SRBD1 | NM_018079 | Hs.14229 | chr2p21 |
| 219054_at | chromosome 5 open reading frame 23 | C5orf23 | NM_024563 | Hs.13528 | chr5p13.3 |
| 219053_s_at | vacuolar protein sorting 37 homolog C (S. cerevisiae) | VPS37C | NM_017966 | Hs.376047 | chr11q12.2 |
| 219052_at | Hermansky-Pudlak syndrome 6 | HPS6 | NM_024747 | Hs.125133 | chr10q24.32 |
| 219049_at | chondroitin beta1,4 N-acetylgalactosaminyltransferase | ChGn | NM_018371 | Hs.341073 | chr8p21.3 |
| 219045_at | ras homolog gene family, member F (in filopodia) | RHOF | NM_019034 | Hs.512618 | chr12q24.31 |
| 219043_s_at | phosducin-like 3 /// phosducin-like 3 pseudogene /// similar to phosducin-like 3 | PDCL3 | NM_024065 | Hs.534733 | chr3q12.3 /// chr2q11.2 |
| 219041_s_at | replication initiator 1 | REPIN1 | NM_014374 | Hs.90693 | chr7q36.1 |
| 219038_at | MORC family CW-type zinc finger 4 | MORC4 | NM_024657 | Hs.61763 | chrxq22.3 |
| 219037_at | CGI-115 protein | CGI-115 | NM_016052 | Hs.408101 | chr1q41 |
| 219035_s_at | ring finger protein 34 | RNF34 | NM_025126 | Hs.292804 | chr12q24.31 |
| 219033_at | poly (ADP-ribose) polymerase family, member 8 | PARP8 | NM_024615 | Hs.310185 | chr5q11.1 |
| 219030_at | TP53RK binding protein | TPRKB | NM_016058 | Hs.157401 | chr2p24.3-p24.1 |
| 219029_at | chromosome 5 open reading frame 28 | C5orf28 | NM_022483 | Hs.294144 | chr5p12 |
| 219027_s_at | myosin IXA | MYO9A | NM_006901 | Hs.112237 | chr15q22-q23 |
| 219023_at | chromosome 4 open reading frame 16 | C4orf16 | NM_018569 | Hs.435991 | chr4q25 |
| 219022_at | chromosome 12 open reading frame 43 | C12orf43 | NM_022895 | Hs.528671 | chr12q |
| 219021_at | ring finger protein 121 | RNF121 | NM_018320 | Hs.434860 | chr11q13.4 |
| 219020_at | HCLS1 binding protein 3 | HS1BP3 | NM_022460 | Hs.288410 | chr2p24.1 |
| 219016_at | FAST kinase domains 5 | FASTKD5 | NM_021826 |  | chr20p13 |
| 219015_s_at | asparagine-linked glycosylation 13 homolog (S. cerevisiae) | ALG13 | NM_018466 | Hs.110853 | chrxq23 |
| 219013_at | UDP-N-acetyl-alpha-D-galactosamine:polypeptide N-acetylgalactosaminyltransferase 11 (GalNAc-T11) | GALNT11 | NM_022087 | Hs.314421 | chr7q34-q36 |
| 219007_at | nucleoporin 43kDa | NUP43 | NM_024647 | Hs.53263 | chr6q25.1 |
| 219006_at | chromosome 6 open reading frame 66 | C6orf66 | NM_014165 | Hs.406328 | chr6q16.1 |
| 219003_s_at | mannosidase, endo-alpha | MANEA | NM_024641 | Hs.46903 | chr6q16.1 |
| 219002_at | FAST kinase domains 1 | FASTKD1 | NM_024622 | Hs.369475 | chr2q31 |
| 218998_at | chromosome 9 open reading frame 6 | C9orf6 | NM_017832 | Hs.29276 | chr9q31.3 |
| 218997_at | polymerase (RNA) I polypeptide E, 53kDa | POLR1E | NM_022490 | Hs.24884 | chr9p13.2 |
| 218994_s_at | stromal antigen 3-like | FLJ13195 | NM_022906 | Hs.213392 | chr7p11.2-q11.2 |
| 218993_at | RNA methyltransferase like 1 | RNMTL1 | NM_018146 | Hs.182729 | chr17p13.3 |
| 218992_at | chromosome 9 open reading frame 46 | C9orf46 | NM_018465 | Hs.416649 | chr9p24.1 |
| 218991_at | amplified in breast cancer 1 | ABC1 | NM_022070 | Hs.17230 | chr17q23.2 |
| 218988_at | solute carrier family 35, member E3 | SLC35E3 | NM_018656 | Hs.445043 | chr12q15 |
| 218987_at | activating transcription factor 7 interacting protein | ATF7IP | NM_018179 | Hs.272210 | chr12p13.1 |
| 218986_s_at | hypothetical protein FLJ20035 | FLJ20035 | NM_017631 | Hs.109309 | chr4q32.3 |
| 218984_at | pseudouridylate synthase 7 homolog (S. cerevisiae) | PUS7 | NM_019042 | Hs.375641 | chr7q22.3 |
| 218983_at | complement component 1, r subcomponent-like | C1RL | NM_016546 | Hs.415792 | chr12p13.31 |
| 218982_s_at | mitochondrial ribosomal protein S17 | MRPS17 | NM_015969 | Hs.44298 | chr7p11 |
| 218981_at | ACN9 homolog (S. cerevisiae) | ACN9 | NM_020186 | Hs.42785 | chr7q21.3 |
| 218980_at | formin homology 2 domain containing 3 | FHOD3 | NM_025135 | Hs.444746 | chr18q12 |
| 218979_at | RMI1, RecQ mediated genome instability 1, homolog (S. cerevisiae) | RMI1 | NM_024945 | Hs.284137 | chr9q21.32 |
| 218977_s_at | tRNA selenocysteine associated protein 1 | TRSPAP1 | NM_017846 | Hs.266935 | chr1p35.3 |
| 218974_at | Hypothetical protein FLJ10159 | FLJ10159 | NM_018013 | Hs.346203 | chr6q21 |
| 218973_at | elongation factor Tu GTP binding domain containing 1 | EFTUD1 | NM_024580 | Hs.19348 | chr15q25.2 |
| 218972_at | tetratricopeptide repeat domain 17 | TTC17 | NM_018259 | Hs.17283 | chr11p12-p11.2 |
| 218970_s_at | cutC copper transporter homolog (E. coli) | CUTC | NM_015960 | Hs.16606 | chr10q24.2 |
| 218969_at | mitochondria-associated protein involved in granulocyte-macrophage colony-stimulating factor signal transduction | Magmas | NM_016069 | Hs.437957 | chr16p13.3 |
| 218968_s_at | zinc finger protein 64 homolog (mouse) | ZFP64 | NM_018197 | Hs.504892 | chr20q13.2 |
| 218962_s_at | transmembrane protein 168 | TMEM168 | NM_022484 | Hs.242327 | chr7q31.32 |
| 218961_s_at | polynucleotide kinase 3'-phosphatase | PNKP | NM_007254 | Hs.78016 | chr19q13.3-q13.4 |
| 218957_s_at | WD repeat domain 71 | WDR71 | NM_025155 | Hs.408526 | chr11q13.4 |
| 218955_at | BRF2, subunit of RNA polymerase III transcription initiation factor, BRF1-like | BRF2 | NM_018310 | Hs.274136 | chr8p12 |
| 218949_s_at | glutaminyl-tRNA synthase (glutamine-hydrolyzing)-like 1 | QRSL1 | NM_018292 | Hs.406917 | chr6q21 |
| 218947_s_at | PAP associated domain containing 1 | PAPD1 | NM_018109 | Hs.173946 | chr10p11.23 |
| 218946_at | NFU1 iron-sulfur cluster scaffold homolog (S. cerevisiae) | NFU1 | NM_015700 | Hs.430439 | chr2p15-p13 |
| 218945_at | chromosome 16 open reading frame 68 | C16orf68 | NM_024109 | Hs.306380 | chr16p13.2 |
| 218942_at | phosphatidylinositol-4-phosphate 5-kinase, type II, gamma | PIP5K2C | NM_024779 | Hs.144502 | chr12q13.3 |
| 218940_at | chromosome 14 open reading frame 138 | C14orf138 | NM_024558 | Hs.390504 | chr14q21.3 |
| 218938_at | F-box and leucine-rich repeat protein 15 | FBXL15 | NM_024326 | Hs.380081 | chr10q24.32 |
| 218937_at | zinc finger protein 434 | ZNF434 | NM_017810 | Hs.123295 | chr16p13.3 |
| 218936_s_at | coiled-coil domain containing 59 | CCDC59 | NM_014167 | Hs.90527 | chr12q21.31 |
| 218933_at | spermatogenesis associated 5-like 1 | SPATA5L1 | NM_024063 | Hs.5555 | chr15q21.1 |
| 218932_at | chromosome 1 open reading frame 181 | C1orf181 | NM_017953 | Hs.5111 | chr1p22.3 |
| 218929_at | CDKN2A interacting protein | CDKN2AIP | NM_017632 | Hs.32922 | chr4q35.1 |
| 218926_at | myoneurin | MYNN | NM_018657 | Hs.507025 | chr3q26.2 |
| 218923_at | chitobiase, di-N-acetyl- | CTBS | AW304174 | Hs.513557 | chr1p22 |
| 218920_at | hypothetical protein FLJ10404 | FLJ10404 | NM_019057 | Hs.437404 | chr5q35.3 |
| 218919_at | zinc finger, AN1-type domain 1 | ZFAND1 | NM_024699 | Hs.390395 | chr8q21.13 |
| 218916_at | zinc finger protein 768 | ZNF768 | NM_024671 | Hs.85658 | chr16p11.2 |
| 218915_at | neurofibromin 2 (bilateral acoustic neuroma) | NF2 | NM_016418 | Hs.902 | chr22q12.2 |
| 218912_at | GRIP and coiled-coil domain containing 1 | GCC1 | NM_024523 | Hs.4783 | chr7q32.2 |
| 218911_at | YEATS domain containing 4 | YEATS4 | NM_006530 | Hs.4029 | chr12q13-q15 |
| 218909_at | ribosomal protein S6 kinase, 52kDa, polypeptide 1 | RPS6KC1 | NM_012424 | Hs.434960 | chr1q41 |
| 218905_at | integrator complex subunit 8 | INTS8 | NM_017864 | Hs.370888 | chr8q22.1 |
| 218903_s_at | oligonucleotide/oligosaccharide-binding fold containing 2B | OBFC2B | NM_024068 | Hs.240170 | chr12q13.3 |
| 218901_at | phospholipid scramblase 4 | PLSCR4 | NM_020353 | Hs.182538 | chr3q24 |
| 218898_at | family with sequence similarity 57, member A | FAM57A | NM_024792 | Hs.154396 | chr17p13.3 |
| 218897_at | transmembrane protein 177 | TMEM177 | NM_030577 | Hs.151930 | chr2q14.2 |
| 218895_at | G patch domain containing 3 | GPATCH3 | NM_022078 | Hs.10903 | chr1p35.3-p35.1 |
| 218894_s_at | mago-nashi homolog 2 | FLJ10292 | NM_018048 | Hs.104650 | chr12p13.2 |
| 218893_at | isochorismatase domain containing 2 | ISOC2 | NM_024710 | Hs.103833 | chr19q13.42 |
| 218889_at | nucleolar complex associated 3 homolog (S. cerevisiae) | NOC3L | NM_022451 | Hs.74899 | chr10q23.33 |
| 218886_at | PAK1 interacting protein 1 /// chromosome 20 open reading frame 7 | PAK1IP1 | NM_017906 | Hs.310231 | chr6p24.1 |
| 218884_s_at | GUF1 GTPase homolog (S. cerevisiae) | GUF1 | NM_021927 | Hs.21398 | chr4p13 |
| 218883_s_at | MLF1 interacting protein | MLF1IP | NM_024629 | Hs.38178 | chr4q35.1 |
| 218882_s_at | WD repeat domain 3 | WDR3 | NM_006784 | Hs.201375 | chr1p13-p12 |
| 218878_s_at | sirtuin (silent mating type information regulation 2 homolog) 1 (S. cerevisiae) | SIRT1 | NM_012238 | Hs.31176 | chr10q21.3 |
| 218877_s_at | tRNA methyltransferase 11 homolog (S. cerevisiae) | TRMT11 | NM_021820 | Hs.282575 | chr6q11.1-q22.33 |
| 218875_s_at | F-box protein 5 | FBXO5 | NM_012177 | Hs.272027 | chr6q25-q26 |
| 218869_at | malonyl-CoA decarboxylase | MLYCD | NM_012213 | Hs.150748 | chr16q24 |
| 218868_at | ARP3 actin-related protein 3 homolog B (yeast) | ACTR3B | NM_020445 | Hs.12887 | chr7q32-q36 |
| 218867_s_at | chromosome 12 open reading frame 49 | C12orf49 | NM_024738 | Hs.528681 | chr12q24.22 |
| 218866_s_at | polymerase (RNA) III (DNA directed) polypeptide K, 12.3 kDa | POLR3K | NM_016310 | Hs.437186 | chr16p13.3 |
| 218862_at | ankyrin repeat and SOCS box-containing 13 | ASB13 | NM_024701 | Hs.300063 | chr10p15.1 |
| 218861_at | ring finger protein 25 | RNF25 | NM_022453 | Hs.97176 | chr2q35 |
| 218860_at | nucleolar complex associated 4 homolog (S. cerevisiae) | NOC4L | NM_024078 | Hs.95196 | chr12q24.33 |
| 218858_at | DEP domain containing 6 | DEPDC6 | NM_022783 | Hs.303788 | chr8q24.12 |
| 218856_at | tumor necrosis factor receptor superfamily, member 21 | TNFRSF21 | NM_016629 | Hs.159651 | chr6p21.1-12.2 |
| 218854_at | squamous cell carcinoma antigen recognized by T cells 2 | SART2 | NM_013352 | Hs.388014 | chr6q22 |
| 218852_at | chromosome 14 open reading frame 10 | C14orf10 | NM_017917 | Hs.419151 | chr14q13.2 |
| 218849_s_at | protein phosphatase 1, regulatory (inhibitor) subunit 13 like | PPP1R13L | NM_006663 | Hs.324051 | chr19q13.32 |
| 218848_at | THO complex 6 homolog (Drosophila) | THOC6 | NM_024339 | Hs.412304 | chr16p13.3 |
| 218847_at | insulin-like growth factor 2 mRNA binding protein 2 | IGF2BP2 | NM_006548 | Hs.30299 | chr3q27.2 |
| 218846_at | cofactor required for Sp1 transcriptional activation, subunit 3, 130kDa | CRSP3 | NM_004830 | Hs.29679 | chr6q22.33-q24.1 |
| 218845_at | dual specificity phosphatase 22 | DUSP22 | NM_020185 | Hs.29106 | chr6p25.3 |
| 218843_at | fibronectin type III domain containing 4 | FNDC4 | NM_022823 | Hs.27836 | chr2p23.3 |
| 218842_at | hypothetical protein FLJ21908 | FLJ21908 | NM_024604 | Hs.323473 | chr12q13.11 |
| 218841_at | ankyrin repeat and SOCS box-containing 8 | ASB8 | NM_024095 | Hs.432699 | chr12q13.11 |
| 218838_s_at | tetratricopeptide repeat domain 31 | TTC31 | NM_022492 | Hs.20242 | chr2p13.1 |
| 218836_at | ribonuclease P 21kDa subunit | RPP21 | NM_024839 | Hs.183232 | chr6p21.33 |
| 218831_s_at | Fc fragment of IgG, receptor, transporter, alpha | FCGRT | NM_004107 | Hs.111903 | chr19q13.3 |
| 218830_at | ribosomal protein L26-like 1 | RPL26L1 | NM_016093 | Hs.430755 | chr5q35.1 |
| 218829_s_at | chromodomain helicase DNA binding protein 7 | CHD7 | NM_017780 | Hs.397426 | chr8q12.2 |
| 218826_at | solute carrier family 35, member F2 | SLC35F2 | NM_017515 | Hs.9711 | chr11q22.3 |
| 218824_at | hypothetical protein FLJ10781 | FLJ10781 | NM_018215 | Hs.8395 | chr19q13.32 |
| 218820_at | chromosome 14 open reading frame 132 | C14orf132 | NM_020215 | Hs.6434 | chr14q32.2 |
| 218817_at | signal peptidase complex subunit 3 homolog (S. cerevisiae) | SPCS3 | NM_021928 | Hs.42194 | chr4q34.2 |
| 218816_at | leucine rich repeat containing 1 | LRRC1 | NM_018214 | Hs.129694 | chr6p12.1 |
| 218812_s_at | transmembrane protein 142B | TMEM142B | NM_025156 | Hs.363308 | chr7q22.1 |
| 218802_at | coiled-coil domain containing 109B | CCDC109B | NM_017918 | Hs.234149 | chr4q25 |
| 218799_at | ATP binding domain 1 family, member B | ATPBD1B | NM_018066 | Hs.19333 | chr1p36.11 |
| 218797_s_at | sirtuin (silent mating type information regulation 2 homolog) 7 (S. cerevisiae) /// similar to NAD-dependent deacetylase sirtuin-7 (SIR2-like protein 7) | SIRT7 | NM_016538 | Hs.528641 | chr17q25 |
| 218795_at | acid phosphatase 6, lysophosphatidic | ACP6 | NM_016361 | Hs.15871 | chr1q21 |
| 218794_s_at | thioredoxin-like 4B | TXNL4B | NM_017853 | Hs.134406 | chr16q22.2 |
| 218791_s_at | chromosome 15 open reading frame 29 | C15orf29 | NM_024713 | Hs.122140 | chr15q14 |
| 218789_s_at | chromosome 11 open reading frame 71 | C11orf71 | NM_019021 | Hs.91816 | chr11q14.2-q14.3 |
| 218786_at | 5'-nucleotidase domain containing 3 | NT5DC3 | NM_016575 | Hs.374350 | chr12q22-q23.1 |
| 218783_at | integrator complex subunit 7 | INTS7 | AL133049 | Hs.385548 | chr1p36.13-q42.3 |
| 218777_at | receptor accessory protein 4 | REEP4 | NM_025232 | Hs.289063 | chr8p21.3 |
| 218776_s_at | transmembrane protein 62 | TMEM62 | NM_024956 | Hs.511175 | chr15q15.2 |
| 218774_at | decapping enzyme, scavenger | DCPS | NM_014026 | Hs.279900 | chr11q24.2 |
| 218771_at | pantothenate kinase 4 | PANK4 | NM_018216 | Hs.26156 | chr1p36.32 |
| 218770_s_at | transmembrane protein 39B | TMEM39B | NM_018056 | Hs.25544 | chr1p35.1 |
| 218769_s_at | ankyrin repeat, family A (RFXANK-like), 2 | ANKRA2 | NM_023039 | Hs.239154 | chr5q12-q13 |
| 218768_at | nucleoporin 107kDa | NUP107 | NM_020401 | Hs.355598 | chr12q15 |
| 218767_at | REX4, RNA exonuclease 4 homolog (S. cerevisiae) | REXO4 | NM_020385 | Hs.438409 | chr9q34.2 |
| 218766_s_at | tryptophanyl tRNA synthetase 2 (mitochondrial) | WARS2 | NM_015836 | Hs.398139 | chr1p13.3-p13.1 |
| 218763_at | syntaxin 18 | STX18 | NM_016930 | Hs.13406 | chr4p16.3-p16.2 |
| 218761_at | ring finger protein 111 | RNF111 | NM_017610 | Hs.12504 | chr15q21 |
| 218757_s_at | UPF3 regulator of nonsense transcripts homolog B (yeast) | UPF3B | NM_023010 | Hs.103832 | chrxq25-q26 |
| 218753_at | XK, Kell blood group complex subunit-related family, member 8 | XKR8 | NM_018053 | Hs.55024 | chr1p35.3 |
| 218751_s_at | F-box and WD-40 domain protein 7 (archipelago homolog, Drosophila) | FBXW7 | NM_018315 | Hs.312503 | chr4q31.3 |
| 218750_at | Josephin domain containing 3 | JOSD3 | NM_024116 | Hs.355750 | chr11q21 |
| 218749_s_at | solute carrier family 24 (sodium/potassium/calcium exchanger), member 6 | SLC24A6 | NM_024959 | Hs.286194 | chr12q24.13 |
| 218747_s_at | TAP binding protein-like | TAPBPL | NM_018009 | Hs.267993 | chr12p13.31 |
| 218743_at | chromatin modifying protein 6 | CHMP6 | NM_024591 | Hs.22897 | chr17q25.3 |
| 218740_s_at | CDK5 regulatory subunit associated protein 3 | CDK5RAP3 | NM_025197 | Hs.20157 | chr17q21.32 |
| 218737_at | strawberry notch homolog 1 (Drosophila) | SBNO1 | NM_018183 | Hs.306665 | chr12q24.31 |
| 218735_s_at | zinc finger protein 544 | ZNF544 | AA349848 | Hs.438994 | chr19q13.43 |
| 218734_at | N-acetyltransferase 11 | NAT11 | NM_024771 | Hs.408443 | chr11q13.1 |
| 218733_at | male-specific lethal 2-like 1 (Drosophila) | MSL2L1 | NM_018133 | Hs.129750 | chr3q22.2 |
| 218732_at | peptidyl-tRNA hydrolase 2 | PTRH2 | NM_016077 | Hs.12677 | chr17q23.2 |
| 218729_at | latexin | LXN | NM_020169 | Hs.124491 | chr3q25.32 |
| 218727_at | amino acid transporter | FLJ10815 | NM_018231 | Hs.10499 | chr16q21 |
| 218724_s_at | TGFB-induced factor 2 (TALE family homeobox) | TGIF2 | NM_021809 | Hs.94785 | chr20q11.2-q12 |
| 218723_s_at | response gene to complement 32 | RGC32 | NM_014059 | Hs.76640 | chr13q14.11 |
| 218722_s_at | coiled-coil domain containing 51 | CCDC51 | NM_024661 | Hs.187657 | chr3p21.31 |
| 218721_s_at | chromosome 1 open reading frame 27 | C1orf27 | NM_017847 | Hs.380971 | chr1q25 |
| 218719_s_at | GINS complex subunit 3 (Psf3 homolog) | GINS3 | NM_022770 | Hs.47125 | chr16q21 |
| 218717_s_at | leprecan-like 1 | LEPREL1 | NM_018192 | Hs.42824 | chr3q28 |
| 218715_at | UTP6, small subunit (SSU) processome component, homolog (yeast) | UTP6 | NM_018428 | Hs.211828 | chr17q11.2 |
| 218714_at | proline rich 14 | PRR14 | NM_024031 | Hs.293629 | chr16p11.2 |
| 218712_at | chromosome 1 open reading frame 109 | C1orf109 | NM_017850 | Hs.272673 | chr1p34.3 |
| 218710_at | tetratricopeptide repeat domain 27 | TTC27 | NM_017735 | Hs.26090 | chr2p22.3 |
| 218708_at | NTF2-like export factor 1 | NXT1 | NM_013248 | Hs.283375 | chr20p12-p11.2 |
| 218706_s_at | GRAM domain containing 3 | GRAMD3 | AW575493 | Hs.281147 | chr5q23.2 |
| 218705_s_at | sorting nexing 24 | SNX24 | NM_014035 | Hs.232779 | chr5q23.2 |
| 218701_at | lactamase, beta 2 | LACTB2 | NM_016027 | Hs.118554 | chr8p22-q22.3 |
| 218700_s_at | RAB7, member RAS oncogene family-like 1 | RAB7L1 | BC002585 | Hs.115325 | chr1q32 |
| 218698_at | APAF1 interacting protein | APIP | NM_015957 | Hs.447794 | chr11p13 |
| 218697_at | NCK interacting protein with SH3 domain | NCKIPSD | NM_016453 | Hs.102929 | chr3p21 |
| 218696_at | eukaryotic translation initiation factor 2-alpha kinase 3 | EIF2AK3 | NM_004836 | Hs.102506 | chr2p12 |
| 218694_at | armadillo repeat containing, X-linked 1 | ARMCX1 | NM_016608 | Hs.9728 | chrxq21.33-q22.2 |
| 218692_at | hypothetical protein FLJ20366 | FLJ20366 | NM_017786 | Hs.354793 | chr8q23.2 |
| 218691_s_at | PDZ and LIM domain 4 | PDLIM4 | AF153882 | Hs.424312 | chr5q31.1 |
| 218688_at | dihydroxyacetone kinase 2 homolog (S. cerevisiae) | DAK | NM_015533 | Hs.6278 | chr11q12.2 |
| 218686_s_at | rhomboid 5 homolog 1 (Drosophila) | RHBDF1 | NM_022450 | Hs.57988 | chr16p13.3 |
| 218685_s_at | single-strand-selective monofunctional uracil-DNA glycosylase 1 | SMUG1 | NM_014311 | Hs.5212 | chr12q13.11-q13.3 |
| 218684_at | leucine rich repeat containing 8 family, member D | LRRC8D | NM_018103 | Hs.199364 | chr1p22.2 |
| 218681_s_at | stromal cell-derived factor 2-like 1 | SDF2L1 | NM_022044 | Hs.303116 | chr22q11.21 |
| 218679_s_at | vacuolar protein sorting 28 homolog (S. cerevisiae) | VPS28 | NM_016208 | Hs.418175 | chr8q24.3 |
| 218676_s_at | phosphatidylcholine transfer protein | PCTP | NM_021213 | Hs.285218 | chr17q21-q24 |
| 218675_at | solute carrier family 22 (organic cation transporter), member 17 | SLC22A17 | NM_020372 | Hs.373498 | chr14q11.2 |
| 218673_s_at | ATG7 autophagy related 7 homolog (S. cerevisiae) | ATG7 | NM_006395 | Hs.38032 | chr3p25.3 |
| 218670_at | pseudouridylate synthase 1 | PUS1 | NM_025215 | Hs.23723 | chr12q24.33 |
| 218667_at | praja 1 | PJA1 | NM_022368 | Hs.21122 | chrxq13.1 |
| 218666_s_at | syntaxin 17 | STX17 | NM_017919 | Hs.130643 | chr9q31.1 |
| 218665_at | frizzled homolog 4 (Drosophila) | FZD4 | NM_012193 | Hs.19545 | chr11q14.2 |
| 218664_at | mitochondrial trans-2-enoyl-CoA reductase | MECR | NM_016011 | Hs.183646 | chr1pter-p22.3 |
| 218661_at | hypothetical protein FLJ14154 | FLJ14154 | NM_024845 | Hs.312841 | chr16p13.3 |
| 218659_at | additional sex combs like 2 (Drosophila) | ASXL2 | NM_018263 | Hs.97568 | chr2p24.1 |
| 218658_s_at | ARP8 actin-related protein 8 homolog (yeast) | ACTR8 | NM_022899 | Hs.412186 | chr3p21.31 |
| 218655_s_at | coiled-coil domain containing 49 | CCDC49 | NM_017748 | Hs.406223 | chr17q12 |
| 218654_s_at | mitochondrial ribosomal protein S33 | MRPS33 | NM_016071 | Hs.83006 | chr7q32-q34 |
| 218653_at | solute carrier family 25 (mitochondrial carrier; ornithine transporter) member 15 | SLC25A15 | NM_014252 | Hs.442781 | chr13q14 |
| 218652_s_at | phosphatidylinositol glycan anchor biosynthesis, class G | PIGG | NM_017733 | Hs.7099 | chr4p16.3 |
| 218651_s_at | La ribonucleoprotein domain family, member 6 | LARP6 | NM_018357 | Hs.416755 | chr15q23 |
| 218650_at | DiGeorge syndrome critical region gene 8 | DGCR8 | NM_022775 | Hs.528643 | chr22q11.2 |
| 218648_at | CREB regulated transcription coactivator 3 | CRTC3 | NM_022769 | Hs.434956 | chr15q26.1 |
| 218647_s_at | yrdC domain containing (E. coli) | YRDC | NM_024640 | Hs.301564 | chr1p34.3 |
| 218646_at | chromosome 4 open reading frame 27 | C4orf27 | NM_017867 | Hs.44344 | chr4q33 |
| 218644_at | pleckstrin 2 | PLEK2 | NM_016445 | Hs.170473 | chr14q23.3 |
| 218643_s_at | cysteine-rich PDZ-binding protein | CRIPT | NM_014171 | Hs.39733 | chr2p21 |
| 218642_s_at | coiled-coil-helix-coiled-coil-helix domain containing 7 | CHCHD7 | NM_024300 | Hs.436913 | chr8q12.1 |
| 218641_at | hypothetical protein MGC3032 | MGC3032 | NM_023941 |  | chr11q13 |
| 218638_s_at | spondin 2, extracellular matrix protein | SPON2 | NM_012445 | Hs.435953 | chr4p16.3 |
| 218637_at | Impact homolog (mouse) | IMPACT | NM_018439 | Hs.284245 | chr18q11.2-q12.1 |
| 218636_s_at | mannosidase, alpha, class 1B, member 1 | MAN1B1 | NM_016219 | Hs.279881 | chr9q34 |
| 218632_at | HECT domain containing 3 | HECTD3 | NM_024602 | Hs.26058 | chr1p34.1 |
| 218631_at | arginine vasopressin-induced 1 | AVPI1 | NM_021732 | Hs.23918 | chr10q24.2 |
| 218628_at | coiled-coil domain containing 53 | CCDC53 | NM_016053 | Hs.18885 | chr12q23.3 |
| 218626_at | eukaryotic translation initiation factor 4E nuclear import factor 1 | EIF4ENIF1 | NM_019843 | Hs.233939 | chr22q11.2 |
| 218622_at | nucleoporin 37kDa | NUP37 | NM_024057 | Hs.5152 | chr12q23.3 |
| 218617_at | tRNA isopentenyltransferase 1 | TRIT1 | NM_017646 | Hs.356554 | chr1p35.3-p34.1 |
| 218616_at | integrator complex subunit 12 | INTS12 | NM_020395 | Hs.432952 | chr4q24 |
| 218612_s_at | tumor suppressing subtransferable candidate 4 | TSSC4 | NM_005706 | Hs.165743 | chr11p15.5 |
| 218611_at | immediate early response 5 | IER5 | NM_016545 | Hs.15725 | chr1q25.3 |
| 218610_s_at | hypothetical protein FLJ11151 | FLJ11151 | NM_018340 | Hs.200481 | chr16p13.12 |
| 218607_s_at | SDA1 domain containing 1 | SDAD1 | NM_018115 | Hs.270107 | chr4q21.1 |
| 218606_at | zinc finger, DHHC-type containing 7 | ZDHHC7 | NM_017740 | Hs.9725 | chr16q24.1 |
| 218605_at | transcription factor B2, mitochondrial | TFB2M | NM_022366 | Hs.7395 | chr1q44 |
| 218604_at | LEM domain containing 3 | LEMD3 | NM_014319 | Hs.105234 | chr12q14 |
| 218603_at | headcase homolog (Drosophila) | HECA | NM_016217 | Hs.6679 | chr6q23-q24 |
| 218598_at | RAD50 interactor 1 | RINT1 | NM_021930 | Hs.302460 | chr7q22.3 |
| 218597_s_at | zinc finger, CDGSH-type domain 1 | ZCD1 | NM_018464 | Hs.43549 | chr10q21.1 |
| 218593_at | RNA binding motif protein 28 | RBM28 | NM_018077 | Hs.274263 | chr7q32.2 |
| 218592_s_at | cat eye syndrome chromosome region, candidate 5 | CECR5 | NM_017829 | Hs.26890 | chr22q11.2 |
| 218590_at | progressive external ophthalmoplegia 1 | PEO1 | NM_021830 | Hs.22678 | chr10q23.3-24.3 |
| 218588_s_at | chromosome 5 open reading frame 3 | C5orf3 | NM_018691 | Hs.166551 | chr5q31-q33 |
| 218587_s_at | chromosome 3 open reading frame 9 | C3orf9 | NM_020231 | Hs.231750 | chr3q13.33 |
| 218586_at | chromosome 20 open reading frame 20 | C20orf20 | NM_018270 | Hs.443260 | chr20q13.33 |
| 218584_at | tectonic | FLJ21127 | NM_024549 | Hs.412559 | chr12q24.11 |
| 218578_at | cell division cycle 73, Paf1/RNA polymerase II complex component, homolog (S. cerevisiae) | CDC73 | NM_024529 | Hs.259502 | chr1q25 |
| 218576_s_at | dual specificity phosphatase 12 | DUSP12 | NM_007240 | Hs.416216 | chr1q21-q22 |
| 218573_at | melanoma antigen family H, 1 | MAGEH1 | NM_014061 | Hs.279819 | chrxp11.22 |
| 218570_at | kelch repeat and BTB (POZ) domain containing 4 | KBTBD4 | NM_018095 | Hs.534536 | chr11p11.2 |
| 218567_x_at | dipeptidyl-peptidase 3 | DPP3 | NM_005700 | Hs.22880 | chr11q12-q13.1 |
| 218566_s_at | cysteine and histidine-rich domain (CHORD)-containing 1 | CHORDC1 | NM_012124 | Hs.22857 | chr11q14.3 |
| 218565_at | chromosome 9 open reading frame 114 | C9orf114 | BG223334 | Hs.224137 | chr9q34.11 |
| 218564_at | ring finger and WD repeat domain 3 | RFWD3 | BC002574 | Hs.77510 | chr16q22.3 |
| 218563_at | NADH dehydrogenase (ubiquinone) 1 alpha subcomplex, 3, 9kDa | NDUFA3 | NM_004542 | Hs.198269 | chr19q13.42 |
| 218561_s_at | LYR motif containing 4 | LYRM4 | NM_020408 | Hs.184860 | chr6p25.1 |
| 218559_s_at | v-maf musculoaponeurotic fibrosarcoma oncogene homolog B (avian) | MAFB | NM_005461 | Hs.169487 | chr20q11.2-q13.1 |
| 218558_s_at | mitochondrial ribosomal protein L39 | MRPL39 | NM_017446 | Hs.420696 | chr21q21.3 |
| 218557_at | nitrilase family, member 2 | NIT2 | NM_020202 | Hs.439152 | chr3q12.2 |
| 218556_at | ORM1-like 2 (S. cerevisiae) | ORMDL2 | NM_014182 | Hs.13144 | chr12q13.2 |
| 218553_s_at | potassium channel tetramerisation domain containing 15 | KCTD15 | NM_024076 | Hs.436348 | chr19q13.11 |
| 218552_at | enoyl Coenzyme A hydratase domain containing 2 | ECHDC2 | NM_018281 | Hs.170915 | chr1p32.3 |
| 218548_x_at | testis expressed sequence 264 | TEX264 | NM_015926 | Hs.279785 | chr3p21.31 |
| 218547_at | dehydrodolichyl diphosphate synthase | DHDDS | NM_024887 | Hs.369385 | chr1p36.11 |
| 218546_at | chromosome 1 open reading frame 115 | C1orf115 | NM_024709 | Hs.103395 | chr1q41 |
| 218545_at | coiled-coil domain containing 91 | CCDC91 | NM_018318 | Hs.406485 | chr12p11.22 |
| 218543_s_at | poly (ADP-ribose) polymerase family, member 12 | PARP12 | NM_022750 | Hs.12646 | chr7q34 |
| 218540_at | thiamine triphosphatase | THTPA | NM_024328 | Hs.15098 | chr14q11.2 |
| 218539_at | F-box protein 34 | FBXO34 | NM_017943 | Hs.15467 | chr14q22.3 |
| 218535_s_at | RIO kinase 2 (yeast) | RIOK2 | NM_018343 | Hs.27021 | chr5q15 |
| 218533_s_at | uridine-cytidine kinase 1-like 1 | UCKL1 | NM_017859 | Hs.504998 | chr20q13.33 |
| 218531_at | transmembrane protein 134 | TMEM134 | NM_025124 | Hs.288761 | chr11q13.2 |
| 218530_at | formin homology 2 domain containing 1 | FHOD1 | NM_013241 | Hs.95231 | chr16q22 |
| 218529_at | CD320 molecule | CD320 | NM_016579 | Hs.333427 | chr19p13.3-p13.2 |
| 218528_s_at | ring finger protein 38 | RNF38 | NM_022781 | Hs.333503 | chr9p13-p12 |
| 218526_s_at | RAN guanine nucleotide release factor | RANGNRF | NM_014185 | Hs.408233 | chr17p13 |
| 218524_at | E4F transcription factor 1 | E4F1 | NM_004424 | Hs.154196 | chr16p13.3 |
| 218522_s_at | microtubule-associated protein 1S | MAP1S | NM_018174 | Hs.66048 | chr19p13.11 |
| 218520_at | TANK-binding kinase 1 | TBK1 | NM_013254 | Hs.432466 | chr12q14.1 |
| 218519_at | solute carrier family 35, member A5 | SLC35A5 | NM_017945 | Hs.237480 | chr3q13.2 |
| 218518_at | chromosome 5 open reading frame 5 | C5orf5 | NM_016603 | Hs.483473 | chr5q31 |
| 218514_at | chromosome 17 open reading frame 71 | C17orf71 | NM_018149 | Hs.7296 | chr17q23.2 |
| 218513_at | hypothetical protein FLJ11184 | FLJ11184 | NM_018352 | Hs.267446 | chr4q32.2 |
| 218512_at | WD repeat domain 12 | WDR12 | NM_018256 | Hs.73291 | chr2q33.2 |
| 218510_x_at | hypothetical protein FLJ20152 | FLJ20152 | AI816291 | Hs.82273 | chr5p15.1 |
| 218508_at | DCP1 decapping enzyme homolog A (S. cerevisiae) | DCP1A | NM_018403 | Hs.370281 | chr3p21.1 |
| 218504_at | fumarylacetoacetate hydrolase domain containing 2A | FAHD2A | NM_016044 | Hs.12397 | chr2p24.3-p11.2 |
| 218503_at | KIAA1797 | KIAA1797 | NM_017794 | Hs.257696 | chr9p21 |
| 218501_at | Rho guanine nucleotide exchange factor (GEF) 3 | ARHGEF3 | NM_019555 | Hs.25951 | chr3p21-p13 |
| 218498_s_at | ERO1-like (S. cerevisiae) | ERO1L | NM_014584 | Hs.10949 | chr14q22.1 |
| 218497_s_at | ribonuclease H1 | RNASEH1 | NM_002936 | Hs.502765 | chr2p25 |
| 218495_at | ubiquitously-expressed transcript | UXT | NM_004182 | Hs.172791 | chrxp11.23-p11.22 |
| 218493_at | chromosome 16 open reading frame 33 | C16orf33 | NM_024571 | Hs.15277 | chr16p13.3 |
| 218492_s_at | THAP domain containing 7 | THAP7 | NM_030573 | Hs.512756 | chr22q11.2 |
| 218489_s_at | aminolevulinate, delta-, dehydratase | ALAD | NM_000031 | Hs.1227 | chr9q33.1 |
| 218486_at | Kruppel-like factor 11 | KLF11 | AA149594 | Hs.12229 | chr2p25 |
| 218483_s_at | chromosome 11 open reading frame 60 | C11orf60 | NM_020153 | Hs.334360 | chr11q23.3 |
| 218481_at | exosome component 5 | EXOSC5 | NM_020158 | Hs.283741 | chr19q13.1 |
| 218480_at | hypothetical protein FLJ21839 | FLJ21839 | NM_021831 | Hs.310960 | chr2p23.3 |
| 218478_s_at | zinc finger, CCHC domain containing 8 | ZCCHC8 | NM_017612 | Hs.37706 | chr12q24.31 |
| 218477_at | transmembrane protein 14A | TMEM14A | NM_014051 | Hs.94896 | chr6p12.3 |
| 218473_s_at | glycosyltransferase 25 domain containing 1 | GLT25D1 | NM_024656 | Hs.418795 | chr19p13.11 |
| 218471_s_at | Bardet-Biedl syndrome 1 | BBS1 | NM_024649 | Hs.129213 | chr11q13.1 |
| 218470_at | tyrosyl-tRNA synthetase 2 (mitochondrial) | YARS2 | NM_015936 | Hs.376743 | chr12p11.21 |
| 218467_at | tumor necrosis factor superfamily, member 5-induced protein 1 | TNFSF5IP1 | NM_020232 | Hs.386802 | chr18p11.21 |
| 218466_at | TBC1 domain family, member 17 | TBC1D17 | NM_024682 | Hs.325860 | chr19q13.33 |
| 218463_s_at | MUS81 endonuclease homolog (S. cerevisiae) | MUS81 | NM_025128 | Hs.288798 | chr11q13 |
| 218462_at | brix domain containing 5 | BXDC5 | NM_025065 | Hs.287863 | chr1p22.3 |
| 218461_at | ATP binding domain 1 family, member C | ATPBD1C | NM_016301 | Hs.525674 | chr12q24.11 |
| 218459_at | torsin family 3, member A | TOR3A | NM_022371 | Hs.523771 | chr1q25.2 |
| 218456_at | C1q domain containing 1 | C1QDC1 | NM_023925 | Hs.234355 | chr12p11 |
| 218455_at | NFS1 nitrogen fixation 1 homolog (S. cerevisiae) | NFS1 | NM_021100 | Hs.194692 | chr20q11.22 |
| 218452_at | SWI/SNF related, matrix associated, actin dependent regulator of chromatin, subfamily a-like 1 | SMARCAL1 | NM_014140 | Hs.435116 | chr2q34-q35 |
| 218450_at | heme binding protein 1 | HEBP1 | NM_015987 | Hs.294133 | chr12p13.1 |
| 218449_at | chromosome 4 open reading frame 20 | C4orf20 | NM_018359 | Hs.368022 | chr4q35.1 |
| 218447_at | chromosome 16 open reading frame 61 | C16orf61 | NM_020188 | Hs.6879 | chr16q23.2 |
| 218446_s_at | family with sequence similarity 18, member B | FAM18B | NM_016078 | Hs.87295 | chr17p11.2 |
| 218445_at | H2A histone family, member Y2 | H2AFY2 | NM_018649 | Hs.92023 | chr10q22 |
| 218443_s_at | DAZ associated protein 1 | DAZAP1 | NM_018959 | Hs.222510 | chr19p13.3 |
| 218441_s_at | RNA polymerase II associated protein 1 | RPAP1 | NM_015540 | Hs.4849 | chr15q15.1 |
| 218440_at | methylcrotonoyl-Coenzyme A carboxylase 1 (alpha) | MCCC1 | NM_020166 | Hs.47649 | chr3q27 |
| 218439_s_at | COMM domain containing 10 | COMMD10 | NM_016144 | Hs.151458 | chr5q23.1 |
| 218436_at | SIL1 homolog, endoplasmic reticulum chaperone (S. cerevisiae) | SIL1 | NM_022464 | Hs.297875 | chr5q31 |
| 218434_s_at | acetoacetyl-CoA synthetase | AACS | NM_023928 | Hs.169054 | chr12q24.31 |
| 218433_at | pantothenate kinase 3 | PANK3 | NM_024594 | Hs.388400 | chr5q34 |
| 218431_at | chromosome 14 open reading frame 133 | C14orf133 | NM_022067 | Hs.16157 | chr14q24.3-q31 |
| 218427_at | serologically defined colon cancer antigen 3 | SDCCAG3 | NM_006643 | Hs.94300 | chr9q34.3 |
| 218426_s_at | TRIAD3 protein | TRIAD3 | NM_019011 | Hs.336227 | chr7p22.1 |
| 218424_s_at | STEAP family member 3 | STEAP3 | NM_018234 | Hs.20805 | chr2q14.2 |
| 218423_x_at | vacuolar protein sorting 54 homolog (S. cerevisiae) | VPS54 | NM_016516 | Hs.48499 | chr2p13-p14 |
| 218421_at | ceramide kinase | CERK | NM_022766 | Hs.34516 | chr22q13.31 |
| 218420_s_at | chromosome 13 open reading frame 23 | C13orf23 | NM_025138 | Hs.318526 | chr13q13.3 |
| 218419_s_at | transmembrane and ubiquitin-like domain containing 2 | TMUB2 | NM_024107 | Hs.534564 | chr17q21.31 |
| 218418_s_at | ankyrin repeat domain 25 | ANKRD25 | NM_015493 | Hs.284208 | chr19p13.2 |
| 218414_s_at | nudE nuclear distribution gene E homolog 1 (A. nidulans) | NDE1 | NM_017668 | Hs.460107 | chr16p13.11 |
| 218412_s_at | GTF2I repeat domain containing 1 | GTF2IRD1 | NM_016328 | Hs.430854 | chr7q11.23 |
| 218411_s_at | MAP3K12 binding inhibitory protein 1 | MBIP | NM_016586 | Hs.368647 | chr14q13.3 |
| 218409_s_at | DnaJ (Hsp40) homolog, subfamily C, member 1 | DNAJC1 | NM_022365 | Hs.13015 | chr10p12.31 |
| 218408_at | translocase of inner mitochondrial membrane 10 homolog (yeast) | TIMM10 | NM_012456 | Hs.235750 | chr11q12.1-q12.3 |
| 218405_at | activator of basal transcription 1 | ABT1 | NM_013375 | Hs.254406 | chr6p22.1 |
| 218404_at | sorting nexin 10 | SNX10 | NM_013322 | Hs.418132 | chr7p15.2 |
| 218403_at | TP53 regulated inhibitor of apoptosis 1 | TRIAP1 | NM_016399 | Hs.69499 | chr12q24.31 |
| 218402_s_at | Hermansky-Pudlak syndrome 4 | HPS4 | NM_022081 | Hs.441481 | chr22cen-q12.3 |
| 218401_s_at | zinc finger protein 281 | ZNF281 | NM_012482 | Hs.59757 | chr1q32.1 |
| 218400_at | 2'-5'-oligoadenylate synthetase 3, 100kDa | OAS3 | NM_006187 | Hs.528634 | chr12q24.2 |
| 218398_at | mitochondrial ribosomal protein S30 | MRPS30 | NM_016640 | Hs.124165 | chr5q11 |
| 218397_at | Fanconi anemia, complementation group L | FANCL | NM_018062 | Hs.534525 | chr2p16.1 |
| 218396_at | vacuolar protein sorting 13 homolog C (S. cerevisiae) | VPS13C | NM_017684 | Hs.328109 | chr15q22.2 |
| 218395_at | ARP6 actin-related protein 6 homolog (yeast) | ACTR6 | NM_022496 | Hs.23259 | chr12q23.1 |
| 218394_at | rogdi homolog (Drosophila) | ROGDI | NM_024589 | Hs.22795 | chr16p13.3 |
| 218389_s_at | anterior pharynx defective 1 homolog A (C. elegans) | APH1A | NM_016022 | Hs.108408 | chr1p36.13-q31.3 |
| 218387_s_at | 6-phosphogluconolactonase | PGLS | NM_012088 | Hs.100071 | chr19p13.2 |
| 218386_x_at | ubiquitin specific peptidase 16 | USP16 | NM_006447 | Hs.99819 | chr21q22.11 |
| 218385_at | mitochondrial ribosomal protein S18A | MRPS18A | NM_018135 | Hs.9622 | chr6p21.3 |
| 218382_s_at | U2 small nuclear RNA auxiliary factor 2 | U2AF2 | NM_007279 | Hs.297629 | chr19q13.42 |
| 218380_at | NLR family, pyrin domain containing 1 | NLRP1 | NM_021730 | Hs.104305 | chr17p13 |
| 218378_s_at | PRKR interacting protein 1 (IL11 inducible) | PRKRIP1 | NM_024653 | Hs.406395 | chr7q22.1 |
| 218377_s_at | chromosome 21 open reading frame 6 | C21orf6 | NM_016940 | Hs.34136 | chr21q22.11 |
| 218376_s_at | microtubule associated monoxygenase, calponin and LIM domain containing 1 | MICAL1 | NM_022765 | Hs.33476 | chr6q21 |
| 218375_at | nudix (nucleoside diphosphate linked moiety X)-type motif 9 | NUDT9 | NM_024047 | Hs.301789 | chr4q22.1 |
| 218374_s_at | chromosome 12 open reading frame 4 | C12orf4 | NM_020374 | Hs.296198 | chr12p13.3 |
| 218372_at | mediator of RNA polymerase II transcription, subunit 9 homolog (S. cerevisiae) | MED9 | NM_018019 | Hs.235195 | chr17p11.2 |
| 218368_s_at | tumor necrosis factor receptor superfamily, member 12A | TNFRSF12A | NM_016639 | Hs.355899 | chr16p13.3 |
| 218365_s_at | aspartyl-tRNA synthetase 2 (mitochondrial) | DARS2 | AI765051 | Hs.59346 | chr1q25.1 |
| 218364_at | leucine rich repeat (in FLII) interacting protein 2 | LRRFIP2 | NM_017724 | Hs.268768 | chr3p22.3 |
| 218362_s_at | KIAA1008 | KIAA1008 | NM_014953 | Hs.323346 | chr13q22.1 |
| 218361_at | golgi phosphoprotein 3-like | GOLPH3L | NM_018178 | Hs.29379 | chr1q21.2 |
| 218358_at | Cyclin K | CCNK | NM_024324 |  |  |
| 218357_s_at | translocase of inner mitochondrial membrane 8 homolog B (yeast) | TIMM8B | NM_012459 | Hs.279915 | chr11q23.1-q23.2 |
| 218354_at | trafficking protein particle complex 2-like | TRAPPC2L | NM_016209 | Hs.265196 | chr16q24.3 |
| 218352_at | regulator of chromosome condensation (RCC1) and BTB (POZ) domain containing protein 1 | RCBTB1 | NM_018191 | Hs.508021 | chr13q14 |
| 218351_at | COMM domain containing 8 | COMMD8 | NM_017845 | Hs.23956 | chr4p12 |
| 218350_s_at | geminin, DNA replication inhibitor | GMNN | NM_015895 | Hs.234896 | chr6p22.2 |
| 218349_s_at | Zwilch, kinetochore associated, homolog (Drosophila) | ZWILCH | NM_017975 | Hs.21331 | chr15q22.31 |
| 218347_at | tRNA-yW synthesizing protein 1 homolog (S. cerevisiae) | TYW1 | NM_018264 | Hs.293336 | chr7q11.21 |
| 218346_s_at | sestrin 1 | SESN1 | NM_014454 | Hs.59554 | chr6q21 |
| 218344_s_at | REST corepressor 3 | RCOR3 | NM_018254 | Hs.125478 | chr1q32.3 |
| 218343_s_at | general transcription factor IIIC, polypeptide 3, 102kDa | GTF3C3 | NM_012086 | Hs.253724 | chr2q33.1 |
| 218341_at | phosphopantothenoylcysteine synthetase | PPCS | NM_024664 | Hs.244220 | chr1p34.2 |
| 218340_s_at | ubiquitin-activating enzyme E1-like 2 | UBE1L2 | NM_018227 | Hs.59838 | chr4q13.2 |
| 218339_at | mitochondrial ribosomal protein L22 | MRPL22 | NM_014180 | Hs.41007 | chr5q33.1-q33.3 |
| 218337_at | retinoic acid induced 16 | RAI16 | NM_022749 | Hs.105216 | chr8p21.3 |
| 218336_at | prefoldin subunit 2 | PFDN2 | NM_012394 | Hs.298229 | chr1q23.3 |
| 218334_at | THO complex 7 homolog (Drosophila) | THOC7 | NM_025075 | Hs.288151 | chr3p14.1 |
| 218333_at | Der1-like domain family, member 2 | DERL2 | NM_016041 | Hs.286131 | chr17p |
| 218332_at | brain expressed, X-linked 1 | BEX1 | NM_018476 | Hs.334370 | chrxq21-q23 |
| 218329_at | PR domain containing 4 | PRDM4 | NM_012406 | Hs.434866 | chr12q23-q24.1 |
| 218327_s_at | synaptosomal-associated protein, 29kDa | SNAP29 | NM_004782 | Hs.194714 | chr22q11.21 |
| 218326_s_at | leucine-rich repeat-containing G protein-coupled receptor 4 | LGR4 | NM_018490 | Hs.160271 | chr11p14-p13 |
| 218320_s_at | NADH dehydrogenase (ubiquinone) 1 beta subcomplex, 11, 17.3kDa | NDUFB11 | NM_019056 | Hs.433328 | chrxp11.3 |
| 218319_at | pellino homolog 1 (Drosophila) | PELI1 | NM_020651 | Hs.7886 | chr2p13.3 |
| 218316_at | translocase of inner mitochondrial membrane 9 homolog (yeast) | TIMM9 | NM_012460 | Hs.440525 | chr14q21 |
| 218315_s_at | CDK5 regulatory subunit associated protein 1 | CDK5RAP1 | NM_016408 | Hs.376227 | chr20pter-q11.23 |
| 218313_s_at | UDP-N-acetyl-alpha-D-galactosamine:polypeptide N-acetylgalactosaminyltransferase 7 (GalNAc-T7) | GALNT7 | NM_017423 | Hs.127407 | chr4q31.1 |
| 218312_s_at | zinc finger protein 447 | ZNF447 | NM_023926 | Hs.235390 | chr19q13.43 |
| 218311_at | mitogen-activated protein kinase kinase kinase kinase 3 | MAP4K3 | NM_003618 | Hs.532903 | chr2p22.1 |
| 218309_at | calcium/calmodulin-dependent protein kinase II inhibitor 1 | CAMK2N1 | NM_018584 | Hs.197922 | chr1p36.12 |
| 218307_at | radical S-adenosyl methionine domain containing 1 | RSAD1 | NM_018346 | Hs.8033 | chr17q21.33 |
| 218306_s_at | hect (homologous to the E6-AP (UBE3A) carboxyl terminus) domain and RCC1 (CHC1)-like domain (RLD) 1 | HERC1 | NM_003922 | Hs.133411 | chr15q22 |
| 218305_at | importin 4 | IPO4 | NM_024658 |  |  |
| 218303_x_at | lysine-rich coiled-coil 1 | KRCC1 | NM_016618 | Hs.5721 | chr2p11.2 |
| 218302_at | presenilin enhancer 2 homolog (C. elegans) | PSENEN | NM_018468 | Hs.283522 | chr19q13.12 |
| 218301_at | arginyl aminopeptidase (aminopeptidase B)-like 1 | RNPEPL1 | NM_018226 | Hs.5345 | chr2q37.3 |
| 218298_s_at | chromosome 14 open reading frame 159 | C14orf159 | NM_024952 | Hs.285673 | chr14q32.12 |
| 218295_s_at | nucleoporin 50kDa | NUP50 | NM_007172 | Hs.362841 | chr22q13.31 |
| 218291_at | mitogen-activated protein-binding protein-interacting protein | MAPBPIP | NM_014017 | Hs.351629 | chr1q22 |
| 218290_at | pleckstrin homology domain containing, family J member 1 | PLEKHJ1 | NM_018049 | Hs.512626 | chr19p13.3 |
| 218288_s_at | coiled-coil domain containing 90B | CCDC90B | NM_021825 | Hs.368866 | chr11q14.1 |
| 218286_s_at | ring finger protein 7 | RNF7 | NM_014245 | Hs.134623 | chr3q22-q24 |
| 218285_s_at | 3-hydroxybutyrate dehydrogenase, type 2 | BDH2 | NM_020139 | Hs.124696 | chr4q24 |
| 218283_at | synovial sarcoma translocation gene on chromosome 18-like 2 | SS18L2 | NM_016305 | Hs.9774 | chr3p21 |
| 218282_at | ER degradation enhancer, mannosidase alpha-like 2 | EDEM2 | NM_018217 | Hs.356273 | chr20q11.22 |
| 218281_at | mitochondrial ribosomal protein L48 | MRPL48 | NM_016055 | Hs.82389 | chr11q13.4 |
| 218273_s_at | protein phosphatase 2C, magnesium-dependent, catalytic subunit | PPM2C | NM_018444 | Hs.22265 | chr8q22.1 |
| 218271_s_at | presenilin associated, rhomboid-like | PARL | NM_018622 | Hs.13094 | chr3q27.1 |
| 218270_at | mitochondrial ribosomal protein L24 | MRPL24 | NM_024540 | Hs.418233 | chr1q21-q22 |
| 218269_at | ribonuclease III, nuclear | RNASEN | NM_013235 | Hs.369988 | chr5p13.3 |
| 218268_at | TBC1 domain family, member 15 | TBC1D15 | NM_022771 | Hs.310422 | chr12q21.1 |
| 218265_at | SECIS binding protein 2 | SECISBP2 | NM_024077 | Hs.288141 | chr9q22.2 |
| 218263_s_at | zinc finger, BED-type containing 5 | ZBED5 | NM_021211 | Hs.25726 | chr11p15.3 |
| 218262_at | required for meiotic nuclear division 5 homolog B (S. cerevisiae) | RMND5B | NM_022762 | Hs.22753 | chr5q35.3 |
| 218260_at | chromosome 19 open reading frame 58 | C19orf58 | NM_024050 | Hs.181551 | chr19p13.11 |
| 218259_at | MKL/myocardin-like 2 | MKL2 | NM_014048 | Hs.151076 | chr16p13.12 |
| 218258_at | polymerase (RNA) I polypeptide D, 16kDa | POLR1D | NM_015972 |  | chr13q12.2 |
| 218256_s_at | nucleoporin 54kDa | NUP54 | NM_017426 | Hs.9082 | chr4q21.1 |
| 218253_s_at | ligatin | LGTN | NM_006893 | Hs.274151 | chr1q31-q32 |
| 218252_at | cytoskeleton associated protein 2 | CKAP2 | NM_018204 | Hs.24641 | chr13q14 |
| 218251_at | MID1 interacting protein 1 (gastrulation specific G12 homolog (zebrafish)) | MID1IP1 | NM_021242 | Hs.236556 | chrxp11.4 |
| 218249_at | zinc finger, DHHC-type containing 6 | ZDHHC6 | NM_022494 | Hs.22353 | chr10q25.2 |
| 218248_at | family with sequence similarity 111, member A | FAM111A | NM_022074 | Hs.19525 | chr11q12.1 |
| 218247_s_at | ring finger and KH domain containing 2 | RKHD2 | NM_016626 | Hs.12830 | chr18q21.1 |
| 218245_at | tsukushin | TSKU | NM_015516 | Hs.8361 | chr11q13.5 |
| 218244_at | nucleolar protein 8 | NOL8 | NM_017948 | Hs.442199 | chr9q22.31 |
| 218243_at | RUN and FYVE domain containing 1 | RUFY1 | NM_025158 | Hs.306769 | chr5q35.3 |
| 218241_at | golgi autoantigen, golgin subfamily a, 5 | GOLGA5 | NM_005113 | Hs.241572 | chr14q32.12-q32.13 |
| 218238_at | GTP binding protein 4 | GTPBP4 | NM_012341 | Hs.215766 | chr10p15-p14 |
| 218235_s_at | UTP11-like, U3 small nucleolar ribonucleoprotein, (yeast) | UTP11L | NM_016037 | Hs.111449 | chr1p34.3 |
| 218233_s_at | chromosome 6 open reading frame 49 | C6orf49 | NM_017601 | Hs.525899 | chr6p21.31 |
| 218231_at | N-acetylglucosamine kinase /// N-acetylglucosamine kinase | NAGK | NM_017567 | Hs.7036 | chr2p13.3 |
| 218229_s_at | pogo transposable element with KRAB domain | POGK | NM_017542 | Hs.432752 | chr1q24.1 |
| 218227_at | nucleotide binding protein 2 (MinD homolog, E. coli) | NUBP2 | NM_012225 | Hs.256549 | chr16p13.3 |
| 218226_s_at | NADH dehydrogenase (ubiquinone) 1 beta subcomplex, 4, 15kDa /// similar to NADH dehydrogenase (ubiquinone) 1 beta subcomplex, 4, 15kDa | NDUFB4 | NM_004547 | Hs.227750 | chr3q13.33 |
| 218225_at | ECSIT homolog (Drosophila) | ECSIT | NM_016581 | Hs.22199 | chr19p13.2 |
| 218224_at | paraneoplastic antigen MA1 | PNMA1 | NM_006029 | Hs.194709 | chr14q24.3 |
| 218223_s_at | pleckstrin homology domain containing, family O member 1 | PLEKHO1 | NM_016274 | Hs.173380 | chr1q21.2 |
| 218220_at | chromosome 12 open reading frame 10 | C12orf10 | NM_021640 | Hs.400801 | chr12q13 |
| 218219_s_at | LanC lantibiotic synthetase component C-like 2 (bacterial) | LANCL2 | NM_018697 | Hs.224282 | chr7q31.1-q31.33 |
| 218218_at | DIP13 beta | DIP13B | NM_018171 | Hs.370019 | chr12q24.1 |
| 218217_at | serine carboxypeptidase 1 | SCPEP1 | NM_021626 | Hs.431107 | chr17q23.2 |
| 218216_x_at | ADP-ribosylation-like factor 6 interacting protein 4 | ARL6IP4 | NM_016638 | Hs.103561 | chr12q24.31 |
| 218215_s_at | nuclear receptor subfamily 1, group H, member 2 | NR1H2 | NM_007121 | Hs.432976 | chr19q13.3-19q13.3 |
| 218214_at | chromosome 12 open reading frame 44 | C12orf44 | NM_021934 | Hs.9911 | chr12q13.13 |
| 218213_s_at | chromosome 11 open reading frame 10 | C11orf10 | NM_014206 | Hs.437779 | chr11q12-q13.1 |
| 218212_s_at | molybdenum cofactor synthesis 2 | MOCS2 | NM_004531 | Hs.163645 | chr5q11 |
| 218211_s_at | melanophilin | MLPH | NM_024101 | Hs.297405 | chr2q37.3 |
| 218210_at | fructosamine-3-kinase-related protein | FN3KRP | NM_024619 | Hs.31431 | chr17q25.3 |
| 218208_at | PQ loop repeat containing 1 | PQLC1 | NM_025078 | Hs.288284 | chr18q23 |
| 218204_s_at | FYVE and coiled-coil domain containing 1 | FYCO1 | NM_024513 | Hs.257267 | chr3p21.31 |
| 218203_at | asparagine-linked glycosylation 5 homolog (S. cerevisiae, dolichyl-phosphate beta-glucosyltransferase) | ALG5 | NM_013338 | Hs.339428 | chr13q13.3 |
| 218202_x_at | mitochondrial ribosomal protein L44 | MRPL44 | NM_022915 | Hs.203559 | chr2q36.1 |
| 218199_s_at | nucleolar protein family 6 (RNA-associated) | NOL6 | NM_022917 | Hs.183253 | chr9p13.3 |
| 218198_at | DEAH (Asp-Glu-Ala-His) box polypeptide 32 | DHX32 | NM_018180 | Hs.171835 | chr10q26.2 |
| 218195_at | chromosome 6 open reading frame 211 | C6orf211 | NM_024573 | Hs.15929 | chr6q25.1 |
| 218193_s_at | golgi transport 1 homolog B (S. cerevisiae) | GOLT1B | NM_016072 | Hs.62275 | chr12p12.1 |
| 218192_at | inositol hexaphosphate kinase 2 | IHPK2 | NM_016291 | Hs.323432 | chr3p21.31 |
| 218191_s_at | LMBR1 domain containing 1 | LMBRD1 | NM_018368 | Hs.439494 | chr6q13 |
| 218189_s_at | N-acetylneuraminic acid synthase (sialic acid synthase) | NANS | NM_018946 | Hs.274424 | chr9p24.1-p23 |
| 218188_s_at | translocase of inner mitochondrial membrane 13 homolog (yeast) | TIMM13 | NM_012458 | Hs.75056 | chr19p13.3 |
| 218185_s_at | armadillo repeat containing 1 | ARMC1 | NM_018120 | Hs.106768 | chr8q13.1 |
| 218183_at | chromosome 16 open reading frame 5 | C16orf5 | NM_013399 | Hs.7765 | chr16p13.3 |
| 218179_s_at | FLJ12716 protein | FLJ12716 | NM_021942 | Hs.443240 | chr4q35.1 |
| 218177_at | chromatin modifying protein 1B | CHMP1B | AA293502 | Hs.136295 | chr18p11.21 |
| 218176_at | melanoma antigen family F, 1 | MAGEF1 | NM_022149 | Hs.306123 | chr3q13 |
| 218175_at | coiled-coil domain containing 92 | CCDC92 | NM_025140 | Hs.387266 | chr12q24.31 |
| 218174_s_at | chromosome 10 open reading frame 57 | C10orf57 | NM_025125 | Hs.288768 | chr10q22.3 |
| 218172_s_at | Der1-like domain family, member 1 | DERL1 | NM_018630 | Hs.241576 | chr8q24.13 |
| 218171_at | vacuolar protein sorting 4 homolog B (S. cerevisiae) | VPS4B | AF195514 | Hs.126550 | chr18q21.32-q21.33 |
| 218170_at | isochorismatase domain containing 1 | ISOC1 | NM_016048 | Hs.11085 | chr5q22.1-q33.3 |
| 218164_at | spermatogenesis associated 20 | SPATA20 | NM_022827 | Hs.103147 | chr17q21.33 |
| 218163_at | malignant T cell amplified sequence 1 | MCTS1 | NM_014060 | Hs.102696 | chrxq22-q24 |
| 218160_at | NADH dehydrogenase (ubiquinone) 1 alpha subcomplex, 8, 19kDa | NDUFA8 | NM_014222 | Hs.31547 | chr9q33.2-q34.11 |
| 218159_at | chromosome 20 open reading frame 116 | C20orf116 | NM_023935 | Hs.28393 | chr20p13 |
| 218154_at | gasdermin domain containing 1 | GSDMDC1 | NM_024736 | Hs.118983 | chr8q24.3 |
| 218153_at | hypothetical protein FLJ12118 | FLJ12118 | NM_024537 | Hs.381043 | chr13q34 |
| 218152_at | high-mobility group 20A | HMG20A | NM_018200 | Hs.69594 | chr15q24 |
| 218150_at | ADP-ribosylation factor-like 5A | ARL5A | NM_012097 | Hs.342849 | chr2q23.3 |
| 218146_at | glycosyltransferase 8 domain containing 1 | GLT8D1 | NM_018446 | Hs.297304 | chr3p21.1 |
| 218145_at | tribbles homolog 3 (Drosophila) | TRIB3 | NM_021158 | Hs.344378 | chr20p13-p12.2 |
| 218138_at | McKusick-Kaufman syndrome | MKKS | NM_018848 | Hs.46743 | chr20p12 |
| 218137_s_at | stromal membrane-associated protein 1 | SMAP1 | NM_021940 | Hs.4273 | chr6q12-q13 |
| 218133_s_at | NIF3 NGG1 interacting factor 3-like 1 (S. pombe) | NIF3L1 | NM_021824 | Hs.21943 | chr2q33 |
| 218132_s_at | tRNA splicing endonuclease 34 homolog (S. cerevisiae) | TSEN34 | NM_024075 | Hs.15580 | chr19q13.4 |
| 218130_at | chromosome 17 open reading frame 62 | C17orf62 | NM_024510 | Hs.163113 | chr17q25.3 |
| 218126_at | family with sequence similarity 82, member C | FAM82C | NM_018145 | Hs.23025 | chr15q15.1 |
| 218123_at | chromosome 21 open reading frame 59 | C21orf59 | NM_017835 | Hs.5811 | chr21q22.1 |
| 218122_s_at | SUMO1/sentrin/SMT3 specific peptidase 2 | SENP2 | NM_021627 | Hs.401388 | chr3q27.2 |
| 218120_s_at | heme oxygenase (decycling) 2 | HMOX2 | D21243 | Hs.284279 | chr16p13.3 |
| 218118_s_at | translocase of inner mitochondrial membrane 23 homolog (yeast) | TIMM23 | NM_006327 | Hs.11866 | chr10q11.21-q11.23 |
| 218117_at | ring-box 1 | RBX1 | NM_014248 | Hs.373992 | chr22q13.2 |
| 218116_at | chromosome 9 open reading frame 78 /// chromosome 9 open reading frame 78 | C9orf78 | NM_016520 | Hs.278429 | chr9q34.11 |
| 218113_at | transmembrane protein 2 | TMEM2 | NM_013390 | Hs.160417 | chr9q13-q21 |
| 218112_at | mitochondrial ribosomal protein S34 | MRPS34 | NM_023936 | Hs.157160 | chr16p13.3 |
| 218110_at | XPA binding protein 2 | XAB2 | NM_020196 | Hs.9822 | chr19p13.2 |
| 218109_s_at | major facilitator superfamily domain containing 1 | MFSD1 | NM_022736 | Hs.7503 | chr3q25.32 |
| 218108_at | chromosome 14 open reading frame 130 | C14orf130 | NM_018108 | Hs.6877 | chr14q32.13 |
| 218106_s_at | mitochondrial ribosomal protein S10 | MRPS10 | NM_018141 | Hs.380887 | chr6p21.1-p12.1 |
| 218104_at | testis expressed sequence 10 | TEX10 | NM_017746 | Hs.180236 | chr9q31.1 |
| 218103_at | FtsJ homolog 3 (E. coli) | FTSJ3 | NM_017647 | Hs.257486 | chr17q23 |
| 218102_at | 2-deoxyribose-5-phosphate aldolase homolog (C. elegans) | DERA | NM_015954 | Hs.39429 | chr12p12.3 |
| 218100_s_at | intraflagellar transport 57 homolog (Chlamydomonas) | IFT57 | NM_018010 | Hs.170318 | chr3q13.12-q13.13 |
| 218099_at | testis expressed sequence 2 | TEX2 | NM_018469 | Hs.16206 | chr17q23.3 |
| 218097_s_at | CUE domain containing 2 | CUEDC2 | NM_024040 | Hs.11270 | chr10q24.32 |
| 218094_s_at | dysbindin (dystrobrevin binding protein 1) domain containing 2 /// C20orf169-DBNDD2 readthrough transcript | DBNDD2 | NM_018478 | Hs.130463 | chr20q13.12 |
| 218090_s_at | bromodomain and WD repeat domain containing 2 | BRWD2 | NM_018117 | Hs.16677 | chr10q26 |
| 218089_at | chromosome 20 open reading frame 4 | C20orf4 | NM_015511 | Hs.11314 | chr20pter-q12 |
| 218088_s_at | Ras-related GTP binding C | RRAGC | NM_022157 | Hs.110950 | chr1p34 |
| 218087_s_at | sorbin and SH3 domain containing 1 | SORBS1 | NM_015385 | Hs.153244 | chr10q23.3-q24.1 |
| 218085_at | chromatin modifying protein 5 | CHMP5 | NM_015961 | Hs.415534 | chr9p13.3 |
| 218083_at | prostaglandin E synthase 2 | PTGES2 | NM_025072 | Hs.288102 | chr9q34.11 |
| 218082_s_at | upstream binding protein 1 (LBP-1a) | UBP1 | NM_014517 | Hs.28423 | chr3p23 |
| 218080_x_at | Fas (TNFRSF6) associated factor 1 | FAF1 | NM_007051 | Hs.12899 | chr1p33 |
| 218079_s_at | zinc finger protein 403 | ZNF403 | NM_024835 | Hs.149055 | chr17q12 |
| 218077_s_at | zinc finger, DHHC-type containing 3 | ZDHHC3 | BE542551 | Hs.61430 | chr3p21.31 |
| 218076_s_at | Rho GTPase activating protein 17 | ARHGAP17 | NM_018054 | Hs.203605 | chr16p12.1 |
| 218074_at | family with sequence similarity 96, member B | FAM96B | NM_016062 | Hs.9825 | chr16q22.1-q22.3 |
| 218073_s_at | transmembrane protein 48 | TMEM48 | NM_018087 | Hs.435982 | chr1p32.3 |
| 218072_at | COMM domain containing 9 | COMMD9 | NM_014186 | Hs.279836 | chr11p13 |
| 218070_s_at | GDP-mannose pyrophosphorylase A | GMPPA | NM_013335 | Hs.27059 | chr2q35 |
| 218069_at | XTP3-transactivated protein A | XTP3TPA | NM_024096 | Hs.237971 | chr16p11.2 |
| 218068_s_at | zinc finger protein 672 | ZNF672 | NM_024836 | Hs.181406 | chr1q44 |
| 218066_at | solute carrier family 12 (potassium/chloride transporters), member 7 | SLC12A7 | NM_006598 | Hs.172613 | chr5p15 |
| 218064_s_at | A kinase (PRKA) anchor protein 8-like | AKAP8L | NM_014371 | Hs.96200 | chr19p13.12 |
| 218062_x_at | CDC42 effector protein (Rho GTPase binding) 4 | CDC42EP4 | NM_012121 | Hs.3903 | chr17q24-q25 |
| 218061_at | male-enhanced antigen 1 | MEA1 | NM_014623 | Hs.278362 | chr6p21.3-p21.1 |
| 218056_at | bifunctional apoptosis regulator | BFAR | NM_016561 | Hs.435556 | chr16p13.12 |
| 218055_s_at | WD repeat domain 41 | WDR41 | NM_018268 | Hs.16470 | chr5q13.3 |
| 218052_s_at | ATPase type 13A1 | ATP13A1 | NM_020410 | Hs.435966 | chr19p13.11 |
| 218051_s_at | 5'-nucleotidase domain containing 2 | NT5DC2 | NM_022908 | Hs.84753 | chr3p21.1 |
| 218049_s_at | mitochondrial ribosomal protein L13 | MRPL13 | NM_014078 | Hs.333823 | chr8q22.1-q22.3 |
| 218048_at | COMM domain containing 3 | COMMD3 | NM_012071 | Hs.534446 | chr10pter-q22.1 |
| 218045_x_at | parathymosin | PTMS | NM_002824 | Hs.446525 | chr12p13 |
| 218042_at | COP9 constitutive photomorphogenic homolog subunit 4 (Arabidopsis) | COPS4 | NM_016129 | Hs.6671 | chr4q21.3 |
| 218041_x_at | solute carrier family 38, member 2 | SLC38A2 | NM_018573 | Hs.298275 | chr12q |
| 218039_at | nucleolar and spindle associated protein 1 | NUSAP1 | NM_016359 | Hs.279905 | chr15q15.1 |
| 218036_x_at | NMD3 homolog (S. cerevisiae) | NMD3 | NM_015938 | Hs.492805 | chr3q26.1 |
| 218035_s_at | RNA-binding protein | FLJ20273 | NM_019027 | Hs.95549 | chr4p13-p12 |
| 218034_at | fission 1 (mitochondrial outer membrane) homolog (S. cerevisiae) | FIS1 | NM_016068 | Hs.423968 | chr7q22.1 |
| 218032_at | stannin | SNN | AF070673 | Hs.76691 | chr16p13 |
| 218028_at | elongation of very long chain fatty acids (FEN1/Elo2, SUR4/Elo3, yeast)-like 1 | ELOVL1 | NM_016031 | Hs.25597 | chr1p34.2 |
| 218027_at | mitochondrial ribosomal protein L15 | MRPL15 | NM_014175 | Hs.18349 | chr8q11.2-q13 |
| 218026_at | coiled-coil domain containing 56 | CCDC56 | NM_014019 | Hs.16059 | chr17q21 |
| 218025_s_at | peroxisomal D3,D2-enoyl-CoA isomerase | PECI | NM_006117 | Hs.15250 | chr6p24.3 |
| 218024_at | brain protein 44-like | BRP44L | NM_016098 | Hs.117963 | chr6q27 |
| 218023_s_at | family with sequence similarity 53, member C | FAM53C | NM_016605 | Hs.54056 | chr5q31 |
| 218022_at | vaccinia related kinase 3 | VRK3 | NM_016440 | Hs.443330 | chr19q13 |
| 218021_at | dehydrogenase/reductase (SDR family) member 4 | DHRS4 | NM_021004 | Hs.382045 | chr14q11.2 |
| 218014_at | nucleoporin 85kDa | NUP85 | NM_024844 | Hs.184352 | chr17q25.1 |
| 218011_at | ubiquitin-like 5 | UBL5 | NM_024292 |  |  |
| 218010_x_at | chromosome 20 open reading frame 149 | C20orf149 | NM_024299 | Hs.79625 | chr20q13.33 |
| 218009_s_at | protein regulator of cytokinesis 1 | PRC1 | NM_003981 | Hs.344037 | chr15q26.1 |
| 218005_at | zinc finger protein 22 (KOX 15) | ZNF22 | AA744771 | Hs.108642 | chr10q11 |
| 218003_s_at | FK506 binding protein 3, 25kDa | FKBP3 | NM_002013 | Hs.379557 | chr14q21.3 |
| 218001_at | mitochondrial ribosomal protein S2 | MRPS2 | NM_016034 | Hs.382044 | chr9q34 |
| 217996_at | pleckstrin homology-like domain, family A, member 1 | PHLDA1 | AA576961 | Hs.534436 | chr12q15 |
| 217993_s_at | methionine adenosyltransferase II, beta | MAT2B | NM_013283 | Hs.54642 | chr5q34-q35.1 |
| 217990_at | guanosine monophosphate reductase 2 | GMPR2 | NM_016576 | Hs.368855 | chr14q11.2 |
| 217989_at | hydroxysteroid (17-beta) dehydrogenase 11 | HSD17B11 | NM_016245 | Hs.282984 | chr4q22.1 |
| 217988_at | cyclin B1 interacting protein 1 | CCNB1IP1 | NM_021178 | Hs.107003 | chr14q11.2 |
| 217987_at | asparagine synthetase domain containing 1 | ASNSD1 | NM_019048 | Hs.101364 | chr2p24.3-q21.3 |
| 217985_s_at | bromodomain adjacent to zinc finger domain, 1A | BAZ1A | AA102574 | Hs.436488 | chr14q12-q13 |
| 217983_s_at | ribonuclease T2 | RNASET2 | NM_003730 | Hs.388130 | chr6q27 |
| 217982_s_at | mortality factor 4 like 1 | MORF4L1 | NM_006791 | Hs.374503 | chr15q24 |
| 217981_s_at | fracture callus 1 homolog (rat) | FXC1 | NM_012192 | Hs.54943 | chr11p15.5-p15.3 |
| 217980_s_at | mitochondrial ribosomal protein L16 | MRPL16 | NM_017840 | Hs.291898 | chr11q12-q13.1 |
| 217979_at | Tetraspanin 13 | TSPAN13 | NM_014399 | Hs.364544 | chr7p21.1 |
| 217978_s_at | ubiquitin-conjugating enzyme E2Q (putative) 1 | UBE2Q1 | NM_017582 | Hs.244003 | chr1q21.3 |
| 217977_at | selenoprotein X, 1 | SEPX1 | NM_016332 | Hs.279623 | chr16p13.3 |
| 217975_at | WW domain binding protein 5 | WBP5 | NM_016303 | Hs.15984 | chrxq22.2 |
| 217973_at | dicarbonyl/L-xylulose reductase | DCXR | NM_016286 | Hs.9857 | chr17q25.3 |
| 217972_at | coiled-coil-helix-coiled-coil-helix domain containing 3 | CHCHD3 | NM_017812 | Hs.434077 | chr7q32.3-q33 |
| 217971_at | mitogen-activated protein kinase kinase 1 interacting protein 1 | MAP2K1IP1 | NM_021970 | Hs.433332 | chr4q23 |
| 217970_s_at | CCR4-NOT transcription complex, subunit 6 | CNOT6 | NM_015455 | Hs.437844 | chr5q35.3 |
| 217969_at | chromosome 11 open reading frame2 | C11orf2 | NM_013265 | Hs.277517 | chr11q13 |
| 217968_at | tumor suppressing subtransferable candidate 1 | TSSC1 | NM_003310 | Hs.4992 | chr2p25.2 |
| 217966_s_at | family with sequence similarity 129, member A | FAM129A | NM_022083 | Hs.48778 | chr1q25 |
| 217964_at | tetratricopeptide repeat domain 19 | TTC19 | NM_017775 | Hs.171044 | chr17p12 |
| 217963_s_at | nerve growth factor receptor (TNFRSF16) associated protein 1 | NGFRAP1 | NM_014380 | Hs.448588 | chrxq22.2 |
| 217962_at | nucleolar protein family A, member 3 (H/ACA small nucleolar RNPs) | NOLA3 | NM_018648 | Hs.14317 | chr15q14-q15 |
| 217961_at | solute carrier family 25, member 38 | SLC25A38 | NM_017875 | Hs.7994 | chr3p22.2 |
| 217958_at | trafficking protein particle complex 4 | TRAPPC4 | NM_016146 | Hs.279901 | chr11q23.3 |
| 217957_at | chromosome 16 open reading frame 80 | C16orf80 | NM_013242 | Hs.279818 | chr16q21 |
| 217956_s_at | E-1 enzyme | MASA | NM_021204 | Hs.18442 | chr4q21.3 |
| 217950_at | nitric oxide synthase interacting protein | NOSIP | NM_015953 | Hs.7236 | chr19q13.33 |
| 217949_s_at | vitamin K epoxide reductase complex, subunit 1 | VKORC1 | NM_024006 | Hs.324844 | chr16p11.2 |
| 217947_at | CKLF-like MARVEL transmembrane domain containing 6 | CMTM6 | NM_017801 | Hs.380627 | chr3p23 |
| 217945_at | BTB (POZ) domain containing 1 | BTBD1 | NM_025238 | Hs.321501 | chr15q24 |
| 217943_s_at | arginine/proline rich coiled-coil 1 | RPRC1 | NM_018067 | Hs.356096 | chr1p34.3 |
| 217942_at | mitochondrial ribosomal protein S35 | MRPS35 | NM_021821 | Hs.10724 | chr12p11 |
| 217941_s_at | erbb2 interacting protein | ERBB2IP | NM_018695 | Hs.8117 | chr5q12.3 |
| 217940_s_at | hypothetical protein FLJ10769 | FLJ10769 | NM_018210 | Hs.8083 | chr13q34 |
| 217939_s_at | aftiphilin | AFTPH | NM_017657 | Hs.213003 | chr2p14 |
| 217937_s_at | histone deacetylase 7A | HDAC7A | NM_016596 | Hs.200063 | chr12q13.1 |
| 217934_x_at | STIP1 homology and U-box containing protein 1 | STUB1 | NM_005861 | Hs.414390 | chr16p13.3 |
| 217933_s_at | leucine aminopeptidase 3 | LAP3 | NM_015907 | Hs.182579 | chr4p15.32 |
| 217932_at | mitochondrial ribosomal protein S7 | MRPS7 | NM_015971 | Hs.325631 | chr17q25 |
| 217931_at | trinucleotide repeat containing 5 | TNRC5 | BC004423 | Hs.414099 | chr6pter-p12.1 |
| 217929_s_at | KIAA0319-like | KIAA0319L | NM_024874 | Hs.456507 | chr1p34.2 |
| 217927_at | signal peptidase complex subunit 1 homolog (S. cerevisiae) | SPCS1 | NM_014041 | Hs.11125 | chr3p21.1 |
| 217926_at | chromosome 19 open reading frame 53 | C19orf53 | NM_014047 | Hs.231616 | chr19p13.13 |
| 217923_at | penta-EF-hand domain containing 1 | PEF1 | NM_012392 | Hs.241531 | chr1p34 |
| 217917_s_at | dynein, light chain, roadblock-type 1 | DYNLRB1 | NM_014183 | Hs.100002 | chr20q11.21 |
| 217916_s_at | family with sequence similarity 49, member B | FAM49B | NM_016623 | Hs.32501 | chr8q24.21 |
| 217915_s_at | chromosome 15 open reading frame 15 | C15orf15 | NM_016304 | Hs.274772 | chr15q21 |
| 217913_at | vacuolar protein sorting 4 homolog A (S. cerevisiae) | VPS4A | NM_013245 | Hs.234839 | chr16q22.1 |
| 217912_at | dihydrouridine synthase 1-like (S. cerevisiae) | DUS1L | NM_022156 | Hs.351484 | chr17q25.3 |
| 217911_s_at | BCL2-associated athanogene 3 | BAG3 | NM_004281 | Hs.15259 | chr10q25.2-q26.2 |
| 217909_s_at | MAX-like protein X | MLX | BF056105 | Hs.383019 | chr17q21.1 |
| 217908_s_at | IQ motif and WD repeats 1 | IQWD1 | NM_018442 | Hs.280168 | chr1q24.2 |
| 217907_at | mitochondrial ribosomal protein L18 | MRPL18 | NM_014161 | Hs.416998 | chr6q25.3 |
| 217906_at | kelch domain containing 2 | KLHDC2 | NM_014315 | Hs.415236 | chr14q21.3 |
| 217905_at | chromosome 10 open reading frame 119 | C10orf119 | NM_024834 | Hs.180638 | chr10q26.12 |
| 217904_s_at | beta-site APP-cleaving enzyme 1 | BACE1 | NM_012104 | Hs.49349 | chr11q23.2-q23.3 |
| 217903_at | striatin, calmodulin binding protein 4 | STRN4 | NM_013403 | Hs.406918 | chr19q13.2 |
| 217902_s_at | hect domain and RLD 2 | HERC2 | NM_004667 | Hs.434890 | chr15q13 |
| 217900_at | isoleucine-tRNA synthetase 2, mitochondrial | IARS2 | NM_018060 | Hs.262823 | chr1q41 |
| 217899_at | hypothetical protein FLJ20254 | FLJ20254 | NM_017727 | Hs.15356 | chr2p23.3 |
| 217898_at | chromosome 15 open reading frame 24 | C15orf24 | NM_020154 | Hs.4245 | chr15q14 |
| 217897_at | FXYD domain containing ion transport regulator 6 | FXYD6 | NM_022003 | Hs.410748 | chr11q23.3 |
| 217896_s_at | NEFA-interacting nuclear protein NIP30 | NIP30 | NM_024946 | Hs.285017 | chr16q13 |
| 217894_at | potassium channel tetramerisation domain containing 3 | KCTD3 | NM_016121 | Hs.239155 | chr1q41 |
| 217888_s_at | ADP-ribosylation factor GTPase activating protein 1 | ARFGAP1 | NM_018209 | Hs.25584 | chr20q13.33 |
| 217886_at | epidermal growth factor receptor pathway substrate 15 | EPS15 | BF213575 | Hs.79095 | chr1p32 |
| 217885_at | importin 9 | IPO9 | NM_018085 | Hs.445587 | chr1q32.1 |
| 217884_at | N-acetyltransferase 10 | NAT10 | NM_024662 | Hs.438995 | chr11p13 |
| 217883_at | chromosome 2 open reading frame 25 | C2orf25 | NM_015702 | Hs.5324 | chr2q23.3 |
| 217882_at | transmembrane protein 111 | TMEM111 | NM_018447 | Hs.444846 | chr3p25.3 |
| 217878_s_at | cell division cycle 27 homolog (S. cerevisiae) | CDC27 | AI203880 | Hs.406631 | chr17q12-17q23.2 |
| 217874_at | succinate-CoA ligase, GDP-forming, alpha subunit | SUCLG1 | NM_003849 | Hs.270428 | chr2p11.2 |
| 217872_at | NOP17 | NOP17 | NM_017916 | Hs.5245 | chr19q13.33 |
| 217871_s_at | macrophage migration inhibitory factor (glycosylation-inhibiting factor) | MIF | NM_002415 | Hs.407995 | chr22q11.23 |
| 217870_s_at | cytidylate kinase | CMPK | NM_016308 | Hs.11463 | chr1p34.1-p33 |
| 217869_at | hydroxysteroid (17-beta) dehydrogenase 12 | HSD17B12 | NM_016142 | Hs.132513 | chr11p11.2 |
| 217867_x_at | beta-site APP-cleaving enzyme 2 | BACE2 | NM_012105 | Hs.436490 | chr21q22.3 |
| 217866_at | pre-mRNA cleavage factor I, 59 kDa subunit | FLJ12529 | NM_024811 | Hs.444552 | chr11q12.2 |
| 217865_at | ring finger protein 130 | RNF130 | NM_018434 | Hs.155718 | chr5q35.3 |
| 217863_at | protein inhibitor of activated STAT, 1 | PIAS1 | AI348378 | Hs.75251 | chr15q |
| 217861_s_at | prolactin regulatory element binding | PREB | NM_013388 | Hs.279784 | chr2p23.3 |
| 217860_at | NADH dehydrogenase (ubiquinone) 1 alpha subcomplex, 10, 42kDa /// similar to NADH dehydrogenase (ubiquinone) 1 alpha subcomplex, 10, 42kDa precursor | NDUFA10 | NM_004544 | Hs.198271 | chr2q37.3 |
| 217859_s_at | solute carrier family 39 (zinc transporter), member 9 | SLC39A9 | NM_018375 | Hs.432690 | chr14q24.1 |
| 217858_s_at | armadillo repeat containing, X-linked 3 | ARMCX3 | NM_016607 | Hs.172788 | chrxq21.33-q22.2 |
| 217857_s_at | RNA binding motif protein 8A | RBM8A | NM_005105 | Hs.10283 | chr1q12 |
| 217855_x_at | stromal cell derived factor 4 | SDF4 | NM_016547 | Hs.42806 | chr1p36.33 |
| 217852_s_at | ADP-ribosylation factor-like 8B | ARL8B | NM_018184 | Hs.277255 | chr3p26.1 |
| 217851_s_at | chromosome 20 open reading frame 45 | C20orf45 | NM_016045 | Hs.3945 | chr20q13.32 |
| 217850_at | guanine nucleotide binding protein-like 3 (nucleolar) | GNL3 | NM_014366 | Hs.313544 | chr3p21.1 |
| 217849_s_at | CDC42 binding protein kinase beta (DMPK-like) | CDC42BPB | NM_006035 | Hs.510533 | chr14q32.3 |
| 217848_s_at | pyrophosphatase (inorganic) 1 | PPA1 | NM_021129 | Hs.380830 | chr10q11.1-q24 |
| 217846_at | glutaminyl-tRNA synthetase | QARS | NM_005051 | Hs.79322 | chr3p21.3-p21.1 |
| 217845_x_at | HIG1 domain family, member 1A | HIGD1A | NM_014056 | Hs.7917 | chr3p22.1 |
| 217844_at | CTD (carboxy-terminal domain, RNA polymerase II, polypeptide A) small phosphatase 1 | CTDSP1 | NM_021198 | Hs.444468 | chr2q35 |
| 217842_at | LUC7-like 2 (S. cerevisiae) | LUC7L2 | NM_016019 | Hs.308777 | chr7q34 |
| 217841_s_at | protein phosphatase methylesterase 1 | PPME1 | NM_016147 | Hs.63304 | chr11q13.4 |
| 217840_at | DEAD (Asp-Glu-Ala-Asp) box polypeptide 41 | DDX41 | NM_016222 | Hs.274317 | chr5q35.3 |
| 217839_at | TRK-fused gene | TFG | NM_006070 | Hs.446568 | chr3q11-q12 |
| 217838_s_at | Enah/Vasp-like | EVL | NM_016337 | Hs.241471 | chr14q32.2 |
| 217837_s_at | vacuolar protein sorting 24 homolog (S. cerevisiae) | VPS24 | NM_016079 | Hs.512608 | chr2p24.3-p24.1 |
| 217835_x_at | chromosome 20 open reading frame 24 | C20orf24 | NM_018840 | Hs.184062 | chr20q11.23 |
| 217831_s_at | NSFL1 (p97) cofactor (p47) | NSFL1C | NM_016143 | Hs.12865 | chr20p13 |
| 217828_at | SAFB-like, transcription modulator | SLTM | NM_024755 | Hs.190114 | chr15q21.3 |
| 217824_at | ubiquitin-conjugating enzyme E2, J1 (UBC6 homolog, yeast) | UBE2J1 | AW500009 | Hs.184325 | chr6q15 |
| 217821_s_at | WW domain binding protein 11 | WBP11 | AF118023 | Hs.164526 | chr12p12.3 |
| 217818_s_at | actin related protein 2/3 complex, subunit 4, 20kDa | ARPC4 | NM_005718 | Hs.323342 | chr3p25.3 |
| 217815_at | suppressor of Ty 16 homolog (S. cerevisiae) | SUPT16H | NM_007192 | Hs.271225 | chr14q11.2 |
| 217814_at | coiled-coil domain containing 47 | CCDC47 | NM_020198 | Hs.8207 | chr17q23.3 |
| 217813_s_at | spindlin | SPIN | NM_006717 | Hs.439052 | chr9q22.1-q22.3 |
| 217812_at | YTH domain family, member 2 | YTHDF2 | NM_016258 | Hs.20993 | chr1p35 |
| 217811_at | selenoprotein T | SELT | NM_016275 | Hs.8148 | chr3q25.1 |
| 217809_at | basic leucine zipper and W2 domains 2 | BZW2 | NM_014038 | Hs.5216 | chr7p21.1 |
| 217797_at | ubiquitin-fold modifier conjugating enzyme 1 | UFC1 | NM_016406 | Hs.301412 | chr1q23.3 |
| 217796_s_at | nuclear protein localization 4 homolog (S. cerevisiae) | NPLOC4 | NM_017921 | Hs.353191 | chr17qter |
| 217794_at | proline rich 13 | PRR13 | NM_018457 | Hs.426359 | chr12q12 |
| 217792_at | sorting nexin 5 | SNX5 | NM_014426 | Hs.13794 | chr20p11 |
| 217791_s_at | aldehyde dehydrogenase 18 family, member A1 | ALDH18A1 | NM_002860 | Hs.114366 | chr10q24.3 |
| 217789_at | sorting nexin 6 | SNX6 | NM_021249 | Hs.283443 | chr14q13.1 |
| 217786_at | protein arginine methyltransferase 5 | PRMT5 | NM_006109 | Hs.367854 | chr14q11.2-q21 |
| 217784_at | YKT6 v-SNARE homolog (S. cerevisiae) | YKT6 | BE384482 | Hs.296244 | chr7p15.1 |
| 217783_s_at | yippee-like 5 (Drosophila) | YPEL5 | NM_016061 | Hs.184542 | chr2p23.1 |
| 217780_at | chromosome 19 open reading frame 56 | C19orf56 | NM_016145 | Hs.108969 | chr19p13.13 |
| 217779_s_at | proline-rich nuclear receptor coactivator 2 | PNRC2 | NM_017761 | Hs.7862 | chr1p36.11 |
| 217778_at | solute carrier family 39 (zinc transporter), member 1 | SLC39A1 | NM_014437 | Hs.7854 | chr1q21 |
| 217777_s_at | protein tyrosine phosphatase-like A domain containing 1 /// similar to butyrate-induced transcript 1 | PTPLAD1 | NM_016395 | Hs.458288 | chr15q22.2 |
| 217775_s_at | retinol dehydrogenase 11 (all-trans/9-cis/11-cis) | RDH11 | NM_016026 | Hs.226007 | chr14q24.1 |
| 217774_s_at | hypothetical protein HSPC152 | HSPC152 | NM_016404 | Hs.333579 | chr11q13.1 |
| 217773_s_at | NADH dehydrogenase (ubiquinone) 1 alpha subcomplex, 4, 9kDa | NDUFA4 | NM_002489 | Hs.50098 | chr7p21.3 |
| 217771_at | golgi phosphoprotein 2 | GOLPH2 | NM_016548 | Hs.352662 | chr9q21.33 |
| 217768_at | chromosome 14 open reading frame 166 | C14orf166 | NM_016039 | Hs.369840 | chr14q22.1 |
| 217766_s_at | transmembrane protein 50A | TMEM50A | NM_014313 | Hs.475792 | chr1p36.11 |
| 217765_at | nuclear receptor binding protein 1 | NRBP1 | NM_013392 | Hs.272736 | chr2p23 |
| 217762_s_at | RAB31, member RAS oncogene family | RAB31 | BE789881 | Hs.223025 | chr18p11.3 |
| 217761_at | acireductone dioxygenase 1 | ADI1 | NM_018269 | Hs.502773 | chr2p25.2 |
| 217759_at | tripartite motif-containing 44 | TRIM44 | BF431488 | Hs.14512 | chr11p13 |
| 217758_s_at | transmembrane 9 superfamily member 3 | TM9SF3 | NM_020123 | Hs.8203 | chr10q24.1 |
| 217755_at | hematological and neurological expressed 1 | HN1 | NM_016185 | Hs.109706 | chr17q25.1 |
| 217754_at | DEAD (Asp-Glu-Ala-Asp) box polypeptide 56 | DDX56 | NM_019082 | Hs.10098 | chr7p13 |
| 217753_s_at | ribosomal protein S26 /// similar to 40S ribosomal protein S26 /// similar to 40S ribosomal protein S26 /// similar to 40S ribosomal protein S26 | RPS26 | NM_001029 | Hs.355957 | chr12q13 |
| 217752_s_at | CNDP dipeptidase 2 (metallopeptidase M20 family) | CNDP2 | NM_018235 | Hs.149185 | chr18q22.3 |
| 217751_at | glutathione S-transferase kappa 1 | GSTK1 | NM_015917 | Hs.390667 | chr7q35 |
| 217750_s_at | ubiquitin-conjugating enzyme E2Z (putative) | UBE2Z | NM_023079 | Hs.369120 | chr17q21.32 |
| 217749_at | coatomer protein complex, subunit gamma | COPG | NM_016128 | Hs.368056 | chr3q21.3 |
| 217748_at | adiponectin receptor 1 /// adiponectin receptor 1 | ADIPOR1 | NM_015999 | Hs.5298 | chr1p36.13-q41 |
| 217744_s_at | PERP, TP53 apoptosis effector | PERP | NM_022121 | Hs.149620 | chr6q24 |
| 217740_x_at | ribosomal protein L7a | RPL7A | NM_000972 | Hs.416801 | chr9q34 |
| 217737_x_at | chromosome 20 open reading frame 43 | C20orf43 | NM_016407 | Hs.380318 | chr20q13.31 |
| 217736_s_at | eukaryotic translation initiation factor 2-alpha kinase 1 | EIF2AK1 | NM_014413 | Hs.434986 | chr7p22 |
| 217733_s_at | thymosin, beta 10 | TMSB10 | NM_021103 | Hs.446574 | chr2p11.2 |
| 217731_s_at | integral membrane protein 2B | ITM2B | NM_021999 | Hs.446450 | chr13q14.3 |
| 217730_at | transmembrane BAX inhibitor motif containing 1 | TMBIM1 | NM_022152 | Hs.418542 | chr2p24.3-p24.1 |
| 217729_s_at | amino-terminal enhancer of split | AES | NM_001130 | Hs.446610 | chr19p13.3 |
| 217728_at | S100 calcium binding protein A6 | S100A6 | NM_014624 | Hs.275243 | chr1q21 |
| 217726_at | coatomer protein complex, subunit zeta 1 | COPZ1 | NM_016057 | Hs.181271 | chr12q13.2-q13.3 |
| 217722_s_at | neugrin, neurite outgrowth associated | NGRN | NM_016645 | Hs.124452 | chr15q26.1 |
| 217720_at | coiled-coil-helix-coiled-coil-helix domain containing 2 | CHCHD2 | NM_016139 | Hs.389996 | chr7p11.2 |
| 217719_at | eukaryotic translation initiation factor 3, subunit 6 interacting protein | EIF3S6IP | NM_016091 | Hs.119503 | chr22q |
| 217713_x_at | Transmembrane protein 163 | TMEM163 | AA126763 |  |  |
| 217704_x_at | Suppressor of zeste 12 homolog pseudogene | SUZ12P | AI820796 |  |  |
| 217703_x_at | Spondin 1, extracellular matrix protein | SPON1 | AA401963 |  |  |
| 217691_x_at | solute carrier family 16, member 3 (monocarboxylic acid transporter 4) | SLC16A3 | AA853175 | Hs.386678 | chr17q25 |
| 217665_at | FLJ16542 protein | FLJ16542 | AA420614 |  |  |
| 217645_at | Chromosome 14 open reading frame 112 | C14orf112 | AW088547 |  |  |
| 217644_s_at | son of sevenless homolog 2 (Drosophila) | SOS2 | AI276593 | Hs.528126 | chr14q21 |
| 217643_x_at | Hypothetical protein LOC440295 | LOC440295 | AA443771 |  |  |
| 217618_x_at | HUS1 checkpoint homolog (S. pombe) | HUS1 | AW007988 | Hs.152983 | chr7p13-p12 |
| 217608_at | P18SRP protein | P18SRP | AW408767 |  |  |
| 217599_s_at | MyoD family inhibitor domain containing | MDFIC | BE910600 | Hs.132739 | chr7q31.1-q31.2 |
| 217598_at | Cyclin-dependent kinase 2-interacting protein | CINP | BG236351 |  |  |
| 217591_at | SKI-like oncogene | SKIL | BF725121 |  |  |
| 217580_x_at | ADP-ribosylation factor-like 6 interacting protein 2 | ARL6IP2 | AW301806 |  |  |
| 217566_s_at | transglutaminase 4 (prostate) | TGM4 | BF222018 | Hs.438265 | chr3p22-p21.33 |
| 217551_at | similar to olfactory receptor, family 7, subfamily A, member 17 | LOC441453 | AA719797 | Hs.381327 | chr19p13.2 |
| 217547_x_at | zinc finger protein 675 | ZNF675 | BF308250 | Hs.264345 | chr19p12 |
| 217544_at | similar to Zinc finger protein 492 /// similar to Zinc finger protein 492 | LOC729806 | AA768909 |  |  |
| 217543_s_at | membrane-bound transcription factor peptidase, site 1 | MBTPS1 | BE890314 | Hs.75890 | chr16q24 |
| 217542_at | Carboxypeptidase M | CPM | BE930512 |  |  |
| 217541_x_at | hypothetical protein LOC125893 /// similar to zinc finger protein 160 | LOC125893 | BG290532 | Hs.446907 | chr19q13.41 |
| 217520_x_at | Hypothetical LOC646278 | LOC646278 | BG396614 | Hs.534745 | chr15q11.2 |
| 217506_at | Hypothetical protein LOC339290 | LOC339290 | H49382 |  |  |
| 217504_at | ATP-binding cassette, sub-family A (ABC1), member 6 | ABCA6 | AA099357 | Hs.15780 | chr17q24.3 |
| 217499_x_at | olfactory receptor, family 7, subfamily E, member 37 pseudogene | OR7E37P | AW874308 | Hs.507861 |  |
| 217496_s_at | insulin-degrading enzyme | IDE | AA918442 | Hs.290659 | chr10q23-q25 |
| 217478_s_at | major histocompatibility complex, class II, DM alpha | HLA-DMA | X76775 | Hs.351279 | chr6p21.3 |
| 217466_x_at | ribosomal protein S2 /// similar to ribosomal protein S2 /// hypothetical gene supported by AB082925; BC019021; NM_002952 /// similar to ribosomal protein S2 /// similar to ribosomal protein S2 /// hypothetical LOC645173 /// similar to ribosomal protein S | RPS2 | L48784 | Hs.498569 | chr16p13.3 |
| 217457_s_at | RAP1, GTP-GDP dissociation stimulator 1 | RAP1GDS1 | X63465 | Hs.132858 | chr4q23-q25 |
| 217452_s_at | UDP-Gal:betaGlcNAc beta 1,3-galactosyltransferase, polypeptide 2 | B3GALT2 | Y15014 | Hs.181353 | chr1q31 |
| 217448_s_at | KIAA0737 /// similar to Epidermal Langerhans cell protein LCP1 | KIAA0737 | AL117508 | Hs.194035 | chr14q11.2 |
| 217436_x_at | hypothetical protein LOC730399 /// hypothetical protein LOC731974 | LOC730399 | M80469 |  |  |
| 217428_s_at | collagen, type X, alpha 1(Schmid metaphyseal chondrodysplasia) | COL10A1 | X98568 | Hs.179729 | chr6q21-q22 |
| 217414_x_at | hemoglobin, alpha 1 /// hemoglobin, alpha 2 | HBA1 | V00489 | Hs.398636 | chr16p13.3 |
| 217403_s_at | zinc finger protein 227 | ZNF227 | AC074331 | Hs.371335 | chr19q13.32 |
| 217393_x_at | ubiquitin-conjugating enzyme E2N-like | UBE2NL | AL109622 |  |  |
| 217379_at | similar to ribosomal protein L10 | LOC442171 | AL121934 |  |  |
| 217367_s_at | zinc fingers and homeoboxes 3 | ZHX3 | AB007855 | Hs.380133 | chr20q12 |
| 217364_x_at | eukaryotic translation initiation factor 3, subunit 1 alpha, 35kDa /// hypothetical protein LOC730021 /// hypothetical protein LOC732068 | EIF3S1 | AL031313 | Hs.173987 | chr15q21.1 |
| 217353_at | heterogeneous nuclear ribonucleoprotein A1 /// similar to Heterogeneous nuclear ribonucleoprotein A1 (Helix-destabilizing protein) (Single-strand binding protein) (hnRNP core protein A1) (HDP-1) (Topoisomerase-inhibitor suppressed) /// heterogeneous nucle | HNRPA1 | AL022097 | Hs.356721 | chr12q13.1 |
| 217347_at | similar to 60S ribosomal protein L35 | LOC643653 | Z82202 |  |  |
| 217346_at | peptidylprolyl isomerase A (cyclophilin A) /// similar to peptidylprolyl isomerase A isoform 1 /// similar to peptidylprolyl isomerase A isoform 1 /// similar to peptidylprolyl isomerase A isoform 1 /// similar to peptidylprolyl isomerase A isoform 1 /// | PPIA | AL021395 |  |  |
| 217336_at | ribosomal protein S10 /// similar to ribosomal protein S10 /// ribosomal protein S10 pseudogene 3 /// similar to 40S ribosomal protein S10 /// hypothetical LOC376693 /// similar to 40S ribosomal protein S10 /// similar to 40S ribosomal protein S10 /// sim | RPS10LOC730965 | AL118510 | Hs.406620 | chr6p21.31 |
| 217317_s_at | hect domain and RLD 2 pseudogene 3 /// hect domain and RLD 2 pseudogene 2 /// hypothetical LOC440248 | HERC2P3 | AB002391 | Hs.458334 | chr15q11-q13 /// chr15q11.2 /// chr15q12 |
| 217299_s_at | nibrin | NBN | AK001017 | Hs.25812 | chr8q21 |
| 217297_s_at | myosin IXB | MYO9B | AF143684 | Hs.159629 | chr19p13.1 |
| 217287_s_at | transient receptor potential cation channel, subfamily C, member 6 | TRPC6 | AJ271068 | Hs.159003 | chr11q21-q22 |
| 217286_s_at | NDRG family member 3 | NDRG3 | BC001805 | Hs.437338 | chr20q11.21-q11.23 |
| 217256_x_at | similar to large subunit ribosomal protein L36a /// similar to large subunit ribosomal protein L36a /// similar to large subunit ribosomal protein L36a /// similar to large subunit ribosomal protein L36a /// similar to large subunit ribosomal protein L36a | LOC641903 | Z98950 |  |  |
| 217232_x_at | hemoglobin, beta | HBB | AF059180 | Hs.155376 | chr11p15.5 |
| 217226_s_at | sideroflexin 3 | SFXN3 | M95929 | Hs.443452 | chr1q24 /// chr10q24.32 |
| 217225_x_at | NODAL modulator 2 | NOMO2 | AL512687 | Hs.374735 | chr16p12.3 /// chr16p13.11 /// chr16p13 |
| 217221_x_at | RNA binding motif protein 10 | RBM10 | AL137421 | Hs.348276 | chrxp11.23 |
| 217216_x_at | mutL homolog 3 (E. coli) | MLH3 | AC006530 | Hs.279843 | chr14q24.3 |
| 217211_at | similar to cytoplasmic beta-actin | LOC390861 | D50604 |  |  |
| 217208_s_at | discs, large homolog 1 (Drosophila) /// tumor necrosis factor receptor superfamily, member 11b (osteoprotegerin) | DLG1 | AL121981 | Hs.389893 | chr3q29 |
| 217205_at | Coiled-coil domain containing 129 | CCDC129 | Y08772 |  |  |
| 217202_s_at | glutamate-ammonia ligase (glutamine synthetase) | GLUL | U08626 | Hs.442669 | chr1q31 |
| 217196_s_at | calmodulin regulated spectrin-associated protein 1-like 1 | CAMSAP1L1 | AL110158 | Hs.23585 | chr1q32.1 |
| 217188_s_at | chromosome 14 open reading frame 1 | C14orf1 | AC007182 | Hs.15106 | chr14q24.3 |
| 217185_s_at | zinc finger protein 259 /// similar to zinc finger protein 259 | ZNF259 | Z95118 | Hs.7165 | chr11q23.3 |
| 217176_s_at | zinc finger protein, X-linked | ZFX | X59740 | Hs.2074 | chrxp21.3 |
| 217173_s_at | low density lipoprotein receptor (familial hypercholesterolemia) | LDLR | S70123 | Hs.213289 | chr19p13.3 |
| 217168_s_at | homocysteine-inducible, endoplasmic reticulum stress-inducible, ubiquitin-like domain member 1 | HERPUD1 | AF217990 | Hs.146393 | chr16q12.2-q13 |
| 217161_x_at | aggrecan 1 (chondroitin sulfate proteoglycan 1, large aggregating proteoglycan, antigen identified by monoclonal antibody A0122) | AGC1 | X17406 | Hs.2159 | chr15q26.1 |
| 217133_x_at | cytochrome P450, family 2, subfamily B, polypeptide 6 | CYP2B6 | X06399 | Hs.1360 | chr19q13.2 |
| 217092_x_at | similar to 60S ribosomal protein L7 | LOC646912 | AL031589 | Hs.421257 | chr8q21.11 |
| 217073_x_at | apolipoprotein A-I | APOA1 | X02162 | Hs.93194 | chr11q23-q24 |
| 217043_s_at | synaptotagmin VII /// mitofusin 1 | SYT7 | U95822 | Hs.135624 | chr3q26.32 |
| 217039_x_at | Immunoglobulin heavy constant gamma 1 (G1m marker) | IGHG1 | AB016195 | Hs.181128 | chrxp11.2 |
| 217028_at | chemokine (C-X-C motif) receptor 4 | CXCR4 | AJ224869 | Hs.421986 | chr2q21 |
| 217027_x_at | karyopherin (importin) beta 1 | KPNB1 | AC004941 | Hs.439683 | chr17q21.32 |
| 217025_s_at | drebrin 1 | DBN1 | AL110225 | Hs.89434 | chr5q35.3 |
| 216996_s_at | FAST kinase domains 2 | FASTKD2 | AK021557 | Hs.84429 | chr2q33.3 |
| 216994_s_at | runt-related transcription factor 2 | RUNX2 | L40992 | Hs.122116 | chr6p21 |
| 216985_s_at | syntaxin 3 | STX3 | AJ002077 | Hs.82240 | chr11q12.1 |
| 216976_s_at | RYK receptor-like tyrosine kinase | RYK | X96588 | Hs.285346 | chr3q22 |
| 216960_s_at | zinc finger protein 133 | ZNF133 | AL049646 | Hs.78434 | chr20p11.23-20p11.22 |
| 216952_s_at | lamin B2 | LMNB2 | M94363 | Hs.76084 | chr19p13.3 |
| 216933_x_at | adenomatosis polyposis coli | APC | S67788 | Hs.75081 | chr5q21-q22 |
| 216913_s_at | ribosomal RNA processing 12 homolog (S. cerevisiae) | RRP12 | AK021460 | Hs.434251 | chr10q24.1 |
| 216908_x_at | RNA polymerase I transcription factor RRN3-like | LOC94431 | AF001549 | Hs.498879 | chr16p12.1 |
| 216903_s_at | calcium binding atopy-related autoantigen 1 | CBARA1 | AK022697 | Hs.61628 | chr10q22.1 |
| 216902_s_at | RRN3 RNA polymerase I transcription factor homolog (S. cerevisiae) /// hypothetical LOC653390 /// hypothetical protein LOC730092 | RRN3 | AF001549 | Hs.110103 | chr16p12 |
| 216883_x_at | phosphodiesterase 6D, cGMP-specific, rod, delta | PDE6D | AJ001626 | Hs.48291 | chr2q35-q36 |
| 216882_s_at | nebulette | NEBL | AL157398 | Hs.5025 | chr10p12 |
| 216881_x_at | proline-rich protein BstNI subfamily 4 | PRB4 | X07882 | Hs.528651 | chr12p13.2 |
| 216873_s_at | ATPase, Class I, type 8B, member 2 | ATP8B2 | AL137537 | Hs.43577 | chr1q21.3 |
| 216867_s_at | platelet-derived growth factor alpha polypeptide | PDGFA | X03795 | Hs.376032 | chr7p22 |
| 216863_s_at | MORC family CW-type zinc finger 2 | MORC2 | AC004542 | Hs.534437 | chr22q12.2 |
| 216841_s_at | superoxide dismutase 2, mitochondrial | SOD2 | X15132 | Hs.384944 | chr6q25.3 |
| 216840_s_at | laminin, alpha 2 (merosin, congenital muscular dystrophy) | LAMA2 | AK026829 | Hs.445120 | chr6q22-q23 |
| 216836_s_at | v-erb-b2 erythroblastic leukemia viral oncogene homolog 2, neuro/glioblastoma derived oncogene homolog (avian) | ERBB2 | X03363 | Hs.446352 | chr17q21.1 |
| 216823_at | ribosomal protein S3A /// similar to ribosomal protein S3a /// similar to 40S ribosomal protein S3a (V-fos transformation effector protein) /// similar to 40S ribosomal protein S3a (V-fos transformation effector protein) /// similar to 40S ribosomal prote | RPS3A | AL356115 |  |  |
| 216684_s_at | synovial sarcoma translocation, chromosome 18 | SS18 | AF257501 | Hs.404263 | chr18q11.2 |
| 216678_at | intraflagellar transport 122 homolog (Chlamydomonas) | IFT122 | AK000773 |  |  |
| 216627_s_at | UDP-Gal:betaGlcNAc beta 1,4- galactosyltransferase, polypeptide 1 | B4GALT1 | U10473 | Hs.396798 | chr9p13 |
| 216620_s_at | Rho guanine nucleotide exchange factor (GEF) 10 | ARHGEF10 | AF009205 | Hs.490857 | chr8p23 |
| 216609_at | Thioredoxin | TXN | AF065241 |  |  |
| 216606_x_at | lysophospholipase II pseudogene 1 | LYPLA2P1 | AL050332 |  | chr6p21.32 |
| 216605_s_at | carcinoembryonic antigen-related cell adhesion molecule 21 | CEACAM21 | AC005626 | Hs.528704 | chr19q13.2 |
| 216593_s_at | phosphatidylinositol glycan anchor biosynthesis, class C | PIGC | AB000359 | Hs.386487 | chr1q23-q25 |
| 216570_x_at | similar to 60S ribosomal protein L29 (P23) | LOC646417 | AL096829 |  |  |
| 216565_x_at | similar to Interferon-induced transmembrane protein 3 (Interferon-inducible protein 1-8U) | LOC391020 | AL121994 |  |  |
| 216547_at | similar to laminin receptor 1 (ribosomal protein SA) | LOC127406 | AL353681 | Hs.159818 | chr1p32.2 |
| 216532_x_at | similar to Thioredoxin-like protein 2 (PKC-interacting cousin of thioredoxin) (PKC-theta-interacting protein) (PKCq-interacting protein) /// similar to Thioredoxin-like protein 2 (PKC-interacting cousin of thioredoxin) (PKC-theta-interacting protein) (PKC | LOC643450 | AL138831 |  |  |
| 216531_at | YY2 transcription factor | YY2 | U73479 | Hs.534995 | chrxp22.2-p22.1 |
| 216521_s_at | BRCA1/BRCA2-containing complex, subunit 3 | BRCC3 | S72931 | Hs.301927 | chrxq28 |
| 216509_x_at | myeloid/lymphoid or mixed-lineage leukemia (trithorax homolog, Drosophila); translocated to, 10 | MLLT10 | AF060938 |  |  |
| 216490_x_at | similar to 60S acidic ribosomal protein P2 (NY-REN-44 antigen) | LOC442175 | AL133267 |  |  |
| 216483_s_at | chromosome 19 open reading frame 10 | C19orf10 | AC005339 | Hs.10927 | chr19p13.3 |
| 216438_s_at | thymosin, beta 4, X-linked /// thymosin-like 3 | TMSB4X | AL133228 | Hs.75968 | chrxq21.3-q22 /// chr4q22.1 |
| 216399_s_at | zinc finger protein 291 | ZNF291 | AK025663 | Hs.285848 | chr15q24 |
| 216392_s_at | SEC23 interacting protein | SEC23IP | AK021846 | Hs.300208 | chr10q25-q26 |
| 216387_x_at | similar to nucleophosmin 1 isoform 1 | LOC390411 | AL353580 |  |  |
| 216384_x_at | similar to prothymosin, alpha (gene sequence 28) | LOC643287 | AF257099 |  |  |
| 216383_at | ribosomal protein L18a /// similar to ribosomal protein L18a /// similar to ribosomal protein L18a /// similar to ribosomal protein L18a; 60S ribosomal protein L18a /// similar to ribosomal protein L18a /// similar to ribosomal protein L18a | RPL18A | U52111 | Hs.337766 | chr19p13 |
| 216380_x_at | similar to 40S ribosomal protein S28 /// similar to 40S ribosomal protein S28 /// similar to 40S ribosomal protein S28 | LOC728453 | AC005011 |  |  |
| 216347_s_at | protein phosphatase 1, regulatory (inhibitor) subunit 13B | PPP1R13B | AK023188 | Hs.371546 | chr14q32.33 |
| 216342_x_at | similar to 40S ribosomal protein S4, X isoform /// similar to 40S ribosomal protein S4, X isoform | LOC390183 | AL121916 |  |  |
| 216338_s_at | Yip1 domain family, member 3 | YIPF3 | AK021433 | Hs.440950 | chr6p21.1 |
| 216336_x_at | metallothionein 1M | MT1M | AL031602 |  |  |
| 216331_at | integrin, alpha 7 | ITGA7 | AK022548 | Hs.524484 | chr12q13 |
| 216326_s_at | histone deacetylase 3 | HDAC3 | AF059650 | Hs.388681 | chr5q31 |
| 216323_x_at | alpha-tubulin isotype H2-alpha | H2-ALPHA | K03460 |  |  |
| 216321_s_at | nuclear receptor subfamily 3, group C, member 1 (glucocorticoid receptor) | NR3C1 | X03348 | Hs.126608 | chr5q31 |
| 216316_x_at | glycerol kinase /// glycerol kinase 3 pseudogene | GK /// GK3P | X78713 | Hs.1466 | chrxp21.3 |
| 216315_x_at | ubiquitin-conjugating enzyme E2 variant 1 /// ubiquitin-conjugating enzyme E2 variant 1 /// similar to ubiquitin-conjugating enzyme E2 variant 1 isoform d /// similar to ubiquitin-conjugating enzyme E2 variant 1 isoform d | UBE2V1 | AL121873 | Hs.381025 | chr20q13.2 |
| 216309_x_at | jerky homolog (mouse) | JRK | AF072467 | Hs.521901 | chr8q24.3 |
| 216305_s_at | chromosome 2 open reading frame 3 | C2orf3 | AC005034 | Hs.158969 | chr2p11.2-p11.1 |
| 216282_x_at | polymerase (RNA) II (DNA directed) polypeptide C, 33kDa | POLR2C | AJ224143 | Hs.279832 | chr16q13-q21 |
| 216267_s_at | transmembrane protein 115 | TMEM115 | BF034906 | Hs.91566 | chr3p21.3 |
| 216264_s_at | laminin, beta 2 (laminin S) | LAMB2 | X79683 | Hs.439726 | chr3p21 |
| 216252_x_at | Fas (TNF receptor superfamily, member 6) | FAS | Z70519 | Hs.82359 | chr10q24.1 |
| 216241_s_at | transcription elongation factor A (SII), 1 | TCEA1 | X57198 | Hs.78869 | chr8q11.2 |
| 216237_s_at | MCM5 minichromosome maintenance deficient 5, cell division cycle 46 (S. cerevisiae) | MCM5 | AA807529 | Hs.77171 | chr22q13.1 |
| 216232_s_at | GCN1 general control of amino-acid synthesis 1-like 1 (yeast) | GCN1L1 | AI697055 | Hs.298716 | chr12q24.2 |
| 216229_x_at | HLA complex group 2 pseudogene 7 | HCG2P7 | X81001 | Hs.512758 | chr6p21.3 |
| 216215_s_at | Ribosomal protein L41 | RPL41 | AL049748 | Hs.433574 | chr22q13.1 |
| 216212_s_at | dyskeratosis congenita 1, dyskerin | DKC1 | AJ010395 | Hs.4747 | chrxq28 |
| 216205_s_at | mitofusin 2 | MFN2 | AK021947 | Hs.376681 | chr1p36.22 |
| 216202_s_at | serine palmitoyltransferase, long chain base subunit 2 | SPTLC2 | U15555 | Hs.59403 | chr14q24.3-q31 |
| 216177_at | similar to 60S ribosomal protein L29 (P23) | LOC391132 | AW582267 |  |  |
| 216125_s_at | RAN binding protein 9 | RANBP9 | AF064606 | Hs.106346 | chr6p23 |
| 216111_x_at | postmeiotic segregation increased 2-like 3 | PMS2L3 | U38979 | Hs.278467 | chr7q11.23 |
| 216092_s_at | solute carrier family 7 (cationic amino acid transporter, y+ system), member 8 | SLC7A8 | AL365347 | Hs.22891 | chr14q11.2 |
| 216080_s_at | fatty acid desaturase 3 | FADS3 | AC004770 | Hs.21765 | chr11q12-q13.1 |
| 216064_s_at | aspartylglucosaminidase | AGA | W27131 | Hs.207776 | chr4q32-q33 |
| 216060_s_at | dishevelled associated activator of morphogenesis 1 | DAAM1 | AK021890 | Hs.356537 | chr14q23.1 |
| 216054_x_at | myosin, light chain 4, alkali; atrial, embryonic | MYL4 | X58851 | Hs.356717 | chr17q21-qter |
| 216049_at | Rho-related BTB domain containing 3 | RHOBTB3 | AK023621 | Hs.31653 | chr5q15 |
| 216044_x_at | family with sequence similarity 69, member A | FAM69A | AK027146 | Hs.180946 | chr1p22.1 |
| 216036_x_at | WD and tetratricopeptide repeats 1 | WDTC1 | AK001734 | Hs.469154 | chr1p36.11 |
| 216032_s_at | ERGIC and golgi 3 | ERGIC3 | AF091085 | Hs.169992 | chr20pter-q12 |
| 216028_at | DKFZP564C152 protein | DKFZP564C152 | AL049980 |  |  |
| 216006_at | WAS/WASL interacting protein family, member 2 | WIPF2 | AF070620 | Hs.514856 |  |
| 216005_at | Tenascin C (hexabrachion) | TNC | BF434846 | Hs.98998 | chr9q33 |
| 215984_s_at | ADP-ribosylation factor related protein 1 | ARFRP1 | AL121845 | Hs.389277 | chr20q13.3 |
| 215983_s_at | UBX domain containing 6 | UBXD6 | D83768 | Hs.153678 | chr8p12-p11.2 |
| 215978_x_at | hypothetical protein LOC152719 | LOC152719 | AK021514 |  |  |
| 215977_x_at | glycerol kinase | GK | X68285 | Hs.1466 | chrxp21.3 |
| 215963_x_at | similar to ribosomal protein L3 isoform a | LOC642741 | Z98200 |  |  |
| 215947_s_at | hypothetical protein FLJ14668 | FLJ14668 | AF090094 | Hs.334644 | chr2p13.3 |
| 215936_s_at | KIAA1033 | KIAA1033 | AK001657 | Hs.12144 | chr12q24.11 |
| 215933_s_at | homeobox, hematopoietically expressed | HHEX | Z21533 | Hs.118651 | chr10q23.33 |
| 215930_s_at | CTAGE family, member 5 | CTAGE5 | U73682 | Hs.434485 | chr14q13.3 |
| 215905_s_at | WD repeat domain 57 (U5 snRNP specific) | WDR57 | AL157420 | Hs.379357 | chr1p35.2 |
| 215884_s_at | ubiquilin 2 | UBQLN2 | AK001029 | Hs.4552 | chrxp11.23-p11.1 |
| 215873_x_at | ATP-binding cassette, sub-family C (CFTR/MRP), member 10 | ABCC10 | AK024446 | Hs.337251 | chr6p21.1 |
| 215870_s_at | phospholipase A2, group V | PLA2G5 | AL158172 | Hs.319438 | chr1p36-p34 |
| 215812_s_at | solute carrier family 6 (neurotransmitter transporter, creatine), member 8 /// solute carrier family 6 (neurotransmitter transporter, creatine), member 10 (pseudogene) /// similar to Sodium- and chloride-dependent creatine transporter 1 (CT1) (Creatine tr | SLC6A8 | U41163 | Hs.388375 | chrxq28 /// chr16p11.2 |
| 215794_x_at | glutamate dehydrogenase 2 | GLUD2 | AC006144 | Hs.368538 | chrxq24-q25 |
| 215792_s_at | DnaJ (Hsp40) homolog, subfamily C, member 11 | DNAJC11 | AL109978 | Hs.462626 | chr1p36.31 |
| 215785_s_at | cytoplasmic FMR1 interacting protein 2 | CYFIP2 | AL161999 | Hs.211201 | chr5q33.3 |
| 215766_at | Glutathione S-transferase A1 | GSTA1 | AL096729 | Hs.446309 | chr6p12.1 |
| 215764_x_at | adaptor-related protein complex 2, alpha 2 subunit | AP2A2 | AA877641 | Hs.19121 | chr11p15.5 |
| 215750_at | KIAA1659 protein | KIAA1659 | AB051446 |  | chr22q13 |
| 215747_s_at | regulator of chromosome condensation 1 /// regulator of chromosome condensation 1 | RCC1 | X06130 | Hs.196769 | chr1p36.1 |
| 215737_x_at | upstream transcription factor 2, c-fos interacting | USF2 | X90824 | Hs.454534 | chr19q13 |
| 215735_s_at | tuberous sclerosis 2 | TSC2 | AC005600 | Hs.90303 | chr16p13.3 |
| 215718_s_at | PHD finger protein 3 | PHF3 | AI949220 | Hs.348921 | chr6q12 |
| 215714_s_at | SWI/SNF related, matrix associated, actin dependent regulator of chromatin, subfamily a, member 4 | SMARCA4 | AF254822 | Hs.78202 | chr19p13.2 |
| 215710_at | ST3 beta-galactoside alpha-2,3-sialyltransferase 4 | ST3GAL4 | AK021929 | Hs.75268 | chr11q23-q24 |
| 215707_s_at | prion protein (p27-30) (Creutzfeldt-Jakob disease, Gerstmann-Strausler-Scheinker syndrome, fatal familial insomnia) | PRNP | AV725328 | Hs.438582 | chr20pter-p12 |
| 215706_x_at | zyxin | ZYX | BC002323 | Hs.75873 | chr7q32 |
| 215696_s_at | KIAA0310 | KIAA0310 | BC001404 | Hs.396443 | chr9q34.3 |
| 215691_x_at | interleukin 17 receptor B | IL17RB | AV702994 | Hs.439171 | chr1p32.1-p33 |
| 215684_s_at | activating signal cointegrator 1 complex subunit 2 | ASCC2 | AL096741 | Hs.436407 | chr22q12.1 |
| 215667_x_at | similar to postmeiotic segregation increased 2-like 2 /// similar to postmeiotic segregation increased 2-like 2 /// similar to postmeiotic segregation increased 2-like 2 | LOC441259 | AI375694 | Hs.292996 | chr7q11-q22 |
| 215631_s_at | breast cancer metastasis suppressor 1 | BRMS1 | AL050008 | Hs.100426 | chr11q13-q13.2 |
| 215629_s_at | deleted in lymphocytic leukemia 2-like | DLEU2L | AA905286 | Hs.446406 | chr13q14.3 |
| 215600_x_at | F-box and WD-40 domain protein 12 | FBXW12 | AK022174 | Hs.288793 | chr3p21.31 |
| 215587_x_at | BTB (POZ) domain containing 14B | BTBD14B | AK023891 | Hs.185254 | chr19p13.13 |
| 215577_at | Ubiquitin-conjugating enzyme E2E 1 (UBC4/5 homolog, yeast) | UBE2E1 | AU146791 | Hs.163546 | chr3p24.2 |
| 215566_x_at | lysophospholipase II | LYPLA2 | AK024724 | Hs.413781 | chr1p36.12-p35.1 |
| 215561_s_at | interleukin 1 receptor, type I | IL1R1 | AK026803 | Hs.82112 | chr2q12 |
| 215548_s_at | sec1 family domain containing 1 | SCFD1 | AB020724 | Hs.27023 | chr14q12 |
| 215541_s_at | diaphanous homolog 1 (Drosophila) | DIAPH1 | AK023345 | Hs.432623 | chr5q31 |
| 215537_x_at | dimethylarginine dimethylaminohydrolase 2 | DDAH2 | AJ012008 | Hs.247362 | chr6p21.3 |
| 215532_x_at | poly(A) binding protein interacting protein 1 /// zinc finger protein 492 /// similar to Zinc finger protein 492 /// similar to Zinc finger protein 492 /// similar to Zinc finger protein 492 | PAIP1 | AB040906 | Hs.232108 | chr19p12 |
| 215519_x_at | RUN and TBC1 domain containing 3 | RUTBC3 | AI081779 | Hs.22129 | chr22q13.1-q13.2 |
| 215511_at | transcription factor 20 (AR1) | TCF20 | U19345 |  |  |
| 215506_s_at | DIRAS family, GTP-binding RAS-like 3 | DIRAS3 | AK021882 | Hs.194695 | chr1p31 |
| 215501_s_at | dual specificity phosphatase 10 | DUSP10 | AK022513 | Hs.177534 | chr1q41 |
| 215489_x_at | homer homolog 3 (Drosophila) | HOMER3 | AI871287 | Hs.410683 | chr19p13.11 |
| 215482_s_at | eukaryotic translation initiation factor 2B, subunit 4 delta, 67kDa | EIF2B4 | AJ011307 | Hs.169474 | chr2p23.3 |
| 215470_at | similar to TFIIH basal transcription factor complex p44 subunit (Basic transcription factor 2 44 kDa subunit) (BTF2-p44) (General transcription factor IIH polypeptide 2) | DKFZP686M0199 | U21915 | Hs.422901 |  |
| 215467_x_at | hypothetical LOC647070 | LOC647070 | AK001442 | Hs.374524 | chr1q25 |
| 215460_x_at | bromodomain containing 1 | BRD1 | AL080149 | Hs.127950 | chr22q13.33 |
| 215452_x_at | SMT3 suppressor of mif two 3 homolog 4 (S. cerevisiae) | SUMO4 | AL031133 |  | chr6q25 |
| 215440_s_at | brain expressed X-linked-like 1 | BEXL1 | AL523320 | Hs.184736 | chrXq22.1-q22.3 |
| 215436_at | Hydroxysteroid dehydrogenase like 2 | HSDL2 | AK023959 |  |  |
| 215435_at | SCC-112 protein | SCC-112 | AK021983 |  |  |
| 215429_s_at | zinc finger protein 428 | ZNF428 | BE045982 | Hs.99093 | chr19q13.31 |
| 215418_at | parvin, alpha | PARVA | AK022316 | Hs.44077 | chr11p15.3 |
| 215417_at | exocyst complex component 6B | EXOC6B | AI803703 | Hs.44175 | chr2p13.2 |
| 215416_s_at | stomatin (EPB72)-like 2 | STOML2 | AC004472 | Hs.3439 | chr9p13.1 |
| 215412_x_at | postmeiotic segregation increased 2-like 2 | PMS2L2 | AB017007 | Hs.292996 | chr7q11-q22 |
| 215411_s_at | TRAF3 interacting protein 2 | TRAF3IP2 | AL008730 | Hs.437508 | chr6q21 |
| 215383_x_at | spastic paraplegia 21, maspardin (autosomal recessive, Mast syndrome) | SPG21 | AL137312 | Hs.242458 | chr15q21-q22 |
| 215380_s_at | chromosome 7 open reading frame 24 | C7orf24 | AK021779 | Hs.444840 | chr7p15-p14 |
| 215373_x_at | hypothetical protein FLJ12151 | FLJ12151 | AK022213 |  |  |
| 215357_s_at | polymerase (DNA-directed), delta interacting protein 3 | POLDIP3 | Z93241 | Hs.458405 | chr22q13.2 |
| 215354_s_at | proline, glutamic acid and leucine rich protein 1 | PELP1 | BC002875 | Hs.409251 | chr17p13.2 |
| 215342_s_at | RAB GTPase activating protein 1-like | RABGAP1L | AB019490 | Hs.258035 | chr1q24 |
| 215336_at | A kinase (PRKA) anchor protein 11 | AKAP11 | AK002166 | Hs.414995 | chr13q14.11 |
| 215321_at | Rap2-binding protein 9 | RPIB9 | AI825798 | Hs.411488 | chr7q21.12 |
| 215307_at | Zinc finger protein 529 | ZNF529 | AL109722 |  |  |
| 215293_s_at | FGF receptor activating protein 1 | FRAG1 | AL049261 | Hs.133968 | chr11p15.5 |
| 215285_s_at | putative homeodomain transcription factor 1 | PHTF1 | AA927671 | Hs.416362 | chr1p13 |
| 215283_at | hypothetical gene supported by BC041875; BX648984 | LOC400642 | U79248 | Hs.435676 | chr18p11.31 |
| 215281_x_at | pogo transposable element with ZNF domain | POGZ | AK000199 | Hs.107088 | chr1q21.3 |
| 215273_s_at | transcriptional adaptor 3 (NGG1 homolog, yeast)-like | TADA3L | AK024982 | Hs.386390 | chr3p25.3 |
| 215253_s_at | Down syndrome critical region gene 1 | DSCR1 | AL049369 | Hs.282326 | chr21q22.12 |
| 215239_x_at | zinc finger protein 273 | ZNF273 | AU132789 | Hs.386264 | chr7q11.21 |
| 215236_s_at | phosphatidylinositol binding clathrin assembly protein | PICALM | AV721177 | Hs.39252 | chr11q14 |
| 215193_x_at | major histocompatibility complex, class II, DR beta 1 /// major histocompatibility complex, class II, DR beta 3 /// hypothetical protein LOC730415 | HLA-DRB1 | AJ297586 |  | chr6p21.3 |
| 215182_x_at | Hypothetical protein LOC730096 | LOC730096 | AL050122 | Hs.274511 |  |
| 215177_s_at | integrin, alpha 6 | ITGA6 | AV733308 | Hs.212296 | chr2q31.1 |
| 215169_at | solute carrier family 35, member E2 | SLC35E2 | BE885244 | Hs.214646 | chr1p36.33 |
| 215160_x_at | similar to FRG1 protein (FSHD region gene 1 protein) | LOC642236 | AL441988 | Hs.459740 | chr9q12 |
| 215157_x_at | poly(A) binding protein, cytoplasmic 1 | PABPC1 | AI734929 | Hs.387804 | chr8q22.2-q23 |
| 215148_s_at | amyloid beta (A4) precursor protein-binding, family A, member 3 (X11-like 2) | APBA3 | AI141541 | Hs.17528 | chr19p13.3 |
| 215136_s_at | exosome component 8 | EXOSC8 | AL050353 | Hs.274170 | chr13q13.1 |
| 215134_at | phosphatidylinositol 4-kinase type II | PI4KII | H84390 | Hs.25300 | chr10q24 |
| 215133_s_at | hypothetical protein LOC202134 /// NY-REN-7 antigen | LOC202134 | AL117630 | Hs.187203 | chr5q35.2 |
| 215095_at | Esterase D/formylglutathione hydrolase | ESD | AU145746 | Hs.432491 | chr13q14.1-q14.2 |
| 215090_x_at | hypothetical protein FLJ11822 | LOC440434 | AK021884 | Hs.449880 | chr17q21.1 |
| 215084_s_at | leucine rich repeat containing 42 | LRRC42 | AL031427 | Hs.40094 | chr1p33-p32.1 |
| 215082_at | Taurine upregulated gene 1 /// ELOVL family member 5, elongation of long chain fatty acids (FEN1/Elo2, SUR4/Elo3-like, yeast) | TUG1 | BF973387 | Hs.343667 | chr6p21.1-p12.1 |
| 215075_s_at | growth factor receptor-bound protein 2 | GRB2 | L29511 | Hs.411366 | chr17q24-q25 |
| 215071_s_at | histone cluster 1, H2ac | HIST1H2AC | AL353759 |  |  |
| 215069_at | N-myristoyltransferase 2 | NMT2 | AK025065 | Hs.311589 | chr10p13 |
| 215068_s_at | F-box and leucine-rich repeat protein 18 | FBXL18 | BC004228 | Hs.34790 | chr7p22.1 |
| 215063_x_at | leucine rich repeat containing 40 | LRRC40 | AL390149 | Hs.283862 | chr1p31.1 |
| 215044_s_at | signal transducing adaptor molecule (SH3 domain and ITAM motif) 2 | STAM2 | AI571996 | Hs.17200 | chr2q23.3 |
| 215032_at | ras responsive element binding protein 1 | RREB1 | AK022442 | Hs.171942 | chr6p25 |
| 215030_at | G-rich RNA sequence binding factor 1 | GRSF1 | AK023187 | Hs.309763 | chr4q13 |
| 215028_at | sema domain, transmembrane domain (TM), and cytoplasmic domain, (semaphorin) 6A | SEMA6A | AB002438 | Hs.443012 | chr5q23.1 |
| 215023_s_at | peroxisome biogenesis factor 1 | PEX1 | AC000064 | Hs.164682 | chr7q21-q22 |
| 215022_x_at | zinc finger protein 33B | ZNF33B | BG429214 | Hs.434201 | chr10q11.2 |
| 215019_x_at | zinc finger protein 528 | ZNF528 | AW474158 | Hs.249479 | chr19q13 |
| 215005_at | EF-hand calcium binding protein 2 | EFCBP2 | AV723666 | Hs.140950 | chr16q23.3 |
| 215004_s_at | splicing factor 4 | SF4 | AC004475 | Hs.436041 | chr19p13.11 |
| 214995_s_at | apolipoprotein B mRNA editing enzyme, catalytic polypeptide-like 3G /// apolipoprotein B mRNA editing enzyme, catalytic polypeptide-like 3F | APOBEC3G | BF508948 | Hs.286849 | chr22q13.1-q13.2 /// chr22q13.1 |
| 214992_s_at | deoxyribonuclease II, lysosomal | DNASE2 | AD000092 | Hs.118243 | chr19p13.2 |
| 214989_x_at | Pleckstrin homology domain containing, family A member 5 | PLEKHA5 | BC000969 | Hs.242537 | chr12p12 |
| 214965_at | chromosome 16 open reading frame 76 | C16orf76 | AF070574 | Hs.374556 | chr16q24.3 |
| 214962_s_at | nucleoporin 160kDa | NUP160 | AK026236 | Hs.22559 | chr11p11.2 |
| 214960_at | apoptosis inhibitor 5 | API5 | AF229253 | Hs.444340 | chr11p12-q12 |
| 214943_s_at | RNA binding motif protein 34 | RBM34 | D38491 | Hs.511939 | chr1q42.3 |
| 214934_at | ATPase, Class II, type 9B | ATP9B | AW411030 | Hs.321101 | chr18q23 |
| 214933_at | calcium channel, voltage-dependent, P/Q type, alpha 1A subunit | CACNA1A | AA769818 | Hs.400460 | chr19p13.2-p13.1 |
| 214931_s_at | SFRS protein kinase 2 | SRPK2 | AC005070 | Hs.221688 | chr7q22-q31.1 |
| 214923_at | ATPase, H+ transporting, lysosomal 34kDa, V1 subunit D | ATP6V1D | AK001155 | Hs.272630 | chr14q23-q24.2 |
| 214919_s_at | eukaryotic translation initiation factor 4E binding protein 3 /// MASK-4E-BP3 alternate reading frame gene | EIF4EBP3 | R39094 |  | chr5q31.3 |
| 214913_at | ADAM metallopeptidase with thrombospondin type 1 motif, 3 | ADAMTS3 | AB002364 | Hs.151435 | chr4q13.3 |
| 214895_s_at | ADAM metallopeptidase domain 10 | ADAM10 | AU135154 | Hs.172028 | chr15q22 |
| 214881_s_at | upstream binding transcription factor, RNA polymerase I | UBTF | X56687 | Hs.89781 | chr17q21.3 |
| 214875_x_at | amyloid beta (A4) precursor-like protein 2 | APLP2 | AW001847 | Hs.279518 | chr11q24 |
| 214866_at | plasminogen activator, urokinase receptor | PLAUR | X74039 | Hs.179657 | chr19q13 |
| 214850_at | glucuronidase, beta pseudogene 1 | GUSBP1 | X75940 | Hs.166361 | chr5q13 |
| 214843_s_at | ubiquitin specific peptidase 33 | USP33 | AK022864 | Hs.173694 | chr1p31.1 |
| 214838_at | SFT2 domain containing 2 | SFT2D2 | AL035297 | Hs.246759 | chr1q24.2 |
| 214835_s_at | succinate-CoA ligase, GDP-forming, beta subunit | SUCLG2 | AF131748 | Hs.128589 | chr3p14.1 |
| 214830_at | solute carrier family 38, member 6 | SLC38A6 | AI537540 | Hs.200738 | chr14q23.1 |
| 214820_at | bromodomain and WD repeat domain containing 1 | BRWD1 | AJ002572 | Hs.435904 | chr21q22.2 |
| 214816_x_at | chromosome 19 open reading frame 40 | C19orf40 | BC003535 | Hs.192127 | chr19q13.11 |
| 214814_at | YTH domain containing 1 | YTHDC1 | BF592058 | Hs.86405 | chr4q13.2 |
| 214794_at | dihydrolipoamide S-succinyltransferase (E2 component of 2-oxo-glutarate complex) | DLST | BF669264 |  |  |
| 214791_at | hypothetical protein BC004921 | LOC93349 | AK023116 | Hs.125635 | chr2q37.1 |
| 214790_at | SUMO1/sentrin specific peptidase 6 | SENP6 | AK001406 | Hs.435628 | chr6q13-q14.3 |
| 214787_at | DENN/MADD domain containing 4A | DENND4A | BE268538 | Hs.283921 | chr15q22.31 |
| 214784_x_at | exportin 6 | XPO6 | BE966299 | Hs.150684 | chr16p11.2 |
| 214783_s_at | annexin A11 | ANXA11 | BG177920 | Hs.75510 | chr10q23 |
| 214778_at | multiple EGF-like-domains 8 | MEGF8 | AB011541 | Hs.158200 | chr19q12 |
| 214766_s_at | AT hook containing transcription factor 1 | AHCTF1 | AL080144 | Hs.33363 | chr1q44 |
| 214764_at | KIAA0507 | KIAA0507 | AW029169 | Hs.497770 |  |
| 214754_at | hypothetical protein MGC22014 | MGC22014 | AB007861 | Hs.447988 | chr2p13.1 |
| 214751_at | zinc finger protein 468 | ZNF468 | BE541042 | Hs.448945 | chr19q13.41 |
| 214749_s_at | armadillo repeat containing, X-linked 6 | ARMCX6 | AK000818 | Hs.83530 | chrxq21.33-q22.3 |
| 214748_at | CG016 | RP11-298P3.3 | U50529 |  | chr13q13.1 |
| 214744_s_at | Ribosomal protein L23 | RPL23 | AK021960 |  |  |
| 214739_at | leucine-rich repeats and calponin homology (CH) domain containing 3 | LRCH3 | AI357539 | Hs.334483 | chr3q29 |
| 214733_s_at | Yip1 domain family, member 1 /// interleukin 17 receptor B | YIPF1 | AL031427 | Hs.11923 | chr1p33-p32.1 |
| 214726_x_at | adducin 1 (alpha) | ADD1 | AL556041 | Hs.183706 | chr4p16.3 |
| 214724_at | DIX domain containing 1 | DIXDC1 | AF070621 | Hs.116796 | chr11q23.2 |
| 214723_x_at | KIAA1641 | KIAA1641 | AB046861 | Hs.469358 | chr2q11.2 |
| 214722_at | Notch homolog 2 (Drosophila) N-terminal like | NOTCH2NL | AW516297 | Hs.438837 | chr1q21.2 |
| 214720_x_at | septin 10 | septin 10 | BF981643 | Hs.355455 | chr2q13 |
| 214719_at | hypothetical protein LOC283537 | LOC283537 | AK026720 | Hs.117167 | chr13q12.3 |
| 214716_at | BMP2 inducible kinase | BMP2K | AW504018 | Hs.368305 | chr4q21.21 |
| 214714_at | zinc finger protein 394 | ZNF394 | AK022360 | Hs.386324 | chr7q22.1 |
| 214711_at | hypothetical protein LOC283459 | 15E1.2 | BE568184 | Hs.77608 | chr12q24.31 |
| 214710_s_at | cyclin B1 | CCNB1 | BE407516 | Hs.15740 | chr5q12 |
| 214707_x_at | Alstrom syndrome 1 | ALMS1 | AB002326 | Hs.97393 | chr2p13 |
| 214706_at | zinc finger protein 200 | ZNF200 | AU149447 | Hs.88219 | chr16p13.3 |
| 214703_s_at | mannosidase, alpha, class 2B, member 2 | MAN2B2 | AW954107 | Hs.200661 | chr4p16.1 |
| 214699_x_at | WD repeat domain, phosphoinositide interacting 2 | WIPI2 | AK024279 | Hs.226372 | chr7p22.1 |
| 214698_at | ROD1 regulator of differentiation 1 (S. pombe) | ROD1 | AW190873 | Hs.374634 | chr9q32 |
| 214696_at | hypothetical protein MGC14376 | MGC14376 | AF070569 | Hs.417157 | chr17p13.3 |
| 214695_at | ubiquitin associated protein 2-like | UBAP2L | AW051361 | Hs.8127 | chr1q21.3 |
| 214693_x_at | calcitonin/calcitonin-related polypeptide, alpha /// calcitonin-related polypeptide, beta /// neuroblastoma breakpoint family, member 14 /// DKFZP564O0823 protein /// neuroblastoma breakpoint family, member 1 /// par-3 partitioning defective 3 homolog B ( | CALCA | BE732345 | Hs.488862 | chr1q21.1 /// chr1q21.2 /// chr1q12-1q21.2 /// chr1 |
| 214686_at | zinc finger protein 266 | ZNF266 | AA868898 | Hs.118281 | chr19p13.2 |
| 214683_s_at | CDC-like kinase 1 | CLK1 | AI251890 | Hs.433732 | chr2q33 |
| 214679_x_at | guanine nucleotide binding protein (G protein), alpha 11 (Gq class) | GNA11 | AL110227 | Hs.435670 | chr19p13.3 |
| 214672_at | tubulin tyrosine ligase-like family, member 5 | TTLL5 | AB023215 | Hs.435270 | chr14q24.3 |
| 214663_at | receptor interacting protein kinase 5 | RIPK5 | AB007941 | Hs.6874 | chr1q32.1 |
| 214662_at | WD repeat domain 43 | WDR43 | D26488 | Hs.169863 | chr2p23.2 |
| 214661_s_at | chromosome 4 open reading frame 9 | C4orf9 | R06783 | Hs.398178 | chr4p16.3 |
| 214660_at | Pelota homolog (Drosophila) | PELO | X68742 | Hs.439320 | chr5q11.2 |
| 214656_x_at | myosin IC | MYO1C | BE790157 | Hs.286226 | chr17p13 |
| 214605_x_at | G protein-coupled receptor 1 | GPR1 | AL046992 | Hs.184907 | chr2q33.3 |
| 214590_s_at | ubiquitin-conjugating enzyme E2D 1 (UBC4/5 homolog, yeast) | UBE2D1 | AL545760 | Hs.129683 | chr10q11.2-q21 |
| 214589_at | fibroblast growth factor 12 | FGF12 | AL119322 | Hs.386193 | chr3q28 |
| 214585_s_at | vacuolar protein sorting 52 homolog (S. cerevisiae) | VPS52 | AL390171 | Hs.433731 | chr6p21.3 |
| 214583_at | regulatory solute carrier protein, family 1, member 1 | RSC1A1 | AI268381 | Hs.239459 | chr1p36.1 |
| 214578_s_at | Rho-associated, coiled-coil containing protein kinase 1 | ROCK1 | AV683882 | Hs.306307 | chr18q11.1 |
| 214577_at | microtubule-associated protein 1B | MAP1B | BG164365 | Hs.103042 | chr5q13 |
| 214553_s_at | cyclic AMP phosphoprotein, 19 kD | ARPP-19 | NM_006628 | Hs.7351 | chr15q21.2 |
| 214545_s_at | proline synthetase co-transcribed homolog (bacterial) | PROSC | NM_007198 | Hs.301959 | chr8p11.2 |
| 214543_x_at | quaking homolog, KH domain RNA binding (mouse) | QKI | AF142421 | Hs.153355 | chr6q26-27 |
| 214526_x_at | postmeiotic segregation increased 2-like 1 | PMS2L1 | NM_005394 |  |  |
| 214522_x_at | histone cluster 1, H3d | HIST1H3D | NM_021065 | Hs.239458 | chr6p21.3 |
| 214501_s_at | toll-like receptor 4 /// H2A histone family, member Y | TLR4 | AF044286 | Hs.75258 | chr5q31.3-q32 |
| 214486_x_at | CASP8 and FADD-like apoptosis regulator | CFLAR | AF041459 | Hs.355724 | chr2q33-q34 |
| 214484_s_at | opioid receptor, sigma 1 | OPRS1 | AF226604 | Hs.24447 | chr9p13.3 |
| 214483_s_at | ADP-ribosylation factor interacting protein 1 (arfaptin 1) | ARFIP1 | AF124489 | Hs.416089 | chr4q31.3 |
| 214474_at | protein kinase, AMP-activated, beta 2 non-catalytic subunit | PRKAB2 | NM_005399 | Hs.50732 | chr1q21.1 |
| 214455_at | histone cluster 1, H2bg /// histone cluster 1, H2bc | HIST1H2BG | NM_003526 |  |  |
| 214453_s_at | interferon-induced protein 44 | IFI44 | NM_006417 | Hs.82316 | chr1p31.1 |
| 214440_at | N-acetyltransferase 1 (arylamine N-acetyltransferase) | NAT1 | NM_000662 | Hs.458430 | chr8p23.1-p21.3 |
| 214436_at | F-box and leucine-rich repeat protein 2 | FBXL2 | AF176518 | Hs.6092 | chr3p23 |
| 214435_x_at | v-ral simian leukemia viral oncogene homolog A (ras related) | RALA | NM_005402 | Hs.6906 | chr7p15-p13 |
| 214434_at | heat shock 70kDa protein 12A | HSPA12A | AB007877 | Hs.372597 |  |
| 214431_at | guanine monphosphate synthetase | GMPS | NM_003875 | Hs.144057 | chr3q24 |
| 214430_at | galactosidase, alpha | GLA | NM_000169 | Hs.69089 | chrxq22 |
| 214429_at | myotubularin related protein 6 | MTMR6 | U47635 | Hs.79877 | chr13q12 |
| 214428_x_at | complement component 4A (Rodgers blood group) /// complement component 4B (Childo blood group) | C4A | K02403 | Hs.150833 | chr6p21.3 |
| 214427_at | nucleolar protein 1, 120kDa | NOL1 | NM_006170 | Hs.15243 | chr12p13 |
| 214414_x_at | hemoglobin, alpha 2 /// hemoglobin, alpha 2 | HBA2 | T50399 | Hs.398636 | chr16p13.3 |
| 214395_x_at | Eukaryotic translation elongation factor 1 delta (guanine nucleotide exchange protein) | EEF1D | AI335509 | Hs.334798 | chr8q24.3 |
| 214390_s_at | branched chain aminotransferase 1, cytosolic | BCAT1 | AI652662 | Hs.438993 | chr12pter-q12 |
| 214378_at | tissue factor pathway inhibitor (lipoprotein-associated coagulation inhibitor) | TFPI | BF109662 | Hs.102301 | chr2q31-q32.1 |
| 214364_at | MTERF domain containing 2 | MTERFD2 | W84525 | Hs.16443 | chr2q37.3 |
| 214355_x_at | similar to CTAGE family, member 5 | LOC643854 | AW274747 | Hs.450234 | chr7q35 |
| 214336_s_at | coatomer protein complex, subunit alpha | COPA | AI621079 | Hs.162121 | chr1q23-q25 |
| 214331_at | Ts translation elongation factor, mitochondrial | TSFM | AI796813 |  |  |
| 214317_x_at | Ribosomal protein S9 | RPS9 | BE348997 | Hs.139876 | chr19q13.4 |
| 214313_s_at | Eukaryotic translation initiation factor 5B | EIF5B | BE138647 |  |  |
| 214306_at | optic atrophy 1 (autosomal dominant) | OPA1 | AA209332 | Hs.131273 | chr3q28-q29 |
| 214293_at | Septin 11 | Septin 11 | AI539361 |  |  |
| 214290_s_at | histone cluster 2, H2aa3 /// histone cluster 2, H2aa4 | HIST2H2AA3 | AI313324 | Hs.417332 | chr1q21.2 |
| 214285_at | fatty acid binding protein 3, muscle and heart (mammary-derived growth inhibitor) | FABP3 | AI041520 | Hs.534324 | chr1p33-p32 |
| 214283_at | transmembrane protein 97 | TMEM97 | AU150186 | Hs.199695 | chr17q11.2 |
| 214279_s_at | NDRG family member 2 | NDRG2 | W74452 | Hs.243960 | chr14q11.2 |
| 214277_at | COX11 homolog, cytochrome c oxidase assembly protein (yeast) /// COX11 homolog, cytochrome c oxidase assembly protein (yeast) pseudogene | COX11 | AI376724 | Hs.436988 | chr17q22 |
| 214273_x_at | chromosome 16 open reading frame 35 | C16orf35 | AV704353 | Hs.19699 | chr16p13.3 |
| 214266_s_at | PDZ and LIM domain 7 (enigma) | PDLIM7 | AW206786 | Hs.436339 | chr5q35.3 |
| 214264_s_at | chromosome 14 open reading frame 143 | C14orf143 | AI656610 | Hs.123232 | chr14q32.11 |
| 214259_s_at | aldo-keto reductase family 7, member A2 (aflatoxin aldehyde reductase) | AKR7A2 | AI144075 | Hs.512807 | chr1p35.1-p36.23 |
| 214257_s_at | SEC22 vesicle trafficking protein homolog B (S. cerevisiae) | SEC22B | AA890010 |  |  |
| 214246_x_at | misshapen-like kinase 1 (zebrafish) | MINK1 | AI859060 | Hs.313227 | chr17p13.2 |
| 214244_s_at | ATPase, H+ transporting, lysosomal 9kDa, V0 subunit e1 | ATP6V0E1 | AV717561 |  |  |
| 214230_at | cell division cycle 42 (GTP binding protein, 25kDa) | CDC42 | R37664 | Hs.355832 | chr1p36.1 |
| 214213_x_at | Lamin A/C | LMNA | AA063189 | Hs.436441 | chr1q21.2-q21.3 |
| 214201_x_at | HLA-B associated transcript 2 | BAT2 | AA742237 | Hs.436093 | chr6p21.3 |
| 214198_s_at | DiGeorge syndrome critical region gene 2 | DGCR2 | AU150824 | Hs.415359 | chr22q11.21 |
| 214197_s_at | SET domain, bifurcated 1 | SETDB1 | AI762193 | Hs.345058 | chr1q21 |
| 214196_s_at | tripeptidyl peptidase I | TPP1 | AA602532 | Hs.429658 | chr11p15 |
| 214157_at | GNAS complex locus | GNAS | AA401492 | Hs.157307 | chr20q13.2-q13.3 |
| 214156_at | myosin VIIA and Rab interacting protein | MYRIP | AL050090 | Hs.162953 | chr3p22.2-p22.1 |
| 214153_at | ELOVL family member 5, elongation of long chain fatty acids (FEN1/Elo2, SUR4/Elo3-like, yeast) | ELOVL5 | BE467941 | Hs.343667 | chr6p21.1-p12.1 |
| 214151_s_at | cell cycle progression 1 | CCPG1 | AU144243 | Hs.126115 | chr15q21-q22 |
| 214144_at | Polymerase (RNA) II (DNA directed) polypeptide D | POLR2D | BF432147 | Hs.318549 | chr2q21 |
| 214140_at | solute carrier family 25 (mitochondrial carrier; Graves disease autoantigen), member 16 | SLC25A16 | AI827990 | Hs.150534 | chr10q21.3 |
| 214132_at | ATP synthase, H+ transporting, mitochondrial F1 complex, gamma polypeptide 1 | ATP5C1 | BG232034 | Hs.155433 | chr10p15.1 |
| 214126_at | Mitochondrial carrier triple repeat 1 | MCART1 | N39314 | Hs.46791 | chr9p13.3-p12 |
| 214114_x_at | Fas-activated serine/threonine kinase | FASTK | AK023141 | Hs.75087 | chr7q35 |
| 214109_at | LPS-responsive vesicle trafficking, beach and anchor containing | LRBA | AI659561 | Hs.209846 | chr4q31.23-q31.3 |
| 214101_s_at | Hypothetical protein LOC728896 | LOC728896 | BG153399 |  |  |
| 214099_s_at | phosphodiesterase 4D interacting protein (myomegalin) /// similar to phosphodiesterase 4D interacting protein isoform 2 | PDE4DIP | AK001619 | Hs.487862 | chr1q12 |
| 214098_at | KIAA1107 | KIAA1107 | AB029030 | Hs.21554 | chr1p22.1 |
| 214097_at | ribosomal protein S21 | RPS21 | AW024383 | Hs.372960 | chr20q13.3 |
| 214095_at | serine hydroxymethyltransferase 2 (mitochondrial) | SHMT2 | AW190316 | Hs.75069 | chr12q12-q14 |
| 214093_s_at | far upstream element (FUSE) binding protein 1 | FUBP1 | AA156865 | Hs.118962 | chr1p31.1 |
| 214079_at | dehydrogenase/reductase (SDR family) member 2 | DHRS2 | AK000345 | Hs.272499 | chr14q11.2 |
| 214077_x_at | Meis1 homolog 3 (mouse) pseudogene 1 | MEIS3P1 | H15129 | Hs.356135 | chr17p12 |
| 214075_at | neuron derived neurotrophic factor | NENF | AI984136 | Hs.109494 | chr1q32.3 |
| 214074_s_at | cortactin | CTTN | BG475299 | Hs.301348 | chr11q13 |
| 214052_x_at | BAT2 domain containing 1 | BAT2D1 | AW301305 |  |  |
| 214051_at | hypothetical protein MGC39900 | MGC39900 | BF677486 | Hs.422848 | chrxq22.2 |
| 214045_at | lipoic acid synthetase | LIAS | BF056778 | Hs.534431 | chr4p14 |
| 214044_at | ryanodine receptor 2 (cardiac) | RYR2 | BE968750 | Hs.90821 | chr1q42.1-q43 |
| 214041_x_at | Ribosomal protein L37a | RPL37A | BE857772 |  |  |
| 214037_s_at | coiled-coil domain containing 22 | CCDC22 | BF224247 | Hs.26333 | chrxp11.23 |
| 214035_x_at | LOC399491 protein | LOC399491 | AA308853 | Hs.513369 | chr16p13.1 |
| 214030_at | hypothetical protein DKFZp667G2110 | DKFZp667G2110 | BE501352 | Hs.23294 | chr3q11.2 |
| 214028_x_at | tudor domain containing 3 | TDRD3 | AU156998 | Hs.112707 | chr13q21.2 |
| 214023_x_at | tubulin, beta 2B | TUBB2B | AL533838 | Hs.300701 | chr6p25 |
| 214007_s_at | twinfilin, actin-binding protein, homolog 1 (Drosophila) | TWF1 | AW665024 | Hs.189075 | chr12q12 |
| 214004_s_at | vestigial like 4 (Drosophila) | VGLL4 | AI806207 | Hs.155584 | chr3p25.3 |
| 214002_at | myosin, light chain 6, alkali, smooth muscle and non-muscle | MYL6 | AA419227 | Hs.77385 | chr12q13.2 |
| 213979_s_at | C-terminal binding protein 1 | CTBP1 | BF984434 |  |  |
| 213971_s_at | suppressor of zeste 12 homolog (Drosophila) /// suppressor of zeste 12 homolog pseudogene | SUZ12 | AI924660 | Hs.335118 | chr17q11.2 |
| 213970_at | RAB, member of RAS oncogene family-like 3 /// similar to RAB, member of RAS oncogene family-like 3 | RABL3 | AA744682 |  |  |
| 213959_s_at | KIAA1005 protein | KIAA1005 | BF515597 | Hs.196470 | chr16q12.2 |
| 213957_s_at | centrosomal protein 350kDa | CEP350 | AA635523 | Hs.413045 | chr1p36.13-q41 |
| 213954_at | KIAA0888 protein | KIAA0888 | AB020695 | Hs.91662 | chr5q13.3 |
| 213951_s_at | PSMC3 interacting protein | PSMC3IP | BE964655 | Hs.534504 | chr17q12-q21 |
| 213946_s_at | obscurin-like 1 /// similar to titin isoform N2-B /// similar to titin isoform N2-B | OBSL1 | AI633851 | Hs.6654 | chr2q35 |
| 213941_x_at | ribosomal protein S7 | RPS7 | AI970731 | Hs.444012 | chr2p25 |
| 213937_s_at | FtsJ homolog 1 (E. coli) | FTSJ1 | AV723177 | Hs.23170 | chrxp11.23 |
| 213936_x_at | surfactant, pulmonary-associated protein B | SFTPB | AW276646 | Hs.512690 | chr2p12-p11.2 |
| 213935_at | abhydrolase domain containing 5 | ABHD5 | AF007132 | Hs.19385 | chr3p21 |
| 213934_s_at | zinc finger protein 19 /// zinc finger protein 23 (KOX 16) | ZNF19 | AL567808 | Hs.376810 | chr16q22 |
| 213932_x_at | major histocompatibility complex, class I, A | HLA-A | AI923492 | Hs.181244 | chr6p21.3 |
| 213926_s_at | HIV-1 Rev binding protein | HRB | AI742626 | Hs.528387 | chr2q36.3 |
| 213922_at | tau tubulin kinase 2 | TTBK2 | AW294686 | Hs.425116 | chr15q15.2 |
| 213908_at | WAS protein homology region 2 domain containing 1-like 1 | WHDC1L1 | AI824078 | Hs.212670 | chr15q11.2 |
| 213907_at | Eukaryotic translation elongation factor 1 epsilon 1 | EEF1E1 | N32257 |  |  |
| 213901_x_at | RNA binding motif protein 9 | RBM9 | AW149379 | Hs.433574 | chr22q13.1 |
| 213897_s_at | mitochondrial ribosomal protein L23 | MRPL23 | AI832239 | Hs.3254 | chr11p15.5-p15.4 |
| 213893_x_at | postmeiotic segregation increased 2-like 5 /// similar to postmeiotic segregation increased 2-like 2 /// similar to postmeiotic segregation increased 2-like 2 /// similar to postmeiotic segregation increased 2-like 2 /// similar to postmeiotic segregation | PMS2L5 | AA161026 | Hs.397073 | chr7q11-q22 |
| 213887_s_at | polymerase (RNA) II (DNA directed) polypeptide E, 25kDa | POLR2E | AI554759 | Hs.24301 | chr19p13.3 |
| 213882_at | TM2 domain containing 1 | TM2D1 | AA012917 |  |  |
| 213878_at | similar to CG10721-PA /// similar to CG10721-PA | LOC642732 | AI685944 | Hs.192570 | chr12p12 |
| 213876_x_at | zinc finger (CCCH type), RNA-binding motif and serine/arginine rich 2 | ZRSR2 | AW089584 | Hs.171909 | chrxp22.1 |
| 213865_at | discoidin, CUB and LCCL domain containing 2 | DCBLD2 | AI378788 | Hs.173374 | chr3q12.1 |
| 213861_s_at | family with sequence similarity 119, member B | FAM119B | N67741 | Hs.528351 | chr12q14.1 |
| 213859_x_at | SWI/SNF related, matrix associated, actin dependent regulator of chromatin, subfamily a, member 5 | SMARCA5 | AI652586 | Hs.135705 | chr4q31.1-q31.2 |
| 213853_at | DPH4, JJJ3 homolog (S. cerevisiae) | DPH4 | AL050199 | Hs.266785 | chr11p13 |
| 213836_s_at | WD repeat domain, phosphoinositide interacting 1 | WIPI1 | AW052084 | Hs.9398 | chr17q24.2 |
| 213835_x_at | GTP binding protein 3 (mitochondrial) | GTPBP3 | AL524262 | Hs.334885 | chr19p13.11 |
| 213815_x_at | chromosome 19 open reading frame 29 | C19orf29 | AI913329 | Hs.128425 | chr19p13.3 |
| 213801_x_at | ribosomal protein SA /// similar to 40S ribosomal protein SA (p40) (34/67 kDa laminin receptor) (Colon carcinoma laminin-binding protein) (NEM/1CHD4) (Multidrug resistance-associated protein MGr1-Ag) /// similar to Laminin receptor 1 /// similar to 40S ri | RPSA | AW304232 | Hs.374553 | chr3p21.3 |
| 213800_at | complement factor H | CFH | X04697 | Hs.278568 | chr1q32 |
| 213799_s_at | protein tyrosine phosphatase, receptor type, A | PTPRA | BF740139 |  | chr20p13 |
| 213794_s_at | neuroguidin, EIF4E binding protein | NGDN | AI269117 | Hs.9043 | chr14q11.2 |
| 213788_s_at | FLJ35348 | FLJ35348 | AI701156 | Hs.6580 | chr9q34 |
| 213773_x_at | NOL1/NOP2/Sun domain family, member 5 | NSUN5 | AW248552 | Hs.488641 | chr7q11.23 |
| 213763_at | Homeodomain interacting protein kinase 2 | HIPK2 | R37104 | Hs.397465 | chr7q32-q34 |
| 213762_x_at | RNA binding motif protein, X-linked | RBMX | AI452524 | Hs.380118 | chrxq26 |
| 213761_at | Mdm4, transformed 3T3 cell double minute 1, p53 binding protein (mouse) | MDM1 | AW664850 | Hs.171265 | chr12q15 |
| 213748_at | tripartite motif-containing 66 | TRIM66 | AW271713 | Hs.196966 | chr11p15.4 |
| 213745_at | attractin-like 1 | ATRNL1 | AW151108 | Hs.407474 | chr10q26 |
| 213742_at | splicing factor, arginine/serine-rich 11 | SFRS11 | AW241752 | Hs.443458 | chr1p31 |
| 213738_s_at | ATP synthase, H+ transporting, mitochondrial F1 complex, alpha subunit 1, cardiac muscle | ATP5A1 | AI587323 | Hs.298280 | chr18q12-q21 |
| 213737_x_at | golgi autoantigen, golgin subfamily a, 8G /// golgi autoantigen, golgin subfamily a, 8E | GOLGA8G | AI620911 | Hs.169639 | chr15q13.1 |
| 213725_x_at | xylosyltransferase I | XYLT1 | AI693140 | Hs.22907 | chr16p13.12 |
| 213718_at | RNA binding motif protein 4 | RBM4 | BE222257 | Hs.211203 | chr11q13 |
| 213712_at | elongation of very long chain fatty acids (FEN1/Elo2, SUR4/Elo3, yeast)-like 2 | ELOVL2 | BF508639 | Hs.132105 | chr6p24.1 |
| 213709_at | basic helix-loop-helix domain containing, class B, 9 | BHLHB9 | H11725 | Hs.4276 | chrxq23 |
| 213704_at | Rab geranylgeranyltransferase, beta subunit | RABGGTB | AA129753 | Hs.78948 | chr1p31 |
| 213693_s_at | mucin 1, cell surface associated | MUC1 | AI610869 |  |  |
| 213689_x_at | Ribosomal protein L5 | RPL5 | AL137958 | Hs.469653 | chr1p22.1 |
| 213688_at | calmodulin 1 (phosphorylase kinase, delta) | CALM1 | N25325 | Hs.282410 | chr14q24-q31 |
| 213686_at | Vacuolar protein sorting 13 homolog A (S. cerevisiae) | VPS13A | AI186145 | Hs.154336 |  |
| 213677_s_at | PMS1 postmeiotic segregation increased 1 (S. cerevisiae) | PMS1 | BG434893 | Hs.111749 | chr2q31.1 |
| 213666_at | septin 6 | septin 6 | AK026589 | Hs.90998 | chrxq24 |
| 213659_at | zinc finger protein 75 (D8C6) | ZNF75 | AA209420 | Hs.131127 | chrxq26.3 |
| 213652_at | Proprotein convertase subtilisin/kexin type 5 | PCSK5 | AU152579 | Hs.288931 | chr9q21.3 |
| 213644_at | coiled-coil domain containing 46 | CCDC46 | AI979276 | Hs.500501 | chr17q24.2 |
| 213639_s_at | zinc finger protein 500 | ZNF500 | AI871396 | Hs.101414 | chr16p13.3 |
| 213632_at | dihydroorotate dehydrogenase | DHODH | M94065 | Hs.405639 | chr16q22 |
| 213630_at | KIAA0363 protein | KIAA0363 | AB002361 | Hs.96633 | chr7p13 |
| 213629_x_at | metallothionein 1F (functional) | MT1F | BF246115 | Hs.438737 | chr16q13 |
| 213625_at | zinc finger protein 307 | ZNF307 | AW190088 | Hs.44720 | chr6p21.33-p21.31 |
| 213624_at | sphingomyelin phosphodiesterase, acid-like 3A | SMPDL3A | AA873600 | Hs.277962 | chr6q22.31 |
| 213622_at | collagen, type IX, alpha 2 | COL9A2 | AI733465 | Hs.418012 | chr1p33-p32 |
| 213618_at | centaurin, delta 1 | CENTD1 | AB011152 | Hs.427719 | chr4p14 |
| 213617_s_at | chromosome 18 open reading frame 10 | C18orf10 | BF063896 | Hs.22981 | chr18q12.2 |
| 213612_x_at | neuroblastoma breakpoint family, member 15 /// neuroblastoma breakpoint family, member 10 /// neuroblastoma breakpoint family, member 8 /// similar to CG10522-PA /// hypothetical protein LOC728980 | NBPF15 | AI800419 | Hs.488862 | chr1q21.1 /// chr1q21.2 /// chr1 |
| 213610_s_at | kelch-like 23 (Drosophila) | KLHL23 | BE326381 | Hs.125465 | chr2q31.1 |
| 213608_s_at | similar to SRR1-like protein | CTB-1048E9.5 | AI220627 | Hs.517500 | chr22q12.1 |
| 213607_x_at | NAD kinase | NADK | BE551347 | Hs.220324 | chr1p36.33-p36.21 |
| 213605_s_at | Similar to Beta-glucuronidase precursor | LOC728411 | AL049987 | Hs.121081 | chr5q13.2 |
| 213593_s_at | transformer-2 alpha | TRA2A | AW978896 | Hs.445652 | chr7p15.3 |
| 213590_at | solute carrier family 16, member 5 (monocarboxylic acid transporter 6) | SLC16A5 | AA705628 | Hs.90911 | chr17q25.1 |
| 213588_x_at | ribosomal protein L14 | RPL14 | AA838274 | Hs.446522 | chr3p22-p21.2 |
| 213587_s_at | ATPase, H+ transporting V0 subunit e2 | ATP6V0E2 | AI884867 | Hs.351612 | chr7q36.1 |
| 213582_at | ATPase, Class VI, type 11A | ATP11A | BF439472 |  |  |
| 213562_s_at | squalene epoxidase | SQLE | BF979497 | Hs.71465 | chr8q24.1 |
| 213556_at | similar to R28379_1 | LOC390940 | BE673445 | Hs.22049 | chr19q13.31 |
| 213552_at | UDP-glucuronic acid epimerase | GLCE | W87398 | Hs.183006 | chr15q23 |
| 213551_x_at | polycomb group ring finger 2 | PCGF2 | AI744229 | Hs.371617 | chr17q12 |
| 213548_s_at | CDV3 homolog (mouse) | CDV3 | BG257762 | Hs.283690 | chr3q22.1 |
| 213546_at | hypothetical protein DKFZp586I1420 | DKFZp586I1420 | AL050378 | Hs.112423 | chr7p15.1 |
| 213540_at | hydroxysteroid (17-beta) dehydrogenase 8 | HSD17B8 | AL031228 | Hs.415058 | chr6p21.3 |
| 213528_at | chromosome 1 open reading frame 156 | C1orf156 | AL035369 | Hs.33922 | chr1q24.2 |
| 213527_s_at | zinc finger protein 688 | ZNF688 | AI095896 | Hs.301463 | chr16p11.2 |
| 213526_s_at | protein F25965 | F25965 | BF215644 | Hs.529100 | chr19q13.1 |
| 213524_s_at | G0/G1switch 2 | G0S2 | NM_015714 | Hs.432132 | chr1q32.2-q41 |
| 213521_at | protein tyrosine phosphatase, non-receptor type 18 (brain-derived) | PTPN18 | AW575379 |  |  |
| 213511_s_at | myotubularin related protein 1 | MTMR1 | AI167164 | Hs.347187 | chrxq28 |
| 213510_x_at | TL132 protein | LOC220594 | AW194543 | Hs.234573 | chr17p11.2 |
| 213509_x_at | carboxylesterase 2 (intestine, liver) | CES2 | AW157619 | Hs.282975 | chr16q22.1 |
| 213508_at | chromosome 14 open reading frame 147 | C14orf147 | AA142942 | Hs.353161 | chr14q13.1 |
| 213504_at | COP9 constitutive photomorphogenic homolog subunit 6 (Arabidopsis) | COPS6 | W63732 | Hs.15591 | chr7q22.1 |
| 213500_at | Coatomer protein complex, subunit beta 2 (beta prime) | COPB2 | AI307760 | Hs.14074 | chr3q23 |
| 213497_at | ankyrin repeat and BTB (POZ) domain containing 2 | ABTB2 | AL050374 | Hs.370774 | chr11p13 |
| 213490_s_at | mitogen-activated protein kinase kinase 2 | MAP2K2 | AI762811 | Hs.366546 | chr19p13.3 |
| 213489_at | Microtubule-associated protein, RP/EB family, member 2 | MAPRE2 | BE671156 |  |  |
| 213483_at | peptidylprolyl isomerase domain and WD repeat containing 1 | PPWD1 | AK025679 | Hs.1191 | chr5q12.3 |
| 213479_at | neuronal pentraxin II | NPTX2 | U26662 | Hs.3281 | chr7q21.3-q22.1 |
| 213478_at | kazrin | KIAA1026 | AB028949 | Hs.368823 | chr1p36.21 |
| 213474_at | Potassium channel tetramerisation domain containing 7 | KCTD7 | AI890903 |  |  |
| 213473_at | BRCA1 associated protein | BRAP | AL042733 | Hs.122764 | chr12q24 |
| 213470_s_at | heterogeneous nuclear ribonucleoprotein H1 (H) | HNRPH1 | BF983406 | Hs.202166 | chr5q35.3 |
| 213469_at | GPI deacylase | PGAP1 | AV705244 | Hs.287525 | chr2q33.1 |
| 213463_s_at | KIAA0974 | KIAA0974 | AW300504 | Hs.408577 | chr10q22.2 |
| 213461_at | nudix (nucleoside diphosphate linked moiety X)-type motif 21 | NUDT21 | AI800983 |  |  |
| 213457_at | malignant fibrous histiocytoma amplified sequence 1 | MFHAS1 | BF739959 | Hs.379414 | chr8p23.1 |
| 213455_at | family with sequence similarity 114, member A1 | FAM114A1 | W87466 | Hs.147096 | chr4p14 |
| 213454_at | apoptosis-inducing, TAF9-like domain 1 | APITD1 | AL578583 | Hs.412311 | chr1p36.22 |
| 213452_at | zinc finger protein 184 | ZNF184 | AI811577 | Hs.158174 | chr6p21.3 |
| 213449_at | processing of precursor 1, ribonuclease P/MRP subunit (S. cerevisiae) | POP1 | D31765 | Hs.170114 | chr8q22.1 |
| 213448_at | Metaxin 1 | MTX1 | AI693193 | Hs.247551 | chr1q21 |
| 213447_at | imprinted in Prader-Willi syndrome | IPW | AI672541 |  | chr15q11-q12 |
| 213445_at | zinc finger CCCH-type containing 3 | ZC3H3 | D63484 | Hs.370491 | chr8q24.3 |
| 213440_at | RAB1A, member RAS oncogene family | RAB1A | AL530264 | Hs.227327 | chr2p14 |
| 213437_at | RUN and FYVE domain containing 3 | RUFY3 | AA861784 | Hs.518801 | chr4q13.3 |
| 213435_at | SATB family member 2 | SATB2 | AB028957 | Hs.412327 | chr2q33 |
| 213434_at | syntaxin 2 | STX2 | H95263 | Hs.99865 | chr12q24.33 |
| 213428_s_at | collagen, type VI, alpha 1 | COL6A1 | AA292373 | Hs.415997 | chr21q22.3 |
| 213427_at | ribonuclease P 40kDa subunit | RPP40 | NM_006638 | Hs.511756 | chr6p25.1 |
| 213424_at | KIAA0895 protein | KIAA0895 | AB020702 | Hs.6224 | chr7p14.1 |
| 213419_at | amyloid beta (A4) precursor protein-binding, family B, member 2 (Fe65-like) | APBB2 | U62325 | Hs.324125 | chr4p14 |
| 213413_at | stonin 1 | STON1 | BG434174 | Hs.44385 | chr2p16.3 |
| 213410_at | chromosome 10 open reading frame 137 | C10orf137 | AL050102 | Hs.355929 | chr10q26.13-q26.2 |
| 213409_s_at | Ras homolog enriched in brain | RHEB | BF593727 | Hs.279903 | chr7q36 |
| 213407_at | PH domain and leucine rich repeat protein phosphatase-like | PHLPPL | AB023148 | Hs.461244 | chr16q22.2 |
| 213405_at | RAB22A, member RAS oncogene family | RAB22A | N95443 | Hs.281117 | chr20q13.32 |
| 213402_at | zinc finger protein 787 | ZNF787 | AA029811 | Hs.397153 | chr19q13.43 |
| 213401_s_at | Transducin (beta)-like 1X-linked | TBL1X | AA724134 |  |  |
| 213398_s_at | chromosome 14 open reading frame 124 | C14orf124 | AI347090 | Hs.283111 | chr14q11.2 |
| 213392_at | IQ motif containing K | IQCK | AW070229 | Hs.306382 | chr16p12.3 |
| 213391_at | dpy-19-like 4 (C. elegans) | DPY19L4 | AI669947 | Hs.165539 | chr8q22.1 |
| 213390_at | chromosome 19 open reading frame 7 | C19orf7 | AB028987 | Hs.377499 | chr19q13.32 |
| 213388_at | similar to phosphodiesterase 4D interacting protein isoform 2 | LOC727942 | H15535 | Hs.487862 | chr1q12 |
| 213387_at | ATPase family, AAA domain containing 2B | ATAD2B | AB033066 | Hs.62576 | chr2p24.1-p23.3 |
| 213379_at | coenzyme Q2 homolog, prenyltransferase (yeast) | COQ2 | AF091086 | Hs.144304 | chr4q21.23 |
| 213377_x_at | ribosomal protein S12 | RPS12 | AI799007 | Hs.380956 | chr6q23.2 |
| 213376_at | zinc finger and BTB domain containing 1 | ZBTB1 | AI656706 | Hs.400802 | chr14q23.3 |
| 213375_s_at | hypothetical gene CG018 | CG018 | N80918 | Hs.277888 | chr13q12-q13 |
| 213373_s_at | caspase 8, apoptosis-related cysteine peptidase | CASP8 | BF439983 | Hs.430124 | chr2q33-q34 |
| 213372_at | progestin and adipoQ receptor family member III | PAQR3 | AW173157 | Hs.369679 | chr4q21.21 |
| 213365_at | exonuclease domain containing 1 | EXOD1 | N64622 | Hs.248437 | chr16p12.3 |
| 213364_s_at | sorting nexin 1 | SNX1 | AI052536 | Hs.498154 | chr15q22.31 |
| 213361_at | tudor domain containing 7 | TDRD7 | AW129593 | Hs.416543 | chr9q22.33 |
| 213358_at | KIAA0802 /// chromosome 21 open reading frame 57 | KIAA0802 | AB018345 | Hs.127716 | chr18p11.22 |
| 213357_at | general transcription factor IIH, polypeptide 5 | GTF2H5 | AV701318 | Hs.356224 | chr6q25.3 |
| 213353_at | ATP-binding cassette, sub-family A (ABC1), member 5 | ABCA5 | BF693921 | Hs.421474 | chr17q24.3 |
| 213352_at | transmembrane and coiled-coil domain family 1 | TMCC1 | AB018322 | Hs.362996 | chr3q21.3 |
| 213348_at | Cyclin-dependent kinase inhibitor 1C (p57, Kip2) | CDKN1C | N33167 | Hs.106070 | chr11p15.5 |
| 213346_at | chromosome 13 open reading frame 27 | C13orf27 | BE748563 | Hs.398111 | chr13q33.1 |
| 213344_s_at | H2A histone family, member X | H2AFX | H51429 |  |  |
| 213342_at | Yes-associated protein 1, 65kDa | YAP1 | AI745185 | Hs.170548 | chr11q13 |
| 213341_at | fem-1 homolog c (C. elegans) | FEM1C | AI862658 | Hs.47367 | chr5q22 |
| 213340_s_at | KIAA0495 | KIAA0495 | AI073551 | Hs.49658 | chr1p36.32 |
| 213333_at | malate dehydrogenase 2, NAD (mitochondrial) | MDH2 | AL520774 | Hs.405860 | chr7p12.3-q11.2 |
| 213327_s_at | ubiquitin specific peptidase 12 | USP12 | AI820101 |  |  |
| 213325_at | poliovirus receptor-related 3 | PVRL3 | AA129716 | Hs.436602 | chr3q13 |
| 213323_s_at | zinc finger CCCH-type containing 7B | ZC3H7B | BE855831 | Hs.25347 | chr22q13.2 |
| 213322_at | chromosome 6 open reading frame 130 | C6orf130 | AL031778 | Hs.227457 | chr6p21.1 |
| 213320_at | protein arginine methyltransferase 3 | PRMT3 | AL551971 | Hs.152337 | chr11p15.1 |
| 213314_at | chromosome 6 open reading frame 162 | C6orf162 | NM_020425 | Hs.70769 | chr6q15-q16.1 |
| 213311_s_at | transcription factor 25 (basic helix-loop-helix) /// matrix-remodelling associated 7 | TCF25 | BF000251 | Hs.227835 | chr16q24.3 |
| 213306_at | multiple PDZ domain protein | MPDZ | AA917899 | Hs.169378 | chr9p24-p22 |
| 213305_s_at | protein phosphatase 2, regulatory subunit B (B56), gamma isoform | PPP2R5C | L42375 | Hs.249955 | chr14q32 |
| 213304_at | KIAA0423 | KIAA0423 | AI823592 | Hs.111373 | chr14q21.3 |
| 213302_at | phosphoribosylformylglycinamidine synthase (FGAR amidotransferase) | PFAS | AL044326 | Hs.88139 | chr17p13.1 |
| 213300_at | hypothetical protein LOC23130 | KIAA0404 | AW168132 | Hs.370671 | chr11q13.1 |
| 213296_at | RER1 retention in endoplasmic reticulum 1 homolog (S. cerevisiae) | RER1 | BF339133 |  |  |
| 213293_s_at | tripartite motif-containing 22 | TRIM22 | AA083478 | Hs.318501 | chr11p15 |
| 213290_at | collagen, type VI, alpha 2 | COL6A2 | AL531750 | Hs.420269 | chr21q22.3 |
| 213289_at | Spermidine/spermine N1-acetyl transferase-like 1 | SATL1 | BE221922 |  |  |
| 213286_at | zinc finger RNA binding protein | ZFR | BF445199 | Hs.173518 | chr5p13.3 |
| 213283_s_at | sal-like 2 (Drosophila) | SALL2 | BG285616 | Hs.416358 | chr14q11.1-q12 |
| 213281_at | Jun oncogene | JUN | BE327172 |  |  |
| 213279_at | dehydrogenase/reductase (SDR family) member 1 | DHRS1 | AL050217 | Hs.348350 | chr14q11.2 |
| 213278_at | myotubularin related protein 9 | MTMR9 | AW014788 | Hs.447785 | chr8p23-p22 |
| 213272_s_at | transmembrane protein 159 | TMEM159 | AF070596 | Hs.27191 | chr16p12 |
| 213263_s_at | poly(rC) binding protein 2 | PCBP2 | AW025150 | Hs.132977 | chr12q13.12-q13.13 |
| 213262_at | spastic ataxia of Charlevoix-Saguenay (sacsin) | SACS | AI932370 | Hs.159492 | chr13q12 |
| 213261_at | lupus brain antigen 1 | LBA1 | AA035414 | Hs.517761 | chr3p22.3 |
| 213259_s_at | sterile alpha and TIR motif containing 1 | SARM1 | AI937297 | Hs.446689 | chr17q11 |
| 213256_at | membrane-associated ring finger (C3HC4) 3 | 03-mars | AW593996 | Hs.124969 | chr5q23.2 |
| 213252_at | SH3 and PX domains 2A | SH3PXD2A | AI739005 | Hs.146233 | chr10q25.1 |
| 213248_at | hypothetical protein LOC221362 /// similar to heterogeneous nuclear ribonucleoprotein A/B | LOC221362 | AL577024 | Hs.7921 | chr6p12.3 |
| 213246_at | chromosome 14 open reading frame 109 | C14orf109 | AI346504 | Hs.275352 | chr14q32.13 |
| 213241_at | plexin C1 | PLXNC1 | AF035307 | Hs.286229 | chr12q23.3 |
| 213239_at | chromosome 13 open reading frame 24 | C13orf24 | NM_006346 | Hs.43913 | chr13q22.1 |
| 213238_at | ATPase, Class V, type 10D | ATP10D | AI478147 | Hs.437241 | chr4p12 |
| 213235_at | similar to TSG118.1 | LOC400506 | BE504197 | Hs.129061 | chr16p12.3 |
| 213226_at | cyclin A2 | CCNA2 | AI346350 | Hs.85137 | chr4q25-q31 |
| 213225_at | protein phosphatase 1B (formerly 2C), magnesium-dependent, beta isoform | PPM1B | AJ271832 | Hs.370284 | chr2p21 |
| 213223_at | ribosomal protein L28 | RPL28 | AK025866 | Hs.356371 | chr19q13.4 |
| 213222_at | phospholipase C, beta 1 (phosphoinositide-specific) | PLCB1 | AL049593 | Hs.429643 | chr20p12 |
| 213221_s_at | SNF1-like kinase 2 | SNF1LK2 | AB018324 | Hs.244981 | chr11q23.1 |
| 213220_at | hypothetical protein LOC92482 | LOC92482 | AV706096 | Hs.192249 | chr10q25.2 |
| 213218_at | zinc finger protein 187 | ZNF187 | AV705032 | Hs.237786 | chr6p21.31 |
| 213216_at | OTU domain containing 3 | OTUD3 | AL537463 | Hs.374987 | chr1p36.13 |
| 213213_at | death inducer-obliterator 1 | DIDO1 | AL035669 | Hs.504960 | chr20q13.33 |
| 213212_x_at | hypothetical protein LOC161527 /// golgi autoantigen, golgin subfamily a-like pseudogene /// similar to golgi autoantigen, golgin subfamily a-like /// similar to golgi autoantigen, golgin subfamily a-like /// similar to golgi autoantigen, golgin subfamily | LOC161527 | AI632181 | Hs.459133 | chr15q25.2 |
| 213208_at | KIAA0240 | KIAA0240 | AI801951 | Hs.196275 | chr6p21.1 |
| 213205_s_at | RAD54-like 2 (S. cerevisiae) | RAD54L2 | AU159543 | Hs.105399 | chr3p21.2 |
| 213203_at | Small nuclear RNA activating complex, polypeptide 5, 19kDa | SNAPC5 | AI633709 | Hs.30174 | chr15q22.31 |
| 213197_at | astrotactin 1 | ASTN1 | AB006627 | Hs.6788 | chr1q25.2 |
| 213196_at | zinc finger protein 629 | ZNF629 | AI924293 | Hs.301094 | chr16p11.2 |
| 213194_at | roundabout, axon guidance receptor, homolog 1 (Drosophila) | ROBO1 | BF059159 | Hs.301198 | chr3p12 |
| 213191_at | toll-like receptor adaptor molecule 1 | TICAM1 | AF070530 | Hs.29344 | chr19p13.3 |
| 213190_at | component of oligomeric golgi complex 7 | COG7 | R61519 | Hs.185807 | chr16p12.1 |
| 213188_s_at | MYC induced nuclear antigen | MINA | AI823896 | Hs.476972 | chr3q11.2 |
| 213180_s_at | golgi SNAP receptor complex member 2 | GOSR2 | BE895285 | Hs.432552 | chr17q21 |
| 213178_s_at | mitogen-activated protein kinase 8 interacting protein 3 | MAPK8IP3 | AB028989 | Hs.207763 | chr16p13.3 |
| 213173_at | pecanex homolog (Drosophila) | PCNX | AI815033 | Hs.55947 | chr14q24.2 |
| 213172_at | tetratricopeptide repeat domain 9 | TTC9 | AW235608 | Hs.79170 | chr14q24.2 |
| 213170_at | glutathione peroxidase 7 | GPX7 | AA406605 | Hs.43728 | chr1p32 |
| 213161_at | chromosome 9 open reading frame 97 /// chromosome 9 open reading frame 97 | C9orf97 | AI583393 | Hs.279062 | chr9q22.33 |
| 213153_at | SET domain containing 1B | SETD1B | AB028999 | Hs.154525 | chr12q24.31 |
| 213151_s_at | septin 7 | septin 7 | AU157515 | Hs.396503 | chr7p14.3-p14.1 |
| 213145_at | F-box and leucine-rich repeat protein 14 | FBXL14 | BF001666 | Hs.367956 | chr12p13.33 |
| 213141_at | protein serine kinase H1 | PSKH1 | AJ272212 |  |  |
| 213140_s_at | synovial sarcoma translocation gene on chromosome 18-like 1 | SS18L1 | AB014593 | Hs.154429 | chr20q13.3 |
| 213139_at | snail homolog 2 (Drosophila) | SNAI2 | AI572079 | Hs.360174 | chr8q11 |
| 213137_s_at | protein tyrosine phosphatase, non-receptor type 2 | PTPN2 | AI828880 | Hs.255158 | chr18p11.3-p11.2 |
| 213132_s_at | malonyl CoA:ACP acyltransferase (mitochondrial) | MCAT | AL022237 | Hs.349111 | chr22q13.31 |
| 213129_s_at | glycine cleavage system protein H (aminomethyl carrier) /// similar to Glycine cleavage system H protein, mitochondrial precursor /// similar to Glycine cleavage system H protein, mitochondrial precursor | GCSH | AI970157 | Hs.513762 | chr16q23.2 |
| 213128_s_at | ubiquitin protein ligase E3A (human papilloma virus E6-associated protein, Angelman syndrome) | UBE3A | AA527499 | Hs.180686 | chr15q11-q13 |
| 213127_s_at | mediator of RNA polymerase II transcription, subunit 8 homolog (S. cerevisiae) | MED8 | BG230758 | Hs.301756 | chr1p34.2 |
| 213125_at | olfactomedin-like 2B | OLFML2B | AW007573 | Hs.43658 | chr1q23.3 |
| 213122_at | TSPY-like 5 | TSPYL5 | AI096375 | Hs.173094 | chr8q22.1 |
| 213119_at | solute carrier family 36 (proton/amino acid symporter), member 1 | SLC36A1 | AW058600 | Hs.409314 | chr5q33.1 |
| 213118_at | KIAA0701 protein | KIAA0701 | AL136821 | Hs.153293 | chr12q23.1 |
| 213117_at | kelch-like 9 (Drosophila) | KLHL9 | AW138594 | Hs.147717 | chr9p22 |
| 213115_at | ATG4 autophagy related 4 homolog A (S. cerevisiae) | ATG4A | AL031177 | Hs.496554 | chrxq22.1-q22.3 |
| 213111_at | phosphatidylinositol-3-phosphate/phosphatidylinositol 5-kinase, type III | PIP5K3 | AB023198 | Hs.158135 | chr2q34 |
| 213105_s_at | chromosome 16 open reading frame 42 | C16orf42 | AI799802 | Hs.134846 | chr16p13.3 |
| 213103_at | START domain containing 13 | STARD13 | AA128023 | Hs.369912 | chr13q12-q13 |
| 213097_s_at | zuotin related factor 1 | ZRF1 | AI338837 | Hs.396817 | chr7q22-q32 |
| 213092_x_at | DnaJ (Hsp40) homolog, subfamily C, member 9 | DNAJC9 | AW241779 | Hs.400676 | chr10q22.2 |
| 213090_s_at | TAF4 RNA polymerase II, TATA box binding protein (TBP)-associated factor, 135kDa | TAF4 | AI744029 | Hs.24644 | chr20q13.33 |
| 213085_s_at | WW and C2 domain containing 1 | WWC1 | AB020676 | Hs.434243 | chr5q34 |
| 213079_at | TSR2, 20S rRNA accumulation, homolog (S. cerevisiae) | TSR2 | AA223871 | Hs.104349 | chrxp11.22 |
| 213076_at | inositol 1,4,5-trisphosphate 3-kinase C | ITPKC | D38169 | Hs.21453 | chr19q13.1 |
| 213073_at | zinc finger, FYVE domain containing 26 | ZFYVE26 | AB002319 | Hs.8663 | chr14q24.1 |
| 213067_at | myosin, heavy chain 10, non-muscle | MYH10 | AI382123 | Hs.280311 | chr17p13 |
| 213062_at | N-terminal asparagine amidase | NTAN1 | AA643304 | Hs.534637 | chr16p13.11 |
| 213058_at | tetratricopeptide repeat domain 28 | TTC28 | AL033538 | Hs.348799 |  |
| 213056_at | FERM domain containing 4B | FRMD4B | AU145019 | Hs.158867 | chr3p14.1 |
| 213052_at | Protein kinase, cAMP-dependent, regulatory, type II, alpha | PRKAR2A | BF246917 | Hs.440404 | chr3p21.3-p21.2 |
| 213046_at | poly(A) binding protein, nuclear 1 | PABPN1 | AI130920 | Hs.117176 | chr14q11.2-q13 |
| 213043_s_at | thyroid hormone receptor associated protein 4 | THRAP4 | AI023317 | Hs.23106 | chr17q21.1 |
| 213039_at | rho/rac guanine nucleotide exchange factor (GEF) 18 | ARHGEF18 | AB011093 | Hs.6150 | chr19p13.3 |
| 213038_at | IBR domain containing 3 | IBRDC3 | AL031602 | Hs.128366 | chr1p35.1 |
| 213036_x_at | ATPase, Ca++ transporting, ubiquitous | ATP2A3 | Y15724 | Hs.5541 | chr17p13.3 |
| 213035_at | ankyrin repeat domain 28 | ANKRD28 | AI081194 | Hs.273104 | chr3p25.1 |
| 213031_s_at | WD repeat domain 73 | WDR73 | AF161382 | Hs.29863 | chr15q25.2 |
| 213028_at | nuclear factor related to kappaB binding protein | NFRKB | AI887378 | Hs.149466 | chr11q24-q25 |
| 213025_at | THUMP domain containing 1 | THUMPD1 | AL134904 | Hs.268371 | chr16p12.3 |
| 213024_at | TATA element modulatory factor 1 | TMF1 | BF593908 | Hs.267632 | chr3p21-p12 |
| 213020_at | golgi SNAP receptor complex member 1 | GOSR1 | AI814252 | Hs.124436 | chr17q11 |
| 213019_at | RAN binding protein 6 | RANBP6 | AI123233 | Hs.167496 | chr9p24.1 |
| 213017_at | abhydrolase domain containing 3 | ABHD3 | AL534702 | Hs.464526 | chr18q11.2 |
| 213012_at | neural precursor cell expressed, developmentally down-regulated 4 | NEDD4 | D42055 | Hs.1565 | chr15q |
| 213010_at | protein kinase C, delta binding protein | PRKCDBP | AI088622 | Hs.434044 | chr11p15.4 |
| 213009_s_at | tripartite motif-containing 37 | TRIM37 | AK022701 | Hs.8164 | chr17q23.2 |
| 213006_at | CCAAT/enhancer binding protein (C/EBP), delta | CEBPD | AV655640 | Hs.442156 | chr8p11.2-p11.1 |
| 213005_s_at | ankyrin repeat domain 15 | ANKRD15 | D79994 | Hs.77546 | chr9p24.3 |
| 213000_at | MORC family CW-type zinc finger 3 | MORC3 | AP000693 | Hs.421150 | chr21q22.13 |
| 212996_s_at | URB1 ribosome biogenesis 1 homolog (S. cerevisiae) | URB1 | AI803485 | Hs.535819 | chr21q22.11 |
| 212990_at | synaptojanin 1 | SYNJ1 | AB020717 | Hs.401730 | chr21q22.2 |
| 212989_at | transmembrane protein 23 | TMEM23 | AI377497 | Hs.156935 | chr10q11.2 |
| 212986_s_at | tousled-like kinase 2 | TLK2 | BF112255 | Hs.445078 | chr17q23 |
| 212983_at | v-Ha-ras Harvey rat sarcoma viral oncogene homolog | HRAS | NM_005343 | Hs.37003 | chr11p15.5 |
| 212982_at | zinc finger, DHHC-type containing 17 | ZDHHC17 | AI621223 | Hs.4014 | chr12q21.2 |
| 212978_at | Leucine rich repeat containing 8 family, member B | LRRC8B | AU146004 | Hs.387915 | chr1p22.2 |
| 212977_at | chemokine (C-X-C motif) receptor 7 | CXCR7 | AI817041 | Hs.231853 | chr2q37.3 |
| 212973_at | ribose 5-phosphate isomerase A (ribose 5-phosphate epimerase) | RPIA | AI692341 | Hs.79886 | chr2p11.2 |
| 212968_at | RFNG O-fucosylpeptide 3-beta-N-acetylglucosaminyltransferase | RFNG | BF940276 | Hs.12862 | chr17q25 |
| 212964_at | hypermethylated in cancer 2 | HIC2 | AI912206 | Hs.109445 | chr22q11.21 |
| 212961_x_at | chromosome X open reading frame 40B | CXorf40B | L43578 | Hs.534613 | chrxq28 |
| 212960_at | TBC1 domain family, member 9 (with GRAM domain) | TBC1D9 | BE646554 | Hs.411317 | chr4q31.21 |
| 212948_at | calmodulin binding transcription activator 2 | CAMTA2 | AB020716 | Hs.107362 | chr17p13.2 |
| 212946_at | KIAA0564 protein | RP11-125A7.3 | AK025432 | Hs.405457 | chr13q14.11 |
| 212945_s_at | MAX gene associated | MGA | BE502432 | Hs.435961 | chr15q14 |
| 212934_at | hypothetical protein LOC137886 | LOC137886 | AI245523 | Hs.155572 | chr8q12.1 |
| 212932_at | RAB3 GTPase activating protein subunit 1 (catalytic) | RAB3GAP1 | AK022494 | Hs.306327 | chr2q21.3 |
| 212930_at | ATPase, Ca++ transporting, plasma membrane 1 | ATP2B1 | AW576457 | Hs.20952 | chr12q21.3 |
| 212929_s_at | family with sequence similarity 21, member B /// family with sequence similarity 21, member C /// similar to KIAA0592 protein /// hypothetical LOC653450 | FAM21B | W68158 | Hs.375174 | chr10q11.23 /// chr10q11.22 |
| 212928_at | TSPY-like 4 | TSPYL4 | AL050331 | Hs.284141 | chr6q22.1 |
| 212927_at | Structural maintenance of chromosomes 5 | SMC5 | AB011166 | Hs.425121 | chr9q21.11 |
| 212923_s_at | chromosome 6 open reading frame 145 | C6orf145 | AK024828 | Hs.69388 | chr6p25.2 |
| 212922_s_at | SET and MYND domain containing 2 | SMYD2 | AI809870 | Hs.66170 | chr1q41 |
| 212916_at | similar to PHD finger protein 8 | LOC731069 | AW249934 | Hs.133352 | chrxp11.22 |
| 212915_at | PDZ domain containing RING finger 3 | PDZRN3 | AL569804 | Hs.177635 | chr3p13 |
| 212912_at | ribosomal protein S6 kinase, 90kDa, polypeptide 2 | RPS6KA2 | AI992251 | Hs.301664 | chr6q27 |
| 212910_at | THAP domain containing 11 | THAP11 | W19873 | Hs.82508 | chr16q22.1 |
| 212908_at | DnaJ (Hsp40) homolog, subfamily C, member 16 | DNAJC16 | AK022530 | Hs.534445 | chr1p36.1 |
| 212906_at | GRAM domain containing 1B | GRAMD1B | BE044440 | Hs.347534 | chr11q24.1 |
| 212904_at | leucine rich repeat containing 47 | LRRC47 | AB033011 | Hs.268488 | chr1p36.32 |
| 212901_s_at | cleavage stimulation factor, 3' pre-RNA, subunit 2, 64kDa, tau variant | CSTF2T | BF732638 | Hs.21992 | chr10q11 |
| 212898_at | KIAA0406 | KIAA0406 | AB007866 | Hs.410618 | chr20q11.23 |
| 212897_at | cell division cycle 2-like 6 (CDK8-like) | CDC2L6 | AI738802 | Hs.129836 | chr6q21 |
| 212896_at | superkiller viralicidic activity 2-like 2 (S. cerevisiae) | SKIV2L2 | D29641 | Hs.274531 | chr5q11.2 |
| 212895_s_at | active BCR-related gene | ABR | AL527773 | Hs.434004 | chr17p13.3 |
| 212894_at | suppressor of var1, 3-like 1 (S. cerevisiae) | SUPV3L1 | NM_003171 | Hs.106469 | chr10q22.1 |
| 212891_s_at | growth arrest and DNA-damage-inducible, gamma interacting protein 1 | GADD45GIP1 | BF972185 | Hs.83135 | chr19p13.13 |
| 212890_at | hypothetical protein MGC15523 | MGC15523 | BF663461 | Hs.352240 | chr17q25.3 |
| 212885_at | M-phase phosphoprotein 10 (U3 small nucleolar ribonucleoprotein) | MPHOSPH10 | AL545921 | Hs.201676 | chr2p13.3 |
| 212882_at | kelch-like 18 (Drosophila) | KLHL18 | AB018338 | Hs.22926 | chr3p21.31 |
| 212881_at | protein inhibitor of activated STAT, 4 | PIAS4 | AK022481 |  |  |
| 212880_at | WD repeat domain 7 | WDR7 | AB011113 | Hs.428344 | chr18q21.1-q22 |
| 212877_at | kinesin 2 | KNS2 | AA284075 | Hs.20107 | chr14q32.3 |
| 212876_at | UDP-Gal:betaGlcNAc beta 1,4- galactosyltransferase, polypeptide 4 | B4GALT4 | BF223021 | Hs.13225 | chr3q13.3 |
| 212875_s_at | chromosome 21 open reading frame 25 | C21orf25 | AP001745 | Hs.16007 | chr21q22.3 |
| 212872_s_at | Trf (TATA binding protein-related factor)-proximal homolog (Drosophila) | TRFP | AK023092 | Hs.278434 | chr6p21.1 |
| 212871_at | mitogen-activated protein kinase-activated protein kinase 5 | MAPKAPK5 | NM_003668 | Hs.413901 | chr12q24.12 |
| 212870_at | Ras association (RalGDS/AF-6) domain family 3 | RASSF3 | AI628605 | Hs.346527 | chr12q14.2 |
| 212866_at | R3H domain and coiled-coil containing 1 | R3HCC1 | AI081543 | Hs.300861 | chr8p21.3 |
| 212864_at | CDP-diacylglycerol synthase (phosphatidate cytidylyltransferase) 2 | CDS2 | Y16521 | Hs.306912 | chr20p13 |
| 212861_at | major facilitator superfamily domain containing 5 | MFSD5 | BF690150 | Hs.19210 | chr12q13.13 |
| 212860_at | zinc finger, DHHC-type containing 18 | ZDHHC18 | BG168720 | Hs.19066 | chr1p36.11 |
| 212859_x_at | metallothionein 1E (functional) | MT1E | BF217861 | Hs.418241 | chr16q13 |
| 212857_x_at | SUB1 homolog (S. cerevisiae) | SUB1 | BG231551 | Hs.229641 | chr5p13.3 |
| 212855_at | DCN1, defective in cullin neddylation 1, domain containing 4 (S. cerevisiae) | DCUN1D4 | D87466 | Hs.343513 | chr4q11 |
| 212854_x_at | neuroblastoma breakpoint family, member 10 | NBPF10 | AB051480 | Hs.502564 | chr1q21.2 |
| 212846_at | KIAA0179 | KIAA0179 | AA811192 | Hs.129621 | chr21q22.3 |
| 212843_at | neural cell adhesion molecule 1 | NCAM1 | AA126505 | Hs.78792 | chr11q23.1 |
| 212840_at | UBX domain containing 7 | UBXD7 | BG339560 | Hs.127287 | chr3q29 |
| 212838_at | dynamin binding protein | DNMBP | AB023227 | Hs.429994 | chr10q24.2 |
| 212837_at | KIAA0157 | KIAA0157 | D63877 | Hs.82324 | chr10q26.13 |
| 212836_at | polymerase (DNA-directed), delta 3, accessory subunit | POLD3 | D26018 | Hs.82502 | chr11q14 |
| 212834_at | DEAD (Asp-Glu-Ala-Asp) box polypeptide 52 | DDX52 | BE963238 | Hs.380430 | chr17q21.1 |
| 212833_at | solute carrier family 25, member 46 | SLC25A46 | M74089 | Hs.75639 | chr5q22.1 |
| 212830_at | multiple EGF-like-domains 9 | MEGF9 | W68084 | Hs.236216 | chr9q32-q33.3 |
| 212828_at | synaptojanin 2 | SYNJ2 | AA191573 | Hs.434494 | chr6q25.3 |
| 212825_at | PAX interacting (with transcription-activation domain) protein 1 | PAXIP1 | AI357401 | Hs.443881 | chr7q36 |
| 212824_at | far upstream element (FUSE) binding protein 3 | FUBP3 | U69127 | Hs.98751 | chr9q34.2 |
| 212822_at | HEG homolog 1 (zebrafish) | HEG1 | AA121502 | Hs.433452 | chr3q21.2 |
| 212820_at | Dmx-like 2 | DMXL2 | AB020663 | Hs.200828 | chr15q21.2 |
| 212817_at | DnaJ (Hsp40) homolog, subfamily B, member 5 | DNAJB5 | AK023253 | Hs.237506 | chr9p13.3 |
| 212816_s_at | cystathionine-beta-synthase | CBS | BE613178 | Hs.171003 | chr21q22.3 |
| 212814_at | adenosylhomocysteinase 3 | KIAA0828 | AB020635 | Hs.356823 | chr7q32.3 |
| 212813_at | junctional adhesion molecule 3 | JAM3 | AA149644 | Hs.419149 | chr11q25 |
| 212812_at | Serine incorporator 5 | SERINC5 | AI700633 | Hs.288232 |  |
| 212810_s_at | solute carrier family 1 (glutamate/neutral amino acid transporter), member 4 | SLC1A4 | W72527 | Hs.153307 | chr2p15-p13 |
| 212808_at | nuclear factor of activated T-cells, cytoplasmic, calcineurin-dependent 2 interacting protein | NFATC2IP | AI884627 | Hs.334788 | chr16p11.2 |
| 212807_s_at | sortilin 1 | SORT1 | BF447105 | Hs.394609 | chr1p21.3-p13.1 |
| 212805_at | KIAA0367 | KIAA0367 | AB002365 | Hs.23311 | chr9q21.2 |
| 212804_s_at | GTPase activating protein and VPS9 domains 1 | GAPVD1 | AI797397 | Hs.287266 | chr9q33.3 |
| 212803_at | NGFI-A binding protein 2 (EGR1 binding protein 2) | NAB2 | BF337329 | Hs.159223 | chr12q13.3-q14.1 |
| 212798_s_at | ankyrin repeat and MYND domain containing 2 | ANKMY2 | AK001389 | Hs.112605 | chr7p21 |
| 212796_s_at | TBC1 domain family, member 2B | TBC1D2B | BF195608 | Hs.166571 | chr15q24.3-q25.1 |
| 212791_at | hypothetical protein FLJ38984 | FLJ38984 | AL042729 | Hs.112023 | chr1p34.3 |
| 212788_x_at | ferritin, light polypeptide | FTL | BG537190 | Hs.433670 | chr19q13.3-q13.4 |
| 212787_at | YLP motif containing 1 | YLPM1 | AI952986 | Hs.303775 | chr14q24.3 |
| 212786_at | KIAA0350 | KIAA0350 | AA731693 | Hs.380599 | chr16p13.2 |
| 212784_at | capicua homolog (Drosophila) | CIC | AB002304 | Hs.388236 | chr19q13.2 |
| 212782_x_at | polymerase (RNA) II (DNA directed) polypeptide J, 13.3kDa | POLR2J | BG335629 | Hs.533383 | chr7q11.2 |
| 212781_at | retinoblastoma binding protein 6 | RBBP6 | AK026954 | Hs.188553 | chr16p12.2 |
| 212780_at | son of sevenless homolog 1 (Drosophila) | SOS1 | AA700167 | Hs.326392 | chr2p22-p21 |
| 212779_at | KIAA1109 | KIAA1109 | AB029032 | Hs.408142 | chr4q27 |
| 212774_at | zinc finger protein 238 | ZNF238 | AJ223321 | Hs.408099 | chr1q44-qter |
| 212771_at | chromosome 10 open reading frame 38 | C10orf38 | AU150943 | Hs.66762 | chr10p13 |
| 212767_at | mitochondrial GTPase 1 homolog (S. cerevisiae) | MTG1 | BC004409 | Hs.449641 | chr10q26.3 |
| 212766_s_at | interferon stimulated exonuclease gene 20kDa-like 2 | ISG20L2 | AW294587 | Hs.301904 | chr1q23.1 |
| 212759_s_at | transcription factor 7-like 2 (T-cell specific, HMG-box) | TCF7L2 | AI703074 | Hs.214039 | chr10q25.3 |
| 212757_s_at | calcium/calmodulin-dependent protein kinase (CaM kinase) II gamma | CAMK2G | BF111268 | Hs.12436 | chr10q22 |
| 212756_s_at | ubiquitin protein ligase E3 component n-recognin 2 | UBR2 | AI761518 | Hs.131842 | chr6p21.1 |
| 212754_s_at | MON2 homolog (S. cerevisiae) | MON2 | AI760249 | Hs.9846 | chr12q14.1 |
| 212752_at | cytoplasmic linker associated protein 1 | CLASP1 | AA176798 | Hs.436937 | chr2q14.2-q14.3 |
| 212751_at | ubiquitin-conjugating enzyme E2N (UBC13 homolog, yeast) | UBE2N | BG290646 | Hs.458359 | chr12q22 |
| 212748_at | megakaryoblastic leukemia (translocation) 1 | MKL1 | AB037859 | Hs.416266 | chr22q13 |
| 212747_at | ankyrin repeat and sterile alpha motif domain containing 1A | ANKS1A | AI990523 | Hs.435285 | chr6p21.31 |
| 212746_s_at | centrosomal protein 170kDa | CEP170 | AA126789 | Hs.25132 | chr1q44 |
| 212744_at | Bardet-Biedl syndrome 4 | BBS4 | AI813772 | Hs.26471 | chr15q22.3-q23 |
| 212742_at | zinc finger protein 364 | ZNF364 | AL530462 | Hs.16537 | chr1q21.1 |
| 212740_at | phosphoinositide-3-kinase, regulatory subunit 4, p150 | PIK3R4 | BF740111 | Hs.306747 | chr3q21.3 |
| 212739_s_at | non-metastatic cells 4, protein expressed in | NME4 | AL523860 | Hs.9235 | chr16p13.3 |
| 212736_at | chromosome 16 open reading frame 45 | C16orf45 | BE299456 | Hs.148258 | chr16p13.11 |
| 212735_at | KIAA0226 | KIAA0226 | BF448041 | Hs.499355 | chr3q29 |
| 212731_at | ankyrin repeat domain 46 | ANKRD46 | U79297 | Hs.11506 | chr8q22.3 |
| 212730_at | desmuslin | DMN | AK026420 | Hs.381347 | chr15q26.3 |
| 212729_at | discs, large homolog 3 (neuroendocrine-dlg, Drosophila) | DLG3 | AI916274 | Hs.11101 | chrxq13.1 |
| 212726_at | PHD finger protein 2 | PHF2 | AB014562 | Hs.93868 | chr9q22.31 |
| 212724_at | Rho family GTPase 3 | RND3 | BG054844 | Hs.6838 | chr2q23.3 |
| 212722_s_at | phosphatidylserine receptor | PTDSR | AK021780 | Hs.72660 | chr17q25 |
| 212720_at | poly(A) polymerase alpha | PAPOLA | AI670847 | Hs.201085 | chr14q32.31 |
| 212719_at | PH domain and leucine rich repeat protein phosphatase | PHLPP | AB011178 | Hs.38176 | chr18q21.33 |
| 212710_at | calmodulin regulated spectrin-associated protein 1 | CAMSAP1 | AL043774 | Hs.380639 | chr9q34.3 |
| 212708_at | male-specific lethal-1 homolog | MSL-1 | AV721987 | Hs.350229 | chr17q21.1 |
| 212707_s_at | RAS p21 protein activator 4 /// hypothetical protein FLJ21767 | RASA4 | AI738591 | Hs.489479 | chr7q22-q31.1 |
| 212700_x_at | pleckstrin homology domain containing, family M (with RUN domain) member 1 /// similar to pleckstrin homology domain containing, family M (with RUN domain) member 1; adapter protein 162 | PLEKHM1 | AJ002220 | Hs.420584 | chr17q21.31 |
| 212697_at | hypothetical protein LOC162427 | LOC162427 | AL515874 | Hs.432850 | chr17q21.2 |
| 212696_s_at | ring finger protein 4 | RNF4 | BF968633 | Hs.66394 | chr4p16.3 |
| 212694_s_at | propionyl Coenzyme A carboxylase, beta polypeptide | PCCB | NM_000532 | Hs.63788 | chr3q21-q22 |
| 212693_at | MDN1, midasin homolog (yeast) | MDN1 | BE670928 | Hs.181334 | chr6q15 |
| 212691_at | nucleoporin 188kDa | NUP188 | AW131863 | Hs.135224 | chr9q34.11 |
| 212689_s_at | jumonji domain containing 1A | JMJD1A | AA524505 | Hs.321707 | chr2p11.2 |
| 212688_at | phosphoinositide-3-kinase, catalytic, beta polypeptide | PIK3CB | BC003393 | Hs.239818 | chr3q22.3 |
| 212686_at | protein phosphatase 1H (PP2C domain containing) | PPM1H | AB032983 | Hs.21894 | chr12q14.1-q14.2 |
| 212685_s_at | transducin (beta)-like 2 | TBL2 | AI608789 | Hs.52515 | chr7q11.23 |
| 212684_at | zinc finger protein 3 | ZNF3 | AI752257 | Hs.435302 | chr7q22.1 |
| 212680_x_at | protein phosphatase 1, regulatory (inhibitor) subunit 14B | PPP1R14B | BE305165 | Hs.120197 | chr11q13 |
| 212678_at | neurofibromin 1 (neurofibromatosis, von Recklinghausen disease, Watson disease) | NF1 | AW054826 | Hs.93207 | chr17q11.2 |
| 212673_at | methionyl aminopeptidase 1 | METAP1 | D42084 | Hs.82007 | chr4q23 |
| 212672_at | ataxia telangiectasia mutated (includes complementation groups A, C and D) | ATM | U82828 | Hs.526394 | chr11q22-q23 |
| 212666_at | SMAD specific E3 ubiquitin protein ligase 1 /// similar to SMAD specific E3 ubiquitin protein ligase 1 /// similar to SMAD specific E3 ubiquitin protein ligase 1 /// similar to SMAD specific E3 ubiquitin protein ligase 1 | SMURF1 | AB046845 | Hs.436249 | chr7q21.1-q31.1 |
| 212665_at | TCDD-inducible poly(ADP-ribose) polymerase | TIPARP | AL556438 | Hs.12813 | chr3q25.31 |
| 212662_at | poliovirus receptor | PVR | BE615277 | Hs.171844 | chr19q13.2 |
| 212660_at | PHD finger protein 15 | PHF15 | AI735639 | Hs.397990 | chr5q31.1 |
| 212658_at | lipoma HMGIC fusion partner-like 2 | LHFPL2 | N66633 | Hs.79299 | chr5q14.1 |
| 212657_s_at | interleukin 1 receptor antagonist | IL1RN | U65590 | Hs.81134 | chr2q14.2 |
| 212655_at | zinc finger, CCHC domain containing 14 | ZCCHC14 | AB011151 | Hs.81505 | chr16q24.2 |
| 212654_at | tropomyosin 2 (beta) /// peptidylprolyl isomerase (cyclophilin)-like 5 | TPM2 | AL566786 | Hs.300772 | chr9p13.2-p13.1 |
| 212651_at | Rho-related BTB domain containing 1 | RHOBTB1 | AB018283 | Hs.15099 | chr10q21.2 |
| 212650_at | EH domain binding protein 1 | EHBP1 | BF116032 | Hs.16218 | chr2p15 |
| 212648_at | DEAH (Asp-Glu-Ala-His) box polypeptide 29 | DHX29 | AL079292 | Hs.444208 | chr5q11.2 |
| 212647_at | related RAS viral (r-ras) oncogene homolog | RRAS | NM_006270 | Hs.9651 | chr19q13.3-qter |
| 212646_at | raftlin, lipid raft linker 1 | RFTN1 | D42043 | Hs.436432 | chr3p25.1-p24.3 |
| 212641_at | human immunodeficiency virus type I enhancer binding protein 2 | HIVEP2 | AL023584 | Hs.75063 | chr6q23-q24 |
| 212637_s_at | WW domain containing E3 ubiquitin protein ligase 1 | WWP1 | AU155187 | Hs.191668 | chr8q21 |
| 212633_at | KIAA0776 | KIAA0776 | AL132776 | Hs.149367 | chr6q16.1 |
| 212630_at | exocyst complex component 3 | EXOC3 | AF055006 | Hs.448580 | chr5p15.33 |
| 212628_at | protein kinase N2 | PKN2 | BG292065 | Hs.69171 | chr1p22.2 |
| 212627_s_at | exosome component 7 | EXOSC7 | AL581473 | Hs.254717 | chr3p21.31 |
| 212625_at | syntaxin 10 | STX10 | NM_003765 | Hs.43812 | chr19p13.13 |
| 212622_at | transmembrane protein 41B | TMEM41B | N64760 |  |  |
| 212621_at | KIAA0286 protein | KIAA0286 | AB006624 | Hs.14912 | chr12q13.3 |
| 212620_at | zinc finger protein 609 | ZNF609 | AW165979 | Hs.437424 | chr15q22.31 |
| 212612_at | REST corepressor 1 | RCOR1 | D31888 | Hs.405373 | chr14q32.32 |
| 212603_at | mitochondrial ribosomal protein S31 | MRPS31 | NM_005830 | Hs.154655 | chr13q14.11 |
| 212602_at | WD repeat and FYVE domain containing 3 | WDFY3 | AI806395 | Hs.105340 | chr4q21.23 |
| 212601_at | zinc finger, ZZ-type with EF-hand domain 1 | ZZEF1 | AB007859 | Hs.172179 | chr17p13.2 |
| 212596_s_at | high-mobility group protein 2-like 1 | HMG2L1 | AJ010070 | Hs.92260 | chr22q13.1 |
| 212594_at | programmed cell death 4 (neoplastic transformation inhibitor) | PDCD4 | AI185160 | Hs.257697 | chr10q24 |
| 212588_at | protein tyrosine phosphatase, receptor type, C | PTPRC | Y00062 | Hs.444324 | chr1q31-q32 |
| 212583_at | aquarius homolog (mouse) | AQR | AB011132 |  |  |
| 212581_x_at | glyceraldehyde-3-phosphate dehydrogenase | GAPDH | BE561479 | Hs.169476 | chr12p13 |
| 212577_at | structural maintenance of chromosomes flexible hinge domain containing 1 | SMCHD1 | AA868754 | Hs.8118 | chr18p11.32 |
| 212576_at | mahogunin, ring finger 1 | MGRN1 | AB011116 | Hs.390004 | chr16p13.3 |
| 212573_at | endonuclease domain containing 1 | ENDOD1 | AF131747 | Hs.167115 | chr11q21 |
| 212571_at | chromodomain helicase DNA binding protein 8 | CHD8 | U00955 | Hs.444045 | chr14q11.2 |
| 212565_at | serine/threonine kinase 38 like | STK38L | BE302191 |  |  |
| 212563_at | block of proliferation 1 /// similar to block of proliferation 1 | BOP1 | BG491842 | Hs.511945 | chr8q24.3 |
| 212561_at | RAB6 interacting protein 1 | RAB6IP1 | AA349595 | Hs.26797 | chr11p15.4 |
| 212559_at | protein kinase, cAMP-dependent, regulatory, type I, beta | PRKAR1B | AU148827 |  |  |
| 212556_at | scribbled homolog (Drosophila) | SCRIB | AI469403 | Hs.436329 | chr8q24.3 |
| 212554_at | CAP, adenylate cyclase-associated protein, 2 (yeast) | CAP2 | N90755 | Hs.296341 | chr6p22.3 |
| 212553_at | KIAA0460 | KIAA0460 | AK026120 | Hs.29956 | chr1q21.2 |
| 212552_at | hippocalcin-like 1 | HPCAL1 | BE617588 | Hs.3618 | chr2p25.1 |
| 212544_at | zinc finger, HIT type 3 | ZNHIT3 | AI131008 | Hs.2210 | chr17q12 |
| 212543_at | absent in melanoma 1 | AIM1 | U83115 | Hs.422550 | chr6q21 |
| 212542_s_at | pleckstrin homology domain interacting protein | PHIP | BF224151 | Hs.9927 | chr6q14 |
| 212540_at | cell division cycle 34 homolog (S. cerevisiae) | CDC34 | BG476661 | Hs.423615 | chr19p13.3 |
| 212539_at | chromodomain helicase DNA binding protein 1-like | CHD1L | AI422099 | Hs.429920 | chr1q12 |
| 212538_at | dedicator of cytokinesis 9 | DOCK9 | AL576253 | Hs.513063 | chr13q32.3 |
| 212533_at | WEE1 homolog (S. pombe) | WEE1 | X62048 | Hs.249441 | chr11p15.3-p15.1 |
| 212530_at | NIMA (never in mitosis gene a)-related kinase 7 | NEK7 | AL080111 | Hs.24119 | chr1q31.3 |
| 212529_at | LSM12 homolog (S. cerevisiae) | LSM12 | BF197707 | Hs.355570 | chr17q21.31 |
| 212527_at | DNA segment, Chr 15, Wayne State University 75, expressed | D15Wsu75e | BF057059 | Hs.335274 | chr22q13.2 |
| 212521_s_at | phosphodiesterase 8A | PDE8A | BE568219 | Hs.78746 | chr15q25.3 |
| 212518_at | phosphatidylinositol-4-phosphate 5-kinase, type I, gamma | PIP5K1C | AB011161 | Hs.282177 | chr19p13.3 |
| 212514_x_at | DEAD (Asp-Glu-Ala-Asp) box polypeptide 3, X-linked | DDX3X | R60068 | Hs.380774 | chrxp11.3-p11.23 |
| 212512_s_at | coactivator-associated arginine methyltransferase 1 | CARM1 | AA551784 | Hs.371416 | chr19p13.2 |
| 212510_at | glycerol-3-phosphate dehydrogenase 1-like | GPD1L | AA135522 | Hs.82432 | chr3p24.1 |
| 212509_s_at | matrix-remodelling associated 7 | MXRA7 | BF968134 | Hs.250723 | chr17q25.3 |
| 212508_at | modulator of apoptosis 1 | MOAP1 | AK024029 | Hs.24719 | chr14q32 |
| 212507_at | transmembrane protein 131 | TMEM131 | D87446 | Hs.318783 | chr2q11.2 |
| 212505_s_at | KIAA0892 | KIAA0892 | AL110250 | Hs.369952 | chr19p13.11 |
| 212504_at | DIP2 disco-interacting protein 2 homolog C (Drosophila) | DIP2C | N31807 | Hs.116204 | chr10p15.3 |
| 212501_at | CCAAT/enhancer binding protein (C/EBP), beta | CEBPB | AL564683 | Hs.99029 | chr20q13.1 |
| 212500_at | chromosome 10 open reading frame 22 | C10orf22 | AL049319 | Hs.99821 | chr10q21.3 |
| 212496_s_at | jumonji domain containing 2B | JMJD2B | BE256900 | Hs.131726 | chr19p13.3 |
| 212490_at | DnaJ (Hsp40) homolog, subfamily C, member 8 | DNAJC8 | AA843895 | Hs.433540 | chr1p35.3 |
| 212485_at | G patch domain containing 8 | GPATCH8 | AU146596 | Hs.396047 | chr17q21.31 |
| 212479_s_at | required for meiotic nuclear division 5 homolog A (S. cerevisiae) | RMND5A | AK022815 | Hs.75277 | chr2p11.2 |
| 212476_at | centaurin, beta 2 | CENTB2 | D26069 | Hs.291851 | chr3q29 |
| 212472_at | microtubule associated monoxygenase, calponin and LIM domain containing 2 | MICAL2 | BE965029 | Hs.309674 | chr11p15.3 |
| 212471_at | KIAA0241 | KIAA0241 | BE503381 | Hs.134792 | chr7p14.3 |
| 212469_at | Nipped-B homolog (Drosophila) | NIPBL | AB019494 | Hs.225767 | chr5p13.2 |
| 212467_at | DnaJ (Hsp40) homolog, subfamily C, member 13 | DNAJC13 | AB014578 | Hs.12707 | chr3q22.1 |
| 212465_at | SET domain containing 3 | SETD3 | AA524500 | Hs.321766 | chr14q32.2 |
| 212458_at | sprouty-related, EVH1 domain containing 2 | SPRED2 | H97931 | Hs.173108 | chr2p14 |
| 212456_at | KIAA0664 | KIAA0664 | AB014564 | Hs.22616 | chr17p13.3 |
| 212453_at | KIAA1279 | KIAA1279 | AB033105 | Hs.279580 | chr10q22.1 |
| 212450_at | KIAA0256 gene product /// trafficking protein particle complex 5 | KIAA0256 | D87445 | Hs.372033 | chr15q21.1 |
| 212439_at | inositol hexaphosphate kinase 1 | IHPK1 | BE614199 |  |  |
| 212437_at | centromere protein B, 80kDa | CENPB | AL109804 | Hs.85004 | chr20p13 |
| 212436_at | tripartite motif-containing 33 | TRIM33 | AI967961 | Hs.293044 |  |
| 212432_at | GrpE-like 1, mitochondrial (E. coli) | GRPEL1 | AL542571 | Hs.151903 | chr4p16 |
| 212430_at | RNA binding motif protein 38 /// RNA binding motif protein 38 | RBM38 | AL109955 | Hs.236361 | chr20q13.32 |
| 212428_at | KIAA0368 | KIAA0368 | AW001101 | Hs.445255 | chr9q31.3 |
| 212423_at | chromosome 10 open reading frame 56 | C10orf56 | AK024784 | Hs.28264 | chr10q22.3 |
| 212422_at | programmed cell death 11 | PDCD11 | AL547263 | Hs.239499 | chr10q24.33 |
| 212421_at | chromosome 22 open reading frame 9 | C22orf9 | AB023147 | Hs.307498 | chr22q13.31 |
| 212418_at | E74-like factor 1 (ets domain transcription factor) | ELF1 | M82882 | Hs.135646 | chr13q13 |
| 212414_s_at | septin 6 /// cytokine-like nuclear factor n-pac | SEPT6 | D50918 | Hs.90998 | chrxq24 |
| 212411_at | IMP4, U3 small nucleolar ribonucleoprotein, homolog (yeast) | IMP4 | BE747342 | Hs.91579 | chr2q21.1 |
| 212410_at | EF-hand domain family, member A1 | EFHA1 | AI346431 | Hs.412103 | chr13q12.11 |
| 212409_s_at | torsin A interacting protein 1 | TOR1AIP1 | AK021613 | Hs.234265 | chr1q24.2 |
| 212406_s_at | protein-L-isoaspartate (D-aspartate) O-methyltransferase domain containing 2 | PCMTD2 | AB028973 | Hs.184628 | chr20q13.33 |
| 212405_s_at | KIAA0859 | KIAA0859 | AK001172 | Hs.19469 | chr1q24-q25.3 |
| 212403_at | ubiquitin protein ligase E3B | UBE3B | AI749193 | Hs.305834 | chr12q24.11 |
| 212396_s_at | KIAA0090 | KIAA0090 | AI143233 | Hs.439200 | chr1p36.13 |
| 212388_at | ubiquitin specific peptidase 24 | USP24 | AB028980 | Hs.7243 | chr1p32.3 |
| 212387_at | Transcription factor 4 | TCF4 | BG495771 | Hs.359289 | chr18q21.1 |
| 212383_at | ATPase, H+ transporting, lysosomal V0 subunit a1 | ATP6V0A1 | AL096733 | Hs.267871 | chr17q21 |
| 212380_at | KIAA0082 | KIAA0082 | D43949 | Hs.202331 | chr6p21.2 |
| 212376_s_at | E1A binding protein p400 | EP400 | BE880591 | Hs.306094 | chr12q24.33 |
| 212374_at | fem-1 homolog b (C. elegans) | FEM1B | NM_015322 | Hs.362733 | chr15q22 |
| 212369_at | zinc finger protein 384 | ZNF384 | AI264312 | Hs.103315 | chr12p12 |
| 212366_at | zinc finger protein 292 | ZNF292 | BF223237 | Hs.448341 | chr6q15 |
| 212364_at | myosin IB | MYO1B | BF432550 | Hs.121576 | chr2q12-q34 |
| 212360_at | adenosine monophosphate deaminase 2 (isoform L) | AMPD2 | AI916249 | Hs.82927 | chr1p13.3 |
| 212359_s_at | KIAA0913 | KIAA0913 | W89120 | Hs.65135 | chr10q22.2 |
| 212357_at | KIAA0280 | KIAA0280 | AI096888 | Hs.534443 | chr11q13.4 |
| 212355_at | KIAA0323 | KIAA0323 | AI075450 | Hs.451063 | chr14q11.2 |
| 212351_at | eukaryotic translation initiation factor 2B, subunit 5 epsilon, 82kDa | EIF2B5 | U23028 | Hs.283551 | chr3q27.1 |
| 212349_at | protein O-fucosyltransferase 1 | POFUT1 | AL045513 | Hs.178292 | chr20q11 |
| 212348_s_at | amine oxidase (flavin containing) domain 2 | AOF2 | AB011173 | Hs.454808 | chr1p36.12 |
| 212346_s_at | MAX dimerization protein 4 | MXD4 | AK026392 | Hs.102402 | chr4p16.3 |
| 212345_s_at | cAMP responsive element binding protein 3-like 2 | CREB3L2 | BE675139 | Hs.59943 | chr7q34 |
| 212343_at | Yip1 domain family, member 6 | YIPF6 | AL117461 | Hs.82719 | chrxq13.1 |
| 212338_at | myosin ID | MYO1D | AA621962 | Hs.462777 | chr17q11-q12 |
| 212337_at | hypothetical protein TI-227H /// taurine upregulated gene 1 | TI-227H | AI687738 |  | chr22 |
| 212336_at | erythrocyte membrane protein band 4.1-like 1 | EPB41L1 | AA912711 | Hs.437422 | chr20q11.2-q12 |
| 212334_at | glucosamine (N-acetyl)-6-sulfatase (Sanfilippo disease IIID) | GNS | BE880245 | Hs.334534 | chr12q14 |
| 212333_at | family with sequence similarity 98, member A | FAM98A | AL049943 | Hs.23060 | chr2p22.3 |
| 212332_at | retinoblastoma-like 2 (p130) | RBL2 | BF110947 | Hs.283604 | chr16q12.2 |
| 212330_at | transcription factor Dp-1 | TFDP1 | R60866 | Hs.79353 | chr13q34 |
| 212329_at | SREBP cleavage-activating protein | SCAP | D83782 | Hs.437096 | chr3p21.31 |
| 212323_s_at | vacuolar protein sorting 13 homolog D (S. cerevisiae) | VPS13D | AA524553 | Hs.194737 | chr1p36.22-p36.21 |
| 212322_at | sphingosine-1-phosphate lyase 1 | SGPL1 | BE999972 | Hs.186613 | chr10q21 |
| 212317_at | transportin 3 | TNPO3 | AK022910 | Hs.412527 | chr7q32.3 |
| 212314_at | KIAA0746 protein | KIAA0746 | AB018289 | Hs.49500 | chr4p15.2 |
| 212313_at | CHMP family, member 7 | CHMP7 | BC004344 | Hs.5019 | chr8p21.3 |
| 212308_at | cytoplasmic linker associated protein 2 | CLASP2 | AL137636 | Hs.368691 | chr3p23 |
| 212302_at | Rtf1, Paf1/RNA polymerase II complex component, homolog (S. cerevisiae) | RTF1 | AI192081 | Hs.83419 | chr15q15.1 |
| 212300_at | taxilin alpha | TXLNA | AL049795 | Hs.121150 | chr1p35.1 |
| 212299_at | NIMA (never in mitosis gene a)- related kinase 9 | NEK9 | AL117502 | Hs.7200 | chr14q24.3 |
| 212297_at | ATPase type 13A3 | ATP13A3 | BF218804 | Hs.371148 | chr3q29 |
| 212296_at | proteasome (prosome, macropain) 26S subunit, non-ATPase, 14 | PSMD14 | NM_005805 | Hs.356623 | chr2q24.2 |
| 212292_at | solute carrier family 7 (cationic amino acid transporter, y+ system), member 1 | SLC7A1 | AW452623 | Hs.14846 | chr13q12-q14 |
| 212291_at | homeodomain interacting protein kinase 1 | HIPK1 | AI393355 | Hs.12259 | chr1p13.2 |
| 212288_at | formin binding protein 1 | FNBP1 | AB011126 | Hs.440808 | chr9q34 |
| 212286_at | ankyrin repeat domain 12 | ANKRD12 | AW572909 | Hs.388877 | chr18p11.22 |
| 212280_x_at | similar to APG4 autophagy 4 homolog B isoform a | LOC727737 | AA532726 | Hs.283610 | chr2q37.3 |
| 212277_at | myotubularin related protein 4 | MTMR4 | AB014547 | Hs.141727 | chr17q22-q23 |
| 212272_at | lipin 1 | LPIN1 | AA813260 | Hs.81412 | chr2p25.1 |
| 212269_s_at | MCM3 minichromosome maintenance deficient 3 (S. cerevisiae) associated protein | MCM3AP | AJ010089 | Hs.389037 | chr21q22.3 |
| 212268_at | serpin peptidase inhibitor, clade B (ovalbumin), member 1 | SERPINB1 | NM_030666 | Hs.381167 | chr6p25 |
| 212259_s_at | pre-B-cell leukemia transcription factor interacting protein 1 | PBXIP1 | BF344265 | Hs.505806 | chr1q22 |
| 212256_at | UDP-N-acetyl-alpha-D-galactosamine:polypeptide N-acetylgalactosaminyltransferase 10 (GalNAc-T10) | GALNT10 | BE906572 | Hs.13785 | chr5q33.2 |
| 212252_at | calcium/calmodulin-dependent protein kinase kinase 2, beta | CAMKK2 | AA181179 | Hs.297343 | chr12q24.2 |
| 212249_at | phosphoinositide-3-kinase, regulatory subunit 1 (p85 alpha) | PIK3R1 | AI934473 | Hs.6241 | chr5q13.1 |
| 212248_at | CDNA FLJ41088 fis, clone ASTRO2002459 /// Metadherin | MTDH | AI886796 | Hs.377155 | chr8q22.1 |
| 212245_at | multiple coagulation factor deficiency 2 | MCFD2 | AL567779 | Hs.293689 | chr2p21 |
| 212242_at | tubulin, alpha 1 | TUBA1 | AL565074 | Hs.75318 | chr2q35 |
| 212241_at | glutamate receptor, ionotropic, N-methyl D-aspartate-like 1A /// GRINL1A combined protein /// similar to glutamate receptor, ionotropic, N-methyl D-aspartate-like 1A isoform 1 | GRINL1A | AI632774 | Hs.201495 | chr15q22.1 |
| 212234_at | additional sex combs like 1 (Drosophila) | ASXL1 | AL034550 | Hs.3686 | chr20q11.1 |
| 212232_at | formin binding protein 4 | FNBP4 | AB023231 | Hs.6834 | chr11p11.2 |
| 212231_at | F-box protein 21 | FBXO21 | AB020682 | Hs.184227 | chr12q24.22 |
| 212228_s_at | coenzyme Q9 homolog (S. cerevisiae) | COQ9 | AC004382 | Hs.444253 | chr16q13 |
| 212220_at | proteasome (prosome, macropain) activator subunit 4 | PSME4 | AI972268 | Hs.119563 | chr2p16.3 |
| 212218_s_at | fatty acid synthase | FASN | AI954041 | Hs.83190 | chr17q25 |
| 212216_at | prolyl endopeptidase-like | PREPL | AW000954 | Hs.535763 | chr2p22.1 |
| 212212_s_at | integrator complex subunit 1 | INTS1 | BF055496 | Hs.112184 | chr7p22.3 |
| 212206_s_at | H2A histone family, member V | H2AFV | BF343852 | Hs.301005 | chr7p13 |
| 212203_x_at | interferon induced transmembrane protein 3 (1-8U) | IFITM3 | BF338947 | Hs.374650 | chr11p15.5 |
| 212201_at | KIAA0692 | KIAA0692 | AW274877 | Hs.385927 | chr12q24.33 |
| 212199_at | Morf4 family associated protein 1-like 1 | MRFAP1L1 | AL566962 | Hs.284281 | chr4p16.1 |
| 212194_s_at | transmembrane 9 superfamily protein member 4 | TM9SF4 | AI418892 | Hs.79305 | chr20q11.21 |
| 212190_at | serpin peptidase inhibitor, clade E (nexin, plasminogen activator inhibitor type 1), member 2 | SERPINE2 | AL541302 | Hs.21858 | chr2q33-q35 |
| 212189_s_at | component of oligomeric golgi complex 4 | COG4 | AK022874 | Hs.208680 | chr16q22.1 |
| 212188_at | potassium channel tetramerisation domain containing 12 /// potassium channel tetramerisation domain containing 12 | KCTD12 | AA551075 | Hs.109438 | chr13q22.3 |
| 212186_at | acetyl-Coenzyme A carboxylase alpha | ACACA | BE855983 | Hs.449863 | chr17q21 |
| 212185_x_at | metallothionein 2A | MT2A | NM_005953 |  |  |
| 212177_at | chromosome 6 open reading frame 111 | C6orf111 | AW081113 | Hs.414993 | chr6q16.3 |
| 212170_at | RNA binding motif protein 12 | RBM12 | BF447705 | Hs.166887 | chr20q11.21 |
| 212169_at | FK506 binding protein 9, 63 kDa | FKBP9 | AL050187 | Hs.497972 | chr7p11.1 |
| 212167_s_at | SWI/SNF related, matrix associated, actin dependent regulator of chromatin, subfamily b, member 1 | SMARCB1 | AK021419 | Hs.512700 | chr22q11.23 |
| 212164_at | transmembrane protein 183A | TMEM183A | AL522296 | Hs.408302 | chr1q32.1 |
| 212162_at | kinase D-interacting substance of 220 kDa | KIDINS220 | AK022873 | Hs.9873 | chr2p24 |
| 212160_at | exportin, tRNA (nuclear export receptor for tRNAs) | XPOT | AI984005 | Hs.85951 | chr12q14.2 |
| 212157_at | syndecan 2 (heparan sulfate proteoglycan 1, cell surface-associated, fibroglycan) | SDC2 | BE048514 | Hs.1501 | chr8q22-q23 |
| 212155_at | ring finger protein 187 | RNF187 | AA085748 | Hs.356377 | chr1q42.13 |
| 212149_at | KIAA0143 protein | KIAA0143 | AW470003 | Hs.84087 | chr8q24.22 |
| 212148_at | Pre-B-cell leukemia transcription factor 1 | PBX1 | AL049381 | Hs.307374 | chr1q23 |
| 212146_at | pleckstrin homology domain containing, family M (with RUN domain) member 2 | PLEKHM2 | AB020649 | Hs.24183 | chr1p36.13 |
| 212145_at | mitochondrial ribosomal protein S27 | MRPS27 | D87453 | Hs.376200 | chr5q13.2 |
| 212144_at | unc-84 homolog B (C. elegans) | UNC84B | AL021707 | Hs.406612 | chr22q13.1 |
| 212143_s_at | insulin-like growth factor binding protein 3 | IGFBP3 | BF340228 | Hs.450230 | chr7p13-p12 |
| 212135_s_at | ATPase, Ca++ transporting, plasma membrane 4 | ATP2B4 | AW517686 | Hs.343522 | chr1q32.1 |
| 212134_at | pleckstrin homology-like domain, family B, member 1 | PHLDB1 | AB014538 | Hs.149653 | chr11q23.3 |
| 212133_at | Non imprinted in Prader-Willi/Angelman syndrome 2 | NIPA2 | AI681536 | Hs.512761 | chr15q11.2 |
| 212131_at | LSM14A, SCD6 homolog A (S. cerevisiae) | LSM14A | BG054966 | Hs.8258 | chr19q13.11 |
| 212128_s_at | dystroglycan 1 (dystrophin-associated glycoprotein 1) | DAG1 | AW411370 | Hs.76111 | chr3p21 |
| 212124_at | zinc finger, MIZ-type containing 1 | ZMIZ1 | AF070622 | Hs.438767 | chr10q22.3 |
| 212123_at | chromosome 10 open reading frame 61 | C10orf61 | AL050022 | Hs.438991 | chr10q24.1 |
| 212122_at | ras homolog gene family, member Q | RHOQ | AW771590 | Hs.442989 | chr2p21 |
| 212114_at | hypothetical LOC552889 | LOC552889 | BE967207 | Hs.336425 | chr12q21.1 |
| 212111_at | syntaxin 12 | STX12 | AA628051 | Hs.433838 | chr1p35-p34.1 |
| 212110_at | solute carrier family 39 (zinc transporter), member 14 | SLC39A14 | D31887 | Hs.301743 | chr8p21.3 |
| 212109_at | hematological and neurological expressed 1-like | HN1L | AI590869 | Hs.437433 | chr16p13.3 |
| 212107_s_at | DEAH (Asp-Glu-Ala-His) box polypeptide 9 | DHX9 | BE561014 | Hs.374524 | chr1q25 |
| 212106_at | UBX domain containing 8 | UBXD8 | BF116183 | Hs.76591 | chr5q35.2 |
| 212102_s_at | karyopherin alpha 6 (importin alpha 7) | KPNA6 | AK002111 | Hs.372741 | chr1p35.1-p34.3 |
| 212098_at | hypothetical protein LOC151162 | LOC151162 | AL134724 | Hs.4988 | chr2q21.3 |
| 212092_at | paternally expressed 10 | PEG10 | BE858180 | Hs.137476 | chr7q21 |
| 212090_at | glutamate receptor, ionotropic, N-methyl D-asparate-associated protein 1 (glutamate binding) | GRINA | AL571424 | Hs.339697 | chr8q24.3 |
| 212088_at | peptidase (mitochondrial processing) alpha /// HIG1 domain family, member 2B pseudogene | PMPCA | BF570122 | Hs.75353 | chr9q34.3 |
| 212087_s_at | Era G-protein-like 1 (E. coli) | ERAL1 | AL562733 | Hs.3426 | chr17q11.2 |
| 212085_at | solute carrier family 25 (mitochondrial carrier; adenine nucleotide translocator), member 6 | SLC25A6 | AA916851 | Hs.350927 | chrxp22.32 and yp |
| 212084_at | testis expressed sequence 261 | TEX261 | AV759552 | Hs.333190 | chr2p13.3 |
| 212079_s_at | myeloid/lymphoid or mixed-lineage leukemia (trithorax homolog, Drosophila) | MLL | AA715041 | Hs.258855 | chr11q23 |
| 212074_at | unc-84 homolog A (C. elegans) | UNC84A | BE972774 | Hs.438072 | chr7p22.3 |
| 212070_at | G protein-coupled receptor 56 | GPR56 | AL554008 | Hs.6527 | chr16q13 |
| 212068_s_at | KIAA0515 | KIAA0515 | AB011087 | Hs.412624 | chr9q34.13 |
| 212067_s_at | complement component 1, r subcomponent | C1R | AL573058 | Hs.376414 | chr12p13 |
| 212065_s_at | thyroid hormone receptor, beta (erythroblastic leukemia viral (v-erb-a) oncogene homolog 2, avian) /// ubiquitin specific peptidase 34 | THRB | AW502434 | Hs.507665 | chr2p15 |
| 212064_x_at | MYC-associated zinc finger protein (purine-binding transcription factor) | MAZ | AI471665 | Hs.330418 | chr16p11.2 |
| 212062_at | ATPase, Class II, type 9A | ATP9A | AB014511 | Hs.368002 | chr20q13.11-q13.2 |
| 212061_at | U2-associated SR140 protein | SR140 | AB002330 | Hs.370907 | chr3q23 |
| 212059_s_at | transient receptor potential cation channel, subfamily C, member 4 associated protein | TRPC4AP | AL117480 | Hs.168073 | chr20q11.22 |
| 212056_at | KIAA0182 | KIAA0182 | D80004 | Hs.222171 | chr16q24.1 |
| 212052_s_at | TBC1 domain family, member 9B (with GRAM domain) | TBC1D9B | AB014576 | Hs.155829 | chr5q35.3 |
| 212048_s_at | tyrosyl-tRNA synthetase | YARS | AW245400 | Hs.322735 | chr1p35.1 |
| 212047_s_at | ring finger protein 167 | RNF167 | AK025329 | Hs.7158 | chr17p13.2 |
| 212046_x_at | mitogen-activated protein kinase 3 | MAPK3 | X60188 | Hs.861 | chr16p12-p11.2 |
| 212045_at | golgi apparatus protein 1 | GLG1 | N32761 | Hs.78979 | chr16q22-q23 |
| 212044_s_at | Ribosomal protein L27a | RPL27A | BE737027 |  |  |
| 212041_at | ATPase, H+ transporting, lysosomal 38kDa, V0 subunit d1 | ATP6V0D1 | AL566172 | Hs.106876 | chr16q22 |
| 212038_s_at | voltage-dependent anion channel 1 | VDAC1 | AL515918 | Hs.404814 | chr5q31 |
| 212032_s_at | prostate tumor overexpressed gene 1 | PTOV1 | AL046054 | Hs.227429 | chr19q13.33 |
| 212026_s_at | exocyst complex component 7 | EXOC7 | BE646386 | Hs.325530 | chr17q25.1 |
| 212024_x_at | flightless I homolog (Drosophila) | FLII | U80184 | Hs.445182 | chr17p11.2 |
| 212019_at | ribosomal L1 domain containing 1 | RSL1D1 | AK025446 | Hs.528354 | chr16p13.13 |
| 212016_s_at | polypyrimidine tract binding protein 1 | PTBP1 | AA679988 | Hs.172550 | chr19p13.3 |
| 212012_at | peroxidasin homolog (Drosophila) | PXDN | BF342851 | Hs.118893 | chr2p25 |
| 212009_s_at | stress-induced-phosphoprotein 1 (Hsp70/Hsp90-organizing protein) | STIP1 | AL553320 | Hs.257827 | chr11q13 |
| 212006_at | UBX domain containing 2 | UBXD2 | AU149908 | Hs.350806 | chr2q21.3 |
| 212004_at | chromosome 1 open reading frame 144 | C1orf144 | AL050028 | Hs.252967 | chr1p36.13 |
| 212001_at | splicing factor, arginine/serine-rich 14 | SFRS14 | AV738039 | Hs.134093 | chr19p12 |
| 211998_at | H3 histone, family 3B (H3.3B) | H3F3B | AW138159 | Hs.180877 | chr17q25 |
| 211996_s_at | KIAA0220-like protein /// hypothetical gene LOC283846 /// hypothetical protein LOC440345 /// nuclear pore complex interacting protein pseudogene /// nuclear pore complex interacting protein pseudogene /// similar to Protein KIAA0220 | LOC23117 | BG256504 | Hs.110613 | chr16p12.3 |
| 211993_at | WNK lysine deficient protein kinase 1 /// WNK lysine deficient protein kinase 1 | WNK1 | AI768512 | Hs.275999 | chr12p13.3 |
| 211991_s_at | major histocompatibility complex, class II, DP alpha 1 | HLA-DPA1 | M27487 | Hs.914 | chr6p21.3 |
| 211987_at | topoisomerase (DNA) II beta 180kDa | TOP2B | NM_001068 | Hs.282346 | chr3p24 |
| 211986_at | AHNAK nucleoprotein (desmoyokin) | AHNAK | BG287862 | Hs.378738 | chr11q12-q13 |
| 211980_at | collagen, type IV, alpha 1 | COL4A1 | AI922605 | Hs.437173 | chr13q34 |
| 211977_at | G protein-coupled receptor 107 | GPR107 | AK024651 | Hs.512461 | chr9q34.11 |
| 211975_at | zinc finger protein 289, ID1 regulated | ZNF289 | BE299671 | Hs.436204 | chr11p11.2-p11.12 |
| 211967_at | transmembrane protein 123 | TMEM123 | BG538627 | Hs.172089 | chr11q22.1 |
| 211966_at | collagen, type IV, alpha 2 | COL4A2 | AA909035 | Hs.407912 | chr13q34 |
| 211965_at | zinc finger protein 36, C3H type-like 1 | ZFP36L1 | BE620915 | Hs.85155 | chr14q22-q24 |
| 211960_s_at | RAB7, member RAS oncogene family | RAB7 | BG261416 | Hs.356386 | chr3q21.3 |
| 211952_at | RAN binding protein 5 | RANBP5 | AF339834 | Hs.113503 | chr13q32.2 |
| 211949_s_at | nucleolar and coiled-body phosphoprotein 1 | NOLC1 | AI355279 | Hs.75337 | chr10q24.32 |
| 211943_x_at | tumor protein, translationally-controlled 1 | TPT1 | AL565449 | Hs.374596 | chr13q12-q14 |
| 211937_at | eukaryotic translation initiation factor 4B | EIF4B | NM_001417 | Hs.93379 | chr12q13.13 |
| 211935_at | ADP-ribosylation factor-like 6 interacting protein 1 | ARL6IP1 | D31885 | Hs.75249 | chr16p12-p11.2 |
| 211934_x_at | glucosidase, alpha; neutral AB | GANAB | W87689 | Hs.76847 | chr11q12.3 |
| 211930_at | heterogeneous nuclear ribonucleoprotein A3 | HNRPA3 | AW080932 | Hs.446430 | chr2q31.2 |
| 211928_at | dynein, cytoplasmic 1, heavy chain 1 | DYNC1H1 | AB002323 | Hs.7720 | chr14q32 |
| 211926_s_at | myosin, heavy chain 9, non-muscle | MYH9 | AI827941 | Hs.146550 | chr22q13.1 |
| 211922_s_at | catalase /// catalase | CAT | AY028632 | Hs.395771 | chr11p13 |
| 211913_s_at | c-mer proto-oncogene tyrosine kinase /// c-mer proto-oncogene tyrosine kinase | MERTK | L08961 | Hs.306178 | chr2q14.1 |
| 211874_s_at | MYST histone acetyltransferase (monocytic leukemia) 4 | MYST4 | AF119230 | Hs.27590 | chr10q22.2 |
| 211864_s_at | fer-1-like 3, myoferlin (C. elegans) | FER1L3 | AF207990 | Hs.362731 | chr10q24 |
| 211855_s_at | solute carrier family 25 (mitochondrial carrier, brain), member 14 | SLC25A14 | AF155810 | Hs.194686 | chrxq24 |
| 211852_s_at | attractin | ATRN | AF106861 | Hs.373838 | chr20p13 |
| 211810_s_at | galactosylceramidase | GALC | D25284 | Hs.408273 | chr14q31 |
| 211799_x_at | major histocompatibility complex, class I, C | HLA-C | U62824 | Hs.181244 | chr6p21.3 |
| 211797_s_at | nuclear transcription factor Y, gamma | NFYC | U62296 | Hs.285133 | chr1p32 |
| 211783_s_at | metastasis associated 1 /// metastasis associated 1 | MTA1 | BC006177 | Hs.101448 | chr14q32.3 |
| 211780_x_at | dynactin 1 (p150, glued homolog, Drosophila) /// dynactin 1 (p150, glued homolog, Drosophila) | DCTN1 | BC006163 | Hs.74617 | chr2p13 |
| 211769_x_at | serine incorporator 3 /// serine incorporator 3 | SERINC3 | BC006088 | Hs.272168 | chr20q13.1-13.3 |
| 211755_s_at | ATP synthase, H+ transporting, mitochondrial F0 complex, subunit B1 /// ATP synthase, H+ transporting, mitochondrial F0 complex, subunit B1 | ATP5F1 | BC005960 | Hs.81634 | chr1p13.2 |
| 211754_s_at | solute carrier family 25 (mitochondrial carrier; peroxisomal membrane protein, 34kDa), member 17 /// solute carrier family 25 (mitochondrial carrier; peroxisomal membrane protein, 34kDa), member 17 | SLC25A17 | BC005957 | Hs.217292 | chr22q13.2 |
| 211752_s_at | NADH dehydrogenase (ubiquinone) Fe-S protein 7, 20kDa (NADH-coenzyme Q reductase) /// NADH dehydrogenase (ubiquinone) Fe-S protein 7, 20kDa (NADH-coenzyme Q reductase) | NDUFS7 | BC005954 | Hs.211914 | chr19p13.3 |
| 211748_x_at | prostaglandin D2 synthase 21kDa (brain) /// prostaglandin D2 synthase 21kDa (brain) | PTGDS | BC005939 | Hs.446429 | chr9q34.2-q34.3 |
| 211742_s_at | ecotropic viral integration site 2B /// ecotropic viral integration site 2B | EVI2B | BC005926 | Hs.5509 | chr17q11.2 |
| 211730_s_at | polymerase (RNA) II (DNA directed) polypeptide L, 7.6kDa /// polymerase (RNA) II (DNA directed) polypeptide L, 7.6kDa | POLR2L | BC005903 | Hs.441072 | chr11p15 |
| 211721_s_at | zinc finger protein 551 /// zinc finger protein 551 | ZNF551 | BC005868 | Hs.184846 | chr19q13.43 |
| 211717_at | ankyrin repeat domain 40 /// ankyrin repeat domain 40 | ANKRD40 | BC005853 | Hs.351247 | chr17q21.33 |
| 211713_x_at | KIAA0101 /// KIAA0101 | KIAA0101 | BC005832 | Hs.81892 | chr15q22.31 |
| 211707_s_at | IQ motif containing B1 /// IQ motif containing B1 | IQCB1 | BC005806 | Hs.435802 | chr3q13.33 |
| 211701_s_at | trophinin /// trophinin | TRO | AF349720 | Hs.434971 | chrxp11.22-p11.21 |
| 211692_s_at | BCL2 binding component 3 /// BCL2 binding component 3 | BBC3 | AF332558 | Hs.87246 | chr19q13.3-q13.4 |
| 211686_s_at | RNA binding motif protein 13 /// RNA binding motif protein 13 | RBM13 | AF251062 | Hs.288716 | chr8p12 |
| 211685_s_at | neurocalcin delta /// neurocalcin delta | NCALD | AF251061 | Hs.90063 | chr8q22-q23 |
| 211684_s_at | dynein, cytoplasmic 1, intermediate chain 2 /// dynein, cytoplasmic 1, intermediate chain 2 /// similar to Cytoplasmic dynein 1 intermediate chain 2 (Dynein intermediate chain 2, cytosolic) (DH IC-2) (Cytoplasmic dynein intermediate chain 2) /// similar t | DYNC1I2 | AF250307 | Hs.87372 | chr2q31.1 |
| 211676_s_at | interferon gamma receptor 1 /// interferon gamma receptor 1 | IFNGR1 | AF056979 | Hs.180866 | chr6q23-q24 |
| 211662_s_at | voltage-dependent anion channel 2 /// voltage-dependent anion channel 2 | VDAC2 | L08666 | Hs.355927 | chr10q22 |
| 211658_at | peroxiredoxin 2 /// peroxiredoxin 2 | PRDX2 | L19185 | Hs.432121 | chr19p13.2 |
| 211654_x_at | major histocompatibility complex, class II, DQ beta 1 /// major histocompatibility complex, class II, DQ beta 1 /// similar to HLA class II histocompatibility antigen, DQ(W1.1) beta chain precursor (DQB1*0501) /// similar to HLA class II histocompatibilit | HLA-DQB1 | M17565 | Hs.409934 | chr6p21.3 |
| 211628_x_at | ferritin, heavy polypeptide pseudogene 1 /// ferritin, heavy polypeptide pseudogene 1 | FTHP1 | J04755 | Hs.453583 | chr6p21.3-p12 |
| 211623_s_at | fibrillarin /// fibrillarin | FBL | M30448 | Hs.99853 | chr19q13.1 |
| 211615_s_at | leucine-rich PPR-motif containing /// leucine-rich PPR-motif containing | LRPPRC | M92439 | Hs.182490 | chr2p21 |
| 211596_s_at | leucine-rich repeats and immunoglobulin-like domains 1 /// leucine-rich repeats and immunoglobulin-like domains 1 | LRIG1 | AB050468 | Hs.528353 | chr3p14 |
| 211595_s_at | mitochondrial ribosomal protein S11 /// mitochondrial ribosomal protein S11 | MRPS11 | AB049944 | Hs.111286 | chr15q25 |
| 211594_s_at | mitochondrial ribosomal protein L9 /// mitochondrial ribosomal protein L9 | MRPL9 | AB049636 | Hs.288936 | chr1q21 |
| 211593_s_at | microtubule associated serine/threonine kinase 2 /// microtubule associated serine/threonine kinase 2 | MAST2 | AB047005 | Hs.101474 | chr1p34.1 |
| 211592_s_at | calcium channel, voltage-dependent, L type, alpha 1C subunit | CACNA1C | L29536 |  |  |
| 211578_s_at | ribosomal protein S6 kinase, 70kDa, polypeptide 1 | RPS6KB1 | M60725 | Hs.86858 | chr17q23.2 |
| 211574_s_at | CD46 molecule, complement regulatory protein | CD46 | D84105 | Hs.83532 | chr1q32 |
| 211569_s_at | hydroxyacyl-Coenzyme A dehydrogenase | HADH | AF001903 | Hs.8110 | chr4q22-q26 |
| 211559_s_at | cyclin G2 | CCNG2 | L49506 | Hs.13291 | chr4q21.1 |
| 211536_x_at | mitogen-activated protein kinase kinase kinase 7 | MAP3K7 | AB009358 | Hs.290346 | chr6q16.1-q16.3 |
| 211535_s_at | fibroblast growth factor receptor 1 (fms-related tyrosine kinase 2, Pfeiffer syndrome) | FGFR1 | M60485 | Hs.748 | chr8p11.2-p11.1 |
| 211530_x_at | HLA-G histocompatibility antigen, class I, G | HLA-G | M90686 | Hs.512152 | chr6p21.3 |
| 211506_s_at | interleukin 8 | IL8 | AF043337 | Hs.624 | chr4q13-q21 |
| 211504_x_at | Rho-associated, coiled-coil containing protein kinase 2 | ROCK2 | D87931 | Hs.58617 | chr2p24 |
| 211501_s_at | eukaryotic translation initiation factor 3, subunit 9 eta, 116kDa | EIF3S9 | BC001173 | Hs.371001 | chr7p22.3 |
| 211478_s_at | dipeptidyl-peptidase 4 (CD26, adenosine deaminase complexing protein 2) | DPP4 | M74777 | Hs.44926 | chr2q24.3 |
| 211474_s_at | serpin peptidase inhibitor, clade B (ovalbumin), member 6 | SERPINB6 | BC004948 | Hs.41072 | chr6p25 |
| 211471_s_at | RAB36, member RAS oncogene family | RAB36 | AF133588 | Hs.369557 | chr22q11.22 |
| 211456_x_at | metallothionein 1H-like protein | LOC645745 | AF333388 |  |  |
| 211450_s_at | mutS homolog 6 (E. coli) | MSH6 | D89646 | Hs.445052 | chr2p16 |
| 211445_x_at | nascent-polypeptide-associated complex alpha polypeptide pseudogene 1 /// similar to nascent polypeptide-associated complex alpha polypeptide | NACAP1 | AF315951 | Hs.458400 | chr8q22.3 |
| 211440_x_at | cytochrome P450, family 3, subfamily A, polypeptide 43 | CYP3A43 | AF280110 | Hs.306220 | chr7q21.1 |
| 211429_s_at | serpin peptidase inhibitor, clade A (alpha-1 antiproteinase, antitrypsin), member 1 | SERPINA1 | AF119873 | Hs.297681 | chr14q32.1 |
| 211423_s_at | sterol-C5-desaturase (ERG3 delta-5-desaturase homolog, fungal)-like | SC5DL | D85181 | Hs.287749 | chr11q23.3 |
| 211406_at | immediate early response 3 interacting protein 1 | IER3IP1 | AF119875 |  |  |
| 211387_x_at | RNA guanylyltransferase and 5'-phosphatase | RNGTT | AB012143 | Hs.27345 | chr6q16 |
| 211379_x_at | beta-1,3-N-acetylgalactosaminyltransferase 1 (globoside blood group) | B3GALNT1 | AB050855 | Hs.418062 | chr3q25 |
| 211376_s_at | non-SMC element 4 homolog A (S. cerevisiae) | NSMCE4A | BC005212 | Hs.258798 | chr10q26.13 |
| 211370_s_at | mitogen-activated protein kinase kinase 5 | MAP2K5 | U71088 | Hs.436145 | chr15q23 |
| 211366_x_at | caspase 1, apoptosis-related cysteine peptidase (interleukin 1, beta, convertase) | CASP1 | U13698 | Hs.2490 | chr11q23 |
| 211358_s_at | CDKN1A interacting zinc finger protein 1 | CIZ1 | AF234161 | Hs.23476 | chr9q34.1 |
| 211352_s_at | nuclear receptor coactivator 3 | NCOA3 | U80737 | Hs.382168 | chr20q12 |
| 211343_s_at | collagen, type XIII, alpha 1 | COL13A1 | M33653 | Hs.211933 | chr10q22 |
| 211339_s_at | IL2-inducible T-cell kinase | ITK | D13720 | Hs.211576 | chr5q31-q32 |
| 211337_s_at | gamma tubulin ring complex protein (76p gene) | 76P | BC000966 | Hs.290581 | chr15q15 |
| 211330_s_at | hemochromatosis | HFE | AF144242 | Hs.233325 | chr6p21.3 |
| 211325_x_at | destrin-2 pseudogene | LOC171220 | U72518 |  | chr12p13 |
| 211318_s_at | RAE1 RNA export 1 homolog (S. pombe) | RAE1 | U85943 | Hs.371698 | chr20q13.32 |
| 211297_s_at | cyclin-dependent kinase 7 (MO15 homolog, Xenopus laevis, cdk-activating kinase) | CDK7 | L20320 | Hs.184298 | chr5q12.1 |
| 211284_s_at | granulin | GRN | BC000324 | Hs.180577 | chr17q21.32 |
| 211277_x_at | amyloid beta (A4) precursor protein (peptidase nexin-II, Alzheimer disease) | APP | BC004369 | Hs.177486 | chr21q21.3 |
| 211276_at | transcription elongation factor A (SII)-like 2 | TCEAL2 | AF063606 | Hs.401835 | chrxq22.1-q22.3 |
| 211256_x_at | butyrophilin, subfamily 2, member A1 | BTN2A1 | U90142 | Hs.195683 | chr6p22.1 |
| 211241_at | annexin A2 pseudogene 3 | ANXA2P3 | M62895 |  |  |
| 211228_s_at | RAD17 homolog (S. pombe) | RAD17 | AF085736 | Hs.16184 | chr5q13 |
| 211212_s_at | origin recognition complex, subunit 5-like (yeast) | ORC5L | AF081459 | Hs.153138 | chr7q22.1 |
| 211168_s_at | UPF1 regulator of nonsense transcripts homolog (yeast) | UPF1 | D86988 | Hs.388125 | chr19p13.2-p13.11 |
| 211162_x_at | stearoyl-CoA desaturase (delta-9-desaturase) | SCD | AF116616 | Hs.119597 | chr10q23-q24 |
| 211160_x_at | actinin, alpha 1 | ACTN1 | M95178 | Hs.119000 | chr14q24 |
| 211153_s_at | tumor necrosis factor (ligand) superfamily, member 11 | TNFSF11 | AB037599 | Hs.333791 | chr13q14 |
| 211152_s_at | HtrA serine peptidase 2 | HTRA2 | AF184911 | Hs.115721 | chr2p12 |
| 211150_s_at | dihydrolipoamide S-acetyltransferase (E2 component of pyruvate dehydrogenase complex) | DLAT | J03866 | Hs.115285 | chr11q23.1 |
| 211126_s_at | cysteine and glycine-rich protein 2 | CSRP2 | U46006 | Hs.10526 | chr12q21.1 |
| 211114_x_at | survival of motor neuron protein interacting protein 1 | SIP1 | AB037702 | Hs.102456 | chr14q13 |
| 211110_s_at | androgen receptor (dihydrotestosterone receptor; testicular feminization; spinal and bulbar muscular atrophy; Kennedy disease) | AR | AF162704 | Hs.99915 | chrxq11.2-q12 |
| 211097_s_at | pre-B-cell leukemia transcription factor 2 | PBX2 | BC003111 | Hs.93728 | chr6p21.3 |
| 211090_s_at | PRP4 pre-mRNA processing factor 4 homolog B (yeast) /// PRP4 pre-mRNA processing factor 4 homolog B (yeast) | PRPF4B | Z25435 | Hs.198891 | chr6p25.2 |
| 211089_s_at | NIMA (never in mitosis gene a)-related kinase 3 /// NIMA (never in mitosis gene a)-related kinase 3 | NEK3 | Z25434 | Hs.2236 | chr13q14.13 |
| 211086_x_at | NIMA (never in mitosis gene a)-related kinase 1 /// NIMA (never in mitosis gene a)-related kinase 1 | NEK1 | Z25431 |  |  |
| 211081_s_at | mitogen-activated protein kinase kinase kinase kinase 5 /// mitogen-activated protein kinase kinase kinase kinase 5 | MAP4K5 | Z25426 | Hs.246970 | chr14q11.2-q21 |
| 211074_at | folate receptor 1 (adult) /// folate receptor 1 (adult) | FOLR1 | AF000381 |  |  |
| 211071_s_at | myeloid/lymphoid or mixed-lineage leukemia (trithorax homolog, Drosophila); translocated to, 11 /// myeloid/lymphoid or mixed-lineage leukemia (trithorax homolog, Drosophila); translocated to, 11 | MLLT11 | BC006471 | Hs.75823 | chr1q21 |
| 211068_x_at | family with sequence similarity 21, member C /// family with sequence similarity 21, member C /// hypothetical LOC653450 /// hypothetical LOC653450 | FAM21C | BC006456 | Hs.439367 | chr10q11.1 |
| 211065_x_at | phosphofructokinase, liver /// phosphofructokinase, liver | PFKL | BC006422 | Hs.368741 | chr21q22.3 |
| 211056_s_at | steroid-5-alpha-reductase, alpha polypeptide 1 (3-oxo-5 alpha-steroid delta 4-dehydrogenase alpha 1) /// steroid-5-alpha-reductase, alpha polypeptide 1 (3-oxo-5 alpha-steroid delta 4-dehydrogenase alpha 1) | SRD5A1 | BC006373 | Hs.552 | chr5p15 |
| 211040_x_at | G-2 and S-phase expressed 1 /// G-2 and S-phase expressed 1 | GTSE1 | BC006325 | Hs.122552 | chr22q13.2-q13.3 |
| 211038_s_at | ciliary rootlet coiled-coil, rootletin /// ciliary rootlet coiled-coil, rootletin /// hypothetical protein MGC12760 /// hypothetical protein MGC12760 /// similar to ciliary rootlet coiled-coil, rootletin /// similar to ciliary rootlet coiled-coil, rootlet | CROCC | BC006312 | Hs.301065 | chr1p36.13 |
| 211034_s_at | AF-1 specific protein phosphatase /// AF-1 specific protein phosphatase | FLJ30092 | BC006270 | Hs.506837 | chr12q24.13 |
| 211031_s_at | cytoplasmic linker 2 /// cytoplasmic linker 2 | CYLN2 | BC006259 | Hs.104717 | chr7q11.23 |
| 211026_s_at | monoglyceride lipase /// monoglyceride lipase | MGLL | BC006230 | Hs.409826 | chr3q21.3 |
| 211022_s_at | alpha thalassemia/mental retardation syndrome X-linked (RAD54 homolog, S. cerevisiae) | ATRX | BC002521 | Hs.440734 | chrxq13.1-q21.1 |
| 211019_s_at | lanosterol synthase (2,3-oxidosqualene-lanosterol cyclase) /// pericentriolar material 1 | LSS | D63807 | Hs.442223 | chr21q22.3 |
| 211016_x_at | heat shock 70kDa protein 4 | HSPA4 | BC002526 | Hs.90093 | chr5q31.1-q31.2 |
| 210997_at | hepatocyte growth factor (hepapoietin A; scatter factor) | HGF | M77227 | Hs.396530 | chr7q21.1 |
| 210995_s_at | tripartite motif-containing 23 | TRIM23 | AF230399 | Hs.792 | chr5q12.3 |
| 210993_s_at | SMAD family member 1 | SMAD1 | U54826 | Hs.357304 | chr4q31 |
| 210981_s_at | G protein-coupled receptor kinase 6 | GRK6 | AF040751 | Hs.235116 | chr5q35 |
| 210976_s_at | phosphofructokinase, muscle | PFKM | U24183 | Hs.75160 | chr12q13.3 |
| 210974_s_at | adaptor-related protein complex 3, delta 1 subunit | AP3D1 | AF130042 | Hs.512815 | chr19p13.3 |
| 210971_s_at | aryl hydrocarbon receptor nuclear translocator-like | ARNTL | AB000815 | Hs.65734 | chr11p15 |
| 210970_s_at | inhibitor of Bruton agammaglobulinemia tyrosine kinase | IBTK | AF235049 | Hs.306425 | chr6q14.1 |
| 210966_x_at | La ribonucleoprotein domain family, member 1 | LARP1 | BC001460 | Hs.6214 | chr5q33.2 |
| 210962_s_at | A kinase (PRKA) anchor protein (yotiao) 9 | AKAP9 | AB019691 | Hs.58103 | chr7q21-q22 |
| 210951_x_at | RAB27A, member RAS oncogene family | RAB27A | AF125393 | Hs.298530 | chr15q15-q21.1 |
| 210947_s_at | mutS homolog 3 (E. coli) | MSH3 | J04810 | Hs.42674 | chr5q11-q12 |
| 210933_s_at | fascin homolog 1, actin-bundling protein (Strongylocentrotus purpuratus) | FSCN1 | BC004908 | Hs.118400 | chr7p22 |
| 210926_at | actin-like protein | FKSG30 | AY014272 | Hs.528531 | chr2q21.1 |
| 210912_x_at | glutathione S-transferase M4 | GSTM4 | M99422 | Hs.348387 | chr1p13.3 |
| 210910_s_at | POM (POM121 homolog, rat) and ZP3 fusion | POMZP3 | BC000487 | Hs.296380 | chr7q11.23 |
| 210907_s_at | programmed cell death 10 | PDCD10 | BC002506 | Hs.424619 | chr3q26.1 |
| 210886_x_at | TP53 activated protein 1 | TP53AP1 | AB007457 | Hs.274329 | chr7q21.1 |
| 210883_x_at | ephrin-B3 | EFNB3 | U57001 | Hs.26988 | chr17p13.1-p11.2 |
| 210879_s_at | RAB11 family interacting protein 5 (class I) | RAB11FIP5 | AF334812 | Hs.24557 | chr2p13-p12 |
| 210876_at | annexin A2 pseudogene 1 | ANXA2P1 | M62896 | Hs.462864 | chr15q21-q22 |
| 210871_x_at | synovial sarcoma, X breakpoint 2 interacting protein | SSX2IP | AL133046 | Hs.22587 | chr1p22.3 |
| 210868_s_at | ELOVL family member 6, elongation of long chain fatty acids (FEN1/Elo2, SUR4/Elo3-like, yeast) | ELOVL6 | BC001305 | Hs.211556 | chr4q25 |
| 210852_s_at | aminoadipate-semialdehyde synthase | AASS | AF229180 | Hs.528295 | chr7q31.3 |
| 210830_s_at | paraoxonase 2 | PON2 | AF001602 | Hs.165598 | chr7q21.3 |
| 210820_x_at | coenzyme Q7 homolog, ubiquinone (yeast) | COQ7 | AL136647 | Hs.157113 | chr16p13.11-p12.3 |
| 210817_s_at | calcium binding and coiled-coil domain 2 | CALCOCO2 | BC004130 | Hs.154230 | chr17q21.32 |
| 210816_s_at | cytochrome b-561 | CYB561 | BC000021 | Hs.355264 | chr17q11-qter |
| 210811_s_at | DEAD (Asp-Glu-Ala-Asp) box polypeptide 49 | DDX49 | BC000979 | Hs.143187 | chr19p12 |
| 210807_s_at | solute carrier family 16, member 7 (monocarboxylic acid transporter 2) | SLC16A7 | AF049608 | Hs.439643 | chr12q13 |
| 210802_s_at | DIM1 dimethyladenosine transferase 1-like (S. cerevisiae) | DIMT1L | BC002841 | Hs.113319 | chr5q11-q14 |
| 210792_x_at | SIVA1, apoptosis-inducing factor | SIVA1 | AF033111 | Hs.112058 | chr14q32.33 |
| 210791_s_at | Rho GTPase-activating protein | RICS | BC000277 | Hs.440379 | chr11q24-q25 |
| 210788_s_at | dehydrogenase/reductase (SDR family) member 7 | DHRS7 | AF126782 | Hs.36049 | chr14q23.1 |
| 210776_x_at | transcription factor 3 (E2A immunoglobulin enhancer binding factors E12/E47) | TCF3 | M31222 | Hs.371282 | chr19p13.3 |
| 210775_x_at | caspase 9, apoptosis-related cysteine peptidase | CASP9 | AB015653 | Hs.329502 | chr1p36.3-p36.1 |
| 210774_s_at | nuclear receptor coactivator 4 | NCOA4 | AL162047 | Hs.422334 | chr10q11.2 |
| 210758_at | PC4 and SFRS1 interacting protein 1 | PSIP1 | AF098482 | Hs.142498 | chr9p22.3 |
| 210756_s_at | Notch homolog 2 (Drosophila) /// Notch homolog 2 (Drosophila) | NOTCH2 | AF308601 | Hs.434872 | chr1p13-p11 |
| 210753_s_at | EPH receptor B1 | EPHB1 | AF037334 | Hs.272311 | chr3q21-q23 |
| 210751_s_at | regucalcin (senescence marker protein-30) | RGN | D31815 | Hs.77854 | chrxp11.3 |
| 210740_s_at | inositol 1,3,4-triphosphate 5/6 kinase | ITPK1 | AF279372 | Hs.408429 | chr14q31 |
| 210735_s_at | carbonic anhydrase XII | CA12 | BC000278 | Hs.279916 | chr15q22 |
| 210732_s_at | lectin, galactoside-binding, soluble, 8 (galectin 8) | LGALS8 | AF342816 | Hs.4082 | chr1q42-q43 |
| 210723_x_at | hypothetical protein MGC4771 | MGC4771 | BC004917 | Hs.326743 | chr2q37.1 |
| 210721_s_at | p21(CDKN1A)-activated kinase 7 | PAK7 | AB040812 | Hs.32539 | chr20p12 |
| 210720_s_at | amyloid beta (A4) precursor protein-binding, family A, member 2 binding protein | APBA2BP | AB039947 | Hs.324104 | chr20q11.22 |
| 210715_s_at | serine peptidase inhibitor, Kunitz type, 2 | SPINT2 | AF027205 | Hs.31439 | chr19q13.1 |
| 210711_at | hypothetical protein MGC5457 | MGC5457 | BC000988 |  | chr1q32.1 |
| 210710_at | angiogenic factor with G patch and FHA domains 1 | AGGF1 | BC002828 | Hs.284216 | chr5q13.3 |
| 210707_x_at | postmeiotic segregation increased 2-like 11 | PMS2L11 | U38980 |  | chr7q |
| 210705_s_at | tripartite motif-containing 5 | TRIM5 | AF220028 | Hs.370515 | chr11p15 |
| 210692_s_at | solute carrier family 43, member 3 | SLC43A3 | BC003163 | Hs.99962 | chr11q11 |
| 210691_s_at | calcyclin binding protein | CACYBP | AF275803 | Hs.27258 | chr1q24-q25 |
| 210667_s_at | chromosome 21 open reading frame 33 | C21orf33 | D86062 | Hs.413482 | chr21q22.3 |
| 210650_s_at | piccolo (presynaptic cytomatrix protein) | PCLO | BC001304 |  |  |
| 210649_s_at | AT rich interactive domain 1A (SWI-like) | ARID1A | AF231056 | Hs.170333 | chr1p35.3 |
| 210647_x_at | phospholipase A2, group VI (cytosolic, calcium-independent) | PLA2G6 | AF102988 | Hs.309169 | chr22q13.1 |
| 210635_s_at | kelch-like 20 (Drosophila) | KLHL20 | BC005253 | Hs.106290 | chr1q24.1-q24.3 |
| 210622_x_at | cyclin-dependent kinase (CDC2-like) 10 | CDK10 | AF153430 | Hs.77313 | chr16q24 |
| 210609_s_at | tumor protein p53 inducible protein 3 | TP53I3 | BC000474 | Hs.50649 | chr2p23.3 |
| 210608_s_at | fucosyltransferase 2 (secretor status included) | FUT2 | BC001899 | Hs.132470 | chr19q13.3 |
| 210605_s_at | milk fat globule-EGF factor 8 protein | MFGE8 | BC003610 | Hs.3745 | chr15q25 |
| 210594_x_at | myelin protein zero-like 1 | MPZL1 | AF239756 | Hs.14891 | chr1q24.2 |
| 210589_s_at | glucosidase, beta; acid (includes glucosylceramidase) /// glucosidase, beta; acid, pseudogene | GBA | D13287 | Hs.282997 | chr1q21 |
| 210573_s_at | polymerase (RNA) III (DNA directed) polypeptide C (62kD) | POLR3C | BC004424 | Hs.250745 | chr1q21.1 |
| 210555_s_at | nuclear factor of activated T-cells, cytoplasmic, calcineurin-dependent 3 | NFATC3 | U85430 | Hs.172674 | chr16q22.2 |
| 210544_s_at | aldehyde dehydrogenase 3 family, member A2 | ALDH3A2 | BC002430 | Hs.440662 | chr17p11.2 |
| 210543_s_at | protein kinase, DNA-activated, catalytic polypeptide | PRKDC | U34994 | Hs.415749 | chr8q11 |
| 210542_s_at | solute carrier organic anion transporter family, member 3A1 | SLCO3A1 | BC000585 | Hs.356925 | chr15q26 |
| 210541_s_at | tripartite motif-containing 27 | TRIM27 | AF230394 | Hs.440382 | chr6p22 |
| 210538_s_at | baculoviral IAP repeat-containing 3 | BIRC3 | U37546 | Hs.127799 | chr11q22 |
| 210534_s_at | B9 protein | EPPB9 | BC002944 | Hs.424099 | chr17p11.2 |
| 210527_x_at | tubulin, alpha 2 | TUBA2 | L11645 | Hs.349695 | chr13q11 |
| 210513_s_at | vascular endothelial growth factor A | VEGFA | AF091352 | Hs.73793 | chr6p12 |
| 210501_x_at | eukaryotic translation initiation factor 3, subunit 12 | EIF3S12 | AF119846 | Hs.143773 | chr19q13.2 |
| 210473_s_at | G protein-coupled receptor 125 | GPR125 | M37712 | Hs.356876 | chr4p15.31 |
| 210458_s_at | TRAF family member-associated NFKB activator | TANK | BC003388 | Hs.123294 | chr2q24-q31 |
| 210449_x_at | mitogen-activated protein kinase 14 | MAPK14 | AF100544 | Hs.79107 | chr6p21.3-p21.2 |
| 210445_at | fatty acid binding protein 6, ileal (gastrotropin) | FABP6 | U19869 | Hs.147391 | chr5q33.3-q34 |
| 210428_s_at | hepatocyte growth factor-regulated tyrosine kinase substrate | HGS | AF260566 | Hs.416959 | chr17q25 |
| 210418_s_at | isocitrate dehydrogenase 3 (NAD+) beta | IDH3B | AF023265 | Hs.436405 | chr20p13 |
| 210417_s_at | phosphatidylinositol 4-kinase, catalytic, beta polypeptide | PIK4CB | U81802 | Hs.154846 | chr1q21 |
| 210385_s_at | type 1 tumor necrosis factor receptor shedding aminopeptidase regulator | ARTS-1 | AF106037 | Hs.436186 | chr5q15 |
| 210381_s_at | cholecystokinin B receptor | CCKBR | BC000740 | Hs.203 | chr11p15.4 |
| 210379_s_at | tousled-like kinase 1 | TLK1 | AF162666 | Hs.369280 | chr2q31.1 |
| 210378_s_at | Sjogren's syndrome nuclear autoantigen 1 | SSNA1 | BC004118 | Hs.18528 | chr9q34.3 |
| 210377_at | acyl-CoA synthetase medium-chain family member 3 | ACSM3 | D16350 | Hs.512678 | chr16p13.11 |
| 210371_s_at | retinoblastoma binding protein 4 | RBBP4 | BC003092 | Hs.348415 | chr1p35.1 |
| 210361_s_at | E74-like factor 2 (ets domain transcription factor) | ELF2 | AF256223 | Hs.82143 | chr4q28 |
| 210357_s_at | spermine oxidase | SMOX | BC000669 | Hs.433337 | chr20p13 |
| 210355_at | parathyroid hormone-like hormone | PTHLH | J03580 | Hs.89626 | chr12p12.1-p11.2 |
| 210337_s_at | ATP citrate lyase | ACLY | U18197 | Hs.387567 | chr17q12-q21 |
| 210317_s_at | tyrosine 3-monooxygenase/tryptophan 5-monooxygenase activation protein, epsilon polypeptide | YWHAE | U28936 | Hs.79474 | chr17p13.3 |
| 210312_s_at | intraflagellar transport 20 homolog (Chlamydomonas) | IFT20 | BC002640 | Hs.4187 | chr17q11.2 |
| 210296_s_at | peroxisomal membrane protein 3, 35kDa (Zellweger syndrome) | PXMP3 | BC005375 | Hs.180612 | chr8q21.1 |
| 210290_at | zinc finger protein 174 | ZNF174 | BC001161 | Hs.155204 | chr16p13.3 |
| 210284_s_at | mitogen-activated protein kinase kinase kinase 7 interacting protein 2 | MAP3K7IP2 | AF241230 | Hs.486780 | chr6q25.1-q25.3 |
| 210281_s_at | zinc finger, MYM-type 2 | ZMYM2 | AL136621 | Hs.315241 | chr13q11-q12 |
| 210275_s_at | zinc finger, AN1-type domain 5 | ZFAND5 | AF062347 | Hs.406096 | chr9q13-q21 |
| 210253_at | HIV-1 Tat interactive protein 2, 30kDa | HTATIP2 | AF092095 | Hs.90753 | chr11p15.1 |
| 210252_s_at | MAP-kinase activating death domain | MADD | AB002356 | Hs.82548 | chr11p11.2 |
| 210243_s_at | UDP-Gal:betaGlcNAc beta 1,4- galactosyltransferase, polypeptide 3 | B4GALT3 | AF038661 | Hs.321231 | chr1q21-q23 |
| 210239_at | iroquois homeobox protein 5 | IRX5 | U90304 | Hs.25351 | chr16q11.2-q13 |
| 210230_at | Hypothetical protein LOC728965 | LOC728965 | BC003629 | Hs.536594 |  |
| 210224_at | major histocompatibility complex, class I-related | MR1 | AF031469 | Hs.434917 | chr1q25.3 |
| 210213_s_at | integrin beta 4 binding protein | ITGB4BP | AF022229 | Hs.5215 | chr20q12 |
| 210211_s_at | heat shock protein 90kDa alpha (cytosolic), class A member 1 | HSP90AA1 | AF028832 | Hs.446579 | chr14q32.33 |
| 210205_at | UDP-Gal:betaGlcNAc beta 1,3-galactosyltransferase, polypeptide 4 | B3GALT4 | AB026730 | Hs.534404 | chr6p21.3 |
| 210198_s_at | proteolipid protein 1 (Pelizaeus-Merzbacher disease, spastic paraplegia 2, uncomplicated) | PLP1 | BC002665 | Hs.1787 | chrxq22 |
| 210180_s_at | splicing factor, arginine/serine-rich 10 (transformer 2 homolog, Drosophila) | SFRS10 | U87836 | Hs.30035 | chr3q26.2-q27 |
| 210178_x_at | FUS interacting protein (serine/arginine-rich) 1 /// similar to FUS-interacting serine-arginine-rich protein 1 (TLS-associated protein with Ser-Arg repeats) (TLS-associated protein with SR repeats) (TASR) (TLS-associated serine-arginine protein) (TLS-asso | FUSIP1 | AF047448 | Hs.3530 | chr1p36.11 |
| 210172_at | splicing factor 1 | SF1 | D26121 | Hs.440835 | chr11q13 |
| 210160_at | platelet-activating factor acetylhydrolase, isoform Ib, beta subunit 30kDa | PAFAH1B2 | BC000398 | Hs.93354 | chr11q23 |
| 210156_s_at | protein-L-isoaspartate (D-aspartate) O-methyltransferase | PCMT1 | D25547 | Hs.79137 | chr6q24-q25 |
| 210149_s_at | ATP synthase, H+ transporting, mitochondrial F0 complex, subunit d | ATP5H | AF061735 | Hs.155728 | chr17q25 |
| 210145_at | phospholipase A2, group IVA (cytosolic, calcium-dependent) | PLA2G4A | M68874 | Hs.211587 | chr1q25 |
| 210144_at | TBC1 domain family, member 22A | TBC1D22A | AK000851 | Hs.505862 | chr22q13.3 |
| 210139_s_at | peripheral myelin protein 22 | PMP22 | L03203 | Hs.372031 | chr17p12-p11.2 |
| 210135_s_at | short stature homeobox 2 | SHOX2 | AF022654 | Hs.55967 | chr3q25-q26.1 |
| 210130_s_at | transmembrane 7 superfamily member 2 | TM7SF2 | AF096304 | Hs.31130 | chr11q13 |
| 210127_at | RAB6B, member RAS oncogene family | RAB6B | BC002510 | Hs.352530 | chr3q22.1 |
| 210125_s_at | barrier to autointegration factor 1 | BANF1 | AF044773 | Hs.433759 | chr11q13.1 |
| 210115_at | ribosomal protein L39-like | RPL39L | L05096 | Hs.132748 | chr3q27 |
| 210114_at | inversin | INVS | AF039217 | Hs.150744 | chr9q31 |
| 210112_at | Hermansky-Pudlak syndrome 1 | HPS1 | U96721 | Hs.404568 | chr10q23.1-q23.3 |
| 210110_x_at | heterogeneous nuclear ribonucleoprotein H3 (2H9) | HNRPH3 | AF132363 | Hs.156481 | chr10q22 |
| 210092_at | mago-nashi homolog, proliferation-associated (Drosophila) | MAGOH | AF067173 | Hs.421576 | chr1p34-p33 |
| 210076_x_at | SERPINE1 mRNA binding protein 1 | SERBP1 | AF151813 | Hs.356427 | chr1p31-p22 |
| 210075_at | membrane-associated ring finger (C3HC4) 2 | 02-mars | AF151074 | Hs.331308 | chr19p13.2 |
| 210074_at | cathepsin L2 | CTSL2 | AF070448 | Hs.87417 | chr9q22.2 |
| 210069_at | choline kinase beta /// carnitine palmitoyltransferase 1B (muscle) /// Rho GTPase activating protein 29 | CHKB | U62733 | Hs.439777 | chr22q13.33 |
| 210059_s_at | mitogen-activated protein kinase 13 | MAPK13 | BC000433 | Hs.178695 | chr6p21.31 |
| 210057_at | PI-3-kinase-related kinase SMG-1 | SMG1 | U32581 | Hs.212761 | chr16p12.3 |
| 210054_at | chromosome 4 open reading frame 15 | C4orf15 | BC003648 | Hs.421054 | chr4p16.3 |
| 210050_at | triosephosphate isomerase 1 | TPI1 | M10036 |  |  |
| 210048_at | N-ethylmaleimide-sensitive factor attachment protein, gamma | NAPG | BC001889 | Hs.370431 | chr18p11.22 |
| 210046_s_at | isocitrate dehydrogenase 2 (NADP+), mitochondrial | IDH2 | U52144 | Hs.5337 | chr15q26.1 |
| 210042_s_at | cathepsin Z | CTSZ | AF073890 | Hs.252549 | chr20q13 |
| 210041_s_at | phosphoglucomutase 3 | PGM3 | BC001258 | Hs.237323 | chr6q14.1-q15 |
| 210036_s_at | potassium voltage-gated channel, subfamily H (eag-related), member 2 | KCNH2 | AB044806 | Hs.188021 | chr7q35-q36 |
| 210028_s_at | origin recognition complex, subunit 3-like (yeast) | ORC3L | AF125507 | Hs.312419 | chr6q14.3-q16.1 |
| 210027_s_at | APEX nuclease (multifunctional DNA repair enzyme) 1 | APEX1 | M80261 | Hs.73722 | chr14q11.2-q12 |
| 210026_s_at | caspase recruitment domain family, member 10 | CARD10 | AY028896 | Hs.57973 | chr22q13.1 |
| 210024_s_at | ubiquitin-conjugating enzyme E2E 3 (UBC4/5 homolog, yeast) | UBE2E3 | AB017644 | Hs.4890 | chr2q32.1 |
| 210023_s_at | polycomb group ring finger 1 | PCGF1 | BC004952 | Hs.316750 | chr2p13.1 |
| 210015_s_at | microtubule-associated protein 2 | MAP2 | U89330 | Hs.167 | chr2q34-q35 |
| 210011_s_at | Ewing sarcoma breakpoint region 1 | EWSR1 | BC000527 | Hs.374477 | chr22q12.2 |
| 210010_s_at | solute carrier family 25 (mitochondrial carrier; citrate transporter), member 1 | SLC25A1 | U25147 | Hs.111024 | chr22q11.21 |
| 210007_s_at | glycerol-3-phosphate dehydrogenase 2 (mitochondrial) | GPD2 | U36310 | Hs.108646 | chr2q24.1 |
| 210006_at | abhydrolase domain containing 14A | ABHD14A | BC002571 | Hs.534449 | chr3p21.1 |
| 210001_s_at | suppressor of cytokine signaling 1 | SOCS1 | AB005043 | Hs.50640 | chr16p13.13 |
| 209998_at | phosphatidylinositol glycan anchor biosynthesis, class O | PIGO | BC001030 | Hs.213029 | chr9p13.3 |
| 209990_s_at | gamma-aminobutyric acid (GABA) B receptor, 2 | GABBR2 | AF056085 | Hs.198612 | chr9q22.1-q22.3 |
| 209984_at | jumonji domain containing 2C | JMJD2C | AB037901 | Hs.157106 | chr9p24-p23 |
| 209981_at | cold shock domain containing C2, RNA binding | CSDC2 | AL023553 | Hs.106635 | chr22q13.2-q13.31 |
| 209971_x_at | JTV1 gene | JTV1 | AI928526 | Hs.534395 | chr7p22 |
| 209967_s_at | cAMP responsive element modulator | CREM | D14826 | Hs.231975 | chr10p11.21 |
| 209964_s_at | ataxin 7 | ATXN7 | AF032105 | Hs.108447 | chr3p21.1-p12 |
| 209953_s_at | cell division cycle 37 homolog (S. cerevisiae) | CDC37 | U63131 | Hs.160958 | chr19p13.2 |
| 209951_s_at | mitogen-activated protein kinase kinase 7 | MAP2K7 | AW007458 | Hs.110299 | chr19p13.3-p13.2 |
| 209946_at | vascular endothelial growth factor C | VEGFC | U58111 | Hs.79141 | chr4q34.1-q34.3 |
| 209945_s_at | glycogen synthase kinase 3 beta | GSK3B | BC000251 | Hs.282359 | chr3q13.3 |
| 209929_s_at | inhibitor of kappa light polypeptide gene enhancer in B-cells, kinase gamma | IKBKG | AF091453 | Hs.43505 | chrxq28 |
| 209925_at | occludin /// similar to Occludin | OCLN | U53823 | Hs.171952 | chr5q13.1 |
| 209921_at | solute carrier family 7, (cationic amino acid transporter, y+ system) member 11 | SLC7A11 | AB040875 | Hs.480754 | chr4q28-q32 |
| 209916_at | dehydrogenase E1 and transketolase domain containing 1 | DHTKD1 | BC002477 | Hs.501565 | chr10p14 |
| 209912_s_at | KIAA0415 protein | KIAA0415 | AI373854 | Hs.416930 | chr7p22.1 |
| 209907_s_at | intersectin 2 | ITSN2 | AF182198 | Hs.444708 | chr2pter-p25.1 |
| 209902_at | ataxia telangiectasia and Rad3 related /// similar to ataxia telangiectasia and Rad3 related protein | ATR | U49844 | Hs.77613 | chr3q22-q24 |
| 209899_s_at | fuse-binding protein-interacting repressor | SIAHBP1 | AF217197 | Hs.74562 | chr8q24.2-qtel |
| 209897_s_at | slit homolog 2 (Drosophila) | SLIT2 | AF055585 | Hs.29802 | chr4p15.2 |
| 209895_at | protein tyrosine phosphatase, non-receptor type 11 (Noonan syndrome 1) | PTPN11 | AF119855 | Hs.83572 | chr12q24 |
| 209894_at | leptin receptor | LEPR | U50748 | Hs.23581 | chr1p31 |
| 209893_s_at | fucosyltransferase 4 (alpha (1,3) fucosyltransferase, myeloid-specific) | FUT4 | M58596 | Hs.390420 | chr11q21 |
| 209885_at | ras homolog gene family, member D | RHOD | BC001338 | Hs.15114 | chr11q14.3 |
| 209884_s_at | solute carrier family 4, sodium bicarbonate cotransporter, member 7 | SLC4A7 | AF047033 | Hs.250072 | chr3p22 |
| 209876_at | G protein-coupled receptor kinase interactor 2 | GIT2 | AF124491 | Hs.418057 | chr12q24.1 |
| 209867_s_at | latrophilin 3 | LPHN3 | AF307080 | Hs.21917 | chr4q13.1 |
| 209865_at | solute carrier family 35 (UDP-N-acetylglucosamine (UDP-GlcNAc) transporter), member A3 | SLC35A3 | BC005136 | Hs.28803 | chr1p21 |
| 209864_at | frequently rearranged in advanced T-cell lymphomas 2 | FRAT2 | AB045118 | Hs.140720 | chr10q24.1 |
| 209846_s_at | butyrophilin, subfamily 3, member A2 | BTN3A2 | BC002832 | Hs.376046 | chr6p22.1 |
| 209838_at | COP9 constitutive photomorphogenic homolog subunit 2 (Arabidopsis) | COPS2 | AA496247 | Hs.30212 | chr15q21.2 |
| 209834_at | carbohydrate (chondroitin 6) sulfotransferase 3 | CHST3 | AB017915 | Hs.158304 | chr10q22.1 |
| 209833_at | CASP2 and RIPK1 domain containing adaptor with death domain | CRADD | U79115 | Hs.155566 | chr12q21.33-q23.1 |
| 209825_s_at | uridine-cytidine kinase 2 | UCK2 | BC002906 | Hs.458360 | chr1q23 |
| 209822_s_at | very low density lipoprotein receptor | VLDLR | L22431 | Hs.370422 | chr9p24 |
| 209821_at | interleukin 33 | IL33 | AB024518 | Hs.348390 | chr9p24.1 |
| 209820_s_at | transducin (beta)-like 3 | TBL3 | BC002361 | Hs.114416 | chr16p13.3 |
| 209815_at | patched homolog 1 (Drosophila) | PTCH1 | BG054916 | Hs.454253 | chr9q22.3 |
| 209814_at | zinc finger protein 330 | ZNF330 | BC004421 | Hs.120766 | chr4q31.1-q31.2 |
| 209808_x_at | inhibitor of growth family, member 1 | ING1 | AW193656 | Hs.46700 | chr13q34 |
| 209803_s_at | pleckstrin homology-like domain, family A, member 2 | PHLDA2 | AF001294 | Hs.154036 | chr11p15.5 |
| 209798_at | nuclear protein, ataxia-telangiectasia locus | NPAT | D83243 | Hs.89385 | chr11q22-q23 |
| 209797_at | transmembrane protein 4 | TMEM4 | BC001027 | Hs.8752 | chr12q15 |
| 209790_s_at | caspase 6, apoptosis-related cysteine peptidase | CASP6 | BC000305 | Hs.3280 | chr4q25 |
| 209780_at | putative homeodomain transcription factor 2 | PHTF2 | AL136883 | Hs.128653 | chr7q11.23-q21 |
| 209778_at | thyroid hormone receptor interactor 11 | TRIP11 | AF007217 | Hs.156892 | chr14q31-q32 |
| 209774_x_at | chemokine (C-X-C motif) ligand 2 | CXCL2 | M57731 | Hs.75765 | chr4q21 |
| 209772_s_at | CD24 molecule | CD24 | X69397 | Hs.375108 | chr6q21 |
| 209770_at | butyrophilin, subfamily 3, member A1 | BTN3A1 | U90552 | Hs.284283 | chr6p22.1 |
| 209760_at | KIAA0922 | KIAA0922 | AL136932 | Hs.511944 | chr4q31.3 |
| 209759_s_at | dodecenoyl-Coenzyme A delta isomerase (3,2 trans-enoyl-Coenzyme A isomerase) | DCI | BC002746 | Hs.403436 | chr16p13.3 |
| 209753_s_at | thymopoietin | TMPO | BG391171 | Hs.11355 | chr12q22 |
| 209743_s_at | itchy homolog E3 ubiquitin protein ligase (mouse) | ITCH | AI989677 | Hs.314676 | chr20q11.22-q11.23 |
| 209739_s_at | patatin-like phospholipase domain containing 4 | PNPLA4 | AI814551 | Hs.264 | chrxp22.3 |
| 209737_at | membrane associated guanylate kinase, WW and PDZ domain containing 2 | MAGI2 | AB014605 | Hs.22599 | chr7q21 |
| 209732_at | C-type lectin domain family 2, member B /// CMT1A duplicated region transcript 15 pseudogene | CLEC2B | BC005254 | Hs.85201 | chr12p13-p12 |
| 209725_at | UTP20, small subunit (SSU) processome component, homolog (yeast) /// similar to Down-regulated in metastasis protein (Key-1A6 protein) (Novel nucleolar protein 73) (NNP73) | UTP20 | AF072718 | Hs.295732 | chr12q23 |
| 209723_at | serpin peptidase inhibitor, clade B (ovalbumin), member 9 | SERPINB9 | BC002538 | Hs.104879 | chr6p25 |
| 209715_at | chromobox homolog 5 (HP1 alpha homolog, Drosophila) | CBX5 | L07515 | Hs.389182 | chr12q13.13 |
| 209714_s_at | cyclin-dependent kinase inhibitor 3 (CDK2-associated dual specificity phosphatase) | CDKN3 | AF213033 | Hs.84113 | chr14q22 |
| 209713_s_at | solute carrier family 35 (UDP-glucuronic acid/UDP-N-acetylgalactosamine dual transporter), member D1 | SLC35D1 | AB044343 | Hs.82635 | chr1p32-p31 |
| 209706_at | NK3 transcription factor related, locus 1 (Drosophila) | NKX3-1 | AF247704 | Hs.55999 | chr8p21 |
| 209703_x_at | methyltransferase like 7A | METTL7A | BC004492 | Hs.288771 | chr12q13.12 |
| 209702_at | fatso | FTO | U79260 | Hs.284741 | chr16q12.2 |
| 209694_at | 6-pyruvoyltetrahydropterin synthase | PTS | M97655 | Hs.415877 | chr11q22.3-q23.3 |
| 209693_at | astrotactin 2 | ASTN2 | AF116574 | Hs.128305 | chr9q33.1 |
| 209691_s_at | docking protein 4 | DOK4 | BC003541 | Hs.534537 | chr16q13 |
| 209689_at | coiled-coil domain containing 93 | CCDC93 | BC005078 | Hs.98324 | chr2q14.1 |
| 209686_at | S100 calcium binding protein B | S100B | BC001766 | Hs.422181 | chr21q22.3 |
| 209685_s_at | protein kinase C, beta 1 | PRKCB1 | M13975 | Hs.349845 | chr16p11.2 |
| 209684_at | Ras and Rab interactor 2 | RIN2 | AL136924 | Hs.446304 | chr20p11.22 |
| 209683_at | family with sequence similarity 49, member A | FAM49A | AA243659 |  |  |
| 209682_at | Cas-Br-M (murine) ecotropic retroviral transforming sequence b | CBLB | U26710 | Hs.436986 | chr3q13.11 |
| 209681_at | solute carrier family 19 (thiamine transporter), member 2 | SLC19A2 | AF153330 | Hs.30246 | chr1q23.3 |
| 209679_s_at | small trans-membrane and glycosylated protein | LOC57228 | BC003379 | Hs.505572 | chr12q13.13 |
| 209677_at | protein kinase C, iota | PRKCI | L18964 | Hs.496511 | chr3q26.3 |
| 209675_s_at | heterogeneous nuclear ribonucleoprotein U-like 1 | HNRPUL1 | BC004242 | Hs.155218 | chr19q13.2 |
| 209674_at | cryptochrome 1 (photolyase-like) | CRY1 | D83702 | Hs.151573 | chr12q23-q24.1 |
| 209666_s_at | conserved helix-loop-helix ubiquitous kinase | CHUK | AF080157 | Hs.198998 | chr10q24-q25 |
| 209665_at | cytochrome b-561 domain containing 2 | CYB561D2 | AF040704 | Hs.149443 | chr3p21.3 |
| 209662_at | centrin, EF-hand protein, 3 (CDC31 homolog, yeast) | CETN3 | BC005383 | Hs.528302 | chr5q14.3 |
| 209657_s_at | heat shock transcription factor 2 | HSF2 | M65217 | Hs.158195 | chr6q22.31 |
| 209655_s_at | transmembrane protein 47 | TMEM47 | AI803181 | Hs.8769 | chrxp11.4 |
| 209653_at | karyopherin alpha 4 (importin alpha 3) | KPNA4 | U93240 | Hs.288193 | chr3q25.33 |
| 209652_s_at | placental growth factor, vascular endothelial growth factor-related protein | PGF | BC001422 | Hs.252820 | chr14q24-q31 |
| 209651_at | transforming growth factor beta 1 induced transcript 1 | TGFB1I1 | BC001830 | Hs.25511 | chr16p11.2 |
| 209646_x_at | aldehyde dehydrogenase 1 family, member B1 | ALDH1B1 | BC001619 | Hs.436219 | chr9p11.1 |
| 209642_at | BUB1 budding uninhibited by benzimidazoles 1 homolog (yeast) | BUB1 | AF043294 | Hs.287472 | chr2q14 |
| 209633_at | protein phosphatase 2 (formerly 2A), regulatory subunit B'', alpha | PPP2R3A | AL389975 | Hs.133234 | chr3q22.1 |
| 209631_s_at | G protein-coupled receptor 37 (endothelin receptor type B-like) | GPR37 | U87460 | Hs.406094 | chr7q31 |
| 209628_at | nuclear transport factor 2-like export factor 2 | NXT2 | AK023289 | Hs.25010 | chrxq23 |
| 209625_at | phosphatidylinositol glycan anchor biosynthesis, class H | PIGH | BC004100 | Hs.177 | chr14q11-q24 |
| 209623_at | methylcrotonoyl-Coenzyme A carboxylase 2 (beta) | MCCC2 | AW439494 | Hs.167531 | chr5q12-q13 |
| 209621_s_at | PDZ and LIM domain 3 | PDLIM3 | AF002280 | Hs.71719 | chr4q35 |
| 209620_s_at | ATP-binding cassette, sub-family B (MDR/TAP), member 7 | ABCB7 | AB005289 | Hs.370480 | chrxq12-q13 |
| 209619_at | CD74 molecule, major histocompatibility complex, class II invariant chain | CD74 | K01144 | Hs.446471 | chr5q32 |
| 209615_s_at | p21/Cdc42/Rac1-activated kinase 1 (STE20 homolog, yeast) | PAK1 | U51120 | Hs.64056 | chr11q13-q14 |
| 209608_s_at | acetyl-Coenzyme A acetyltransferase 2 (acetoacetyl Coenzyme A thiolase) | ACAT2 | BC000408 | Hs.4112 | chr6q25.3-q26 |
| 209607_x_at | sulfotransferase family, cytosolic, 1A, phenol-preferring, member 3 /// sulfotransferase family, cytosolic, 1A, phenol-preferring, member 4 | SULT1A3 | U08032 | Hs.499044 | chr16p11.2 |
| 209605_at | thiosulfate sulfurtransferase (rhodanese) | TST | D87292 | Hs.351863 | chr22q13.1 |
| 209595_at | general transcription factor IIF, polypeptide 2, 30kDa | GTF2F2 | BC001771 | Hs.58593 | chr13q14 |
| 209593_s_at | torsin family 1, member B (torsin B) | TOR1B | AF317129 | Hs.252682 | chr9q34 |
| 209586_s_at | prune homolog (Drosophila) | PRUNE | AF123539 | Hs.78524 | chr1q21 |
| 209585_s_at | multiple inositol polyphosphate histidine phosphatase, 1 | MINPP1 | AF084943 | Hs.95907 | chr10q23 |
| 209584_x_at | apolipoprotein B mRNA editing enzyme, catalytic polypeptide-like 3C | APOBEC3C | AF165520 | Hs.441124 | chr22q13.1-q13.2 |
| 209582_s_at | CD200 molecule | CD200 | H23979 | Hs.79015 | chr3q12-q13 |
| 209581_at | HRAS-like suppressor 3 | HRASLS3 | BC001387 | Hs.528308 | chr11q12.3-q13.1 |
| 209579_s_at | methyl-CpG binding domain protein 4 | MBD4 | AL556619 | Hs.35947 | chr3q21-q22 |
| 209578_s_at | protein O-fucosyltransferase 2 | POFUT2 | BC000626 | Hs.22982 | chr21q22.3 |
| 209577_at | phosphate cytidylyltransferase 2, ethanolamine | PCYT2 | BC000351 | Hs.534363 | chr17q25.3 |
| 209576_at | guanine nucleotide binding protein (G protein), alpha inhibiting activity polypeptide 1 | GNAI1 | AL049933 | Hs.203862 | chr7q21 |
| 209572_s_at | embryonic ectoderm development | EED | AF080227 | Hs.512812 | chr11q14.2-q22.3 |
| 209571_at | CBF1 interacting corepressor | CIR | U03644 | Hs.89421 | chr2q31.1 |
| 209569_x_at | DNA segment on chromosome 4 (unique) 234 expressed sequence | D4S234E | NM_014392 | Hs.79404 | chr4p16.3 |
| 209568_s_at | ral guanine nucleotide dissociation stimulator-like 1 | RGL1 | AF186779 | Hs.79219 | chr1q25.3 |
| 209567_at | RRS1 ribosome biogenesis regulator homolog (S. cerevisiae) | RRS1 | BC001811 | Hs.71827 | chr8q13.1 |
| 209566_at | insulin induced gene 2 | INSIG2 | AL080184 | Hs.7089 | chr2q14.1 |
| 209565_at | ring finger protein 113A | RNF113A | BC000832 | Hs.458365 | chrxq25-q26 |
| 209560_s_at | delta-like 1 homolog (Drosophila) | DLK1 | U15979 | Hs.169228 | chr14q32 |
| 209556_at | neurochondrin | NCDN | AB011179 | Hs.121870 | chr1p34.3 |
| 209553_at | vacuolar protein sorting 8 homolog (S. cerevisiae) | VPS8 | BC001001 | Hs.361493 | chr3q27.2 |
| 209551_at | Yip1 domain family, member 4 | YIPF4 | BC004875 | Hs.66309 | chr2p22.3 |
| 209550_at | necdin homolog (mouse) | NDN | U35139 | Hs.50130 | chr15q11.2-q12 |
| 209545_s_at | receptor-interacting serine-threonine kinase 2 | RIPK2 | AF064824 | Hs.103755 | chr8q21 |
| 209539_at | Rac/Cdc42 guanine nucleotide exchange factor (GEF) 6 | ARHGEF6 | D25304 | Hs.372800 | chrxq26 |
| 209538_at | zinc finger protein 32 | ZNF32 | U69645 | Hs.78765 | chr10q22-q25 |
| 209537_at | exostoses (multiple)-like 2 | EXTL2 | AF000416 | Hs.357637 | chr1p21 |
| 209536_s_at | EH-domain containing 4 | EHD4 | AF320070 | Hs.55058 | chr15q11.1 |
| 209533_s_at | phospholipase A2-activating protein | PLAA | AF145020 | Hs.27182 | chr9p21 |
| 209531_at | glutathione transferase zeta 1 (maleylacetoacetate isomerase) | GSTZ1 | BC001453 | Hs.26403 | chr14q24.3 |
| 209527_at | exosome component 2 | EXOSC2 | BC000747 | Hs.211973 | chr9q34 |
| 209522_s_at | carnitine acetyltransferase | CRAT | BC000723 | Hs.12068 | chr9q34.1 |
| 209520_s_at | nuclear cap binding protein subunit 1, 80kDa | NCBP1 | BC001450 | Hs.439203 | chr9q34.1 |
| 209518_at | SWI/SNF related, matrix associated, actin dependent regulator of chromatin, subfamily d, member 1 | SMARCD1 | AI869240 | Hs.79335 | chr12q13-q14 |
| 209517_s_at | ash2 (absent, small, or homeotic)-like (Drosophila) | ASH2L | AB020982 | Hs.6856 | chr8p11.2 |
| 209511_at | polymerase (RNA) II (DNA directed) polypeptide F | POLR2F | BC003582 | Hs.46405 | chr22q13.1 |
| 209510_at | ring finger protein 139 | RNF139 | AF064801 | Hs.28285 | chr8q24 |
| 209509_s_at | dolichyl-phosphate (UDP-N-acetylglucosamine) N-acetylglucosaminephosphotransferase 1 (GlcNAc-1-P transferase) | DPAGT1 | BC000325 | Hs.26433 | chr11q23.3 |
| 209507_at | replication protein A3, 14kDa | RPA3 | BC005264 | Hs.1608 | chr7p22 |
| 209506_s_at | nuclear receptor subfamily 2, group F, member 1 | NR2F1 | BC004154 | Hs.361748 | chr5q14 |
| 209503_s_at | proteasome (prosome, macropain) 26S subunit, ATPase, 5 | PSMC5 | AF035309 | Hs.79387 | chr17q23-q25 |
| 209493_at | PDZ domain containing 2 | PDZD2 | AF338650 | Hs.287460 | chr5p13.3 |
| 209492_x_at | ATP synthase, H+ transporting, mitochondrial F0 complex, subunit E | ATP5I | BC003679 | Hs.85539 | chr4p16.3 |
| 209490_s_at | palmitoyl-protein thioesterase 2 | PPT2 | AF020543 | Hs.332138 | chr6p21.3 |
| 209488_s_at | RNA binding protein with multiple splicing | RBPMS | D84109 | Hs.195825 | chr8p12-p11 |
| 209486_at | disrupter of silencing 10 | SAS10 | BC004546 | Hs.322901 | chr4q13.3 |
| 209485_s_at | oxysterol binding protein-like 1A | OSBPL1A | W19983 | Hs.415753 | chr18q11.1 |
| 209482_at | processing of precursor 7, ribonuclease P subunit (S. cerevisiae) | POP7 | BC001430 | Hs.416994 | chr7q22 |
| 209481_at | SNF related kinase | SNRK | AF226044 | Hs.79025 | chr3p22.1 |
| 209479_at | coiled-coil domain containing 28A | CCDC28A | BC000758 | Hs.44468 | chr6q23.1-q24.1 |
| 209477_at | emerin (Emery-Dreifuss muscular dystrophy) | EMD | BC000738 | Hs.2985 | chrxq28 |
| 209472_at | kynurenine aminotransferase III | RP11-82K18.3 | BC000819 | Hs.134460 | chr1p22.2 |
| 209467_s_at | MAP kinase interacting serine/threonine kinase 1 | MKNK1 | BC002755 | Hs.79516 | chr1p33 |
| 209463_s_at | TAF12 RNA polymerase II, TATA box binding protein (TBP)-associated factor, 20kDa | TAF12 | D50544 | Hs.421646 | chr1p35.3 |
| 209461_x_at | WD repeat domain 18 | WDR18 | BC001648 | Hs.325321 | chr19p13.3 |
| 209457_at | dual specificity phosphatase 5 | DUSP5 | U16996 | Hs.2128 | chr10q25 |
| 209455_at | F-box and WD-40 domain protein 11 | FBXW11 | BE963245 | Hs.221281 | chr5q35.1 |
| 209454_s_at | TEA domain family member 3 | TEAD3 | AF142482 | Hs.203846 | chr6p21.2 |
| 209450_at | O-sialoglycoprotein endopeptidase | OSGEP | AB050442 | Hs.413652 | chr14q11.2 |
| 209449_at | LSM2 homolog, U6 small nuclear RNA associated (S. cerevisiae) | LSM2 | AF196468 | Hs.103106 | chr6p21.3 |
| 209443_at | serpin peptidase inhibitor, clade A (alpha-1 antiproteinase, antitrypsin), member 5 | SERPINA5 | J02639 | Hs.76353 | chr14q32.1 |
| 209439_s_at | phosphorylase kinase, alpha 2 (liver) | PHKA2 | D38616 | Hs.54941 | chrxp22.2-p22.1 |
| 209434_s_at | phosphoribosyl pyrophosphate amidotransferase | PPAT | U00238 | Hs.311625 | chr4q12 |
| 209432_s_at | cAMP responsive element binding protein 3 | CREB3 | AF029674 | Hs.287921 | chr9pter-p22.1 |
| 209431_s_at | POZ (BTB) and AT hook containing zinc finger 1 | PATZ1 | AF254083 | Hs.27801 | chr22q12.2 |
| 209430_at | BTAF1 RNA polymerase II, B-TFIID transcription factor-associated, 170kDa (Mot1 homolog, S. cerevisiae) | BTAF1 | AJ001017 | Hs.180930 | chr10q22-q23 |
| 209426_s_at | alpha-methylacyl-CoA racemase | AMACR | AF047020 | Hs.49598 | chr5p13.2-q11.1 |
| 209421_at | mutS homolog 2, colon cancer, nonpolyposis type 1 (E. coli) | MSH2 | U04045 | Hs.440394 | chr2p22-p21 |
| 209418_s_at | THO complex 5 | THOC5 | BC003615 | Hs.75361 | chr22q12.2 |
| 209417_s_at | interferon-induced protein 35 | IFI35 | BC001356 | Hs.50842 | chr17q21 |
| 209413_at | UDP-Gal:betaGlcNAc beta 1,4- galactosyltransferase, polypeptide 2 | B4GALT2 | BC002431 | Hs.442808 | chr1p34-p33 |
| 209410_s_at | growth factor receptor-bound protein 10 | GRB10 | AF000017 | Hs.130119 | chr7p12-p11.2 |
| 209406_at | BCL2-associated athanogene 2 | BAG2 | AF095192 | Hs.55220 | chr6p12.3-p11.2 |
| 209404_s_at | transmembrane emp24 protein transport domain containing 7 | TMED7 | AF151867 | Hs.278391 | chr5q22.3 |
| 209403_at | TBC1 domain family, member 3 /// TBC1 domain family, member 3C /// similar to USP6 N-terminal like /// similar to USP6 N-terminal like /// similar to TBC1 domain family member 3 (Rab GTPase-activating protein PRC17) (Prostate cancer gene 17 protein) (TRE1 | TBC1D3 | AL136860 | Hs.95871 | chr17q12 /// chr17q21.1 |
| 209398_at | histone cluster 1, H1c | HIST1H1C | BC002649 | Hs.7644 | chr6p21.3 |
| 209394_at | acetylserotonin O-methyltransferase-like | ASMTL | BC002508 | Hs.458420 | chrxp22.3; yp11.3 |
| 209391_at | dolichyl-phosphate mannosyltransferase polypeptide 2, regulatory subunit | DPM2 | AF061729 | Hs.108973 | chr9q34.13 |
| 209390_at | tuberous sclerosis 1 | TSC1 | AF013168 | Hs.69429 | chr9q34 |
| 209386_at | transmembrane 4 L six family member 1 | TM4SF1 | AI346835 | Hs.351316 | chr3q21-q25 |
| 209378_s_at | KIAA1128 | KIAA1128 | AI478879 | Hs.81897 | chr10q23.1 |
| 209377_s_at | high mobility group nucleosomal binding domain 3 | HMGN3 | AF274949 | Hs.77558 | chr6q14.1 |
| 209375_at | xeroderma pigmentosum, complementation group C | XPC | D21089 | Hs.320 | chr3p25 |
| 209372_x_at | tubulin, beta 2A /// tubulin, beta 2B | TUBB2A | BF971587 | Hs.512712 | chr6p25 |
| 209367_at | syntaxin binding protein 2 | STXBP2 | AB002559 | Hs.379204 | chr19p13.3-p13.2 |
| 209365_s_at | extracellular matrix protein 1 | ECM1 | U65932 | Hs.81071 | chr1q21 |
| 209364_at | BCL2-antagonist of cell death | BAD | U66879 | Hs.76366 | chr11q13.1 |
| 209356_x_at | EGF-containing fibulin-like extracellular matrix protein 2 | EFEMP2 | AB030655 | Hs.381870 | chr11q13 |
| 209355_s_at | phosphatidic acid phosphatase type 2B | PPAP2B | AB000889 | Hs.432840 | chr1pter-p22.1 |
| 209354_at | tumor necrosis factor receptor superfamily, member 14 (herpesvirus entry mediator) | TNFRSF14 | BC002794 | Hs.279899 | chr1p36.3-p36.2 |
| 209352_s_at | SIN3 homolog B, transcription regulator (yeast) | SIN3B | AB014600 | Hs.13999 | chr19p13.11 |
| 209350_s_at | G protein pathway suppressor 2 | GPS2 | AL157493 | Hs.438219 | chr17p13 |
| 209349_at | RAD50 homolog (S. cerevisiae) | RAD50 | U63139 | Hs.41587 | chr5q31 |
| 209341_s_at | inhibitor of kappa light polypeptide gene enhancer in B-cells, kinase beta | IKBKB | AU153366 | Hs.413513 | chr8p11.2 |
| 209340_at | UDP-N-acteylglucosamine pyrophosphorylase 1 | UAP1 | S73498 | Hs.21293 | chr1q23.3 |
| 209339_at | seven in absentia homolog 2 (Drosophila) /// seven in absentia homolog 2 (Drosophila) | SIAH2 | U76248 | Hs.20191 | chr3q25 |
| 209338_at | transcription factor CP2 | TFCP2 | U03494 | Hs.154970 | chr12q13 |
| 209336_at | PWP2 periodic tryptophan protein homolog (yeast) | PWP2 | U56085 | Hs.449076 | chr21q22.3 |
| 209335_at | decorin | DCN | AI281593 | Hs.156316 | chr12q13.2 |
| 209333_at | unc-51-like kinase 1 (C. elegans) | ULK1 | AB018265 | Hs.47061 | chr12q24.3 |
| 209331_s_at | MYC associated factor X | MAX | AA723514 | Hs.42712 | chr14q23 |
| 209328_x_at | HIG1 domain family, member 2A | HIGD2A | AA243508 | Hs.424552 | chr5q35.2 |
| 209323_at | protein-kinase, interferon-inducible double stranded RNA dependent inhibitor, repressor of (P58 repressor) | PRKRIR | AF081567 | Hs.177574 | chr11q13.5 |
| 209321_s_at | adenylate cyclase 3 | ADCY3 | AF033861 | Hs.188402 | chr2p24-p22 |
| 209314_s_at | HBS1-like (S. cerevisiae) | HBS1L | AK024258 | Hs.221040 | chr6q23-q24 |
| 209313_at | XPA binding protein 1, GTPase | XAB1 | AB044661 | Hs.18259 | chr2p23.3 |
| 209311_at | BCL2-like 2 | BCL2L2 | D87461 | Hs.410026 | chr14q11.2-q12 |
| 209310_s_at | caspase 4, apoptosis-related cysteine peptidase | CASP4 | U25804 | Hs.74122 | chr11q22.2-q22.3 |
| 209308_s_at | BCL2/adenovirus E1B 19kDa interacting protein 2 | BNIP2 | BC002461 | Hs.204539 | chr15q22.2 |
| 209306_s_at | SWAP-70 protein | SWAP70 | AI139569 | Hs.153026 | chr11p15 |
| 209305_s_at | growth arrest and DNA-damage-inducible, beta | GADD45B | AF078077 | Hs.110571 | chr19p13.3 |
| 209303_at | NADH dehydrogenase (ubiquinone) Fe-S protein 4, 18kDa (NADH-coenzyme Q reductase) | NDUFS4 | BC005270 | Hs.10758 | chr5q11.1 |
| 209302_at | polymerase (RNA) II (DNA directed) polypeptide H | POLR2H | U37689 | Hs.432574 | chr3q28 |
| 209301_at | carbonic anhydrase II | CA2 | M36532 | Hs.155097 | chr8q22 |
| 209300_s_at | NECAP endocytosis associated 1 | NECAP1 | BC002888 | Hs.527839 | chr12p13.31 |
| 209298_s_at | intersectin 1 (SH3 domain protein) | ITSN1 | AF114488 | Hs.66392 | chr21q22.1-q22.2 |
| 209295_at | tumor necrosis factor receptor superfamily, member 10b | TNFRSF10B | AF016266 | Hs.521456 | chr8p22-p21 |
| 209287_s_at | CDC42 effector protein (Rho GTPase binding) 3 | CDC42EP3 | AF104857 | Hs.352554 | chr2p21 |
| 209283_at | crystallin, alpha B | CRYAB | AF007162 | Hs.408767 | chr11q22.3-q23.1 |
| 209279_s_at | NAD(P) dependent steroid dehydrogenase-like | NSDHL | BC000245 | Hs.57698 | chrxq28 |
| 209277_at | tissue factor pathway inhibitor 2 | TFPI2 | AL574096 | Hs.438231 | chr7q22 |
| 209276_s_at | glutaredoxin (thioltransferase) | GLRX | AF162769 | Hs.28988 | chr5q14 |
| 209275_s_at | ceroid-lipofuscinosis, neuronal 3, juvenile (Batten, Spielmeyer-Vogt disease) | CLN3 | AF015593 | Hs.446747 | chr16p12.1 |
| 209268_at | vacuolar protein sorting 45 homolog (S. cerevisiae) | VPS45 | AF165513 | Hs.134231 | chr1q21-q22 |
| 209267_s_at | solute carrier family 39 (zinc transporter), member 8 | SLC39A8 | AB040120 | Hs.284205 | chr4q22-q24 |
| 209265_s_at | methyltransferase like 3 | METTL3 | BC001650 | Hs.168799 | chr14q11.1 |
| 209264_s_at | tetraspanin 4 | TSPAN4 | AF054841 | Hs.26518 | chr11p15.5 |
| 209257_s_at | structural maintenance of chromosomes 3 | SMC3 | BF795297 | Hs.24485 | chr10q25 |
| 209254_at | KIAA0265 protein | KIAA0265 | AI808625 | Hs.192966 | chr7q32.2 |
| 209252_at | histidyl-tRNA synthetase-like | HARSL | U18937 | Hs.432560 | chr5q31.3 |
| 209251_x_at | tubulin, alpha 6 | TUBA6 | BC004949 | Hs.406578 | chr12q12-q14 |
| 209249_s_at | growth hormone inducible transmembrane protein | GHITM | AF131820 | Hs.352656 | chr10q23.1 |
| 209239_at | nuclear factor of kappa light polypeptide gene enhancer in B-cells 1 (p105) | NFKB1 | M55643 | Hs.431926 | chr4q24 |
| 209236_at | solute carrier family 23 (nucleobase transporters), member 2 | SLC23A2 | AL389886 | Hs.82042 | chr20p13 |
| 209233_at | EMG1 nucleolar protein homolog (S. cerevisiae) | EMG1 | U72514 | Hs.135643 | chr12p13 |
| 209231_s_at | dynactin 5 (p25) | DCTN5 | AI038068 | Hs.435941 | chr16p12.1 |
| 209230_s_at | nuclear protein 1 | NUPR1 | AF135266 | Hs.418692 | chr16p11.2 |
| 209229_s_at | SAPS domain family, member 1 | SAPS1 | BC002799 | Hs.411875 | chr19q13.42 |
| 209228_x_at | tumor suppressor candidate 3 | TUSC3 | U42349 | Hs.426324 | chr8p22 |
| 209224_s_at | NADH dehydrogenase (ubiquinone) 1 alpha subcomplex, 2, 8kDa | NDUFA2 | BC003674 | Hs.163867 | chr5q31 |
| 209221_s_at | oxysterol binding protein-like 2 | OSBPL2 | AI753638 | Hs.15519 | chr20q13.3 |
| 209219_at | RD RNA binding protein | RDBP | L03411 | Hs.423935 | chr6p21.3 |
| 209216_at | WD repeat domain 45 | WDR45 | BC000464 | Hs.21753 | chrxp11.23 |
| 209215_at | tetracycline transporter-like protein | TETRAN | L11669 | Hs.157145 | chr4p16.3 |
| 209213_at | carbonyl reductase 1 | CBR1 | BC002511 | Hs.88778 | chr21q22.13 |
| 209212_s_at | Kruppel-like factor 5 (intestinal) | KLF5 | AB030824 | Hs.84728 | chr13q22.1 |
| 209209_s_at | pleckstrin homology domain containing, family C (with FERM domain) member 1 | PLEKHC1 | AW469573 | Hs.270411 | chr14q22.1 |
| 209204_at | LIM domain only 4 | LMO4 | AI824831 | Hs.3844 | chr1p22.3 |
| 209203_s_at | bicaudal D homolog 2 (Drosophila) | BICD2 | BC002327 | Hs.436939 | chr9q22.31 |
| 209199_s_at | MADS box transcription enhancer factor 2, polypeptide C (myocyte enhancer factor 2C) | MEF2C | N22468 | Hs.368950 | chr5q14 |
| 209197_at | synaptotagmin XI | SYT11 | AA626780 | Hs.380439 | chr1q21.2 |
| 209196_at | WD repeat domain 46 | WDR46 | BC000388 | Hs.436930 | chr6p21.3 |
| 209194_at | centrin, EF-hand protein, 2 | CETN2 | BC005334 | Hs.82794 | chrxq28 |
| 209193_at | pim-1 oncogene /// pim-1 oncogene | PIM1 | M24779 |  |  |
| 209192_x_at | HIV-1 Tat interacting protein, 60kDa | HTATIP | BC000166 | Hs.397010 | chr11q13 |
| 209186_at | ATPase, Ca++ transporting, cardiac muscle, slow twitch 2 | ATP2A2 | M23114 | Hs.374535 | chr12q23-q24.1 |
| 209182_s_at | chromosome 10 open reading frame 10 | C10orf10 | AI302100 | Hs.93675 | chr10q11.21 |
| 209179_s_at | leukocyte receptor cluster (LRC) member 4 | LENG4 | BC003164 | Hs.78768 | chr19q13.4 |
| 209178_at | DEAH (Asp-Glu-Ala-His) box polypeptide 38 | DHX38 | AF038391 | Hs.127092 | chr16q21-q22.3 |
| 209177_at | chromosome 3 open reading frame 60 | C3orf60 | BC002873 | Hs.31387 | chr3p21.31 |
| 209174_s_at | glutamine-rich 1 | QRICH1 | BC000978 | Hs.297389 | chr3p21.31 |
| 209171_at | inosine triphosphatase (nucleoside triphosphate pyrophosphatase) | ITPA | AF219116 | Hs.6817 | chr20p |
| 209169_at | glycoprotein M6B | GPM6B | N63576 | Hs.5422 | chrxp22.2 |
| 209166_s_at | mannosidase, alpha, class 2B, member 1 | MAN2B1 | U68567 | Hs.99447 | chr19cen-q13.1 |
| 209165_at | apoptosis antagonizing transcription factor | AATF | AF083208 | Hs.311079 | chr17q11.2-q12 |
| 209161_at | PRP4 pre-mRNA processing factor 4 homolog (yeast) | PRPF4 | AI184802 | Hs.374973 | chr9q31-q33 |
| 209158_s_at | pleckstrin homology, Sec7 and coiled-coil domains 2 (cytohesin-2) | PSCD2 | BC004361 | Hs.8517 | chr19q13.3 |
| 209157_at | DnaJ (Hsp40) homolog, subfamily A, member 2 | DNAJA2 | AF011793 | Hs.368078 | chr16q11.1-q11.2 |
| 209155_s_at | 5'-nucleotidase, cytosolic II | NT5C2 | BC001595 | Hs.415988 | chr10q24.33 |
| 209154_at | Tax1 (human T-cell leukemia virus type I) binding protein 3 | TAX1BP3 | AF234997 | Hs.12956 | chr17p13 |
| 209148_at | retinoid X receptor, beta | RXRB | BC001167 | Hs.388034 | chr6p21.3 |
| 209146_at | sterol-C4-methyl oxidase-like | SC4MOL | AV704962 | Hs.393239 | chr4q32-q34 |
| 209139_s_at | protein kinase, interferon-inducible double stranded RNA dependent activator | PRKRA | AF083033 | Hs.144946 | chr2q31.2 |
| 209136_s_at | ubiquitin specific peptidase 10 | USP10 | BG390445 | Hs.78829 | chr16q24.1 |
| 209135_at | aspartate beta-hydroxylase | ASPH | AF289489 | Hs.413557 | chr8q12.1 |
| 209129_at | thyroid hormone receptor interactor 6 | TRIP6 | AF000974 | Hs.380230 | chr7q22 |
| 209124_at | myeloid differentiation primary response gene (88) | MYD88 | U70451 | Hs.82116 | chr3p22 |
| 209123_at | quinoid dihydropteridine reductase | QDPR | BC000576 | Hs.75438 | chr4p15.31 |
| 209122_at | adipose differentiation-related protein | ADFP | BC005127 | Hs.3416 | chr9p22.1 |
| 209118_s_at | tubulin, alpha 3 | TUBA3 | AF141347 | Hs.433394 | chr12q12-12q14.3 |
| 209117_at | WW domain binding protein 2 | WBP2 | U79458 | Hs.231840 | chr17q25 |
| 209115_at | ubiquitin-activating enzyme E1C (UBA3 homolog, yeast) | UBE1C | AL117566 | Hs.154320 | chr3p24.3-p13 |
| 209113_s_at | high-mobility group 20B | HMG20B | AF288679 | Hs.406534 | chr19p13.3 |
| 209112_at | cyclin-dependent kinase inhibitor 1B (p27, Kip1) | CDKN1B | BC001971 | Hs.238990 | chr12p13.1-p12 |
| 209111_at | ring finger protein 5 | RNF5 | BC004155 | Hs.216354 | chr6p21.3 |
| 209110_s_at | ral guanine nucleotide dissociation stimulator-like 2 | RGL2 | AL050259 | Hs.170160 | chr6p21.3 |
| 209109_s_at | tetraspanin 6 /// zinc finger protein 444 | TSPAN6 | U84895 | Hs.43233 | chrxq22 |
| 209105_at | nuclear receptor coactivator 1 | NCOA1 | AI672428 | Hs.386092 | chr2p23 |
| 209104_s_at | nucleolar protein family A, member 2 (H/ACA small nucleolar RNPs) | NOLA2 | BC000009 | Hs.386392 | chr5q35.3 |
| 209103_s_at | ubiquitin fusion degradation 1 like (yeast) | UFD1L | BC001049 | Hs.404525 | chr22q11.21 |
| 209102_s_at | HMG-box transcription factor 1 | HBP1 | AF019214 | Hs.437606 | chr7q22-q31 |
| 209101_at | connective tissue growth factor | CTGF | M92934 | Hs.410037 | chr6q23.1 |
| 209100_at | interferon-related developmental regulator 2 | IFRD2 | BC001327 | Hs.315177 | chr3p21.3 |
| 209099_x_at | jagged 1 (Alagille syndrome) | JAG1 | U73936 | Hs.409202 | chr20p12.1-p11.23 |
| 209096_at | ubiquitin-conjugating enzyme E2 variant 2 | UBE2V2 | U62136 | Hs.371278 | chr8q11.21 |
| 209094_at | dimethylarginine dimethylaminohydrolase 1 | DDAH1 | AL078459 | Hs.380870 | chr1p22 |
| 209092_s_at | chromosome 17 open reading frame 25 | C17orf25 | AF061730 | Hs.279061 | chr17p13.3 |
| 209090_s_at | SH3-domain GRB2-like endophilin B1 | SH3GLB1 | AL049597 | Hs.136309 | chr1p22 |
| 209085_x_at | replication factor C (activator 1) 1, 145kDa | RFC1 | L14922 | Hs.166563 | chr4p14-p13 |
| 209084_s_at | RAB28, member RAS oncogene family | RAB28 | BE504689 | Hs.306899 | chr4p15.33 |
| 209081_s_at | collagen, type XVIII, alpha 1 | COL18A1 | NM_030582 | Hs.413175 | chr21q22.3 |
| 209080_x_at | thioredoxin-like 2 | TXNL2 | AF118652 | Hs.42644 | chr6p25.3 |
| 209077_at | thioredoxin 2 | TXN2 | AL022313 | Hs.211929 | chr22q13.1 |
| 209076_s_at | WDR45-like | WDR45L | BC000974 | Hs.201390 | chr17q25.3 |
| 209075_s_at | IscU iron-sulfur cluster scaffold homolog (E. coli) | ISCU | AY009128 | Hs.350702 | chr12q24.1 |
| 209070_s_at | regulator of G-protein signalling 5 | RGS5 | AI183997 | Hs.24950 | chr1q23.1 |
| 209068_at | heterogeneous nuclear ribonucleoprotein D-like | HNRPDL | D89678 | Hs.372673 | chr4q13-q21 |
| 209065_at | ubiquinol-cytochrome c reductase binding protein | UQCRB | BC005230 | Hs.131255 | chr8q22 |
| 209058_at | endothelial differentiation-related factor 1 | EDF1 | AB002282 | Hs.174050 | chr9q34.3 |
| 209055_s_at | CDC5 cell division cycle 5-like (S. pombe) | CDC5L | AW268817 | Hs.443753 | chr6p21 |
| 209051_s_at | ral guanine nucleotide dissociation stimulator | RALGDS | AF295773 | Hs.106185 | chr9q34.3 |
| 209046_s_at | GABA(A) receptor-associated protein-like 2 | GABARAPL2 | AB030710 | Hs.6518 | chr16q22.3-q24.1 |
| 209044_x_at | splicing factor 3b, subunit 4, 49kDa | SF3B4 | BC004273 | Hs.406186 | chr1q12-q21 |
| 209043_at | 3'-phosphoadenosine 5'-phosphosulfate synthase 1 | PAPSS1 | AF033026 | Hs.3833 | chr4q24 |
| 209041_s_at | ubiquitin-conjugating enzyme E2G 2 (UBC7 homolog, yeast) | UBE2G2 | BG395660 | Hs.250387 | chr21q22.3 |
| 209040_s_at | proteasome (prosome, macropain) subunit, beta type, 8 (large multifunctional peptidase 7) | PSMB8 | U17496 | Hs.180062 | chr6p21.3 |
| 209035_at | midkine (neurite growth-promoting factor 2) | MDK | M69148 | Hs.82045 | chr11p11.2 |
| 209034_at | proline-rich nuclear receptor coactivator 1 | PNRC1 | AF279899 | Hs.75969 | chr6q15 |
| 209033_s_at | dual-specificity tyrosine-(Y)-phosphorylation regulated kinase 1A | DYRK1A | D86550 | Hs.75842 | chr21q22.13 |
| 209032_s_at | immunoglobulin superfamily, member 4 | IGSF4 | AF132811 | Hs.156682 | chr11q23.2 |
| 209029_at | COP9 constitutive photomorphogenic homolog subunit 7A (Arabidopsis) | COPS7A | AF193844 | Hs.125309 | chr12p13.31 |
| 209027_s_at | abl-interactor 1 | ABI1 | BF673013 | Hs.42710 | chr10p11.2 |
| 209026_x_at | tubulin, beta | TUBB | AF141349 | Hs.356729 | chr6p21.33 |
| 209021_x_at | KIAA0652 | KIAA0652 | BC001331 | Hs.410092 | chr11p11.2 |
| 209018_s_at | PTEN induced putative kinase 1 | PINK1 | BF432478 | Hs.389171 | chr1p36 |
| 209017_s_at | lon peptidase 1, mitochondrial | LONP1 | U02389 | Hs.350265 | chr19p13.2 |
| 209015_s_at | DnaJ (Hsp40) homolog, subfamily B, member 6 | DNAJB6 | BC002446 | Hs.181195 | chr7q36.3 |
| 209014_at | melanoma antigen family D, 1 | MAGED1 | AF217963 | Hs.5258 | chrxp11.23 |
| 209013_x_at | triple functional domain (PTPRF interacting) | TRIO | AF091395 | Hs.367689 | chr5p15.1-p14 |
| 209008_x_at | keratin 8 /// keratin 8 | KRT8 | U76549 | Hs.356123 | chr12q13 |
| 209006_s_at | chromosome 1 open reading frame 63 | C1orf63 | AF247168 | Hs.259412 | chr1p36.13-p35.1 |
| 209005_at | F-box and leucine-rich repeat protein 5 | FBXL5 | AF157323 | Hs.5548 | chr4p15.33 |
| 209002_s_at | calcium binding and coiled-coil domain 1 | CALCOCO1 | BC003177 | Hs.156667 | chr12q13.13 |
| 209001_s_at | anaphase promoting complex subunit 13 | ANAPC13 | BC005398 | Hs.106909 | chr3q22.1 |
| 208999_at | septin 8 | septin 8 | D86957 | Hs.307944 | chr5q31 |
| 208998_at | uncoupling protein 2 (mitochondrial, proton carrier) | UCP2 | U94592 | Hs.80658 | chr11q13 |
| 208995_s_at | peptidylprolyl isomerase G (cyclophilin G) | PPIG | U40763 | Hs.77965 | chr2q31.1 |
| 208989_s_at | F-box and leucine-rich repeat protein 11 | FBXL11 | AF179221 | Hs.219614 | chr11q13.2 |
| 208980_s_at | ubiquitin C | UBC | M26880 | Hs.183704 | chr12q24.3 |
| 208979_at | nuclear receptor coactivator 6 | NCOA6 | AF128458 | Hs.435788 | chr20q11 |
| 208977_x_at | tubulin, beta 2C | TUBB2C | BC004188 | Hs.433615 |  |
| 208973_at | prion protein interacting protein | PRNPIP | BC001072 | Hs.368557 | chr1p32 |
| 208972_s_at | ATP synthase, H+ transporting, mitochondrial F0 complex, subunit C1 (subunit 9) | ATP5G1 | AL080089 | Hs.80986 | chr17q21.32 |
| 208969_at | NADH dehydrogenase (ubiquinone) 1 alpha subcomplex, 9, 39kDa | NDUFA9 | AF050641 | Hs.75227 | chr12p13.3 |
| 208962_s_at | fatty acid desaturase 1 | FADS1 | BE540552 | Hs.503546 | chr11q12.2-q13.1 |
| 208957_at | thioredoxin domain containing 4 (endoplasmic reticulum) | TXNDC4 | BF439241 | Hs.154023 | chr9q31.1 |
| 208955_at | dUTP pyrophosphatase | DUT | AB049113 | Hs.367676 | chr15q15-q21.1 |
| 208951_at | aldehyde dehydrogenase 7 family, member A1 | ALDH7A1 | BC002515 | Hs.312102 | chr5q31 |
| 208949_s_at | lectin, galactoside-binding, soluble, 3 (galectin 3) | LGALS3 | BC001120 | Hs.411701 | chr14q21-q22 |
| 208945_s_at | beclin 1 (coiled-coil, myosin-like BCL2 interacting protein) | BECN1 | NM_003766 | Hs.12272 | chr17q21 |
| 208944_at | transforming growth factor, beta receptor II (70/80kDa) | TGFBR2 | D50683 |  |  |
| 208940_at | Selenophosphate synthetase 1 | SEPHS1 | AI885670 | Hs.124027 | chr10p14 |
| 208938_at | papillary renal cell carcinoma (translocation-associated) | PRCC | BC004913 | Hs.9629 | chr1q21.1 |
| 208932_at | protein phosphatase 4 (formerly X), catalytic subunit | PPP4C | BC001416 | Hs.534358 | chr16p12-16p11 |
| 208930_s_at | interleukin enhancer binding factor 3, 90kDa | ILF3 | BG032366 | Hs.256583 | chr19p13.2 |
| 208928_at | P450 (cytochrome) oxidoreductase | POR | AF258341 | Hs.354056 | chr7q11.2 |
| 208926_at | sialidase 1 (lysosomal sialidase) | NEU1 | U84246 | Hs.118721 | chr6p21.3 |
| 208924_at | ring finger protein 11 | RNF11 | AB024703 | Hs.96334 | chr1pter-p22.1 |
| 208923_at | cytoplasmic FMR1 interacting protein 1 | CYFIP1 | BC005097 | Hs.26704 | chr15q11 |
| 208922_s_at | nuclear RNA export factor 1 | NXF1 | BC004904 | Hs.323502 | chr11q12-q13 |
| 208920_at | sorcin | SRI | AV752215 | Hs.422340 | chr7q21.1 |
| 208916_at | solute carrier family 1 (neutral amino acid transporter), member 5 | SLC1A5 | AF105230 | Hs.183556 | chr19q13.3 |
| 208914_at | golgi associated, gamma adaptin ear containing, ARF binding protein 2 | GGA2 | BE646414 | Hs.133340 | chr16p12 |
| 208912_s_at | 2',3'-cyclic nucleotide 3' phosphodiesterase | CNP | BC001362 | Hs.443836 | chr17q21 |
| 208911_s_at | pyruvate dehydrogenase (lipoamide) beta | PDHB | M34055 | Hs.161357 | chr3p21.1-p14.2 |
| 208910_s_at | complement component 1, q subcomponent binding protein | C1QBP | L04636 | Hs.413852 | chr17p13.3 |
| 208909_at | ubiquinol-cytochrome c reductase, Rieske iron-sulfur polypeptide 1 | UQCRFS1 | BC000649 | Hs.355735 | chr19q12-q13.1 |
| 208907_s_at | mitochondrial ribosomal protein S18B | MRPS18B | BC005373 | Hs.274417 | chr6p21.3 |
| 208904_s_at | ribosomal protein S28 /// similar to 40S ribosomal protein S28 /// similar to 40S ribosomal protein S28 | RPS28 | BC000354 | Hs.153177 | chr19p13.2 |
| 208900_s_at | topoisomerase (DNA) I | TOP1 | AW025108 | Hs.253536 | chr20q12-q13.1 |
| 208896_at | DEAD (Asp-Glu-Ala-Asp) box polypeptide 18 | DDX18 | X98743 | Hs.363492 | chr2q14.1 |
| 208895_s_at | wingless-type MMTV integration site family, member 8B /// DEAD (Asp-Glu-Ala-Asp) box polypeptide 18 | WNT8B | BG530850 | Hs.363492 | chr2q14.1 |
| 208894_at | major histocompatibility complex, class II, DR alpha /// major histocompatibility complex, class II, DR alpha | HLA-DRA | M60334 | Hs.409805 | chr6p21.3 |
| 208891_at | dual specificity phosphatase 6 | DUSP6 | BC003143 | Hs.298654 | chr12q22-q23 |
| 208890_s_at | plexin B2 | PLXNB2 | BC004542 | Hs.3989 | chr22q13.33 |
| 208887_at | eukaryotic translation initiation factor 3, subunit 4 delta, 44kDa | EIF3S4 | BC000733 | Hs.28081 | chr19p13.2 |
| 208886_at | H1 histone family, member 0 | H1F0 | BC000145 | Hs.226117 | chr22q13.1 |
| 208880_s_at | PRP6 pre-mRNA processing factor 6 homolog (S. cerevisiae) | PRPF6 | AB019219 | Hs.31334 | chr20q13.33 |
| 208874_x_at | protein phosphatase 2A, regulatory subunit B' (PR 53) | PPP2R4 | BC002545 | Hs.400740 | chr9q34 |
| 208872_s_at | receptor accessory protein 5 | REEP5 | AA814140 | Hs.173119 | chr5q22-q23 |
| 208868_s_at | GABA(A) receptor-associated protein like 1 | GABARAPL1 | BF125756 | Hs.336429 | chr12p13.2 |
| 208858_s_at | family with sequence similarity 62 (C2 domain containing), member A | FAM62A | BC004998 | Hs.8309 | chr12q13.2 |
| 208854_s_at | serine/threonine kinase 24 (STE20 homolog, yeast) | STK24 | AA586774 | Hs.168913 | chr13q31.2-q32.3 |
| 208847_s_at | alcohol dehydrogenase 5 (class III), chi polypeptide | ADH5 | M29872 | Hs.78989 | chr4q21-q25 |
| 208845_at | voltage-dependent anion channel 3 | VDAC3 | BC002456 | Hs.439253 | chr8p11.2 |
| 208843_s_at | golgi reassembly stacking protein 2, 55kDa | GORASP2 | BC001408 | Hs.6880 | chr2q31.1-q31.2 |
| 208839_s_at | cullin-associated and neddylation-dissociated 1 | CAND1 | AL136810 | Hs.512638 | chr12q14 |
| 208837_at | transmembrane emp24 protein transport domain containing 3 | TMED3 | BC000027 | Hs.424551 | chr15q24-q25 |
| 208836_at | ATPase, Na+/K+ transporting, beta 3 polypeptide | ATP1B3 | U51478 | Hs.76941 | chr3q23 |
| 208832_at | ataxin 10 | ATXN10 | AW241832 | Hs.191294 | chr22q13.31 |
| 208831_x_at | suppressor of Ty 6 homolog (S. cerevisiae) | SUPT6H | D79984 | Hs.250429 | chr17q11.2 |
| 208829_at | TAP binding protein (tapasin) | TAPBP | AF029750 | Hs.370937 | chr6p21.3 |
| 208828_at | polymerase (DNA directed), epsilon 3 (p17 subunit) | POLE3 | BC004170 | Hs.108112 | chr9q33 |
| 208827_at | proteasome (prosome, macropain) subunit, beta type, 6 | PSMB6 | BC000835 | Hs.77060 | chr17p13 |
| 208823_s_at | PCTAIRE protein kinase 1 | PCTK1 | BE787860 | Hs.171834 | chrxp11.3-p11.23 |
| 208822_s_at | death associated protein 3 | DAP3 | U18321 | Hs.270920 | chr1q21-q22 |
| 208821_at | small nuclear ribonucleoprotein polypeptides B and B1 | SNRPB | J04564 | Hs.83753 | chr20p13 |
| 208819_at | RAB8A, member RAS oncogene family | RAB8A | BC002977 | Hs.5947 | chr19p13.1 |
| 208816_x_at | annexin A2 pseudogene 2 | ANXA2P2 | M62898 | Hs.534303 | chr9p13 |
| 208813_at | glutamic-oxaloacetic transaminase 1, soluble (aspartate aminotransferase 1) | GOT1 | BC000498 | Hs.597 | chr10q24.1-q25.1 |
| 208808_s_at | high-mobility group box 2 | HMGB2 | BC000903 | Hs.434953 | chr4q31 |
| 208806_at | chromodomain helicase DNA binding protein 3 | CHD3 | BE379542 | Hs.25601 | chr17p13.1 |
| 208805_at | proteasome (prosome, macropain) subunit, alpha type, 6 | PSMA6 | BC002979 | Hs.370121 | chr14q13 |
| 208799_at | proteasome (prosome, macropain) subunit, beta type, 5 | PSMB5 | BC004146 | Hs.422990 | chr14q11.2 |
| 208798_x_at | golgi autoantigen, golgin subfamily a, 8A | GOLGA8A | AF204231 | Hs.182982 | chr15q11.2 |
| 208796_s_at | cyclin G1 | CCNG1 | BC000196 | Hs.79101 | chr5q32-q34 |
| 208789_at | polymerase I and transcript release factor | PTRF | BC004295 | Hs.437191 | chr17q21.2 |
| 208787_at | mitochondrial ribosomal protein L3 | MRPL3 | BC003375 | Hs.320961 | chr3q21-q23 |
| 208785_s_at | microtubule-associated protein 1 light chain 3 beta | MAP1LC3B | BE893893 | Hs.356061 | chr16q24.2 |
| 208784_s_at | kelch domain containing 3 | KLHDC3 | BC001793 | Hs.412468 | chr6p21.1 |
| 208782_at | follistatin-like 1 | FSTL1 | BC000055 | Hs.433622 | chr3q13.33 |
| 208777_s_at | proteasome (prosome, macropain) 26S subunit, non-ATPase, 11 | PSMD11 | AF001212 | Hs.443379 | chr17q11.2 |
| 208775_at | exportin 1 (CRM1 homolog, yeast) | XPO1 | D89729 | Hs.157367 | chr2p16 |
| 208771_s_at | leukotriene A4 hydrolase | LTA4H | J02959 | Hs.81118 | chr12q22 |
| 208768_x_at | ribosomal protein L22 | RPL22 | D17652 | Hs.326249 | chr1p36.3-p36.2 |
| 208764_s_at | ATP synthase, H+ transporting, mitochondrial F0 complex, subunit C2 (subunit 9) | ATP5G2 | D13119 | Hs.89399 | chr12q13.13 |
| 208762_at | SMT3 suppressor of mif two 3 homolog 1 (S. cerevisiae) | SUMO1 | U83117 | Hs.81424 | chr2q33 |
| 208760_at | Ubiquitin-conjugating enzyme E2I (UBC9 homolog, yeast) | UBE2I | AL031714 | Hs.302903 | chr16p13.3 |
| 208758_at | 5-aminoimidazole-4-carboxamide ribonucleotide formyltransferase/IMP cyclohydrolase | ATIC | D89976 | Hs.90280 | chr2q35 |
| 208756_at | eukaryotic translation initiation factor 3, subunit 2 beta, 36kDa | EIF3S2 | U36764 | Hs.192023 | chr1p34.1 |
| 208755_x_at | H3 histone, family 3A /// H3 histone, family 3A pseudogene | H3F3A | BF312331 | Hs.181307 | chr1q41 |
| 208749_x_at | flotillin 1 | FLOT1 | AF085357 | Hs.179986 | chr6p21.3 |
| 208743_s_at | tyrosine 3-monooxygenase/tryptophan 5-monooxygenase activation protein, beta polypeptide | YWHAB | BC001359 | Hs.279920 | chr20q13.1 |
| 208741_at | Sin3A-associated protein, 18kDa | SAP18 | AW274856 | Hs.23964 | chr13q12.11 |
| 208739_x_at | SMT3 suppressor of mif two 3 homolog 2 (S. cerevisiae) | SUMO2 | L76416 | Hs.380973 | chr17q25.1 |
| 208736_at | actin related protein 2/3 complex, subunit 3, 21kDa | ARPC3 | AF004561 | Hs.439511 | chr12q24.11 |
| 208735_s_at | CTD (carboxy-terminal domain, RNA polymerase II, polypeptide A) small phosphatase 2 | CTDSP2 | AF022231 | Hs.355816 | chr12q13-q15 |
| 208730_x_at | RAB2, member RAS oncogene family | RAB2 | AA535244 | Hs.78305 | chr8q12.1 |
| 208729_x_at | major histocompatibility complex, class I, B | HLA-B | D83043 | Hs.77961 | chr6p21.3 |
| 208726_s_at | eukaryotic translation initiation factor 2, subunit 2 beta, 38kDa | EIF2S2 | BC000461 | Hs.429180 | chr20pter-q12 |
| 208723_at | ubiquitin specific peptidase 11 | USP11 | BC000350 | Hs.171501 | chrxp11.23 |
| 208721_s_at | anaphase promoting complex subunit 5 | ANAPC5 | BF967271 | Hs.7101 | chr12q24.31 |
| 208717_at | oxidase (cytochrome c) assembly 1-like | OXA1L | BC001669 | Hs.151134 | chr14q11.2 |
| 208715_at | transmembrane and coiled-coil domains 1 | TMCO1 | BF002031 | Hs.93832 | chr1q22-q25 |
| 208714_at | NADH dehydrogenase (ubiquinone) flavoprotein 1, 51kDa | NDUFV1 | AF092131 | Hs.7744 | chr11q13 |
| 208699_x_at | transketolase (Wernicke-Korsakoff syndrome) | TKT | BF696840 | Hs.89643 | chr3p14.3 |
| 208695_s_at | ribosomal protein L39 | RPL39 | BC001019 | Hs.300141 | chrxq22-q24 |
| 208693_s_at | glycyl-tRNA synthetase | GARS | D30658 | Hs.293885 | chr7p15 |
| 208692_at | ribosomal protein S3 /// structural maintenance of chromosomes 1B | RPS3 | U14990 | Hs.387576 | chr11q13.3-q13.5 |
| 208690_s_at | PDZ and LIM domain 1 (elfin) | PDLIM1 | BC000915 | Hs.75807 | chr10q22-q26.3 |
| 208689_s_at | ribophorin II | RPN2 | BC003560 | Hs.406532 | chr20q12-q13.1 |
| 208687_x_at | heat shock 70kDa protein 8 | HSPA8 | AF352832 | Hs.180414 | chr11q24.1 |
| 208686_s_at | bromodomain containing 2 | BRD2 | D42040 | Hs.75243 | chr6p21.3 |
| 208683_at | calpain 2, (m/II) large subunit | CAPN2 | M23254 | Hs.350899 | chr1q41-q42 |
| 208682_s_at | melanoma antigen family D, 2 | MAGED2 | AF126181 | Hs.376719 | chrxp11.2 |
| 208680_at | peroxiredoxin 1 | PRDX1 | L19184 | Hs.180909 | chr1p34.1 |
| 208678_at | ATPase, H+ transporting, lysosomal 31kDa, V1 subunit E1 | ATP6V1E1 | BC004443 | Hs.77805 | chr22q11.1 |
| 208677_s_at | basigin (Ok blood group) | BSG | AL550657 | Hs.371654 | chr19p13.3 |
| 208674_x_at | dolichyl-diphosphooligosaccharide-protein glycosyltransferase | DDOST | BC002594 | Hs.301882 | chr1p36.1 |
| 208671_at | serine incorporator 1 | SERINC1 | AF164794 | Hs.146668 | chr6q22.31 |
| 208669_s_at | EP300 interacting inhibitor of differentiation 1 | EID1 | AF109873 | Hs.381137 | chr15q21.1-q21.2 |
| 208668_x_at | high-mobility group nucleosomal binding domain 2 | HMGN2 | BC003689 | Hs.181163 | chr1p36.1 |
| 208664_s_at | tetratricopeptide repeat domain 3 | TTC3 | AU131711 | Hs.132605 | chr21q22.2 |
| 208660_at | citrate synthase | CS | BC000105 | Hs.430606 | chr12q13.2-q13.3 |
| 208659_at | chloride intracellular channel 1 | CLIC1 | AF034607 | Hs.414565 | chr6p22.1-p21.2 |
| 208658_at | protein disulfide isomerase family A, member 4 | PDIA4 | BC000425 | Hs.93659 | chr7q35 |
| 208653_s_at | CD164 molecule, sialomucin | CD164 | AF263279 | Hs.43910 | chr6q21 |
| 208652_at | protein phosphatase 2 (formerly 2A), catalytic subunit, alpha isoform | PPP2CA | BC000400 | Hs.91773 | chr5q23-q31 |
| 208648_at | valosin-containing protein | VCP | W60953 | Hs.408723 | chr9p13-p12 |
| 208647_at | farnesyl-diphosphate farnesyltransferase 1 | FDFT1 | AA872727 | Hs.191435 | chr8p23.1-p22 |
| 208645_s_at | ribosomal protein S14 | RPS14 | AF116710 | Hs.381126 | chr5q31-q33 |
| 208644_at | poly (ADP-ribose) polymerase family, member 1 | PARP1 | M32721 | Hs.177766 | chr1q41-q42 |
| 208642_s_at | X-ray repair complementing defective repair in Chinese hamster cells 5 (double-strand-break rejoining; Ku autoantigen, 80kDa) | XRCC5 | AA205834 | Hs.257082 | chr2q35 |
| 208632_at | ring finger protein 10 /// ring finger protein 10 | RNF10 | AL578551 | Hs.528722 | chr12q24.31 |
| 208630_at | hydroxyacyl-Coenzyme A dehydrogenase/3-ketoacyl-Coenzyme A thiolase/enoyl-Coenzyme A hydratase (trifunctional protein), alpha subunit | HADHA | AI972144 | Hs.75860 | chr2p23 |
| 208627_s_at | Y box binding protein 1 | YBX1 | BE966374 | Hs.74497 | chr1p34 |
| 208626_s_at | vesicle amine transport protein 1 homolog (T. californica) | VAT1 | BC001913 | Hs.157236 | chr17q21 |
| 208624_s_at | eukaryotic translation initiation factor 4 gamma, 1 | EIF4G1 | BE966878 | Hs.433750 | chr3q27-qter |
| 208620_at | poly(rC) binding protein 1 | PCBP1 | U24223 | Hs.2853 | chr2p13-p12 |
| 208619_at | damage-specific DNA binding protein 1, 127kDa | DDB1 | L40326 | Hs.290758 | chr11q12-q13 |
| 208615_s_at | protein tyrosine phosphatase type IVA, member 2 | PTP4A2 | BF795101 | Hs.82911 | chr1p35 |
| 208613_s_at | filamin B, beta (actin binding protein 278) | FLNB | AV712733 | Hs.81008 | chr3p14.3 |
| 208611_s_at | spectrin, alpha, non-erythrocytic 1 (alpha-fodrin) | SPTAN1 | U83867 | Hs.387905 | chr9q33-q34 |
| 208598_s_at | HECT, UBA and WWE domain containing 1 | HUWE1 | NM_005703 | Hs.407756 | chrxp11.22 |
| 208588_at | apoptosis inhibitor | FKSG2 | NM_021631 | Hs.302015 | chr8p11.2 |
| 208579_x_at | histone cluster 1, H2bk | HIST1H2BK | NM_017445 | Hs.473961 | chr21q22.3 |
| 208555_x_at | cystatin SA | CST2 | NM_001322 | Hs.247955 | chr20p11.21 |
| 208549_x_at | prothymosin, alpha (gene sequence 28) /// hypothetical gene supported by BC013859 /// similar to prothymosin, alpha (gene sequence 28) /// similar to prothymosin, alpha (gene sequence 28) /// similar to prothymosin, alpha (gene sequence 28) /// hypothetic | PTMA | NM_016171 | Hs.264317 | chr2q35-q36 |
| 208546_x_at | histone cluster 1, H2bh | HIST1H2BH | NM_003524 | Hs.247815 | chr6p21.3 |
| 208540_x_at | similar to Putative S100 calcium-binding protein A11 pseudogene /// similar to Putative S100 calcium-binding protein A11 pseudogene /// similar to Putative S100 calcium-binding protein A11 pseudogene | LOC729659 | NM_021039 |  |  |
| 208527_x_at | histone cluster 1, H2be | HIST1H2BE | NM_003523 |  | chr6p21.3 |
| 208523_x_at | histone cluster 1, H2bi | HIST1H2BI | NM_003525 |  | chr6p21.3 |
| 208517_x_at | basic transcription factor 3 | BTF3 | NM_001207 | Hs.446567 | chr5q13.2 |
| 208503_s_at | GATA zinc finger domain containing 1 | GATAD1 | NM_021167 | Hs.21145 | chr7q21-q22 |
| 208499_s_at | DnaJ (Hsp40) homolog, subfamily C, member 3 | DNAJC3 | NM_006260 | Hs.6019 | chr13q32 |
| 208490_x_at | histone cluster 1, H2bf | HIST1H2BF | NM_003522 |  | chr6p21.3 |
| 208478_s_at | BCL2-associated X protein | BAX | NM_004324 | Hs.159428 | chr19q13.3-q13.4 |
| 208459_s_at | exportin 7 | XPO7 | NM_015024 | Hs.172685 | chr8p21 |
| 208456_s_at | related RAS viral (r-ras) oncogene homolog 2 | RRAS2 | NM_012250 | Hs.206097 | chr11p15.2 |
| 208454_s_at | plasma glutamate carboxypeptidase | PGCP | NM_016134 | Hs.197335 | chr8q22.2 |
| 208453_s_at | X-prolyl aminopeptidase (aminopeptidase P) 1, soluble | XPNPEP1 | NM_006523 | Hs.390623 | chr10q25.3 |
| 208447_s_at | phosphoribosyl pyrophosphate synthetase 1 | PRPS1 | NM_002764 | Hs.56 | chrxq21-q27 |
| 208445_s_at | bromodomain adjacent to zinc finger domain, 1B | BAZ1B | NM_023005 | Hs.194688 | chr7q11.23 |
| 208436_s_at | interferon regulatory factor 7 | IRF7 | NM_004030 | Hs.166120 | chr11p15.5 |
| 208433_s_at | low density lipoprotein receptor-related protein 8, apolipoprotein e receptor | LRP8 | NM_017522 | Hs.410784 | chr1p34 |
| 208425_s_at | tetratricopeptide repeat, ankyrin repeat and coiled-coil containing 2 | TANC2 | NM_015623 | Hs.4996 | chr17q23.3 |
| 208424_s_at | cytokine induced apoptosis inhibitor 1 | CIAPIN1 | NM_020313 | Hs.4900 | chr16q13-q21 |
| 208398_s_at | TBP-like 1 | TBPL1 | NM_004865 | Hs.13993 | chr6q22.1-q22.3 |
| 208374_s_at | capping protein (actin filament) muscle Z-line, alpha 1 | CAPZA1 | NM_006135 | Hs.309415 | chr1p13.2 |
| 208371_s_at | ring finger protein 1 | RING1 | NM_002931 | Hs.202430 | chr6p21.3 |
| 208351_s_at | mitogen-activated protein kinase 1 | MAPK1 | NM_002745 | Hs.324473 | chr22q11.21 |
| 208336_s_at | glycoprotein, synaptic 2 | GPSN2 | NM_004868 | Hs.306122 | chr19p13.12 |
| 208328_s_at | MADS box transcription enhancer factor 2, polypeptide A (myocyte enhancer factor 2A) | MEF2A | NM_005587 | Hs.268675 | chr15q26 |
| 208322_s_at | ST3 beta-galactoside alpha-2,3-sialyltransferase 1 | ST3GAL1 | NM_003033 | Hs.356036 | chr8q24.22 |
| 208316_s_at | oculocerebrorenal syndrome of Lowe | OCRL | M88162 | Hs.126357 | chrxq25-q26.1 |
| 208309_s_at | mucosa associated lymphoid tissue lymphoma translocation gene 1 | MALT1 | NM_006785 | Hs.180566 | chr18q21 |
| 208308_s_at | glucose phosphate isomerase | GPI | NM_000175 | Hs.406458 | chr19q13.1 |
| 208300_at | protein tyrosine phosphatase, receptor type, H | PTPRH | NM_002842 | Hs.179770 | chr19q13.4 |
| 208297_s_at | ecotropic viral integration site 5 | EVI5 | NM_005665 | Hs.387251 | chr1p22 |
| 208290_s_at | eukaryotic translation initiation factor 5 | EIF5 | NM_001969 | Hs.433702 | chr14q32.32 |
| 208289_s_at | etoposide induced 2.4 mRNA | EI24 | NM_004879 | Hs.343911 | chr11q24 |
| 208249_s_at | TDP-glucose 4,6-dehydratase | TGDS | NM_014305 | Hs.12393 | chr13q32.1 |
| 208199_s_at | zinc finger protein 161 homolog (mouse) | ZFP161 | NM_003409 | Hs.156000 | chr18pter-p11.2 |
| 208190_s_at | lipolysis stimulated lipoprotein receptor | LSR | NM_015925 | Hs.312129 | chr19q13.12 |
| 208186_s_at | lipase, hormone-sensitive | LIPE | NM_005357 | Hs.95351 | chr19q13.2 |
| 208161_s_at | ATP-binding cassette, sub-family C (CFTR/MRP), member 3 | ABCC3 | NM_020037 | Hs.90786 | chr17q22 |
| 208156_x_at | epiplakin 1 /// epiplakin 1 | EPPK1 | NM_031308 | Hs.200412 | chr8q24.3 |
| 208152_s_at | DEAD (Asp-Glu-Ala-Asp) box polypeptide 21 /// DEAD (Asp-Glu-Ala-Asp) box polypeptide 21 | DDX21 | NM_004728 | Hs.223141 | chr10q21 |
| 208137_x_at | zinc finger protein 611 /// zinc finger protein 611 /// similar to zinc finger protein 160 /// similar to zinc finger protein 160 | ZNF611 | NM_030972 | Hs.446500 | chr19q13.41 |
| 208131_s_at | prostaglandin I2 (prostacyclin) synthase /// prostaglandin I2 (prostacyclin) synthase | PTGIS | NM_000961 | Hs.302085 | chr20q13.11-q13.13 |
| 208127_s_at | suppressor of cytokine signaling 5 | SOCS5 | NM_014011 | Hs.169836 | chr2p21 |
| 208119_s_at | zinc finger protein 93 /// zinc finger protein 93 | ZNF93 | NM_031218 | Hs.301059 | chr19p12 |
| 208118_x_at | SLC7A5 pseudogene /// SLC7A5 pseudogene /// SLC7A5 pseudogene /// SLC7A5 pseudogene /// hypothetical protein LOC440345 /// hypothetical protein LOC440345 /// PI-3-kinase-related kinase SMG-1 pseudogene /// PI-3-kinase-related kinase SMG-1 pseudogene /// P | LAT1-3TM | NM_031211 | Hs.448808 | chr16p12 |
| 208116_s_at | mannosidase, alpha, class 1A, member 1 | MAN1A1 | NM_005907 | Hs.255149 | chr6q22 |
| 208113_x_at | poly(A) binding protein, cytoplasmic 3 /// poly(A) binding protein, cytoplasmic 3 | PABPC3 | NM_030979 | Hs.458280 | chr13q12-q13 |
| 208103_s_at | acidic (leucine-rich) nuclear phosphoprotein 32 family, member E /// acidic (leucine-rich) nuclear phosphoprotein 32 family, member E | ANP32E | NM_030920 | Hs.385913 | chr1q21.2 |
| 208101_s_at | ubiquitin related modifier 1 homolog (S. cerevisiae) /// ubiquitin related modifier 1 homolog (S. cerevisiae) | URM1 | NM_030914 | Hs.406421 | chr9q34.11 |
| 208097_s_at | thioredoxin domain containing 1 /// thioredoxin domain containing 1 | TXNDC1 | NM_030755 | Hs.125221 | chr14q22.1 |
| 208096_s_at | collagen, type XXI, alpha 1 /// collagen, type XXI, alpha 1 | COL21A1 | NM_030820 | Hs.408757 | chr6p12.3-p11.2 |
| 208095_s_at | signal recognition particle 72kDa | SRP72 | NM_001222 | Hs.237825 | chr4q11 |
| 208094_s_at | coiled-coil domain containing 130 /// coiled-coil domain containing 130 | CCDC130 | NM_030818 | Hs.24998 | chr19p13.13 |
| 208093_s_at | nudE nuclear distribution gene E homolog like 1 (A. nidulans) /// nudE nuclear distribution gene E homolog like 1 (A. nidulans) | NDEL1 | NM_030808 | Hs.148576 | chr17p13.1 |
| 208091_s_at | EGFR-coamplified and overexpressed protein /// EGFR-coamplified and overexpressed protein | ECOP | NM_030796 | Hs.4750 | chr7p11.2 |
| 208086_s_at | dystrophin (muscular dystrophy, Duchenne and Becker types) | DMD | M92650 | Hs.495912 | chrxp21.2 |
| 208079_s_at | aurora kinase A | AURKA | NM_003158 | Hs.250822 | chr20q13.2-q13.3 |
| 208078_s_at | SNF1-like kinase /// SNF1-like kinase | SNF1LK | NM_030751 | Hs.380991 | chr21q22.3 |
| 208072_s_at | diacylglycerol kinase, delta 130kDa | DGKD | NM_003648 | Hs.115907 | chr2q37.1 |
| 208070_s_at | REV3-like, catalytic subunit of DNA polymerase zeta (yeast) | REV3L | NM_002912 | Hs.232021 | chr6q21 |
| 208066_s_at | general transcription factor IIB /// general transcription factor IIB | GTF2B | NM_001514 | Hs.258561 | chr1p22-p21 |
| 208055_s_at | hect domain and RLD 4 | HERC4 | NM_015601 | Hs.96867 | chr10q21.3 |
| 208050_s_at | caspase 2, apoptosis-related cysteine peptidase (neural precursor cell expressed, developmentally down-regulated 2) | CASP2 | NM_001224 | Hs.433103 | chr7q34-q35 |
| 208047_s_at | NGFI-A binding protein 1 (EGR1 binding protein 1) | NAB1 | NM_005966 | Hs.107474 | chr2q32.3-q33 |
| 208025_s_at | high mobility group AT-hook 2 /// high mobility group AT-hook 2 | HMGA2 | NM_003483 | Hs.528686 | chr12q15 |
| 208024_s_at | DiGeorge syndrome critical region gene 6 /// DiGeorge syndrome critical region gene 6-like | DGCR6 | NM_005675 | Hs.347285 | chr22q11 /// chr22q11.21 |
| 208002_s_at | acyl-CoA thioesterase 7 | ACOT7 | NM_007274 | Hs.435092 | chr1p36.31-p36.11 |
| 207995_s_at | C-type lectin domain family 4, member M | CLEC4M | NM_014257 | Hs.421437 | chr19p13 |
| 207993_s_at | calcium binding protein P22 | CHP | NM_007236 | Hs.406234 | chr15q13.3 |
| 207992_s_at | adenosine monophosphate deaminase (isoform E) | AMPD3 | NM_000480 | Hs.83918 | chr11p15 |
| 207988_s_at | actin related protein 2/3 complex, subunit 2, 34kDa | ARPC2 | NM_005731 | Hs.83583 | chr2q36.1 |
| 207983_s_at | stromal antigen 2 | STAG2 | NM_006603 | Hs.8217 | chrxq25 |
| 207980_s_at | Cbp/p300-interacting transactivator, with Glu/Asp-rich carboxy-terminal domain, 2 | CITED2 | NM_006079 | Hs.82071 | chr6q23.3 |
| 207971_s_at | centrosomal protein 68kDa | CEP68 | NM_015147 | Hs.534442 | chr2p14 |
| 207945_s_at | casein kinase 1, delta | CSNK1D | NM_001893 | Hs.378918 | chr17q25 |
| 207943_x_at | pleiomorphic adenoma gene-like 1 | PLAGL1 | NM_006718 |  |  |
| 207936_x_at | ret finger protein-like 3 | RFPL3 | NM_006604 | Hs.534424 | chr22q12.3 |
| 207922_s_at | macrophage erythroblast attacher /// similar to macrophage erythroblast attacher | MAEA | NM_005882 | Hs.253067 | chr4p16.3 |
| 207891_s_at | UCHL5 interacting protein | UCHL5IP | NM_017518 | Hs.170835 | chrxq28 |
| 207876_s_at | filamin C, gamma (actin binding protein 280) | FLNC | NM_001458 | Hs.58414 | chr7q32-q35 |
| 207871_s_at | suppression of tumorigenicity 7 | ST7 | NM_018412 | Hs.203557 | chr7q31.1-q31.3 |
| 207869_s_at | calcium channel, voltage-dependent, alpha 1G subunit | CACNA1G | NM_018896 | Hs.194746 | chr17q22 |
| 207856_s_at | sphingomyelin phosphodiesterase 4, neutral membrane (neutral sphingomyelinase-3) /// FLJ41352 protein | SMPD4 | NM_017951 | Hs.94491 | chr2q21.1 /// chr2q21.2 |
| 207855_s_at | chloride channel CLIC-like 1 | CLCC1 | NM_015127 | Hs.534441 | chr1p13.3 |
| 207843_x_at | cytochrome b5 type A (microsomal) | CYB5A | NM_001914 | Hs.83834 | chr18q23 |
| 207842_s_at | cancer susceptibility candidate 3 | CASC3 | NM_007359 | Hs.514852 | chr17q11-q21.3 |
| 207831_x_at | deoxyhypusine synthase | DHPS | NM_013407 | Hs.79064 | chr19p13.2-p13.1 |
| 207830_s_at | protein phosphatase 1, regulatory (inhibitor) subunit 8 | PPP1R8 | NM_002713 | Hs.356590 | chr1p35 |
| 207828_s_at | centromere protein F, 350/400ka (mitosin) | CENPF | NM_005196 | Hs.77204 | chr1q32-q41 |
| 207815_at | platelet factor 4 variant 1 | PF4V1 | NM_002620 | Hs.72933 | chr4q12-q21 |
| 207813_s_at | ferredoxin reductase | FDXR | NM_004110 | Hs.69745 | chr17q24-q25 |
| 207809_s_at | ATPase, H+ transporting, lysosomal accessory protein 1 | ATP6AP1 | NM_001183 | Hs.6551 | chrxq28 |
| 207808_s_at | protein S (alpha) | PROS1 | NM_000313 | Hs.64016 | chr3q11.2 |
| 207805_s_at | proteasome (prosome, macropain) 26S subunit, non-ATPase, 9 | PSMD9 | NM_002813 | Hs.131151 | chr12q24.31-q24.32 |
| 207785_s_at | recombining binding protein suppressor of hairless (Drosophila) | RBPSUH | NM_015874 | Hs.347340 | chr4p15.2 |
| 207782_s_at | presenilin 1 (Alzheimer disease 3) | PSEN1 | NM_007319 | Hs.3260 | chr14q24.3 |
| 207769_s_at | polyglutamine binding protein 1 | PQBP1 | NM_005710 | Hs.30570 | chrxp11.23 |
| 207767_s_at | early growth response 4 | EGR4 | NM_001965 | Hs.26744 | chr2p13 |
| 207765_s_at | KIAA1539 | KIAA1539 | NM_025182 | Hs.301696 | chr9p13.3 |
| 207760_s_at | nuclear receptor co-repressor 2 | NCOR2 | NM_006312 | Hs.287994 | chr12q24 |
| 207753_at | zinc finger protein 304 | ZNF304 | NM_020657 | Hs.287374 | chr19q13.4 |
| 207752_x_at | proline-rich protein BstNI subfamily 1 /// proline-rich protein BstNI subfamily 2 | PRB1 | NM_005039 | Hs.525288 | chr12p13.2 |
| 207738_s_at | NCK-associated protein 1 | NCKAP1 | NM_013436 | Hs.278411 | chr2q32 |
| 207730_x_at | Hepatoma-derived growth factor-related protein 2 | HDGF2 | NM_017932 | Hs.406701 |  |
| 207727_s_at | mutY homolog (E. coli) | MUTYH | NM_012222 | Hs.271353 | chr1p34.3-p32.1 |
| 207724_s_at | spastin | SPAST | NM_014946 | Hs.512701 | chr2p24-p21 |
| 207717_s_at | plakophilin 2 | PKP2 | NM_004572 | Hs.25051 | chr12p11 |
| 207714_s_at | serpin peptidase inhibitor, clade H (heat shock protein 47), member 1, (collagen binding protein 1) | SERPINH1 | NM_004353 | Hs.241579 | chr11q13.5 |
| 207713_s_at | RanBP-type and C3HC4-type zinc finger containing 1 | RBCK1 | NM_006462 | Hs.247280 | chr20p13 |
| 207707_s_at | SEC13 homolog (S. cerevisiae) | SEC13 | NM_030673 | Hs.227949 | chr3p25-p24 |
| 207705_s_at | KIAA0980 protein | RP4-691N24.1 | NM_025176 | Hs.227743 | chr20p11.22-p11.1 |
| 207688_s_at | inhibin, beta C | INHBC | NM_005538 |  |  |
| 207668_x_at | protein disulfide isomerase family A, member 6 | PDIA6 | NM_005742 | Hs.212102 | chr2p25.1 |
| 207667_s_at | mitogen-activated protein kinase kinase 3 | MAP2K3 | NM_002756 | Hs.180533 | chr17q11.2 |
| 207657_x_at | transportin 1 | TNPO1 | NM_002270 | Hs.405954 | chr5q13.2 |
| 207654_x_at | down-regulator of transcription 1, TBP-binding (negative cofactor 2) | DR1 | NM_001938 | Hs.348418 | chr1p22.1 |
| 207643_s_at | tumor necrosis factor receptor superfamily, member 1A | TNFRSF1A | NM_001065 | Hs.159 | chr12p13.2 |
| 207629_s_at | rho/rac guanine nucleotide exchange factor (GEF) 2 | ARHGEF2 | NM_004723 | Hs.337774 | chr1q21-q22 |
| 207628_s_at | Williams Beuren syndrome chromosome region 22 | WBSCR22 | NM_017528 | Hs.413036 | chr7q11.23 |
| 207625_s_at | core-binding factor, runt domain, alpha subunit 2; translocated to, 2 | CBFA2T2 | NM_005093 | Hs.153934 | chr20q11 |
| 207624_s_at | retinitis pigmentosa GTPase regulator | RPGR | NM_000328 | Hs.378949 | chrxp11.4 |
| 207622_s_at | ATP-binding cassette, sub-family F (GCN20), member 2 | ABCF2 | NM_005692 | Hs.438823 | chr7q36 |
| 207621_s_at | phosphatidylethanolamine N-methyltransferase | PEMT | NM_007169 | Hs.15192 | chr17p11.2 |
| 207618_s_at | BCS1-like (yeast) | BCS1L | NM_004328 | Hs.150922 | chr2q33 |
| 207608_x_at | cytochrome P450, family 1, subfamily A, polypeptide 2 | CYP1A2 | NM_000761 | Hs.1361 | chr15q22-qter |
| 207606_s_at | Rho GTPase activating protein 12 | ARHGAP12 | NM_018287 | Hs.396643 | chr10q11.1 |
| 207598_x_at | X-ray repair complementing defective repair in Chinese hamster cells 2 | XRCC2 | NM_005431 | Hs.129727 | chr7q36.1 |
| 207585_s_at | ribosomal protein L36a-like | RPL36AL | NM_001001 | Hs.444749 | chr14q21 |
| 207573_x_at | ATP synthase, H+ transporting, mitochondrial F0 complex, subunit G | ATP5L | NM_006476 | Hs.107476 | chr11q23.3 |
| 207571_x_at | chromosome 1 open reading frame 38 | C1orf38 | NM_004848 | Hs.10649 | chr1p35.3 |
| 207563_s_at | O-linked N-acetylglucosamine (GlcNAc) transferase (UDP-N-acetylglucosamine:polypeptide-N-acetylglucosaminyl transferase) | OGT | U77413 | Hs.405410 | chrxq13 |
| 207558_s_at | paired-like homeodomain transcription factor 2 | PITX2 | NM_000325 | Hs.92282 | chr4q25-q27 |
| 207556_s_at | diacylglycerol kinase, zeta 104kDa | DGKZ | NM_003646 | Hs.277445 | chr11p11.2 |
| 207551_s_at | male-specific lethal 3-like 1 (Drosophila) | MSL3L1 | NM_006800 | Hs.371233 | chrxp22.3 |
| 207543_s_at | procollagen-proline, 2-oxoglutarate 4-dioxygenase (proline 4-hydroxylase), alpha polypeptide I | P4HA1 | NM_000917 | Hs.76768 | chr10q21.3-q23.1 |
| 207541_s_at | exosome component 10 | EXOSC10 | NM_002685 | Hs.321234 | chr1p36.22 |
| 207535_s_at | nuclear factor of kappa light polypeptide gene enhancer in B-cells 2 (p49/p100) | NFKB2 | NM_002502 | Hs.73090 | chr10q24 |
| 207526_s_at | interleukin 1 receptor-like 1 | IL1RL1 | NM_003856 | Hs.66 | chr2q12 |
| 207525_s_at | GIPC PDZ domain containing family, member 1 | GIPC1 | NM_005716 | Hs.396978 | chr19p13.1 |
| 207520_at | TROVE domain family, member 2 | TROVE2 | BG494940 | Hs.288178 | chr1q31 |
| 207515_s_at | polymerase (RNA) I polypeptide C, 30kDa | POLR1C | NM_004875 | Hs.5409 | chr6p21.1 |
| 207513_s_at | zinc finger protein 189 | ZNF189 | NM_003452 | Hs.50123 | chr9q22-q31 |
| 207480_s_at | Meis1, myeloid ecotropic viral integration site 1 homolog 2 (mouse) | MEIS2 | NM_020149 | Hs.362805 | chr15q14 |
| 207469_s_at | pirin (iron-binding nuclear protein) | PIR | NM_003662 | Hs.424966 | chrxp22.2 |
| 207468_s_at | secreted frizzled-related protein 5 | SFRP5 | NM_003015 | Hs.523195 | chr10q24.1 |
| 207467_x_at | calpastatin | CAST | NM_001750 | Hs.440961 | chr5q15-q21 |
| 207439_s_at | solute carrier family 35 (UDP-galactose transporter), member A2 | SLC35A2 | NM_005660 | Hs.21899 | chrxp11.23-p11.22 |
| 207438_s_at | snurportin 1 | SNUPN | NM_005701 | Hs.21577 | chr15q23 |
| 207435_s_at | serine/arginine repetitive matrix 2 | SRRM2 | NM_016333 | Hs.433343 | chr16p13.3 |
| 207431_s_at | degenerative spermatocyte homolog 1, lipid desaturase (Drosophila) | DEGS1 | NM_003676 | Hs.299878 | chr1q42.12 |
| 207428_x_at | cell division cycle 2-like 1 (PITSLRE proteins) /// similar to cell division cycle 2-like 1 (PITSLRE proteins) isoform 1 | CDC2L1 | NM_001787 | Hs.192316 | chr1p36 |
| 207426_s_at | tumor necrosis factor (ligand) superfamily, member 4 (tax-transcriptionally activated glycoprotein 1, 34kDa) | TNFSF4 | NM_003326 | Hs.181097 | chr1q25 |
| 207417_s_at | zinc finger protein 177 | ZNF177 | NM_003451 | Hs.172979 | chr19p13.2 |
| 207394_at | zinc finger protein 137 | ZNF137 | NM_003438 | Hs.373648 | chr19q13.4 |
| 207391_s_at | phosphatidylinositol-4-phosphate 5-kinase, type I, alpha | PIP5K1A | NM_003557 | Hs.149255 | chr1q22-q24 |
| 207388_s_at | prostaglandin E synthase | PTGES | NM_004878 | Hs.146688 | chr9q34.3 |
| 207358_x_at | microtubule-actin crosslinking factor 1 | MACF1 | NM_012090 | Hs.372463 | chr1p32-p31 |
| 207350_s_at | vesicle-associated membrane protein 4 | VAMP4 | NM_003762 | Hs.6651 | chr1q24-q25 |
| 207332_s_at | transferrin receptor (p90, CD71) | TFRC | NM_003234 | Hs.185726 | chr3q29 |
| 207327_at | eyes absent homolog 4 (Drosophila) | EYA4 | NM_004100 | Hs.364499 | chr6q23 |
| 207326_at | betacellulin | BTC | NM_001729 | Hs.73105 | chr4q13-q21 |
| 207320_x_at | staufen, RNA binding protein, homolog 1 (Drosophila) | STAU1 | NM_004602 | Hs.6113 | chr20q13.1 |
| 207319_s_at | cell division cycle 2-like 5 (cholinesterase-related cell division controller) | CDC2L5 | NM_003718 | Hs.404501 | chr7p13 |
| 207305_s_at | KIAA1012 | KIAA1012 | NM_014939 | Hs.42959 | chr18q12.1 |
| 207283_at | ribosomal protein L23a pseudogene 13 | RPL23AP13 | NM_020217 | Hs.534542 | chr2p16.3 |
| 207275_s_at | acyl-CoA synthetase long-chain family member 1 | ACSL1 | NM_001995 | Hs.406678 | chr4q34-q35 |
| 207268_x_at | abl interactor 2 | ABI2 | NM_005759 | Hs.387906 | chr2q33 |
| 207265_s_at | KDEL (Lys-Asp-Glu-Leu) endoplasmic reticulum protein retention receptor 3 | KDELR3 | NM_016657 | Hs.528305 | chr22q13.1 |
| 207253_s_at | ubinuclein 1 | UBN1 | NM_016936 | Hs.21479 | chr16p13.3 |
| 207240_s_at | luteinizing hormone/choriogonadotropin receptor | LHCGR | NM_000233 | Hs.166604 | chr2p21 |
| 207233_s_at | microphthalmia-associated transcription factor | MITF | NM_000248 | Hs.166017 | chr3p14.2-p14.1 |
| 207231_at | zinc finger DAZ interacting protein 3 | DZIP3 | NM_014648 | Hs.409210 | chr3q13.13 |
| 207219_at | zinc finger protein 643 | ZNF643 | NM_023070 | Hs.133034 | chr1p34.2 |
| 207198_s_at | LIM and senescent cell antigen-like domains 1 | LIMS1 | NM_004987 | Hs.112378 | chr2q12.3-q13 |
| 207196_s_at | TNFAIP3 interacting protein 1 | TNIP1 | NM_006058 | Hs.355141 | chr5q32-q33.1 |
| 207181_s_at | caspase 7, apoptosis-related cysteine peptidase | CASP7 | NM_001227 | Hs.9216 | chr10q25 |
| 207170_s_at | LETM1 domain containing 1 | LETMD1 | NM_015416 | Hs.370457 | chr12q13.12 |
| 207168_s_at | H2A histone family, member Y | H2AFY | NM_004893 | Hs.75258 | chr5q31.3-q32 |
| 207163_s_at | v-akt murine thymoma viral oncogene homolog 1 | AKT1 | NM_005163 | Hs.368861 | chr14q32.32 |
| 207157_s_at | guanine nucleotide binding protein (G protein), gamma 5 | GNG5 | NM_005274 | Hs.436765 | chr1p22 |
| 207156_at | histone cluster 1, H2ag | HIST1H2AG | NM_021064 | Hs.51011 | chr6p22.1 |
| 207153_s_at | glomulin, FKBP associated protein | GLMN | NM_007070 | Hs.49105 | chr1p22.1 |
| 207149_at | cadherin 12, type 2 (N-cadherin 2) | CDH12 | L33477 | Hs.504149 | chr5p14-p13 |
| 207144_s_at | Cbp/p300-interacting transactivator, with Glu/Asp-rich carboxy-terminal domain, 1 | CITED1 | NM_004143 | Hs.40403 | chrxq13.1 |
| 207132_x_at | prefoldin subunit 5 | PFDN5 | NM_002624 | Hs.288856 | chr12q12 |
| 207131_x_at | gamma-glutamyltransferase 1 | GGT1 | NM_013430 | Hs.352119 | chr22q11.23 |
| 207128_s_at | zinc finger protein 223 | ZNF223 | NM_013361 |  |  |
| 207124_s_at | guanine nucleotide binding protein (G protein), beta 5 | GNB5 | NM_006578 | Hs.155090 | chr15q21.2 |
| 207121_s_at | mitogen-activated protein kinase 6 | MAPK6 | NM_002748 | Hs.271980 | chr15q21 |
| 207120_at | zinc finger protein 667 | ZNF667 | NM_022103 | Hs.433473 | chr19q13.43 |
| 207103_at | potassium voltage-gated channel, Shal-related subfamily, member 2 | KCND2 | NM_012281 | Hs.21703 | chr7q31 |
| 207098_s_at | mitofusin 1 | MFN1 | NM_017927 | Hs.135624 | chr3q26.32 |
| 207088_s_at | solute carrier family 25 (mitochondrial carrier; oxoglutarate carrier), member 11 | SLC25A11 | NM_003562 | Hs.184877 | chr17p13.3 |
| 207081_s_at | phosphatidylinositol 4-kinase, catalytic, alpha polypeptide | PIK4CA | NM_002650 | Hs.334874 | chr22q11.21 |
| 207076_s_at | argininosuccinate synthetase 1 | ASS1 | NM_000050 | Hs.160786 | chr9q34.1 |
| 207071_s_at | aconitase 1, soluble /// ankyrin repeat domain 15 | ACO1 | NM_002197 | Hs.319677 | chr9p22-p13 |
| 207064_s_at | amine oxidase, copper containing 2 (retina-specific) | AOC2 | NM_009590 | Hs.143102 | chr17q21 |
| 207040_s_at | suppression of tumorigenicity 13 (colon carcinoma) (Hsp70 interacting protein) | ST13 | NM_003932 | Hs.377199 | chr22q13.2 |
| 207038_at | solute carrier family 16, member 6 (monocarboxylic acid transporter 7) | SLC16A6 | NM_004694 | Hs.42645 | chr17q24.2 |
| 207023_x_at | keratin 10 (epidermolytic hyperkeratosis; keratosis palmaris et plantaris) | KRT10 | NM_000421 | Hs.99936 | chr17q21 |
| 207016_s_at | aldehyde dehydrogenase 1 family, member A2 | ALDH1A2 | AB015228 | Hs.435689 | chr15q21.3 |
| 207012_at | matrix metallopeptidase 16 (membrane-inserted) | MMP16 | U79292 | Hs.103849 | chr8q21 |
| 207011_s_at | PTK7 protein tyrosine kinase 7 | PTK7 | NM_002821 | Hs.90572 | chr6p21.1-p12.2 |
| 207001_x_at | TSC22 domain family, member 3 | TSC22D3 | NM_004089 | Hs.420569 | chrxq22.3 |
| 206998_x_at | proline-rich protein BstNI subfamily 3 | PRB3 | NM_006249 | Hs.525288 | chr12p13.2 |
| 206994_at | cystatin S | CST4 | NM_001899 | Hs.56319 | chr20p11.21 |
| 206976_s_at | heat shock 105kDa/110kDa protein 1 | HSPH1 | NM_006644 | Hs.36927 | chr13q12.3 |
| 206959_s_at | UPF3 regulator of nonsense transcripts homolog A (yeast) | UPF3A | NM_023011 | Hs.399740 | chr13q34 |
| 206956_at | bone gamma-carboxyglutamate (gla) protein (osteocalcin) | BGLAP | NM_000711 | Hs.408848 | chr1q12 /// chr1q25-q31 |
| 206953_s_at | latrophilin 2 | LPHN2 | NM_012302 | Hs.24212 | chr1p31.1 |
| 206950_at | sodium channel, voltage-gated, type IX, alpha | SCN9A | NM_002977 | Hs.2319 | chr2q24 |
| 206949_s_at | RUN and SH3 domain containing 1 | RUSC1 | NM_014328 | Hs.226499 | chr1q21-q22 |
| 206945_at | lactase | LCT | NM_002299 | Hs.2251 | chr2q21 |
| 206929_s_at | nuclear factor I/C (CCAAT-binding transcription factor) | NFIC | NM_005597 | Hs.436639 | chr19p13.3 |
| 206928_at | zinc finger protein 124 | ZNF124 | NM_003431 | Hs.421238 | chr1q44 |
| 206923_at | protein kinase C, alpha | PRKCA | NM_002737 | Hs.349611 | chr17q22-q23.2 |
| 206918_s_at | copine I | CPNE1 | NM_003915 |  | chr20q11.22 |
| 206910_x_at | complement factor H-related 2 | CFHR2 | NM_005666 |  | chr1q31-q32.1 |
| 206900_x_at | zinc finger protein 253 | ZNF253 | NM_021047 | Hs.407162 | chr19p12 /// chr19p13.11 |
| 206875_s_at | STE20-like kinase (yeast) | SLK | NM_014720 | Hs.105751 | chr10q25.1 |
| 206861_s_at | CGG triplet repeat binding protein 1 | CGGBP1 | NM_003663 | Hs.444818 | chr3p12-p11.1 |
| 206860_s_at | hypothetical protein FLJ20323 | FLJ20323 | NM_019005 | Hs.387140 | chr7p22-p21 |
| 206858_s_at | homeobox C6 | HOXC6 | NM_004503 | Hs.224601 | chr12q13.3 |
| 206857_s_at | FK506 binding protein 1B, 12.6 kDa | FKBP1B | NM_004116 | Hs.306834 | chr2p23.3 |
| 206855_s_at | hyaluronoglucosaminidase 2 | HYAL2 | NM_003773 | Hs.76873 | chr3p21.3 |
| 206848_at | homeobox A7 | HOXA7 | NM_006896 |  |  |
| 206846_s_at | histone deacetylase 6 | HDAC6 | NM_006044 | Hs.6764 | chrxp11.23 |
| 206845_s_at | ring finger protein 40 | RNF40 | NM_014771 | Hs.65238 | chr16p11.2-p11.1 |
| 206833_s_at | acylphosphatase 2, muscle type | ACYP2 | NM_001108 | Hs.433071 | chr2p16.2 |
| 206831_s_at | arylsulfatase D | ARSD | NM_001669 | Hs.349326 | chrxp22.3 |
| 206825_at | oxytocin receptor | OXTR | NM_000916 | Hs.2820 | chr3p25 |
| 206818_s_at | cyclin M2 | CNNM2 | NM_017649 | Hs.438588 | chr10q24.33 |
| 206809_s_at | heterogeneous nuclear ribonucleoprotein A3 pseudogene 1 /// heterogeneous nuclear ribonucleoprotein A3 | HNRPA3P1 | NM_005758 | Hs.446430 | chr2q31.2 |
| 206796_at | WNT1 inducible signaling pathway protein 1 | WISP1 | NM_003882 | Hs.194680 | chr8q24.1-q24.3 |
| 206792_x_at | phosphodiesterase 4C, cAMP-specific (phosphodiesterase E1 dunce homolog, Drosophila) | PDE4C | NM_000923 | Hs.437211 | chr19p13.11 |
| 206790_s_at | NADH dehydrogenase (ubiquinone) 1 beta subcomplex, 1, 7kDa | NDUFB1 | NM_004545 | Hs.183435 | chr14q32.12 |
| 206789_s_at | POU domain, class 2, transcription factor 1 | POU2F1 | NM_002697 | Hs.444234 | chr1q22-q23 |
| 206788_s_at | core-binding factor, beta subunit | CBFB | AF294326 | Hs.179881 | chr16q22.1 |
| 206767_at | RNA binding motif, single stranded interacting protein | RBMS3 | NM_014483 | Hs.118358 | chr3p24-p23 |
| 206766_at | integrin, alpha 10 | ITGA10 | AF112345 | Hs.158237 | chr1q21 |
| 206758_at | endothelin 2 | EDN2 | NM_001956 | Hs.1407 | chr1p34 |
| 206756_at | carbohydrate (N-acetylglucosamine 6-O) sulfotransferase 7 | CHST7 | NM_019886 | Hs.138155 | chrxp11.23 |
| 206752_s_at | DNA fragmentation factor, 40kDa, beta polypeptide (caspase-activated DNase) | DFFB | NM_004402 | Hs.133089 | chr1p36.3 |
| 206748_s_at | sperm associated antigen 9 | SPAG9 | NM_003971 | Hs.500367 | chr17q21.33 |
| 206744_s_at | zinc finger, MYM-type 5 | ZMYM5 | NM_014242 | Hs.534410 | chr13q12 |
| 206734_at | jerky homolog-like (mouse) | JRKL | NM_003772 | Hs.105940 | chr11q21 |
| 206707_x_at | chromosome 6 open reading frame 32 | C6orf32 | NM_015864 | Hs.389488 | chr6p22.3-p21.32 |
| 206695_x_at | zinc finger protein 43 | ZNF43 | NM_003423 | Hs.419763 | chr19p13.1-p12 |
| 206688_s_at | cleavage and polyadenylation specific factor 4, 30kDa | CPSF4 | NM_006693 | Hs.434994 | chr7q22.1 |
| 206687_s_at | protein tyrosine phosphatase, non-receptor type 6 | PTPN6 | NM_002831 | Hs.63489 | chr12p13 |
| 206672_at | aquaporin 2 (collecting duct) | AQP2 | NM_000486 | Hs.130730 | chr12q12-q13 |
| 206667_s_at | secretory carrier membrane protein 1 | SCAMP1 | AF005037 | Hs.31218 | chr5q13.3-q14.1 |
| 206656_s_at | chromosome 20 open reading frame 3 | C20orf3 | BC000353 | Hs.22391 | chr20p11.22-p11.21 |
| 206649_s_at | transcription factor binding to IGHM enhancer 3 | TFE3 | NM_006521 | Hs.274184 | chrxp11.22 |
| 206648_at | zinc finger protein 571 | ZNF571 | NM_016536 | Hs.272384 | chr19q13.12 |
| 206645_s_at | nuclear receptor subfamily 0, group B, member 1 | NR0B1 | NM_000475 | Hs.268490 | chrxp21.3-p21.2 |
| 206632_s_at | apolipoprotein B mRNA editing enzyme, catalytic polypeptide-like 3B | APOBEC3B | NM_004900 | Hs.226307 | chr22q13.1-q13.2 |
| 206631_at | prostaglandin E receptor 2 (subtype EP2), 53kDa | PTGER2 | NM_000956 | Hs.2090 | chr14q22 |
| 206621_s_at | eukaryotic translation initiation factor 4H | EIF4H | NM_022170 | Hs.180900 | chr7q11.23 |
| 206613_s_at | TATA box binding protein (TBP)-associated factor, RNA polymerase I, A, 48kDa | TAF1A | NM_005681 | Hs.153088 | chr1q42 |
| 206593_s_at | surfeit 5 | SURF5 | NM_006752 | Hs.78354 | chr9q34.2 |
| 206587_at | chaperonin containing TCP1, subunit 6B (zeta 2) | CCT6B | NM_006584 | Hs.73072 | chr17q12 |
| 206572_x_at | zinc finger protein 85 | ZNF85 | NM_003429 | Hs.37138 | chr19p13.1-p12 |
| 206571_s_at | mitogen-activated protein kinase kinase kinase kinase 4 | MAP4K4 | NM_004834 | Hs.6631 | chr2q11.2-q12 |
| 206565_x_at | SMA3 | SMA3 | NM_006780 | Hs.435018 | chr5q13 |
| 206554_x_at | SET domain and mariner transposase fusion gene | SETMAR | NM_006515 | Hs.265855 | chr3p26.1 |
| 206544_x_at | SWI/SNF related, matrix associated, actin dependent regulator of chromatin, subfamily a, member 2 | SMARCA2 | NM_003070 | Hs.396404 | chr9p22.3 |
| 206540_at | galactosidase, beta 1-like | GLB1L | NM_024506 | Hs.181173 | chr2q35 |
| 206538_at | muscle RAS oncogene homolog | MRAS | NM_012219 | Hs.349227 | chr3q22.3 |
| 206536_s_at | baculoviral IAP repeat-containing 4 | BIRC4 | U32974 | Hs.356076 | chrxq25 |
| 206527_at | 4-aminobutyrate aminotransferase | ABAT | NM_000663 | Hs.1588 | chr16p13.2 |
| 206516_at | anti-Mullerian hormone | AMH | NM_000479 | Hs.112432 | chr19p13.3 |
| 206507_at | zinc finger protein 96 | ZNF96 | NM_014724 | Hs.134816 | chr6p22.2-p21.3 |
| 206506_s_at | suppressor of Ty 3 homolog (S. cerevisiae) | SUPT3H | NM_003599 | Hs.190587 | chr6p21.1-p21.3 |
| 206491_s_at | N-ethylmaleimide-sensitive factor attachment protein, alpha | NAPA | NM_003827 | Hs.75932 | chr19q13.32 |
| 206467_x_at | tumor necrosis factor receptor superfamily, member 6b, decoy /// regulator of telomere elongation helicase 1 | TNFRSF6B | NM_003823 | Hs.434878 | chr20q13.3 |
| 206461_x_at | metallothionein 1H | MT1H | NM_005951 | Hs.438462 | chr16q13 |
| 206456_at | gamma-aminobutyric acid (GABA) A receptor, alpha 5 | GABRA5 | NM_000810 | Hs.24969 | chr15q11.2-q12 |
| 206451_at | TBCC domain containing 1 | TBCCD1 | NM_018138 | Hs.235888 | chr3q27.3 |
| 206447_at | elastase 2A | ELA2A | NM_001971 | Hs.21 | chr1p36.21 |
| 206445_s_at | protein arginine methyltransferase 1 | PRMT1 | NM_001536 | Hs.20521 | chr19q13.3 |
| 206441_s_at | COMM domain containing 4 | COMMD4 | NM_017828 | Hs.351327 | chr15q23 |
| 206439_at | epiphycan | EPYC | NM_004950 | Hs.506285 | chr12q21 |
| 206432_at | hyaluronan synthase 2 | HAS2 | NM_005328 | Hs.159226 | chr8q24.12 |
| 206429_at | coagulation factor II (thrombin) receptor-like 1 | F2RL1 | NM_005242 | Hs.154299 | chr5q13 |
| 206425_s_at | transient receptor potential cation channel, subfamily C, member 3 | TRPC3 | NM_003305 | Hs.150981 | chr4q27 |
| 206416_at | zinc finger protein 205 /// zinc finger protein 205 | ZNF205 | NM_003456 | Hs.522143 | chr16p13.3 |
| 206414_s_at | development and differentiation enhancing factor 2 | DDEF2 | NM_003887 | Hs.200666 | chr2p25 |
| 206412_at | fer (fps/fes related) tyrosine kinase (phosphoprotein NCP94) | FER | NM_005246 | Hs.121558 | chr5q21 |
| 206411_s_at | v-abl Abelson murine leukemia viral oncogene homolog 2 (arg, Abelson-related gene) | ABL2 | NM_007314 | Hs.159472 | chr1q24-q25 |
| 206405_x_at | ubiquitin specific peptidase 6 (Tre-2 oncogene) | USP6 | NM_004505 | Hs.448851 | chr17q11 |
| 206392_s_at | retinoic acid receptor responder (tazarotene induced) 1 | RARRES1 | NM_002888 | Hs.82547 | chr3q25.32 |
| 206385_s_at | ankyrin 3, node of Ranvier (ankyrin G) | ANK3 | NM_020987 | Hs.440478 | chr10q21 |
| 206383_s_at | GTPase activating protein (SH3 domain) binding protein 2 | G3BP2 | NM_012297 | Hs.303676 | chr4q21.1 |
| 206375_s_at | heat shock 27kDa protein 3 | HSPB3 | NM_006308 | Hs.41707 | chr5q11.2 |
| 206374_at | dual specificity phosphatase 8 | DUSP8 | NM_004420 | Hs.41688 | chr11p15.5 |
| 206363_at | v-maf musculoaponeurotic fibrosarcoma oncogene homolog (avian) | MAF | NM_005360 | Hs.134859 | chr16q22-q23 |
| 206352_s_at | peroxisome biogenesis factor 10 | PEX10 | AB013818 | Hs.449936 | chr1p36.32 |
| 206346_at | prolactin receptor | PRLR | NM_000949 | Hs.212892 | chr5p14-p13 |
| 206336_at | chemokine (C-X-C motif) ligand 6 (granulocyte chemotactic protein 2) | CXCL6 | NM_002993 | Hs.164021 | chr4q21 |
| 206335_at | galactosamine (N-acetyl)-6-sulfate sulfatase (Morquio syndrome, mucopolysaccharidosis type IVA) | GALNS | NM_000512 | Hs.271383 | chr16q24.3 |
| 206332_s_at | interferon, gamma-inducible protein 16 | IFI16 | NM_005531 | Hs.370873 | chr1q22 |
| 206323_x_at | oligophrenin 1 | OPHN1 | NM_002547 | Hs.128824 | chrxq12 |
| 206314_at | zinc finger protein 167 | ZNF167 | NM_018651 | Hs.1148 | chr3p22.3-p21.1 |
| 206308_at | tRNA aspartic acid methyltransferase 1 | TRDMT1 | AJ223333 | Hs.351665 | chr10p15.1 |
| 206306_at | ryanodine receptor 3 | RYR3 | NM_001036 | Hs.427717 | chr15q14-q15 |
| 206303_s_at | nudix (nucleoside diphosphate linked moiety X)-type motif 4 /// nudix (nucleoside diphosphate linked moiety X)-type motif 4 pseudogene 1 | NUDT4 | AF191653 | Hs.355399 | chr12q21 |
| 206294_at | hydroxy-delta-5-steroid dehydrogenase, 3 beta- and steroid delta-isomerase 2 | HSD3B2 | NM_000198 | Hs.825 | chr1p13.1 |
| 206290_s_at | regulator of G-protein signalling 7 | RGS7 | NM_002924 | Hs.130171 | chr1q43 |
| 206288_at | protein geranylgeranyltransferase type I, beta subunit | PGGT1B | NM_005023 | Hs.254006 | chr5q22.3 |
| 206279_at | protein kinase, Y-linked | PRKY | NM_002760 | Hs.183165 | chryp11.2 |
| 206272_at | S-phase response (cyclin-related) | SPHAR | NM_006542 | Hs.511754 | chr1q42.11-q42.3 |
| 206271_at | toll-like receptor 3 | TLR3 | NM_003265 | Hs.29499 | chr4q35 |
| 206261_at | zinc finger protein 239 | ZNF239 | NM_005674 | Hs.25040 | chr10q11.22-q11.23 |
| 206249_at | mitogen-activated protein kinase kinase kinase 13 | MAP3K13 | NM_004721 | Hs.311106 | chr3q27 |
| 206240_s_at | zinc finger protein 136 | ZNF136 | NM_003437 | Hs.479874 | chr19p13.2-p13.12 |
| 206235_at | ligase IV, DNA, ATP-dependent | LIG4 | NM_002312 | Hs.166091 | chr13q33-q34 |
| 206227_at | cartilage intermediate layer protein, nucleotide pyrophosphohydrolase | CILP | NM_003613 | Hs.442180 | chr15q22 |
| 206209_s_at | carbonic anhydrase IV | CA4 | NM_000717 | Hs.89485 | chr17q23 |
| 206204_at | growth factor receptor-bound protein 14 | GRB14 | NM_004490 | Hs.411881 | chr2q22-q24 |
| 206197_at | non-metastatic cells 5, protein expressed in (nucleoside-diphosphate kinase) | NME5 | NM_003551 | Hs.72050 | chr5q31 |
| 206194_at | homeobox C4 | HOXC4 | AW299598 |  | chr12q13.3 |
| 206189_at | unc-5 homolog C (C. elegans) | UNC5C | NM_003728 | Hs.125605 | chr4q21-q23 |
| 206188_at | zinc finger protein 623 | ZNF623 | NM_014789 | Hs.43133 | chr8q24.3 |
| 206184_at | v-crk sarcoma virus CT10 oncogene homolog (avian)-like | CRKL | NM_005207 | Hs.5613 | chr22q11.21 |
| 206183_s_at | hect domain and RLD 3 | HERC3 | NM_014606 | Hs.35804 | chr4q21 |
| 206182_at | zinc finger protein 134 | ZNF134 | NM_003435 | Hs.469694 | chr19q13.4 |
| 206175_x_at | zinc finger protein 222 | ZNF222 | NM_013360 | Hs.279840 | chr19q13.2 |
| 206174_s_at | protein phosphatase 6, catalytic subunit | PPP6C | NM_002721 | Hs.495128 | chr9q33.3 |
| 206167_s_at | Rho GTPase activating protein 6 | ARHGAP6 | NM_001174 | Hs.250830 | chrxp22.3 |
| 206163_at | mab-21-like 1 (C. elegans) | MAB21L1 | NM_005584 | Hs.507743 | chr13q13 |
| 206158_s_at | CCHC-type zinc finger, nucleic acid binding protein | CNBP | NM_003418 | Hs.2110 | chr3q21 |
| 206157_at | pentraxin-related gene, rapidly induced by IL-1 beta | PTX3 | NM_002852 | Hs.127657 | chr3q25 |
| 206147_x_at | sex comb on midleg-like 2 (Drosophila) | SCML2 | NM_006089 | Hs.171558 | chrxp22 |
| 206144_at | membrane associated guanylate kinase, WW and PDZ domain containing 1 | MAGI1 | NM_004742 | Hs.169441 | chr3p14.1 |
| 206141_at | molybdenum cofactor synthesis 3 | MOCS3 | NM_014484 | Hs.159410 | chr20q13.13 |
| 206137_at | regulating synaptic membrane exocytosis 2 | RIMS2 | NM_014677 | Hs.153610 | chr8q22.3 |
| 206133_at | XIAP associated factor-1 | BIRC4BP | NM_017523 | Hs.441975 | chr17p13.1 |
| 206116_s_at | tropomyosin 1 (alpha) | TPM1 | NM_000366 | Hs.133892 | chr15q22.1 |
| 206115_at | early growth response 3 | EGR3 | NM_004430 | Hs.74088 | chr8p23-p21 |
| 206113_s_at | RAB5A, member RAS oncogene family | RAB5A | NM_004162 | Hs.73957 | chr3p24-p22 |
| 206110_at | histone cluster 1, H3h | HIST1H3H | NM_003536 | Hs.70937 | chr6p22-p21.3 |
| 206108_s_at | splicing factor, arginine/serine-rich 6 | SFRS6 | NM_006275 | Hs.6891 | chr20q12-q13.1 |
| 206105_at | AF4/FMR2 family, member 2 | AFF2 | NM_002025 | Hs.54472 | chrxq28 |
| 206101_at | extracellular matrix protein 2, female organ and adipocyte specific | ECM2 | NM_001393 | Hs.117060 | chr9q22.3 |
| 206098_at | zinc finger and BTB domain containing 6 | ZBTB6 | NM_006626 | Hs.3053 | chr9q33.2 |
| 206096_at | zinc finger protein 35 | ZNF35 | AI809774 | Hs.288658 | chr3p22-p21 |
| 206091_at | matrilin 3 | MATN3 | NM_002381 | Hs.6985 | chr2p24-p23 |
| 206090_s_at | disrupted in schizophrenia 1 | DISC1 | NM_018662 | Hs.26985 | chr1q42.1 |
| 206085_s_at | cystathionase (cystathionine gamma-lyase) | CTH | NM_001902 | Hs.19904 | chr1p31.1 |
| 206066_s_at | RAD51 homolog C (S. cerevisiae) | RAD51C | NM_002876 | Hs.412587 | chr17q22-q23 |
| 206061_s_at | Dicer1, Dcr-1 homolog (Drosophila) | DICER1 | NM_030621 | Hs.87889 | chr14q32.13 |
| 206052_s_at | stem-loop (histone) binding protein | SLBP | NM_006527 | Hs.251574 | chr4p16.3 |
| 206050_s_at | ribonuclease/angiogenin inhibitor 1 | RNH1 | NM_002939 | Hs.130958 | chr11p15.5 |
| 206046_at | ADAM metallopeptidase domain 23 | ADAM23 | NM_003812 | Hs.432317 | chr2q33 |
| 206044_s_at | v-raf murine sarcoma viral oncogene homolog B1 | BRAF | NM_004333 | Hs.162967 | chr7q34 |
| 206039_at | RAB33A, member RAS oncogene family | RAB33A | NM_004794 | Hs.56294 | chrxq25 |
| 206036_s_at | v-rel reticuloendotheliosis viral oncogene homolog (avian) | REL | NM_002908 | Hs.44313 | chr2p13-p12 |
| 206029_at | ankyrin repeat domain 1 (cardiac muscle) | ANKRD1 | NM_014391 | Hs.448589 | chr10q23.31 |
| 206022_at | Norrie disease (pseudoglioma) | NDP | NM_000266 | Hs.2839 | chrxp11.4 |
| 206015_s_at | forkhead box J3 | FOXJ3 | NM_014947 | Hs.26023 | chr1pter-q31.3 |
| 206012_at | left-right determination factor 2 | LEFTY2 | NM_003240 | Hs.25195 | chr1q42.1 |
| 206009_at | integrin, alpha 9 | ITGA9 | NM_002207 | Hs.8042 | chr3p21.3 |
| 206003_at | centrosomal protein 135kDa | CEP135 | NM_014645 | Hs.185091 | chr4q12 |
| 205998_x_at | cytochrome P450, family 3, subfamily A, polypeptide 4 | CYP3A4 | NM_017460 | Hs.442527 | chr7q21.1 |
| 205996_s_at | adenylate kinase 2 | AK2 | NM_013411 | Hs.294008 | chr1p34 |
| 205992_s_at | interleukin 15 | IL15 | NM_000585 | Hs.528402 | chr4q31 |
| 205984_at | corticotropin releasing hormone binding protein | CRHBP | NM_001882 | Hs.115617 | chr5q11.2-q13.3 |
| 205981_s_at | inhibitor of growth family, member 2 | ING2 | NM_001564 | Hs.107153 | chr4q35.1 |
| 205978_at | klotho | KL | NM_004795 | Hs.171062 | chr13q12 |
| 205969_at | arylacetamide deacetylase (esterase) | AADAC | NM_001086 | Hs.444003 | chr3q21.3-q25.2 |
| 205967_at | histone cluster 1, H4c | HIST1H4C | NM_003542 | Hs.46423 | chr6p21.3 |
| 205966_at | TAF13 RNA polymerase II, TATA box binding protein (TBP)-associated factor, 18kDa | TAF13 | NM_005645 | Hs.502508 | chr1p13.3 |
| 205964_at | zinc finger protein 426 | ZNF426 | NM_024106 | Hs.324978 | chr19p13.2 |
| 205963_s_at | DnaJ (Hsp40) homolog, subfamily A, member 3 | DNAJA3 | NM_005147 | Hs.459779 | chr16p13.3 |
| 205953_at | leucine-rich repeats and immunoglobulin-like domains 2 | LRIG2 | NM_014813 | Hs.448972 | chr1p13.1 |
| 205952_at | potassium channel, subfamily K, member 3 | KCNK3 | NM_002246 | Hs.24040 | chr2p23 |
| 205945_at | interleukin 6 receptor /// interleukin 6 receptor | IL6R | NM_000565 | Hs.193400 | chr1q21 |
| 205934_at | phospholipase C-like 1 | PLCL1 | NM_006226 | Hs.153322 | chr2q33 |
| 205930_at | general transcription factor IIE, polypeptide 1, alpha 56kDa | GTF2E1 | NM_005513 | Hs.145381 | chr3q21-q24 |
| 205928_at | zinc finger protein 443 | ZNF443 | NM_005815 | Hs.142150 | chr19p13.2 |
| 205917_at | zinc finger protein 264 | ZNF264 | NM_003417 | Hs.426358 | chr19q13.4 |
| 205905_s_at | MHC class I polypeptide-related sequence A /// MHC class I polypeptide-related sequence B | MICA | NM_000247 | Hs.98631 | chr6p21.3 |
| 205899_at | cyclin A1 | CCNA1 | NM_003914 | Hs.417050 | chr13q12.3-q13 |
| 205893_at | neuroligin 1 | NLGN1 | NM_014932 | Hs.368087 | chr3q26.31 |
| 205891_at | adenosine A2b receptor | ADORA2B | NM_000676 | Hs.45743 | chr17p12-p11.2 |
| 205889_s_at | janus kinase and microtubule interacting protein 2 | JAKMIP2 | NM_014790 |  |  |
| 205883_at | zinc finger and BTB domain containing 16 | ZBTB16 | NM_006006 | Hs.292207 | chr11q23.1 |
| 205880_at | protein kinase D1 | PRKD1 | NM_002742 | Hs.2891 | chr14q11 |
| 205876_at | leukemia inhibitory factor receptor alpha | LIFR | NM_002310 | Hs.446501 | chr5p13-p12 |
| 205862_at | GREB1 protein | GREB1 | NM_014668 | Hs.438037 | chr2p25.1 |
| 205851_at | non-metastatic cells 6, protein expressed in (nucleoside-diphosphate kinase) | NME6 | BC001808 | Hs.465558 | chr3p21 |
| 205841_at | Janus kinase 2 (a protein tyrosine kinase) | JAK2 | NM_004972 | Hs.434374 | chr9p24 |
| 205835_s_at | YTH domain containing 2 | YTHDC2 | AW975818 | Hs.283283 | chr5q22.2 |
| 205830_at | calmegin | CLGN | NM_004362 | Hs.86368 | chr4q28.3-q31.1 |
| 205822_s_at | 3-hydroxy-3-methylglutaryl-Coenzyme A synthase 1 (soluble) | HMGCS1 | NM_002130 | Hs.397729 | chr5p14-p13 |
| 205812_s_at | transmembrane emp24 protein transport domain containing 9 | TMED9 | NM_006588 | Hs.279929 | chr5q35.3 |
| 205809_s_at | Wiskott-Aldrich syndrome-like | WASL | BE504979 | Hs.182469 | chr7q31.3 |
| 205807_s_at | tuftelin 1 | TUFT1 | NM_020127 | Hs.283009 | chr1q21 |
| 205805_s_at | receptor tyrosine kinase-like orphan receptor 1 | ROR1 | NM_005012 | Hs.445242 | chr1p32-p31 |
| 205803_s_at | transient receptor potential cation channel, subfamily C, member 1 | TRPC1 | NM_003304 | Hs.250687 | chr3q22-q24 |
| 205798_at | interleukin 7 receptor /// interleukin 7 receptor | IL7R | NM_002185 | Hs.362807 | chr5p13 |
| 205796_at | t-complex 11 (mouse)-like 1 | TCP11L1 | NM_018393 | Hs.22383 | chr11p13 |
| 205794_s_at | neuro-oncological ventral antigen 1 | NOVA1 | NM_002515 | Hs.292511 | chr14q |
| 205792_at | WNT1 inducible signaling pathway protein 2 | WISP2 | NM_003881 | Hs.194679 | chr20q12-q13.1 |
| 205781_at | chromosome 16 open reading frame 7 | C16orf7 | NM_004913 | Hs.164410 | chr16q24 |
| 205777_at | dual specificity phosphatase 9 | DUSP9 | NM_001395 | Hs.144879 | chrxq28 |
| 205775_at | family with sequence similarity 50, member B | FAM50B | NM_012135 | Hs.140944 | chr6p25-pter |
| 205771_s_at | A kinase (PRKA) anchor protein 7 | AKAP7 | AL137063 | Hs.12835 | chr6q23 |
| 205770_at | glutathione reductase | GSR | NM_000637 | Hs.414334 | chr8p21.1 |
| 205767_at | epiregulin | EREG | NM_001432 | Hs.115263 | chr4q13.3 |
| 205762_s_at | dihydrouridine synthase 4-like (S. cerevisiae) | DUS4L | NM_007016 | Hs.97627 | chr7q22-q31 |
| 205760_s_at | 8-oxoguanine DNA glycosylase | OGG1 | NM_016821 | Hs.380271 | chr3p26.2 |
| 205756_s_at | coagulation factor VIII, procoagulant component (hemophilia A) | F8 | NM_000132 | Hs.413083 | chrxq28 |
| 205750_at | biphenyl hydrolase-like (serine hydrolase; breast epithelial mucin-associated antigen) | BPHL | NM_004332 | Hs.351334 | chr6p25 |
| 205746_s_at | ADAM metallopeptidase domain 17 (tumor necrosis factor, alpha, converting enzyme) | ADAM17 | U86755 | Hs.509314 | chr2p25 |
| 205743_at | SH3 and cysteine rich domain | STAC | NM_003149 | Hs.56045 | chr3p22.3 |
| 205742_at | troponin I type 3 (cardiac) | TNNI3 | NM_000363 | Hs.512709 | chr19q13.4 |
| 205740_s_at | hypothetical protein MGC10433 | MGC10433 | NM_024321 | Hs.5086 | chr19q13.12 |
| 205739_x_at | zinc finger protein 588 | ZNF588 | NM_016220 | Hs.50216 | chr7q11.2 |
| 205730_s_at | actin binding LIM protein family, member 3 | ABLIM3 | NM_014945 | Hs.282566 | chr5q32 |
| 205717_x_at | protocadherin gamma subfamily C, 3 /// protocadherin gamma subfamily B, 4 /// protocadherin gamma subfamily A, 8 /// protocadherin gamma subfamily A, 12 /// protocadherin gamma subfamily C, 5 /// protocadherin gamma subfamily C, 4 /// protocadherin gamma | PCDHGC3 | NM_002588 | Hs.283794 | chr5q31 |
| 205709_s_at | CDP-diacylglycerol synthase (phosphatidate cytidylyltransferase) 1 | CDS1 | NM_001263 | Hs.444430 | chr4q21.23 |
| 205707_at | interleukin 17 receptor A | IL17RA | NM_014339 | Hs.129751 | chr22q11.1 |
| 205690_s_at | BUD31 homolog (yeast) | BUD31 | NM_003910 | Hs.380233 | chr7q22.1 |
| 205687_at | ubiquitin-binding protein homolog | UBPH | NM_019116 | Hs.3459 | chr16p12 |
| 205684_s_at | DENN/MADD domain containing 4C | DENND4C | NM_017925 | Hs.249591 | chr9p22.1 |
| 205682_x_at | apolipoprotein M | APOM | NM_019101 | Hs.247323 | chr6p21.33 |
| 205681_at | BCL2-related protein A1 | BCL2A1 | NM_004049 | Hs.227817 | chr15q24.3 |
| 205677_s_at | deleted in lymphocytic leukemia, 1 /// SPANX family, member C | DLEU1 | NM_005887 | Hs.344524 | chr13q14.3 |
| 205672_at | xeroderma pigmentosum, complementation group A | XPA | NM_000380 | Hs.288867 | chr9q22.3 |
| 205668_at | lymphocyte antigen 75 | LY75 | NM_002349 | Hs.153563 | chr2q24 |
| 205667_at | Werner syndrome | WRN | NM_000553 | Hs.269809 | chr8p12-p11.2 |
| 205664_at | KIN, antigenic determinant of recA protein homolog (mouse) | KIN | NM_012311 | Hs.123647 | chr10p15-p14 |
| 205661_s_at | FAD1 flavin adenine dinucleotide synthetase homolog (S. cerevisiae) | FLAD1 | NM_025207 | Hs.118666 | chr1q22 |
| 205652_s_at | tubulin tyrosine ligase-like family, member 1 | TTLL1 | NM_012263 | Hs.122854 | chr22q13.1 |
| 205651_x_at | Rap guanine nucleotide exchange factor (GEF) 4 | RAPGEF4 | NM_007023 | Hs.91971 | chr2q31-q32 |
| 205644_s_at | small nuclear ribonucleoprotein polypeptide G | SNRPG | NM_003096 | Hs.436656 | chr2p13.3 |
| 205641_s_at | TNFRSF1A-associated via death domain | TRADD | NM_003789 | Hs.89862 | chr16q22 |
| 205633_s_at | aminolevulinate, delta-, synthase 1 | ALAS1 | NM_000688 | Hs.78712 | chr3p21.1 |
| 205632_s_at | phosphatidylinositol-4-phosphate 5-kinase, type I, beta | PIP5K1B | NM_003558 | Hs.297604 | chr9q13 |
| 205628_at | primase, polypeptide 2A, 58kDa | PRIM2A | NM_000947 | Hs.440603 | chr6p12-p11.1 |
| 205621_at | alkB, alkylation repair homolog 1 (E. coli) | ALKBH1 | NM_006020 | Hs.94542 | chr14q24 |
| 205618_at | proline rich Gla (G-carboxyglutamic acid) 1 | PRRG1 | NM_000950 | Hs.40637 | chrxp21.1 |
| 205603_s_at | diaphanous homolog 2 (Drosophila) | DIAPH2 | NM_007309 | Hs.226483 | chrxq22 |
| 205594_at | zinc finger protein 652 | ZNF652 | NM_014897 | Hs.190386 | chr17q21.32 |
| 205586_x_at | VGF nerve growth factor inducible | VGF | NM_003378 | Hs.232008 | chr7q22 |
| 205579_at | histamine receptor H1 | HRH1 | NM_000861 | Hs.1570 | chr3p25 |
| 205573_s_at | sorting nexin 7 | SNX7 | NM_015976 | Hs.127241 | chr1p21.3 |
| 205572_at | angiopoietin 2 | ANGPT2 | NM_001147 | Hs.115181 | chr8p23.1 |
| 205571_at | lipoyltransferase 1 | LIPT1 | NM_015929 | Hs.112356 | chr2q11.2 |
| 205570_at | phosphatidylinositol-4-phosphate 5-kinase, type II, alpha | PIP5K2A | NM_005028 | Hs.435008 | chr10p12.32 |
| 205567_at | carbohydrate (keratan sulfate Gal-6) sulfotransferase 1 | CHST1 | NM_003654 | Hs.104576 | chr11p11.2-p11.1 |
| 205566_at | abhydrolase domain containing 2 | ABHD2 | NM_007011 | Hs.412022 | chr15q26.1 |
| 205565_s_at | frataxin | FXN | NM_000144 | Hs.360041 | chr9q13-q21.1 |
| 205562_at | ribonuclease P/MRP 38kDa subunit | RPP38 | NM_006414 | Hs.60339 | chr10p13 |
| 205550_s_at | brain and reproductive organ-expressed (TNFRSF1A modulator) | BRE | NM_004899 | Hs.80426 | chr2p23.2 |
| 205548_s_at | BTG family, member 3 | BTG3 | NM_006806 | Hs.77311 | chr21q21.1-q21.2 |
| 205547_s_at | transgelin | TAGLN | NM_003186 | Hs.512705 | chr11q23.2 |
| 205546_s_at | tyrosine kinase 2 | TYK2 | NM_003331 | Hs.75516 | chr19p13.2 |
| 205542_at | six transmembrane epithelial antigen of the prostate 1 | STEAP1 | NM_012449 | Hs.61635 | chr7q21 |
| 205541_s_at | G1 to S phase transition 2 /// G1 to S phase transition 2 | GSPT2 | NM_018094 | Hs.59523 | chrxp11.23-p11.21 |
| 205532_s_at | cadherin 6, type 2, K-cadherin (fetal kidney) | CDH6 | AU151483 | Hs.32963 | chr5p15.1-p14 |
| 205530_at | electron-transferring-flavoprotein dehydrogenase | ETFDH | NM_004453 | Hs.155729 | chr4q32-q35 |
| 205527_s_at | gem (nuclear organelle) associated protein 4 | GEMIN4 | NM_015487 | Hs.302421 | chr17p13 |
| 205526_s_at | katanin p60 (ATPase-containing) subunit A 1 | KATNA1 | NM_007044 | Hs.440341 | chr6q25.1 |
| 205518_s_at | cytidine monophosphate-N-acetylneuraminic acid hydroxylase (CMP-N-acetylneuraminate monooxygenase) | CMAH | NM_003570 | Hs.24697 | chr6p21.32 |
| 205517_at | GATA binding protein 4 | GATA4 | AV700724 | Hs.243987 | chr8p23.1-p22 |
| 205514_at | zinc finger protein 415 | ZNF415 | NM_018355 | Hs.21838 | chr19q13.42 |
| 205512_s_at | apoptosis-inducing factor, mitochondrion-associated, 1 | AIFM1 | NM_004208 | Hs.18720 | chrxq25-q26 |
| 205511_at | hypothetical protein FLJ10038 | FLJ10038 | NM_017976 |  | chr15q15.3 |
| 205505_at | glucosaminyl (N-acetyl) transferase 1, core 2 (beta-1,6-N-acetylglucosaminyltransferase) | GCNT1 | NM_001490 | Hs.434857 | chr9q13 |
| 205499_at | sushi-repeat-containing protein, X-linked 2 | SRPX2 | NM_014467 | Hs.306339 | chrxq21.33-q23 |
| 205497_at | zinc finger protein 175 | ZNF175 | NM_007147 | Hs.119014 | chr19q13.4 |
| 205493_s_at | dihydropyrimidinase-like 4 | DPYSL4 | NM_006426 | Hs.100058 | chr10q26 |
| 205483_s_at | ISG15 ubiquitin-like modifier | ISG15 | NM_005101 | Hs.458485 | chr1p36.33 |
| 205482_x_at | sorting nexin 15 | SNX15 | NM_013306 | Hs.80132 | chr11q12 |
| 205480_s_at | UDP-glucose pyrophosphorylase 2 | UGP2 | NM_006759 | Hs.417361 | chr2p14-p13 |
| 205472_s_at | dachshund homolog 1 (Drosophila) | DACH1 | NM_004392 | Hs.63931 | chr13q22 |
| 205457_at | chromosome 6 open reading frame 106 | C6orf106 | NM_024294 | Hs.300691 | chr6p21.31 |
| 205450_at | phosphorylase kinase, alpha 1 (muscle) | PHKA1 | NM_002637 | Hs.2393 | chrxq12-q13 |
| 205449_at | SAC3 domain containing 1 | SAC3D1 | NM_013299 | Hs.23642 | chr11q13.1 |
| 205446_s_at | activating transcription factor 2 | ATF2 | NM_001880 | Hs.80285 | chr2q32 |
| 205443_at | small nuclear RNA activating complex, polypeptide 1, 43kDa | SNAPC1 | NM_003082 | Hs.179312 | chr14q22 |
| 205442_at | microfibrillar-associated protein 3-like | MFAP3L | NM_021647 | Hs.178121 | chr4q32.3 |
| 205441_at | occludin/ELL domain containing 1 | OCEL1 | NM_024578 | Hs.422676 | chr19p13.11 |
| 205429_s_at | membrane protein, palmitoylated 6 (MAGUK p55 subfamily member 6) | MPP6 | NM_016447 | Hs.368037 | chr7p15 |
| 205428_s_at | calbindin 2, 29kDa (calretinin) | CALB2 | NM_001740 | Hs.106857 | chr16q22.2 |
| 205427_at | zinc finger protein 354A | ZNF354A | NM_005649 | Hs.321304 | chr5q35.3 |
| 205426_s_at | huntingtin interacting protein 1 | HIP1 | U79734 | Hs.444795 | chr7q11.23 |
| 205423_at | adaptor-related protein complex 1, beta 1 subunit | AP1B1 | NM_001127 | Hs.331602 | chr22q12.2 |
| 205420_at | peroxisomal biogenesis factor 7 | PEX7 | NM_000288 | Hs.79993 | chr6q21-q22.2 |
| 205413_at | metallophosphoesterase domain containing 2 | MPPED2 | NM_001584 | Hs.432000 | chr11p13 |
| 205412_at | acetyl-Coenzyme A acetyltransferase 1 (acetoacetyl Coenzyme A thiolase) | ACAT1 | NM_000019 | Hs.37 | chr11q22.3-q23.1 |
| 205406_s_at | sperm autoantigenic protein 17 | SPA17 | NM_017425 | Hs.286233 | chr11q24.2 |
| 205404_at | hydroxysteroid (11-beta) dehydrogenase 1 | HSD11B1 | NM_005525 | Hs.275215 | chr1q32-q41 |
| 205403_at | interleukin 1 receptor, type II | IL1R2 | NM_004633 | Hs.25333 | chr2q12-q22 |
| 205401_at | alkylglycerone phosphate synthase | AGPS | NM_003659 | Hs.407933 | chr2q31 |
| 205399_at | doublecortin and CaM kinase-like 1 | DCAMKL1 | NM_004734 | Hs.21355 | chr13q13 |
| 205395_s_at | MRE11 meiotic recombination 11 homolog A (S. cerevisiae) | MRE11A | NM_005590 | Hs.20555 | chr11q21 |
| 205386_s_at | Mdm2, transformed 3T3 cell double minute 2, p53 binding protein (mouse) | MDM2 | NM_002392 | Hs.369849 | chr12q14.3-q15 |
| 205381_at | leucine rich repeat containing 17 | LRRC17 | NM_005824 | Hs.288720 | chr7q22.1 |
| 205362_s_at | prefoldin subunit 4 | PFDN4 | NM_002623 | Hs.91161 | chr20q13.2 |
| 205357_s_at | angiotensin II receptor, type 1 | AGTR1 | NM_000685 | Hs.89472 | chr3q21-q25 |
| 205356_at | ubiquitin specific peptidase 13 (isopeptidase T-3) | USP13 | NM_003940 | Hs.85482 | chr3q26.2-q26.3 |
| 205355_at | acyl-Coenzyme A dehydrogenase, short/branched chain | ACADSB | NM_001609 | Hs.81934 | chr10q25-q26 |
| 205353_s_at | phosphatidylethanolamine binding protein 1 | PEBP1 | NM_002567 | Hs.433863 | chr12q24.23 |
| 205351_at | gamma-glutamyl carboxylase | GGCX | NM_000821 | Hs.77719 | chr2p12 |
| 205348_s_at | dynein, cytoplasmic 1, intermediate chain 1 | DYNC1I1 | NM_004411 | Hs.65248 | chr7q21.3-q22.1 |
| 205347_s_at | thymosin-like 8 | TMSL8 | NM_021992 | Hs.56145 | chrxq21.33-q22.3 |
| 205335_s_at | signal recognition particle 19kDa | SRP19 | NM_003135 | Hs.2943 | chr5q21-q22 |
| 205333_s_at | RCE1 homolog, prenyl protein peptidase (S. cerevisiae) | RCE1 | NM_005133 | Hs.435003 | chr11q13 |
| 205329_s_at | sorting nexin 4 | SNX4 | NM_003794 | Hs.267812 | chr3q21.2 |
| 205322_s_at | metal-regulatory transcription factor 1 | MTF1 | AW182367 | Hs.406066 | chr1p33 |
| 205321_at | eukaryotic translation initiation factor 2, subunit 3 gamma, 52kDa | EIF2S3 | NM_001415 | Hs.480368 | chrxp22.2-p22.1 |
| 205310_at | F-box protein 46 | FBXO46 | NM_012066 | Hs.128702 | chr19q13.3 |
| 205302_at | insulin-like growth factor binding protein 1 | IGFBP1 | NM_000596 | Hs.401316 | chr7p13-p12 |
| 205300_s_at | U11/U12 snRNP 35K | U1SNRNPBP | NM_022717 | Hs.528306 | chr12q24.31 |
| 205298_s_at | butyrophilin, subfamily 2, member A2 | BTN2A2 | W58757 | Hs.91813 | chr6p22.1 |
| 205294_at | BAI1-associated protein 2 | BAIAP2 | NM_017450 | Hs.128316 | chr17q25 |
| 205283_at | Fukuyama type congenital muscular dystrophy (fukutin) | FCMD | NM_006731 | Hs.55777 | chr9q31-q33 |
| 205281_s_at | phosphatidylinositol glycan anchor biosynthesis, class A (paroxysmal nocturnal hemoglobinuria) /// phosphatidylinositol glycan anchor biosynthesis, class A (paroxysmal nocturnal hemoglobinuria) | PIGA | NM_002641 | Hs.51 | chrxp22.1 |
| 205279_s_at | glycine receptor, beta | GLRB | AF094754 | Hs.32973 | chr4q31.3 |
| 205277_at | PR domain containing 2, with ZNF domain | PRDM2 | NM_012231 | Hs.413375 | chr1p36 |
| 205273_s_at | pitrilysin metallopeptidase 1 | PITRM1 | NM_014968 | Hs.528300 | chr10p15.2 |
| 205266_at | leukemia inhibitory factor (cholinergic differentiation factor) | LIF | NM_002309 | Hs.2250 | chr22q12.2 |
| 205264_at | CD3e molecule, epsilon associated protein | CD3EAP | NM_012099 | Hs.446684 | chr19q13.3 |
| 205260_s_at | acylphosphatase 1, erythrocyte (common) type | ACYP1 | NM_001107 | Hs.18573 | chr14q24.3 |
| 205258_at | inhibin, beta B (activin AB beta polypeptide) | INHBB | NM_002193 | Hs.1735 | chr2cen-q13 |
| 205255_x_at | transcription factor 7 (T-cell specific, HMG-box) | TCF7 | NM_003202 | Hs.169294 | chr5q31.1 |
| 205251_at | period homolog 2 (Drosophila) | PER2 | NM_022817 | Hs.410692 | chr2q37.3 |
| 205248_at | dopey family member 2 | DOPEY2 | NM_005128 | Hs.129781 | chr21q22.2 |
| 205246_at | peroxisome biogenesis factor 13 | PEX13 | NM_002618 | Hs.407477 | chr2p14-p16 |
| 205245_at | par-6 partitioning defective 6 homolog alpha (C. elegans) | PARD6A | NM_016948 | Hs.112933 | chr16q22.1 |
| 205241_at | SCO cytochrome oxidase deficient homolog 2 (yeast) | SCO2 | NM_005138 | Hs.410944 | chr22q13.33 |
| 205239_at | amphiregulin (schwannoma-derived growth factor) /// similar to Amphiregulin precursor (AR) (Colorectum cell-derived growth factor) (CRDGF) | AREG | NM_001657 | Hs.270833 | chr4q13-q21 |
| 205235_s_at | M-phase phosphoprotein 1 | MPHOSPH1 | NM_016195 | Hs.240 | chr10q23.31 |
| 205232_s_at | platelet-activating factor acetylhydrolase 2, 40kDa | PAFAH2 | U89386 | Hs.477083 | chr1p34.3 |
| 205231_s_at | epilepsy, progressive myoclonus type 2A, Lafora disease (laforin) | EPM2A | NM_005670 | Hs.458373 | chr6q24 |
| 205222_at | enoyl-Coenzyme A, hydratase/3-hydroxyacyl Coenzyme A dehydrogenase | EHHADH | NM_001966 | Hs.429879 | chr3q26.3-q28 |
| 205219_s_at | galactokinase 2 /// retinoblastoma binding protein 8 | GALK2 /// RBBP8 | NM_002044 | Hs.410609 | chr15q21.1 |
| 205218_at | polymerase (RNA) III (DNA directed) polypeptide F, 39 kDa | POLR3F | NM_006466 | Hs.242775 | chr20p11.23 |
| 205217_at | translocase of inner mitochondrial membrane 8 homolog A (yeast) | TIMM8A | NM_004085 | Hs.447877 | chrxq22.1 |
| 205214_at | serine/threonine kinase 17b (apoptosis-inducing) | STK17B | NM_004226 | Hs.488187 | chr2q32.3 |
| 205209_at | activin A receptor, type IB | ACVR1B | BC000254 | Hs.371974 | chr12q13 |
| 205206_at | Kallmann syndrome 1 sequence | KAL1 | NM_000216 | Hs.380850 | chrxp22.32 |
| 205205_at | v-rel reticuloendotheliosis viral oncogene homolog B, nuclear factor of kappa light polypeptide gene enhancer in B-cells 3 (avian) | RELB | NM_006509 | Hs.307905 | chr19q13.31-q13.32 |
| 205204_at | neuromedin B | NMB | NM_021077 | Hs.386470 | chr15q22-qter |
| 205203_at | phospholipase D1, phosphatidylcholine-specific | PLD1 | NM_002662 | Hs.380819 | chr3q26 |
| 205201_at | GLI-Kruppel family member GLI3 (Greig cephalopolysyndactyly syndrome) | GLI3 | NM_000168 | Hs.72916 | chr7p13 |
| 205195_at | adaptor-related protein complex 1, sigma 1 subunit | AP1S1 | NM_001283 | Hs.418710 | chr7q22.1 |
| 205193_at | v-maf musculoaponeurotic fibrosarcoma oncogene homolog F (avian) | MAFF | NM_012323 | Hs.460889 | chr22q13.1 |
| 205191_at | retinitis pigmentosa 2 (X-linked recessive) | RP2 | NM_006915 | Hs.44766 | chrxp11.4-p11.21 |
| 205190_at | plastin 1 (I isoform) | PLS1 | NM_002670 | Hs.203637 | chr3q23 |
| 205189_s_at | Fanconi anemia, complementation group C | FANCC | NM_000136 | Hs.253236 | chr9q22.3 |
| 205187_at | SMAD family member 5 | SMAD5 | AF010601 | Hs.167700 | chr5q31 |
| 205184_at | guanine nucleotide binding protein (G protein), gamma 4 | GNG4 | NM_004485 | Hs.447973 | chr1q42.3 |
| 205181_at | zinc finger protein 193 | ZNF193 | NM_006299 | Hs.100921 | chr6p21.3 |
| 205176_s_at | integrin beta 3 binding protein (beta3-endonexin) | ITGB3BP | NM_014288 | Hs.405465 | chr1p31.3 |
| 205174_s_at | glutaminyl-peptide cyclotransferase (glutaminyl cyclase) | QPCT | NM_012413 | Hs.79033 | chr2p22.2 |
| 205172_x_at | clathrin, light chain (Lcb) | CLTB | NM_007097 | Hs.380749 | chr5q35 |
| 205171_at | protein tyrosine phosphatase, non-receptor type 4 (megakaryocyte) | PTPN4 | NM_002830 | Hs.317466 | chr2q14.2 |
| 205169_at | retinoblastoma binding protein 5 | RBBP5 | NM_005057 | Hs.72984 | chr1q32 |
| 205168_at | discoidin domain receptor family, member 2 | DDR2 | NM_006182 | Hs.440905 | chr1q12-q23 |
| 205162_at | excision repair cross-complementing rodent repair deficiency, complementation group 8 | ERCC8 | NM_000082 | Hs.32967 | chr5q12.1 |
| 205158_at | ribonuclease, RNase A family, 4 | RNASE4 | NM_002937 | Hs.283749 | chr14q11.1 |
| 205145_s_at | myosin, light chain 5, regulatory | MYL5 | NM_002477 | Hs.410970 | chr4p16.3 |
| 205142_x_at | ATP-binding cassette, sub-family D (ALD), member 1 | ABCD1 | NM_000033 | Hs.159546 | chrxq28 |
| 205141_at | angiogenin, ribonuclease, RNase A family, 5 /// ribonuclease, RNase A family, 4 | ANG | NM_001145 |  | chr14q11.1-q11.2 |
| 205140_at | fucose-1-phosphate guanylyltransferase | FPGT | NM_003838 | Hs.47122 | chr1p31.1 |
| 205139_s_at | uronyl-2-sulfotransferase | UST | NM_005715 | Hs.534417 | chr6q25.1 |
| 205135_s_at | nuclear fragile X mental retardation protein interacting protein 1 | NUFIP1 | AL049842 | Hs.120247 | chr13q14 |
| 205133_s_at | heat shock 10kDa protein 1 (chaperonin 10) | HSPE1 | NM_002157 | Hs.1197 | chr2q33.1 |
| 205132_at | actin, alpha, cardiac muscle 1 | ACTC1 | NM_005159 | Hs.118127 | chr15q11-q14 |
| 205129_at | nucleophosmin/nucleoplasmin, 3 | NPM3 | NM_006993 | Hs.90691 | chr10q24.31 |
| 205128_x_at | prostaglandin-endoperoxide synthase 1 (prostaglandin G/H synthase and cyclooxygenase) | PTGS1 | NM_000962 | Hs.201978 | chr9q32-q33.3 |
| 205126_at | vaccinia related kinase 2 | VRK2 | NM_006296 | Hs.411433 | chr2p16-p15 |
| 205122_at | transmembrane protein with EGF-like and two follistatin-like domains 1 | TMEFF1 | BF439316 | Hs.303720 | chr9q31 |
| 205115_s_at | RNA binding motif protein 19 | RBM19 | NM_016196 | Hs.7482 | chr12q24.13-q24.21 |
| 205109_s_at | Rho guanine nucleotide exchange factor (GEF) 4 | ARHGEF4 | NM_015320 | Hs.6066 | chr2q22 |
| 205105_at | mannosidase, alpha, class 2A, member 1 | MAN2A1 | NM_002372 | Hs.439599 | chr5q21-q22 |
| 205100_at | glutamine-fructose-6-phosphate transaminase 2 | GFPT2 | NM_005110 | Hs.30332 | chr5q34-q35 |
| 205094_at | peroxisomal biogenesis factor 12 | PEX12 | NM_000286 | Hs.270532 | chr17q12 |
| 205091_x_at | RecQ protein-like (DNA helicase Q1-like) | RECQL | NM_002907 | Hs.192570 | chr12p12 |
| 205090_s_at | N-acetylglucosamine-1-phosphodiester alpha-N-acetylglucosaminidase | NAGPA | NM_016256 | Hs.21334 | chr16p13.3 |
| 205089_at | zinc finger protein 7 | ZNF7 | NM_003416 | Hs.2076 | chr8q24 |
| 205088_at | chromosome X open reading frame 6 | CXorf6 | NM_005491 | Hs.20136 | chrxq28 |
| 205087_at | RWD domain containing 3 | RWDD3 | NM_015485 | Hs.196585 | chr1p21.3 |
| 205084_at | B-cell receptor-associated protein 29 | BCAP29 | NM_018844 | Hs.303787 | chr7q22-q31 |
| 205077_s_at | phosphatidylinositol glycan anchor biosynthesis, class F | PIGF | NM_002643 | Hs.426142 | chr2p21-p16 |
| 205072_s_at | X-ray repair complementing defective repair in Chinese hamster cells 4 | XRCC4 | NM_022406 | Hs.150930 | chr5q13-q14 |
| 205061_s_at | exosome component 9 | EXOSC9 | NM_005033 | Hs.91728 | chr4q27 |
| 205060_at | poly (ADP-ribose) glycohydrolase /// similar to poly (ADP-ribose) glycohydrolase | PARG | NM_003631 | Hs.91390 | chr10q11.23 |
| 205052_at | AU RNA binding protein/enoyl-Coenzyme A hydratase | AUH | NM_001698 | Hs.81886 | chr9q22.31 |
| 205047_s_at | asparagine synthetase | ASNS | NM_001673 | Hs.446546 | chr7q21.3 |
| 205042_at | glucosamine (UDP-N-acetyl)-2-epimerase/N-acetylmannosamine kinase | GNE | NM_005476 | Hs.5920 | chr9p13.2 |
| 205037_at | RAB, member of RAS oncogene family-like 4 /// chromosome 21 open reading frame 21 | RABL4 | NM_006860 | Hs.415172 | chr22q13.1 |
| 205036_at | LSM6 homolog, U6 small nuclear RNA associated (S. cerevisiae) /// chromodomain helicase DNA binding protein 9 | LSM6 | NM_007080 | Hs.149675 | chr4q31.22 |
| 205032_at | integrin, alpha 2 (CD49B, alpha 2 subunit of VLA-2 receptor) | ITGA2 | NM_002203 | Hs.387725 | chr5q23-q31 |
| 205026_at | signal transducer and activator of transcription 5B | STAT5B | NM_012448 | Hs.434992 | chr17q11.2 |
| 205022_s_at | checkpoint suppressor 1 | CHES1 | NM_005197 | Hs.211773 | chr14q24.3-q32.11 |
| 205020_s_at | ADP-ribosylation factor-like 4A | ARL4A | NM_005738 | Hs.245540 | chr7p21-p15.3 |
| 205017_s_at | muscleblind-like 2 (Drosophila) | MBNL2 | AI088145 | Hs.372571 | chr13q32.1 |
| 205012_s_at | hydroxyacylglutathione hydrolase | HAGH | NM_005326 | Hs.155482 | chr16p13.3 |
| 205011_at | loss of heterozygosity, 11, chromosomal region 2, gene A | LOH11CR2A | NM_014622 | Hs.152944 | chr11q23 |
| 205004_at | NF-kappaB repressing factor | NKRF | NM_017544 | Hs.437084 | chrxq24 |
| 205003_at | dedicator of cytokinesis 4 | DOCK4 | NM_014705 | Hs.118140 | chr7q31.1 |
| 204998_s_at | activating transcription factor 5 | ATF5 | NM_012068 | Hs.9754 | chr19q13.3 |
| 204995_at | cyclin-dependent kinase 5, regulatory subunit 1 (p35) | CDK5R1 | AL567411 | Hs.93597 | chr17q11.2 |
| 204992_s_at | profilin 2 | PFN2 | NM_002628 | Hs.91747 | chr3q25.1-q25.2 |
| 204986_s_at | TAO kinase 2 | TAOK2 | NM_016151 | Hs.460576 | chr16p11.2 |
| 204985_s_at | trafficking protein particle complex 6A | TRAPPC6A | NM_024108 | Hs.386422 | chr19q13.32 |
| 204984_at | glypican 4 | GPC4 | NM_001448 | Hs.58367 | chrxq26.1 |
| 204981_at | solute carrier family 22 (organic cation transporter), member 18 | SLC22A18 | NM_002555 | Hs.50868 | chr11p15.5 |
| 204978_at | splicing factor, arginine/serine-rich 16 | SFRS16 | NM_007056 | Hs.43543 | chr19q13.3 |
| 204977_at | DEAD (Asp-Glu-Ala-Asp) box polypeptide 10 | DDX10 | NM_004398 | Hs.41706 | chr11q22-q23 |
| 204976_s_at | Alport syndrome, mental retardation, midface hypoplasia and elliptocytosis chromosomal region, gene 1 | AMMECR1 | AK023637 | Hs.366346 | chrxq22.3 |
| 204975_at | epithelial membrane protein 2 | EMP2 | NM_001424 | Hs.356835 | chr16p13.2 |
| 204971_at | cystatin A (stefin A) | CSTA | NM_005213 | Hs.412999 | chr3q21 |
| 204970_s_at | v-maf musculoaponeurotic fibrosarcoma oncogene homolog G (avian) /// similar to v-maf musculoaponeurotic fibrosarcoma oncogene family, protein G | MAFG | NM_002359 | Hs.333389 | chr17q25.3 |
| 204969_s_at | radixin | RDX | NM_002906 | Hs.263671 | chr11q23 |
| 204968_at | chromosome 6 open reading frame 47 | C6orf47 | NM_021184 | Hs.458331 | chr6p21.3 |
| 204967_at | shroom family member 2 | SHROOM2 | NM_001649 | Hs.2391 | chrxp22.3 |
| 204962_s_at | centromere protein A | CENPA | NM_001809 | Hs.1594 | chr2p24-p21 |
| 204959_at | myeloid cell nuclear differentiation antigen /// myeloid cell nuclear differentiation antigen | MNDA | NM_002432 | Hs.153837 | chr1q22 |
| 204956_at | methylthioadenosine phosphorylase | MTAP | NM_002451 | Hs.446152 | chr9p21 |
| 204955_at | sushi-repeat-containing protein, X-linked | SRPX | NM_006307 | Hs.15154 | chrxp21.1 |
| 204949_at | intercellular adhesion molecule 3 | ICAM3 | NM_002162 | Hs.353214 | chr19p13.3-p13.2 |
| 204948_s_at | follistatin | FST | NM_013409 | Hs.9914 | chr5q11.2 |
| 204946_s_at | topoisomerase (DNA) III alpha | TOP3A | NM_004618 | Hs.435124 | chr17p12-17p11.2 |
| 204945_at | protein tyrosine phosphatase, receptor type, N | PTPRN | NM_002846 | Hs.89655 | chr2q35-q36.1 |
| 204932_at | tumor necrosis factor receptor superfamily, member 11b (osteoprotegerin) | TNFRSF11B | BF433902 | Hs.81791 | chr8q24 |
| 204931_at | transcription factor 21 | TCF21 | NM_003206 | Hs.78061 | chr6pter-qter |
| 204930_s_at | BCL2/adenovirus E1B 19kDa interacting protein 1 | BNIP1 | NM_013979 | Hs.145726 | chr5q33-q34 |
| 204929_s_at | vesicle-associated membrane protein 5 (myobrevin) | VAMP5 | NM_006634 | Hs.534425 | chr2p11.2 |
| 204928_s_at | solute carrier family 10 (sodium/bile acid cotransporter family), member 3 | SLC10A3 | NM_019848 | Hs.458393 | chrxq28 |
| 204926_at | inhibin, beta A (activin A, activin AB alpha polypeptide) | INHBA | NM_002192 | Hs.28792 | chr7p15-p13 |
| 204925_at | cystinosis, nephropathic | CTNS | NM_004937 | Hs.187667 | chr17p13 |
| 204918_s_at | myeloid/lymphoid or mixed-lineage leukemia (trithorax homolog, Drosophila); translocated to, 3 | MLLT3 | NM_004529 | Hs.404 | chr9p22 |
| 204916_at | receptor (G protein-coupled) activity modifying protein 1 | RAMP1 | NM_005855 | Hs.32989 | chr2q36-q37.1 |
| 204909_at | DEAD (Asp-Glu-Ala-Asp) box polypeptide 6 | DDX6 | NM_004397 | Hs.271541 | chr11q23.3 |
| 204908_s_at | B-cell CLL/lymphoma 3 | BCL3 | NM_005178 | Hs.31210 | chr19q13.1-q13.2 |
| 204902_s_at | ATG4 autophagy related 4 homolog B (S. cerevisiae) /// similar to APG4 autophagy 4 homolog B isoform a | ATG4B | NM_013325 | Hs.283610 | chr2q37.3 |
| 204900_x_at | Sin3A-associated protein, 30kDa | SAP30 | NM_003864 | Hs.413835 | chr4q34.1 |
| 204897_at | prostaglandin E receptor 4 (subtype EP4) | PTGER4 | AA897516 | Hs.199248 | chr5p13.1 |
| 204894_s_at | amine oxidase, copper containing 3 (vascular adhesion protein 1) | AOC3 | NM_003734 | Hs.198241 | chr17q21 |
| 204893_s_at | zinc finger, FYVE domain containing 9 | ZFYVE9 | NM_004799 | Hs.162051 | chr1p32.3 |
| 204881_s_at | UDP-glucose ceramide glucosyltransferase | UGCG | NM_003358 | Hs.432605 | chr9q31 |
| 204880_at | O-6-methylguanine-DNA methyltransferase | MGMT | NM_002412 | Hs.164140 | chr10q26 |
| 204872_at | transducin-like enhancer of split 4 (E(sp1) homolog, Drosophila) | TLE4 | NM_007005 | Hs.494269 | chr9q21.31 |
| 204868_at | immature colon carcinoma transcript 1 | ICT1 | NM_001545 | Hs.407955 | chr17q25.1 |
| 204866_at | PHD finger protein 16 | PHF16 | NM_014735 | Hs.82292 | chrxp11.3 |
| 204864_s_at | interleukin 6 signal transducer (gp130, oncostatin M receptor) | IL6ST | NM_002184 | Hs.71968 | chr5q11 |
| 204859_s_at | apoptotic peptidase activating factor 1 | APAF1 | NM_013229 | Hs.373575 | chr12q23 |
| 204858_s_at | endothelial cell growth factor 1 (platelet-derived) | ECGF1 | NM_001953 | Hs.435067 | chr22q13.33 |
| 204857_at | MAD1 mitotic arrest deficient-like 1 (yeast) | MAD1L1 | NM_003550 | Hs.266820 | chr7p22 |
| 204853_at | origin recognition complex, subunit 2-like (yeast) | ORC2L | NM_006190 | Hs.444870 | chr2q33 |
| 204849_at | transcription factor-like 5 (basic helix-loop-helix) | TCFL5 | NM_006602 | Hs.30696 | chr20q13.3-qter |
| 204847_at | zinc finger and BTB domain containing 11 | ZBTB11 | NM_014415 | Hs.534463 | chr3q12.3 |
| 204841_s_at | early endosome antigen 1, 162kD | EEA1 | NM_003566 | Hs.2864 | chr12q22 |
| 204839_at | processing of precursor 5, ribonuclease P/MRP subunit (S. cerevisiae) | POP5 | NM_015918 | Hs.279913 | chr12q24.31 |
| 204835_at | polymerase (DNA directed), alpha 1 | POLA1 | NM_016937 | Hs.267289 | chrxp22.1-p21.3 |
| 204833_at | ATG12 autophagy related 12 homolog (S. cerevisiae) | ATG12 | NM_004707 | Hs.264482 | chr5q21-q22 |
| 204832_s_at | bone morphogenetic protein receptor, type IA | BMPR1A | NM_004329 | Hs.2534 | chr10q22.3 |
| 204825_at | maternal embryonic leucine zipper kinase | MELK | NM_014791 | Hs.184339 | chr9p13.2 |
| 204824_at | endonuclease G | ENDOG | NM_004435 | Hs.420106 | chr9q34.1 |
| 204812_at | ZW10, kinetochore associated, homolog (Drosophila) | ZW10 | NM_004724 | Hs.13512 | chr11q23.2 |
| 204809_at | ClpX caseinolytic peptidase X homolog (E. coli) | CLPX | NM_006660 | Hs.113823 | chr15q22.2-q22.3 |
| 204807_at | transmembrane protein 5 | TMEM5 | BF224146 | Hs.112986 | chr12q14.2 |
| 204806_x_at | major histocompatibility complex, class I, F | HLA-F | NM_018950 | Hs.411958 | chr6p21.3 |
| 204805_s_at | H1 histone family, member X | H1FX | NM_006026 | Hs.75307 | chr3q21.3 |
| 204804_at | tripartite motif-containing 21 /// gap junction protein, alpha 10, 59kDa | TRIM21 | NM_003141 | Hs.1042 | chr11p15.5 |
| 204803_s_at | Ras-related associated with diabetes | RRAD | NM_004165 | Hs.1027 | chr16q22 |
| 204800_s_at | dehydrogenase/reductase (SDR family) member 12 | DHRS12 | AW194384 | Hs.101821 | chr13q14.3 |
| 204799_at | zinc finger, BED-type containing 4 | ZBED4 | NM_014838 | Hs.13604 | chr22q13.33 |
| 204795_at | proline rich 3 | PRR3 | NM_025263 | Hs.118354 | chr6p21.33 |
| 204793_at | G protein-coupled receptor associated sorting protein 1 | GPRASP1 | NM_014710 | Hs.113082 | chrxq22.1 |
| 204791_at | nuclear receptor subfamily 2, group C, member 1 | NR2C1 | NM_003297 | Hs.108301 | chr12q22 |
| 204790_at | SMAD family member 7 | SMAD7 | NM_005904 | Hs.370849 | chr18q21.1 |
| 204788_s_at | protoporphyrinogen oxidase | PPOX | NM_000309 | Hs.376314 | chr1q22 |
| 204785_x_at | interferon (alpha, beta and omega) receptor 2 | IFNAR2 | NM_000874 | Hs.86958 | chr21q22.11 |
| 204783_at | Myeloid leukemia factor 1 | MLF1 | AI911434 | Hs.85195 | chr3q25.1 |
| 204776_at | thrombospondin 4 | THBS4 | NM_003248 | Hs.415041 | chr5q13 |
| 204774_at | ecotropic viral integration site 2A | EVI2A | NM_014210 | Hs.445237 | chr17q11.2 |
| 204772_s_at | transcription termination factor, RNA polymerase I | TTF1 | NM_007344 | Hs.54780 | chr9q34.13 |
| 204767_s_at | flap structure-specific endonuclease 1 | FEN1 | BC000323 | Hs.409065 | chr11q12 |
| 204765_at | Rho guanine nucleotide exchange factor (GEF) 5 | ARHGEF5 | NM_005435 | Hs.334 | chr7q33-q35 |
| 204761_at | USP6 N-terminal like | USP6NL | NM_014688 | Hs.278526 | chr10p13 |
| 204759_at | regulator of chromosome condensation (RCC1) and BTB (POZ) domain containing protein 2 | RCBTB2 | NM_001268 | Hs.25447 | chr13q14.3 |
| 204752_x_at | poly (ADP-ribose) polymerase family, member 2 | PARP2 | NM_005484 | Hs.409412 | chr14q11.2-q12 |
| 204751_x_at | desmocollin 2 | DSC2 | NM_004949 | Hs.95612 | chr18q12.1 |
| 204749_at | nucleosome assembly protein 1-like 3 | NAP1L3 | NM_004538 | Hs.21365 | chrxq21.3-q22 |
| 204745_x_at | metallothionein 1G | MT1G | NM_005950 | Hs.433391 | chr16q13 |
| 204742_s_at | androgen-induced proliferation inhibitor | APRIN | NM_015032 | Hs.53106 | chr13q12.3 |
| 204741_at | bicaudal D homolog 1 (Drosophila) | BICD1 | NM_001714 | Hs.412020 | chr12p11.2-p11.1 |
| 204739_at | centromere protein C 1 | CENPC1 | NM_001812 | Hs.388457 | chr4q12-q13.3 |
| 204731_at | transforming growth factor, beta receptor III (betaglycan, 300kDa) | TGFBR3 | NM_003243 | Hs.342874 | chr1p33-p32 |
| 204730_at | regulating synaptic membrane exocytosis 3 | RIMS3 | NM_014747 | Hs.434924 | chr1pter-p22.2 |
| 204725_s_at | NCK adaptor protein 1 | NCK1 | NM_006153 | Hs.54589 | chr3q21 |
| 204720_s_at | DnaJ (Hsp40) homolog, subfamily C, member 6 | DNAJC6 | AV729634 | Hs.389075 | chr1pter-q31.3 |
| 204719_at | ATP-binding cassette, sub-family A (ABC1), member 8 | ABCA8 | NM_007168 | Hs.58351 | chr17q24 |
| 204716_at | coiled-coil domain containing 6 | CCDC6 | NM_005436 | Hs.446360 | chr10q21 |
| 204715_at | pannexin 1 | PANX1 | NM_015368 | Hs.32163 | chr11q21 |
| 204706_at | inositol polyphosphate-5-phosphatase, 72 kDa | INPP5E | NM_019892 | Hs.25156 | chr9q34.3 |
| 204703_at | intraflagellar transport 88 homolog (Chlamydomonas) | IFT88 | NM_006531 | Hs.251328 | chr13q12.1 |
| 204693_at | CDC42 effector protein (Rho GTPase binding) 1 | CDC42EP1 | NM_007061 | Hs.148101 | chr22q13.1 |
| 204690_at | syntaxin 8 | STX8 | NM_004853 | Hs.380938 | chr17p12 |
| 204688_at | sarcoglycan, epsilon | SGCE | NM_003919 | Hs.409798 | chr7q21-q22 |
| 204687_at | DKFZP564O0823 protein | DKFZP564O0823 | NM_015393 | Hs.105460 | chr4q13.3-q21.3 |
| 204682_at | latent transforming growth factor beta binding protein 2 | LTBP2 | NM_000428 | Hs.105689 | chr14q24 |
| 204678_s_at | potassium channel, subfamily K, member 1 | KCNK1 | U90065 | Hs.376874 | chr1q42-q43 |
| 204676_at | chromosome 16 open reading frame 51 | C16orf51 | NM_015421 | Hs.70877 | chr16p13.13-p12.3 |
| 204672_s_at | ankyrin repeat domain 6 | ANKRD6 | NM_014942 | Hs.30991 | chr6q14.2-q16.1 |
| 204669_s_at | ring finger protein 24 | RNF24 | NM_007219 | Hs.30524 | chr20p13-p12.1 |
| 204663_at | malic enzyme 3, NADP(+)-dependent, mitochondrial /// protease, serine, 23 | ME3 | NM_006680 | Hs.376590 | chr11cen-q22.3 |
| 204651_at | nuclear respiratory factor 1 | NRF1 | AW003022 | Hs.490003 | chr7q32 |
| 204646_at | dihydropyrimidine dehydrogenase | DPYD | NM_000110 | Hs.1602 | chr1p22 |
| 204645_at | cyclin T2 | CCNT2 | NM_001241 | Hs.292754 | chr2q21.3 |
| 204643_s_at | cytosolic ovarian carcinoma antigen 1 | COVA1 | NM_006375 | Hs.155185 | chrxq25-q26.2 |
| 204639_at | adenosine deaminase | ADA | NM_000022 | Hs.407135 | chr20q12-q13.11 |
| 204634_at | NIMA (never in mitosis gene a)-related kinase 4 | NEK4 | NM_003157 | Hs.433008 | chr3p21.1 |
| 204633_s_at | ribosomal protein S6 kinase, 90kDa, polypeptide 5 | RPS6KA5 | AF074393 | Hs.510225 | chr14q31-q32.1 |
| 204624_at | ATPase, Cu++ transporting, beta polypeptide | ATP7B | NM_000053 | Hs.84999 | chr13q14.3 |
| 204619_s_at | chondroitin sulfate proteoglycan 2 (versican) | CSPG2 | BF590263 | Hs.434488 | chr5q14.3 |
| 204617_s_at | adrenocortical dysplasia homolog (mouse) | ACD | NM_022914 | Hs.78019 | chr16q22.1 |
| 204616_at | ubiquitin carboxyl-terminal esterase L3 (ubiquitin thiolesterase) | UCHL3 | NM_006002 | Hs.77917 | chr13q22.2 |
| 204615_x_at | isopentenyl-diphosphate delta isomerase 1 | IDI1 | NM_004508 | Hs.76038 | chr10p15.3 |
| 204608_at | argininosuccinate lyase | ASL | NM_000048 | Hs.442047 | chr7cen-q11.2 |
| 204605_at | cell growth regulator with ring finger domain 1 | CGRRF1 | NM_006568 | Hs.59106 | chr14q22.2 |
| 204604_at | PFTAIRE protein kinase 1 | PFTK1 | NM_012395 | Hs.57856 | chr7q21-q22 |
| 204602_at | dickkopf homolog 1 (Xenopus laevis) | DKK1 | NM_012242 | Hs.40499 | chr10q11.2 |
| 204601_at | Nedd4 binding protein 1 | N4BP1 | NM_014664 | Hs.323712 | chr16q12.1 |
| 204599_s_at | mitochondrial ribosomal protein L28 | MRPL28 | NM_006428 | Hs.279869 | chr16p13.3 |
| 204595_s_at | stanniocalcin 1 | STC1 | AI300520 | Hs.25590 | chr8p21-p11.2 |
| 204594_s_at | Smith-Magenis syndrome chromosome region, candidate 7-like | SMCR7L | NM_013298 | Hs.505742 | chr22q13 |
| 204590_x_at | vacuolar protein sorting 33 homolog A (S. cerevisiae) | VPS33A | NM_022916 | Hs.487883 | chr12q24.31 |
| 204589_at | NUAK family, SNF1-like kinase, 1 | NUAK1 | NM_014840 | Hs.200598 | chr12q23.3 |
| 204578_at | histidine acid phosphatase domain containing 2A | HISPPD2A | NM_014659 | Hs.156814 | chr15q15.3 |
| 204576_s_at | clusterin associated protein 1 | CLUAP1 | AA207013 | Hs.155995 | chr16p13.3 |
| 204575_s_at | matrix metallopeptidase 19 /// similar to Matrix metalloproteinase-19 precursor (MMP-19) (Matrix metalloproteinase RASI) (MMP-18) | MMP19 | U38321 | Hs.154057 | chr12q14 |
| 204571_x_at | protein (peptidylprolyl cis/trans isomerase) NIMA-interacting, 4 (parvulin) | PIN4 | BE797213 | Hs.118076 | chrxq13 |
| 204569_at | intestinal cell (MAK-like) kinase | ICK | NM_014920 | Hs.417022 | chr6p12.3-p11.2 |
| 204568_at | KIAA0831 | KIAA0831 | NM_014924 | Hs.414809 | chr14q22.2 |
| 204566_at | protein phosphatase 1D magnesium-dependent, delta isoform | PPM1D | NM_003620 | Hs.286073 | chr17q23.2 |
| 204565_at | thioesterase superfamily member 2 | THEM2 | NM_018473 | Hs.9676 | chr6p22.2 |
| 204563_at | selectin L (lymphocyte adhesion molecule 1) | SELL | NM_000655 | Hs.82848 | chr1q23-q25 |
| 204560_at | FK506 binding protein 5 | FKBP5 | NM_004117 | Hs.7557 | chr6p21.3-21.2 |
| 204559_s_at | LSM7 homolog, U6 small nuclear RNA associated (S. cerevisiae) | LSM7 | NM_016199 | Hs.512610 | chr19p13.3 |
| 204550_x_at | glutathione S-transferase M1 | GSTM1 | NM_000561 | Hs.301961 | chr1p13.3 |
| 204548_at | steroidogenic acute regulator | STAR | NM_000349 | Hs.440760 | chr8p11.2 |
| 204547_at | RAB40B, member RAS oncogene family | RAB40B | NM_006822 | Hs.302498 | chr17q25.3 |
| 204544_at | Hermansky-Pudlak syndrome 5 | HPS5 | NM_007216 | Hs.437599 | chr11p14 |
| 204542_at | ST6 (alpha-N-acetyl-neuraminyl-2,3-beta-galactosyl-1,3)-N-acetylgalactosaminide alpha-2,6-sialyltransferase 2 | ST6GALNAC2 | NM_006456 | Hs.288215 | chr17q25.1 |
| 204540_at | eukaryotic translation elongation factor 1 alpha 2 | EEF1A2 | NM_001958 | Hs.433839 | chr20q13.3 |
| 204538_x_at | nuclear pore complex interacting protein /// hypothetical protein LOC339047 /// similar to nuclear pore complex interacting protein /// similar to nuclear pore complex interacting protein | NPIP | NM_006985 | Hs.533789 | chr16p13-p11 |
| 204534_at | vitronectin | VTN | NM_000638 | Hs.2257 | chr17q11 |
| 204528_s_at | nucleosome assembly protein 1-like 1 | NAP1L1 | NM_004537 | Hs.419776 | chr12q21.2 |
| 204527_at | myosin VA (heavy chain 12, myoxin) | MYO5A | NM_000259 | Hs.21213 | chr15q21 |
| 204526_s_at | TBC1 domain family, member 8 (with GRAM domain) | TBC1D8 | NM_007063 | Hs.442657 | chr2q11.2 |
| 204525_at | PHD finger protein 14 | PHF14 | NM_014660 | Hs.159918 | chr7p21.3 |
| 204524_at | 3-phosphoinositide dependent protein kinase-1 | PDPK1 | NM_002613 | Hs.154729 | chr16p13.3 |
| 204523_at | zinc finger protein 140 | ZNF140 | NM_003440 | Hs.181552 | chr12q24.32-q24.33 |
| 204521_at | chromosome 12 open reading frame 24 | C12orf24 | NM_013300 | Hs.150555 | chr12q24.11 |
| 204518_s_at | peptidylprolyl isomerase C (cyclophilin C) | PPIC | NM_000943 | Hs.110364 | chr5q23.2 |
| 204515_at | hydroxy-delta-5-steroid dehydrogenase, 3 beta- and steroid delta-isomerase 1 | HSD3B1 | NM_000862 | Hs.364941 | chr1p13.1 |
| 204514_at | DPH2 homolog (S. cerevisiae) | DPH2 | NM_001384 | Hs.324830 | chr1p34 |
| 204512_at | human immunodeficiency virus type I enhancer binding protein 1 | HIVEP1 | NM_002114 | Hs.484659 | chr6p24-p22.3 |
| 204510_at | cell division cycle 7 homolog (S. cerevisiae) | CDC7 | NM_003503 | Hs.28853 | chr1p22 |
| 204497_at | adenylate cyclase 9 | ADCY9 | AB011092 | Hs.20196 | chr16p13.3 |
| 204496_at | striatin, calmodulin binding protein 3 | STRN3 | NM_014574 | Hs.183105 | chr14q13-q21 |
| 204495_s_at | chromosome 15 open reading frame 39 | C15orf39 | NM_015492 |  |  |
| 204493_at | BH3 interacting domain death agonist | BID | NM_001196 | Hs.300825 | chr22q11.1 |
| 204491_at | phosphodiesterase 4D, cAMP-specific (phosphodiesterase E3 dunce homolog, Drosophila) | PDE4D | R40917 | Hs.28482 | chr5q12 |
| 204488_at | transmembrane protein 15 | TMEM15 | NM_014908 | Hs.531563 | chr9q34.11 |
| 204487_s_at | potassium voltage-gated channel, KQT-like subfamily, member 1 | KCNQ1 | NM_000218 |  | chr11p15.5 |
| 204481_at | bromodomain and PHD finger containing, 1 | BRPF1 | NM_004634 | Hs.1004 | chr3p26-p25 |
| 204479_at | osteoclast stimulating factor 1 | OSTF1 | NM_012383 | Hs.47011 | chr9q13-q21.2 |
| 204477_at | RAB interacting factor | RABIF | U74324 | Hs.90875 | chr1q32-q41 |
| 204472_at | GTP binding protein overexpressed in skeletal muscle | GEM | NM_005261 | Hs.79022 | chr8q13-q21 |
| 204471_at | growth associated protein 43 | GAP43 | NM_002045 | Hs.79000 | chr3q13.1-q13.2 |
| 204470_at | chemokine (C-X-C motif) ligand 1 (melanoma growth stimulating activity, alpha) | CXCL1 | NM_001511 | Hs.789 | chr4q21 |
| 204467_s_at | synuclein, alpha (non A4 component of amyloid precursor) /// synuclein, alpha (non A4 component of amyloid precursor) | SNCA | NM_000345 | Hs.76930 | chr4q21 |
| 204458_at | lysophospholipase 3 (lysosomal phospholipase A2) | LYPLA3 | AL110209 | Hs.435050 | chr16q22.1 |
| 204457_s_at | growth arrest-specific 1 | GAS1 | NM_002048 | Hs.65029 | chr9q21.3-q22 |
| 204454_at | leucine zipper, down-regulated in cancer 1 | LDOC1 | NM_012317 | Hs.45231 | chrxq27 |
| 204451_at | frizzled homolog 1 (Drosophila) | FZD1 | NM_003505 | Hs.94234 | chr7q21 |
| 204449_at | phosducin-like | PDCL | NM_005388 | Hs.9302 | chr9q12-q13 |
| 204445_s_at | arachidonate 5-lipoxygenase | ALOX5 | AI361850 | Hs.89499 | chr10q11.2 |
| 204443_at | arylsulfatase A | ARSA | NM_000487 | Hs.88251 | chr22q13.33 |
| 204440_at | CD83 molecule | CD83 | NM_004233 | Hs.444310 | chr6p23 |
| 204434_at | spermatogenesis associated 2 | SPATA2 | NM_006038 | Hs.48513 | chr20q13.1-q13.2 |
| 204432_at | SRY (sex determining region Y)-box 12 | SOX12 | NM_006943 | Hs.43627 | chr20p13 |
| 204426_at | transmembrane emp24 domain trafficking protein 2 | TMED2 | NM_006815 | Hs.75914 | chr12q24.31 |
| 204422_s_at | fibroblast growth factor 2 (basic) | FGF2 | NM_002006 | Hs.422889 | chr4q26-q27 |
| 204420_at | FOS-like antigen 1 | FOSL1 | BG251266 | Hs.283565 | chr11q13 |
| 204418_x_at | glutathione S-transferase M2 (muscle) | GSTM2 | NM_000848 | Hs.279837 | chr1p13.3 |
| 204416_x_at | apolipoprotein C-I | APOC1 | NM_001645 | Hs.268571 | chr19q13.2 |
| 204415_at | interferon, alpha-inducible protein 6 | IFI6 | NM_022873 | Hs.469349 | chr1p35 |
| 204412_s_at | neurofilament, heavy polypeptide 200kDa | NEFH | NM_021076 | Hs.198760 | chr22q12.2 |
| 204408_at | APEX nuclease (apurinic/apyrimidinic endonuclease) 2 | APEX2 | NM_014481 | Hs.154149 | chrxp11.22 |
| 204407_at | transcription termination factor, RNA polymerase II | TTF2 | AF080255 | Hs.201774 | chr1p22 |
| 204404_at | solute carrier family 12 (sodium/potassium/chloride transporters), member 2 | SLC12A2 | NM_001046 | Hs.110736 | chr5q23.3 |
| 204403_x_at | KIAA0738 gene product | KIAA0738 | NM_014719 | Hs.406492 | chr7q34-q35 |
| 204402_at | rhomboid domain containing 3 | RHBDD3 | NM_012265 | Hs.106730 | chr22q12.1-q12.2 |
| 204398_s_at | echinoderm microtubule associated protein like 2 | EML2 | NM_012155 | Hs.24178 | chr19q13.32 |
| 204392_at | calcium/calmodulin-dependent protein kinase I | CAMK1 | NM_003656 | Hs.434875 | chr3p25.3 |
| 204391_x_at | tripartite motif-containing 24 | TRIM24 | NM_015905 | Hs.183858 | chr7q32-q34 |
| 204383_at | DiGeorge syndrome critical region gene 14 | DGCR14 | NM_022719 | Hs.306465 | chr22q11.21 |
| 204382_at | N-acetyltransferase 9 | NAT9 | NM_015654 | Hs.144058 | chr17q25.1 |
| 204378_at | breast carcinoma amplified sequence 1 | BCAS1 | NM_003657 | Hs.400556 | chr20q13.2-q13.3 |
| 204372_s_at | KH-type splicing regulatory protein (FUSE binding protein 2) | KHSRP | NM_003685 | Hs.91142 | chr19p13.3 |
| 204370_at | CLP1, cleavage and polyadenylation factor I subunit, homolog (S. cerevisiae) | CLP1 | NM_006831 | Hs.87465 | chr11q12 |
| 204368_at | solute carrier organic anion transporter family, member 2A1 | SLCO2A1 | NM_005630 | Hs.83974 | chr3q21 |
| 204364_s_at | receptor accessory protein 1 | REEP1 | BE535746 | Hs.7358 | chr2p11.2 |
| 204363_at | coagulation factor III (thromboplastin, tissue factor) | F3 | NM_001993 | Hs.62192 | chr1p22-p21 |
| 204362_at | src kinase associated phosphoprotein 2 | SKAP2 | NM_003930 | Hs.410745 | chr7p21-p15 |
| 204360_s_at | N-acetylglucosaminidase, alpha- (Sanfilippo disease IIIB) | NAGLU | NM_000263 | Hs.50727 | chr17q21 |
| 204358_s_at | fibronectin leucine rich transmembrane protein 2 | FLRT2 | AF169676 | Hs.48998 | chr14q24-q32 |
| 204357_s_at | LIM domain kinase 1 | LIMK1 | NM_002314 | Hs.36566 | chr7q11.23 |
| 204355_at | DEAH (Asp-Glu-Ala-His) box polypeptide 30 | DHX30 | NM_014966 | Hs.323462 | chr3p21.31 |
| 204353_s_at | POT1 protection of telomeres 1 homolog (S. pombe) | POT1 | BC002923 | Hs.31968 | chr7q31.33 |
| 204352_at | TNF receptor-associated factor 5 | TRAF5 | NM_004619 | Hs.385685 | chr1q32 |
| 204349_at | cofactor required for Sp1 transcriptional activation, subunit 9, 33kDa | CRSP9 | BC005250 | Hs.279902 | chr5q33.3 |
| 204348_s_at | adenylate kinase 3-like 1 | AK3L1 | NM_013410 | Hs.10862 | chr1p31.3 |
| 204347_at | similar to Adenylate kinase isoenzyme 4, mitochondrial (ATP-AMP transphosphorylase) /// similar to Adenylate kinase isoenzyme 4, mitochondrial (Adenylate kinase 3-like 1) (ATP-AMP transphosphorylase) | LOC645619 | AI653169 | Hs.10862 | chr1p31.3 /// chr12p11.22 |
| 204344_s_at | Sec23 homolog A (S. cerevisiae) | SEC23A | NM_006364 | Hs.272927 | chr14q21.1 |
| 204343_at | ATP-binding cassette, sub-family A (ABC1), member 3 | ABCA3 | NM_001089 | Hs.26630 | chr16p13.3 |
| 204342_at | solute carrier family 25 (mitochondrial carrier; phosphate carrier), member 24 | SLC25A24 | NM_013386 | Hs.334938 | chr1p13.3 |
| 204341_at | tripartite motif-containing 16 /// tripartite motif-containing 16-like | TRIM16 | NM_006470 | Hs.241305 | chr17p11.2 |
| 204340_at | chromosome X open reading frame 12 | CXorf12 | NM_003492 | Hs.23119 | chrxq28 |
| 204338_s_at | regulator of G-protein signalling 4 | RGS4 | NM_005613 | Hs.386726 | chr1q23.3 |
| 204336_s_at | regulator of G-protein signalling 19 | RGS19 | NM_005873 | Hs.422336 | chr20q13.3 |
| 204331_s_at | mitochondrial ribosomal protein S12 | MRPS12 | NM_021107 | Hs.411125 | chr19q13.1-q13.2 |
| 204327_s_at | zinc finger protein 202 | ZNF202 | N91520 | Hs.112556 | chr11q23.3 |
| 204326_x_at | metallothionein 1X | MT1X | NM_002450 | Hs.374950 | chr16q13 |
| 204324_s_at | golgi phosphoprotein 4 | GOLPH4 | NM_014498 | Hs.143600 | chr3q26.2 |
| 204321_at | neogenin homolog 1 (chicken) | NEO1 | NM_002499 | Hs.388613 | chr15q22.3-q23 |
| 204316_at | regulator of G-protein signalling 10 | RGS10 | W19676 | Hs.82280 | chr10q25 |
| 204312_x_at | cAMP responsive element binding protein 1 | CREB1 | AI655737 | Hs.22315 | chr2q34 |
| 204310_s_at | natriuretic peptide receptor B/guanylate cyclase B (atrionatriuretic peptide receptor B) | NPR2 | NM_003995 | Hs.78518 | chr9p21-p12 |
| 204309_at | cytochrome P450, family 11, subfamily A, polypeptide 1 /// cytochrome P450, family 11, subfamily A, polypeptide 1 | CYP11A1 | NM_000781 | Hs.76205 | chr15q23-q24 |
| 204308_s_at | KIAA0329 | KIAA0329 | NM_014844 | Hs.11711 | chr14q32.32 |
| 204306_s_at | CD151 molecule (Raph blood group) | CD151 | NM_004357 | Hs.370921 | chr11p15.5 |
| 204303_s_at | KIAA0427 | KIAA0427 | NM_014772 | Hs.64096 | chr18q21.1 |
| 204295_at | surfeit 1 | SURF1 | NM_003172 | Hs.423854 | chr9q34.2 |
| 204282_s_at | phenylalanine-tRNA synthetase 2 (mitochondrial) | FARS2 | NM_006567 | Hs.57969 | chr6p25.1 |
| 204279_at | proteasome (prosome, macropain) subunit, beta type, 9 (large multifunctional peptidase 2) | PSMB9 | NM_002800 | Hs.381081 | chr6p21.3 |
| 204275_at | small optic lobes homolog (Drosophila) | SOLH | AI796687 | Hs.416262 | chr16p13.3 |
| 204274_at | estrogen receptor binding site associated, antigen, 9 | EBAG9 | AA812215 | Hs.9222 | chr8q23 |
| 204271_s_at | endothelin receptor type B | EDNRB | M74921 | Hs.82002 | chr13q22 |
| 204268_at | S100 calcium binding protein A2 | S100A2 | NM_005978 | Hs.413843 | chr1q21 |
| 204267_x_at | protein kinase, membrane associated tyrosine/threonine 1 | PKMYT1 | NM_004203 | Hs.534409 | chr16p13.3 |
| 204264_at | carnitine palmitoyltransferase II | CPT2 | NM_000098 | Hs.525391 | chr1p32 |
| 204262_s_at | presenilin 2 (Alzheimer disease 4) | PSEN2 | NM_000447 | Hs.25363 | chr1q31-q42 |
| 204260_at | chromogranin B (secretogranin 1) | CHGB | NM_001819 | Hs.2281 | chr20pter-p12 |
| 204252_at | cyclin-dependent kinase 2 /// beta-carotene dioxygenase 2 | CDK2 | M68520 | Hs.19192 | chr12q13 |
| 204251_s_at | centrosomal protein 164kDa | CEP164 | NM_014956 | Hs.504009 | chr11q23.3 |
| 204246_s_at | dynactin 3 (p22) | DCTN3 | NM_007234 | Hs.511768 | chr9p13 |
| 204244_s_at | DBF4 homolog (S. cerevisiae) | DBF4 | NM_006716 | Hs.152759 | chr7q21.3 |
| 204243_at | rearranged L-myc fusion | RLF | NM_012421 | Hs.13321 | chr1p32 |
| 204241_at | acyl-Coenzyme A oxidase 3, pristanoyl | ACOX3 | BF055171 | Hs.12773 | chr4p15.3 |
| 204237_at | GULP, engulfment adaptor PTB domain containing 1 | GULP1 | NM_016315 | Hs.107056 | chr2q32.3-q33 |
| 204234_s_at | zinc finger protein 195 | ZNF195 | AI476267 | Hs.104382 | chr11p15.5 |
| 204232_at | Fc fragment of IgE, high affinity I, receptor for; gamma polypeptide | FCER1G | NM_004106 | Hs.433300 | chr1q23 |
| 204228_at | peptidylprolyl isomerase H (cyclophilin H) | PPIH | NM_006347 | Hs.9880 | chr1p34.1 |
| 204227_s_at | thymidine kinase 2, mitochondrial | TK2 | NM_004614 | Hs.274701 | chr16q22-q23.1 |
| 204225_at | histone deacetylase 4 | HDAC4 | NM_006037 | Hs.222874 | chr2q37.2 |
| 204224_s_at | GTP cyclohydrolase 1 (dopa-responsive dystonia) | GCH1 | NM_000161 | Hs.86724 | chr14q22.1-q22.2 |
| 204219_s_at | proteasome (prosome, macropain) 26S subunit, ATPase, 1 | PSMC1 | NM_002802 | Hs.356654 | chr14q32.11 |
| 204218_at | chromosome 11 open reading frame 51 | C11orf51 | NM_014042 | Hs.38044 | chr11q13.4 |
| 204216_s_at | zinc finger CCCH-type containing 14 | ZC3H14 | NM_024824 | Hs.323443 | chr14q31.3 |
| 204215_at | chromosome 7 open reading frame 23 | C7orf23 | NM_024315 | Hs.322404 | chr7q21.1-q21.2 |
| 204214_s_at | RAB32, member RAS oncogene family | RAB32 | NM_006834 | Hs.493406 | chr6q24.3 |
| 204211_x_at | eukaryotic translation initiation factor 2-alpha kinase 2 | EIF2AK2 | NM_002759 | Hs.439523 | chr2p22-p21 |
| 204210_s_at | phosphate cytidylyltransferase 1, choline, alpha | PCYT1A | NM_005017 | Hs.435767 | chr3q29 |
| 204206_at | MAX binding protein | MNT | NM_020310 | Hs.253552 | chr17p13.3 |
| 204204_at | solute carrier family 31 (copper transporters), member 2 | SLC31A2 | NM_001860 | Hs.24030 | chr9q31-q32 |
| 204202_at | IQ motif containing E | IQCE | NM_017604 | Hs.446063 | chr7p22.3 |
| 204201_s_at | protein tyrosine phosphatase, non-receptor type 13 (APO-1/CD95 (Fas)-associated phosphatase) | PTPN13 | NM_006264 | Hs.387553 | chr4q21.3 |
| 204195_s_at | PBX/knotted 1 homeobox 1 | PKNOX1 | AL570914 | Hs.431043 | chr21q22.3 |
| 204194_at | BTB and CNC homology 1, basic leucine zipper transcription factor 1 | BACH1 | NM_001186 | Hs.154276 | chr21q22.11 |
| 204190_at | ubiquitin specific peptidase like 1 | USPL1 | NM_005800 | Hs.291431 | chr13q12-q14 |
| 204182_s_at | zinc finger and BTB domain containing 43 | ZBTB43 | NM_014007 | Hs.355581 | chr9p24.1-q22.33 |
| 204175_at | zinc finger protein 593 | ZNF593 | NM_015871 | Hs.102419 | chr1p36.11 |
| 204174_at | arachidonate 5-lipoxygenase-activating protein | ALOX5AP | NM_001629 | Hs.100194 | chr13q12 |
| 204173_at | myosin, light chain 6B, alkali, smooth muscle and non-muscle | MYL6B | NM_002475 | Hs.90318 | chr12q13.13 |
| 204172_at | coproporphyrinogen oxidase | CPOX | NM_000097 | Hs.89866 | chr3q12 |
| 204170_s_at | CDC28 protein kinase regulatory subunit 2 | CKS2 | NM_001827 | Hs.83758 | chr9q22 |
| 204168_at | microsomal glutathione S-transferase 2 | MGST2 | NM_002413 | Hs.81874 | chr4q28.3 |
| 204165_at | WAS protein family, member 1 | WASF1 | NM_003931 | Hs.75850 | chr6q21-q22 |
| 204161_s_at | ectonucleotide pyrophosphatase/phosphodiesterase 4 (putative function) | ENPP4 | NM_014936 | Hs.54037 | chr6p21.1 |
| 204151_x_at | aldo-keto reductase family 1, member C1 (dihydrodiol dehydrogenase 1; 20-alpha (3-alpha)-hydroxysteroid dehydrogenase) | AKR1C1 | NM_001353 | Hs.295131 | chr10p15-p14 |
| 204148_s_at | zona pellucida glycoprotein 3 (sperm receptor) /// POM (POM121 homolog, rat) and ZP3 fusion /// Meis1, myeloid ecotropic viral integration site 1 homolog 3 (mouse) | ZP3 | NM_012230 | Hs.512724 | chr7q11.23 |
| 204144_s_at | phosphatidylinositol glycan anchor biosynthesis, class Q | PIGQ | NM_004204 | Hs.18079 | chr16p13.3 |
| 204143_s_at | enolase superfamily member 1 | ENOSF1 | NM_017512 | Hs.475848 | chr18p11.32 |
| 204137_at | G protein-coupled receptor 137B | GPR137B | NM_003272 | Hs.15791 | chr1q42-q43 |
| 204129_at | B-cell CLL/lymphoma 9 | BCL9 | NM_004326 | Hs.415209 | chr1q21 |
| 204128_s_at | replication factor C (activator 1) 3, 38kDa | RFC3 | NM_002915 | Hs.115474 | chr13q12.3-q13 |
| 204125_at | NADH dehydrogenase (ubiquinone) 1 alpha subcomplex, assembly factor 1 | NDUFAF1 | NM_016013 | Hs.106529 | chr15q11.2-q21.3 |
| 204122_at | TYRO protein tyrosine kinase binding protein | TYROBP | NM_003332 | Hs.9963 | chr19q13.1 |
| 204121_at | growth arrest and DNA-damage-inducible, gamma | GADD45G | NM_006705 | Hs.9701 | chr9q22.1-q22.2 |
| 204120_s_at | adenosine kinase | ADK | NM_001123 | Hs.355533 | chr10q22 |
| 204115_at | guanine nucleotide binding protein (G protein), gamma 11 | GNG11 | NM_004126 | Hs.83381 | chr7q31-q32 |
| 204114_at | nidogen 2 (osteonidogen) | NID2 | NM_007361 | Hs.147697 | chr14q21-q22 |
| 204108_at | nuclear transcription factor Y, alpha | NFYA | AL031778 | Hs.10441 | chr6p21.3 |
| 204106_at | testis-specific kinase 1 | TESK1 | NM_006285 | Hs.79358 | chr9p13 |
| 204105_s_at | neuronal cell adhesion molecule | NRCAM | NM_005010 | Hs.7912 | chr7q31.1-q31.2 |
| 204099_at | SWI/SNF related, matrix associated, actin dependent regulator of chromatin, subfamily d, member 3 | SMARCD3 | NM_003078 | Hs.444445 | chr7q35-q36 |
| 204098_at | RNA binding motif protein, X-linked 2 | RBMX2 | NM_016024 | Hs.61184 | chrxq25 |
| 204093_at | cyclin H | CCNH | NM_001239 | Hs.514 | chr5q13.3-q14 |
| 204089_x_at | mitogen-activated protein kinase kinase kinase 4 | MAP3K4 | NM_006724 | Hs.390428 | chr6q26 |
| 204088_at | purinergic receptor P2X, ligand-gated ion channel, 4 | P2RX4 | NM_002560 | Hs.321709 | chr12q24.32 |
| 204086_at | preferentially expressed antigen in melanoma | PRAME | NM_006115 | Hs.30743 | chr22q11.22 |
| 204085_s_at | ceroid-lipofuscinosis, neuronal 5 | CLN5 | NM_006493 | Hs.30213 | chr13q21.1-q32 |
| 204082_at | pre-B-cell leukemia transcription factor 3 | PBX3 | NM_006195 | Hs.294101 | chr9q33-q34 |
| 204079_at | tyrosylprotein sulfotransferase 2 | TPST2 | NM_003595 | Hs.438649 | chr22q12.1 |
| 204078_at | synaptonemal complex protein SC65 | SC65 | NM_006455 | Hs.446459 | chr17q21.2 |
| 204077_x_at | ectonucleoside triphosphate diphosphohydrolase 4 | ENTPD4 | NM_004901 | Hs.122301 | chr8p21.3 |
| 204074_s_at | KIAA0562 | KIAA0562 | AI936976 | Hs.201972 | chr1p36.32 |
| 204072_s_at | furry homolog (Drosophila) | FRY | NM_023037 | Hs.390874 | chr13q13.1 |
| 204071_s_at | topoisomerase I binding, arginine/serine-rich | TOPORS | NM_005802 | Hs.533976 | chr9p21 |
| 204068_at | serine/threonine kinase 3 (STE20 homolog, yeast) | STK3 | NM_006281 | Hs.166684 | chr8q22.2 |
| 204066_s_at | centaurin, gamma 2 | CENTG2 | NM_014914 | Hs.154116 | chr2p24.3-p24.1 |
| 204063_s_at | unc-51-like kinase 2 (C. elegans) | ULK2 | NM_014683 | Hs.168762 | chr17p11.2 |
| 204061_at | protein kinase, X-linked | PRKX | NM_005044 | Hs.147996 | chrxp22.3 |
| 204058_at | malic enzyme 1, NADP(+)-dependent, cytosolic | ME1 | AL049699 | Hs.14732 | chr6q12 |
| 204056_s_at | mevalonate kinase (mevalonic aciduria) | MVK | NM_000431 | Hs.130607 | chr12q24 |
| 204054_at | phosphatase and tensin homolog (mutated in multiple advanced cancers 1) | PTEN | NM_000314 | Hs.253309 | chr10q23.3 |
| 204051_s_at | secreted frizzled-related protein 4 | SFRP4 | AW089415 | Hs.105700 | chr7p14.1 |
| 204048_s_at | phosphatase and actin regulator 2 | PHACTR2 | AA551142 | Hs.102471 | chr6q24.2 |
| 204045_at | transcription elongation factor A (SII)-like 1 | TCEAL1 | NM_004780 | Hs.95243 | chrxq22.1 |
| 204042_at | WAS protein family, member 3 | WASF3 | AB020707 | Hs.82318 | chr13q12 |
| 204041_at | monoamine oxidase B | MAOB | NM_000898 | Hs.82163 | chrxp11.23 |
| 204040_at | ring finger protein 144 | RNF144 | NM_014746 | Hs.78894 | chr2p25.1 |
| 204035_at | secretogranin II (chromogranin C) | SCG2 | NM_003469 | Hs.436577 | chr2q35-q36 |
| 204034_at | ethylmalonic encephalopathy 1 | ETHE1 | NM_014297 | Hs.7486 | chr19q13.31 |
| 204033_at | thyroid hormone receptor interactor 13 | TRIP13 | NM_004237 | Hs.436187 | chr5p15.33 |
| 204032_at | breast cancer anti-estrogen resistance 3 | BCAR3 | NM_003567 | Hs.201993 | chr1p22.1 |
| 204030_s_at | schwannomin interacting protein 1 | SCHIP1 | NM_014575 | Hs.61490 | chr3q25.32-q25.33 |
| 204028_s_at | RAB GTPase activating protein 1 | RABGAP1 | NM_012197 | Hs.55099 | chr9q33.2-q33.3 |
| 204027_s_at | methyltransferase like 1 | METTL1 | NM_005371 | Hs.42957 | chr12q13 |
| 204026_s_at | ZW10 interactor | ZWINT | NM_007057 | Hs.42650 | chr10q21-q22 |
| 204024_at | oxidative stress induced growth inhibitor family member 2 | OSGIN2 | NM_004337 | Hs.436445 | chr8q21 |
| 204023_at | replication factor C (activator 1) 4, 37kDa | RFC4 | NM_002916 | Hs.35120 | chr3q27 |
| 204022_at | WW domain containing E3 ubiquitin protein ligase 2 | WWP2 | AI668780 | Hs.315485 | chr16q22.1 |
| 204020_at | purine-rich element binding protein A | PURA | BF739943 | Hs.29117 | chr5q31 |
| 204019_s_at | SH3 domain containing, Ysc84-like 1 (S. cerevisiae) | SH3YL1 | NM_015677 | Hs.147365 | chr2p25.3 |
| 204012_s_at | leucine carboxyl methyltransferase 2 | LCMT2 | AL529189 | Hs.200596 | chr15q15.3 |
| 204011_at | sprouty homolog 2 (Drosophila) | SPRY2 | NM_005842 | Hs.18676 | chr13q31.1 |
| 204009_s_at | v-Ki-ras2 Kirsten rat sarcoma viral oncogene homolog | KRAS | W80678 | Hs.412107 | chr12p12.1 |
| 204008_at | dynein, axonemal, light chain 4 | DNAL4 | NM_005740 | Hs.258203 | chr22q13.1 |
| 204007_at | Fc fragment of IgG, low affinity IIIb, receptor (CD16b) | FCGR3B | J04162 |  | chr1q23 |
| 204003_s_at | nucleoporin like 2 | NUPL2 | NM_007342 | Hs.408241 | chr7p15 |
| 203990_s_at | ubiquitously transcribed tetratricopeptide repeat, X chromosome | UTX | AI140752 | Hs.201790 | chrxp11.2 |
| 203989_x_at | coagulation factor II (thrombin) receptor | F2R | NM_001992 | Hs.128087 | chr5q13 |
| 203987_at | frizzled homolog 6 (Drosophila) | FZD6 | NM_003506 | Hs.276005 | chr8q22.3-q23.1 |
| 203986_at | genethonin 1 | GENX-3414 | NM_003943 | Hs.109590 | chr4q24-q25 |
| 203985_at | zinc finger protein 212 | ZNF212 | NM_012256 | Hs.108139 | chr7q36.1 |
| 203983_at | translin-associated factor X | TSNAX | NM_005999 | Hs.96247 | chr1q42.1 |
| 203981_s_at | ATP-binding cassette, sub-family D (ALD), member 4 | ABCD4 | AL574660 | Hs.94395 | chr14q24.3 |
| 203978_at | nucleotide binding protein 1 (MinD homolog, E. coli) | NUBP1 | NM_002484 | Hs.81469 | chr16p13.13 |
| 203975_s_at | chromatin assembly factor 1, subunit A (p150) | CHAF1A | BF000239 | Hs.408036 | chr19p13.3 |
| 203974_at | haloacid dehalogenase-like hydrolase domain containing 1A | HDHD1A | NM_012080 | Hs.432580 | chrxp22.32 |
| 203970_s_at | peroxisomal biogenesis factor 3 | PEX3 | NM_003630 | Hs.7277 | chr6q23-q24 |
| 203964_at | N-myc (and STAT) interactor | NMI | NM_004688 | Hs.54483 | chr2p24.3-q21.3 |
| 203960_s_at | chromosome 1 open reading frame 41 /// interleukin 17 receptor B | C1orf41 | NM_016126 | Hs.439171 | chr1p32.1-p33 |
| 203958_s_at | zinc finger and BTB domain containing 40 | ZBTB40 | AI557467 | Hs.528723 | chr1pter-q31.3 |
| 203957_at | E2F transcription factor 6 | E2F6 | NM_001952 | Hs.135465 | chr2p25.1 |
| 203955_at | KIAA0649 | KIAA0649 | NM_014811 | Hs.26163 | chr9q34.3 |
| 203954_x_at | claudin 3 | CLDN3 | NM_001306 | Hs.25640 | chr7q11.23 |
| 203952_at | activating transcription factor 6 | ATF6 | NM_007348 | Hs.433046 | chr1q22-q23 |
| 203950_s_at | chloride channel 6 | CLCN6 | NM_001286 | Hs.371458 | chr1p36 |
| 203946_s_at | arginase, type II | ARG2 | U75667 | Hs.534306 | chr14q24.1-q24.3 |
| 203943_at | kinesin family member 3B | KIF3B | NM_004798 | Hs.301206 | chr20q11.21 |
| 203941_at | integrator complex subunit 9 | RC74 | NM_018250 | Hs.15562 | chr8p21.1 |
| 203938_s_at | TATA box binding protein (TBP)-associated factor, RNA polymerase I, C, 110kDa | TAF1C | NM_005679 | Hs.153022 | chr16q24 |
| 203936_s_at | matrix metallopeptidase 9 (gelatinase B, 92kDa gelatinase, 92kDa type IV collagenase) | MMP9 | NM_004994 | Hs.151738 | chr20q11.2-q13.1 |
| 203935_at | activin A receptor, type I | ACVR1 | NM_001105 | Hs.150402 | chr2q23-q24 |
| 203932_at | major histocompatibility complex, class II, DM beta /// major histocompatibility complex, class II, DM beta | HLA-DMB | NM_002118 | Hs.1162 | chr6p21.3 |
| 203931_s_at | mitochondrial ribosomal protein L12 | MRPL12 | NM_002949 | Hs.109059 | chr17q25 |
| 203926_x_at | ATP synthase, H+ transporting, mitochondrial F1 complex, delta subunit | ATP5D | NM_001687 | Hs.501304 | chr19p13.3 |
| 203921_at | carbohydrate (N-acetylglucosamine-6-O) sulfotransferase 2 | CHST2 | NM_004267 | Hs.8786 | chr3q24 |
| 203920_at | nuclear receptor subfamily 1, group H, member 3 | NR1H3 | NM_005693 | Hs.438863 | chr11p11.2 |
| 203919_at | transcription elongation factor A (SII), 2 | TCEA2 | NM_003195 | Hs.224397 | chr20q13.33 |
| 203910_at | Rho GTPase activating protein 29 | ARHGAP29 | NM_004815 | Hs.430919 | chr1p22.1 |
| 203909_at | solute carrier family 9 (sodium/hydrogen exchanger), member 6 | SLC9A6 | NM_006359 | Hs.62185 | chrxq26.3 |
| 203905_at | poly(A)-specific ribonuclease (deadenylation nuclease) | PARN | NM_002582 | Hs.43445 | chr16p13 |
| 203899_s_at | calcitonin gene-related peptide-receptor component protein | RCP9 | NM_014478 | Hs.300684 | chr7q11.21 |
| 203897_at | LYR motif containing 1 | LYRM1 | BE963444 | Hs.28607 | chr16p11.2 |
| 203896_s_at | phospholipase C, beta 4 | PLCB4 | NM_000933 | Hs.151408 | chr20p12 |
| 203894_at | tubulin, gamma 2 | TUBG2 | NM_016437 | Hs.279669 | chr17q21 |
| 203890_s_at | death-associated protein kinase 3 | DAPK3 | BF686824 | Hs.153908 | chr19p13.3 |
| 203889_at | secretogranin V (7B2 protein) | SCG5 | NM_003020 | Hs.2265 | chr15q13-q14 |
| 203887_s_at | thrombomodulin | THBD | NM_000361 | Hs.2030 | chr20p12-cen |
| 203883_s_at | RAB11 family interacting protein 2 (class I) | RAB11FIP2 | BG249608 | Hs.173656 | chr10q26.11 |
| 203882_at | interferon-stimulated transcription factor 3, gamma 48kDa | ISGF3G | NM_006084 | Hs.1706 | chr14q11.2 |
| 203880_at | COX17 cytochrome c oxidase assembly homolog (S. cerevisiae) | COX17 | NM_005694 | Hs.16297 | chr3q13.33 |
| 203879_at | phosphoinositide-3-kinase, catalytic, delta polypeptide /// phosphoinositide-3-kinase, catalytic, delta polypeptide | PIK3CD | U86453 | Hs.426967 | chr1p36.2 |
| 203878_s_at | matrix metallopeptidase 11 (stromelysin 3) | MMP11 | NM_005940 | Hs.143751 | chr22q11.23 |
| 203874_s_at | SWI/SNF related, matrix associated, actin dependent regulator of chromatin, subfamily a, member 1 | SMARCA1 | NM_003069 | Hs.152292 | chrxq25 |
| 203871_at | SUMO1/sentrin/SMT3 specific peptidase 3 | SENP3 | NM_015670 | Hs.255022 | chr17p13 |
| 203868_s_at | vascular cell adhesion molecule 1 | VCAM1 | NM_001078 | Hs.109225 | chr1p32-p31 |
| 203860_at | propionyl Coenzyme A carboxylase, alpha polypeptide | PCCA | NM_000282 | Hs.80741 | chr13q32 |
| 203858_s_at | COX10 homolog, cytochrome c oxidase assembly protein, heme A: farnesyltransferase (yeast) | COX10 | NM_001303 | Hs.77513 | chr17p12-17p11.2 |
| 203857_s_at | protein disulfide isomerase family A, member 5 | PDIA5 | NM_006810 | Hs.76901 | chr3q21.1 |
| 203856_at | vaccinia related kinase 1 | VRK1 | NM_003384 | Hs.422662 | chr14q32 |
| 203855_at | WD repeat domain 47 | WDR47 | NM_014969 | Hs.485081 | chr1p13.3 |
| 203853_s_at | GRB2-associated binding protein 2 | GAB2 | NM_012296 | Hs.30687 | chr11q14.1 |
| 203852_s_at | survival of motor neuron 1, telomeric /// survival of motor neuron 2, centromeric | SMN1 | NM_000344 | Hs.288986 | chr5q13 |
| 203848_at | A kinase (PRKA) anchor protein 8 | AKAP8 | AL050160 | Hs.25059 | chr19p13.1-q12 |
| 203846_at | tripartite motif-containing 32 | TRIM32 | BC003154 | Hs.236218 | chr9q33.1 |
| 203845_at | p300/CBP-associated factor | PCAF | AV727449 | Hs.203475 | chr3p24 |
| 203843_at | ribosomal protein S6 kinase, 90kDa, polypeptide 3 | RPS6KA3 | AA906056 | Hs.188361 | chrxp22.2-p22.1 |
| 203836_s_at | mitogen-activated protein kinase kinase kinase 5 | MAP3K5 | D84476 | Hs.151988 | chr6q22.33 |
| 203834_s_at | trans-golgi network protein 2 | TGOLN2 | NM_006464 | Hs.14894 | chr2p11.2 |
| 203832_at | small nuclear ribonucleoprotein polypeptide F | SNRPF | NM_003095 | Hs.105465 | chr12q23.1 |
| 203831_at | R3H domain containing 2 | R3HDM2 | NM_014925 | Hs.443673 | chr12q13.3 |
| 203830_at | chromosome 17 open reading frame 75 | C17orf75 | NM_022344 | Hs.9800 | chr17q11.2 |
| 203829_at | elongation protein 4 homolog (S. cerevisiae) | ELP4 | NM_019040 | Hs.134725 | chr11p13 |
| 203825_at | bromodomain containing 3 | BRD3 | NM_007371 | Hs.86896 | chr9q34 |
| 203823_at | regulator of G-protein signalling 3 | RGS3 | NM_021106 | Hs.82294 | chr9q32 |
| 203818_s_at | splicing factor 3a, subunit 3, 60kDa | SF3A3 | NM_006802 | Hs.77897 | chr1p34.3 |
| 203817_at | guanylate cyclase 1, soluble, beta 3 | GUCY1B3 | W93728 | Hs.77890 | chr4q31.3-q33 |
| 203816_at | deoxyguanosine kinase | DGUOK | NM_001929 | Hs.409430 | chr2p13 |
| 203814_s_at | NAD(P)H dehydrogenase, quinone 2 | NQO2 | NM_000904 | Hs.441039 | chr6pter-q12 |
| 203811_s_at | DnaJ (Hsp40) homolog, subfamily B, member 4 | DNAJB4 | NM_007034 | Hs.380282 | chr1p31.1 |
| 203803_at | prenylcysteine oxidase 1 | PCYOX1 | N45309 | Hs.356544 | chr2p13.3 |
| 203801_at | mitochondrial ribosomal protein S14 | MRPS14 | AA013164 | Hs.247324 | chr1q23-1q25 |
| 203799_at | CD302 molecule | CD302 | NM_014880 | Hs.2441 | chr2q24.2 |
| 203796_s_at | B-cell CLL/lymphoma 7A | BCL7A | AI950380 | Hs.131121 | chr12q24.13 |
| 203794_at | CDC42 binding protein kinase alpha (DMPK-like) | CDC42BPA | NM_014826 | Hs.18586 | chr1q42.11 |
| 203791_at | Dmx-like 1 | DMXL1 | NM_005509 | Hs.181042 | chr5q22 |
| 203790_s_at | heat-responsive protein 12 | HRSP12 | N54448 | Hs.18426 | chr8q22 |
| 203788_s_at | sema domain, immunoglobulin domain (Ig), short basic domain, secreted, (semaphorin) 3C | SEMA3C | AI962897 | Hs.171921 | chr7q21-q31 |
| 203786_s_at | tumor protein D52-like 1 | TPD52L1 | NM_003287 | Hs.16611 | chr6q22-q23 |
| 203785_s_at | DEAD (Asp-Glu-Ala-Asp) box polypeptide 28 | DDX28 | NM_018380 | Hs.458313 | chr16q22.1 |
| 203781_at | mitochondrial ribosomal protein L33 | MRPL33 | NM_004891 | Hs.14454 | chr2p21 |
| 203778_at | mannosidase, beta A, lysosomal | MANBA | NM_005908 | Hs.398082 | chr4q22-q25 |
| 203777_s_at | ribosomal protein S6 kinase, 70kDa, polypeptide 2 | RPS6KB2 | NM_003952 | Hs.534368 | chr11q13.2 |
| 203776_at | G patch domain and KOW motifs | GPKOW | NM_015698 | Hs.503666 | chrxp11.23 |
| 203774_at | 5-methyltetrahydrofolate-homocysteine methyltransferase | MTR | NM_000254 | Hs.82283 | chr1q43 |
| 203771_s_at | biliverdin reductase A | BLVRA | AA740186 | Hs.435726 | chr7p14-cen |
| 203769_s_at | steroid sulfatase (microsomal), arylsulfatase C, isozyme S | STS | NM_000351 | Hs.79876 | chrxp22.32 |
| 203765_at | grancalcin, EF-hand calcium binding protein /// grancalcin, EF-hand calcium binding protein | GCA | NM_012198 | Hs.377894 | chr2q24.2 |
| 203758_at | cathepsin O | CTSO | AV729484 | Hs.75262 | chr4q31-q32 |
| 203752_s_at | jun D proto-oncogene | JUND | NM_005354 | Hs.2780 | chr19p13.2 |
| 203746_s_at | holocytochrome c synthase (cytochrome c heme-lyase) | HCCS | NM_005333 | Hs.211571 | chrxp22.3 |
| 203744_at | high-mobility group box 3 | HMGB3 | NM_005342 | Hs.19114 | chrxq28 |
| 203742_s_at | thymine-DNA glycosylase /// similar to G/T mismatch-specific thymine DNA glycosylase /// similar to G/T mismatch-specific thymine DNA glycosylase | TDG | BF674842 | Hs.506556 | chr12q24.1 |
| 203741_s_at | adenylate cyclase 7 | ADCY7 | NM_001114 | Hs.172199 | chr16q12-q13 |
| 203739_at | zinc finger protein 217 | ZNF217 | NM_006526 | Hs.155040 | chr20q13.2 |
| 203738_at | chromosome 5 open reading frame 22 | C5orf22 | AI421192 | Hs.151046 | chr5p13.3 |
| 203737_s_at | peroxisome proliferator-activated receptor gamma, coactivator-related 1 | PPRC1 | NM_015062 | Hs.146957 | chr10q24.32 |
| 203736_s_at | PTPRF interacting protein, binding protein 1 (liprin beta 1) | PPFIBP1 | NM_003622 | Hs.389023 | chr12p11.23-p11.22 |
| 203734_at | forkhead box J2 | FOXJ2 | NM_018416 | Hs.120844 | chr12p13.31 |
| 203733_at | dexamethasone-induced transcript | DEXI | NM_014015 | Hs.157925 | chr16p13.13 |
| 203732_at | thyroid hormone receptor interactor 4 | TRIP4 | NM_016213 | Hs.116784 | chr15q22.31 |
| 203730_s_at | zinc finger protein 95 homolog (mouse) | ZFP95 | BF196931 | Hs.110839 | chr7q22 |
| 203729_at | epithelial membrane protein 3 | EMP3 | NM_001425 | Hs.9999 | chr19q13.3 |
| 203726_s_at | laminin, alpha 3 | LAMA3 | NM_000227 | Hs.83450 | chr18q11.2 |
| 203725_at | growth arrest and DNA-damage-inducible, alpha | GADD45A | NM_001924 | Hs.80409 | chr1p31.2-p31.1 |
| 203721_s_at | UTP18, small subunit (SSU) processome component, homolog (yeast) | UTP18 | NM_016001 | Hs.441503 | chr17q21.33 |
| 203720_s_at | excision repair cross-complementing rodent repair deficiency, complementation group 1 (includes overlapping antisense sequence) | ERCC1 | NM_001983 | Hs.435981 | chr19q13.2-q13.3 |
| 203718_at | patatin-like phospholipase domain containing 6 | PNPLA6 | NM_006702 | Hs.511760 | chr19p13.3-p13.2 |
| 203715_at | tubulin folding cofactor E | TBCE | NM_003193 | Hs.445778 | chr1q42.3 |
| 203712_at | KIAA0020 | KIAA0020 | NM_014878 | Hs.443866 | chr9p24.2 |
| 203707_at | zinc finger protein 263 | ZNF263 | NM_005741 | Hs.124553 | chr16p13.3 |
| 203705_s_at | frizzled homolog 7 (Drosophila) | FZD7 | AI333651 | Hs.173859 | chr2q33 |
| 203694_s_at | DEAH (Asp-Glu-Ala-His) box polypeptide 16 | DHX16 | NM_003587 | Hs.12797 | chr6p21.3 |
| 203693_s_at | E2F transcription factor 3 | E2F3 | NM_001949 | Hs.1189 | chr6p22 |
| 203690_at | tubulin, gamma complex associated protein 3 | TUBGCP3 | NM_006322 | Hs.9884 | chr13q34 |
| 203689_s_at | fragile X mental retardation 1 | FMR1 | AI743037 | Hs.89764 | chrxq27.3 |
| 203688_at | polycystic kidney disease 2 (autosomal dominant) | PKD2 | NM_000297 | Hs.458291 | chr4q21-q23 |
| 203686_at | N-methylpurine-DNA glycosylase | MPG | NM_002434 | Hs.79396 | chr16p13.3 |
| 203685_at | B-cell CLL/lymphoma 2 | BCL2 | NM_000633 | Hs.79241 | chr18q21.3 |
| 203683_s_at | vascular endothelial growth factor B | VEGFB | NM_003377 | Hs.78781 | chr11q13 |
| 203682_s_at | isovaleryl Coenzyme A dehydrogenase | IVD | NM_002225 | Hs.410396 | chr15q14-q15 |
| 203680_at | protein kinase, cAMP-dependent, regulatory, type II, beta | PRKAR2B | NM_002736 | Hs.77439 | chr7q22 |
| 203679_at | transmembrane emp24 protein transport domain containing 1 | TMED1 | NM_006858 | Hs.446686 | chr19p13.2 |
| 203678_at | myotubularin related protein 15 | MTMR15 | NM_014967 | Hs.528123 | chr15q13.2-q13.3 |
| 203677_s_at | Tar (HIV-1) RNA binding protein 2 | TARBP2 | NM_004178 | Hs.326 | chr12q12-q13 |
| 203675_at | nucleobindin 2 | NUCB2 | NM_005013 | Hs.128686 | chr11p15.1-p14 |
| 203672_x_at | thiopurine S-methyltransferase | TPMT | U12387 | Hs.377402 | chr6p22.3 |
| 203669_s_at | diacylglycerol O-acyltransferase homolog 1 (mouse) /// similar to Diacylglycerol O-acyltransferase 1 (Diglyceride acyltransferase) (ACAT-related gene product 1) | DGAT1 | NM_012079 | Hs.512810 | chr8q24.3 |
| 203668_at | mannosidase, alpha, class 2C, member 1 | MAN2C1 | NM_006715 | Hs.26232 | chr15q11-q13 |
| 203666_at | chemokine (C-X-C motif) ligand 12 (stromal cell-derived factor 1) | CXCL12 | NM_000609 | Hs.436042 | chr10q11.1 |
| 203665_at | heme oxygenase (decycling) 1 | HMOX1 | NM_002133 | Hs.202833 | chr22q13.1 |
| 203663_s_at | cytochrome c oxidase subunit Va | COX5A | NM_004255 | Hs.401903 | chr15q25 |
| 203661_s_at | tropomodulin 1 | TMOD1 | BC002660 | Hs.404289 | chr9q22.3 |
| 203660_s_at | pericentrin (kendrin) | PCNT | NM_006031 | Hs.396473 | chr21q22.3 |
| 203658_at | solute carrier family 25 (carnitine/acylcarnitine translocase), member 20 | SLC25A20 | BC001689 | Hs.13845 | chr3p21.31 |
| 203657_s_at | cathepsin F | CTSF | NM_003793 | Hs.11590 | chr11q13 |
| 203656_at | KIAA0274 | KIAA0274 | NM_014845 | Hs.419998 | chr6q21 |
| 203655_at | X-ray repair complementing defective repair in Chinese hamster cells 1 | XRCC1 | NM_006297 | Hs.98493 | chr19q13.2 |
| 203654_s_at | coilin | COIL | NM_004645 | Hs.966 | chr17q22-q23 |
| 203652_at | mitogen-activated protein kinase kinase kinase 11 | MAP3K11 | NM_002419 | Hs.432787 | chr11q13.1-q13.3 |
| 203651_at | zinc finger, FYVE domain containing 16 | ZFYVE16 | NM_014733 | Hs.438240 | chr5p15.2-q14.3 |
| 203650_at | protein C receptor, endothelial (EPCR) | PROCR | NM_006404 | Hs.82353 | chr20q11.2 |
| 203648_at | TatD DNase domain containing 2 | TATDN2 | NM_014760 | Hs.75863 | chr3p25.3 |
| 203643_at | Ets2 repressor factor | ERF | NM_006494 | Hs.440332 | chr19q13 |
| 203642_s_at | COBL-like 1 | COBLL1 | NM_014900 | Hs.443943 | chr2q24.3 |
| 203637_s_at | midline 1 (Opitz/BBB syndrome) | MID1 | NM_000381 | Hs.27695 | chrxp22 |
| 203635_at | Down syndrome critical region gene 3 | DSCR3 | NM_006052 | Hs.26146 | chr21q22.2 |
| 203633_at | carnitine palmitoyltransferase 1A (liver) | CPT1A | BF001714 | Hs.259785 | chr11q13.1-q13.2 |
| 203632_s_at | G protein-coupled receptor, family C, group 5, member B | GPRC5B | NM_016235 | Hs.448805 | chr16p12 |
| 203629_s_at | component of oligomeric golgi complex 5 | COG5 | AU152134 | Hs.239631 | chr7q22-q31 |
| 203628_at | insulin-like growth factor 1 receptor | IGF1R | H05812 | Hs.239176 | chr15q26.3 |
| 203625_x_at | melanoma cell adhesion molecule | MCAM | BG105365 | Hs.23348 | chr5p13 |
| 203624_at | chromosome X and Y open reading frame 3 | CXYorf3 | NM_005088 | Hs.21595 | chrxp22.32; ypter-p11.2 |
| 203623_at | plexin A3 | PLXNA3 | AI675453 | Hs.21432 | chrxq28 |
| 203621_at | NADH dehydrogenase (ubiquinone) 1 beta subcomplex, 5, 16kDa | NDUFB5 | NM_002492 | Hs.19236 | chr3q26.33 |
| 203617_x_at | ELK1, member of ETS oncogene family | ELK1 | NM_005229 | Hs.181128 | chrxp11.2 |
| 203616_at | polymerase (DNA directed), beta | POLB | NM_002690 | Hs.180107 | chr8p11.2 |
| 203615_x_at | sulfotransferase family, cytosolic, 1A, phenol-preferring, member 1 | SULT1A1 | NM_001055 | Hs.142 | chr16p12.1 |
| 203613_s_at | NADH dehydrogenase (ubiquinone) 1 beta subcomplex, 6, 17kDa | NDUFB6 | NM_002493 | Hs.109646 | chr9p21.1 |
| 203611_at | telomeric repeat binding factor 2 | TERF2 | NM_005652 | Hs.63335 | chr16q22.1 |
| 203607_at | inositol polyphosphate-5-phosphatase F | INPP5F | NM_014937 | Hs.52463 | chr10q26.11-q26.12 |
| 203606_at | NADH dehydrogenase (ubiquinone) Fe-S protein 6, 13kDa (NADH-coenzyme Q reductase) | NDUFS6 | NM_004553 | Hs.408257 | chr5p15.33 |
| 203605_at | signal recognition particle 54kDa | SRP54 | NM_003136 | Hs.49346 | chr14q13.2 |
| 203604_at | zinc finger protein 516 | ZNF516 | N38750 | Hs.48450 | chr18q23 |
| 203602_s_at | zinc finger and BTB domain containing 17 | ZBTB17 | NM_003443 | Hs.433764 | chr1p36.2-p36.1 |
| 203600_s_at | chromosome 4 open reading frame 8 | C4orf8 | NM_003704 | Hs.325987 | chr4p16.3 |
| 203597_s_at | WW domain binding protein 4 (formin binding protein 21) | WBP4 | AI734228 | Hs.411300 | chr13q14.11 |
| 203596_s_at | interferon-induced protein with tetratricopeptide repeats 5 | IFIT5 | NM_012420 | Hs.252839 | chr10q23.31 |
| 203594_at | RNA terminal phosphate cyclase domain 1 | RTCD1 | NM_003729 | Hs.274518 | chr1p21.3 |
| 203593_at | CD2-associated protein | CD2AP | NM_012120 | Hs.374340 | chr6p12 |
| 203592_s_at | follistatin-like 3 (secreted glycoprotein) | FSTL3 | NM_005860 | Hs.433827 | chr19p13 |
| 203590_at | dynein, cytoplasmic 1, light intermediate chain 2 | DYNC1LI2 | NM_006141 | Hs.369068 | chr16q22.1 |
| 203588_s_at | transcription factor Dp-2 (E2F dimerization partner 2) | TFDP2 | BG034328 | Hs.379018 | chr3q23 |
| 203586_s_at | ADP-ribosylation factor-like 4D | ARL4D | NM_001661 | Hs.183153 | chr17q12-q21 |
| 203585_at | zinc finger protein 185 (LIM domain) | ZNF185 | NM_007150 | Hs.16622 | chrxq28 |
| 203584_at | tetratricopeptide repeat domain 35 | TTC35 | NM_014673 | Hs.130456 | chr8q23.1 |
| 203583_at | unc-50 homolog (C. elegans) | UNC50 | NM_014044 | Hs.13370 | chr2q11.2 |
| 203582_s_at | RAB4A, member RAS oncogene family | RAB4A | NM_004578 | Hs.296169 | chr1q42-q43 |
| 203577_at | general transcription factor IIH, polypeptide 4, 52kDa | GTF2H4 | NM_001517 | Hs.102910 | chr6p21.3 |
| 203576_at | branched chain aminotransferase 2, mitochondrial | BCAT2 | NM_001190 | Hs.512670 | chr19q13 |
| 203575_at | casein kinase 2, alpha prime polypeptide | CSNK2A2 | NM_001896 | Hs.82201 | chr16p13.3-p13.2 |
| 203574_at | nuclear factor, interleukin 3 regulated | NFIL3 | NM_005384 | Hs.79334 | chr9q22 |
| 203573_s_at | Rab geranylgeranyltransferase, alpha subunit | RABGGTA | NM_004581 | Hs.377992 | chr14q11.2 |
| 203572_s_at | TAF6 RNA polymerase II, TATA box binding protein (TBP)-associated factor, 80kDa | TAF6 | NM_005641 | Hs.289950 | chr7q22.1 |
| 203569_s_at | oral-facial-digital syndrome 1 | OFD1 | NM_003611 | Hs.6483 | chrxp22.2-p22.3 |
| 203567_s_at | tripartite motif-containing 38 | TRIM38 | AU157590 | Hs.404335 | chr6p21.3 |
| 203566_s_at | amylo-1, 6-glucosidase, 4-alpha-glucanotransferase (glycogen debranching enzyme, glycogen storage disease type III) | AGL | NM_000645 | Hs.904 | chr1p21 |
| 203563_at | actin filament associated protein | AFAP | NM_021638 | Hs.115912 | chr4p16 |
| 203562_at | fasciculation and elongation protein zeta 1 (zygin I) | FEZ1 | NM_005103 | Hs.79226 | chr11q24.2 |
| 203560_at | gamma-glutamyl hydrolase (conjugase, folylpolygammaglutamyl hydrolase) | GGH | NM_003878 | Hs.78619 | chr8q12.3 |
| 203557_s_at | pterin-4 alpha-carbinolamine dehydratase/dimerization cofactor of hepatocyte nuclear factor 1 alpha (TCF1) | PCBD1 | NM_000281 | Hs.3192 | chr10q22 |
| 203556_at | zinc fingers and homeoboxes 2 | ZHX2 | NM_014943 | Hs.30209 | chr8q24.13 |
| 203554_x_at | pituitary tumor-transforming 1 | PTTG1 | NM_004219 | Hs.350966 | chr5q35.1 |
| 203550_s_at | chromosome 1 open reading frame 2 | C1orf2 | NM_006589 | Hs.348308 | chr1q21 |
| 203548_s_at | lipoprotein lipase | LPL | BF672975 | Hs.180878 | chr8p22 |
| 203546_at | importin 13 | IPO13 | NM_014652 |  |  |
| 203545_at | asparagine-linked glycosylation 8 homolog (S. cerevisiae, alpha-1,3-glucosyltransferase) | ALG8 | NM_024079 | Hs.440117 | chr11q14.1 |
| 203544_s_at | signal transducing adaptor molecule (SH3 domain and ITAM motif) 1 | STAM | NM_003473 | Hs.441498 | chr10p14-p13 |
| 203542_s_at | Kruppel-like factor 9 | KLF9 | AI690205 | Hs.150557 | chr9q13 |
| 203538_at | calcium modulating ligand /// family with sequence similarity 39, member D pseudogene | CAMLG | NM_001745 | Hs.13572 | chr5q23 |
| 203537_at | phosphoribosyl pyrophosphate synthetase-associated protein 2 | PRPSAP2 | NM_002767 | Hs.462418 | chr17p11.2-p12 |
| 203536_s_at | cytosolic iron-sulfur protein assembly 1 homolog (S. cerevisiae) | CIAO1 | NM_004804 | Hs.12109 | chr2q11.2 |
| 203534_at | LSM1 homolog, U6 small nuclear RNA associated (S. cerevisiae) | LSM1 | NM_014462 | Hs.425311 | chr8p11.2 |
| 203532_x_at | cullin 5 | CUL5 | AF017061 | Hs.440320 | chr11q22-q23 |
| 203530_s_at | syntaxin 4 | STX4 | NM_004604 | Hs.83734 | chr16p11.2 |
| 203524_s_at | mercaptopyruvate sulfurtransferase | MPST | NM_021126 | Hs.248267 | chr22q13.1 |
| 203522_at | copper chaperone for superoxide dismutase | CCS | NM_005125 | Hs.5002 | chr11q13 |
| 203521_s_at | zinc finger protein 318 | ZNF318 | NM_014345 | Hs.147868 | chr6pter-p12.1 |
| 203519_s_at | UPF2 regulator of nonsense transcripts homolog (yeast) | UPF2 | NM_015542 | Hs.3862 | chr10p14-p13 |
| 203518_at | lysosomal trafficking regulator | LYST | NM_000081 | Hs.130188 | chr1q42.1-q42.2 |
| 203517_at | metaxin 2 | MTX2 | NM_006554 | Hs.31584 | chr2q31.1 |
| 203515_s_at | phosphomevalonate kinase | PMVK | NM_006556 | Hs.30954 | chr1p13-q23 |
| 203514_at | mitogen-activated protein kinase kinase kinase 3 | MAP3K3 | BF971923 | Hs.29282 | chr17q23.3 |
| 203511_s_at | trafficking protein particle complex 3 | TRAPPC3 | AF041432 | Hs.288013 | chr1p34.3 |
| 203506_s_at | mediator of RNA polymerase II transcription, subunit 12 homolog (S. cerevisiae) | MED12 | NM_005120 | Hs.409226 | chrxq13 |
| 203504_s_at | ATP-binding cassette, sub-family A (ABC1), member 1 | ABCA1 | NM_005502 | Hs.147259 | chr9q31.1 |
| 203500_at | glutaryl-Coenzyme A dehydrogenase | GCDH | NM_000159 | Hs.292233 | chr19p13.2 |
| 203496_s_at | PPAR binding protein | PPARBP | AF055994 | Hs.15589 | chr17q12-q21.1 |
| 203495_at | leucine rich repeat containing 14 | LRRC14 | NM_014665 | Hs.155650 | chr8q24.3 |
| 203491_s_at | centrosomal protein 57kDa | CEP57 | AI123527 | Hs.101014 | chr11q21 |
| 203490_at | E74-like factor 4 (ets domain transcription factor) | ELF4 | NM_001421 | Hs.151139 | chrxq26 |
| 203484_at | Sec61 gamma subunit | SEC61G | NM_014302 | Hs.9950 | chr7p11.2 |
| 203481_at | chromosome 10 open reading frame 6 | C10orf6 | AI655902 | Hs.447458 | chr10q24.32 |
| 203478_at | NADH dehydrogenase (ubiquinone) 1, subcomplex unknown, 1, 6kDa | NDUFC1 | NM_002494 | Hs.84549 | chr4q28.2-q31.1 |
| 203477_at | collagen, type XV, alpha 1 | COL15A1 | NM_001855 | Hs.409034 | chr9q21-q22 |
| 203476_at | trophoblast glycoprotein | TPBG | NM_006670 | Hs.82128 | chr6q14-q15 |
| 203467_at | phosphomannomutase 1 | PMM1 | NM_002676 | Hs.75835 | chr22q13.2 |
| 203466_at | MpV17 mitochondrial inner membrane protein | MPV17 | NM_002437 | Hs.75659 | chr2p23-p21 |
| 203464_s_at | epsin 2 | EPN2 | NM_014964 | Hs.7407 | chr17p11.2 |
| 203459_s_at | vacuolar protein sorting 16 homolog (S. cerevisiae) | VPS16 | NM_022575 | Hs.306676 | chr20p13-p12 |
| 203458_at | sepiapterin reductase (7,8-dihydrobiopterin:NADP+ oxidoreductase) | SPR | AI951454 | Hs.301540 | chr2p14-p12 |
| 203457_at | syntaxin 7 | STX7 | NM_003569 | Hs.152335 | chr6q23.1 |
| 203456_at | PRA1 domain family, member 2 | PRAF2 | NM_007213 | Hs.29595 | chrxp11.23 |
| 203454_s_at | ATX1 antioxidant protein 1 homolog (yeast) | ATOX1 | NM_004045 |  |  |
| 203450_at | PKD2 interactor, golgi and endoplasmic reticulum associated 1 | PGEA1 | NM_015373 | Hs.334911 | chr22q12 |
| 203448_s_at | telomeric repeat binding factor (NIMA-interacting) 1 | TERF1 | AI347136 | Hs.442707 | chr8q13 |
| 203447_at | proteasome (prosome, macropain) 26S subunit, non-ATPase, 5 | PSMD5 | AU157008 | Hs.193725 | chr9q33.2 |
| 203442_x_at | echinoderm microtubule associated protein like 3 | EML3 | AA478965 | Hs.379785 | chr11q12.3 |
| 203439_s_at | stanniocalcin 2 | STC2 | BC000658 | Hs.155223 | chr5q35.1 |
| 203437_at | transmembrane protein 11 | TMEM11 | NM_003876 | Hs.15196 | chr17p11.2 |
| 203436_at | ribonuclease P/MRP 30kDa subunit | RPP30 | NM_006413 | Hs.139120 | chr10q23.31 |
| 203430_at | heme binding protein 2 | HEBP2 | NM_014320 | Hs.439081 | chr6q24 |
| 203429_s_at | chromosome 1 open reading frame 9 | C1orf9 | NM_016227 | Hs.108636 | chr1q24 |
| 203427_at | ASF1 anti-silencing function 1 homolog A (S. cerevisiae) | ASF1A | NM_014034 | Hs.292316 | chr6q22.31 |
| 203423_at | retinol binding protein 1, cellular | RBP1 | NM_002899 | Hs.101850 | chr3q23 |
| 203420_at | family with sequence similarity 8, member A1 | FAM8A1 | NM_016255 | Hs.95260 | chr6p22-p23 |
| 203419_at | myeloid/lymphoid or mixed-lineage leukemia 4 | MLL4 | NM_014727 | Hs.92236 | chr19q13.1 |
| 203416_at | CD53 molecule | CD53 | NM_000560 | Hs.443057 | chr1p13 |
| 203414_at | monocyte to macrophage differentiation-associated | MMD | NM_012329 | Hs.79889 | chr17q |
| 203413_at | NEL-like 2 (chicken) /// NEL-like 2 (chicken) | NELL2 | NM_006159 | Hs.79389 | chr12q13.11-q13.12 |
| 203412_at | leucine-zipper-like transcription regulator 1 | LZTR1 | NM_006767 | Hs.78788 | chr22q11.21 |
| 203409_at | damage-specific DNA binding protein 2, 48kDa | DDB2 | NM_000107 | Hs.534319 | chr11p12-p11 |
| 203408_s_at | special AT-rich sequence binding protein 1 (binds to nuclear matrix/scaffold-associating DNA's) | SATB1 | NM_002971 | Hs.416026 | chr3p23 |
| 203406_at | microfibrillar-associated protein 1 | MFAP1 | NM_005926 | Hs.61418 | chr15q15-q21 |
| 203405_at | Down syndrome critical region gene 2 | DSCR2 | NM_003720 | Hs.5198 | chr21q22.3 |
| 203404_at | armadillo repeat containing, X-linked 2 | ARMCX2 | NM_014782 | Hs.48924 | chrxq21.33-q22.2 |
| 203403_s_at | ring finger protein (C3H2C3 type) 6 | RNF6 | NM_005977 | Hs.136885 | chr13q12.2 |
| 203396_at | proteasome (prosome, macropain) subunit, alpha type, 4 | PSMA4 | NM_002789 | Hs.251531 | chr15q25.1 |
| 203395_s_at | hairy and enhancer of split 1, (Drosophila) | HES1 | NM_005524 | Hs.250666 | chr3q28-q29 |
| 203391_at | FK506 binding protein 2, 13kDa | FKBP2 | NM_004470 | Hs.227729 | chr11q13.1-q13.3 |
| 203386_at | TBC1 domain family, member 4 | TBC1D4 | AI650848 | Hs.173802 | chr13q22.2 |
| 203385_at | diacylglycerol kinase, alpha 80kDa /// beta-carotene dioxygenase 2 | DGKA | NM_001345 | Hs.172690 | chr12q13.3 |
| 203381_s_at | apolipoprotein E | APOE | N33009 | Hs.110675 | chr19q13.2 |
| 203380_x_at | splicing factor, arginine/serine-rich 5 | SFRS5 | NM_006925 | Hs.166975 | chr14q24 |
| 203379_at | ribosomal protein S6 kinase, 90kDa, polypeptide 1 | RPS6KA1 | NM_002953 | Hs.149957 | chr1p |
| 203377_s_at | cell division cycle 40 homolog (S. cerevisiae) | CDC40 | NM_015891 | Hs.116674 | chr6q21 |
| 203375_s_at | tripeptidyl peptidase II | TPP2 | NM_003291 | Hs.432424 | chr13q32-q33 |
| 203372_s_at | suppressor of cytokine signaling 2 | SOCS2 | AB004903 | Hs.405946 | chr12q |
| 203371_s_at | NADH dehydrogenase (ubiquinone) 1 beta subcomplex, 3, 12kDa | NDUFB3 | NM_002491 | Hs.109760 | chr2q31.3 |
| 203367_at | dual specificity phosphatase 14 | DUSP14 | NM_007026 | Hs.91448 | chr17q12 |
| 203366_at | polymerase (DNA directed), gamma | POLG | NM_002693 | Hs.440902 | chr15q25 |
| 203362_s_at | MAD2 mitotic arrest deficient-like 1 (yeast) | MAD2L1 | NM_002358 | Hs.79078 | chr4q27 |
| 203360_s_at | c-myc binding protein | MYCBP | D50692 | Hs.78221 | chr1p33-p32.2 |
| 203357_s_at | calpain 7 | CAPN7 | NM_014296 | Hs.7145 | chr3p24 |
| 203355_s_at | pleckstrin and Sec7 domain containing 3 | PSD3 | NM_015310 | Hs.408177 | chr8pter-p23.3 |
| 203353_s_at | methyl-CpG binding domain protein 1 | MBD1 | NM_015846 | Hs.405610 | chr18q21 |
| 203352_at | origin recognition complex, subunit 4-like (yeast) | ORC4L | NM_002552 | Hs.439114 | chr2q22-q23 |
| 203347_s_at | metal response element binding transcription factor 2 | MTF2 | NM_007358 | Hs.31016 | chr1p22.1 |
| 203344_s_at | retinoblastoma binding protein 8 | RBBP8 | NM_002894 | Hs.121423 | chr18q11.2 |
| 203343_at | UDP-glucose dehydrogenase | UGDH | NM_003359 | Hs.28309 | chr4p15.1 |
| 203342_at | translocase of inner mitochondrial membrane 17 homolog B (yeast) | TIMM17B | NM_005834 | Hs.19105 | chrxp11.23 |
| 203340_s_at | solute carrier family 25 (mitochondrial carrier, Aralar), member 12 | SLC25A12 | NM_003705 | Hs.462188 | chr2q24 |
| 203336_s_at | integrin beta 1 binding protein 1 | ITGB1BP1 | AL548363 | Hs.408468 | chr2p25.2 |
| 203335_at | phytanoyl-CoA 2-hydroxylase | PHYH | NM_006214 | Hs.172887 | chr10pter-p11.2 |
| 203334_at | DEAH (Asp-Glu-Ala-His) box polypeptide 8 | DHX8 | NM_004941 | Hs.171872 | chr17q21.31 |
| 203333_at | kinesin-associated protein 3 | KIFAP3 | NM_014970 | Hs.433442 | chr1q24.2 |
| 203330_s_at | syntaxin 5 | STX5 | NM_003164 | Hs.421457 | chr11q12.3 |
| 203323_at | caveolin 2 | CAV2 | BF197655 | Hs.139851 | chr7q31.1 |
| 203321_s_at | zinc finger protein 508 | ZNF508 | AK022688 | Hs.131915 | chr18q23 |
| 203320_at | SH2B adaptor protein 3 | SH2B3 | NM_005475 | Hs.13131 | chr12q24 |
| 203318_s_at | zinc finger protein 148 | ZNF148 | NM_021964 | Hs.442787 | chr3q21 |
| 203316_s_at | small nuclear ribonucleoprotein polypeptide E | SNRPE | NM_003094 | Hs.334612 | chr1q32 |
| 203315_at | NCK adaptor protein 2 /// similar to NCK adaptor protein 2 | NCK2 | BC000103 | Hs.101695 | chr2q12 |
| 203314_at | GTP binding protein 6 (putative) | GTPBP6 | NM_012227 | Hs.437145 | chrxp22.33; yp11.32 |
| 203313_s_at | TGFB-induced factor (TALE family homeobox) | TGIF | NM_003244 | Hs.161999 | chr18p11.3 |
| 203311_s_at | ADP-ribosylation factor 6 | ARF6 | M57763 | Hs.89474 | chr14q21.3 |
| 203310_at | syntaxin binding protein 3 | STXBP3 | NM_007269 | Hs.8813 | chr1p13.3 |
| 203307_at | guanine nucleotide binding protein-like 1 | GNL1 | NM_005275 | Hs.83147 | chr6p21.3 |
| 203306_s_at | solute carrier family 35 (CMP-sialic acid transporter), member A1 | SLC35A1 | NM_006416 | Hs.82921 | chr6q15 |
| 203304_at | BMP and activin membrane-bound inhibitor homolog (Xenopus laevis) | BAMBI | NM_012342 | Hs.348802 | chr10p12.3-p11.2 |
| 203303_at | dynein, light chain, Tctex-type 3 | DYNLT3 | NM_006520 | Hs.446392 | chrxp21 |
| 203302_at | deoxycytidine kinase | DCK | NM_000788 | Hs.709 | chr4q13.3-q21.1 |
| 203301_s_at | cyclin D binding myb-like transcription factor 1 | DMTF1 | NM_021145 | Hs.174941 | chr7q21 |
| 203299_s_at | adaptor-related protein complex 1, sigma 2 subunit /// similar to adaptor-related protein complex 1 sigma 2 subunit /// similar to adaptor-related protein complex 1 sigma 2 subunit | AP1S2 | AF251295 | Hs.40368 | chrxp22.2 |
| 203298_s_at | jumonji, AT rich interactive domain 2 | JARID2 | NM_004973 | Hs.40154 | chr6p24-p23 |
| 203292_s_at | vacuolar protein sorting 11 homolog (S. cerevisiae) | VPS11 | NM_021729 | Hs.234282 | chr11q23 |
| 203291_at | CCR4-NOT transcription complex, subunit 4 | CNOT4 | NM_013316 | Hs.20423 | chr7q22-qter |
| 203288_at | KIAA0355 | KIAA0355 | NM_014686 | Hs.436976 | chr19q13.11 |
| 203286_at | ring finger protein 44 | RNF44 | NM_014901 | Hs.434888 | chr5q35.2 |
| 203282_at | glucan (1,4-alpha-), branching enzyme 1 (glycogen branching enzyme, Andersen disease, glycogen storage disease type IV) | GBE1 | NM_000158 | Hs.1691 | chr3p12.3 |
| 203276_at | lamin B1 | LMNB1 | NM_005573 | Hs.89497 | chr5q23.3-q31.1 |
| 203275_at | interferon regulatory factor 2 | IRF2 | NM_002199 | Hs.374097 | chr4q34.1-q35.1 |
| 203274_at | coagulation factor VIII-associated (intronic transcript) 1 | F8A1 | NM_012151 | Hs.387463 | chrxq28 |
| 203272_s_at | tumor suppressor candidate 2 | TUSC2 | BF308548 | Hs.8186 | chr3p21.3 |
| 203265_s_at | mitogen-activated protein kinase kinase 4 | MAP2K4 | AA810268 | Hs.134106 | chr17p11.2 |
| 203264_s_at | Cdc42 guanine nucleotide exchange factor (GEF) 9 | ARHGEF9 | NM_015185 | Hs.54697 | chrxq11.2 |
| 203262_s_at | family with sequence similarity 50, member A | FAM50A | NM_004699 | Hs.54277 | chrxq28 |
| 203261_at | dynactin 6 | DCTN6 | NM_006571 | Hs.158427 | chr8p12-p11 |
| 203259_s_at | HD domain containing 2 | HDDC2 | BC001671 | Hs.32826 | chr6q13-q24.3 |
| 203258_at | DR1-associated protein 1 (negative cofactor 2 alpha) | DRAP1 | NM_006442 | Hs.356742 | chr11q13.3 |
| 203257_s_at | chromosome 11 open reading frame 49 | C11orf49 | NM_024113 | Hs.291003 | chr11p11.2 |
| 203254_s_at | talin 1 | TLN1 | NM_006289 | Hs.375001 | chr9p13 |
| 203253_s_at | histidine acid phosphatase domain containing 1 | HISPPD1 | NM_015216 | Hs.26179 | chr5q21.1 |
| 203252_at | CDK2-associated protein 2 | CDK2AP2 | NM_005851 | Hs.379039 | chr11q13 |
| 203250_at | RNA binding motif protein 16 | RBM16 | NM_014892 | Hs.227602 | chr6q25.1-q25.3 |
| 203248_at | zinc finger protein 24 | ZNF24 | NM_006965 | Hs.173911 | chr18q12 |
| 203246_s_at | tumor suppressor candidate 4 | TUSC4 | NM_006545 | Hs.437083 | chr3p21.3 |
| 203244_at | peroxisomal biogenesis factor 5 | PEX5 | NM_000319 | Hs.342386 | chr12p13.3 |
| 203241_at | UV radiation resistance associated gene | UVRAG | NM_003369 | Hs.446223 | chr11q13.5 |
| 203239_s_at | CCR4-NOT transcription complex, subunit 3 | CNOT3 | NM_014516 | Hs.172407 | chr19q13.4 |
| 203235_at | thimet oligopeptidase 1 | THOP1 | NM_003249 | Hs.78769 | chr19q13.3 |
| 203233_at | interleukin 4 receptor | IL4R | NM_000418 | Hs.75545 | chr16p11.2-12.1 |
| 203230_at | dishevelled, dsh homolog 1 (Drosophila) /// similar to dishevelled 1 isoform a | DVL1 | AF006011 | Hs.74375 | chr1p36 |
| 203229_s_at | CDC-like kinase 2 | CLK2 | NM_003993 | Hs.73986 | chr1q21 |
| 203226_s_at | tetraspanin 31 | TSPAN31 | AL514076 | Hs.50984 | chr12q13.3 |
| 203224_at | riboflavin kinase | RFK | BF340123 | Hs.37558 | chr9q21.13 |
| 203222_s_at | transducin-like enhancer of split 1 (E(sp1) homolog, Drosophila) | TLE1 | NM_005077 | Hs.406491 | chr9q21.32 |
| 203219_s_at | adenine phosphoribosyltransferase | APRT | NM_000485 | Hs.28914 | chr16q24 |
| 203218_at | mitogen-activated protein kinase 9 | MAPK9 | W37431 | Hs.348446 | chr5q35 |
| 203217_s_at | ST3 beta-galactoside alpha-2,3-sialyltransferase 5 | ST3GAL5 | NM_003896 | Hs.415117 | chr2p11.2 |
| 203215_s_at | myosin VI | MYO6 | AA877789 | Hs.118483 | chr6q13 |
| 203213_at | cell division cycle 2, G1 to S and G2 to M | CDC2 | AL524035 | Hs.334562 | chr10q21.1 |
| 203209_at | replication factor C (activator 1) 5, 36.5kDa | RFC5 | BC001866 | Hs.443227 | chr12q24.2-q24.3 |
| 203207_s_at | mitochondrial fission regulator 1 | MTFR1 | BF214329 | Hs.170198 | chr8q13.1 |
| 203206_at | family with sequence similarity 53, member B | FAM53B | NM_014661 | Hs.156016 | chr10q26.13 |
| 203205_at | jumonji domain containing 2A | JMJD2A | NM_014663 | Hs.155983 | chr1p34.2-p34.1 |
| 203202_at | KRR1, small subunit (SSU) processome component, homolog (yeast) | KRR1 | AI950314 | Hs.511765 | chr12q21.1 |
| 203201_at | phosphomannomutase 2 | PMM2 | NM_000303 | Hs.159914 | chr16p13.3-p13.2 |
| 203199_s_at | 5-methyltetrahydrofolate-homocysteine methyltransferase reductase | MTRR | N29717 | Hs.153792 | chr5p15.3-p15.2 |
| 203198_at | cyclin-dependent kinase 9 (CDC2-related kinase) | CDK9 | NM_001261 | Hs.150423 | chr9q34.1 |
| 203197_s_at | chromosome 1 open reading frame 123 | C1orf123 | AW157077 | Hs.146861 | chr1p32.3 |
| 203194_s_at | nucleoporin 98kDa | NUP98 | AA527238 | Hs.112255 | chr11p15.5 |
| 203192_at | ATP-binding cassette, sub-family B (MDR/TAP), member 6 | ABCB6 | NM_005689 | Hs.107911 | chr2q36 |
| 203189_s_at | NADH dehydrogenase (ubiquinone) Fe-S protein 8, 23kDa (NADH-coenzyme Q reductase) | NDUFS8 | NM_002496 | Hs.90443 | chr11q13 |
| 203188_at | UDP-GlcNAc:betaGal beta-1,3-N-acetylglucosaminyltransferase 1 | B3GNT1 | NM_006876 | Hs.8526 | chr11q13.2 |
| 203185_at | Ras association (RalGDS/AF-6) domain family 2 | RASSF2 | NM_014737 | Hs.80905 | chr20pter-p12.1 |
| 203180_at | aldehyde dehydrogenase 1 family, member A3 | ALDH1A3 | NM_000693 | Hs.75746 | chr15q26.3 |
| 203179_at | galactose-1-phosphate uridylyltransferase /// chromosome 10 open reading frame 4 | GALT | NM_000155 | Hs.75641 | chr9p13 |
| 203178_at | glycine amidinotransferase (L-arginine:glycine amidinotransferase) | GATM | NM_001482 | Hs.75335 | chr15q21.1 |
| 203176_s_at | transcription factor A, mitochondrial | TFAM | BE552470 | Hs.75133 | chr10q21 |
| 203175_at | ras homolog gene family, member G (rho G) | RHOG | NM_001665 | Hs.75082 | chr11p15.5-p15.4 |
| 203172_at | fragile X mental retardation, autosomal homolog 2 | FXR2 | NM_004860 | Hs.52788 | chr17p13.1 |
| 203171_s_at | KIAA0409 | KIAA0409 | NM_015324 | Hs.511948 | chr11p15.4 |
| 203169_at | KIAA0258 | KIAA0258 | NM_014785 | Hs.534415 | chr9pter-p22.1 |
| 203168_at | cAMP responsive element binding protein-like 1 | CREBL1 | NM_004381 | Hs.42853 | chr6p21.3 |
| 203167_at | TIMP metallopeptidase inhibitor 2 | TIMP2 | NM_003255 | Hs.6441 | chr17q25 |
| 203166_at | craniofacial development protein 1 | CFDP1 | NM_006324 | Hs.433402 | chr16q22.2-q22.3 |
| 203162_s_at | katanin p80 (WD repeat containing) subunit B 1 | KATNB1 | NM_005886 | Hs.275675 | chr16q13 |
| 203160_s_at | ring finger protein 8 | RNF8 | AK022075 | Hs.533306 | chr6p21.3 |
| 203158_s_at | glutaminase | GLS | AF097493 | Hs.128410 | chr2q32-q34 |
| 203154_s_at | p21(CDKN1A)-activated kinase 4 | PAK4 | NM_005884 | Hs.20447 | chr19q13.2 |
| 203152_at | mitochondrial ribosomal protein L40 | MRPL40 | NM_003776 | Hs.431307 | chr22q11.21 |
| 203151_at | microtubule-associated protein 1A | MAP1A | AW296788 | Hs.194301 | chr15q13-qter |
| 203150_at | Rab9 effector protein with kelch motifs | RABEPK | NM_005833 | Hs.19012 | chr9q33.3 |
| 203149_at | poliovirus receptor-related 2 (herpesvirus entry mediator B) | PVRL2 | NM_002856 | Hs.183986 | chr19q13.2-q13.4 |
| 203148_s_at | tripartite motif-containing 14 | TRIM14 | NM_014788 | Hs.370530 | chr9q22.33 |
| 203141_s_at | adaptor-related protein complex 3, beta 1 subunit | AP3B1 | AW058575 | Hs.446648 | chr5q14.1 |
| 203139_at | death-associated protein kinase 1 | DAPK1 | NM_004938 | Hs.244318 | chr9q34.1 |
| 203138_at | histone acetyltransferase 1 | HAT1 | NM_003642 | Hs.13340 | chr2q31.2-q33.1 |
| 203136_at | Rab acceptor 1 (prenylated) | RABAC1 | NM_006423 | Hs.11417 | chr19q13.2 |
| 203135_at | TATA box binding protein | TBP | NM_003194 | Hs.1100 | chr6q27 |
| 203133_at | Sec61 beta subunit | SEC61B | NM_006808 | Hs.191887 | chr9q22.32-q31.3 |
| 203132_at | retinoblastoma 1 (including osteosarcoma) | RB1 | NM_000321 | Hs.408528 | chr13q14.2 |
| 203123_s_at | solute carrier family 11 (proton-coupled divalent metal ion transporters), member 2 | SLC11A2 | AU154469 | Hs.57435 | chr12q13 |
| 203122_at | tetratricopeptide repeat domain 15 | TTC15 | NM_016030 | Hs.5008 | chr2p25.2 |
| 203120_at | tumor protein p53 binding protein, 2 | TP53BP2 | NM_005426 | Hs.44585 | chr1q42.1 |
| 203119_at | coiled-coil domain containing 86 | CCDC86 | NM_024098 | Hs.4253 | chr11q12.2 |
| 203117_s_at | ubiquitin specific peptidase 52 | USP52 | NM_014871 | Hs.273397 | chr12q13.2-q13.3 |
| 203115_at | ferrochelatase (protoporphyria) | FECH | AU152635 | Hs.443610 | chr18q21.3 |
| 203112_s_at | Wolf-Hirschhorn syndrome candidate 2 | WHSC2 | NM_005663 | Hs.21771 | chr4p16.3 |
| 203109_at | ubiquitin-conjugating enzyme E2M (UBC12 homolog, yeast) | UBE2M | NM_003969 | Hs.406068 | chr19q13.43 |
| 203108_at | G protein-coupled receptor, family C, group 5, member A | GPRC5A | NM_003979 | Hs.194691 | chr12p13-p12.3 |
| 203106_s_at | vacuolar protein sorting 41 homolog (S. cerevisiae) | VPS41 | NM_014396 | Hs.413840 | chr7p14-p13 |
| 203105_s_at | dynamin 1-like | DNM1L | NM_012062 | Hs.180628 | chr12p11.21 |
| 203103_s_at | PRP19/PSO4 pre-mRNA processing factor 19 homolog (S. cerevisiae) | PRPF19 | NM_014502 | Hs.173980 | chr11q12.2 |
| 203102_s_at | mannosyl (alpha-1,6-)-glycoprotein beta-1,2-N-acetylglucosaminyltransferase | MGAT2 | NM_002408 | Hs.93338 | chr14q21 |
| 203100_s_at | chromodomain protein, Y-like | CDYL | NM_004824 | Hs.16081 | chr6p25.1 |
| 203096_s_at | Rap guanine nucleotide exchange factor (GEF) 2 | RAPGEF2 | BF439282 | Hs.373588 | chr4q32.1 |
| 203095_at | mitochondrial translational initiation factor 2 | MTIF2 | NM_002453 | Hs.149894 | chr2p14-p16 |
| 203094_at | MAD2L1 binding protein | MAD2L1BP | NM_014628 | Hs.122346 | chr6p21.1 |
| 203093_s_at | translocase of inner mitochondrial membrane 44 homolog (yeast) | TIMM44 | NM_006351 | Hs.123178 | chr19p13.3-p13.2 |
| 203090_at | stromal cell-derived factor 2 | SDF2 | NM_006923 | Hs.118684 | chr17q11.2 |
| 203088_at | fibulin 5 | FBLN5 | NM_006329 | Hs.11494 | chr14q32.1 |
| 203087_s_at | kinesin heavy chain member 2A | KIF2A | NM_004520 | Hs.519342 | chr5q12-q13 |
| 203085_s_at | transforming growth factor, beta 1 (Camurati-Engelmann disease) | TGFB1 | BC000125 | Hs.1103 | chr19q13.1 |
| 203082_at | BMS1-like, ribosome assembly protein (yeast) | BMS1L | NM_014753 | Hs.10848 | chr10q11.21 |
| 203080_s_at | bromodomain adjacent to zinc finger domain, 2B | BAZ2B | NM_013450 | Hs.388208 | chr2q23-q24 |
| 203078_at | cullin 2 | CUL2 | U83410 | Hs.82919 | chr10p11.21 |
| 203073_at | component of oligomeric golgi complex 2 | COG2 | NM_007357 | Hs.82399 | chr1q42.2 |
| 203072_at | myosin IE /// similar to CDK105 protein | MYO1E | NM_004998 | Hs.437459 | chr15q21-q22 |
| 203071_at | sema domain, immunoglobulin domain (Ig), short basic domain, secreted, (semaphorin) 3B | SEMA3B | NM_004636 | Hs.82222 | chr3p21.3 |
| 203069_at | synaptic vesicle glycoprotein 2A | SV2A | NM_014849 | Hs.7979 | chr1q21.2 |
| 203067_at | pyruvate dehydrogenase complex, component X | PDHX | NM_003477 | Hs.329817 | chr11p13 |
| 203065_s_at | caveolin 1, caveolae protein, 22kDa | CAV1 | NM_001753 | Hs.74034 | chr7q31.1 |
| 203062_s_at | mediator of DNA damage checkpoint 1 | MDC1 | NM_014641 | Hs.433653 | chr6pter-p21.31 |
| 203059_s_at | 3'-phosphoadenosine 5'-phosphosulfate synthase 2 | PAPSS2 | NM_004670 | Hs.274230 | chr10q23-q24 |
| 203054_s_at | T-cell leukemia translocation altered gene | TCTA | NM_022171 | Hs.250894 | chr3p21 |
| 203053_at | breast carcinoma amplified sequence 2 | BCAS2 | NM_005872 | Hs.22960 | chr1p21-p13.3 |
| 203051_at | bromo adjacent homology domain containing 1 | BAHD1 | NM_014952 | Hs.22109 | chr15q15.1 |
| 203050_at | tumor protein p53 binding protein, 1 | TP53BP1 | NM_005657 | Hs.440968 | chr15q15-q21 |
| 203046_s_at | timeless homolog (Drosophila) | TIMELESS | NM_003920 | Hs.118631 | chr12q12-q13 |
| 203044_at | carbohydrate (chondroitin) synthase 1 | CHSY1 | NM_014918 | Hs.110488 | chr15q26.3 |
| 203040_s_at | hydroxymethylbilane synthase | HMBS | NM_000190 | Hs.82609 | chr11q23.3 |
| 203038_at | protein tyrosine phosphatase, receptor type, K | PTPRK | NM_002844 | Hs.354262 | chr6q22.2-23.1 |
| 203037_s_at | metastasis suppressor 1 | MTSS1 | NM_014751 | Hs.77694 | chr8p22 |
| 203035_s_at | protein inhibitor of activated STAT, 3 | PIAS3 | NM_006099 | Hs.435761 | chr1q21 |
| 203033_x_at | fumarate hydratase | FH | NM_000143 | Hs.391168 | chr1q42.1 |
| 203029_s_at | protein tyrosine phosphatase, receptor type, N polypeptide 2 | PTPRN2 | NM_002847 | Hs.74624 | chr7q36 |
| 203028_s_at | cytochrome b-245, alpha polypeptide | CYBA | NM_000101 | Hs.68877 | chr16q24 |
| 203027_s_at | mevalonate (diphospho) decarboxylase | MVD | AI189359 | Hs.252457 | chr16q24.3 |
| 203026_at | zinc finger and BTB domain containing 5 | ZBTB5 | NM_014872 | Hs.3682 | chr9p13.2 |
| 203025_at | ARD1 homolog A, N-acetyltransferase (S. cerevisiae) | ARD1A | NM_003491 | Hs.433291 | chrxq28 |
| 203024_s_at | chromosome 5 open reading frame 15 | C5orf15 | NM_020199 | Hs.283437 | chr5q31.1 |
| 203023_at | hypothetical protein HSPC111 | HSPC111 | NM_016391 | Hs.529475 | chr5q35.2 |
| 203022_at | ribonuclease H2, subunit A | RNASEH2A | NM_006397 | Hs.25292 | chr19p13.13 |
| 203013_at | ecdysoneless homolog (Drosophila) | ECD | NM_007265 | Hs.446373 | chr10q22.1 |
| 203012_x_at | ribosomal protein L23a | RPL23A | NM_000984 | Hs.419463 | chr17q11 |
| 203011_at | inositol(myo)-1(or 4)-monophosphatase 1 | IMPA1 | NM_005536 | Hs.334022 | chr8q21.13-q21.3 |
| 203008_x_at | thioredoxin domain containing 9 | TXNDC9 | NM_005783 | Hs.153884 | chr2q11.2 |
| 203007_x_at | lysophospholipase I | LYPLA1 | AF077198 | Hs.446676 | chr8q11.23 |
| 203006_at | inositol polyphosphate-5-phosphatase, 40kDa | INPP5A | NM_005539 | Hs.408063 | chr10q26.3 |
| 203005_at | lymphotoxin beta receptor (TNFR superfamily, member 3) | LTBR | NM_002342 | Hs.1116 | chr12p13 |
| 203004_s_at | MADS box transcription enhancer factor 2, polypeptide D (myocyte enhancer factor 2D) | MEF2D | NM_005920 | Hs.77955 | chr1q12-q23 |
| 203002_at | angiomotin like 2 | AMOTL2 | NM_016201 | Hs.426312 | chr3q21-q22 |
| 202998_s_at | lysyl oxidase-like 2 | LOXL2 | NM_002318 | Hs.83354 | chr8p21.3-p21.2 |
| 202996_at | polymerase (DNA-directed), delta 4 | POLD4 | NM_021173 | Hs.82520 | chr11q13 |
| 202993_at | ilvB (bacterial acetolactate synthase)-like | ILVBL | NM_006844 | Hs.78880 | chr19p13.1 |
| 202991_at | START domain containing 3 | STARD3 | NM_006804 | Hs.77628 | chr17q11-q12 |
| 202990_at | phosphorylase, glycogen; liver (Hers disease, glycogen storage disease type VI) | PYGL | NM_002863 | Hs.282417 | chr14q21-q22 |
| 202985_s_at | BCL2-associated athanogene 5 | BAG5 | NM_004873 | Hs.5443 | chr14q32.32 |
| 202983_at | helicase-like transcription factor | HLTF | AI760760 | Hs.3068 | chr3q25.1-q26.1 |
| 202982_s_at | acyl-CoA thioesterase 2 /// acyl-CoA thioesterase 1 | ACOT2 | NM_006821 | Hs.446685 | chr14q24.3 |
| 202980_s_at | seven in absentia homolog 1 (Drosophila) | SIAH1 | AI953523 | Hs.295923 | chr16q12 |
| 202978_s_at | CREB/ATF bZIP transcription factor | CREBZF | AW204564 | Hs.534553 | chr11q14 |
| 202974_at | membrane protein, palmitoylated 1, 55kDa | MPP1 | NM_002436 | Hs.422215 | chrxq28 |
| 202972_s_at | family with sequence similarity 13, member A1 | FAM13A1 | AW450403 | Hs.442818 | chr4q22.1 |
| 202968_s_at | dual-specificity tyrosine-(Y)-phosphorylation regulated kinase 2 | DYRK2 | Y09216 | Hs.173135 | chr12q15 |
| 202964_s_at | regulatory factor X, 5 (influences HLA class II expression) | RFX5 | NM_000449 | Hs.166891 | chr1q21 |
| 202962_at | kinesin family member 13B | KIF13B | NM_015254 | Hs.15711 | chr8p12 |
| 202961_s_at | ATP synthase, H+ transporting, mitochondrial F0 complex, subunit F2 | ATP5J2 | NM_004889 | Hs.235557 | chr7q22.1 |
| 202959_at | methylmalonyl Coenzyme A mutase | MUT | AI433712 | Hs.155212 | chr6p21 |
| 202958_at | protein tyrosine phosphatase, non-receptor type 9 | PTPN9 | NM_002833 | Hs.147663 | chr15q23 |
| 202955_s_at | ADP-ribosylation factor guanine nucleotide-exchange factor 1(brefeldin A-inhibited) | ARFGEF1 | AF084520 | Hs.94631 | chr8q13 |
| 202952_s_at | ADAM metallopeptidase domain 12 (meltrin alpha) | ADAM12 | NM_003474 | Hs.8850 | chr10q26.3 |
| 202950_at | crystallin, zeta (quinone reductase) | CRYZ | NM_001889 | Hs.83114 | chr1p31-p22 |
| 202949_s_at | four and a half LIM domains 2 | FHL2 | NM_001450 | Hs.8302 | chr2q12-q14 |
| 202947_s_at | glycophorin C (Gerbich blood group) | GYPC | NM_002101 | Hs.81994 | chr2q14-q21 |
| 202945_at | folylpolyglutamate synthase | FPGS | NM_004957 | Hs.754 | chr9cen-q34 |
| 202942_at | electron-transfer-flavoprotein, beta polypeptide | ETFB | NM_001985 | Hs.187845 | chr19q13.3 |
| 202939_at | zinc metallopeptidase (STE24 homolog, yeast) | ZMPSTE24 | NM_005857 | Hs.25846 | chr1p34 |
| 202935_s_at | SRY (sex determining region Y)-box 9 (campomelic dysplasia, autosomal sex-reversal) | SOX9 | AI382146 | Hs.2316 | chr17q24.3-q25.1 |
| 202934_at | hexokinase 2 | HK2 | AI761561 | Hs.406266 | chr2p13 |
| 202932_at | v-yes-1 Yamaguchi sarcoma viral oncogene homolog 1 | YES1 | NM_005433 | Hs.194148 | chr18p11.31-p11.21 |
| 202931_x_at | bridging integrator 1 | BIN1 | NM_004305 | Hs.193163 | chr2q14 |
| 202930_s_at | succinate-CoA ligase, ADP-forming, beta subunit | SUCLA2 | NM_003850 | Hs.182217 | chr13q12.2-q13.3 |
| 202929_s_at | D-dopachrome tautomerase | DDT | NM_001355 | Hs.433902 | chr22q11.23 |
| 202927_at | protein (peptidylprolyl cis/trans isomerase) NIMA-interacting 1 | PIN1 | NM_006221 | Hs.434863 | chr19p13 |
| 202926_at | neuroblastoma-amplified protein | NAG | NM_015909 | Hs.413771 | chr2p24 |
| 202925_s_at | pleiomorphic adenoma gene-like 2 | PLAGL2 | NM_002657 | Hs.154104 | chr20q11.21 |
| 202922_at | glutamate-cysteine ligase, catalytic subunit | GCLC | BF676980 | Hs.414985 | chr6p12 |
| 202920_at | ankyrin 2, neuronal | ANK2 | BF726212 | Hs.480569 | chr4q25-q27 |
| 202919_at | preimplantation protein 3 | PREI3 | NM_015387 | Hs.107942 | chr2q33.1 |
| 202917_s_at | S100 calcium binding protein A8 | S100A8 | NM_002964 | Hs.416073 | chr1q21 |
| 202915_s_at | family with sequence similarity 20, member B | FAM20B | BF115776 | Hs.5737 | chr1p36.13-q41 |
| 202912_at | adrenomedullin | ADM | NM_001124 | Hs.441047 | chr11p15.4 |
| 202909_at | EPM2A (laforin) interacting protein 1 | EPM2AIP1 | NM_014805 | Hs.28020 | chr3p22.1 |
| 202908_at | Wolfram syndrome 1 (wolframin) | WFS1 | NM_006005 | Hs.26077 | chr4p16 |
| 202904_s_at | LSM5 homolog, U6 small nuclear RNA associated (S. cerevisiae) | LSM5 | NM_012322 | Hs.424908 | chr7p14.3 |
| 202900_s_at | nucleoporin 88kDa | NUP88 | NM_002532 | Hs.188446 | chr17p13.2 |
| 202898_at | syndecan 3 (N-syndecan) | SDC3 | NM_014654 | Hs.158287 | chr1pter-p22.3 |
| 202896_s_at | signal-regulatory protein alpha | SIRPA | NM_004648 | Hs.128846 | chr20p13 |
| 202893_at | unc-13 homolog B (C. elegans) | UNC13B | NM_006377 | Hs.155001 | chr9p12-p11 |
| 202891_at | nitrilase 1 | NIT1 | NM_005600 | Hs.169681 | chr1q21-q22 |
| 202887_s_at | DNA-damage-inducible transcript 4 | DDIT4 | NM_019058 | Hs.111244 | chr10pter-q26.12 |
| 202883_s_at | protein phosphatase 2 (formerly 2A), regulatory subunit A (PR 65), beta isoform | PPP2R1B | T79584 | Hs.306864 | chr11q23.2 |
| 202882_x_at | nucleolar protein 7, 27kDa | NOL7 | NM_016167 | Hs.189023 | chr6p23 |
| 202880_s_at | pleckstrin homology, Sec7 and coiled-coil domains 1(cytohesin 1) | PSCD1 | NM_004762 | Hs.1050 | chr17q25 |
| 202871_at | TNF receptor-associated factor 4 | TRAF4 | NM_004295 | Hs.8375 | chr17q11-q12 |
| 202868_s_at | processing of precursor 4, ribonuclease P/MRP subunit (S. cerevisiae) | POP4 | NM_006627 | Hs.412870 | chr19q12 |
| 202866_at | DnaJ (Hsp40) homolog, subfamily B, member 12 | DNAJB12 | BG283782 | Hs.7960 | chr10q22.1 |
| 202862_at | fumarylacetoacetate hydrolase (fumarylacetoacetase) | FAH | NM_000137 | Hs.73875 | chr15q23-q25 |
| 202861_at | period homolog 1 (Drosophila) | PER1 | NM_002616 | Hs.445534 | chr17p13.1-17p12 |
| 202860_at | DENN/MADD domain containing 4B | DENND4B | NM_014856 | Hs.505623 | chr1p36.13-q31.3 |
| 202858_at | U2 small nuclear RNA auxiliary factor 1 | U2AF1 | NM_006758 | Hs.365116 | chr21q22.3 |
| 202854_at | hypoxanthine phosphoribosyltransferase 1 (Lesch-Nyhan syndrome) | HPRT1 | NM_000194 | Hs.412707 | chrxq26.1 |
| 202852_s_at | hypothetical protein FLJ11506 | FLJ11506 | NM_024666 | Hs.254642 | chr15q22.33-q23 |
| 202847_at | phosphoenolpyruvate carboxykinase 2 (mitochondrial) | PCK2 | NM_004563 | Hs.75812 | chr14q11.2 |
| 202843_at | DnaJ (Hsp40) homolog, subfamily B, member 9 | DNAJB9 | NM_012328 | Hs.6790 | chr7q31 |
| 202839_s_at | NADH dehydrogenase (ubiquinone) 1 beta subcomplex, 7, 18kDa | NDUFB7 | NM_004146 | Hs.433482 | chr19p13.12-p13.11 |
| 202838_at | fucosidase, alpha-L- 1, tissue | FUCA1 | NM_000147 | Hs.576 | chr1p34 |
| 202837_at | TRAF-type zinc finger domain containing 1 | TRAFD1 | NM_006700 | Hs.5148 | chr12q |
| 202836_s_at | thioredoxin-like 4A | TXNL4A | NM_006701 | Hs.433683 | chr18q23 |
| 202832_at | GRIP and coiled-coil domain containing 2 | GCC2 | NM_014635 | Hs.278671 | chr2q12.3 |
| 202829_s_at | synaptobrevin-like 1 | SYBL1 | NM_005638 | Hs.24167 | chrxq28 |
| 202825_at | solute carrier family 25 (mitochondrial carrier; adenine nucleotide translocator), member 4 | SLC25A4 | NM_001151 | Hs.2043 | chr4q35 |
| 202823_at | transcription elongation factor B (SIII), polypeptide 1 (15kDa, elongin C) | TCEB1 | N89607 |  |  |
| 202820_at | aryl hydrocarbon receptor | AHR | NM_001621 | Hs.170087 | chr7p15 |
| 202819_s_at | transcription elongation factor B (SIII), polypeptide 3 (110kDa, elongin A) | TCEB3 | NM_003198 | Hs.15535 | chr1p36.1 |
| 202815_s_at | hexamethylene bis-acetamide inducible 1 | HEXIM1 | NM_006460 | Hs.15299 | chr17q21.31 |
| 202812_at | glucosidase, alpha; acid (Pompe disease, glycogen storage disease type II) | GAA | NM_000152 | Hs.1437 | chr17q25.2-q25.3 |
| 202810_at | developmentally regulated GTP binding protein 1 | DRG1 | NM_004147 | Hs.115242 | chr22q12.2 |
| 202809_s_at | integrator complex subunit 3 | INTS3 | NM_023015 | Hs.105894 | chr1q21.3 |
| 202807_s_at | target of myb1 (chicken) | TOM1 | NM_005488 | Hs.9482 | chr22q13.1 |
| 202805_s_at | ATP-binding cassette, sub-family C (CFTR/MRP), member 1 | ABCC1 | NM_004996 | Hs.391464 | chr16p13.1 |
| 202801_at | protein kinase, cAMP-dependent, catalytic, alpha | PRKACA | NM_002730 | Hs.194350 | chr19p13.1 |
| 202799_at | ClpP caseinolytic peptidase, ATP-dependent, proteolytic subunit homolog (E. coli) | CLPP | NM_006012 | Hs.317335 | chr19p13.3 |
| 202798_at | SEC24 related gene family, member B (S. cerevisiae) | SEC24B | NM_006323 | Hs.7239 | chr4q25 |
| 202797_at | SAC1 suppressor of actin mutations 1-like (yeast) | SACM1L | NM_014016 | Hs.5867 | chr3p21.3 |
| 202796_at | synaptopodin | SYNPO | NM_007286 | Hs.435228 | chr5q33.1 |
| 202794_at | inositol polyphosphate-1-phosphatase | INPP1 | NM_002194 | Hs.32309 | chr2q32 |
| 202791_s_at | SAPS domain family, member 2 | SAPS2 | AK022669 | Hs.449098 | chr22q13.33 |
| 202789_at | phospholipase C, gamma 1 /// copine family member IX | PLCG1 | AL022394 | Hs.268177 | chr20q12-q13.1 |
| 202788_at | mitogen-activated protein kinase-activated protein kinase 3 | MAPKAPK3 | NM_004635 | Hs.234521 | chr3p21.3 |
| 202786_at | serine threonine kinase 39 (STE20/SPS1 homolog, yeast) | STK39 | NM_013233 | Hs.199263 | chr2q24.3 |
| 202785_at | NADH dehydrogenase (ubiquinone) 1 alpha subcomplex, 7, 14.5kDa | NDUFA7 | NM_005001 | Hs.19561 | chr19p13.2 |
| 202783_at | nicotinamide nucleotide transhydrogenase | NNT | U40490 | Hs.106620 | chr5p13.1-5cen |
| 202781_s_at | skeletal muscle and kidney enriched inositol phosphatase | SKIP | AI806031 | Hs.178347 | chr17p13.3 |
| 202780_at | 3-oxoacid CoA transferase 1 | OXCT1 | NM_000436 | Hs.177584 | chr5p13.1 |
| 202779_s_at | ubiquitin-conjugating enzyme E2S /// similar to Ubiquitin-conjugating enzyme E2S (Ubiquitin-conjugating enzyme E2-24 kDa) (Ubiquitin-protein ligase) (Ubiquitin carrier protein) (E2-EPF5) | UBE2S | NM_014501 | Hs.396393 | chr19q13.43 |
| 202777_at | soc-2 suppressor of clear homolog (C. elegans) | SHOC2 | NM_007373 | Hs.104315 | chr10q25 |
| 202776_at | deoxynucleotidyltransferase, terminal, interacting protein 2 | DNTTIP2 | NM_014597 | Hs.85769 | chr1p22.1 |
| 202775_s_at | splicing factor, arginine/serine-rich 8 (suppressor-of-white-apricot homolog, Drosophila) | SFRS8 | NM_004592 | Hs.213739 | chr12q24.33 |
| 202772_at | 3-hydroxymethyl-3-methylglutaryl-Coenzyme A lyase (hydroxymethylglutaricaciduria) | HMGCL | NM_000191 | Hs.444925 | chr1p36.1-p35 |
| 202767_at | acid phosphatase 2, lysosomal | ACP2 | NM_001610 | Hs.75589 | chr11p11.2-p11.11 |
| 202765_s_at | fibrillin 1 | FBN1 | AI264196 | Hs.750 | chr15q21.1 |
| 202763_at | caspase 3, apoptosis-related cysteine peptidase | CASP3 | NM_004346 | Hs.141125 | chr4q34 |
| 202761_s_at | spectrin repeat containing, nuclear envelope 2 | SYNE2 | NM_015180 | Hs.129541 | chr14q23.2 |
| 202759_s_at | A kinase (PRKA) anchor protein 2 /// PALM2-AKAP2 protein | AKAP2 | BE879367 | Hs.386224 | chr9q31-q33 |
| 202758_s_at | regulatory factor X-associated ankyrin-containing protein | RFXANK | NM_003721 | Hs.296776 | chr19p12 |
| 202757_at | cofactor of BRCA1 | COBRA1 | NM_015456 | Hs.410095 | chr9q34 |
| 202755_s_at | glypican 1 | GPC1 | AI354864 | Hs.328232 | chr2q35-q37 |
| 202754_at | R3H domain containing 1 | R3HDM1 | NM_015361 | Hs.144476 | chr2q21.3 |
| 202753_at | proteasome (prosome, macropain) 26S subunit, non-ATPase, 6 | PSMD6 | NM_014814 | Hs.350939 | chr3p14.1 |
| 202749_at | tryptophan rich basic protein | WRB | NM_004627 | Hs.198308 | chr21q22.3 |
| 202738_s_at | phosphorylase kinase, beta | PHKB | BG149218 | Hs.78060 | chr16q12-q13 |
| 202737_s_at | LSM4 homolog, U6 small nuclear RNA associated (S. cerevisiae) | LSM4 | NM_012321 | Hs.76719 | chr19p13.11 |
| 202732_at | protein kinase (cAMP-dependent, catalytic) inhibitor gamma | PKIG | NM_007066 | Hs.3407 | chr20q12-q13.1 |
| 202728_s_at | latent transforming growth factor beta binding protein 1 | LTBP1 | AI986120 | Hs.368256 | chr2p22-p21 |
| 202725_at | polymerase (RNA) II (DNA directed) polypeptide A, 220kDa | POLR2A | NM_000937 | Hs.171880 | chr17p13.1 |
| 202723_s_at | forkhead box O1A (rhabdomyosarcoma) | FOXO1A | AW117498 | Hs.170133 | chr13q14.1 |
| 202722_s_at | glutamine-fructose-6-phosphate transaminase 1 | GFPT1 | NM_002056 | Hs.1674 | chr2p13 |
| 202718_at | insulin-like growth factor binding protein 2, 36kDa | IGFBP2 | NM_000597 | Hs.433326 | chr2q33-q34 |
| 202717_s_at | cell division cycle 16 homolog (S. cerevisiae) | CDC16 | NM_003903 | Hs.1592 | chr13q34 |
| 202716_at | protein tyrosine phosphatase, non-receptor type 1 | PTPN1 | NM_002827 | Hs.418004 | chr20q13.1-q13.2 |
| 202715_at | carbamoyl-phosphate synthetase 2, aspartate transcarbamylase, and dihydroorotase /// Rho guanine nucleotide exchange factor (GEF) 5 /// FLJ40722-like | CAD | NM_004341 | Hs.377010 | chr2p22-p21 |
| 202713_s_at | KIAA0391 | KIAA0391 | AA129755 | Hs.458487 | chr14q13.2 |
| 202710_at | BET1 homolog (S. cerevisiae) | BET1 | BC000899 | Hs.23103 | chr7q21.1-q22 |
| 202709_at | fibromodulin | FMOD | NM_002023 | Hs.442844 | chr1q32 |
| 202708_s_at | histone cluster 2, H2be | HIST2H2BE | NM_003528 | Hs.2178 | chr1q21-q23 |
| 202706_s_at | uridine monophosphate synthetase (orotate phosphoribosyl transferase and orotidine-5'-decarboxylase) | UMPS | D86227 | Hs.2057 | chr3q13 |
| 202703_at | dual specificity phosphatase 11 (RNA/RNP complex 1-interacting) | DUSP11 | NM_003584 | Hs.14611 | chr2p13.1 |
| 202702_at | tripartite motif-containing 26 | TRIM26 | NM_003449 | Hs.436871 | chr6p21.3 |
| 202696_at | oxidative-stress responsive 1 | OXSR1 | NM_005109 | Hs.95220 | chr3p22-p21.3 |
| 202695_s_at | serine/threonine kinase 17a (apoptosis-inducing) | STK17A | NM_004760 | Hs.9075 | chr7p12-p14 |
| 202691_at | small nuclear ribonucleoprotein D1 polypeptide 16kDa | SNRPD1 | NM_006938 | Hs.500995 | chr18q11.2 |
| 202686_s_at | AXL receptor tyrosine kinase | AXL | NM_021913 | Hs.83341 | chr19q13.1 |
| 202684_s_at | RNA (guanine-7-) methyltransferase | RNMT | AB020966 | Hs.8086 | chr18p11.22-p11.23 |
| 202682_s_at | ubiquitin specific peptidase 4 (proto-oncogene) | USP4 | NM_003363 | Hs.403828 | chr3p21.3 |
| 202680_at | general transcription factor IIE, polypeptide 2, beta 34kDa | GTF2E2 | NM_002095 | Hs.77100 | chr8p21-p12 |
| 202679_at | Niemann-Pick disease, type C1 | NPC1 | NM_000271 | Hs.404930 | chr18q11-q12 |
| 202678_at | general transcription factor IIA, 2, 12kDa | GTF2A2 | NM_004492 | Hs.279528 | chr15q22.2 |
| 202675_at | succinate dehydrogenase complex, subunit B, iron sulfur (Ip) | SDHB | NM_003000 | Hs.64 | chr1p36.1-p35 |
| 202673_at | dolichyl-phosphate mannosyltransferase polypeptide 1, catalytic subunit | DPM1 | NM_003859 | Hs.5085 | chr20q13.13 |
| 202670_at | mitogen-activated protein kinase kinase 1 | MAP2K1 | AI571419 | Hs.132311 | chr15q22.1-q22.33 |
| 202669_s_at | ephrin-B2 | EFNB2 | U16797 | Hs.30942 | chr13q33 |
| 202667_s_at | solute carrier family 39 (zinc transporter), member 7 | SLC39A7 | NM_006979 | Hs.278721 | chr6p21.3 |
| 202666_s_at | actin-like 6A | ACTL6A | NM_004301 | Hs.435326 | chr3q26.33 |
| 202664_at | WAS/WASL interacting protein family, member 1 | WIPF1 | AW058622 | Hs.401414 | chr2q31.1 |
| 202661_at | inositol 1,4,5-triphosphate receptor, type 2 | ITPR2 | AI963873 | Hs.406751 | chr12p11 |
| 202659_at | proteasome (prosome, macropain) subunit, beta type, 10 | PSMB10 | NM_002801 | Hs.9661 | chr16q22.1 |
| 202658_at | peroxisomal biogenesis factor 11B | PEX11B | NM_003846 | Hs.432876 | chr1q21.1 |
| 202656_s_at | SERTA domain containing 2 | SERTAD2 | BG107456 | Hs.77293 | chr2p14 |
| 202655_at | arginine-rich, mutated in early stage tumors | ARMET | NM_006010 | Hs.436446 | chr3p21.1 |
| 202650_s_at | KIAA0195 | KIAA0195 | NM_014738 | Hs.301132 | chr17q25.1 |
| 202649_x_at | ribosomal protein S19 | RPS19 | NM_001022 | Hs.381184 | chr19q13.2 |
| 202647_s_at | neuroblastoma RAS viral (v-ras) oncogene homolog | NRAS | NM_002524 | Hs.260523 | chr1p13.2 |
| 202646_s_at | cold shock domain containing E1, RNA-binding | CSDE1 | AA167775 | Hs.260523 | chr1p13.2 |
| 202645_s_at | multiple endocrine neoplasia I | MEN1 | NM_000244 | Hs.423348 | chr11q13 |
| 202643_s_at | tumor necrosis factor, alpha-induced protein 3 | TNFAIP3 | AI738896 | Hs.211600 | chr6q23 |
| 202642_s_at | transformation/transcription domain-associated protein | TRRAP | NM_003496 | Hs.203952 | chr7q21.2-q22.1 |
| 202641_at | ADP-ribosylation factor-like 3 | ARL3 | NM_004311 | Hs.182215 | chr10q23.3 |
| 202640_s_at | RAN binding protein 3 | RANBP3 | NM_003624 | Hs.176657 | chr19p13.3 |
| 202636_at | ring finger protein 103 | RNF103 | NM_005667 | Hs.147159 | chr2p11.2 |
| 202634_at | polymerase (RNA) II (DNA directed) polypeptide K, 7.0kDa | POLR2K | AL558030 | Hs.351475 | chr8q22.2 |
| 202633_at | topoisomerase (DNA) II binding protein 1 | TOPBP1 | NM_007027 | Hs.91417 | chr3q22.1 |
| 202629_at | amyloid beta precursor protein (cytoplasmic tail) binding protein 2 | APPBP2 | AV681579 | Hs.84084 | chr17q21-q23 |
| 202627_s_at | serpin peptidase inhibitor, clade E (nexin, plasminogen activator inhibitor type 1), member 1 | SERPINE1 | AL574210 | Hs.414795 | chr7q21.3-q22 |
| 202624_s_at | calcineurin binding protein 1 | CABIN1 | NM_012295 | Hs.435798 | chr22q11.23 |
| 202623_at | chromosome 14 open reading frame 11 | C14orf11 | NM_018453 | Hs.433269 | chr14q13.1 |
| 202622_s_at | ataxin 2 | ATXN2 | NM_002973 | Hs.76253 | chr12q24.1 |
| 202621_at | interferon regulatory factor 3 | IRF3 | NM_001571 | Hs.75254 | chr19q13.3-q13.4 |
| 202619_s_at | procollagen-lysine, 2-oxoglutarate 5-dioxygenase 2 | PLOD2 | AI754404 | Hs.41270 | chr3q23-q24 |
| 202616_s_at | methyl CpG binding protein 2 (Rett syndrome) | MECP2 | AI631140 | Hs.3239 | chrxq28 |
| 202615_at | Guanine nucleotide binding protein (G protein), q polypeptide | GNAQ | BF222895 | Hs.380144 | chr9q21 |
| 202614_at | solute carrier family 30 (zinc transporter), member 9 | SLC30A9 | NM_006345 | Hs.364615 | chr4p13-p12 |
| 202613_at | CTP synthase | CTPS | NM_001905 | Hs.251871 | chr1p34.1 |
| 202611_s_at | cofactor required for Sp1 transcriptional activation, subunit 2, 150kDa | CRSP2 | AI971089 | Hs.407604 | chrxp11.4-p11.2 |
| 202609_at | epidermal growth factor receptor pathway substrate 8 | EPS8 | NM_004447 | Hs.2132 | chr12q23-q24 |
| 202607_at | N-deacetylase/N-sulfotransferase (heparan glucosaminyl) 1 | NDST1 | AL526632 | Hs.20894 | chr5q33.1 |
| 202605_at | glucuronidase, beta | GUSB | NM_000181 | Hs.255230 | chr7q21.11 |
| 202602_s_at | HIV-1 Tat specific factor 1 | HTATSF1 | NM_014500 | Hs.204475 | chrxq26.1-q27.2 |
| 202599_s_at | nuclear receptor interacting protein 1 | NRIP1 | NM_003489 | Hs.155017 | chr21q11.2 |
| 202598_at | S100 calcium binding protein A13 | S100A13 | NM_005979 | Hs.446592 | chr1q21 |
| 202594_at | leptin receptor overlapping transcript-like 1 | LEPROTL1 | NM_015344 | Hs.146585 | chr8p21.2-p21.1 |
| 202593_s_at | membrane interacting protein of RGS16 | MIR16 | NM_016641 | Hs.107014 | chr16p12-p11.2 |
| 202592_at | biogenesis of lysosome-related organelles complex-1, subunit 1 | BLOC1S1 | NM_001487 | Hs.94672 | chr12q13-q14 |
| 202591_s_at | single-stranded DNA binding protein 1 | SSBP1 | NM_003143 | Hs.923 | chr7q34 |
| 202584_at | nuclear transcription factor, X-box binding 1 | NFX1 | AW291398 | Hs.413074 | chr9p13.3 |
| 202581_at | heat shock 70kDa protein 1B | HSPA1B | NM_005346 | Hs.274402 | chr6p21.3 |
| 202579_x_at | high mobility group nucleosomal binding domain 4 | HMGN4 | NM_006353 | Hs.236774 | chr6p21.3 |
| 202578_s_at | DEAD (Asp-Glu-Ala-As) box polypeptide 19A | DDX19A | NM_018332 | Hs.414126 | chr16q22.1 |
| 202576_s_at | DEAD (Asp-Glu-Ala-As) box polypeptide 19B /// DEAD (Asp-Glu-Ala-As) box polypeptide 19A | DDX19B | AL553254 | Hs.289097 | chr16q22.1 |
| 202573_at | casein kinase 1, gamma 2 | CSNK1G2 | AL530441 | Hs.181390 | chr19p13.3 |
| 202569_s_at | MAP/microtubule affinity-regulating kinase 3 | MARK3 | NM_002376 | Hs.437625 | chr14q32.3 |
| 202567_at | small nuclear ribonucleoprotein D3 polypeptide 18kDa | SNRPD3 | NM_004175 | Hs.356549 | chr22q11.23 |
| 202565_s_at | supervillin | SVIL | NM_003174 | Hs.163111 | chr10p11.2 |
| 202559_x_at | chromosome 1 open reading frame 77 | C1orf77 | AW005776 | Hs.323817 | chr1q21.3 |
| 202558_s_at | stress 70 protein chaperone, microsome-associated, 60kDa | STCH | NM_006948 | Hs.352341 | chr21q11 |
| 202556_s_at | microspherule protein 1 | MCRS1 | NM_006337 | Hs.25313 | chr12q13.12 |
| 202553_s_at | SYF2 homolog, RNA splicing factor (S. cerevisiae) | SYF2 | NM_015484 | Hs.20013 | chr1p36.11 |
| 202551_s_at | cysteine rich transmembrane BMP regulator 1 (chordin-like) | CRIM1 | BG546884 | Hs.444252 | chr2p21 |
| 202550_s_at | VAMP (vesicle-associated membrane protein)-associated protein B and C | VAPB | NM_004738 | Hs.182625 | chr20q13 |
| 202548_s_at | Rho guanine nucleotide exchange factor (GEF) 7 | ARHGEF7 | NM_003899 | Hs.172813 | chr13q34 |
| 202545_at | protein kinase C, delta | PRKCD | NM_006254 | Hs.155342 | chr3p21.31 |
| 202543_s_at | glia maturation factor, beta | GMFB | BC005359 | Hs.151413 | chr14q22.2 |
| 202541_at | small inducible cytokine subfamily E, member 1 (endothelial monocyte-activating) | SCYE1 | BF589679 | Hs.105656 | chr4q24 |
| 202539_s_at | 3-hydroxy-3-methylglutaryl-Coenzyme A reductase | HMGCR | AL518627 | Hs.11899 | chr5q13.3-q14 |
| 202538_s_at | chromatin modifying protein 2B | CHMP2B | NM_014043 | Hs.11449 | chr3p12.1 |
| 202535_at | Fas (TNFRSF6)-associated via death domain | FADD | NM_003824 | Hs.86131 | chr11q13.3 |
| 202532_s_at | dihydrofolate reductase | DHFR | BC000192 | Hs.83765 | chr5q11.2-q13.2 |
| 202531_at | interferon regulatory factor 1 | IRF1 | NM_002198 | Hs.80645 | chr5q31.1 |
| 202529_at | phosphoribosyl pyrophosphate synthetase-associated protein 1 | PRPSAP1 | NM_002766 | Hs.77498 | chr17q24-q25 |
| 202524_s_at | sparc/osteonectin, cwcv and kazal-like domains proteoglycan (testican) 2 | SPOCK2 | NM_014767 | Hs.436193 | chr10pter-q25.3 |
| 202521_at | CCCTC-binding factor (zinc finger protein) | CTCF | NM_006565 | Hs.411515 | chr16q21-q22.3 |
| 202520_s_at | mutL homolog 1, colon cancer, nonpolyposis type 2 (E. coli) | MLH1 | NM_000249 | Hs.433618 | chr3p21.3 |
| 202519_at | MLX interacting protein | MLXIP | NM_014938 | Hs.437153 | chr12q21.31 |
| 202518_at | B-cell CLL/lymphoma 7B | BCL7B | NM_001707 | Hs.408219 | chr7q11.23 |
| 202513_s_at | protein phosphatase 2, regulatory subunit B (B56), delta isoform | PPP2R5D | NM_006245 | Hs.118244 | chr6p21.1 |
| 202512_s_at | ATG5 autophagy related 5 homolog (S. cerevisiae) | ATG5 | NM_004849 | Hs.11171 | chr6q21 |
| 202505_at | small nuclear ribonucleoprotein polypeptide B'' | SNRPB2 | NM_003092 | Hs.280378 | chr20p12.2-p11.22 |
| 202502_at | acyl-Coenzyme A dehydrogenase, C-4 to C-12 straight chain | ACADM | NM_000016 | Hs.254593 | chr1p31 |
| 202500_at | DnaJ (Hsp40) homolog, subfamily B, member 2 | DNAJB2 | NM_006736 | Hs.77768 | chr2q32-q34 |
| 202496_at | enhancer of mRNA decapping 4 | EDC4 | NM_014329 | Hs.75682 | chr16q22.1 |
| 202495_at | tubulin folding cofactor C | TBCC | NM_003192 | Hs.75064 | chr6pter-p12.1 |
| 202494_at | peptidylprolyl isomerase E (cyclophilin E) | PPIE | NM_006112 | Hs.22516 | chr1p32 |
| 202492_at | ATG9 autophagy related 9 homolog A (S. cerevisiae) | ATG9A | NM_024085 | Hs.323363 | chr2q35 |
| 202490_at | inhibitor of kappa light polypeptide gene enhancer in B-cells, kinase complex-associated protein | IKBKAP | AF153419 | Hs.31323 | chr9q31 |
| 202484_s_at | methyl-CpG binding domain protein 2 | MBD2 | AF072242 | Hs.25674 | chr18q21 |
| 202483_s_at | RAN binding protein 1 | RANBP1 | NM_002882 | Hs.24763 | chr22q11.21 |
| 202481_at | dehydrogenase/reductase (SDR family) member 3 | DHRS3 | NM_004753 | Hs.125062 | chr1p36.1 |
| 202480_s_at | death effector domain containing | DEDD | NM_004216 | Hs.517342 | chr1q23.3 |
| 202479_s_at | tribbles homolog 2 (Drosophila) | TRIB2 | BC002637 | Hs.155418 | chr2p24.3 |
| 202475_at | transmembrane protein 147 | TMEM147 | NM_006326 | Hs.9234 | chr19q13.1 |
| 202474_s_at | host cell factor C1 (VP16-accessory protein) | HCFC1 | NM_005334 | Hs.83634 | chrxq28 |
| 202472_at | mannose phosphate isomerase | MPI | NM_002435 | Hs.75694 | chr15q22-qter |
| 202471_s_at | isocitrate dehydrogenase 3 (NAD+) gamma | IDH3G | NM_004135 | Hs.410197 | chrxq28 |
| 202468_s_at | catenin (cadherin-associated protein), alpha-like 1 | CTNNAL1 | NM_003798 | Hs.58488 | chr9q31.2 |
| 202466_at | polymerase (DNA directed) sigma | POLS | NM_006999 | Hs.39488 | chr5p15 |
| 202464_s_at | 6-phosphofructo-2-kinase/fructose-2,6-biphosphatase 3 | PFKFB3 | NM_004566 | Hs.195471 | chr10p14-p15 |
| 202461_at | eukaryotic translation initiation factor 2B, subunit 2 beta, 39kDa | EIF2B2 | NM_014239 | Hs.497429 | chr14q24.3 |
| 202459_s_at | lipin 2 | LPIN2 | U55968 | Hs.437425 | chr18p11.31 |
| 202454_s_at | v-erb-b2 erythroblastic leukemia viral oncogene homolog 3 (avian) | ERBB3 | NM_001982 | Hs.306251 | chr12q13 |
| 202453_s_at | general transcription factor IIH, polypeptide 1, 62kDa | GTF2H1 | NM_005316 | Hs.432593 | chr11p15.1-p14 |
| 202450_s_at | cathepsin K | CTSK | NM_000396 | Hs.83942 | chr1q21 |
| 202449_s_at | retinoid X receptor, alpha | RXRA | NM_002957 | Hs.20084 | chr9q34.3 |
| 202447_at | 2,4-dienoyl CoA reductase 1, mitochondrial | DECR1 | NM_001359 | Hs.414754 | chr8q21.3 |
| 202444_s_at | SPFH domain family, member 1 | SPFH1 | NM_006459 | Hs.285818 | chr10q21-q22 |
| 202442_at | adaptor-related protein complex 3, sigma 1 subunit | AP3S1 | NM_001284 | Hs.406191 | chr5q22 |
| 202440_s_at | suppression of tumorigenicity 5 | ST5 | NM_005418 | Hs.117715 | chr11p15 |
| 202438_x_at | iduronate 2-sulfatase (Hunter syndrome) | IDS | BF346014 | Hs.522169 | chrxq28 |
| 202433_at | solute carrier family 35, member B1 | SLC35B1 | NM_005827 | Hs.154073 | chr17q21.33 |
| 202432_at | protein phosphatase 3 (formerly 2B), catalytic subunit, beta isoform (calcineurin A beta) | PPP3CB | NM_021132 | Hs.187543 | chr10q21-q22 |
| 202431_s_at | v-myc myelocytomatosis viral oncogene homolog (avian) | MYC | NM_002467 | Hs.202453 | chr8q24.12-q24.13 |
| 202430_s_at | phospholipid scramblase 1 | PLSCR1 | NM_021105 | Hs.348478 | chr3q23 |
| 202428_x_at | diazepam binding inhibitor (GABA receptor modulator, acyl-Coenzyme A binding protein) | DBI | NM_020548 | Hs.78888 | chr2q12-q21 |
| 202427_s_at | brain protein 44 | BRP44 | NM_015415 | Hs.76285 | chr1q24 |
| 202423_at | MYST histone acetyltransferase (monocytic leukemia) 3 | MYST3 | NM_006766 | Hs.93231 | chr8p11 |
| 202422_s_at | acyl-CoA synthetase long-chain family member 4 | ACSL4 | NM_022977 | Hs.496560 | chrxq22.3-q23 |
| 202421_at | immunoglobulin superfamily, member 3 | IGSF3 | AB007935 | Hs.81234 | chr1p13 |
| 202419_at | follicular lymphoma variant translocation 1 | FVT1 | NM_002035 | Hs.74050 | chr18q21.3 |
| 202418_at | Yip1 interacting factor homolog A (S. cerevisiae) | YIF1A | NM_020470 | Hs.446445 | chr11q13 |
| 202417_at | kelch-like ECH-associated protein 1 | KEAP1 | NM_012289 | Hs.57729 | chr19p13.2 |
| 202416_at | DnaJ (Hsp40) homolog, subfamily C, member 7 | DNAJC7 | NM_003315 | Hs.446481 | chr17q11.2 |
| 202415_s_at | hsp70-interacting protein | HSPBP1 | NM_012267 | Hs.53066 | chr19q13.42 |
| 202414_at | excision repair cross-complementing rodent repair deficiency, complementation group 5 (xeroderma pigmentosum, complementation group G (Cockayne syndrome)) | ERCC5 | NM_000123 | Hs.258429 | chr13q22 |
| 202412_s_at | ubiquitin specific peptidase 1 | USP1 | AW499935 | Hs.35086 | chr1p32.1-p31.3 |
| 202411_at | interferon, alpha-inducible protein 27 | IFI27 | NM_005532 | Hs.278613 | chr14q32 |
| 202409_at | insulin-like growth factor 2 (somatomedin A) /// insulin- insulin-like growth factor 2 | IGF2 | X07868 | Hs.251664 | chr11p15.5 |
| 202407_s_at | PRP31 pre-mRNA processing factor 31 homolog (S. cerevisiae) | PRPF31 | BF342707 | Hs.312927 | chr19q13.42 |
| 202405_at | TIA1 cytotoxic granule-associated RNA binding protein-like 1 | TIAL1 | BF432532 | Hs.64840 | chr10q |
| 202403_s_at | collagen, type I, alpha 2 | COL1A2 | AA788711 | Hs.232115 | chr7q22.1 |
| 202402_s_at | cysteinyl-tRNA synthetase | CARS | NM_001751 | Hs.304134 | chr11p15.5 |
| 202401_s_at | serum response factor (c-fos serum response element-binding transcription factor) | SRF | NM_003131 | Hs.444086 | chr6p21.1 |
| 202398_at | adaptor-related protein complex 3, sigma 2 subunit | AP3S2 | BC002785 | Hs.19720 | chr15q26.1 |
| 202396_at | transcription elongation regulator 1 | TCERG1 | NM_006706 | Hs.300052 | chr5q31 |
| 202395_at | N-ethylmaleimide-sensitive factor | NSF | NM_006178 | Hs.431279 | chr17q21 |
| 202394_s_at | ATP-binding cassette, sub-family F (GCN20), member 3 | ABCF3 | NM_018358 | Hs.361323 | chr3q27.1 |
| 202393_s_at | Kruppel-like factor 10 | KLF10 | NM_005655 | Hs.82173 | chr8q22.2 |
| 202392_s_at | phosphatidylserine decarboxylase | PISD | NM_014338 | Hs.420559 | chr22q12.2 |
| 202391_at | brain abundant, membrane attached signal protein 1 | BASP1 | NM_006317 | Hs.511745 | chr5p15.1-p14 |
| 202389_s_at | huntingtin (Huntington disease) | HD | L12392 | Hs.437162 | chr4p16.3 |
| 202388_at | regulator of G-protein signalling 2, 24kDa | RGS2 | NM_002923 | Hs.78944 | chr1q31 |
| 202387_at | BCL2-associated athanogene /// BCL2-associated athanogene | BAG1 | NM_004323 | Hs.377484 | chr9p12 |
| 202386_s_at | KIAA0430 | KIAA0430 | NM_019081 | Hs.432741 | chr16p13.11 |
| 202385_s_at | Treacher Collins-Franceschetti syndrome 1 | TCOF1 | NM_000356 | Hs.301266 | chr5q32-q33.1 |
| 202382_s_at | glucosamine-6-phosphate deaminase 1 | GNPDA1 | NM_005471 | Hs.278500 | chr5q21 |
| 202380_s_at | natural killer-tumor recognition sequence | NKTR | NM_005385 | Hs.369815 | chr3p23-p21 |
| 202378_s_at | leptin receptor overlapping transcript | LEPROT | NM_017526 |  | chr1p31.2 |
| 202376_at | serpin peptidase inhibitor, clade A (alpha-1 antiproteinase, antitrypsin), member 3 | SERPINA3 | NM_001085 | Hs.388547 | chr14q32.1 |
| 202375_at | SEC24 related gene family, member D (S. cerevisiae) | SEC24D | NM_014822 | Hs.409424 | chr4q26 |
| 202374_s_at | RAB3 GTPase activating protein subunit 2 (non-catalytic) | RAB3GAP2 | NM_012414 | Hs.497803 | chr1q41 |
| 202371_at | transcription elongation factor A (SII)-like 4 | TCEAL4 | NM_024863 | Hs.194329 | chrxq22.2 |
| 202368_s_at | translocation associated membrane protein 2 | TRAM2 | AI986461 | Hs.310230 | chr6p21.1-p12 |
| 202367_at | cut-like 1, CCAAT displacement protein (Drosophila) | CUTL1 | NM_001913 | Hs.438974 | chr7q22.1 |
| 202366_at | acyl-Coenzyme A dehydrogenase, C-2 to C-3 short chain | ACADS | NM_000017 | Hs.507076 | chr12q22-qter |
| 202365_at | hypothetical protein MGC5139 | MGC5139 | BC004815 | Hs.127610 | chr12q24.31 |
| 202364_at | MAX interactor 1 /// MAX interactor 1 | MXI1 | NM_005962 | Hs.118630 | chr10q24-q25 |
| 202361_at | SEC24 related gene family, member C (S. cerevisiae) | SEC24C | NM_004922 | Hs.81964 | chr10q22.2 |
| 202360_at | mastermind-like 1 (Drosophila) | MAML1 | NM_014757 | Hs.484349 | chr5q35 |
| 202358_s_at | sorting nexin 19 | SNX19 | BG434168 |  | chr11q25 |
| 202354_s_at | general transcription factor IIF, polypeptide 1, 74kDa | GTF2F1 | AW190445 | Hs.68257 | chr19p13.3 |
| 202352_s_at | proteasome (prosome, macropain) 26S subunit, non-ATPase, 12 | PSMD12 | AI446530 | Hs.4295 | chr17q24.2 |
| 202350_s_at | matrilin 2 | MATN2 | NM_002380 | Hs.153647 | chr8q22 |
| 202349_at | torsin family 1, member A (torsin A) | TOR1A | NM_000113 | Hs.534322 | chr9q34 |
| 202346_at | huntingtin interacting protein 2 | HIP2 | NM_005339 | Hs.246603 | chr4p14 |
| 202345_s_at | fatty acid binding protein 5 (psoriasis-associated) /// similar to Fatty acid-binding protein, epidermal (E-FABP) (Psoriasis-associated fatty acid-binding protein homolog) (PA-FABP) /// similar to Fatty acid-binding protein, epidermal (E-FABP) (Psoriasis- | FABP5 | NM_001444 | Hs.408061 | chr8q21.13 |
| 202344_at | heat shock transcription factor 1 | HSF1 | NM_005526 | Hs.132625 | chr8q24.3 |
| 202343_x_at | cytochrome c oxidase subunit Vb | COX5B | NM_001862 | Hs.1342 | chr2cen-q13 |
| 202341_s_at | tripartite motif-containing 2 | TRIM2 | AA149745 | Hs.435734 | chr4q31.3 |
| 202339_at | symplekin | SYMPK | NM_004819 | Hs.378736 | chr19q13.3 |
| 202337_at | polyamine-modulated factor 1 | PMF1 | NM_007221 | Hs.408848 | chr1q12 |
| 202335_s_at | ubiquitin-conjugating enzyme E2B (RAD6 homolog) | UBE2B | NM_003337 |  |  |
| 202332_at | casein kinase 1, epsilon | CSNK1E | NM_001894 | Hs.355669 | chr22q13.1 |
| 202331_at | branched chain keto acid dehydrogenase E1, alpha polypeptide | BCKDHA | NM_000709 |  |  |
| 202330_s_at | uracil-DNA glycosylase | UNG | NM_003362 | Hs.78853 | chr12q23-q24.1 |
| 202329_at | c-src tyrosine kinase | CSK | NM_004383 | Hs.77793 | chr15q23-q25 |
| 202328_s_at | polycystic kidney disease 1 (autosomal dominant) | PKD1 | NM_000296 | Hs.75813 | chr16p13.3 |
| 202325_s_at | ATP synthase, H+ transporting, mitochondrial F0 complex, subunit F6 | ATP5J | NM_001685 | Hs.246310 | chr21q21.1 |
| 202323_s_at | acyl-Coenzyme A binding domain containing 3 | ACBD3 | AI636775 | Hs.6831 | chr1q42.12 |
| 202321_at | geranylgeranyl diphosphate synthase 1 | GGPS1 | AW299507 | Hs.55498 | chr1q43 |
| 202320_at | general transcription factor IIIC, polypeptide 1, alpha 220kDa | GTF3C1 | NM_001520 | Hs.331 | chr16p12 |
| 202317_s_at | ubiquitination factor E4B (UFD2 homolog, yeast) | UBE4B | NM_006048 | Hs.386404 | chr1p36.3 |
| 202315_s_at | breakpoint cluster region | BCR | NM_004327 | Hs.446394 | chr22q11.23 |
| 202314_at | cytochrome P450, family 51, subfamily A, polypeptide 1 | CYP51A1 | NM_000786 | Hs.417077 | chr7q21.2-q21.3 |
| 202309_at | methylenetetrahydrofolate dehydrogenase (NADP+ dependent) 1, methenyltetrahydrofolate cyclohydrolase, formyltetrahydrofolate synthetase | MTHFD1 | NM_005956 | Hs.509628 | chr14q24 |
| 202308_at | sterol regulatory element binding transcription factor 1 | SREBF1 | NM_004176 | Hs.190284 | chr17p11.2 |
| 202307_s_at | transporter 1, ATP-binding cassette, sub-family B (MDR/TAP) | TAP1 | NM_000593 | Hs.352018 | chr6p21.3 |
| 202306_at | polymerase (RNA) II (DNA directed) polypeptide G | POLR2G | NM_002696 | Hs.14839 | chr11q13.1 |
| 202305_s_at | fasciculation and elongation protein zeta 2 (zygin II) | FEZ2 | AI685892 | Hs.258563 | chr2p21 |
| 202301_s_at | similar to splicing factor, arginine/serine-rich 4 | FLJ11021 | BE396879 | Hs.432996 | chr12q24.31 |
| 202299_s_at | hepatitis B virus x interacting protein | HBXIP | NM_006402 | Hs.367886 | chr1p13.3 |
| 202298_at | NADH dehydrogenase (ubiquinone) 1 alpha subcomplex, 1, 7.5kDa | NDUFA1 | NM_004541 | Hs.74823 | chrxq24 |
| 202295_s_at | cathepsin H | CTSH | NM_004390 | Hs.114931 | chr15q24-q25 |
| 202293_at | stromal antigen 1 | STAG1 | AW168948 | Hs.444211 | chr3q22.3 |
| 202290_at | PDGFA associated protein 1 | PDAP1 | NM_014891 | Hs.278426 | chr7q22.1 |
| 202288_at | FK506 binding protein 12-rapamycin associated protein 1 | FRAP1 | U88966 | Hs.338207 | chr1p36.2 |
| 202284_s_at | cyclin-dependent kinase inhibitor 1A (p21, Cip1) | CDKN1A | NM_000389 | Hs.370771 | chr6p21.2 |
| 202282_at | hydroxysteroid (17-beta) dehydrogenase 10 | HSD17B10 | NM_004493 | Hs.171280 | chrxp11.2 |
| 202281_at | cyclin G associated kinase | GAK | NM_005255 | Hs.153227 | chr4p16 |
| 202279_at | chromosome 14 open reading frame 2 | C14orf2 | NM_004894 | Hs.109052 | chr14q32.33 |
| 202276_at | split hand/foot malformation (ectrodactyly) type 1 | SHFM1 | NM_006304 | Hs.333495 | chr7q21.3-q22.1 |
| 202275_at | glucose-6-phosphate dehydrogenase | G6PD | NM_000402 | Hs.80206 | chrxq28 |
| 202274_at | actin, gamma 2, smooth muscle, enteric | ACTG2 | NM_001615 | Hs.403989 | chr2p13.1 |
| 202271_at | F-box protein 28 | FBXO28 | AB007952 |  |  |
| 202268_s_at | amyloid beta precursor protein binding protein 1 | APPBP1 | NM_003905 | Hs.418162 | chr16q22 |
| 202266_at | TRAF and TNF receptor associated protein | TTRAP | NM_016614 | Hs.210628 | chr6p22.3-p22.1 |
| 202265_at | B lymphoma Mo-MLV insertion region (mouse) | BMI1 | NM_005180 | Hs.380403 | chr10p11.23 |
| 202264_s_at | translocase of outer mitochondrial membrane 40 homolog (yeast) | TOMM40 | NM_006114 | Hs.310542 | chr19q13 |
| 202263_at | cytochrome b5 reductase 1 | CYB5R1 | NM_016243 | Hs.334832 | chr1p36.13-q41 |
| 202261_at | vacuolar protein sorting 72 homolog (S. cerevisiae) | VPS72 | NM_005997 | Hs.2430 | chr1q21 |
| 202260_s_at | syntaxin binding protein 1 | STXBP1 | NM_003165 | Hs.325862 | chr9q34.1 |
| 202257_s_at | CD2 (cytoplasmic tail) binding protein 2 | CD2BP2 | NM_006110 | Hs.202677 | chr16p11.2 |
| 202255_s_at | signal-induced proliferation-associated 1 like 1 | SIPA1L1 | NM_015556 | Hs.172180 | chr14q24.2 |
| 202253_s_at | dynamin 2 | DNM2 | NM_004945 | Hs.432832 | chr19p13.2 |
| 202252_at | RAB13, member RAS oncogene family | RAB13 | NM_002870 | Hs.151536 | chr1q21.2 |
| 202251_at | PRP3 pre-mRNA processing factor 3 homolog (S. cerevisiae) | PRPF3 | NM_004698 | Hs.11776 | chr1q21.1 |
| 202250_s_at | WD repeat domain 42A | WDR42A | NM_015726 | Hs.120904 | chr1q22-q23 |
| 202246_s_at | cyclin-dependent kinase 4 | CDK4 | NM_000075 | Hs.95577 | chr12q14 |
| 202242_at | tetraspanin 7 | TSPAN7 | NM_004615 | Hs.439586 | chrxp11.4 |
| 202241_at | tribbles homolog 1 (Drosophila) | TRIB1 | NM_025195 | Hs.444947 | chr8q24.13 |
| 202239_at | poly (ADP-ribose) polymerase family, member 4 | PARP4 | NM_006437 | Hs.437959 | chr13q11 |
| 202238_s_at | nicotinamide N-methyltransferase | NNMT | NM_006169 | Hs.364345 | chr11q23.1 |
| 202235_at | solute carrier family 16, member 1 (monocarboxylic acid transporter 1) | SLC16A1 | BF511091 | Hs.75231 | chr1p12 |
| 202233_s_at | ubiquinol-cytochrome c reductase hinge protein | UQCRH | NM_006004 | Hs.285761 | chr1p34.1 |
| 202231_at | PCI domain containing 1 (herpesvirus entry mediator) | PCID1 | NM_006360 | Hs.44893 | chr11p13 |
| 202230_s_at | calcium homeostasis endoplasmic reticulum protein | CHERP | NM_006387 | Hs.386465 | chr19p13.1 |
| 202227_s_at | bromodomain containing 8 | BRD8 | NM_006696 | Hs.468207 | chr5q31 |
| 202226_s_at | v-crk sarcoma virus CT10 oncogene homolog (avian) | CRK | NM_016823 | Hs.343220 | chr17p13.3 |
| 202223_at | STT3, subunit of the oligosaccharyltransferase complex, homolog A (S. cerevisiae) | STT3A | NM_002219 | Hs.287850 | chr11q23.3 |
| 202221_s_at | E1A binding protein p300 | EP300 | AV727101 | Hs.271730 | chr22q13.2 |
| 202220_at | KIAA0907 | KIAA0907 | NM_014949 | Hs.24656 | chr1q22 |
| 202218_s_at | fatty acid desaturase 2 | FADS2 | NM_004265 | Hs.388164 | chr11q12-q13.1 |
| 202213_s_at | cullin 4B | CUL4B | AI650819 | Hs.155976 | chrxq23 |
| 202212_at | pescadillo homolog 1, containing BRCT domain (zebrafish) | PES1 | NM_014303 | Hs.132037 | chr22q12.1 |
| 202211_at | ADP-ribosylation factor GTPase activating protein 3 | ARFGAP3 | BC005122 | Hs.13014 | chr22q13.2-q13.3 |
| 202209_at | LSM3 homolog, U6 small nuclear RNA associated (S. cerevisiae) | LSM3 | NM_014463 | Hs.111632 | chr3p25.1 |
| 202205_at | vasodilator-stimulated phosphoprotein | VASP | NM_003370 | Hs.403914 | chr19q13.2-q13.3 |
| 202204_s_at | autocrine motility factor receptor | AMFR | AF124145 | Hs.295137 | chr16q21 |
| 202202_s_at | laminin, alpha 4 | LAMA4 | NM_002290 | Hs.437536 | chr6q21 |
| 202201_at | biliverdin reductase B (flavin reductase (NADPH)) | BLVRB | NM_000713 | Hs.76289 | chr19q13.1-q13.2 |
| 202199_s_at | SFRS protein kinase 1 | SRPK1 | AW082913 | Hs.369358 | chr6p21.3-p21.2 |
| 202197_at | myotubularin related protein 3 | MTMR3 | NM_021090 | Hs.412833 | chr22q12.2 |
| 202191_s_at | growth arrest-specific 7 | GAS7 | BE439987 | Hs.462214 | chr17p13.1 |
| 202188_at | nucleoporin 93kDa | NUP93 | NM_014669 | Hs.295014 | chr16q13 |
| 202187_s_at | protein phosphatase 2, regulatory subunit B (B56), alpha isoform | PPP2R5A | NM_006243 | Hs.155079 | chr1q32.2-q32.3 |
| 202185_at | procollagen-lysine, 2-oxoglutarate 5-dioxygenase 3 | PLOD3 | NM_001084 | Hs.153357 | chr7q22 |
| 202184_s_at | nucleoporin 133kDa | NUP133 | NM_018230 | Hs.12457 | chr1q42.13 |
| 202181_at | KIAA0247 | KIAA0247 | NM_014734 | Hs.82426 | chr14q24.1 |
| 202179_at | bleomycin hydrolase | BLMH | NM_000386 | Hs.387183 | chr17q11.2 |
| 202176_at | excision repair cross-complementing rodent repair deficiency, complementation group 3 (xeroderma pigmentosum group B complementing) | ERCC3 | NM_000122 | Hs.77929 | chr2q21 |
| 202175_at | chondroitin polymerizing factor | CHPF | NM_024536 | Hs.458374 | chr2q35 |
| 202171_at | vascular endothelial zinc finger 1 | VEZF1 | AU146275 | Hs.414455 | chr17q23.2 |
| 202169_s_at | aminoadipate-semialdehyde dehydrogenase-phosphopantetheinyl transferase | AASDHPPT | AF302110 | Hs.64595 | chr11q22 |
| 202168_at | TAF9 RNA polymerase II, TATA box binding protein (TBP)-associated factor, 32kDa | TAF9 | NM_003187 | Hs.60679 | chr5q11.2-q13.1 |
| 202167_s_at | MMS19-like (MET18 homolog, S. cerevisiae) | MMS19L | NM_022362 | Hs.288891 | chr10q24-q25 |
| 202165_at | protein phosphatase 1, regulatory (inhibitor) subunit 2 | PPP1R2 | BF966540 | Hs.442946 | chr3q29 |
| 202162_s_at | CCR4-NOT transcription complex, subunit 8 | CNOT8 | AI769416 | Hs.26703 | chr5q31-q33 |
| 202161_at | protein kinase N1 | PKN1 | NM_002741 | Hs.2499 | chr19p13.1-p12 |
| 202160_at | CREB binding protein (Rubinstein-Taybi syndrome) | CREBBP | NM_004380 | Hs.270804 | chr16p13.3 |
| 202159_at | phenylalanine-tRNA synthetase-like, alpha subunit | FARSLA | NM_004461 | Hs.23111 | chr19p13.2 |
| 202156_s_at | CUG triplet repeat, RNA binding protein 2 | CUGBP2 | N36839 | Hs.211610 | chr10p13 |
| 202155_s_at | nucleoporin 214kDa | NUP214 | NM_005085 | Hs.460294 | chr9q34.1 |
| 202154_x_at | tubulin, beta 3 | TUBB3 | NM_006086 | Hs.511743 | chr16q24.3 |
| 202153_s_at | nucleoporin 62kDa | NUP62 | NM_016553 | Hs.437023 | chr19q13.33 |
| 202150_s_at | neural precursor cell expressed, developmentally down-regulated 9 | NEDD9 | U64317 | Hs.388589 | chr6p25-p24 |
| 202148_s_at | pyrroline-5-carboxylate reductase 1 | PYCR1 | NM_006907 | Hs.458332 | chr17q25.3 |
| 202147_s_at | interferon-related developmental regulator 1 | IFRD1 | NM_001550 | Hs.7879 | chr7q22-q31 |
| 202145_at | lymphocyte antigen 6 complex, locus E | LY6E | NM_002346 | Hs.77667 | chr8q24.3 |
| 202144_s_at | adenylosuccinate lyase | ADSL | NM_000026 | Hs.75527 | chr22q13.2 |
| 202140_s_at | CDC-like kinase 3 | CLK3 | NM_003992 | Hs.73987 | chr15q24 |
| 202137_s_at | zinc finger, MYND domain containing 11 | ZMYND11 | NM_006624 | Hs.145894 | chr10p14 |
| 202135_s_at | ARP1 actin-related protein 1 homolog B, centractin beta (yeast) | ACTR1B | NM_005735 | Hs.2477 | chr2q11.1-q11.2 |
| 202132_at | WW domain containing transcription regulator 1 | WWTR1 | AA081084 | Hs.24341 | chr3q23-q24 |
| 202129_s_at | RIO kinase 3 (yeast) /// RIO kinase 3 (yeast) | RIOK3 | AW006290 | Hs.209061 | chr18q11.2 |
| 202128_at | KIAA0317 | KIAA0317 | NM_014821 | Hs.200628 | chr14q24.3 |
| 202124_s_at | trafficking protein, kinesin binding 2 | TRAK2 | AV705253 | Hs.154248 | chr2q33 |
| 202123_s_at | v-abl Abelson murine leukemia viral oncogene homolog 1 | ABL1 | NM_005157 | Hs.446504 | chr9q34.1 |
| 202122_s_at | mannose-6-phosphate receptor binding protein 1 | M6PRBP1 | NM_005817 | Hs.140452 | chr19p13.3 |
| 202121_s_at | chromatin modifying protein 2A | CHMP2A | NM_014453 | Hs.12107 | chr19q |
| 202120_x_at | adaptor-related protein complex 2, sigma 1 subunit | AP2S1 | NM_004069 | Hs.119591 | chr19q13.2-q13.3 |
| 202118_s_at | copine III | CPNE3 | AA541758 | Hs.14158 | chr8q21.3 |
| 202117_at | Rho GTPase activating protein 1 | ARHGAP1 | BG468434 | Hs.138860 | chr11p12-q12 |
| 202116_at | D4, zinc and double PHD fingers family 2 | DPF2 | NM_006268 | Hs.13495 | chr11q13 |
| 202115_s_at | nucleolar complex associated 2 homolog (S. cerevisiae) | NOC2L | NM_015658 | Hs.335293 | chr1p36.33 |
| 202113_s_at | sorting nexin 2 | SNX2 | AF043453 | Hs.11183 | chr5q23 |
| 202111_at | solute carrier family 4, anion exchanger, member 2 (erythrocyte membrane protein band 3-like 1) | SLC4A2 | NM_003040 | Hs.433984 | chr7q35-q36 |
| 202110_at | cytochrome c oxidase subunit VIIb | COX7B | NM_001866 | Hs.432170 | chrxq21.1 |
| 202109_at | ADP-ribosylation factor interacting protein 2 (arfaptin 2) | ARFIP2 | NM_012402 | Hs.75139 | chr11p15 |
| 202108_at | peptidase D | PEPD | NM_000285 | Hs.444207 | chr19q12-q13.2 |
| 202105_at | immunoglobulin (CD79A) binding protein 1 | IGBP1 | NM_001551 | Hs.3631 | chrxq13.1-q13.3 |
| 202104_s_at | spastic paraplegia 7, paraplegin (pure and complicated autosomal recessive) | SPG7 | NM_003119 | Hs.311765 | chr16q24.3 |
| 202103_at | bromodomain containing 4 | BRD4 | AI991631 | Hs.278675 | chr19p13.1 |
| 202101_s_at | v-ral simian leukemia viral oncogene homolog B (ras related; GTP binding protein) | RALB | NM_002881 | Hs.348024 | chr2cen-q13 |
| 202097_at | nucleoporin 153kDa | NUP153 | NM_005124 | Hs.146449 | chr6p22.3 |
| 202096_s_at | translocator protein (18kDa) | TSPO | NM_000714 | Hs.202 | chr22q13.31 |
| 202093_s_at | Paf1, RNA polymerase II associated factor, homolog (S. cerevisiae) | PAF1 | NM_019088 | Hs.152894 | chr19q13.1 |
| 202092_s_at | ADP-ribosylation factor-like 2 binding protein | ARL2BP | BF244411 | Hs.9552 | chr16q13 |
| 202090_s_at | ubiquinol-cytochrome c reductase, 6.4kDa subunit /// transmembrane protein 54 | UQCR | NM_006830 | Hs.8372 | chr19p13.3 |
| 202089_s_at | solute carrier family 39 (zinc transporter), member 6 | SLC39A6 | NM_012319 | Hs.79136 | chr18q12.2 |
| 202087_s_at | cathepsin L | CTSL | NM_001912 | Hs.418123 | chr9q21-q22 |
| 202085_at | tight junction protein 2 (zona occludens 2) | TJP2 | NM_004817 | Hs.75608 | chr9q13-q21 |
| 202084_s_at | SEC14-like 1 (S. cerevisiae) | SEC14L1 | NM_003003 | Hs.75232 | chr17q25.1-17q25.2 |
| 202081_at | immediate early response 2 | IER2 | NM_004907 | Hs.737 | chr19p13.13 |
| 202080_s_at | trafficking protein, kinesin binding 1 | TRAK1 | NM_014965 | Hs.534438 | chr3p25.3-p24.1 |
| 202078_at | COP9 constitutive photomorphogenic homolog subunit 3 (Arabidopsis) | COPS3 | NM_003653 | Hs.6076 | chr17p11.2 |
| 202077_at | NADH dehydrogenase (ubiquinone) 1, alpha/beta subcomplex, 1, 8kDa | NDUFAB1 | NM_005003 | Hs.5556 | chr16p12.1 |
| 202076_at | baculoviral IAP repeat-containing 2 | BIRC2 | NM_001166 | Hs.289107 | chr11q22 |
| 202075_s_at | integrin, alpha V (vitronectin receptor, alpha polypeptide, antigen CD51) /// phospholipid transfer protein | ITGAV | NM_006227 | Hs.439312 | chr20q12-q13.1 |
| 202073_at | optineurin | OPTN | AV757675 | Hs.390162 | chr10p13 |
| 202072_at | heterogeneous nuclear ribonucleoprotein L | HNRPL | NM_001533 | Hs.446623 | chr19q13.2 |
| 202071_at | syndecan 4 (amphiglycan, ryudocan) | SDC4 | NM_002999 | Hs.252189 | chr20q12 |
| 202070_s_at | isocitrate dehydrogenase 3 (NAD+) alpha | IDH3A | NM_005530 | Hs.277543 | chr15q25.1-q25.2 |
| 202066_at | protein tyrosine phosphatase, receptor type, f polypeptide (PTPRF), interacting protein (liprin), alpha 1 | PPFIA1 | AA195259 | Hs.503095 | chr11q13.3 |
| 202064_s_at | sel-1 suppressor of lin-12-like (C. elegans) | SEL1L | AF052059 | Hs.181300 | chr14q24.3-q31 |
| 202058_s_at | karyopherin alpha 1 (importin alpha 5) | KPNA1 | BC002374 | Hs.161008 | chr3q21 |
| 202052_s_at | retinoic acid induced 14 | RAI14 | NM_015577 | Hs.368605 | chr5p13.3-p13.2 |
| 202049_s_at | zinc finger, MYM-type 4 | ZMYM4 | AA521508 | Hs.150390 | chr1p32-p34 |
| 202047_s_at | chromobox homolog 6 | CBX6 | AI458128 | Hs.511952 | chr22q13.1 |
| 202043_s_at | spermine synthase | SMS | NM_004595 | Hs.449032 | chrxp22.1 |
| 202042_at | histidyl-tRNA synthetase | HARS | NM_002109 | Hs.77798 | chr5q31.3 |
| 202041_s_at | fibroblast growth factor (acidic) intracellular binding protein | FIBP | NM_004214 | Hs.7768 | chr11q13.1 |
| 202038_at | ubiquitination factor E4A (UFD2 homolog, yeast) | UBE4A | NM_004788 | Hs.75275 | chr11q23.3 |
| 202036_s_at | secreted frizzled-related protein 1 | SFRP1 | AF017987 | Hs.7306 | chr8p12-p11.1 |
| 202033_s_at | RB1-inducible coiled-coil 1 | RB1CC1 | BG402105 | Hs.151202 | chr8p22-q21.13 |
| 202032_s_at | mannosidase, alpha, class 2A, member 2 | MAN2A2 | NM_006122 | Hs.116459 | chr15q26.1 |
| 202030_at | branched chain ketoacid dehydrogenase kinase | BCKDK | NM_005881 | Hs.20644 | chr16p11.2 |
| 202028_s_at | ribosomal protein L38 | RPL38 | BC000603 |  |  |
| 202027_at | chromosome 22 open reading frame 5 | C22orf5 | NM_012264 | Hs.182626 | chr22q12 |
| 202026_at | succinate dehydrogenase complex, subunit D, integral membrane protein | SDHD | NM_003002 | Hs.356270 | chr11q23 |
| 202025_x_at | acetyl-Coenzyme A acyltransferase 1 (peroxisomal 3-oxoacyl-Coenzyme A thiolase) | ACAA1 | NM_001607 | Hs.166160 | chr3p23-p22 |
| 202024_at | arsA arsenite transporter, ATP-binding, homolog 1 (bacterial) | ASNA1 | NM_004317 | Hs.165439 | chr19q13.3 |
| 202023_at | ephrin-A1 | EFNA1 | NM_004428 | Hs.399713 | chr1q21-q22 |
| 202022_at | aldolase C, fructose-bisphosphate | ALDOC | NM_005165 | Hs.155247 | chr17cen-q12 |
| 202020_s_at | LanC lantibiotic synthetase component C-like 1 (bacterial) | LANCL1 | NM_006055 | Hs.13351 | chr2q33-q35 |
| 202017_at | epoxide hydrolase 1, microsomal (xenobiotic) | EPHX1 | NM_000120 | Hs.89649 | chr1q42.1 |
| 202014_at | protein phosphatase 1, regulatory (inhibitor) subunit 15A | PPP1R15A | NM_014330 | Hs.76556 | chr19q13.2 |
| 202013_s_at | exostoses (multiple) 2 | EXT2 | NM_000401 | Hs.75334 | chr11p12-p11 |
| 202010_s_at | zinc finger protein 410 | ZNF410 | NM_021188 | Hs.405945 | chr14q24.3 |
| 202009_at | twinfilin, actin-binding protein, homolog 2 (Drosophila) | TWF2 | NM_007284 | Hs.436439 | chr3p21.1 |
| 202007_at | nidogen 1 | NID1 | BF940043 | Hs.356624 | chr1q43 |
| 202006_at | protein tyrosine phosphatase, non-receptor type 12 | PTPN12 | NM_002835 | Hs.62 | chr7q11.23 |
| 202003_s_at | acetyl-Coenzyme A acyltransferase 2 (mitochondrial 3-oxoacyl-Coenzyme A thiolase) | ACAA2 | NM_006111 | Hs.172506 | chr18q21.1 |
| 202000_at | NADH dehydrogenase (ubiquinone) 1 alpha subcomplex, 6, 14kDa | NDUFA6 | BC002772 | Hs.274416 | chr22q13.2-q13.31 |
| 201999_s_at | dynein, light chain, Tctex-type 1 | DYNLT1 | NM_006519 | Hs.266940 | chr6q25.2-q25.3 |
| 201992_s_at | kinesin family member 5B | KIF5B | NM_004521 | Hs.149436 | chr10pter-q22.1 |
| 201988_s_at | cAMP responsive element binding protein-like 2 | CREBL2 | BF438056 | Hs.13313 | chr12p13 |
| 201985_at | KIAA0196 | KIAA0196 | NM_014846 | Hs.437991 | chr8p22 |
| 201980_s_at | Ras suppressor protein 1 | RSU1 | NM_012425 | Hs.351342 | chr10p13 |
| 201976_s_at | myosin X | MYO10 | NM_012334 | Hs.61638 | chr5p15.1-p14.3 |
| 201973_s_at | chromosome 7 open reading frame 28A | C7orf28A | AL550875 | Hs.289112 | chr7p22.1 |
| 201971_s_at | ATPase, H+ transporting, lysosomal 70kDa, V1 subunit A | ATP6V1A | NM_001690 | Hs.409131 | chr3q13.2-q13.31 |
| 201969_at | nuclear autoantigenic sperm protein (histone-binding) | NASP | AW003362 | Hs.446206 | chr1p34.1 |
| 201968_s_at | phosphoglucomutase 1 | PGM1 | NM_002633 | Hs.1869 | chr1p31 |
| 201966_at | NADH dehydrogenase (ubiquinone) Fe-S protein 2, 49kDa (NADH-coenzyme Q reductase) | NDUFS2 | NM_004550 | Hs.173611 | chr1q23 |
| 201961_s_at | ring finger protein 41 | RNF41 | AL583171 | Hs.436596 | chr12q13.2-q13.3 |
| 201956_s_at | glyceronephosphate O-acyltransferase | GNPAT | NM_014236 | Hs.12482 | chr1q42.11-42.3 |
| 201955_at | cyclin C | CCNC | AL137784 | Hs.435450 | chr6q21 |
| 201954_at | actin related protein 2/3 complex, subunit 1B, 41kDa /// similar to Actin-related protein 2/3 complex subunit 1B (ARP2/3 complex 41 kDa subunit) (p41-ARC) | ARPC1B | NM_005720 | Hs.433506 | chr7q22.1 |
| 201953_at | calcium and integrin binding 1 (calmyrin) | CIB1 | NM_006384 | Hs.135471 | chr15q25.3-q26 |
| 201951_at | activated leukocyte cell adhesion molecule | ALCAM | BF242905 | Hs.434858 | chr3q13.1 |
| 201949_x_at | capping protein (actin filament) muscle Z-line, beta | CAPZB | AL572341 | Hs.333417 | chr1p36.1 |
| 201948_at | guanine nucleotide binding protein-like 2 (nucleolar) | GNL2 | NM_013285 | Hs.75528 | chr1p34.3 |
| 201946_s_at | chaperonin containing TCP1, subunit 2 (beta) | CCT2 | AL545982 | Hs.189772 | chr12q15 |
| 201944_at | hexosaminidase B (beta polypeptide) | HEXB | NM_000521 | Hs.69293 | chr5q13 |
| 201940_at | carboxypeptidase D | CPD | AA897514 | Hs.5057 | chr17p11.1-q11.2 |
| 201939_at | polo-like kinase 2 (Drosophila) | PLK2 | NM_006622 | Hs.398157 | chr5q12.1-q13.2 |
| 201938_at | CDK2-associated protein 1 | CDK2AP1 | NM_004642 | Hs.433201 | chr12q24.31 |
| 201935_s_at | eukaryotic translation initiation factor 4 gamma, 3 | EIF4G3 | AI768122 | Hs.402697 | chr1p36.12 |
| 201934_at | Transmembrane protein 113 | TMEM113 | N92524 | Hs.194110 | chr3p21.2 |
| 201933_at | procollagen (type III) N-endopeptidase | PCOLN3 | NM_002768 | Hs.183138 | chr16q24.3 |
| 201931_at | electron-transfer-flavoprotein, alpha polypeptide (glutaric aciduria II) | ETFA | NM_000126 | Hs.39925 | chr15q23-q25 |
| 201930_at | minichromosome maintenance deficient 6 homolog (S. cerevisiae) | MCM6 | NM_005915 | Hs.444118 | chr2q21 |
| 201927_s_at | plakophilin 4 | PKP4 | BG292559 | Hs.277132 | chr2q23-q31 |
| 201924_at | AF4/FMR2 family, member 1 | AFF1 | NM_005935 | Hs.114765 | chr4q21 |
| 201923_at | peroxiredoxin 4 | PRDX4 | NM_006406 | Hs.83383 | chrxp22.11 |
| 201922_at | TGF beta-inducible nuclear protein 1 | TINP1 | NM_014886 | Hs.436152 | chr5q13.3 |
| 201921_at | guanine nucleotide binding protein (G protein), gamma 10 /// hypothetical protein LOC552891 | GNG10 | NM_004125 | Hs.433898 | chr9q31.3 |
| 201920_at | solute carrier family 20 (phosphate transporter), member 1 | SLC20A1 | NM_005415 | Hs.110855 | chr2q11-q14 |
| 201917_s_at | solute carrier family 25, member 36 | SLC25A36 | AI694452 | Hs.144130 | chr3q23 |
| 201915_at | SEC63 homolog (S. cerevisiae) | SEC63 | AI806665 | Hs.330767 | chr6q21 |
| 201913_s_at | Coenzyme A synthase | COASY | NM_025233 | Hs.296422 | chr17q12-q21 |
| 201912_s_at | G1 to S phase transition 1 /// G1 to S phase transition 1 | GSPT1 | NM_002094 | Hs.2707 | chr16p13.1 |
| 201908_at | dishevelled, dsh homolog 3 (Drosophila) | DVL3 | NM_004423 | Hs.381928 | chr3q27 |
| 201904_s_at | CTD (carboxy-terminal domain, RNA polymerase II, polypeptide A) small phosphatase-like | CTDSPL | BF031714 | Hs.147189 | chr3p21.3 |
| 201903_at | ubiquinol-cytochrome c reductase core protein I | UQCRC1 | NM_003365 | Hs.119251 | chr3p21.3 |
| 201900_s_at | aldo-keto reductase family 1, member A1 (aldehyde reductase) | AKR1A1 | NM_006066 | Hs.372170 | chr1p33-p32 |
| 201898_s_at | ubiquitin-conjugating enzyme E2A (RAD6 homolog) | UBE2A | AI126625 | Hs.379466 | chrxq24-q25 |
| 201897_s_at | CDC28 protein kinase regulatory subunit 1B | CKS1B | NM_001826 | Hs.374378 | chr1q21.2 |
| 201895_at | v-raf murine sarcoma 3611 viral oncogene homolog | ARAF | NM_001654 | Hs.522631 | chrxp11.4-p11.2 |
| 201892_s_at | IMP (inosine monophosphate) dehydrogenase 2 | IMPDH2 | NM_000884 | Hs.75432 | chr3p21.2 |
| 201891_s_at | beta-2-microglobulin | B2M | NM_004048 | Hs.48516 | chr15q21-q22.2 |
| 201889_at | family with sequence similarity 3, member C | FAM3C | NM_014888 | Hs.434053 | chr7q22.1-q31.1 |
| 201888_s_at | interleukin 13 receptor, alpha 1 | IL13RA1 | U81379 | Hs.285115 | chrxq24 |
| 201886_at | WD repeat domain 23 | WDR23 | NM_025230 | Hs.283976 | chr14q11.2 |
| 201872_s_at | ATP-binding cassette, sub-family E (OABP), member 1 | ABCE1 | AI002002 | Hs.12013 | chr4q31 |
| 201871_s_at | unknown protein LOC51035 | LOC51035 | NM_015853 | Hs.351296 | chr11q12.3 |
| 201870_at | translocase of outer mitochondrial membrane 34 | TOMM34 | NM_006809 | Hs.76927 | chr20q12-q13.1 |
| 201864_at | GDP dissociation inhibitor 1 | GDI1 | NM_001493 | Hs.74576 | chrxq28 |
| 201863_at | family with sequence similarity 32, member A | FAM32A | NM_014077 | Hs.4766 | chr19pter-p13.3 |
| 201860_s_at | plasminogen activator, tissue | PLAT | NM_000930 | Hs.274404 | chr8p12 |
| 201858_s_at | proteoglycan 1, secretory granule | PRG1 | J03223 | Hs.1908 | chr10q22.1 |
| 201854_s_at | ATM/ATR-Substrate Chk2-Interacting Zn2+-finger protein | ASCIZ | AI744148 | Hs.16349 | chr16q23.2 |
| 201853_s_at | cell division cycle 25 homolog B (S. cerevisiae) | CDC25B | NM_021873 | Hs.153752 | chr20p13 |
| 201851_at | SH3-domain GRB2-like 1 | SH3GL1 | NM_003025 | Hs.97616 | chr19p13.3 |
| 201850_at | capping protein (actin filament), gelsolin-like | CAPG | NM_001747 | Hs.82422 | chr2cen-q24 |
| 201848_s_at | BCL2/adenovirus E1B 19kDa interacting protein 3 | BNIP3 | U15174 | Hs.60389 | chr10q26.3 |
| 201847_at | lipase A, lysosomal acid, cholesterol esterase (Wolman disease) | LIPA | NM_000235 | Hs.85226 | chr10q23.2-q23.3 |
| 201844_s_at | RING1 and YY1 binding protein | RYBP | W84482 | Hs.7910 | chr3p13 |
| 201842_s_at | EGF-containing fibulin-like extracellular matrix protein 1 | EFEMP1 | AI826799 | Hs.76224 | chr2p16 |
| 201841_s_at | heat shock 27kDa protein 1 /// Meis1, myeloid ecotropic viral integration site 1 homolog 3 (mouse) | HSPB1 | NM_001540 | Hs.76067 | chr7q11.23 |
| 201840_at | neural precursor cell expressed, developmentally down-regulated 8 | NEDD8 | NM_006156 | Hs.75512 | chr14q11.2 |
| 201839_s_at | tumor-associated calcium signal transducer 1 | TACSTD1 | NM_002354 | Hs.692 | chr2p21 |
| 201836_s_at | suppressor of Ty 7 (S. cerevisiae)-like | SUPT7L | AU154740 | Hs.6232 | chr2pter-p25.1 |
| 201834_at | protein kinase, AMP-activated, beta 1 non-catalytic subunit | PRKAB1 | BC001007 | Hs.6061 | chr12q24.1 |
| 201833_at | histone deacetylase 2 | HDAC2 | NM_001527 | Hs.3352 | chr6q21 |
| 201830_s_at | neuroepithelial cell transforming gene 1 | NET1 | NM_005863 | Hs.25155 | chr10p15 |
| 201828_x_at | family with sequence similarity 127, member A | FAM127A | NM_003928 | Hs.250708 | chrxq26 |
| 201827_at | SWI/SNF related, matrix associated, actin dependent regulator of chromatin, subfamily d, member 2 | SMARCD2 | AF113019 | Hs.250581 | chr17q23-q24 |
| 201825_s_at | saccharopine dehydrogenase (putative) | SCCPDH | AL572542 | Hs.238126 | chr1q44 |
| 201823_s_at | ring finger protein 14 | RNF14 | NM_004290 | Hs.170926 | chr5q23.3-q31.1 |
| 201821_s_at | translocase of inner mitochondrial membrane 17 homolog A (yeast) | TIMM17A | BC004439 | Hs.20716 | chr1q32.1 |
| 201818_at | acyltransferase like 2 | AYTL2 | NM_024830 | Hs.179882 | chr5p15.33 |
| 201817_at | ubiquitin protein ligase E3C | UBE3C | NM_014671 | Hs.155287 | chr7q36.3 |
| 201816_s_at | glioblastoma amplified sequence | GBAS | NM_001483 | Hs.152707 | chr7p12 |
| 201813_s_at | TBC1 domain family, member 5 | TBC1D5 | AI654161 | Hs.115740 | chr3p24.3 |
| 201812_s_at | translocase of outer mitochondrial membrane 7 homolog (yeast) /// hypothetical protein LOC201725 | TOMM7 | NM_019059 | Hs.380920 | chr4q32.1 /// chr7p15.3 |
| 201810_s_at | SH3-domain binding protein 5 (BTK-associated) | SH3BP5 | AL562152 | Hs.109150 | chr3p24.3 |
| 201807_at | vacuolar protein sorting 26 homolog A (yeast) | VPS26A | NM_004896 | Hs.114740 | chr10q21.1 |
| 201805_at | protein kinase, AMP-activated, gamma 1 non-catalytic subunit | PRKAG1 | NM_002733 | Hs.3136 | chr12q12-q14 |
| 201804_x_at | tubulin folding cofactor B | TBCB | NM_001281 | Hs.31053 | chr19q13.11-q13.12 |
| 201799_s_at | oxysterol binding protein | OSBP | AI927993 | Hs.24734 | chr11q12-q13 |
| 201797_s_at | valyl-tRNA synthetase | VARS | NM_006295 | Hs.159637 | chr6p21.3 |
| 201795_at | lamin B receptor | LBR | NM_002296 | Hs.435166 | chr1q42.1 |
| 201794_s_at | Smg-7 homolog, nonsense mediated mRNA decay factor (C. elegans) | SMG7 | NM_014837 | Hs.43660 | chr1q25 |
| 201792_at | AE binding protein 1 | AEBP1 | NM_001129 | Hs.439463 | chr7p13 |
| 201790_s_at | 7-dehydrocholesterol reductase | DHCR7 | AW150953 | Hs.11806 | chr11q13.2-q13.5 |
| 201788_at | DEAD (Asp-Glu-Ala-Asp) box polypeptide 42 | DDX42 | NM_007372 | Hs.8765 | chr17q23.3 |
| 201786_s_at | adenosine deaminase, RNA-specific | ADAR | NM_001111 | Hs.7957 | chr1q21.1-q21.2 |
| 201785_at | ribonuclease, RNase A family, 1 (pancreatic) | RNASE1 | NM_002933 | Hs.78224 | chr14q11.2 |
| 201783_s_at | v-rel reticuloendotheliosis viral oncogene homolog A, nuclear factor of kappa light polypeptide gene enhancer in B-cells 3, p65 (avian) | RELA | NM_021975 |  |  |
| 201781_s_at | aryl hydrocarbon receptor interacting protein | AIP | AL558532 | Hs.412433 | chr11q13.3 |
| 201779_s_at | ring finger protein 13 | RNF13 | AF070558 | Hs.6900 | chr3q25.1 |
| 201775_s_at | KIAA0494 | KIAA0494 | AA676790 | Hs.269902 | chr1pter-p22.1 |
| 201771_at | secretory carrier membrane protein 3 | SCAMP3 | NM_005698 | Hs.200600 | chr1q21 |
| 201770_at | small nuclear ribonucleoprotein polypeptide A | SNRPA | NM_004596 | Hs.173255 | chr19q13.1 |
| 201768_s_at | clathrin interactor 1 | CLINT1 | BC004467 | Hs.132853 | chr5q23.1-q33.3 |
| 201767_s_at | elaC homolog 2 (E. coli) | ELAC2 | NM_018127 | Hs.12124 | chr17p11.2 |
| 201765_s_at | hexosaminidase A (alpha polypeptide) | HEXA | AL523158 | Hs.411157 | chr15q23-q24 |
| 201763_s_at | death-associated protein 6 | DAXX | NM_001350 | Hs.336916 | chr6p21.3 |
| 201762_s_at | proteasome (prosome, macropain) activator subunit 2 (PA28 beta) | PSME2 | NM_002818 | Hs.434081 | chr14q11.2 |
| 201761_at | methylenetetrahydrofolate dehydrogenase (NADP+ dependent) 2, methenyltetrahydrofolate cyclohydrolase | MTHFD2 | NM_006636 | Hs.154672 | chr2p13.1 |
| 201760_s_at | WD repeat and SOCS box-containing 2 | WSB2 | NM_018639 | Hs.459470 | chr12q24.23 |
| 201758_at | tumor susceptibility gene 101 | TSG101 | NM_006292 | Hs.444556 | chr11p15 |
| 201757_at | NADH dehydrogenase (ubiquinone) Fe-S protein 5, 15kDa (NADH-coenzyme Q reductase) | NDUFS5 | NM_004552 | Hs.409829 | chr1p34.2-p33 |
| 201756_at | replication protein A2, 32kDa | RPA2 | NM_002946 | Hs.79411 | chr1p35 |
| 201754_at | cytochrome c oxidase subunit VIc | COX6C | NM_004374 | Hs.351875 | chr8q22-q23 |
| 201751_at | Josephin domain containing 1 | JOSD1 | NM_014876 | Hs.3094 | chr22q13.1 |
| 201750_s_at | endothelin converting enzyme 1 | ECE1 | NM_001397 | Hs.492802 | chr1p36.1 |
| 201748_s_at | scaffold attachment factor B | SAFB | NM_002967 | Hs.23978 | chr19p13.3-p13.2 |
| 201746_at | tumor protein p53 (Li-Fraumeni syndrome) | TP53 | NM_000546 | Hs.408312 | chr17p13.1 |
| 201744_s_at | lumican | LUM | NM_002345 | Hs.406475 | chr12q21.3-q22 |
| 201740_at | NADH dehydrogenase (ubiquinone) Fe-S protein 3, 30kDa (NADH-coenzyme Q reductase) | NDUFS3 | NM_004551 | Hs.429506 | chr11p11.11 |
| 201739_at | serum/glucocorticoid regulated kinase | SGK | NM_005627 | Hs.296323 | chr6q23 |
| 201738_at | eukaryotic translation initiation factor 1B | EIF1B | NM_005875 | Hs.315230 | chr3p22.1 |
| 201736_s_at | membrane-associated ring finger (C3HC4) 6 | 06-mars | BF000409 | Hs.380875 | chr5p15.2 |
| 201728_s_at | KIAA0100 /// coiled-coil domain containing 134 | KIAA0100 | AA904674 | Hs.151761 | chr17q11.2 |
| 201725_at | cell division cycle 123 homolog (S. cerevisiae) | CDC123 | NM_006023 | Hs.412842 | chr10p13 |
| 201721_s_at | lysosomal associated multispanning membrane protein 5 | LAPTM5 | NM_006762 | Hs.436200 | chr1p34 |
| 201717_at | mitochondrial ribosomal protein L49 | MRPL49 | NM_004927 | Hs.75859 | chr11q13 |
| 201715_s_at | apoptotic chromatin condensation inducer 1 | ACIN1 | NM_014977 | Hs.227133 | chr14q11.2 |
| 201714_at | tubulin, gamma 1 | TUBG1 | NM_001070 | Hs.21635 | chr17q21 |
| 201712_s_at | RAN binding protein 2 | RANBP2 | NM_006267 | Hs.199179 | chr2q12.3 |
| 201709_s_at | nipsnap homolog 1 (C. elegans) | NIPSNAP1 | NM_003634 | Hs.173878 | chr22q12.2 |
| 201706_s_at | peroxisomal biogenesis factor 19 | PEX19 | BC000496 | Hs.441109 | chr1q22 |
| 201705_at | proteasome (prosome, macropain) 26S subunit, non-ATPase, 7 (Mov34 homolog) | PSMD7 | NM_002811 | Hs.440604 | chr16q23-q24 |
| 201704_at | ectonucleoside triphosphate diphosphohydrolase 6 (putative function) | ENTPD6 | NM_001247 | Hs.438431 | chr20p11.2-p11.22 |
| 201702_s_at | protein phosphatase 1, regulatory subunit 10 | PPP1R10 | AI492873 | Hs.106019 | chr6p21.3 |
| 201701_s_at | progesterone receptor membrane component 2 | PGRMC2 | NM_006320 | Hs.9071 | chr4q26 |
| 201699_at | proteasome (prosome, macropain) 26S subunit, ATPase, 6 | PSMC6 | NM_002806 | Hs.156171 | chr14q22.1 |
| 201695_s_at | nucleoside phosphorylase | NP | NM_000270 | Hs.75514 | chr14q13.1 |
| 201693_s_at | early growth response 1 | EGR1 | AV733950 | Hs.326035 | chr5q31.1 |
| 201688_s_at | tumor protein D52 | TPD52 | BG389015 | Hs.162089 | chr8q21 |
| 201682_at | peptidase (mitochondrial processing) beta | PMPCB | NM_004279 | Hs.184211 | chr7q22-q32 |
| 201681_s_at | discs, large homolog 5 (Drosophila) | DLG5 | AB011155 | Hs.500245 | chr10q23 |
| 201680_x_at | ARS2 protein | ARS2 | NM_015908 | Hs.111801 | chr7q21 |
| 201677_at | Chromosome 3 open reading frame 37 | C3orf37 | AI937543 | Hs.458320 | chr3q21.3 |
| 201676_x_at | proteasome (prosome, macropain) subunit, alpha type, 1 | PSMA1 | NM_002786 | Hs.82159 | chr11p15.1 |
| 201675_at | A kinase (PRKA) anchor protein 1 | AKAP1 | NM_003488 | Hs.78921 | chr17q21-q23 |
| 201673_s_at | glycogen synthase 1 (muscle) | GYS1 | NM_002103 | Hs.386225 | chr19q13.3 |
| 201671_x_at | ubiquitin specific peptidase 14 (tRNA-guanine transglycosylase) | USP14 | BC003556 | Hs.75981 | chr18p11.32 |
| 201668_x_at | myristoylated alanine-rich protein kinase C substrate | MARCKS | AW163148 | Hs.318603 | chr6q22.2 |
| 201667_at | gap junction protein, alpha 1, 43kDa (connexin 43) | GJA1 | NM_000165 | Hs.74471 | chr6q21-q23.2 |
| 201666_at | TIMP metallopeptidase inhibitor 1 | TIMP1 | NM_003254 | Hs.446641 | chrxp11.3-p11.23 |
| 201665_x_at | ribosomal protein S17 | RPS17 | NM_001021 | Hs.433427 | chr15q |
| 201663_s_at | structural maintenance of chromosomes 4 | SMC4 | NM_005496 | Hs.212957 | chr3q26.1 |
| 201660_at | Acyl-CoA synthetase long-chain family member 3 | ACSL3 | AL525798 | Hs.268012 | chr2q34-q35 |
| 201657_at | ADP-ribosylation factor-like 1 | ARL1 | BE890745 | Hs.372616 | chr12q23.3 |
| 201654_s_at | heparan sulfate proteoglycan 2 (perlecan) | HSPG2 | AI991033 | Hs.211573 | chr1p36.1-p35 |
| 201653_at | cornichon homolog (Drosophila) | CNIH | NM_005776 | Hs.294603 | chr14q22.2 |
| 201652_at | COP9 constitutive photomorphogenic homolog subunit 5 (Arabidopsis) | COPS5 | NM_006837 | Hs.446492 | chr8q13.2 |
| 201649_at | ubiquitin-conjugating enzyme E2L 6 | UBE2L6 | NM_004223 | Hs.425777 | chr11q12 |
| 201646_at | scavenger receptor class B, member 2 | SCARB2 | AA885297 | Hs.349656 | chr4q21.1 |
| 201643_x_at | jumonji domain containing 1B | JMJD1B | NM_016604 | Hs.24125 | chr5q31 |
| 201642_at | interferon gamma receptor 2 (interferon gamma transducer 1) | IFNGR2 | NM_005534 | Hs.409200 | chr21q22.11 |
| 201640_x_at | cleft lip and palate associated transmembrane protein 1 | CLPTM1 | NM_001294 | Hs.444441 | chr19q13.2-q13.3 |
| 201639_s_at | cleavage and polyadenylation specific factor 1, 160kDa | CPSF1 | NM_013291 | Hs.83727 | chr8q24.23 |
| 201635_s_at | fragile X mental retardation, autosomal homolog 1 | FXR1 | AI990766 | Hs.408096 | chr3q28 |
| 201632_at | eukaryotic translation initiation factor 2B, subunit 1 alpha, 26kDa | EIF2B1 | NM_001414 | Hs.78592 | chr12q24.31 |
| 201631_s_at | immediate early response 3 | IER3 | NM_003897 | Hs.76095 | chr6p21.3 |
| 201629_s_at | acid phosphatase 1, soluble | ACP1 | BE872974 | Hs.130873 | chr2p25 |
| 201628_s_at | Ras-related GTP binding A | RRAGA | NM_006570 | Hs.432330 | chr9p22.1 |
| 201625_s_at | insulin induced gene 1 | INSIG1 | BE300521 | Hs.416385 | chr7q36 |
| 201623_s_at | aspartyl-tRNA synthetase | DARS | BC000629 | Hs.32393 | chr2q21.3 |
| 201622_at | staphylococcal nuclease and tudor domain containing 1 | SND1 | NM_014390 | Hs.511400 | chr7q31.3 |
| 201619_at | peroxiredoxin 3 | PRDX3 | NM_006793 | Hs.397062 | chr10q25-q26 |
| 201618_x_at | glycosylphosphatidylinositol anchor attachment protein 1 homolog (yeast) | GPAA1 | NM_003801 | Hs.4742 | chr8q24.3 |
| 201615_x_at | caldesmon 1 | CALD1 | AI685060 | Hs.443811 | chr7q33 |
| 201614_s_at | RuvB-like 1 (E. coli) | RUVBL1 | NM_003707 | Hs.272822 | chr3q21 |
| 201613_s_at | adaptor-related protein complex 1, gamma 2 subunit | AP1G2 | BC000519 | Hs.343244 | chr14q11.2 |
| 201612_at | aldehyde dehydrogenase 9 family, member A1 | ALDH9A1 | NM_000696 | Hs.2533 | chr1q23.1 |
| 201609_x_at | isoprenylcysteine carboxyl methyltransferase | ICMT | AL578502 | Hs.183212 | chr1p36.21 |
| 201607_at | PWP1 homolog (S. cerevisiae) | PWP1 | AI694451 | Hs.172589 | chr12q23.3 |
| 201605_x_at | calponin 2 | CNN2 | NM_004368 | Hs.169718 | chr21q11.1 |
| 201602_s_at | protein phosphatase 1, regulatory (inhibitor) subunit 12A | PPP1R12A | BE737620 | Hs.377908 | chr12q15-q21 |
| 201601_x_at | interferon induced transmembrane protein 1 (9-27) | IFITM1 | NM_003641 | Hs.458414 | chr11p15.5 |
| 201600_at | prohibitin 2 | PHB2 | NM_007273 | Hs.444499 | chr12p13 |
| 201599_at | ornithine aminotransferase (gyrate atrophy) | OAT | NM_000274 | Hs.75485 | chr10q26 |
| 201598_s_at | inositol polyphosphate phosphatase-like 1 | INPPL1 | NM_001567 | Hs.75339 | chr11q23 |
| 201597_at | cytochrome c oxidase subunit VIIa polypeptide 2 (liver) | COX7A2 | NM_001865 | Hs.70312 | chr6q12 |
| 201594_s_at | protein phosphatase 4, regulatory subunit 1 | PPP4R1 | NM_005134 | Hs.435789 | chr18p11.22 |
| 201593_s_at | zinc finger CCCH-type containing 15 | ZC3H15 | AV716798 | Hs.368598 | chr2q32.1 |
| 201592_at | eukaryotic translation initiation factor 3, subunit 3 gamma, 40kDa | EIF3S3 | NM_003756 | Hs.127149 | chr8q24.11 |
| 201591_s_at | nischarin | NISCH | NM_007184 | Hs.435290 | chr3p21.1 |
| 201590_x_at | annexin A2 | ANXA2 | NM_004039 | Hs.462864 | chr15q21-q22 |
| 201589_at | structural maintenance of chromosomes 1A | SMC1A | D80000 | Hs.211602 | chrxp11.22-p11.21 |
| 201588_at | thioredoxin-like 1 | TXNL1 | NM_004786 | Hs.114412 | chr18q21.2 |
| 201587_s_at | interleukin-1 receptor-associated kinase 1 | IRAK1 | NM_001569 | Hs.182018 | chrxq28 |
| 201584_s_at | DEAD (Asp-Glu-Ala-Asp) box polypeptide 39 | DDX39 | NM_005804 | Hs.311609 | chr19p13.12 |
| 201582_at | Sec23 homolog B (S. cerevisiae) | SEC23B | AL121900 | Hs.369373 | chr20p11.23 |
| 201580_s_at | thioredoxin domain containing 13 | TXNDC13 | AL544094 | Hs.169358 | chr20p12 |
| 201579_at | FAT tumor suppressor homolog 1 (Drosophila) | FAT | NM_005245 | Hs.166994 | chr4q34-q35 |
| 201577_at | non-metastatic cells 1, protein (NM23A) expressed in | NME1 | NM_000269 | Hs.118638 | chr17q21.3 |
| 201573_s_at | eukaryotic translation termination factor 1 | ETF1 | M75715 | Hs.77324 | chr5q31.1 |
| 201571_s_at | dCMP deaminase | DCTD | AI656493 | Hs.76894 | chr4q35.1 |
| 201569_s_at | sorting and assembly machinery component 50 homolog (S. cerevisiae) | SAMM50 | NM_015380 | Hs.505824 | chr22q13.31 |
| 201568_at | ubiquinol-cytochrome c reductase, complex III subunit VII, 9.5kDa | UQCRQ | NM_014402 | Hs.146602 | chr5q31.1 |
| 201567_s_at | golgi autoantigen, golgin subfamily a, 4 | GOLGA4 | NM_002078 | Hs.434433 | chr3p22-p21.3 |
| 201566_x_at | inhibitor of DNA binding 2, dominant negative helix-loop-helix protein /// inhibitor of DNA binding 2B, dominant negative helix-loop-helix protein | ID2 | D13891 | Hs.180919 | chr2p25 |
| 201563_at | sorbitol dehydrogenase | SORD | L29008 | Hs.878 | chr15q15.3 |
| 201561_s_at | calsyntenin 1 | CLSTN1 | NM_014944 | Hs.29665 | chr1p36.22 |
| 201559_s_at | chloride intracellular channel 4 | CLIC4 | AF109196 | Hs.25035 | chr1p36.11 |
| 201556_s_at | vesicle-associated membrane protein 2 (synaptobrevin 2) | VAMP2 | BC002737 | Hs.25348 | chr17p13.1 |
| 201555_at | MCM3 minichromosome maintenance deficient 3 (S. cerevisiae) | MCM3 | NM_002388 | Hs.179565 | chr6p12 |
| 201554_x_at | glycogenin 1 | GYG1 | NM_004130 | Hs.174071 | chr3q24-q25.1 |
| 201551_s_at | lysosomal-associated membrane protein 1 | LAMP1 | J03263 | Hs.150101 | chr13q34 |
| 201550_x_at | actin, gamma 1 | ACTG1 | NM_001614 | Hs.14376 | chr17q25 |
| 201548_s_at | jumonji, AT rich interactive domain 1B | JARID1B | W02593 | Hs.370679 | chr1q32.1 |
| 201542_at | SAR1 gene homolog A (S. cerevisiae) | SAR1A | AY008268 | Hs.110796 | chr10q22.1 |
| 201541_s_at | zinc finger, HIT type 1 | ZNHIT1 | NM_006349 | Hs.211079 | chr7q22.1 |
| 201540_at | four and a half LIM domains 1 | FHL1 | NM_001449 | Hs.421383 | chrxq26 |
| 201538_s_at | dual specificity phosphatase 3 (vaccinia virus phosphatase VH1-related) | DUSP3 | NM_004090 | Hs.181046 | chr17q21 |
| 201534_s_at | ubiquitin-like 3 | UBL3 | AF044221 | Hs.145575 | chr13q12-q13 |
| 201532_at | proteasome (prosome, macropain) subunit, alpha type, 3 | PSMA3 | NM_002788 | Hs.246240 | chr14q23 |
| 201531_at | zinc finger protein 36, C3H type, homolog (mouse) | ZFP36 | NM_003407 | Hs.343586 | chr19q13.1 |
| 201530_x_at | eukaryotic translation initiation factor 4A, isoform 1 | EIF4A1 | NM_001416 | Hs.129673 | chr17p13 |
| 201529_s_at | replication protein A1, 70kDa | RPA1 | NM_002945 | Hs.84318 | chr17p13.3 |
| 201527_at | ATPase, H+ transporting, lysosomal 14kDa, V1 subunit F | ATP6V1F | NM_004231 | Hs.78089 | chr7q32 |
| 201526_at | ADP-ribosylation factor 5 | ARF5 | NM_001662 | Hs.430657 | chr7q31.3 |
| 201522_x_at | small nuclear ribonucleoprotein polypeptide N /// SNRPN upstream reading frame | SNRPN | NM_003097 | Hs.48375 | chr15q12 /// chr15q11.2 |
| 201521_s_at | nuclear cap binding protein subunit 2, 20kDa | NCBP2 | NM_007362 | Hs.240770 | chr3q29 |
| 201518_at | chromobox homolog 1 (HP1 beta homolog Drosophila ) | CBX1 | NM_006807 | Hs.77254 | chr17q |
| 201516_at | spermidine synthase | SRM | NM_003132 | Hs.76244 | chr1p36-p22 |
| 201512_s_at | translocase of outer mitochondrial membrane 70 homolog A (S. cerevisiae) | TOMM70A | BC003633 | Hs.370688 | chr3q12.2 |
| 201511_at | angio-associated, migratory cell protein | AAMP | NM_001087 | Hs.83347 | chr2q35 |
| 201507_at | prefoldin subunit 1 | PFDN1 | NM_002622 | Hs.132881 | chr5q31 |
| 201505_at | laminin, beta 1 | LAMB1 | NM_002291 | Hs.30237 | chr7q22 |
| 201504_s_at | translin | TSN | AI435302 | Hs.75066 | chr2q21.1 |
| 201502_s_at | nuclear factor of kappa light polypeptide gene enhancer in B-cells inhibitor, alpha | NFKBIA | AI078167 | Hs.81328 | chr14q13 |
| 201498_at | ubiquitin specific peptidase 7 (herpes virus-associated) | USP7 | AI160440 | Hs.386939 | chr16p13.3 |
| 201494_at | prolylcarboxypeptidase (angiotensinase C) | PRCP | NM_005040 | Hs.314089 | chr11q14 |
| 201493_s_at | pumilio homolog 2 (Drosophila) | PUM2 | BE778078 | Hs.133543 | chr2p22-p21 |
| 201491_at | AHA1, activator of heat shock 90kDa protein ATPase homolog 1 (yeast) | AHSA1 | NM_012111 | Hs.204041 | chr14q23.3-31 |
| 201490_s_at | peptidylprolyl isomerase F (cyclophilin F) | PPIF | NM_005729 | Hs.381072 | chr10q22-q23 |
| 201485_s_at | reticulocalbin 2, EF-hand calcium binding domain | RCN2 | BC004892 | Hs.79088 | chr15q23 |
| 201484_at | suppressor of Ty 4 homolog 1 (S. cerevisiae) | SUPT4H1 | NM_003168 | Hs.79058 | chr17q21-q23 |
| 201482_at | quiescin Q6 | QSCN6 | NM_002826 | Hs.77266 | chr1q24 |
| 201481_s_at | phosphorylase, glycogen; brain | PYGB | NM_002862 | Hs.145820 | chr20p11.2-p11.1 |
| 201480_s_at | suppressor of Ty 5 homolog (S. cerevisiae) | SUPT5H | NM_003169 | Hs.437056 | chr19q13 |
| 201476_s_at | ribonucleotide reductase M1 polypeptide | RRM1 | AI692974 | Hs.383396 | chr11p15.5 |
| 201475_x_at | methionine-tRNA synthetase | MARS | NM_004990 | Hs.355867 | chr12q13.2 |
| 201473_at | jun B proto-oncogene | JUNB | NM_002229 | Hs.515157 | chr19p13.2 |
| 201472_at | von Hippel-Lindau binding protein 1 | VBP1 | NM_003372 | Hs.436803 | chrxq28 |
| 201469_s_at | SHC (Src homology 2 domain containing) transforming protein 1 | SHC1 | AI809967 | Hs.433795 | chr1q21 |
| 201468_s_at | NAD(P)H dehydrogenase, quinone 1 | NQO1 | NM_000903 | Hs.406515 | chr16q22.1 |
| 201463_s_at | transaldolase 1 | TALDO1 | NM_006755 | Hs.438678 | chr11p15.5-p15.4 |
| 201462_at | secernin 1 | SCRN1 | NM_014766 | Hs.75137 | chr7p14.3-p14.1 |
| 201461_s_at | mitogen-activated protein kinase-activated protein kinase 2 | MAPKAPK2 | NM_004759 | Hs.75074 | chr1q32 |
| 201459_at | RuvB-like 2 (E. coli) | RUVBL2 | NM_006666 | Hs.6455 | chr19q13.3 |
| 201456_s_at | BUB3 budding uninhibited by benzimidazoles 3 homolog (yeast) | BUB3 | AU160695 | Hs.418533 | chr10q26 |
| 201454_s_at | aminopeptidase puromycin sensitive | NPEPPS | AW055008 | Hs.293007 | chr17q21 |
| 201446_s_at | TIA1 cytotoxic granule-associated RNA binding protein | TIA1 | BF692742 | Hs.391858 | chr2p13 |
| 201445_at | calponin 3, acidic | CNN3 | NM_001839 | Hs.194662 | chr1p22-p21 |
| 201443_s_at | ATPase, H+ transporting, lysosomal accessory protein 2 | ATP6AP2 | AF248966 | Hs.183434 | chrxq21 |
| 201441_at | cytochrome c oxidase subunit Vib polypeptide 1 (ubiquitous) | COX6B1 | NM_001863 | Hs.431668 | chr19q13.1 |
| 201439_at | golgi-specific brefeldin A resistance factor 1 | GBF1 | NM_004193 | Hs.155499 | chr10q24 |
| 201438_at | collagen, type VI, alpha 3 | COL6A3 | NM_004369 | Hs.233240 | chr2q37 |
| 201436_at | eukaryotic translation initiation factor 4E | EIF4E | AI742789 | Hs.79306 | chr4q21-q25 |
| 201434_at | tetratricopeptide repeat domain 1 | TTC1 | NM_003314 | Hs.7733 | chr5q32-q33.2 |
| 201433_s_at | phosphatidylserine synthase 1 | PTDSS1 | NM_014754 | Hs.77329 | chr8q22 |
| 201430_s_at | dihydropyrimidinase-like 3 | DPYSL3 | W72516 | Hs.150358 | chr5q32 |
| 201429_s_at | polo-like kinase 1 (Drosophila) /// ribosomal protein L37a | PLK1 | NM_000998 | Hs.433701 | chr2q35 |
| 201427_s_at | selenoprotein P, plasma, 1 | SEPP1 | NM_005410 | Hs.275775 | chr5q31 |
| 201425_at | aldehyde dehydrogenase 2 family (mitochondrial) | ALDH2 | NM_000690 | Hs.436437 | chr12q24.2 |
| 201422_at | interferon, gamma-inducible protein 30 | IFI30 | NM_006332 | Hs.14623 | chr19p13.1 |
| 201421_s_at | WD repeat domain 77 | WDR77 | NM_024102 | Hs.446551 | chr1p13.2 |
| 201419_at | BRCA1 associated protein-1 (ubiquitin carboxy-terminal hydrolase) | BAP1 | NM_004656 | Hs.106674 | chr3p21.31-p21.2 |
| 201416_at | SRY (sex determining region Y)-box 4 | SOX4 | BG528420 | Hs.357901 | chr6p22.3 |
| 201415_at | glutathione synthetase | GSS | NM_000178 | Hs.82327 | chr20q11.2 |
| 201413_at | hydroxysteroid (17-beta) dehydrogenase 4 | HSD17B4 | NM_000414 | Hs.356894 | chr5q21 |
| 201410_at | Pleckstrin homology domain containing, family B (evectins) member 2 | PLEKHB2 | AI983043 | Hs.307033 | chr2q21.1 |
| 201407_s_at | protein phosphatase 1, catalytic subunit, beta isoform | PPP1CB | AI186712 | Hs.21537 | chr2p23 |
| 201406_at | ribosomal protein L36a /// similar to large subunit ribosomal protein L36a | RPL36A | NM_021029 | Hs.454495 | chrxq22.1 |
| 201400_at | proteasome (prosome, macropain) subunit, beta type, 3 | PSMB3 | NM_002795 | Hs.82793 | chr17q12 |
| 201399_s_at | translocation associated membrane protein 1 | TRAM1 | NM_014294 | Hs.4147 | chr8q13.3 |
| 201397_at | phosphoglycerate dehydrogenase | PHGDH | NM_006623 | Hs.3343 | chr1p12 |
| 201396_s_at | small glutamine-rich tetratricopeptide repeat (TPR)-containing, alpha | SGTA | NM_003021 | Hs.203910 | chr19p13 |
| 201394_s_at | RNA binding motif protein 5 | RBM5 | U23946 | Hs.439480 | chr3p21.3 |
| 201392_s_at | insulin-like growth factor 2 receptor | IGF2R | BG031974 | Hs.76473 | chr6q26 |
| 201391_at | TNF receptor-associated protein 1 | TRAP1 | NM_016292 | Hs.183803 | chr16p13.3 |
| 201390_s_at | casein kinase 2, beta polypeptide | CSNK2B | NM_001320 | Hs.73527 | chr6p21.3 |
| 201389_at | integrin, alpha 5 (fibronectin receptor, alpha polypeptide) | ITGA5 | NM_002205 | Hs.149609 | chr12q11-q13 |
| 201388_at | proteasome (prosome, macropain) 26S subunit, non-ATPase, 3 | PSMD3 | NM_002809 | Hs.9736 | chr17q21.1 |
| 201387_s_at | ubiquitin carboxyl-terminal esterase L1 (ubiquitin thiolesterase) | UCHL1 | NM_004181 | Hs.76118 | chr4p14 |
| 201385_at | DEAH (Asp-Glu-Ala-His) box polypeptide 15 | DHX15 | NM_001358 | Hs.220704 | chr4p15.3 |
| 201383_s_at | neighbor of BRCA1 gene 1 /// similar to neighbor of BRCA1 gene 1 | NBR1 | AL044170 | Hs.135500 | chr17q21.1 |
| 201379_s_at | tumor protein D52-like 2 | TPD52L2 | NM_003288 | Hs.154718 | chr20q13.2-q13.3 |
| 201376_s_at | heterogeneous nuclear ribonucleoprotein F | HNRPF | AI591354 | Hs.808 | chr10q11.21-q11.22 |
| 201375_s_at | protein phosphatase 2 (formerly 2A), catalytic subunit, beta isoform | PPP2CB | NM_004156 | Hs.80350 | chr8p12-p11.2 |
| 201368_at | zinc finger protein 36, C3H type-like 2 | ZFP36L2 | U07802 | Hs.78909 | chr2p22.3-p21 |
| 201366_at | annexin A7 | ANXA7 | NM_004034 | Hs.386741 | chr10q21.1-q21.2 |
| 201364_s_at | ornithine decarboxylase antizyme 2 | OAZ2 | AF242521 | Hs.74563 | chr15q22.31 |
| 201363_s_at | influenza virus NS1A binding protein | IVNS1ABP | AB020657 | Hs.197298 | chr1q25.1-q31.1 |
| 201358_s_at | coatomer protein complex, subunit beta 1 | COPB1 | NM_016451 | Hs.3059 | chr11p15.2 |
| 201354_s_at | bromodomain adjacent to zinc finger domain, 2A | BAZ2A | AA788652 | Hs.150707 | chr12q24.3-qter |
| 201350_at | flotillin 2 | FLOT2 | NM_004475 | Hs.18799 | chr17q11-q12 |
| 201349_at | solute carrier family 9 (sodium/hydrogen exchanger), member 3 regulator 1 | SLC9A3R1 | NM_004252 | Hs.396783 | chr17q25.1 |
| 201348_at | glutathione peroxidase 3 (plasma) | GPX3 | NM_002084 | Hs.386793 | chr5q23 |
| 201347_x_at | glyoxylate reductase/hydroxypyruvate reductase | GRHPR | NM_012203 | Hs.155742 | chr9q12 |
| 201346_at | adiponectin receptor 2 | ADIPOR2 | NM_024551 | Hs.334854 | chr12p13.31 |
| 201342_at | small nuclear ribonucleoprotein polypeptide C | SNRPC | NM_003093 | Hs.1063 | chr6p21.31 |
| 201341_at | ectodermal-neural cortex (with BTB-like domain) | ENC1 | NM_003633 | Hs.104925 | chr5q12-q13.3 |
| 201339_s_at | sterol carrier protein 2 | SCP2 | NM_002979 | Hs.75760 | chr1p32 |
| 201338_x_at | general transcription factor IIIA | GTF3A | NM_002097 | Hs.445977 | chr13q12.3-q13.1 |
| 201337_s_at | vesicle-associated membrane protein 3 (cellubrevin) | VAMP3 | NM_004781 | Hs.66708 | chr1p36.23 |
| 201334_s_at | Rho guanine nucleotide exchange factor (GEF) 12 | ARHGEF12 | AB002380 | Hs.413112 | chr11q23.3 |
| 201331_s_at | signal transducer and activator of transcription 6, interleukin-4 induced | STAT6 | BC004973 | Hs.437475 | chr12q13 |
| 201330_at | arginyl-tRNA synthetase | RARS | NM_002887 | Hs.14280 | chr5q35.1 |
| 201328_at | v-ets erythroblastosis virus E26 oncogene homolog 2 (avian) | ETS2 | AL575509 | Hs.292477 | chr21q22.2 |
| 201326_at | chaperonin containing TCP1, subunit 6A (zeta 1) | CCT6A | BE737030 | Hs.82916 | chr7p11.2 |
| 201325_s_at | epithelial membrane protein 1 | EMP1 | NM_001423 | Hs.306692 | chr12p12.3 |
| 201323_at | EBNA1 binding protein 2 | EBNA1BP2 | NM_006824 | Hs.346868 | chr1p35-p33 |
| 201322_at | ATP synthase, H+ transporting, mitochondrial F1 complex, beta polypeptide | ATP5B | NM_001686 | Hs.406510 | chr12q13.13 |
| 201321_s_at | SWI/SNF related, matrix associated, actin dependent regulator of chromatin, subfamily c, member 2 | SMARCC2 | NM_003075 | Hs.236030 | chr12q13-q14 |
| 201316_at | proteasome (prosome, macropain) subunit, alpha type, 2 | PSMA2 | AL523904 | Hs.333786 | chr7p14.1 |
| 201315_x_at | interferon induced transmembrane protein 2 (1-8D) | IFITM2 | NM_006435 | Hs.174195 | chr11p15.5 |
| 201314_at | serine/threonine kinase 25 (STE20 homolog, yeast) | STK25 | NM_006374 | Hs.155206 | chr2q37.3 |
| 201313_at | enolase 2 (gamma, neuronal) | ENO2 | NM_001975 | Hs.146580 | chr12p13 |
| 201311_s_at | SH3 domain binding glutamic acid-rich protein like | SH3BGRL | AL515318 | Hs.14368 | chrxq13.3 |
| 201305_x_at | acidic (leucine-rich) nuclear phosphoprotein 32 family, member B | ANP32B | AV712577 | Hs.459987 | chr9q22.32 |
| 201304_at | NADH dehydrogenase (ubiquinone) 1 alpha subcomplex, 5, 13kDa | NDUFA5 | NM_005000 | Hs.83916 | chr7q32 |
| 201303_at | eukaryotic translation initiation factor 4A, isoform 3 | EIF4A3 | NM_014740 | Hs.389649 | chr17q25.3 |
| 201301_s_at | annexin A4 | ANXA4 | BC000182 | Hs.422986 | chr2p13 |
| 201297_s_at | MOB1, Mps One Binder kinase activator-like 1B (yeast) | MOBK1B | AK023321 | Hs.196437 | chr2p13.1 |
| 201296_s_at | WD repeat and SOCS box-containing 1 | WSB1 | NM_015626 | Hs.315379 | chr17q11.1 |
| 201291_s_at | topoisomerase (DNA) II alpha 170kDa | TOP2A | AU159942 | Hs.156346 | chr17q21-q22 |
| 201290_at | SEC11 homolog A (S. cerevisiae) | SEC11A | NM_014300 | Hs.9534 | chr15q25.3 |
| 201289_at | cysteine-rich, angiogenic inducer, 61 | CYR61 | NM_001554 | Hs.8867 | chr1p31-p22 |
| 201285_at | makorin, ring finger protein, 1 /// makorin, ring finger protein, 1 | MKRN1 | NM_013446 | Hs.7838 | chr7q34 |
| 201284_s_at | N-acylaminoacyl-peptide hydrolase | APEH | NM_001640 | Hs.221589 | chr3p21.31 |
| 201282_at | oxoglutarate (alpha-ketoglutarate) dehydrogenase (lipoamide) | OGDH | NM_002541 | Hs.168669 | chr7p14-p13 |
| 201281_at | adhesion regulating molecule 1 | ADRM1 | NM_007002 | Hs.90107 | chr20q13.33 |
| 201279_s_at | disabled homolog 2, mitogen-responsive phosphoprotein (Drosophila) | DAB2 | BC003064 | Hs.81988 | chr5p13 |
| 201277_s_at | heterogeneous nuclear ribonucleoprotein A/B | HNRPAB | NM_004499 | Hs.248746 | chr5q35.3 |
| 201276_at | RAB5B, member RAS oncogene family | RAB5B | AF267863 | Hs.77690 | chr12q13 |
| 201274_at | proteasome (prosome, macropain) subunit, alpha type, 5 | PSMA5 | NM_002790 | Hs.76913 | chr1p13 |
| 201273_s_at | signal recognition particle 9kDa | SRP9 | NM_003133 | Hs.528205 | chr1q42.12 |
| 201272_at | aldo-keto reductase family 1, member B1 (aldose reductase) | AKR1B1 | NM_001628 | Hs.75313 | chr7q35 |
| 201271_s_at | RNA binding protein, autoantigenic (hnRNP-associated with lethal yellow homolog (mouse)) | RALY | NM_016732 | Hs.389101 | chr20q11.21-q11.23 |
| 201268_at | non-metastatic cells 2, protein (NM23B) expressed in /// NM23-LV | NME2 | NM_002512 | Hs.433416 | chr17q21.3 |
| 201267_s_at | proteasome (prosome, macropain) 26S subunit, ATPase, 3 | PSMC3 | AL545523 | Hs.250758 | chr11p12-p13 |
| 201266_at | thioredoxin reductase 1 | TXNRD1 | NM_003330 | Hs.501739 | chr12q23-q24.1 |
| 201264_at | coatomer protein complex, subunit epsilon | COPE | NM_007263 | Hs.10326 | chr19p13.11 |
| 201263_at | threonyl-tRNA synthetase | TARS | NM_003191 | Hs.84131 | chr5p13.2 |
| 201259_s_at | synaptophysin-like 1 | SYPL1 | AI768845 | Hs.80919 | chr7q22.3 |
| 201258_at | ribosomal protein S16 | RPS16 | NM_001020 | Hs.397609 | chr19q13.1 |
| 201256_at | cytochrome c oxidase subunit VIIa polypeptide 2 like | COX7A2L | NM_004718 | Hs.423404 | chr2p21 |
| 201255_x_at | HLA-B associated transcript 3 | BAT3 | NM_004639 | Hs.440900 | chr6p21.3 |
| 201253_s_at | CDP-diacylglycerol--inositol 3-phosphatidyltransferase (phosphatidylinositol synthase) | CDIPT | NM_006319 | Hs.121549 | chr16p11.2 |
| 201252_at | proteasome (prosome, macropain) 26S subunit, ATPase, 4 /// similar to 26S protease regulatory subunit 6B (MIP224) (MB67-interacting protein) (TAT-binding protein 7) (TBP-7) | PSMC4 | NM_006503 | Hs.211594 | chr19q13.11-q13.13 |
| 201251_at | pyruvate kinase, muscle | PKM2 | NM_002654 | Hs.198281 | chr15q22 |
| 201250_s_at | solute carrier family 2 (facilitated glucose transporter), member 1 | SLC2A1 | NM_006516 | Hs.169902 | chr1p35-p31.3 |
| 201248_s_at | sterol regulatory element binding transcription factor 2 | SREBF2 | NM_004599 | Hs.108689 | chr22q13 |
| 201242_s_at | ATPase, Na+/K+ transporting, beta 1 polypeptide | ATP1B1 | BC000006 | Hs.274479 | chr1q24 |
| 201241_at | DEAD (Asp-Glu-Ala-Asp) box polypeptide 1 | DDX1 | NM_004939 | Hs.440599 | chr2p24 |
| 201240_s_at | signal peptidase complex subunit 2 homolog (S. cerevisiae) | SPCS2 | NM_014752 | Hs.87095 | chr11q13.4 |
| 201237_at | capping protein (actin filament) muscle Z-line, alpha 2 | CAPZA2 | AV685920 | Hs.369579 | chr7q31.2-q31.3 |
| 201234_at | integrin-linked kinase | ILK | NM_004517 | Hs.89657 | chr11p15.5-p15.4 |
| 201233_at | proteasome (prosome, macropain) 26S subunit, non-ATPase, 13 | PSMD13 | NM_002817 | Hs.279554 | chr11p15.5 |
| 201231_s_at | enolase 1, (alpha) | ENO1 | NM_001428 | Hs.433455 | chr1p36.3-p36.2 |
| 201228_s_at | ariadne homolog 2 (Drosophila) | ARIH2 | AW074830 | Hs.241558 | chr3p21.2-p21.3 |
| 201226_at | NADH dehydrogenase (ubiquinone) 1 beta subcomplex, 8, 19kDa | NDUFB8 | NM_005004 | Hs.198273 | chr10q23.2-q23.33 |
| 201224_s_at | serine/arginine repetitive matrix 1 | SRRM1 | AU147713 | Hs.18192 | chr1p36.11 |
| 201222_s_at | RAD23 homolog B (S. cerevisiae) | RAD23B | AL527365 | Hs.159087 | chr9q31.2 |
| 201221_s_at | small nuclear ribonucleoprotein 70kDa polypeptide (RNP antigen) | SNRP70 | NM_003089 | Hs.174051 | chr19q13.3 |
| 201219_at | C-terminal binding protein 2 /// similar to C-terminal-binding protein 2 (CtBP2) /// similar to C-terminal binding protein 2 | CTBP2 | AW269836 | Hs.171391 | chr10q26.13 |
| 201217_x_at | ribosomal protein L3 | RPL3 | NM_000967 | Hs.119598 | chr22q13 |
| 201216_at | endoplasmic reticulum protein 29 | ERP29 | NM_006817 | Hs.75841 | chr12q24.13 |
| 201215_at | plastin 3 (T isoform) | PLS3 | NM_005032 | Hs.430166 | chrxq23 |
| 201214_s_at | protein phosphatase 1, regulatory subunit 7 | PPP1R7 | NM_002712 | Hs.36587 | chr2q37.3 |
| 201209_at | histone deacetylase 1 | HDAC1 | NM_004964 | Hs.88556 | chr1p34 |
| 201207_at | tumor necrosis factor, alpha-induced protein 1 (endothelial) | TNFAIP1 | NM_021137 | Hs.76090 | chr17q22-q23 |
| 201206_s_at | ribosome binding protein 1 homolog 180kDa (dog) | RRBP1 | NM_004587 | Hs.98614 | chr20p12 |
| 201202_at | proliferating cell nuclear antigen | PCNA | NM_002592 | Hs.78996 | chr20pter-p12 |
| 201200_at | cellular repressor of E1A-stimulated genes 1 | CREG1 | NM_003851 | Hs.5710 | chr1q24 |
| 201198_s_at | proteasome (prosome, macropain) 26S subunit, non-ATPase, 1 | PSMD1 | AI860431 | Hs.3887 | chr2q37.1 |
| 201196_s_at | adenosylmethionine decarboxylase 1 | AMD1 | M21154 | Hs.159118 | chr6q21-q22 |
| 201195_s_at | solute carrier family 7 (cationic amino acid transporter, y+ system), member 5 | SLC7A5 | AB018009 | Hs.184601 | chr16q24.3 |
| 201191_at | phosphatidylinositol transfer protein, alpha | PITPNA | H15647 | Hs.461903 | chr17p13.3 |
| 201186_at | low density lipoprotein receptor-related protein associated protein 1 | LRPAP1 | NM_002337 | Hs.75140 | chr4p16.3 |
| 201185_at | HtrA serine peptidase 1 | HTRA1 | NM_002775 | Hs.75111 | chr10q26.3 |
| 201184_s_at | chromodomain helicase DNA binding protein 4 | CHD4 | NM_001273 | Hs.74441 | chr12p13 |
| 201179_s_at | guanine nucleotide binding protein (G protein), alpha inhibiting activity polypeptide 3 | GNAI3 | J03005 | Hs.73799 | chr1p13 |
| 201176_s_at | archain 1 | ARCN1 | NM_001655 | Hs.33642 | chr11q23.3 |
| 201175_at | thioredoxin domain containing 14 | TXNDC14 | NM_015959 | Hs.279861 | chr11cen-q22.3 |
| 201174_s_at | telomeric repeat binding factor 2, interacting protein | TERF2IP | NM_018975 | Hs.274428 | chr16q23.1 |
| 201173_x_at | nuclear distribution gene C homolog (A. nidulans) | NUDC | NM_006600 | Hs.263812 | chr1p35-p34 |
| 201170_s_at | basic helix-loop-helix domain containing, class B, 2 | BHLHB2 | NM_003670 | Hs.171825 | chr3p26 |
| 201167_x_at | Rho GDP dissociation inhibitor (GDI) alpha | ARHGDIA | D13989 | Hs.159161 | chr17q25.3 |
| 201165_s_at | pumilio homolog 1 (Drosophila) | PUM1 | BE670915 | Hs.153834 | chr1p35.2 |
| 201162_at | insulin-like growth factor binding protein 7 | IGFBP7 | NM_001553 | Hs.435795 | chr4q12 |
| 201160_s_at | cold shock domain protein A | CSDA | AL556190 | Hs.221889 | chr12p13.1 |
| 201158_at | N-myristoyltransferase 1 | NMT1 | AI570834 | Hs.346743 | chr17q21.31 |
| 201148_s_at | TIMP metallopeptidase inhibitor 3 (Sorsby fundus dystrophy, pseudoinflammatory) | TIMP3 | AW338933 | Hs.245188 | chr22q12.3 |
| 201145_at | HCLS1 associated protein X-1 | HAX1 | NM_006118 | Hs.199625 | chr1q21.3 |
| 201142_at | eukaryotic translation initiation factor 2, subunit 1 alpha, 35kDa | EIF2S1 | AA577698 | Hs.151777 | chr14q23.3 |
| 201140_s_at | RAB5C, member RAS oncogene family | RAB5C | NM_004583 | Hs.479 | chr17q21.2 |
| 201138_s_at | Sjogren syndrome antigen B (autoantigen La) | SSB | BG532929 | Hs.309316 | chr2q31.1 |
| 201136_at | proteolipid protein 2 (colonic epithelium-enriched) | PLP2 | NM_002668 | Hs.77422 | chrxp11.23 |
| 201135_at | enoyl Coenzyme A hydratase, short chain, 1, mitochondrial | ECHS1 | NM_004092 | Hs.76394 | chr10q26.2-q26.3 |
| 201134_x_at | cytochrome c oxidase subunit VIIc | COX7C | NM_001867 | Hs.430075 | chr5q14 |
| 201133_s_at | praja 2, RING-H2 motif containing | PJA2 | AA142966 | Hs.224262 | chr5q21.3 |
| 201132_at | heterogeneous nuclear ribonucleoprotein H2 (H') | HNRPH2 | NM_019597 | Hs.278857 | chrxq22 |
| 201129_at | splicing factor, arginine/serine-rich 7, 35kDa | SFRS7 | NM_006276 | Hs.309090 | chr2p22.1 |
| 201126_s_at | mannosyl (alpha-1,3-)-glycoprotein beta-1,2-N-acetylglucosaminyltransferase | MGAT1 | NM_002406 | Hs.120870 | chr5q35 |
| 201124_at | integrin, beta 5 | ITGB5 | AL048423 | Hs.149846 | chr3q21.2 |
| 201122_x_at | eukaryotic translation initiation factor 5A | EIF5A | BC000751 | Hs.310621 | chr17p13-p12 |
| 201120_s_at | progesterone receptor membrane component 1 | PGRMC1 | AL547946 | Hs.90061 | chrxq22-q24 |
| 201119_s_at | cytochrome c oxidase subunit 8A (ubiquitous) | COX8A | NM_004074 | Hs.433901 | chr11q12-q13 |
| 201118_at | phosphogluconate dehydrogenase /// phosphogluconate dehydrogenase /// UDP-glucose dehydrogenase /// UDP-glucose dehydrogenase | PGD | NM_002631 | Hs.392837 | chr1p36.3-p36.13 |
| 201117_s_at | carboxypeptidase E | CPE | NM_001873 | Hs.75360 | chr4q32.3 |
| 201115_at | polymerase (DNA directed), delta 2, regulatory subunit 50kDa | POLD2 | NM_006230 | Hs.306791 | chr7p13 |
| 201114_x_at | proteasome (prosome, macropain) subunit, alpha type, 7 | PSMA7 | NM_002792 | Hs.233952 | chr20q13.33 |
| 201113_at | Tu translation elongation factor, mitochondrial | TUFM | NM_003321 | Hs.12084 | chr16p11.2 |
| 201111_at | CSE1 chromosome segregation 1-like (yeast) | CSE1L | AF053641 | Hs.90073 | chr20q13 |
| 201106_at | glutathione peroxidase 4 (phospholipid hydroperoxidase) | GPX4 | NM_002085 | Hs.433951 | chr19p13.3 |
| 201105_at | lectin, galactoside-binding, soluble, 1 (galectin 1) | LGALS1 | NM_002305 | Hs.407909 | chr22q13.1 |
| 201096_s_at | ADP-ribosylation factor 4 | ARF4 | AL537042 | Hs.435639 | chr3p21.2-p21.1 |
| 201095_at | death-associated protein | DAP | NM_004394 | Hs.75189 | chr5p15.2 |
| 201094_at | ribosomal protein S29 | RPS29 | NM_001032 | Hs.539 | chr14q |
| 201092_at | retinoblastoma binding protein 7 | RBBP7 | NM_002893 | Hs.406078 | chrxp22.2 |
| 201090_x_at | alpha tubulin | K-ALPHA-1 | NM_006082 | Hs.446608 | chr12q13.12 |
| 201089_at | ATPase, H+ transporting, lysosomal 56/58kDa, V1 subunit B2 | ATP6V1B2 | NM_001693 | Hs.295917 | chr8p22-p21 |
| 201088_at | karyopherin alpha 2 (RAG cohort 1, importin alpha 1) /// similar to Importin alpha-2 subunit (Karyopherin alpha-2 subunit) (SRP1-alpha) (RAG cohort protein 1) | KPNA2 | NM_002266 | Hs.159557 | chr17q23.1-q23.3 |
| 201085_s_at | SON DNA binding protein | SON | AA664291 | Hs.430541 | chr21q22.11 |
| 201083_s_at | BCL2-associated transcription factor 1 | BCLAF1 | AA740754 | Hs.80338 | chr6q22-q23 |
| 201079_at | synaptogyrin 2 | SYNGR2 | NM_004710 | Hs.433753 | chr17q25.3 |
| 201078_at | transmembrane 9 superfamily member 2 | TM9SF2 | NM_004800 | Hs.298272 | chr13q32.3 |
| 201076_at | NHP2 non-histone chromosome protein 2-like 1 (S. cerevisiae) | NHP2L1 | NM_005008 | Hs.182255 | chr22q13.2-q13.31 |
| 201073_s_at | SWI/SNF related, matrix associated, actin dependent regulator of chromatin, subfamily c, member 1 | SMARCC1 | AL040633 | Hs.162086 | chr3p23-p21 |
| 201070_x_at | splicing factor 3b, subunit 1, 155kDa | SF3B1 | AI739389 | Hs.334826 | chr2q33.1 |
| 201066_at | cytochrome c-1 | CYC1 | NM_001916 | Hs.289271 | chr8q24.3 |
| 201065_s_at | general transcription factor II, i /// general transcription factor II, i, pseudogene 1 /// similar to general transcription factor II, i isoform 1 | GTF2I | NM_001518 | Hs.408507 | chr7q11.23 |
| 201064_s_at | poly(A) binding protein, cytoplasmic 4 (inducible form) | PABPC4 | NM_003819 | Hs.169900 | chr1p32-p36 |
| 201063_at | reticulocalbin 1, EF-hand calcium binding domain | RCN1 | NM_002901 | Hs.167791 | chr11p13 |
| 201060_x_at | stomatin | STOM | AI537887 | Hs.439776 | chr9q34.1 |
| 201056_at | Golgi autoantigen, golgin subfamily b, macrogolgin (with transmembrane signal), 1 | GOLGB1 | N53479 | Hs.445083 | chr3q13 |
| 201053_s_at | proteasome (prosome, macropain) inhibitor subunit 1 (PI31) | PSMF1 | NM_006814 | Hs.437495 | chr20p13 |
| 201050_at | phospholipase D family, member 3 | PLD3 | NM_012268 | Hs.74573 | chr19q13.2 |
| 201049_s_at | ribosomal protein S18 | RPS18 | NM_022551 | Hs.275865 | chr6p21.3 |
| 201047_x_at | RAB6A, member RAS oncogene family | RAB6A | BC003617 | Hs.5636 | chr11q13.3 |
| 201046_s_at | RAD23 homolog A (S. cerevisiae) | RAD23A | NM_005053 | Hs.440960 | chr19p13.2 |
| 201044_x_at | dual specificity phosphatase 1 | DUSP1 | AA530892 | Hs.171695 | chr5q34 |
| 201042_at | transglutaminase 2 (C polypeptide, protein-glutamine-gamma-glutamyltransferase) | TGM2 | AL031651 | Hs.458353 | chr20q12 |
| 201040_at | guanine nucleotide binding protein (G protein), alpha inhibiting activity polypeptide 2 | GNAI2 | NM_002070 | Hs.77269 | chr3p21 |
| 201038_s_at | Acidic (leucine-rich) nuclear phosphoprotein 32 family, member A | ANP32A | T67821 | Hs.124977 | chr15q22.3-q23 |
| 201037_at | phosphofructokinase, platelet | PFKP | NM_002627 | Hs.26010 | chr10p15.3-p15.2 |
| 201033_x_at | ribosomal protein, large, P0 | RPLP0 | NM_001002 | Hs.443796 | chr12q24.2 |
| 201032_at | bladder cancer associated protein | BLCAP | NM_006698 | Hs.5300 | chr20q11.2-q12 |
| 201030_x_at | lactate dehydrogenase B | LDHB | NM_002300 | Hs.234489 | chr12p12.2-p12.1 |
| 201023_at | TAF7 RNA polymerase II, TATA box binding protein (TBP)-associated factor, 55kDa | TAF7 | NM_005642 | Hs.438838 | chr5q31 |
| 201021_s_at | destrin (actin depolymerizing factor) | DSTN | BF697964 | Hs.408576 | chr20p12.1 |
| 201020_at | tyrosine 3-monooxygenase/tryptophan 5-monooxygenase activation protein, eta polypeptide | YWHAH | NM_003405 | Hs.226755 | chr22q12.3 |
| 201016_at | eukaryotic translation initiation factor 1A, X-linked | EIF1AX | BE542684 | Hs.147824 | chrxp22.12 |
| 201015_s_at | junction plakoglobin | JUP | NM_021991 | Hs.2340 | chr17q21 |
| 201012_at | annexin A1 | ANXA1 | NM_000700 | Hs.287558 | chr9q12-q21.2 |
| 201011_at | ribophorin I | RPN1 | NM_002950 | Hs.2280 | chr3q21.3-q25.2 |
| 201008_s_at | thioredoxin interacting protein | TXNIP | AA812232 | Hs.179526 | chr1q21.1 |
| 201007_at | hydroxyacyl-Coenzyme A dehydrogenase/3-ketoacyl-Coenzyme A thiolase/enoyl-Coenzyme A hydratase (trifunctional protein), beta subunit | HADHB | NM_000183 | Hs.269878 | chr2p23 |
| 201005_at | CD9 molecule | CD9 | NM_001769 | Hs.387579 | chr12p13.3 |
| 201004_at | signal sequence receptor, delta (translocon-associated protein delta) | SSR4 | NM_006280 | Hs.409223 | chrxq28 |
| 201003_x_at | arginyl aminopeptidase (aminopeptidase B) /// ubiquitin-conjugating enzyme E2 variant 1 /// ubiquitin-conjugating enzyme variant Kua /// ubiquitin-conjugating enzyme E2 variant 1 /// similar to ubiquitin-conjugating enzyme E2 variant 1 isoform d /// simil | RNPEP | NM_003349 |  |  |
| 201000_at | alanyl-tRNA synthetase | AARS | NM_001605 | Hs.315137 | chr16q22 |
| 200998_s_at | cytoskeleton-associated protein 4 | CKAP4 | AW029619 | Hs.74368 | chr12q23.3 |
| 200996_at | ARP3 actin-related protein 3 homolog (yeast) | ACTR3 | NM_005721 | Hs.433512 | chr2q14.1 |
| 200992_at | importin 7 | IPO7 | AL137335 | Hs.434408 | chr11p15.4 |
| 200991_s_at | sorting nexin 17 | SNX17 | NM_014748 | Hs.278569 | chr2p23-p22 |
| 200990_at | tripartite motif-containing 28 | TRIM28 | NM_005762 | Hs.433612 | chr19q13.4 |
| 200988_s_at | proteasome (prosome, macropain) activator subunit 3 (PA28 gamma; Ki) | PSME3 | NM_005789 | Hs.152978 | chr17q21 |
| 200986_at | serpin peptidase inhibitor, clade G (C1 inhibitor), member 1, (angioedema, hereditary) | SERPING1 | NM_000062 | Hs.384598 | chr11q12-q13.1 |
| 200982_s_at | annexin A6 | ANXA6 | NM_001155 | Hs.412117 | chr5q32-q34 |
| 200979_at | Mitogen-activated protein kinase kinase kinase 15 | MAP3K15 | BF739979 | Hs.471144 | chrXp22.12 |
| 200976_s_at | Tax1 (human T-cell leukemia virus type I) binding protein 1 | TAX1BP1 | NM_006024 | Hs.5437 | chr7p15 |
| 200975_at | palmitoyl-protein thioesterase 1 (ceroid-lipofuscinosis, neuronal 1, infantile) | PPT1 | NM_000310 | Hs.3873 | chr1p32 |
| 200974_at | actin, alpha 2, smooth muscle, aorta | ACTA2 | NM_001613 | Hs.208641 | chr10q23.3 |
| 200972_at | tetraspanin 3 | TSPAN3 | BC000704 | Hs.259212 | chr15q24.3 |
| 200969_at | stress-associated endoplasmic reticulum protein 1 | SERP1 | BG107676 | Hs.439874 | chr3q25.1 |
| 200967_at | peptidylprolyl isomerase B (cyclophilin B) | PPIB | NM_000942 | Hs.434937 | chr15q21-q22 |
| 200966_x_at | aldolase A, fructose-bisphosphate | ALDOA | NM_000034 | Hs.273415 | chr16q22-q24 |
| 200965_s_at | actin binding LIM protein 1 | ABLIM1 | NM_006720 | Hs.442540 | chr10q25 |
| 200964_at | ubiquitin-activating enzyme E1 (A1S9T and BN75 temperature sensitivity complementing) | UBE1 | NM_003334 | Hs.406693 | chrxp11.23 |
| 200962_at | ribosomal protein L31 /// similar to ribosomal protein L31 /// ribosomal protein L31 pseudogene 4 /// ribosomal protein L31 pseudogene 10 /// similar to ribosomal protein L31 /// similar to ribosomal protein L31 /// similar to ribosomal protein L31 /// si | RPL31 | AI348010 | Hs.375921 | chr2q11.2 |
| 200961_at | selenophosphate synthetase 2 | SEPHS2 | NM_012248 | Hs.118725 | chr16p11.2 |
| 200958_s_at | syndecan binding protein (syntenin) | SDCBP | NM_005625 | Hs.164067 | chr8q12 |
| 200956_s_at | structure specific recognition protein 1 | SSRP1 | BE795648 | Hs.79162 | chr11q12 |
| 200955_at | inner membrane protein, mitochondrial (mitofilin) | IMMT | NM_006839 | Hs.271773 | chr2p11.2 |
| 200952_s_at | cyclin D2 | CCND2 | AI635187 | Hs.376071 | chr12p13 |
| 200950_at | actin related protein 2/3 complex, subunit 1A, 41kDa | ARPC1A | NM_006409 | Hs.291981 | chr7q22.1 |
| 200949_x_at | ribosomal protein S20 | RPS20 | NM_001023 | Hs.8102 | chr8q12 |
| 200948_at | myeloid leukemia factor 2 | MLF2 | NM_005439 | Hs.79026 | chr12p13 |
| 200946_x_at | glutamate dehydrogenase 1 | GLUD1 | AI339331 | Hs.355697 | chr10q23.3 |
| 200945_s_at | SEC31 homolog A (S. cerevisiae) | SEC31A | NM_014933 | Hs.436549 | chr4q21.3 |
| 200943_at | high-mobility group nucleosome binding domain 1 | HMGN1 | NM_004965 | Hs.356285 | chr21q22.2 |
| 200941_at | heat shock factor binding protein 1 | HSBP1 | AK026575 | Hs.250899 | chr16q23.3 |
| 200936_at | ribosomal protein L8 | RPL8 | NM_000973 | Hs.178551 | chr8q24.3 |
| 200935_at | calreticulin | CALR | NM_004343 | Hs.433673 | chr19p13.3-p13.2 |
| 200934_at | DEK oncogene (DNA binding) | DEK | NM_003472 | Hs.110713 | chr6p23 |
| 200933_x_at | ribosomal protein S4, X-linked | RPS4X | NM_001007 | Hs.446628 | chrxq13.1 |
| 200932_s_at | dynactin 2 (p50) | DCTN2 | NM_006400 | Hs.289123 | chr12q13.2-q13.3 |
| 200931_s_at | vinculin | VCL | NM_014000 | Hs.75350 | chr10q22.1-q23 |
| 200928_s_at | RAB14, member RAS oncogene family | RAB14 | AL162081 | Hs.5807 | chr9q32-q34.11 |
| 200925_at | cytochrome c oxidase subunit VIa polypeptide 1 | COX6A1 | NM_004373 | Hs.180714 | chr12q24.2 |
| 200924_s_at | solute carrier family 3 (activators of dibasic and neutral amino acid transport), member 2 | SLC3A2 | NM_002394 | Hs.79748 | chr11q13 |
| 200923_at | lectin, galactoside-binding, soluble, 3 binding protein | LGALS3BP | NM_005567 | Hs.79339 | chr17q25 |
| 200920_s_at | B-cell translocation gene 1, anti-proliferative | BTG1 | AL535380 | Hs.255935 | chr12q22 |
| 200919_at | polyhomeotic homolog 2 (Drosophila) | PHC2 | NM_004427 | Hs.165263 | chr1p34.3 |
| 200918_s_at | signal recognition particle receptor ('docking protein') | SRPR | NM_003139 | Hs.444058 | chr11q24.3 |
| 200916_at | transgelin 2 | TAGLN2 | NM_003564 | Hs.406504 | chr1q21-q25 |
| 200914_x_at | kinectin 1 (kinesin receptor) | KTN1 | BF589024 | Hs.368212 | chr14q22.1 |
| 200913_at | protein phosphatase 1G (formerly 2C), magnesium-dependent, gamma isoform | PPM1G | NM_002707 | Hs.17883 | chr2p23.3 |
| 200912_s_at | eukaryotic translation initiation factor 4A, isoform 2 | EIF4A2 | NM_001967 | Hs.511904 | chr3q28 |
| 200910_at | chaperonin containing TCP1, subunit 3 (gamma) | CCT3 | NM_005998 | Hs.1708 | chr1q23 |
| 200908_s_at | ribosomal protein, large, P2 | RPLP2 | BC005354 |  |  |
| 200904_at | major histocompatibility complex, class I, E | HLA-E | X56841 | Hs.381008 | chr6p21.3 |
| 200903_s_at | S-adenosylhomocysteine hydrolase | AHCY | NM_000687 | Hs.388004 | chr20cen-q13.1 |
| 200902_at | 15 kDa selenoprotein | 15-sept. | NM_004261 | Hs.90606 | chr1p31 |
| 200901_s_at | mannose-6-phosphate receptor (cation dependent) | M6PR | NM_002355 | Hs.134084 | chr12p13 |
| 200897_s_at | palladin, cytoskeletal associated protein | PALLD | NM_016081 | Hs.194431 | chr4q32.3 |
| 200896_x_at | hepatoma-derived growth factor (high-mobility group protein 1-like) | HDGF | NM_004494 | Hs.89525 | chr1q21-q23 |
| 200894_s_at | FK506 binding protein 4, 59kDa | FKBP4 | AA894574 | Hs.848 | chr12p13.33 |
| 200887_s_at | signal transducer and activator of transcription 1, 91kDa | STAT1 | NM_007315 | Hs.21486 | chr2q32.2 |
| 200886_s_at | phosphoglycerate mutase 1 (brain) /// similar to Phosphoglycerate mutase 1 (Phosphoglycerate mutase isozyme B) (PGAM-B) (BPG-dependent PGAM 1) /// similar to Phosphoglycerate mutase 1 (Phosphoglycerate mutase isozyme B) (PGAM-B) (BPG-dependent PGAM 1) | PGAM1 | NM_002629 | Hs.447492 | chr10q25.3 |
| 200885_at | ras homolog gene family, member C | RHOC | NM_005167 | Hs.179735 | chr1p13.1 |
| 200884_at | creatine kinase, brain | CKB | NM_001823 | Hs.173724 | chr14q32 |
| 200883_at | ubiquinol-cytochrome c reductase core protein II | UQCRC2 | NM_003366 | Hs.173554 | chr16p12 |
| 200882_s_at | proteasome (prosome, macropain) 26S subunit, non-ATPase, 4 | PSMD4 | NM_002810 | Hs.505059 | chr1q21.2 |
| 200880_at | DnaJ (Hsp40) homolog, subfamily A, member 1 | DNAJA1 | AL534104 |  |  |
| 200878_at | endothelial PAS domain protein 1 | EPAS1 | AF052094 | Hs.8136 | chr2p21-p16 |
| 200877_at | chaperonin containing TCP1, subunit 4 (delta) | CCT4 | NM_006430 | Hs.374334 | chr2p15 |
| 200876_s_at | proteasome (prosome, macropain) subunit, beta type, 1 | PSMB1 | NM_002793 | Hs.352768 | chr6q27 |
| 200874_s_at | nucleolar protein 5A (56kDa with KKE/D repeat) | NOL5A | BE796327 | Hs.376064 | chr20p13 |
| 200873_s_at | chaperonin containing TCP1, subunit 8 (theta) | CCT8 | NM_006585 | Hs.416211 | chr21q22.11 |
| 200868_s_at | zinc finger protein 313 | ZNF313 | NM_018683 | Hs.144949 | chr20q13.13 |
| 200866_s_at | prosaposin (variant Gaucher disease and variant metachromatic leukodystrophy) | PSAP | M32221 | Hs.406455 | chr10q21-q22 |
| 200863_s_at | RAB11A, member RAS oncogene family | RAB11A | AI215102 | Hs.75618 | chr15q21.3-q22.31 |
| 200862_at | 24-dehydrocholesterol reductase | DHCR24 | NM_014762 | Hs.75616 | chr1p33-p31.1 |
| 200861_at | CCR4-NOT transcription complex, subunit 1 | CNOT1 | NM_016284 | Hs.279949 | chr16q21 |
| 200859_x_at | filamin A, alpha (actin binding protein 280) | FLNA | NM_001456 | Hs.195464 | chrxq28 |
| 200858_s_at | ribosomal protein S8 | RPS8 | NM_001012 | Hs.512675 | chr1p34.1-p32 |
| 200855_at | nuclear receptor co-repressor 1 /// chromosome 20 open reading frame 191 | NCOR1 | AW771910 | Hs.144904 | chr17p11.2 |
| 200853_at | H2A histone family, member Z | H2AFZ | NM_002106 | Hs.119192 | chr4q24 |
| 200852_x_at | guanine nucleotide binding protein (G protein), beta polypeptide 2 | GNB2 | NM_005273 | Hs.185172 | chr7q22 |
| 200851_s_at | KIAA0174 | KIAA0174 | NM_014761 | Hs.287613 | chr16q22.2 |
| 200848_at | S-adenosylhomocysteine hydrolase-like 1 | AHCYL1 | AA479488 | Hs.4113 | chr1p13.2 |
| 200847_s_at | transmembrane protein 66 | TMEM66 | NM_016127 | Hs.279921 | chr8p12 |
| 200846_s_at | protein phosphatase 1, catalytic subunit, alpha isoform | PPP1CA | NM_002708 | Hs.240457 | chr11q13 |
| 200844_s_at | peroxiredoxin 6 | PRDX6 | BE869583 | Hs.120 | chr1q25.1 |
| 200841_s_at | glutamyl-prolyl-tRNA synthetase | EPRS | AI142677 | Hs.171292 | chr1q41-q42 |
| 200838_at | cathepsin B | CTSB | NM_001908 | Hs.135226 | chr8p22 |
| 200837_at | B-cell receptor-associated protein 31 | BCAP31 | NM_005745 | Hs.381232 | chrxq28 |
| 200833_s_at | RAP1B, member of RAS oncogene family | RAP1B | NM_015646 | Hs.374418 | chr12q14 |
| 200830_at | proteasome (prosome, macropain) 26S subunit, non-ATPase, 2 | PSMD2 | NM_002808 | Hs.388921 | chr3q27.1 |
| 200827_at | procollagen-lysine 1, 2-oxoglutarate 5-dioxygenase 1 | PLOD1 | NM_000302 | Hs.75093 | chr1p36.3-p36.2 |
| 200826_at | small nuclear ribonucleoprotein D2 polypeptide 16.5kDa | SNRPD2 | NM_004597 | Hs.424327 | chr19q13.2 |
| 200825_s_at | hypoxia up-regulated 1 | HYOU1 | NM_006389 | Hs.277704 | chr11q23.1-q23.3 |
| 200824_at | glutathione S-transferase pi | GSTP1 | NM_000852 | Hs.411509 | chr11q13 |
| 200823_x_at | ribosomal protein L29 | RPL29 | NM_000992 | Hs.430207 | chr3p21.3-p21.2 |
| 200820_at | proteasome (prosome, macropain) 26S subunit, non-ATPase, 8 | PSMD8 | NM_002812 | Hs.78466 | chr19q13.2 |
| 200819_s_at | ribosomal protein S15 | RPS15 | NM_001018 | Hs.406683 | chr19p13.3 |
| 200818_at | ATP synthase, H+ transporting, mitochondrial F1 complex, O subunit (oligomycin sensitivity conferring protein) | ATP5O | NM_001697 | Hs.409140 | chr21q22.11 |
| 200814_at | proteasome (prosome, macropain) activator subunit 1 (PA28 alpha) | PSME1 | NM_006263 | Hs.75348 | chr14q11.2 |
| 200813_s_at | platelet-activating factor acetylhydrolase, isoform Ib, alpha subunit 45kDa | PAFAH1B1 | BE256969 | Hs.77318 | chr17p13.3 |
| 200812_at | chaperonin containing TCP1, subunit 7 (eta) | CCT7 | NM_006429 | Hs.368149 | chr2p13.2 |
| 200810_s_at | cold inducible RNA binding protein | CIRBP | NM_001280 | Hs.515010 | chr19p13.3 |
| 200806_s_at | heat shock 60kDa protein 1 (chaperonin) | HSPD1 | BE256479 | Hs.79037 | chr2q33.1 |
| 200805_at | lectin, mannose-binding 2 | LMAN2 | NM_006816 | Hs.75864 | chr5q35.3 |
| 200803_s_at | testis enhanced gene transcript (BAX inhibitor 1) | TEGT | AF033095 | Hs.35052 | chr12q12-q13 |
| 200802_at | seryl-tRNA synthetase | SARS | NM_006513 | Hs.444261 | chr1p13.3-p13.1 |
| 200799_at | heat shock 70kDa protein 1A | HSPA1A | NM_005345 | Hs.75452 | chr6p21.3 |
| 200796_s_at | myeloid cell leukemia sequence 1 (BCL2-related) | MCL1 | BF594446 | Hs.86386 | chr1q21 |
| 200793_s_at | aconitase 2, mitochondrial | ACO2 | NM_001098 | Hs.202505 | chr22q13.2-q13.31 |
| 200792_at | X-ray repair complementing defective repair in Chinese hamster cells 6 (Ku autoantigen, 70kDa) | XRCC6 | NM_001469 | Hs.169744 | chr22q13.2-q13.31 |
| 200791_s_at | IQ motif containing GTPase activating protein 1 | IQGAP1 | NM_003870 | Hs.1742 | chr15q26.1 |
| 200790_at | ornithine decarboxylase 1 | ODC1 | NM_002539 | Hs.443409 | chr2p25 |
| 200789_at | enoyl Coenzyme A hydratase 1, peroxisomal | ECH1 | NM_001398 | Hs.196176 | chr19q13.1 |
| 200787_s_at | phosphoprotein enriched in astrocytes 15 | PEA15 | BC002426 | Hs.194673 | chr1q21.1 |
| 200786_at | proteasome (prosome, macropain) subunit, beta type, 7 | PSMB7 | NM_002799 | Hs.197071 | chr9q34.11-q34.12 |
| 200783_s_at | stathmin 1/oncoprotein 18 | STMN1 | NM_005563 | Hs.209983 | chr1p36.1-p35 |
| 200782_at | annexin A5 | ANXA5 | NM_001154 | Hs.145741 | chr4q28-q32 |
| 200781_s_at | ribosomal protein S15a | RPS15A | NM_001019 | Hs.370504 | chr16p /// chr16p12-p11.2 |
| 200780_x_at | fibrinogen beta chain /// GNAS complex locus | FGB | NM_000516 | Hs.157307 | chr20q13.2-q13.3 |
| 200779_at | activating transcription factor 4 (tax-responsive enhancer element B67) | ATF4 | NM_001675 | Hs.181243 | chr22q13.1 |
| 200776_s_at | basic leucine zipper and W2 domains 1 /// similar to basic leucine zipper and W2 domains 1 | BZW1 | AL518328 | Hs.355983 | chr2q33 |
| 200770_s_at | laminin, gamma 1 (formerly LAMB2) | LAMC1 | J03202 | Hs.432855 | chr1q31 |
| 200769_s_at | methionine adenosyltransferase II, alpha | MAT2A | NM_005911 | Hs.77502 | chr2p11.2 |
| 200766_at | cathepsin D | CTSD | NM_001909 | Hs.121575 | chr11p15.5 |
| 200763_s_at | ribosomal protein, large, P1 | RPLP1 | NM_001003 | Hs.356502 | chr15q22 |
| 200762_at | dihydropyrimidinase-like 2 | DPYSL2 | NM_001386 | Hs.173381 | chr8p22-p21 |
| 200760_s_at | ADP-ribosylation-like factor 6 interacting protein 5 | ARL6IP5 | N92494 | Hs.92384 | chr3p14 |
| 200758_s_at | nuclear factor (erythroid-derived 2)-like 1 | NFE2L1 | AI361227 | Hs.83469 | chr17q21.3 |
| 200756_x_at | calumenin | CALU | U67280 | Hs.7753 | chr7q32 |
| 200753_x_at | splicing factor, arginine/serine-rich 2 | SFRS2 | BE866585 | Hs.73965 | chr17q25.1 |
| 200752_s_at | calpain 1, (mu/I) large subunit | CAPN1 | NM_005186 | Hs.356181 | chr11q13 |
| 200749_at | RAN, member RAS oncogene family | RAN | BF112006 | Hs.10842 | chr12q24.3 |
| 200748_s_at | ferritin, heavy polypeptide 1 | FTH1 | NM_002032 | Hs.132319 | chr11q13 |
| 200747_s_at | nuclear mitotic apparatus protein 1 | NUMA1 | NM_006185 | Hs.523873 | chr11q13 |
| 200739_s_at | SMT3 suppressor of mif two 3 homolog 3 (S. cerevisiae) | SUMO3 | BG338532 | Hs.85119 | chr21q22.3 |
| 200737_at | phosphoglycerate kinase 1 | PGK1 | NM_000291 | Hs.78771 | chrxq13 |
| 200736_s_at | glutathione peroxidase 1 | GPX1 | NM_000581 | Hs.76686 | chr3p21.3 |
| 200735_x_at | nascent-polypeptide-associated complex alpha polypeptide | NACA | NM_005594 | Hs.32916 | chr12q23-q24.1 |
| 200730_s_at | protein tyrosine phosphatase type IVA, member 1 | PTP4A1 | BF576710 | Hs.227777 | chr6q12 |
| 200726_at | protein phosphatase 1, catalytic subunit, gamma isoform | PPP1CC | NM_002710 | Hs.79081 | chr12q24.1-q24.2 |
| 200721_s_at | ARP1 actin-related protein 1 homolog A, centractin alpha (yeast) | ACTR1A | NM_005736 | Hs.153961 | chr10q24.32 |
| 200719_at | S-phase kinase-associated protein 1A (p19A) | SKP1A | BE964043 | Hs.171626 | chr5q31 |
| 200717_x_at | ribosomal protein L7 | RPL7 | NM_000971 | Hs.421257 | chr8q21.11 |
| 200715_x_at | ribosomal protein L13a | RPL13A | BC000514 | Hs.449070 | chr19q13.3 |
| 200714_x_at | amplified in osteosarcoma | OS9 | NM_006812 | Hs.76228 | chr12q13 |
| 200712_s_at | microtubule-associated protein, RP/EB family, member 1 | MAPRE1 | AI633566 | Hs.408754 | chr20q11.1-11.23 |
| 200710_at | acyl-Coenzyme A dehydrogenase, very long chain | ACADVL | NM_000018 | Hs.437178 | chr17p13-p11 |
| 200708_at | glutamic-oxaloacetic transaminase 2, mitochondrial (aspartate aminotransferase 2) | GOT2 | NM_002080 | Hs.314658 | chr16q21 |
| 200707_at | protein kinase C substrate 80K-H | PRKCSH | NM_002743 | Hs.512640 | chr19p13.2 |
| 200706_s_at | lipopolysaccharide-induced TNF factor | LITAF | NM_004862 | Hs.76507 | chr16p13.3-p12 |
| 200705_s_at | eukaryotic translation elongation factor 1 beta 2 | EEF1B2 | NM_001959 | Hs.421608 | chr2q33-q34 |
| 200703_at | dynein, light chain, LC8-type 1 | DYNLL1 | NM_003746 | Hs.5120 | chr12q24.23 |
| 200702_s_at | DEAD (Asp-Glu-Ala-Asp) box polypeptide 24 | DDX24 | BG421209 | Hs.372267 | chr14q32 |
| 200701_at | Niemann-Pick disease, type C2 | NPC2 | NM_006432 | Hs.433222 | chr14q24.3 |
| 200697_at | hexokinase 1 | HK1 | NM_000188 | Hs.118625 | chr10q22 |
| 200696_s_at | gelsolin (amyloidosis, Finnish type) | GSN | NM_000177 | Hs.446537 | chr9q33 |
| 200695_at | protein phosphatase 2 (formerly 2A), regulatory subunit A (PR 65), alpha isoform | PPP2R1A | NM_014225 | Hs.173902 | chr19q13.41 |
| 200693_at | tyrosine 3-monooxygenase/tryptophan 5-monooxygenase activation protein, theta polypeptide | YWHAQ | NM_006826 | Hs.74405 | chr2p25.1 |
| 200690_at | heat shock 70kDa protein 9 (mortalin) | HSPA9 | AA927701 | Hs.184233 | chr5q31.1 |
| 200689_x_at | eukaryotic translation elongation factor 1 gamma /// similar to Elongation factor 1-gamma (EF-1-gamma) (eEF-1B gamma) | EEF1G | NM_001404 | Hs.256184 | chr11q12.3 |
| 200687_s_at | splicing factor 3b, subunit 3, 130kDa | SF3B3 | NM_012426 | Hs.195614 | chr16q22.1 |
| 200681_at | glyoxalase I | GLO1 | NM_006708 | Hs.268849 | chr6p21.3-p21.1 |
| 200677_at | pituitary tumor-transforming 1 interacting protein | PTTG1IP | NM_004339 | Hs.369026 | chr21q22.3 |
| 200676_s_at | ubiquitin-conjugating enzyme E2L 3 | UBE2L3 | NM_003347 | Hs.108104 | chr22q11.21 |
| 200675_at | CD81 molecule | CD81 | NM_004356 | Hs.54457 | chr11p15.5 |
| 200674_s_at | ribosomal protein L32 | RPL32 | NM_000994 | Hs.265174 | chr3p25-p24 |
| 200673_at | lysosomal-associated protein transmembrane 4 alpha | LAPTM4A | NM_014713 | Hs.111894 | chr2p24.1 |
| 200670_at | X-box binding protein 1 | XBP1 | NM_005080 | Hs.437638 | chr22q12.1 |
| 200665_s_at | secreted protein, acidic, cysteine-rich (osteonectin) /// secreted protein, acidic, cysteine-rich (osteonectin) | SPARC | NM_003118 | Hs.111779 | chr5q31.3-q32 |
| 200664_s_at | DnaJ (Hsp40) homolog, subfamily B, member 1 | DNAJB1 | BG537255 | Hs.82646 | chr19p13.2 |
| 200663_at | CD63 molecule | CD63 | NM_001780 | Hs.445570 | chr12q12-q13 |
| 200662_s_at | translocase of outer mitochondrial membrane 20 homolog (yeast) | TOMM20 | NM_014765 | Hs.75187 | chr1q42 |
| 200661_at | cathepsin A | CTSA | NM_000308 | Hs.118126 | chr20q13.1 |
| 200660_at | S100 calcium binding protein A11 | S100A11 | NM_005620 | Hs.417004 | chr1q21 |
| 200659_s_at | prohibitin | PHB | NM_002634 | Hs.75323 | chr17q21 |
| 200657_at | solute carrier family 25 (mitochondrial carrier; adenine nucleotide translocator), member 5 | SLC25A5 | NM_001152 | Hs.79172 | chrxq24-q26 |
| 200652_at | signal sequence receptor, beta (translocon-associated protein beta) | SSR2 | NM_003145 | Hs.74564 | chr1q21-q23 |
| 200650_s_at | lactate dehydrogenase A | LDHA | NM_005566 | Hs.2795 | chr11p15.4 |
| 200647_x_at | eukaryotic translation initiation factor 3, subunit 8, 110kDa /// similar to eukaryotic translation initiation factor 3, subunit 8, 110kDa | EIF3S8 | NM_003752 | Hs.534402 | chr16p11.2 |
| 200646_s_at | nucleobindin 1 | NUCB1 | NM_006184 | Hs.172609 | chr19q13.2-q13.4 |
| 200645_at | GABA(A) receptor-associated protein | GABARAP | NM_007278 | Hs.534435 | chr17p13.1 |
| 200644_at | MARCKS-like 1 | MARCKSL1 | NM_023009 | Hs.75061 | chr1p35.1 |
| 200642_at | superoxide dismutase 1, soluble (amyotrophic lateral sclerosis 1 (adult)) | SOD1 | NM_000454 | Hs.443914 | chr21q22.11 |
| 200638_s_at | tyrosine 3-monooxygenase/tryptophan 5-monooxygenase activation protein, zeta polypeptide | YWHAZ | BC003623 | Hs.386834 | chr8q23.1 |
| 200637_s_at | protein tyrosine phosphatase, receptor type, F | PTPRF | AI762627 | Hs.75216 | chr1p34 |
| 200634_at | profilin 1 | PFN1 | NM_005022 | Hs.408943 | chr17p13.3 |
| 200633_at | ubiquitin B /// ubiquitin B | UBB | NM_018955 | Hs.356190 | chr17p12-p11.2 |
| 200632_s_at | N-myc downstream regulated gene 1 | NDRG1 | NM_006096 | Hs.318567 | chr8q24.3 |
| 200630_x_at | SET translocation (myeloid leukemia-associated) | SET | AV702810 | Hs.436687 | chr9q34 |
| 200629_at | tryptophanyl-tRNA synthetase | WARS | NM_004184 | Hs.82030 | chr14q32.31 |
| 200627_at | prostaglandin E synthase 3 (cytosolic) | PTGES3 | BC003005 | Hs.355693 | chr12q13.3 |
| 200625_s_at | CAP, adenylate cyclase-associated protein 1 (yeast) | CAP1 | NM_006367 | Hs.104125 | chr1p34.2 |
| 200621_at | cysteine and glycine-rich protein 1 | CSRP1 | NM_004078 | Hs.108080 | chr1q32 |
| 200620_at | transmembrane protein 59 | TMEM59 | NM_004872 | Hs.416495 | chr1p36-p31 |
| 200619_at | splicing factor 3b, subunit 2, 145kDa | SF3B2 | NM_006842 | Hs.406423 | chr11q13.1 |
| 200618_at | LIM and SH3 protein 1 | LASP1 | NM_006148 | Hs.334851 | chr17q11-q21.3 |
| 200616_s_at | KIAA0152 | KIAA0152 | BC000371 | Hs.181418 | chr12q24.31 |
| 200615_s_at | adaptor-related protein complex 2, beta 1 subunit | AP2B1 | AL567295 | Hs.370123 | chr17q11.2-q12 |
| 200614_at | clathrin, heavy chain (Hc) | CLTC | NM_004859 | Hs.187416 | chr17q11-qter |
| 200613_at | adaptor-related protein complex 2, mu 1 subunit | AP2M1 | NM_004068 | Hs.433892 | chr3q28 |
| 200610_s_at | nucleolin | NCL | NM_005381 | Hs.79110 | chr2q12-qter |
| 200609_s_at | WD repeat domain 1 | WDR1 | NM_017491 | Hs.85100 | chr4p16.1 |
| 200607_s_at | RAD21 homolog (S. pombe) | RAD21 | BG289967 | Hs.81848 | chr8q24 |
| 200603_at | protein kinase, cAMP-dependent, regulatory, type I, alpha (tissue specific extinguisher 1) | PRKAR1A | AL050038 | Hs.280342 | chr17q23-q24 |
| 200601_at | actinin, alpha 4 | ACTN4 | U48734 | Hs.374303 | chr19q13 |
| 200600_at | moesin | MSN | NM_002444 | Hs.170328 | chrxq11.2-q12 |
| 200598_s_at | heat shock protein 90kDa beta (Grp94), member 1 | HSP90B1 | AI582238 | Hs.192374 | chr12q24.2-q24.3 |
| 200597_at | eukaryotic translation initiation factor 3, subunit 10 theta, 150/170kDa | EIF3S10 | AI123320 | Hs.389559 | chr10q26 |
| 200097_s_at | heterogeneous nuclear ribonucleoprotein K /// heterogeneous nuclear ribonucleoprotein K | HNRPK | AI701949 | Hs.307544 | chr9q21.32-q21.33 |
| 200095_x_at | ribosomal protein S10 /// ribosomal protein S10 | RPS10 | AA320764 | Hs.406620 | chr6p21.31 |
| 200094_s_at | eukaryotic translation elongation factor 2 /// eukaryotic translation elongation factor 2 | EEF2 | AI004246 | Hs.75309 | chr19pter-q12 |
| 200091_s_at | ribosomal protein S25 /// ribosomal protein S25 | RPS25 | AA888388 | Hs.512676 | chr11q23.3 |
| 200089_s_at | ribosomal protein L4 /// ribosomal protein L4 | RPL4 | AI953886 | Hs.186350 | chr15q22 |
| 200088_x_at | ribosomal protein L12 /// ribosomal protein L12 | RPL12 | AK026491 | Hs.408054 | chr9q34 |
| 200086_s_at | cytochrome c oxidase subunit IV isoform 1 /// cytochrome c oxidase subunit IV isoform 1 | COX4I1 | AA854966 | Hs.433419 | chr16q22-qter |
| 200085_s_at | transcription elongation factor B (SIII), polypeptide 2 (18kDa, elongin B) /// transcription elongation factor B (SIII), polypeptide 2 (18kDa, elongin B) | TCEB2 | NM_007108 | Hs.172772 | chr16p12.3 |
| 200084_at | chromosome 11 open reading frame 58 /// chromosome 11 open reading frame 58 | C11orf58 | BE748698 | Hs.78050 | chr11p15.1 |
| 200083_at | ubiquitin specific peptidase 22 /// ubiquitin specific peptidase 22 | USP22 | AA621731 | Hs.12064 | chr17p11.2 |
| 200079_s_at | lysyl-tRNA synthetase /// lysyl-tRNA synthetase | KARS | AF285758 | Hs.3100 | chr16q23-q24 |
| 200078_s_at | ATPase, H+ transporting, lysosomal 21kDa, V0 subunit b /// ATPase, H+ transporting, lysosomal 21kDa, V0 subunit b | ATP6V0B | BC005876 | Hs.7476 | chr1p32.3 |
| 200077_s_at | ornithine decarboxylase antizyme 1 /// ornithine decarboxylase antizyme 1 | OAZ1 | D87914 | Hs.446427 | chr19p13.3 |
| 200076_s_at | chromosome 19 open reading frame 50 /// chromosome 19 open reading frame 50 | C19orf50 | BC006479 | Hs.369785 | chr19p13.11 |
| 200075_s_at | guanylate kinase 1 /// guanylate kinase 1 | GUK1 | BC006249 | Hs.376933 | chr1q32-q41 |
| 200071_at | survival motor neuron domain containing 1 /// survival motor neuron domain containing 1 | SMNDC1 | BF224259 | Hs.79968 | chr10q23 |
| 200070_at | chromosome 2 open reading frame 24 /// chromosome 2 open reading frame 24 | C2orf24 | BC001393 | Hs.4973 | chr2q35 |
| 200069_at | squamous cell carcinoma antigen recognized by T cells 3 /// squamous cell carcinoma antigen recognized by T cells 3 | SART3 | AI656011 |  |  |
| 200067_x_at | sorting nexin 3 /// sorting nexin 3 | SNX3 | AL078596 |  |  |
| 200066_at | IK cytokine, down-regulator of HLA II /// IK cytokine, down-regulator of HLA II | IK | AF182645 | Hs.421245 | chr5q31.3 |
| 200063_s_at | nucleophosmin (nucleolar phosphoprotein B23, numatrin) /// nucleophosmin (nucleolar phosphoprotein B23, numatrin) | NPM1 | BC002398 | Hs.411098 | chr5q35 |
| 200062_s_at | ribosomal protein L30 /// ribosomal protein L30 | RPL30 | L05095 | Hs.400295 | chr8q22 |
| 200060_s_at | RNA binding protein S1, serine-rich domain /// RNA binding protein S1, serine-rich domain | RNPS1 | BC001659 | Hs.355643 | chr16p13.3 |
| 200058_s_at | activating signal cointegrator 1 complex subunit 3-like 1 /// activating signal cointegrator 1 complex subunit 3-like 1 | ASCC3L1 | BC001417 | Hs.246112 | chr2q11.2 |
| 200057_s_at | non-POU domain containing, octamer-binding /// non-POU domain containing, octamer-binding | NONO | NM_007363 | Hs.355861 | chrxq13.1 |
| 200056_s_at | nuclear DNA-binding protein /// nuclear DNA-binding protein | C1D | NM_006333 | Hs.380759 | chr2p13-p12 |
| 200055_at | TAF10 RNA polymerase II, TATA box binding protein (TBP)-associated factor, 30kDa /// TAF10 RNA polymerase II, TATA box binding protein (TBP)-associated factor, 30kDa | TAF10 | NM_006284 | Hs.534377 | chr11p15.3 |
| 200053_at | sperm associated antigen 7 /// sperm associated antigen 7 | SPAG7 | NM_004890 | Hs.90436 | chr17p13.2 |
| 200052_s_at | interleukin enhancer binding factor 2, 45kDa /// interleukin enhancer binding factor 2, 45kDa | ILF2 | NM_004515 | Hs.75117 | chr1q21.3 |
| 200051_at | squamous cell carcinoma antigen recognized by T cells /// squamous cell carcinoma antigen recognized by T cells | SART1 | NM_005146 | Hs.433004 | chr11q13.1 |
| 200049_at | MYST histone acetyltransferase 2 /// MYST histone acetyltransferase 2 | MYST2 | NM_007067 | Hs.21907 | chr17q21.32 |
| 200048_s_at | jumping translocation breakpoint /// jumping translocation breakpoint | JTB | NM_006694 | Hs.6396 | chr1q21 |
| 200047_s_at | YY1 transcription factor /// YY1 transcription factor | YY1 | NM_003403 | Hs.388927 | chr14q |
| 200046_at | defender against cell death 1 /// defender against cell death 1 | DAD1 | NM_001344 | Hs.82890 | chr14q11-q12 |
| 200045_at | ATP-binding cassette, sub-family F (GCN20), member 1 /// ATP-binding cassette, sub-family F (GCN20), member 1 | ABCF1 | NM_001090 | Hs.9573 | chr6p21.33 |
| 200044_at | splicing factor, arginine/serine-rich 9 /// splicing factor, arginine/serine-rich 9 | SFRS9 | NM_003769 | Hs.369624 | chr12q24.31 |
| 200043_at | enhancer of rudimentary homolog (Drosophila) /// enhancer of rudimentary homolog (Drosophila) | ERH | NM_004450 | Hs.433413 | chr14q24.1 |
| 200042_at | chromosome 22 open reading frame 28 /// chromosome 22 open reading frame 28 | C22orf28 | NM_014306 | Hs.273213 | chr22q12 |
| 200041_s_at | HLA-B associated transcript 1 /// HLA-B associated transcript 1 | BAT1 | NM_004640 | Hs.254042 | chr6p21.3 |
| 200040_at | KH domain containing, RNA binding, signal transduction associated 1 /// KH domain containing, RNA binding, signal transduction associated 1 | KHDRBS1 | NM_006559 | Hs.119537 | chr1p32 |
| 200039_s_at | proteasome (prosome, macropain) subunit, beta type, 2 /// proteasome (prosome, macropain) subunit, beta type, 2 | PSMB2 | NM_002794 | Hs.432607 | chr1p34.2 |
| 200038_s_at | ribosomal protein L17 /// ribosomal protein L17 | RPL17 | NM_000985 | Hs.374588 | chr18q21 |
| 200036_s_at | ribosomal protein L10a /// ribosomal protein L10a | RPL10A | NM_007104 | Hs.448396 | chr6p21.3-p21.2 |
| 200035_at | dullard homolog (Xenopus laevis) /// dullard homolog (Xenopus laevis) | DULLARD | NM_015343 | Hs.84359 | chr17p13 |
| 200034_s_at | ribosomal protein L6 /// ribosomal protein L6 | RPL6 | NM_000970 | Hs.416566 | chr12q24.1 |
| 200033_at | DEAD (Asp-Glu-Ala-Asp) box polypeptide 5 /// DEAD (Asp-Glu-Ala-Asp) box polypeptide 5 | DDX5 | NM_004396 | Hs.279806 | chr17q21 |
| 200032_s_at | ribosomal protein L9 /// ribosomal protein L9 | RPL9 | NM_000661 | Hs.412370 | chr4p13 |
| 200031_s_at | ribosomal protein S11 /// ribosomal protein S11 | RPS11 | NM_001015 | Hs.433529 | chr19q13.3 |
| 200030_s_at | solute carrier family 25 (mitochondrial carrier; phosphate carrier), member 3 /// solute carrier family 25 (mitochondrial carrier; phosphate carrier), member 3 | SLC25A3 | NM_002635 | Hs.290404 | chr12q23 |
| 200029_at | ribosomal protein L19 /// ribosomal protein L19 | RPL19 | NM_000981 | Hs.381061 | chr17q11.2-q12 |
| 200028_s_at | START domain containing 7 /// START domain containing 7 | STARD7 | NM_020151 | Hs.445446 | chr2q11.2 |
| 200027_at | asparaginyl-tRNA synthetase /// asparaginyl-tRNA synthetase | NARS | NM_004539 | Hs.427212 | chr18q21.2-q21.3 |
| 200026_at | ribosomal protein L34 /// ribosomal protein L34 /// similar to ribosomal protein L34 /// similar to ribosomal protein L34 /// similar to ribosomal protein L34 /// similar to ribosomal protein L34 /// hypothetical protein LOC729536 /// hypothetical protein | RPL34 | NM_000995 | Hs.250895 | chr4q25 |
| 200025_s_at | ribosomal protein L27 /// ribosomal protein L27 | RPL27 | NM_000988 | Hs.405528 | chr17q21.1-q21.2 |
| 200024_at | ribosomal protein S5 /// ribosomal protein S5 /// similar to ribosomal protein S5 /// similar to ribosomal protein S5 | RPS5 | NM_001009 |  |  |
| 200023_s_at | eukaryotic translation initiation factor 3, subunit 5 epsilon, 47kDa /// eukaryotic translation initiation factor 3, subunit 5 epsilon, 47kDa | EIF3S5 | NM_003754 | Hs.381255 | chr11p15.4 |
| 200022_at | ribosomal protein L18 /// ribosomal protein L18 | RPL18 | NM_000979 | Hs.409634 | chr19q13 |
| 200019_s_at | Finkel-Biskis-Reilly murine sarcoma virus (FBR-MuSV) ubiquitously expressed (fox derived); ribosomal protein S30 /// Finkel-Biskis-Reilly murine sarcoma virus (FBR-MuSV) ubiquitously expressed (fox derived); ribosomal protein S30 | FAU | NM_001997 | Hs.387208 | chr11q13 |
| 200018_at | ribosomal protein S13 /// ribosomal protein S13 | RPS13 | NM_001017 | Hs.446588 | chr11p15 |
| 200013_at | ribosomal protein L24 /// ribosomal protein L24 | RPL24 | NM_000986 | Hs.184582 | chr3q12 |
| 200012_x_at | ribosomal protein L21 /// ribosomal protein L21 /// similar to ribosomal protein L21 /// similar to ribosomal protein L21 /// similar to 60S ribosomal protein L21 /// similar to 60S ribosomal protein L21 /// similar to 60S ribosomal protein L21 /// simila | RPL21 | NM_000982 | Hs.381123 | chr13q12.2 |
| 200011_s_at | ADP-ribosylation factor 3 /// ADP-ribosylation factor 3 | ARF3 | NM_001659 | Hs.119177 | chr12q13 |
| 200010_at | Ribosomal protein L11 /// Ribosomal protein L11 | RPL11 | NM_000975 | Hs.388664 | chr1p36.1-p35 |
| 200008_s_at | GDP dissociation inhibitor 2 /// GDP dissociation inhibitor 2 | GDI2 | D13988 | Hs.56845 | chr10p15 |
| 200007_at | signal recognition particle 14kDa (homologous Alu RNA binding protein) /// signal recognition particle 14kDa (homologous Alu RNA binding protein) | SRP14 | NM_003134 | Hs.180394 | chr15q22 |
| 200006_at | Parkinson disease (autosomal recessive, early onset) 7 /// Parkinson disease (autosomal recessive, early onset) 7 | PARK7 | NM_007262 | Hs.419640 | chr1p36.33-p36.12 |
| 200005_at | eukaryotic translation initiation factor 3, subunit 7 zeta, 66/67kDa /// eukaryotic translation initiation factor 3, subunit 7 zeta, 66/67kDa | EIF3S7 | NM_003753 | Hs.55682 | chr22q13.1 |
| 200004_at | eukaryotic translation initiation factor 4 gamma, 2 /// eukaryotic translation initiation factor 4 gamma, 2 | EIF4G2 | NM_001418 | Hs.183684 | chr11p15 |
| 200002_at | ribosomal protein L35 /// ribosomal protein L35 | RPL35 | NM_007209 | Hs.182825 | chr9q34.1 |
| 200001_at | calpain, small subunit 1 /// calpain, small subunit 1 | CAPNS1 | NM_001749 | Hs.388469 | chr19q13.12 |
| 200000_s_at | PRP8 pre-mRNA processing factor 8 homolog (S. cerevisiae) /// PRP8 pre-mRNA processing factor 8 homolog (S. cerevisiae) | PRPF8 | NM_006445 | Hs.181368 | chr17p13.3 |
| 160020_at | matrix metallopeptidase 14 (membrane-inserted) | MMP14 | Z48481 | Hs.2399 | chr14q11-q12 |
| 1570552_at | chromosome 18 open reading frame 50 | C18orf50 | AF363068 |  |  |
| 1570523_s_at | ATG10 autophagy related 10 homolog (S. cerevisiae) | ATG10 | AF318326 | Hs.386384 | chr5q14.1-q14.2 |
| 1570198_x_at | Bone marrow stromal cell antigen 2 | BST2 | BC019872 |  |  |
| 1570165_at | Carbohydrate (chondroitin 4) sulfotransferase 11 | CHST11 | BC027983 |  |  |
| 1569935_at | Zinc finger protein 664 | ZNF664 | BC033539 |  |  |
| 1569906_s_at | PHD finger protein 20 | PHF20 | BC015538 | Hs.301055 | chr20q11.22-q11.23 |
| 1569792_a_at | hypothetical protein MGC50559 | MGC50559 | BC039107 | Hs.409538 | chr12p11.21 |
| 1569629_x_at | similar to Serine/threonine-protein kinase PRKX (Protein kinase PKX1) | LOC389906 | BC017231 | Hs.497008 | chrXp22.33 |
| 1569607_s_at | ankyrin repeat domain 20 family, member A1 /// chromosome 21 open reading frame 81 /// ankyrin repeat domain 20 family, member A3 /// ankyrin repeat domain 20 family, member A2 /// similar to ankyrin repeat domain 20A /// similar to ankyrin repeat domain | ANKRD20A1 | BC016022 | Hs.511774 | chr21q11.2 /// chr9q12 |
| 1569520_at | Leukocyte-derived arginine aminopeptidase | LRAP | BC019347 | Hs.127981 | chr5q15 |
| 1569470_a_at | FERM domain containing 5 | FRMD5 | BC020191 | Hs.368399 | chr15q15.3 |
| 1569459_a_at | Zinc finger protein 451 | ZNF451 | BC035337 |  |  |
| 1569409_x_at | Inhibin, alpha | INHA | BC037812 | Hs.407506 | chr2q33-q36 |
| 1569349_at | chromosome 11 open reading frame 30 | C11orf30 | BG272041 | Hs.352588 | chr11q13.5 |
| 1569311_at | Hypothetical LOC554203 | LOC554203 | BC038589 |  |  |
| 1569302_at | KIAA1731 | KIAA1731 | BC017394 | Hs.458418 | chr11q21 |
| 1569253_at | integrator complex subunit 4 | INTS4 | BC018672 | Hs.141245 | chr11q13.5 |
| 1569183_a_at | choroideremia (Rab escort protein 1) | CHM | BC032237 |  | chrxq21.2 |
| 1569180_at | Leucine rich repeat containing 37, member A2 | LRRC37A2 | BC017896 |  |  |
| 1569157_s_at | hypothetical protein LOC162993 | LOC162993 | BC037359 | Hs.445295 | chr19p13.2 |
| 1569132_s_at | Arylsulfatase family, member K | ARSK | BC015723 | Hs.163914 | chr5q15 |
| 1569112_at | solute carrier family 44, member 5 | SLC44A5 | AW020413 | Hs.213897 | chr1p31.1 |
| 1569110_x_at | programmed cell death protein 6-like | LOC728613 | BC020552 | Hs.24087 | chr5pter-p15.2 |
| 1569107_s_at | zinc finger protein 642 | ZNF642 | BQ433060 | Hs.434385 | chr1p34.2 |
| 1569106_s_at | SET domain containing 5 | SETD5 | BI087313 | Hs.405917 | chr3p25.3 |
| 1569057_s_at | melanoma inhibitory activity family, member 3 | MIA3 | BC031805 | Hs.118474 | chr1p36.33 |
| 1569030_s_at | negative regulator of ubiquitin-like proteins 1 | NUB1 | BC034716 | Hs.182454 | chr7q36 |
| 1569013_s_at | Hypothetical protein similar to KIAA0187 gene product | LOC96610 | BC033148 | Hs.356397 | chr22q11.22 |
| 1568955_at | similar to SLIT-ROBO Rho GTPase-activating protein 2 (srGAP2) (Formin-binding protein 2) | LOC653464 | BE877775 |  |  |
| 1568877_a_at | acyl-Coenzyme A binding domain containing 5 | ACBD5 | BC025309 | Hs.14051 | chr10p12.1 |
| 1568830_at | RNA binding motif, single stranded interacting protein 1 | RBMS1 | BC029493 | Hs.268552 | chr12q14.3 |
| 1568781_at | Uroporphyrinogen III synthase (congenital erythropoietic porphyria) | UROS | BC024936 |  |  |
| 1568780_at | Hypothetical LOC497257 | LOC497257 | BC030211 | Hs.449392 | chr8p23.1 /// chr12p13.31 |
| 1568742_at | Hypothetical protein LOC284757 | LOC284757 | BC027461 |  |  |
| 1568695_s_at | integrator complex subunit 6 | INTS6 | AW665713 | Hs.396557 | chr13q14.12-q14.2 |
| 1568678_s_at | FGFR1 oncogene partner /// chromosome 9 open reading frame 4 | FGFR1OP | BC037785 | Hs.180296 | chr6q27 |
| 1568658_at | hypothetical gene supported by AK075484; BC014578 | LOC339804 | BU069195 | Hs.140617 | chr2p15 |
| 1568618_a_at | UDP-N-acetyl-alpha-D-galactosamine:polypeptide N-acetylgalactosaminyltransferase 1 (GalNAc-T1) | GALNT1 | BC038440 | Hs.277324 | chr18q12.1 |
| 1568611_at | Procollagen-proline, 2-oxoglutarate 4-dioxygenase (proline 4-hydroxylase), alpha polypeptide II | P4HA2 | CA418310 |  |  |
| 1568594_s_at | tripartite motif-containing 52 | TRIM52 | AW965171 | Hs.458412 | chr5q35.3 |
| 1568592_at | tripartite motif-containing 69 | TRIM69 | BM976092 | Hs.400456 | chr15q21.1 |
| 1567575_at | Brain-derived neurotrophic factor opposite strand | BDNFOS | AJ011601 |  |  |
| 1567458_s_at | ras-related C3 botulinum toxin substrate 1 (rho family, small GTP binding protein Rac1) | RAC1 | AJ012502 | Hs.413812 | chr7p22 |
| 1567410_at | zinc finger protein 135 | ZNF135 | X65231 |  |  |
| 1567214_a_at | pinin, desmosome associated protein | PNN | U59479 | Hs.409965 | chr14q21.1 |
| 1567032_s_at | zinc finger protein 160 | ZNF160 | X78928 | Hs.467236 | chr19q13.41 |
| 1567013_at | nuclear factor (erythroid-derived 2)-like 2 | NFE2L2 | AF323119 | Hs.446484 | chr20p13 |
| 1566603_s_at | RNA pseudouridylate synthase domain containing 3 | RPUSD3 | AK094587 | Hs.33719 | chr3p25.3 |
| 1566577_at | Dihydropyrimidinase-like 5 | DPYSL5 | AL831879 | Hs.299315 | chr2p23.3 |
| 1566557_at | hypothetical protein LOC440465 | FLJ90757 | AK096609 |  |  |
| 1566472_s_at | retinol saturase (all-trans-retinol 13,14-reductase) | RETSAT | AK098125 | Hs.440401 | chr2p11.2 |
| 1566303_s_at | protein phosphatase 1, regulatory (inhibitor) subunit 11 | PPP1R11 | AV755778 | Hs.121178 | chr6p21.3 |
| 1566084_at | Hypothetical LOC644215 | LOC644215 | AK090649 | Hs.469370 |  |
| 1565867_a_at | zinc finger CCCH-type containing 11A | ZC3H11A | W01876 |  |  |
| 1565703_at | SMAD family member 4 | SMAD4 | AL832789 | Hs.75862 | chr18q21.1 |
| 1565651_at | ADP-ribosylation factor 1 | ARF1 | BI868311 | Hs.282450 |  |
| 1565269_s_at | activating transcription factor 1 | ATF1 | AF047022 | Hs.246842 | chr12q13 |
| 1565162_s_at | microsomal glutathione S-transferase 1 | MGST1 | D16947 | Hs.389700 | chr12p12.3-p12.1 |
| 1564637_a_at | family with sequence similarity 98, member B | FAM98B | BC035600 | Hs.6799 | chr15q14 |
| 1564494_s_at | procollagen-proline, 2-oxoglutarate 4-dioxygenase (proline 4-hydroxylase), beta polypeptide | P4HB | AK075503 | Hs.410578 | chr17q25 |
| 1564474_at | Hypothetical protein LOC728723 | LOC728723 | AK055144 | Hs.382199 | chr5q13.3 |
| 1564467_at | Hypothetical protein FLJ13305 | FLJ13305 | AK091575 | Hs.440466 | chr2p15 |
| 1564424_at | Family with sequence similarity 62 (C2 domain containing) member B | FAM62B | BC035983 | Hs.388073 | chr7q36.3 |
| 1564381_s_at | CCAAT/enhancer binding protein zeta | CEBPZ | AK001814 | Hs.381077 |  |
| 1564362_x_at | hypothetical protein MGC46336 | MGC46336 | AK096248 | Hs.381893 | chr16p11.2 |
| 1564207_at | hypothetical protein FLJ35390 | FLJ35390 | BC014556 |  |  |
| 1564129_a_at | Quinolinate phosphoribosyltransferase (nicotinate-nucleotide pyrophosphorylase (carboxylating)) | QPRT | AK090801 |  |  |
| 1564053_a_at | YTH domain family, member 3 | YTHDF3 | AK093081 | Hs.5518 | chr8q12.3 |
| 1563745_a_at | hypothetical protein LOC283050 | LOC283050 | AK098249 | Hs.309176 | chr10q22.3 |
| 1563469_at | AT rich interactive domain 5B (MRF1-like) | ARID5B | AL832681 |  |  |
| 1563467_at | Platelet derived growth factor C | PDGFC | AL833266 | Hs.43080 | chr4q32 |
| 1563431_x_at | Calmodulin 3 (phosphorylase kinase, delta) | CALM3 | AA807959 |  |  |
| 1563111_a_at | phosphatidylinositol glycan anchor biosynthesis, class X | PIGX | AK000529 | Hs.223296 | chr3q29 |
| 1562849_at | Sperm antigen with calponin homology and coiled-coil domains 1 | SPECC1 | BQ002451 | Hs.364299 |  |
| 1562612_at | Malic enzyme 2, NAD(+)-dependent, mitochondrial | ME2 | BC020933 | Hs.75342 | chr6p25-p24 |
| 1562529_s_at | RAR-related orphan receptor A | RORA | BC040965 | Hs.60770 | chr15q21-q22 |
| 1562415_a_at | SPOC domain containing 1 | SPOCD1 | AK096192 | Hs.62604 | chr1p35.2 |
| 1562386_s_at | zinc finger protein 501 | ZNF501 | H16098 | Hs.438260 | chr3p21.31 |
| 1562228_s_at | phosphodiesterase 5A, cGMP-specific | PDE5A | AF043732 | Hs.408150 | chr4q25-q27 |
| 1562063_x_at | neuroblastoma breakpoint family, member 1 /// neuroblastoma breakpoint family, member 3 /// KIAA1245 /// neuroblastoma breakpoint family, member 11 /// neuroblastoma breakpoint family, member 20 /// neuroblastoma breakpoint family, member 9 /// neuroblast | NBPF1 | BM041211 |  |  |
| 1561687_a_at | zinc finger protein 382 | ZNF382 | BC038358 | Hs.138297 | chr19q13.12 |
| 1561365_at | Neuropilin 1 | NRP1 | AA609131 | Hs.173548 | chr10p12 |
| 1561286_a_at | DIP2 disco-interacting protein 2 homolog A (Drosophila) | DIP2A | BQ017399 | Hs.322903 | chr21q22.3 |
| 1561281_a_at | hypothetical protein LOC284576 | LOC284576 | AK090497 | Hs.202262 | chr1q32.1 |
| 1561135_at | FLJ34870 protein | FLJ34870 | AF085980 | Hs.270083 | chr2q21.2 |
| 1561093_at | putative UST1-like organic anion transporter | LOC387601 | AK091990 | Hs.332119 | chr11q12.3 |
| 1560916_a_at | dpy-19-like 1 (C. elegans) | DPY19L1 | BI461155 | Hs.408623 | chr7p14.3-p14.2 |
| 1560622_at | Transmembrane protein 165 | TMEM165 | AK000203 | Hs.429961 | chr4q12 |
| 1560587_s_at | peroxiredoxin 5 | PRDX5 | AI718223 | Hs.31731 | chr11q13 |
| 1560562_a_at | Zinc finger protein 677 | ZNF677 | AK026366 | Hs.20506 | chr19q13.42 |
| 1560469_at | nuclear receptor subfamily 5, group A, member 2 | NR5A2 | BC027893 | Hs.183123 | chr1q32.1 |
| 1560434_x_at | Clathrin, light chain (Lca) /// CDNA FLJ31398 fis, clone NT2NE1000175 | CLTA | AK055960 | Hs.283453 | chr9p13.2 |
| 1560339_s_at | nucleosome assembly protein 1-like 4 | NAP1L4 | AK095320 | Hs.269403 | chr11p15.5 |
| 1560271_at | Polypyrimidine tract binding protein 2 | PTBP2 | BC030757 | Hs.34956 | chr1p22.1-p21.3 |
| 1560265_at | glutamate receptor, ionotropic, kainate 2 | GRIK2 | BQ434382 | Hs.98262 | chr6q16.3-q21 |
| 1560250_s_at | hypothetical protein LOC284242 | LOC284242 | BC035844 |  | chr18q12.1 |
| 1560001_at | Hypothetical LOC389634 | LOC389634 | AK092544 | Hs.449392 | chr8p23.1 |
| 1559970_at | Hypothetical protein LOC730034 | LOC730034 | BG680343 | Hs.402352 | chr14q31.3 |
| 1559957_a_at | hypothetical LOC642852 | LOC642852 | BG396868 | Hs.271450 |  |
| 1559881_s_at | zinc finger protein 12 | ZNF12 | BM463827 | Hs.102397 | chr7p22-p21 |
| 1559826_a_at | hypothetical LOC401074 | LOC401074 | BC039495 | Hs.528540 | chr3p12.3 |
| 1559716_at | chromosome 18 open reading frame 37 | C18orf37 | AK095502 | Hs.407011 | chr18q12.2 |
| 1559517_a_at | spire homolog 1 (Drosophila) | SPIRE1 | AL833817 | Hs.16758 | chr18p11.21 |
| 1559467_at | 5,10-methenyltetrahydrofolate synthetase (5-formyltetrahydrofolate cyclo-ligase) | MTHFS | BI520422 | Hs.407031 |  |
| 1559400_s_at | pregnancy-associated plasma protein A, pappalysin 1 | PAPPA | BG620958 | Hs.440769 | chr9q33.2 |
| 1559391_s_at | UDP-Gal:betaGlcNAc beta 1,4- galactosyltransferase, polypeptide 5 | B4GALT5 | AI084451 | Hs.107526 | chr20q13.1-q13.2 |
| 1559227_s_at | von Hippel-Lindau tumor suppressor | VHL | BF972755 | Hs.421597 | chr3p26-p25 |
| 1559190_s_at | MRNA; cDNA DKFZp313H0740 (from clone DKFZp313H0740) /// Retinol dehydrogenase 13 (all-trans/9-cis) | RDH13 | AL833150 | Hs.327631 | chr19q13.42 |
| 1559140_at | family with sequence similarity 87, member A | FAM87A | BC037297 | Hs.406174 | chr1p36.33 |
| 1559101_at | FYN oncogene related to SRC, FGR, YES | FYN | AK090692 | Hs.390567 | chr6q21 |
| 1559060_a_at | Folliculin interacting protein 1 | FNIP1 | BF677986 |  | chr5q31.1 |
| 1559052_s_at | p21 (CDKN1A)-activated kinase 2 | PAK2 | U25975 | Hs.284275 | chr3q29 |
| 1559048_at | Hypothetical protein LOC728498 /// BAH domain and coiled-coil containing 1 /// Similar to golgi autoantigen, golgin subfamily a, 8E /// Transcribed locus, strongly similar to XP_940396.1 similar to golgi autoantigen, golgin family member isoform 3 [Homo | LOC728498 | AA331299 | Hs.154483 |  |
| 1559005_s_at | chromosome 9 open reading frame 39 | C9orf39 | AK098502 | Hs.270502 | chr9p22.2 |
| 1559002_at | hypothetical protein LOC340544 | LOC340544 | BC043382 | Hs.441076 | chrxq22.2 |
| 1558969_a_at | ribosomal protein L32 pseudogene 3 | RPL32P3 | BG741817 | Hs.437334 | chr3q21.3 |
| 1558956_s_at | intraflagellar transport 80 homolog (Chlamydomonas) | IFT80 | AI473255 | Hs.186547 | chr3q25.33 |
| 1558943_x_at | zinc finger protein 765 | ZNF765 | BU686397 |  |  |
| 1558924_s_at | restin (Reed-Steinberg cell-expressed intermediate filament-associated protein) | RSN | BF673049 | Hs.410113 | chr12q24.3 |
| 1558896_at | chromosome 1 open reading frame 69 | C1orf69 | AA442490 | Hs.145749 |  |
| 1558871_at | Epithelial mitogen homolog (mouse) | EPGN | BC016361 | Hs.337220 |  |
| 1558801_at | Similar to S-adenosylmethionine decarboxylase proenzyme 2 (AdoMetDC 2) (SamDC 2) | LOC730846 | AK055769 |  |  |
| 1558794_at | Hypothetical protein LOC728190 | LOC728190 | AK091716 |  |  |
| 1558783_at | Wilms tumor 1 associated protein | WTAP | AK024805 | Hs.446091 | chr6q25-q27 |
| 1558754_at | zinc finger protein 763 | ZNF763 | AA484731 | Hs.465952 | chr19p13.2 |
| 1558692_at | Chromosome 1 open reading frame 85 | C1orf85 | AW090182 | Hs.202522 | chr1q22 |
| 1558685_a_at | hypothetical protein BC009467 | LOC158960 | BC009467 | Hs.111407 | chrxq28 |
| 1558643_s_at | EGF-like repeats and discoidin I-like domains 3 | EDIL3 | AA297258 | Hs.441044 | chr5q14 |
| 1558641_at | hypothetical protein LOC202051 | LOC202051 | AK098740 | Hs.373612 | chr5q31.2 |
| 1558569_at | Mastermind-like 2 (Drosophila) | MAML2 | AL832308 | Hs.503649 | chr11q21 |
| 1558518_at | mitogen-activated protein kinase kinase kinase 7 interacting protein 3 | MAP3K7IP3 | AL832071 | Hs.371135 | chrxp21.3 |
| 1558508_a_at | chromosome 1 open reading frame 53 | C1orf53 | BI832220 | Hs.61329 | chr1q31.3 |
| 1558501_at | dynamin 3 | DNM3 | AI631915 | Hs.135159 | chr1q24.3 |
| 1558487_a_at | transmembrane emp24 protein transport domain containing 4 | TMED4 | AK074557 | Hs.405972 | chr7p13 |
| 1558430_at | KIAA0251 protein | KIAA0251 | R08650 | Hs.439211 | chr16p13.11 |
| 1558404_at | Hypothetical protein LOC644242 | LOC644242 | BC015390 | Hs.380331 |  |
| 1558369_at | M-phase phosphoprotein 9 | MPHOSPH9 | BC036600 | Hs.445084 | chr12q24.31 |
| 1558345_a_at | hypothetical gene supported by NM_194304 | LOC439911 | BI715235 |  |  |
| 1558322_a_at | progestin and adipoQ receptor family member IX | PAQR9 | BG702061 | Hs.408385 | chr3q23 |
| 1558305_at | Trinucleotide repeat containing 15 | TNRC15 | BC029456 | Hs.334871 | chr2q37.1 |
| 1558292_s_at | phosphatidylinositol glycan anchor biosynthesis, class W | PIGW | BF037819 | Hs.378885 | chr17q12 |
| 1558290_a_at | Pvt1 oncogene homolog, MYC activator (mouse) | PVT1 | BG200951 | Hs.100425 | chr8q24 |
| 1558253_x_at | zinc finger protein 587 | ZNF587 | AK055448 | Hs.288995 | chr19q13.43 |
| 1558214_s_at | catenin (cadherin-associated protein), alpha 1, 102kDa | CTNNA1 | BG330076 | Hs.254321 | chr5q31 |
| 1558201_s_at | solute carrier family 4 (anion exchanger), member 1, adaptor protein | SLC4A1AP | AI889922 | Hs.306000 | chr2p23.3-p23.2 |
| 1558199_at | fibronectin 1 | FN1 | W73431 | Hs.418138 | chr2q34 |
| 1558139_at | hypothetical protein LOC642477 | FLJ39632 | BE730158 | Hs.252930 | chr14q11.1 |
| 1558136_s_at | TAF11 RNA polymerase II, TATA box binding protein (TBP)-associated factor, 28kDa | TAF11 | BQ709323 | Hs.83126 | chr6p21.31 |
| 1558117_s_at | ubiquitin specific peptidase 31 | USP31 | BG778320 | Hs.16953 | chr16p12.1 |
| 1558116_x_at | reversion-inducing-cysteine-rich protein with kazal motifs | RECK | BC032240 |  |  |
| 1558102_at | Transmembrane 6 superfamily member 1 | TM6SF1 | AK055438 | Hs.151155 | chr15q24-q26 |
| 1558097_at | chromosome 22 open reading frame 30 | C22orf30 | W21283 | Hs.438906 | chr22q12.2 |
| 1558080_s_at | hypothetical protein LOC144871 | LOC144871 | BG913589 |  | chr13q32.1 |
| 1558044_s_at | Exosome component 6 | EXOSC6 | BF692729 |  |  |
| 1558028_x_at | hypothetical protein LOC647979 | LOC647979 | BI857154 |  |  |
| 1558014_s_at | male sterility domain containing 2 | MLSTD2 | BG261090 | Hs.288368 | chr11p15.2 |
| 1558002_at | Serine/threonine kinase receptor associated protein | STRAP | BQ944989 | Hs.3727 | chr12p12.3 |
| 1557987_at | PI-3-kinase-related kinase SMG-1 - like locus | LOC641298 | BC042832 | Hs.375005 | chr16p12.2 |
| 1557954_at | chromosome X open reading frame 15 | CXorf15 | BM757251 | Hs.406078 | chrXp22.2 |
| 1557953_at | zinc finger with KRAB and SCAN domains 1 | ZKSCAN1 | BG761185 | Hs.24758 | chr7q21.3-q22.1 |
| 1557950_at | serologically defined colon cancer antigen 1 | SDCCAG1 | BF984340 | Hs.388584 | chr14q22 |
| 1557945_at | T-complex-associated-testis-expressed 3 | TCTE3 | BC043594 | Hs.25094 | chr6q27 |
| 1557944_s_at | catenin (cadherin-associated protein), delta 1 | CTNND1 | BG248249 | Hs.166011 | chr11q11 |
| 1557915_s_at | glutathione S-transferase omega 1 | GSTO1 | U56250 | Hs.11465 | chr10q25.1 |
| 1557910_at | heat shock protein 90kDa alpha (cytosolic), class B member 1 | HSP90AB1 | BG612458 | Hs.74335 | chr6p12 |
| 1557905_s_at | CD44 molecule (Indian blood group) /// mitogen-activated protein kinase 10 | CD44 | AL552534 | Hs.306278 | chr11p13 |
| 1557828_a_at | hypothetical protein LOC646916 | LOC646916 | BE675061 | Hs.294144 | chr5p12 |
| 1557820_at | AFG3 ATPase family gene 3-like 2 (yeast) | AFG3L2 | AA905788 | Hs.436683 | chr18p11 |
| 1557813_at | Single-stranded DNA binding protein 2 | SSBP2 | BF724621 | Hs.152207 | chr5q14.1 |
| 1557799_at | chromosome 11 open reading frame 31 | C11orf31 | AF085883 | Hs.412817 | chr11q12.1 |
| 1557754_at | hypothetical gene supported by BC028186 | LOC401068 | BC028186 | Hs.385650 | chr3p21.1 |
| 1557751_at | Zinc finger protein 644 | ZNF644 | AI537913 | Hs.173001 | chr1p22.2 |
| 1557729_at | G protein-coupled receptor kinase 5 | GRK5 | AK097099 | Hs.375498 |  |
| 1557666_s_at | Chromosome 9 open reading frame 98 | C9orf98 | AW511637 | Hs.421340 | chr9q34.13 |
| 1557586_s_at | ATPase, H+ transporting, lysosomal 50/57kDa, V1 subunit H | ATP6V1H | AW468382 | Hs.19575 | chr8p22-q22.3 |
| 1557553_at | Protein phosphatase 1, regulatory (inhibitor) subunit 12B | PPP1R12B | BF438357 | Hs.269777 | chr1q32.1 |
| 1557539_at | KIAA1545 protein | KIAA1545 | BC008052 | Hs.127270 | chr12q24.33 |
| 1557527_at | Runt-related transcription factor 1 (acute myeloid leukemia 1; aml1 oncogene) | RUNX1 | BU789637 | Hs.410774 | chr21q22.3 |
| 1557455_s_at | motile sperm domain containing 1 | MOSPD1 | AF086333 | Hs.57549 | chrxq26.3 |
| 1557433_at | G-protein signalling modulator 2 (AGS3-like, C. elegans) | GPSM2 | BU687162 |  |  |
| 1557432_at | RAS protein activator like 2 | RASAL2 | BQ003426 | Hs.128869 | chr1q24 |
| 1557415_s_at | leucine zipper-EF-hand containing transmembrane protein 2 | LETM2 | AI023774 | Hs.521540 | chr8p12 |
| 1557411_s_at | solute carrier family 25, member 43 | SLC25A43 | AK094254 | Hs.294799 | chrxq24 |
| 1557370_s_at | MYC binding protein 2 | MYCBP2 | AK000968 | Hs.151411 | chr13q22 |
| 1557331_at | polymerase (RNA) I polypeptide B, 128kDa | POLR1B | CA442932 |  |  |
| 1557285_at | Similar to Amphiregulin precursor (AR) (Colorectum cell-derived growth factor) (CRDGF) | LOC727738 | AI891075 | Hs.270833 | chr4q13-q21 |
| 1557270_at | Zinc finger protein 69 | ZNF69 | AA632049 | Hs.396544 |  |
| 1557258_a_at | B-cell CLL/lymphoma 10 | BCL10 | AA994334 | Hs.193516 | chr1p22 |
| 1557227_s_at | translocated promoter region (to activated MET oncogene) | TPR | AW235355 | Hs.170472 | chr1q25 |
| 1557174_a_at | Interleukin-1 receptor-associated kinase 1 binding protein 1 | IRAK1BP1 | AK096861 | Hs.529940 | chr6q14-q15 |
| 1557169_x_at | HLA complex group 11 | HCG11 | AK024111 | Hs.272939 |  |
| 1557164_a_at | RAD26L hypothetical protein | LOC375748 | BM683657 | Hs.512650 | chr9q22.32 |
| 1557158_s_at | myeloid/lymphoid or mixed-lineage leukemia 3 | MLL3 | AI479224 | Hs.298119 | chr7q34-q36 |
| 1557137_at | transmembrane protein 17 | TMEM17 | AA084725 | Hs.308028 | chr2p15 |
| 1557081_at | RNA binding motif protein 25 | RBM25 | AA580691 | Hs.197184 | chr14q24.3 |
| 1557064_s_at | heparan-alpha-glucosaminide N-acetyltransferase | HGSNAT | AW328331 | Hs.191320 | chr8p11.1 |
| 1557049_at | Hypothetical protein LOC149478 | LOC149478 | BQ183759 | Hs.421430 | chr1p34.1 |
| 1556827_at | hypothetical protein LOC339929 | LOC339929 | BC042414 | Hs.365471 | chr3q28 |
| 1556820_a_at | deleted in lymphocytic leukemia, 2 | DLEU2 | H48516 | Hs.446406 | chr13q14.3 |
| 1556787_s_at | hypothetical protein LOC730456 | LOC730456 | AW975338 | Hs.408150 | chr4q25-q27 |
| 1556730_at | hypothetical LOC652993 | LOC652993 | AK074510 | Hs.534436 | chr12q15 |
| 1556696_s_at | Hypothetical gene supported by AK124699 | FLJ42709 | AK095719 | Hs.446400 | chr5q15 |
| 1556690_s_at | Myeloid zinc finger 1 | MZF1 | AW341225 | Hs.458361 | chr19q13.2-q13.4 |
| 1556672_a_at | RNA binding motif protein 6 | RBM6 | AI190489 | Hs.188879 | chr3p21.3 |
| 1556613_s_at | hypothetical protein LOC203107 | LOC203107 | BC038568 |  | chr8q22.1 |
| 1556588_at | chromosome 15 open reading frame 37 | C15orf37 | AK091107 | Hs.512015 | chr15q25.1 |
| 1556568_a_at | Nemo-like kinase | NLK | N46436 |  |  |
| 1556442_x_at | RAB guanine nucleotide exchange factor (GEF) 1 | RABGEF1 | AU159357 |  |  |
| 1556425_a_at | hypothetical protein LOC284219 | LOC284219 | BF509747 |  | chr18p11.22 |
| 1556385_at | Cardiotrophin-like cytokine factor 1 | CLCF1 | BQ028191 | Hs.191548 | chr11q13.3 |
| 1556316_s_at | hypothetical protein LOC284889 | LOC284889 | AI285192 | Hs.535827 | chr22q11.23 |
| 1556285_s_at | pyrophosphatase (inorganic) 2 | PPA2 | AF086012 | Hs.421825 | chr4q25 |
| 1556244_s_at | hypothetical protein LOC375196 | LOC375196 | BC030087 |  | chr2p22.3 |
| 1556239_a_at | HERPUD family member 2 | HERPUD2 | BC040322 | Hs.424711 | chr7p14.2 |
| 1556180_at | hypothetical protein LOC729678 | LOC729678 | BE646146 | Hs.348292 | chr5q35.3 |
| 1556176_at | taube nuss homolog (mouse) | TBN | BU618741 | Hs.254211 | chr6p21.1 |
| 1556067_a_at | jumonji domain containing 3 | JMJD3 | AI830331 | Hs.103915 | chr17p13.1 |
| 1556064_at | Hypothetical protein LOC284926 | LOC284926 | BQ575161 | Hs.449096 | chr22q13.31 |
| 1556061_at | hypothetical protein LOC283012 | LOC283012 | AI760272 |  | chr10q23.32 |
| 1556060_a_at | KIAA1702 protein | KIAA1702 | AK027074 |  | chr6p11 |
| 1556059_s_at | spen homolog, transcriptional regulator (Drosophila) | SPEN | BM992098 | Hs.184245 | chr1p36.33-p36.11 |
| 1556035_s_at | zinc finger protein 207 | ZNF207 | AI201248 | Hs.97128 | chr17q11.2 |
| 1556015_a_at | mesoderm posterior 2 homolog (mouse) | MESP2 | AL360139 | Hs.37311 | chr15q26.1 |
| 1556006_s_at | Casein kinase 1, alpha 1 | CSNK1A1 | BQ025347 | Hs.318381 | chr5q32 |
| 1555997_s_at | insulin-like growth factor binding protein 5 | IGFBP5 | BM128432 | Hs.369982 | chr2q33-q36 |
| 1555976_s_at | Hypothetical protein LOC727918 | LOC727918 | BU676221 | Hs.535080 | chr18p11.31 |
| 1555963_x_at | UDP-GlcNAc:betaGal beta-1,3-N-acetylglucosaminyltransferase 7 | B3GNT7 | CA503291 | Hs.299329 | chr2q37.1 |
| 1555961_a_at | histidine triad nucleotide binding protein 1 | HINT1 | AK054976 | Hs.256697 | chr5q31.2 |
| 1555950_a_at | CD55 molecule, decay accelerating factor for complement (Cromer blood group) | CD55 | CA448665 | Hs.408864 | chr1q32 |
| 1555938_x_at | vimentin | VIM | AI520969 | Hs.435800 | chr10p13 |
| 1555923_a_at | chromosome 10 open reading frame 114 | C10orf114 | BQ007522 | Hs.315568 | chr10p12.31 |
| 1555920_at | Chromobox homolog 3 (HP1 gamma homolog, Drosophila) | CBX3 | BU683892 | Hs.381189 | chr7p15.2 |
| 1555912_at | ST7 overlapping transcript 1 (antisense non-coding RNA) | ST7OT1 | AI016213 |  | chr7q31.1-7q31.2 |
| 1555910_at | pentatricopeptide repeat domain 2 | PTCD2 | AK056761 | Hs.126906 | chr5q13.2 |
| 1555908_at | family with sequence similarity 120A | FAM120A | BF591199 | Hs.446534 | chr9q22.31 |
| 1555905_a_at | chromosome 3 open reading frame 23 | C3orf23 | AI147556 | Hs.55131 | chr3p21.33-p21.32 |
| 1555892_s_at | hypothetical protein LOC253039 | LOC253039 | BF476087 |  | chr9q33.3 |
| 1555883_s_at | spindlin family, member 3 | SPIN3 | AJ271379 | Hs.447893 | chrxp11.1 |
| 1555881_s_at | leucine zipper, putative tumor suppressor 2 | LZTS2 | AK097997 | Hs.25298 | chr10q24 |
| 1555878_at | Ribosomal protein S24 | RPS24 | AK094613 | Hs.356794 | chr10q22-q23 |
| 1555874_x_at | hypothetical protein MGC21881 | RP11-262H14.4 | BC019880 | Hs.494004 | chr9q21.11 |
| 1555869_a_at | Hypothetical protein LOC729070 | LOC729070 | BG205347 | Hs.179213 |  |
| 1555867_at | hypothetical protein LOC200169 | LOC200169 | BM666010 |  | chr1q42.3 |
| 1555866_a_at | hexosaminidase (glycosyl hydrolase family 20, catalytic domain) containing | HEXDC | BM980844 | Hs.444743 | chr17q25.3 |
| 1555864_s_at | pyruvate dehydrogenase (lipoamide) alpha 1 | PDHA1 | AW057819 | Hs.1023 | chrxp22.2-p22.1 |
| 1555860_x_at | Hypothetical gene supported by AK128398 | LOC440944 | CA430188 | Hs.440069 | chr3p25.3 |
| 1555851_s_at | selenoprotein W, 1 | SEPW1 | AW514401 | Hs.433941 | chr19q13.3 |
| 1555847_a_at | hypothetical protein LOC284454 | LOC284454 | BU617052 | Hs.436426 | chr19p13.13 |
| 1555844_s_at | heterogeneous nuclear ribonucleoprotein M | HNRPM | AL713781 | Hs.385766 | chr19p13.3-p13.2 |
| 1555837_s_at | polymerase (RNA) II (DNA directed) polypeptide B, 140kDa | POLR2B | BE614461 | Hs.149353 | chr4q12 |
| 1555832_s_at | Kruppel-like factor 6 | KLF6 | BU683415 | Hs.4055 |  |
| 1555831_s_at | leucine rich repeat containing 41 | LRRC41 | BC004948 | Hs.144941 | chr1p34.1 |
| 1555821_a_at | AKT1 substrate 1 (proline-rich) | AKT1S1 | BC016043 | Hs.242889 | chr19q13.33 |
| 1555820_a_at | Meckel syndrome, type 1 | MKS1 | BC010061 | Hs.408843 | chr17q23.2 |
| 1555814_a_at | ras homolog gene family, member A | RHOA | AF498970 | Hs.77273 | chr3p21.3 |
| 1555803_a_at | chromosome 11 open reading frame 57 | C11orf57 | BC030546 | Hs.195060 | chr11q23.1 |
| 1555801_s_at | zinc finger protein 533 | ZNF533 | BC038422 | Hs.470791 | chr2q31.2-q31.3 |
| 1555797_a_at | actin related protein 2/3 complex, subunit 5, 16kDa | ARPC5 | AF017807 | Hs.126222 | chr1q25.3 |
| 1555793_a_at | zinc finger protein 545 | ZNF545 | AL834267 | Hs.386338 | chr19q13.12 |
| 1555789_s_at | PHD finger protein 23 | PHF23 | AY099328 | Hs.534566 | chr17p13.1 |
| 1555785_a_at | 5'-3' exoribonuclease 1 | XRN1 | AY137776 | Hs.131168 | chr3q23 |
| 1555762_s_at | RNA binding motif protein 15 | RBM15 | AF364036 | Hs.435947 | chr1p13 |
| 1555751_a_at | gem (nuclear organelle) associated protein 7 | GEMIN7 | AY114106 | Hs.102991 | chr19q13.32 |
| 1555745_a_at | lysozyme (renal amyloidosis) | LYZ | U25677 | Hs.234734 | chr12q15 |
| 1555741_at | melanocortin 2 receptor accessory protein | MRAP | AF483549 | Hs.443257 | chr21q22.1 |
| 1555730_a_at | cofilin 1 (non-muscle) | CFL1 | D00682 | Hs.170622 | chr11q13 |
| 1555725_a_at | uroplakin 1B /// regulator of G-protein signalling 5 | UPK1B | AF493929 | Hs.24950 | chr1q23.1 |
| 1555705_a_at | CKLF-like MARVEL transmembrane domain containing 3 | CMTM3 | AY166714 | Hs.298198 | chr16q22.1 |
| 1555630_a_at | RAB34, member RAS oncogene family | RAB34 | AF327350 | Hs.301853 | chr17q11.2 |
| 1555618_s_at | SUMO1 activating enzyme subunit 1 | SAE1 | AF110956 | Hs.32748 | chr19q13.32 |
| 1555594_a_at | muscleblind-like (Drosophila) | MBNL1 | AF401998 | Hs.478000 | chr3q25 |
| 1555575_a_at | KDEL (Lys-Asp-Glu-Leu) endoplasmic reticulum protein retention receptor 1 | KDELR1 | BC008958 | Hs.78040 | chr19q13.3 |
| 1555564_a_at | complement factor I | CFI | BC020718 | Hs.312485 | chr4q25 |
| 1555561_a_at | UDP-glucose ceramide glucosyltransferase-like 2 | UGCGL2 | BC032302 | Hs.308242 | chr13q32.1 |
| 1555495_a_at | serologically defined colon cancer antigen 10 | SDCCAG10 | BC012117 | Hs.23557 | chr5q12.3 |
| 1555491_a_at | hypothetical protein FLJ11286 | FLJ11286 | BC035817 | Hs.175120 | chr19p13.2 |
| 1555467_a_at | CUG triplet repeat, RNA binding protein 1 | CUGBP1 | AF267533 | Hs.534422 | chr11p11 |
| 1555448_at | chromosome 14 open reading frame 108 | C14orf108 | BC013174 | Hs.106210 | chr14q22.3 |
| 1555446_s_at | transmembrane protein 1 | TMEM1 | BC046241 | Hs.438927 | chr21q22.3 |
| 1555419_a_at | N-acylsphingosine amidohydrolase (acid ceramidase) 1 | ASAH1 | BC016828 | Hs.324808 | chr8p22-p21.3 |
| 1555392_at | Testis derived transcript (3 LIM domains) | TES | AY143171 | Hs.129129 | chr7q31.2 |
| 1555384_a_at | La ribonucleoprotein domain family, member 4 | LARP4 | BC022377 | Hs.26613 | chr12q13.12 |
| 1555355_a_at | v-ets erythroblastosis virus E26 oncogene homolog 1 (avian) | ETS1 | BC017314 | Hs.189679 | chr11q23.3 |
| 1555326_a_at | ADAM metallopeptidase domain 9 (meltrin gamma) | ADAM9 | AF495383 | Hs.2442 | chr8p11.23 |
| 1555325_s_at | zinc finger protein 26 | ZNF26 | BC046206 | Hs.489608 | chr12q24.33 |
| 1555306_a_at | endothelin converting enzyme 2 | ECE2 | AF521189 | Hs.129801 | chr3q28-q29 |
| 1555294_a_at | ELKS/RAB6-interacting/CAST family member 1 | ERC1 | AB053469 | Hs.128282 | chr12p13.3 |
| 1555292_at | family with sequence similarity 40, member B | FAM40B | BC019064 | Hs.414787 | chr7q32.3 |
| 1555281_x_at | armadillo repeat containing 8 | ARMC8 | BC007934 | Hs.102708 | chr3q22.3 |
| 1555278_a_at | cytoskeleton associated protein 5 | CKAP5 | BC035554 | Hs.201253 | chr11p11.2 |
| 1555274_a_at | selenoprotein I | SELI | BC021229 | Hs.127243 | chr2p23.3 |
| 1555259_at | sterile alpha motif and leucine zipper containing kinase AZK | ZAK | AF465843 | Hs.115175 | chr2q24.2 |
| 1555248_a_at | WNK lysine deficient protein kinase 3 | WNK3 | AY082340 | Hs.92423 | chrxp11.23-p11.21 |
| 1555247_a_at | Rap guanine nucleotide exchange factor (GEF) 6 | RAPGEF6 | AF394782 | Hs.434867 | chr5q23.3 |
| 1555241_at | chromosome 8 open reading frame 59 | C8orf59 | BC032347 | Hs.443072 | chr8q21.2 |
| 1555240_s_at | guanine nucleotide binding protein (G protein), gamma 12 | GNG12 | AF493879 | Hs.8107 | chr1p31.2 |
| 1555229_a_at | complement component 1, s subcomponent | C1S | BC007010 | Hs.458355 | chr12p13 |
| 1555225_at | chromosome 1 open reading frame 43 | C1orf43 | BC008306 | Hs.355906 | chr1q21.2 |
| 1555201_a_at | required for meiotic nuclear division 1 homolog (S. cerevisiae) | RMND1 | BC012081 | Hs.238270 | chr6q25.1 |
| 1555193_a_at | zinc finger protein 277 pseudogene | ZNF277P | BC020626 | Hs.489722 | chr7q31.1 |
| 1555167_s_at | pre-B-cell colony enhancing factor 1 | PBEF1 | BC020691 | Hs.293464 | chr7q22.3 |
| 1555123_at | ST6 beta-galactosamide alpha-2,6-sialyltranferase 2 | ST6GAL2 | BC008680 | Hs.98265 | chr2q11.2-q12.1 |
| 1555112_a_at | chromosome 1 open reading frame 114 | C1orf114 | BC026073 | Hs.130746 | chr1q24 |
| 1555097_a_at | prostaglandin F receptor (FP) | PTGFR | BC035694 | Hs.292843 | chr1p31.1 |
| 1555058_a_at | lysophosphatidylglycerol acyltransferase 1 | LPGAT1 | BC034621 | Hs.407774 | chr1p36.13-q42.3 |
| 1555041_a_at | N-acetylgalactosaminidase, alpha- | NAGA | M29276 | Hs.75372 | chr22q11 |
| 1555037_a_at | isocitrate dehydrogenase 1 (NADP+), soluble | IDH1 | BC012846 | Hs.11223 | chr2q33.3 |
| 1555021_a_at | scavenger receptor class F, member 1 | SCARF1 | AB052950 | Hs.534401 | chr17p13.3 |
| 1554997_a_at | prostaglandin-endoperoxide synthase 2 (prostaglandin G/H synthase and cyclooxygenase) | PTGS2 | AY151286 | Hs.196384 | chr1q25.2-q25.3 |
| 1554980_a_at | activating transcription factor 3 | ATF3 | AB066566 | Hs.460 | chr1q32.3 |
| 1554966_a_at | filamin A interacting protein 1-like | FILIP1L | AF329092 | Hs.15432 | chr3q12.1 |
| 1554940_a_at | hypothetical LOC388882 | LOC388882 | BC036910 | Hs.126178 | chr22q11.23 |
| 1554918_a_at | ATP-binding cassette, sub-family C (CFTR/MRP), member 4 | ABCC4 | BC041560 | Hs.307915 | chr13q32 |
| 1554915_a_at | 2'-phosphodiesterase | 2'-PDE | BC034978 | Hs.151293 | chr3p14.3 |
| 1554906_a_at | M-phase phosphoprotein 6 | MPHOSPH6 | BC029395 | Hs.344400 | chr16q23.3 |
| 1554894_a_at | pterin-4 alpha-carbinolamine dehydratase/dimerization cofactor of hepatocyte nuclear factor 1 alpha (TCF1) 2 | PCBD2 | AF499009 | Hs.519327 | chr5q31.1 |
| 1554878_a_at | ATP-binding cassette, sub-family D (ALD), member 3 | ABCD3 | BC009712 | Hs.76781 | chr1p22-p21 |
| 1554868_s_at | PEST proteolytic signal containing nuclear protein | PCNP | BC013916 | Hs.170184 | chr3q12.3 |
| 1554863_s_at | docking protein 5 | DOK5 | BC008992 | Hs.127751 | chr20q13.2 |
| 1554821_a_at | zinc finger, BED-type containing 1 | ZBED1 | BC015030 | Hs.458462 | chrxp22.33; yp11 |
| 1554789_a_at | phosphodiesterase 8B | PDE8B | AB085825 | Hs.78106 | chr5q13.3 |
| 1554767_s_at | crystallin, zeta (quinone reductase)-like 1 | CRYZL1 | BC013155 | Hs.352671 | chr21q21.3 |
| 1554762_a_at | WW and C2 domain containing 2 | WWC2 | BC017957 | Hs.257336 | chr4q35.1 |
| 1554747_a_at | septin 2 | septin 2 | BC033559 | Hs.131736 | chr2q37 |
| 1554741_s_at | fibroblast growth factor 7 (keratinocyte growth factor) /// keratinocyte growth factor-like protein 1 /// keratinocyte growth factor-like protein 2 | FGF7 | AF523265 | Hs.433252 | chr15q15-q21.1 /// chr9q12 |
| 1554726_at | zinc finger protein 655 | ZNF655 | AY099353 | Hs.293392 | chr7q22.1 |
| 1554721_a_at | TAF2 RNA polymerase II, TATA box binding protein (TBP)-associated factor, 150kDa | TAF2 | AF040701 | Hs.122752 | chr8q24.12 |
| 1554701_a_at | TBC1 domain family, member 16 | TBC1D16 | BC028290 | Hs.458300 | chr17q25.3 |
| 1554697_at | ADAM metallopeptidase with thrombospondin type 1 motif, 9 | ADAMTS9 | AF488803 | Hs.127811 | chr3p14.3-p14.2 |
| 1554691_a_at | protein kinase C and casein kinase substrate in neurons 2 | PACSIN2 | BC008037 | Hs.454530 | chr22q13.2-13.33 |
| 1554690_a_at | transforming, acidic coiled-coil containing protein 1 | TACC1 | BC041391 | Hs.279245 | chr8p11 |
| 1554679_a_at | lysosomal associated protein transmembrane 4 beta | LAPTM4B | AF317417 | Hs.296398 | chr8q22.1 |
| 1554667_s_at | methyltransferase like 8 | METTL8 | BC025250 | Hs.135146 | chr2q31.1 |
| 1554661_s_at | chromosome 1 open reading frame 71 | C1orf71 | BC036200 | Hs.288742 | chr1q44 |
| 1554628_at | zinc finger protein 57 | ZNF57 | BC028974 | Hs.442538 | chr19p13.3 |
| 1554627_a_at | activating signal cointegrator 1 complex subunit 1 | ASCC1 | BC012291 | Hs.112694 | chr10pter-q25.3 |
| 1554609_at | similar to Cytochrome c, somatic | MGC12965 | BC019340 | Hs.292791 | chr11q13.3 |
| 1554606_at | coiled-coil domain containing 100 | CCDC100 | BC040527 | Hs.423367 | chr5q23.2 |
| 1554577_a_at | proteasome (prosome, macropain) 26S subunit, non-ATPase, 10 | PSMD10 | AY057056 | Hs.433559 | chrxq22.3 |
| 1554574_a_at | cytochrome b5 reductase 3 | CYB5R3 | AF361370 | Hs.274464 | chr22q13.2-q13.31 |
| 1554572_a_at | suppressor of variegation 3-9 homolog 2 (Drosophila) | SUV39H2 | BC029360 | Hs.85567 | chr10p13 |
| 1554556_a_at | ATPase, Class VI, type 11B | ATP11B | BC033880 | Hs.432405 | chr3q27 |
| 1554547_at | family with sequence similarity 13, member C1 | FAM13C1 | BC036453 | Hs.311205 | chr10q21.1 |
| 1554518_at | glutathione S-transferase, C-terminal domain containing | GSTCD | BC032942 | Hs.126119 | chr4q24 |
| 1554512_a_at | coiled-coil domain containing 123 | CCDC123 | BC032307 | Hs.317590 | chr19q13.11 |
| 1554509_a_at | chromosome 10 open reading frame 97 | C10orf97 | BC020605 | Hs.285107 | chr10p13 |
| 1554480_a_at | SVH protein | SVH | AY150851 | Hs.269418 | chr7q22.1 |
| 1554472_a_at | PHD finger protein 20-like 1 | PHF20L1 | BC015211 | Hs.44159 | chr8q24.22 |
| 1554470_s_at | zinc finger and BTB domain containing 44 | ZBTB44 | BC030580 | Hs.178499 | chr11q24.3 |
| 1554465_s_at | zinc finger protein 673 /// zinc finger protein 674 | ZNF673 | BC012569 | Hs.116532 | chrxp11.3 |
| 1554464_a_at | cartilage associated protein | CRTAP | BC008745 | Hs.483205 | chr3p22.3 |
| 1554455_at | lines homolog 1 (Drosophila) | LINS1 | BC010363 | Hs.105633 | chr15q26.3 |
| 1554452_a_at | hypoxia-inducible protein 2 | HIG2 | BC001863 | Hs.521171 | chr7q32.2 |
| 1554443_s_at | bestrophin 1 | BEST1 | BC041664 | Hs.167344 | chr11q13 |
| 1554441_a_at | wings apart-like homolog (Drosophila) | WAPAL | AF479418 | Hs.439188 | chr10q23.2 |
| 1554433_a_at | zinc finger protein 146 | ZNF146 | BC005154 | Hs.444223 | chr19q13.1 |
| 1554429_a_at | dystrophia myotonica-containing WD repeat motif | DMWD | BC019266 | Hs.275924 | chr19q13.3 |
| 1554424_at | platelet-derived growth factor receptor, alpha polypeptide /// FIP1 like 1 (S. cerevisiae) | PDGFRA | BC017724 | Hs.66295 | chr4q12 |
| 1554423_a_at | F-box protein 7 | FBXO7 | AF233225 | Hs.5912 | chr22q12-q13 |
| 1554415_at | TAF5-like RNA polymerase II, p300/CBP-associated factor (PCAF)-associated factor, 65kDa | TAF5L | BC041094 | Hs.26782 | chr1q42.13 |
| 1554397_s_at | UEV and lactate/malate dehyrogenase domains | UEVLD | BC011011 | Hs.407991 | chr11p15.1 |
| 1554390_s_at | ARP2 actin-related protein 2 homolog (yeast) | ACTR2 | BC036253 | Hs.393201 | chr2p14 |
| 1554376_s_at | protein tyrosine phosphatase-like (proline instead of catalytic arginine), member A | PTPLA | BC027709 |  |  |
| 1554345_a_at | desmoglein 1 /// zinc finger, H2C2 domain containing | DSG1 | BC015325 | Hs.24088 | chr5q21.1 |
| 1554344_s_at | aquaporin 12A /// similar to aquaporin 12A | AQP12A | AB040748 | Hs.534783 | chr2q37.3 |
| 1554296_at | cytochrome P450, family 19, subfamily A, polypeptide 1 | CYP19A1 | BC035714 | Hs.126074 | chr15q21.1 |
| 1554287_at | tripartite motif-containing 4 | TRIM4 | BC011763 | Hs.439522 | chr7q22-q31.1 |
| 1554271_a_at | centromere protein L | CENPL | BC019022 | Hs.434498 | chr1q25.1 |
| 1554260_a_at | FRY-like | FRYL | BC021803 | Hs.446102 | chr4p12 |
| 1554250_s_at | tripartite motif-containing 73 | TRIM73 | BC033812 | Hs.511015 | chr7q11.23 |
| 1554241_at | coagulation factor C homolog, cochlin (Limulus polyphemus) | COCH | BC007230 | Hs.377466 | chr14q12-q13 |
| 1554229_at | adult retina protein | LOC153222 | AY174896 | Hs.163725 | chr5q35.1 |
| 1554205_s_at | islet cell autoantigen 1,69kDa-like | ICA1L | BC000993 | Hs.516629 | chr2q33.2 |
| 1554171_at | zinc finger, MYM-type 3 | ZMYM3 | BC013009 | Hs.9568 | chrxq13.1 |
| 1554168_a_at | SH3-domain kinase binding protein 1 | SH3KBP1 | AF542051 | Hs.153260 | chrxp22.1-p21.3 |
| 1554167_a_at | golgi autoantigen, golgin subfamily a, 7 | GOLGA7 | BC012032 | Hs.7953 | chr8p11.21 |
| 1554153_a_at | PHD finger protein 21A | PHF21A | BC015714 | Hs.104888 | chr11p11.2 |
| 1554149_at | claudin domain containing 1 | CLDND1 | BC013610 | Hs.107393 | chr3p11-q11 |
| 1554148_a_at | solute carrier family 33 (acetyl-CoA transporter), member 1 | SLC33A1 | BC014416 | Hs.285176 | chr3q25.31 |
| 1554145_a_at | coiled-coil domain containing 128 | CCDC128 | BC040721 | Hs.271916 | chr2p16.3 |
| 1554116_s_at | poly (ADP-ribose) polymerase family, member 11 | PARP11 | BC031073 | Hs.212262 | chr12p13.3 |
| 1554097_a_at | hypothetical LOC554202 | LOC554202 | BC021861 | Hs.458096 |  |
| 1554095_at | similar to proline rich 8 /// similar to proline rich 8 /// similar to proline rich 8 | LOC727871 | BC011923 | Hs.521316 | chr7q36.3 |
| 1554089_s_at | Shwachman-Bodian-Diamond syndrome /// Shwachman-Bodian-Diamond syndrome pseudogene | SBDS | BC010183 | Hs.110445 | chr7q11.21 /// chr7q11.23 |
| 1554085_at | DEAD (Asp-Glu-Ala-Asp) box polypeptide 51 | DDX51 | BC012461 | Hs.445168 | chr12q24.33 |
| 1554082_a_at | nucleolar protein 9 | NOL9 | BC009257 | Hs.59425 | chr1p36.31 |
| 1554080_at | RCD1 required for cell differentiation1 homolog (S. pombe) | RQCD1 | BC007102 | Hs.148767 | chr2q35 |
| 1554079_at | UDP-N-acetyl-alpha-D-galactosamine:polypeptide N-acetylgalactosaminyltransferase-like 4 | GALNTL4 | BC037341 | Hs.501911 | chr11p15.3 |
| 1554067_at | hypothetical protein FLJ32549 | FLJ32549 | BC036246 | Hs.396626 | chr12q14.2 |
| 1554057_at | hypothetical LOC645676 | LOC645676 | BC017347 | Hs.445445 | chr1q22 |
| 1554053_at | serine palmitoyltransferase, long chain base subunit 1 | SPTLC1 | BC007085 | Hs.90458 | chr9q22.2 |
| 1554036_at | zinc finger and BTB domain containing 24 | ZBTB24 | BC036731 | Hs.409876 | chr6q21 |
| 1554029_a_at | KIAA0372 | KIAA0372 | BC030966 | Hs.435330 | chr5q15 |
| 1554016_a_at | chromosome 16 open reading frame 57 | C16orf57 | BC006291 | Hs.408702 | chr16q13 |
| 1554008_at | oncostatin M receptor | OSMR | BC010943 | Hs.238648 | chr5p13.1 |
| 1554004_a_at | Rho-guanine nucleotide exchange factor | RGNEF | BC012946 | Hs.33254 | chr5q13.2 |
| 1553998_at | DMRT-like family C1 /// similar to doublesex and mab-3 related transcription factor 8.1 isoform a | DMRTC1 | BC029799 | Hs.374521 | chrxq13.1 |
| 1553997_a_at | aspartate beta-hydroxylase domain containing 1 | ASPHD1 | BC034275 | Hs.408351 | chr16p11.2 |
| 1553994_at | 5'-nucleotidase, ecto (CD73) | NT5E | BC015940 | Hs.153952 | chr6q14-q21 |
| 1553984_s_at | deoxythymidylate kinase (thymidylate kinase) /// similar to deoxythymidylate kinase (thymidylate kinase) | DTYMK | AF258562 | Hs.367821 | chr2q37.3 |
| 1553978_at | MADS box transcription enhancer factor 2, polypeptide B (myocyte enhancer factor 2B) | MEF2B | BC010931 | Hs.78881 | chr19p12 |
| 1553976_a_at | deleted in a mouse model of primary ciliary dyskinesia | RP11-529I10.4 | BC031695 | Hs.32990 | chr10q24.32 |
| 1553974_at | hypothetical protein LOC128977 | LOC128977 | BC030758 | Hs.433195 | chr22q11.21 |
| 1553962_s_at | ras homolog gene family, member B | RHOB | BI668074 | Hs.406064 | chr2p24 |
| 1553961_s_at | sorting nexin family member 21 | SNX21 | CA447177 | Hs.334538 | chr20q13.12 |
| 1553957_at | zinc finger protein 564 | ZNF564 | AK092760 | Hs.534687 | chr19p13.2 |
| 1553956_at | amyotrophic lateral sclerosis 2 (juvenile) chromosome region, candidate 4 | ALS2CR4 | BG194770 | Hs.12319 | chr2q33.2 |
| 1553954_at | asparagine-linked glycosylation 14 homolog (yeast) | ALG14 | BU682208 | Hs.501321 | chr1p21.3 |
| 1553928_at | ELMO/CED-12 domain containing 2 | ELMOD2 | NM_153702 | Hs.450105 | chr4q31.21 |
| 1553801_a_at | chromosome 14 open reading frame 126 | C14orf126 | NM_080664 | Hs.116014 | chr14q12 |
| 1553780_at | hypothetical protein MGC23270 | MGC23270 | NM_152646 | Hs.352220 | chr14q32.33 |
| 1553749_at | family with sequence similarity 76, member B | FAM76B | NM_144664 | Hs.288304 | chr11q21 |
| 1553736_at | coiled-coil domain containing 131 | CCDC131 | NM_144982 | Hs.435048 | chr12q21.1 |
| 1553719_s_at | zinc finger protein 548 | ZNF548 | NM_152909 |  |  |
| 1553715_s_at | chromosome 16 open reading frame 14 | C16orf14 | NM_032371 | Hs.417710 | chr16p13.3 |
| 1553710_at | hypothetical protein FLJ31659 | FLJ31659 | NM_153027 | Hs.178648 | chr4q32.3 |
| 1553704_x_at | zinc finger protein 791 | ZNF791 | NM_153358 | Hs.346875 | chr19p13.2-p13.13 |
| 1553696_s_at | zinc finger protein 569 | ZNF569 | NM_152484 | Hs.511848 | chr19q13.12 |
| 1553694_a_at | phosphoinositide-3-kinase, class 2, alpha polypeptide | PIK3C2A | NM_002645 | Hs.249235 | chr11p15.5-p14 |
| 1553693_s_at | carbonyl reductase 4 | CBR4 | NM_032783 | Hs.200113 | chr4q32.3 |
| 1553689_s_at | methyltransferase like 6 | METTL6 | NM_152396 | Hs.534646 | chr3p25.1 |
| 1553684_at | peptidylprolyl isomerase (cyclophilin)-like 6 | PPIL6 | NM_173672 | Hs.32234 | chr6q21 |
| 1553679_s_at | vitamin K epoxide reductase complex, subunit 1-like 1 | VKORC1L1 | NM_173517 | Hs.43112 | chr7q11.21 |
| 1553672_at | enabled homolog (Drosophila) | ENAH | NM_145240 | Hs.446693 | chr1q42.12 |
| 1553647_at | chromodomain protein, Y-like 2 | CDYL2 | NM_152342 | Hs.208837 | chr16q23.2 |
| 1553646_at | chromosome X open reading frame 43 | CXorf43 | NM_144657 | Hs.128010 | chrxq21.1 |
| 1553613_s_at | forkhead box C1 | FOXC1 | NM_001453 | Hs.348883 | chr6p25 |
| 1553567_s_at | ATPase type 13A5 | ATP13A5 | NM_173702 |  | chrmitochondrion |
| 1553535_a_at | Ran GTPase activating protein 1 | RANGAP1 | NM_002883 | Hs.183800 | chr22q13 |
| 1553530_a_at | integrin, beta 1 (fibronectin receptor, beta polypeptide, antigen CD29 includes MDF2, MSK12) | ITGB1 | NM_033669 | Hs.287797 | chr10p11.2 |
| 1553528_a_at | TAF5 RNA polymerase II, TATA box binding protein (TBP)-associated factor, 100kDa | TAF5 | NM_139052 | Hs.171625 | chr10q24-q25.2 |
| 1553422_s_at | ataxin 2-binding protein 1 | A2BP1 | NM_145892 | Hs.57937 | chr16p13.3 |
| 1553322_s_at | TEA domain family member 1 (SV40 transcriptional enhancer factor) | TEAD1 | NM_021961 | Hs.153408 | chr11p15.4 |
| 1553313_s_at | solute carrier family 5 (inositol transporters), member 3 | SLC5A3 | NM_006933 | Hs.534372 | chr21q22.12 |
| 1553304_at | LSM14B, SCD6 homolog B (S. cerevisiae) | LSM14B | NM_144703 | Hs.105379 | chr20q13.33 |
| 1553299_at | hypothetical protein FLJ31401 | RP4-621O15.2 | NM_152664 | Hs.511905 | chr1q42.13 |
| 1553288_a_at | hypothetical protein FLJ37538 | FLJ37538 | NM_173564 | Hs.320086 | chr7q22.1 |
| 1553286_at | zinc finger protein 555 | ZNF555 | NM_152791 | Hs.12471 | chr19p13.3 |
| 1553252_a_at | bromodomain and WD repeat domain containing 3 | BRWD3 | NM_153252 | Hs.134880 | chrxq21.1 |
| 1553220_at | amyotrophic lateral sclerosis 2 (juvenile) chromosome region, candidate 13 | ALS2CR13 | NM_173511 | Hs.283707 | chr2q33.2 |
| 1553218_a_at | zinc finger protein 512 | ZNF512 | NM_032434 | Hs.294122 | chr2p23 |
| 1553193_at | zinc finger protein 441 | ZNF441 | NM_152355 | Hs.134713 | chr19p13.2 |
| 1553185_at | RAS and EF-hand domain containing | RASEF | NM_152573 | Hs.129136 | chr9q21.32 |
| 1553181_at | DEAD (Asp-Glu-Ala-Asp) box polypeptide 31 | DDX31 | NM_138620 | Hs.133082 | chr9q34.13 |
| 1553172_at | zinc finger protein 777 | ZNF777 | NM_015694 | Hs.145661 | chr7q36.1 |
| 1553162_x_at | chromosome 19 open reading frame 55 | C19orf55 | NM_144692 | Hs.505040 | chr19q13.12 |
| 1553155_x_at | ATPase, H+ transporting, lysosomal 38kDa, V0 subunit d2 | ATP6V0D2 | NM_152565 | Hs.436360 | chr8q21.13 |
| 1553148_a_at | sorting nexin 13 | SNX13 | R75838 | Hs.269881 | chr7p21.1 |
| 1553142_at | chromosome 13 open reading frame 31 | C13orf31 | NM_153218 | Hs.210586 | chr13q14.11 |
| 1553117_a_at | serine/threonine kinase 38 | STK38 | NM_007271 | Hs.367811 | chr6p21 |
| 1553113_s_at | cyclin-dependent kinase 8 | CDK8 | BF700678 | Hs.397734 | chr13q12 |
| 1553111_a_at | kelch repeat and BTB (POZ) domain containing 6 | KBTBD6 | NM_152903 | Hs.112198 | chr13q14.11 |
| 1553108_at | chromosome 5 open reading frame 24 | C5orf24 | AW068615 | Hs.406549 | chr5q31.1 |
| 1553105_s_at | desmoglein 2 | DSG2 | NM_001943 | Hs.412597 | chr18q12.1 |
| 1553102_a_at | coiled-coil domain containing 69 | CCDC69 | BC016647 | Hs.132994 | chr5q33.1 |
| 1553101_a_at | alkB, alkylation repair homolog 5 (E. coli) | ALKBH5 | NM_017758 | Hs.356770 | chr17p11.2 |
| 1553099_at | tigger transposable element derived 1 | TIGD1 | NM_145702 | Hs.534703 | chr2q37.1 |
| 1553048_a_at | phosphatidylinositol-4-phosphate 5-kinase, type II, beta | PIP5K2B | NM_138687 | Hs.6335 | chr17q12 |
| 1553034_at | phosphodiesterase 9A | PDE9A | NM_006642 | Hs.511758 | chr1q44 |
| 1553027_a_at | kelch-like 4 (Drosophila) | KLHL4 | NM_057162 | Hs.49075 | chrxq21.3 |
| 1552911_at | sialic acid binding Ig-like lectin 11 | SIGLEC11 | AB072038 | Hs.269347 | chr19q13.33 |
| 1552897_a_at | potassium voltage-gated channel, subfamily G, member 3 | KCNG3 | NM_133329 | Hs.352633 | chr2p21 |
| 1552863_a_at | calcium channel, voltage-dependent, gamma subunit 6 | CACNG6 | NM_145815 | Hs.326764 | chr19q13.4 |
| 1552848_a_at | patched domain containing 1 | PTCHD1 | NM_173495 | Hs.319503 | chrxp22.11 |
| 1552789_at | translocation protein 1 | TLOC1 | NM_153039 | Hs.283326 | chr3q26.31 |
| 1552785_at | zinc finger protein 781 | ZNF781 | NM_152605 | Hs.325834 | chr19q13.12 |
| 1552769_at | zinc finger protein 625 | ZNF625 | NM_145233 | Hs.528709 | chr19p13.2 |
| 1552733_at | kelch domain containing 1 | KLHDC1 | NM_172193 | Hs.509258 | chr14q21.3 |
| 1552727_s_at | ADAM metallopeptidase with thrombospondin type 1 motif, 17 | ADAMTS17 | AA022668 | Hs.498479 |  |
| 1552711_a_at | cytochrome b5 domain containing 1 | CYB5D1 | NM_144607 | Hs.27475 | chr17p13.1 |
| 1552696_at | non imprinted in Prader-Willi/Angelman syndrome 1 | NIPA1 | NM_144599 | Hs.352617 | chr15q11.2 |
| 1552670_a_at | hypothetical protein LOC286044 | LOC286044 | NM_024607 |  | chr8p23.1 |
| 1552665_at | hypothetical protein MGC14425 | MGC14425 | NM_032903 |  | chr10q22.1 |
| 1552664_at | folliculin | FLCN | NM_144997 | Hs.396533 | chr17p11.2 |
| 1552644_a_at | polyhomeotic homolog 3 (Drosophila) | PHC3 | NM_024947 | Hs.25328 | chr3q26.2 |
| 1552633_at | zinc finger protein 101 | ZNF101 | NM_033204 | Hs.164284 | chr19p13.11 |
| 1552622_s_at | DNA directed RNA polymerase II polypeptide J-related gene /// similar to postmeiotic segregation increased 2-like 2 /// similar to postmeiotic segregation increased 2-like 2 | POLR2J2 | BQ613856 | Hs.455206 /// Hs.489481 /// Hs.520965 | chr7q11-q22 /// chr7q11.22 /// chr7q11.23 |
| 1552617_a_at | ring finger and WD repeat domain 2 | RFWD2 | NM_022457 | Hs.412957 | chr1q25.1-q25.2 |
| 1552613_s_at | CDC42 small effector 2 | CDC42SE2 | NM_020240 |  |  |
| 1552610_a_at | Janus kinase 1 (a protein tyrosine kinase) | JAK1 | NM_002227 | Hs.309244 | chr1p32.3-p31.3 |
| 1552485_at | lactamase, beta | LACTB | NM_171846 | Hs.410388 | chr15q22.1 |
| 1552481_s_at | mannosidase, alpha, class 1A, member 2 | MAN1A2 | NM_006699 | Hs.367638 | chr1p13 |
| 1552426_a_at | TM2 domain containing 3 | TM2D3 | NM_078474 | Hs.288912 | chr15q26.3 |
| 1552423_at | ets variant gene 3 | ETV3 | NM_005240 | Hs.352672 | chr1q21-q23 |
| 1552370_at | chromosome 4 open reading frame 33 | C4orf33 | NM_173487 | Hs.128777 | chr4q28.2 |
| 1552344_s_at | CCR4-NOT transcription complex, subunit 7 | CNOT7 | NM_054026 | Hs.170553 | chr8p22-p21.3 |
| 1552334_at | TRIO and F-actin binding protein | TRIOBP | BG571343 |  |  |
| 1552323_s_at | family with sequence similarity 122C | FAM122C | NM_138819 | Hs.192806 | chrxq26.3 |
| 1552310_at | chromosome 15 open reading frame 40 | C15orf40 | NM_144597 | Hs.352541 | chr15q25.2 |
| 1552302_at | transmembrane protein 106A /// similar to transmembrane protein 106A | TMEM106A | NM_145041 | Hs.446690 | chr17q21.31 |
| 1552301_a_at | coronin 6 | CORO6 | NM_032854 | Hs.143046 | chr17q11.2 |
| 1552287_s_at | AFG3 ATPase family gene 3-like 1 (S. cerevisiae) | AFG3L1 | NM_001132 | Hs.114318 | chr16q24.3 |
| 1552279_a_at | proton-coupled folate transporter | PCFT | AK074161 | Hs.534621 | chr17q11.2 |
| 1552277_a_at | chromosome 9 open reading frame 30 | C9orf30 | NM_080655 | Hs.162669 | chr9q31.1 |
| 1552275_s_at | PX domain containing serine/threonine kinase | PXK | BG573647 | Hs.77646 | chr3p14.3 |
| 1552257_a_at | tubulin tyrosine ligase-like family, member 12 | TTLL12 | NM_015140 | Hs.82563 | chr22q13.31 |
| 1487_at | estrogen-related receptor alpha | ESRRA | L38487 | Hs.110849 | chr11q13 |
| 1294_at | ubiquitin-activating enzyme E1-like | UBE1L | L13852 | Hs.16695 | chr3p21 |
| 121_at | paired box gene 8 | PAX8 | X69699 | Hs.308061 | chr2q12-q14 |
| 1053_at | replication factor C (activator 1) 2, 40kDa | RFC2 | M87338 | Hs.139226 | chr7q11.23 |
| 1007_s_at | discoidin domain receptor family, member 1 | DDR1 | U48705 | Hs.423573 | chr6p21.3 |

Table SIV: detailed list of the genes presented in the network (Supplementary Figure SII)

| **Symbol** | **Gene Name** | **Location** | **Type(s)** |
| --- | --- | --- | --- |
| AKT1 | v-akt murine thymoma viral oncogene homolog 1 | Cytoplasm | kinase |
| APP | amyloid beta (A4) precursor protein | Plasma Membrane | other |
| BAX | BCL2-associated X protein | Cytoplasm | transporter |
| CFLAR | CASP8 and FADD-like apoptosis regulator | Cytoplasm | other |
| CRYAB | crystallin, alpha B | Nucleus | other |
| EP300 | E1A binding protein p300 | Nucleus | transcription regulator |
| FYN | FYN oncogene related to SRC, FGR, YES | Plasma Membrane | kinase |
| GLUL | glutamate-ammonia ligase | Cytoplasm | enzyme |
| GRB2 | growth factor receptor-bound protein 2 | Cytoplasm | other |
| GSK3B | glycogen synthase kinase 3 beta | Nucleus | kinase |
| HBA1/HBA2 | hemoglobin, alpha 1 | Cytoplasm | transporter |
| HSD17B10 | hydroxysteroid (17-beta) dehydrogenase 10 | Cytoplasm | enzyme |
| HSP90AA1 | heat shock protein 90kDa alpha (cytosolic), class A member 1 | Cytoplasm | enzyme |
| HSPA8 | heat shock 70kDa protein 8 | Cytoplasm | enzyme |
| HSPD1 | heat shock 60kDa protein 1 (chaperonin) | Cytoplasm | enzyme |
| IL8 | interleukin 8 | Extracellular Space | cytokine |
| IL1B | interleukin 1, beta | Extracellular Space | cytokine |
| ITM2B | integral membrane protein 2B | Plasma Membrane | other |
| MAP2 | microtubule-associated protein 2 | Cytoplasm | other |
| MIF | macrophage migration inhibitory factor (glycosylation-inhibiting factor) | Extracellular Space | cytokine |
| NFKB1 | nuclear factor of kappa light polypeptide gene enhancer in B-cells 1 | Nucleus | transcription regulator |
| NSF | N-ethylmaleimide-sensitive factor | Cytoplasm | transporter |
| PGAM1 | phosphoglycerate mutase 1 (brain) | Cytoplasm | phosphatase |
| PPIA | peptidylprolyl isomerase A (cyclophilin A) | Cytoplasm | enzyme |
| PPP1R12A | protein phosphatase 1, regulatory subunit 12A | Cytoplasm | phosphatase |
| PSEN1 | presenilin 1 | Plasma Membrane | peptidase |
| PSENEN | presenilin enhancer 2 homolog (C. elegans) | Cytoplasm | peptidase |
| PTGS2 | prostaglandin-endoperoxide synthase 2 (prostaglandin G/H synthase and cyclooxygenase) | Cytoplasm | enzyme |
| RELA | v-rel reticuloendotheliosis viral oncogene homolog A (avian) | Nucleus | transcription regulator |
| RPL10 | ribosomal protein L10 | Cytoplasm | other |
| SERPINA3 | serpin peptidase inhibitor, clade A (alpha-1 antiproteinase, antitrypsin), member 3 | Extracellular Space | other |
| SHC1 (includes EG:20416) | SHC (Src homology 2 domain containing) transforming protein 1 | Cytoplasm | kinase |
| SLC40A1 | solute carrier family 40 (iron-regulated transporter), member 1 | Plasma Membrane | transporter |
| STXBP1 | syntaxin binding protein 1 | Cytoplasm | transporter |
| UCHL1 | ubiquitin carboxyl-terminal esterase L1 (ubiquitin thiolesterase) | Cytoplasm | peptidase |
